# Identification of the *Schistosoma mansoni* TNF-Alpha Receptor Gene and the Effect of Human TNF-Alpha on the Parasite Gene Expression Profile

**DOI:** 10.1371/journal.pntd.0000556

**Published:** 2009-12-01

**Authors:** Katia C. Oliveira, Mariana L. P. Carvalho, Thiago M. Venancio, Patricia A. Miyasato, Toshie Kawano, Ricardo DeMarco, Sergio Verjovski-Almeida

**Affiliations:** 1 Laboratory of Gene Expression in Eukaryotes, Departamento de Bioquímica, Instituto de Química, Universidade de São Paulo, São Paulo, Brazil; 2 Laboratory of Parasitology, Instituto Butantan, São Paulo, Brazil; 3 Departamento de Física e Informática, Instituto de Física de São Carlos, Universidade de São Paulo, São Carlos, Brazil; Uniformed Services University, United States of America

## Abstract

**Background:**

*Schistosoma mansoni* is the major causative agent of schistosomiasis. The parasite takes advantage of host signals to complete its development in the human body. Tumor necrosis factor-alpha (TNF-α) is a human cytokine involved in skin inflammatory responses, and although its effect on the adult parasite's metabolism and egg-laying process has been previously described, a comprehensive assessment of the TNF-α pathway and its downstream molecular effects is lacking.

**Methodology/Principal Findings:**

In the present work we describe a possible TNF-α receptor (TNFR) homolog gene in *S. mansoni* (*Sm*TNFR). *Sm*TNFR encodes a complete receptor sequence composed of 599 amino acids, and contains four cysteine-rich domains as described for TNFR members. Real-time RT-PCR experiments revealed that *Sm*TNFR highest expression level is in cercariae, 3.5 (±0.7) times higher than in adult worms. Downstream members of the known human TNF-α pathway were identified by an *in silico* analysis, revealing a possible TNF-α signaling pathway in the parasite. In order to simulate parasite's exposure to human cytokine during penetration of the skin, schistosomula were exposed to human TNF-α just 3 h after cercariae-to-schistosomula *in vitro* transformation, and large-scale gene expression measurements were performed with microarrays. A total of 548 genes with significantly altered expression were detected, when compared to control parasites. In addition, treatment of adult worms with TNF-α caused a significantly altered expression of 1857 genes. Interestingly, the set of genes altered in adults is different from that of schistosomula, with 58 genes in common, representing 3% of altered genes in adults and 11% in 3 h-old early schistosomula.

**Conclusions/Significance:**

We describe the possible molecular elements and targets involved in human TNF-α effect on *S. mansoni*, highlighting the mechanism by which recently transformed schistosomula may sense and respond to this host mediator at the site of cercarial penetration into the skin.

## Author Summary

*Schistosoma mansoni* is the major causative agent of schistosomiasis in the Americas. This parasite takes advantage of host signaling molecules such as cytokines and hormones to complete its development inside the host. Tumor necrosis factor-alpha (TNF-α) is one of the most important host cytokines involved in the inflammatory response. When cercariae, the infective stage, penetrates the human skin the release of TNF-α is started. In this work the authors describe the complete sequence of a possible TNF-α receptor in *S. mansoni* and detect that the receptor is most highly expressed in cercariae among all life cycle stages. Aiming to mimic the situation at the site of skin penetration, cercariae were mechanically transformed *in vitro* into schistosomula and exposed to human TNF-α. Exposure of early-developing schistosomula to the human hormone caused a large-scale change in the expression of parasite genes. Exposure of adult worms to human TNF-α caused gene expression changes as well, and the set of parasite altered genes in the adult parasite was different from that of schistosomula. This work increases the number of known signaling pathways of the parasite, and opens new perspectives into understanding the molecular components of TNF-α response as well as into possibly interfering with parasite–host interaction.

## Introduction

Schistosomiasis is a public health problem in many developing countries, and *Schistosoma mansoni* is one of the most widespread species of the causative trematode parasite, affecting 200 million people in the world. Parasite eggs laid in the hepatic portal vasculature are the principal cause of morbidity, and the resulting pathology may prove fatal [1].

The complex parasite life cycle in the vertebrate host begins when cercariae penetrate the human host skin, transforms into schistosomula and begin their life as endoparasites. To continue their development schistosomula take advantage of signals from the host; co-evolution has allowed the schistosome parasite to sense and respond to host mediators [2]. One of the important responses of human host at the site of cercariae penetration in the skin is TNF-α (Tumor necrosis factor alpha) cytokine production in the early steps of the inflammatory process, along with the synthesis of other cytokines such as IL-1, IL-8, TGF-β, IFN-γ [3],[4].

Human cytokines such as IL-7 and TGF-β have been described as host signals that interfere with the metabolism, gene expression and development of schistosomes [2],[5]. The effects of TNF-α on *S. mansoni* metabolism, development and egg-laying process have been described a long time ago [6]–[8]; Amiri *et al* reported that TNF-α induces liver granulomas and egg-laying of parasites *in vivo* [6]; additionally, Cheever *et al* [8] observed that egg laying and fecundity were delayed when SCID immuno deficient schistosome-infected mice were studied, suggesting the possibility that the delay in fecundity is due to a delay in tissue produced TNF-α in SCID mice [8]; nevertheless, their data provided little evidence that TNF-α alone can reconstitute early fecundity in this infected-mouse model [8]. Conversely, Haseeb *et al* showed that in *S. mansoni* females the egg-laying process is decreased and tyrosine up-take is increased in the presence of TNF-α [7]. There is controversial evidence of TNF-α influence on the parasite's metabolism, and the molecular mechanisms of its action have never been explored.

The TNF receptor (TNFR) superfamily comprises membrane bound or soluble receptors which interact with one or more specific ligands. Currently more than 40 members of the TNFR superfamily have been identified in humans [9]. The TNF-like receptors are transmembrane proteins characterized by extra-cellular Cysteine-Rich domains (CRD) that are the hallmark of the TNFR superfamily. These pseudo-repeats are defined by intrachain disulphides, typically having highly conserved cysteine residues within the receptor chains. Significant variation in the number of CRDs exists among the receptor family members, with the most common structure bearing four CRDs and other members having from one up to six CRDs [9]–[11]. TNFR superfamily can be divided into three groups: (i) the death receptors, which mediate cell death through their cytoplasmic Death Domain, with TNFR1 as the typical member; (ii) the non-death receptors, which signal mostly through one or more of the TNF receptor-associated factors (TRAFs), TNFR2 being the typical member; and (iii) decoy receptors which bind TNF-related apoptosis-inducing ligands but may prevent ligand-mediated apoptosis through a number of different mechanisms [12]. Until this moment, no member of the TNFR superfamily has ever been described in *S. mansoni*.

Recent advances on schistosome genomics have led to considerable progress in understanding of the complex molecular mechanisms controlling the life cycle of this helminth parasite. Publication of large-scale sequence databases of both *S. mansoni* and *S. japonicum* transcriptomes in 2003 provided the first large repository of schistosome genes and brought insights into several aspects of schistosome biology [13],[14]. A large-scale joint effort to sequence the *S. mansoni* genome is under way [15],[16] and a draft of the assembled sequences is accessible at the project website. One of the post-genomic approaches that have been explored in schistosomes is the use of microarrays to perform large-scale studies of gene expression at different stages of the life cycle [17]–[20] and gender associated expression profiles [21]–[25]. However, no studies have looked at the effect of hormones or cytokines on large-scale gene expression in the parasite.

In the present work we describe the homolog to human TNF-α receptor and the possible TNF-α downstream signaling pathway elements in *S. mansoni*; we also evaluate the in vitro effect of human TNF-α on the gene expression profile of *S. mansoni* adult parasites along with the effect of TNF-α on schistosomula just 3 hours after cercariae-to-schistosomula *in vitro* transformation.

## Materials and Methods

### Amplification of *Sm*TNFR transcript by PCR and RACE experiments

Total RNA (1 µg) was extracted from BH strain adult worms using Trizol reagent (Invitrogen, Life Technologies Inc., Carlsbad, CA, USA) and then treated with DNAse I (QIAGEN, Hilden, Germany). Total RNA was used for reverse transcription with SuperScript III First Strand Synthesis SuperMix (Invitrogen), using 50 ng of random hexamers according to manufacturer's instructions. The PCR step was performed with Advantage II polymerase (Clontech, Mountain View, CA, USA) with buffer supplied by the manufacturer, 2 µl of reverse transcription reaction and 200 nM of each primer using the following program: 95°C (1min); 40 cycles of 95°C (30 s), 49 to 57°C (30 s), depending on the primer pair combination used, and 68°C (5min); final extension of 68°C (5min). Primers used are listed in **Table S1**, **part A**.

Rapid Amplification of cDNA Ends (RACE) 3′kit (Invitrogen) and RACE 5′kit (Invitrogen) were used according to manufacturer's instructions. The PCR step was performed as described above. Products from all PCR experiments were cloned into pGem-T vector (Promega, Madison, WI, USA) transformed into *E. coli* and stocked in TB-ampicillin. Selected clones were sequenced and the sequences were analyzed and assembled using PhredPhrap program [26]. The resulting *Sm*TNFR sequence has been deposited in GenBank with accession number GQ222226.

### Real-time RT-PCR experiments

Total RNA (3 µg) from each sample was treated with DNAse I (QIAGEN) and purified using RNeasy Mini kit (QIAGEN) according to the manufacturer's instructions. 1.5 µg of total RNA were used as template to perform reverse transcription with random hexamer primers, 200 units of SuperScript III (Invitrogen) reverse transcriptase for 10 min at 25°C, 50 min at 50°C followed by 5 min at 85°C. 1 µl of Rnase H was added to the reaction and incubated for 20 min at 37°C. A parallel negative control reaction was carried out with addition of all components except reverse transcriptase. Each resulting cDNA sample was assayed by real-time PCR in triplicate reactions using gene-specific primers that were designed with the Primer Express program (V2.0) with default parameters (Applied Biosystems, Life Technologies Inc., Carlsbad, CA, USA). Reactions were carried out with SybrGreen PCR core reagent (Applied Biosystems) for 40 cycles in a volume of 20 µl and according to the manufacturer's instructions using the GenAmp5700 sequence detector (Applied Biosystems). Tubulin was used as internal standard gene in expression measurements among parasite's life cycle stages. Gene specific primers used are described in **Table S1, part A**. The results were analyzed by comparative CT method [27], and the statistical significance among expression changes in the expression level was calculated using ANOVA followed by Tukey Range Test [28].

### *In silico* analysis of members of TNF-α signal transduction pathway in *S. mansoni*

Human protein sequences of TNF-α signal transduction pathway elements were used as queries to perform a search in the *S. mansoni* genome (downloaded from the Sanger Institute website; ftp://ftp.sanger.ac.uk/pub/pathogens/Schistosoma/mansoni/genome) using the locally installed copy of the genome sequence and the tBLASTn algorithm. A similar search with Blastp was performed against gene predictions at *Schisto* GeneDB website (available at http://www.genedb.org/genedb/smansoni). *S. mansoni* ESTs available at GenBank were assembled into EST contigs with Cap3 [29] (contig sequences are available in a FASTA format in supplementary **File S1**) and were searched with Human protein sequences of TNF-α signal transduction pathway elements as queries, using tBLASTn algorithm. The *S. mansoni* contig sequences obtained or their translated sequences were queried back to the GenBank protein dataset with BLASTx or BLASTp in order to confirm their identities with the respective element used for the initial search. Sequences with BLASTx or BLASTp best hit *e-value*<10^−5^ were considered as putative *S. mansoni* homologs of TNF-α signaling elements. For *Sm*TNFR translated protein, a signal peptide was predicted with SignalP algorithm [30] and transmembrane helix domains were predicted with the TMHMM-2.0 algorithm [31] available at http://www.cbs.dtu.dk/services/TMHMM-2.0/. Conserved protein domains were identified using PFAM algorithm [32] (available at http://pfam.sanger.ac.uk/), Interpro algorithms [33] (available at http://www.ebi.ac.uk/Tools/InterProScan/) and SMART database [34] (available at http://smart.embl-heidelberg.de/).

### Phylogenetic analysis of TNFR/NGFR sequences

Sequences corresponding to the TNFR conserved domain (cd00185) from 17 different species were aligned along with *Sm*TNFR sequence using ClustalX. Sequence alignment was imported to MEGA 4.0 [35] and the evolutionary history was inferred using the Neighbor-Joining method. The percentage of replicate trees in which the associated taxa clustered together in the bootstrap test (100 replicates) was calculated. Evolutionary distances were computed using the Poisson correction method and are in the units of the number of amino acid substitutions per site. All positions containing gaps and missing data were eliminated from the dataset (Complete deletion option). There were a total of 96 positions in the final dataset. Accession numbers for the sequences used in the alignment are: AAX43474, NP_035741.2, XP_522334.2, NP_777099.1, AAA40465.1, NP_001025950.1, EDL29730.1, NP_001035580.1, NP_001057.1, XP_548191.2, NP_001095948.1, AAD17943.1, P18519.1, XP_001339291.2, XP_002224391.1, XM_001625712.1, FF473364.1.

### Schistosomula and adult worm *in vitro* treatment with human TNF-α

Schistosomula were obtained by mechanical transformation as described by Basch *et al* [36], transformed schistosomula were incubated during 3 h (37°C, 5% CO_2_) in 11 ml M-169 medium containing 10% bovine fetal serum. Then 20 ng/ml human TNF-α (Sigma; stock solution dissolved at 100 µg/ml in 10 mM Tris-Cl pH 8.0) were added to the cultures according to [7] and incubated during 1 h (37°C, 5% CO_2_). Negative control schistosomula were incubated in parallel in 11 ml M-169 containing 10% bovine fetal serum with the vehicle (2.2 µL 10mM Tris-Cl pH8.0). Three independent biological replicas of schistosomula treated with human TNF-α during 1 h with respective control were obtained (in a total of 6 samples).

Adult parasites were obtained from portal vein perfusion of hamsters 7–8 weeks after infection. After perfusion the worms were washed in PBS and only paired worms were incubated during 1 h, and 24 h (37°C, 5% CO_2_) in 30 ml RPMI containing 10% bovine fetal serum with 20 ng/ml human TNF-α (Sigma, stock solution dissolved as before) in parallel with negative control worms (incubated in 30 ml RPMI containing 10% bovine fetal serum with the vehicle, 6 µL 10mM Tris-Cl pH8.0). Three independent biological replicas were obtained for each experimental time with respective controls (in a total of 12 samples).

Infected hamsters were maintained at Instituto Adolfo Lutz that approved the study, and the experimental procedures were conducted adhering to the institution's guidelines for animal husbandry.

### Total RNA extraction and microarray experiments

Total RNA was extracted using Trizol reagent (Invitrogen) according to the manufacturer's instructions. After Trizol extraction RNAs were treated with DNAse I (QIAGEN) and subsequently purified using RNA easy mini kit (QIAGEN). The integrity of RNA samples was evaluated using microfluidic electrophoresis in the Bioanalyzer equipment (Agilent Technologies, Santa Clara, CA, USA).

The oligoarray platform used in this work was previously described by Verjovski-Almeida *et al* [37] for the experiments using adult worm samples. A new platform, 4×44k, containing the same probe set, but a different control set was used for the experiments with 3-hour old early schistosomula. For each of the two platforms, the control set is the one recommended by Agilent, according to the probe disposition on the array. The platforms contain the same set of 44000 oligonucleotide probes that were designed by our group to represent all *S. mansoni* gene fragments available in the public GenBank database; slides were manufactured for us by Agilent Technologies. The microarray platform design along with gene annotation names was deposited at NCBI gene expression omnibus (GEO) under accession numbers GPL4791 (1×44K) and GPL8606 (4×44K). MIAME compliant data were deposited under accession numbers GSE16261 (adults) and GSE16260 (schistosomula). **Table S1**, part B contains the oligoarray probe numbers, the corresponding ESTs contig represented by each probe, the predicted protein Smp_xxxxxx number when available (see annotation method below), and the putative human homolog (E-value <1e-20) Accession number when available (see below).

Either 230 ng from each RNA sample of schistosomula treated for 1 h and their respective control or 300 ng from each RNA from paired adult worms treated for 1 h and 24 h with human TNF-α and their respective negative controls were used for labeling with the Linear RNA amplification and labeling kit (Agilent Technologies) according to manufacturer's instructions. Each sample was separately labeled with either Cy3 or Cy5 and 825 ng cRNA from each amplification were used for hybridization; in experiments with schistosomula a control sample was combined with a treated sample and hybridized; in experiments with adult worm samples Self-Self hybridizations were performed [38]. Washing and scanning procedures were according to the manufacturer's instructions using GenePix 4000B scanner (Molecular Devices, Sunnyvale, CA, USA). Data was extracted using Feature Extraction software (Agilent Technologies).

### Microarray data analysis

Low intensity data points were filtered out according to Feature Extraction software criteria, which essentially determine those points that are significantly below the average background signal of the array. Total intensity data from each experiment were normalized by Quantiles Normalization Method [39], excluding positive and negative external controls. For both experiments (treated schistosomula and treated adult paired worms) Significance Analysis of Microarray (SAM) was used as the statistical test to identify differentially expressed genes [40]. For schistosomula, we performed a z-score transformation of normalized data was performed [41] and SAM two class tests were applied; genes were considered as significantly differentially expressed at *q-value* ≤0.05. For adult worms, we calculated a virtual Log2 ratio between intensities of TNF-α Treated/Control, for each gene. With these ratios, we used two different approaches for SAM statistical analyses. SAM one-class approach was used to identify genes with sustained changes in their expression levels along the entire time period of observation (1 and 24 h); SAM two-class approach was used to identify genes with transient changes in their expression levels. In both cases, genes were considered differentially expressed at *q-value *≤0.05. Hierarchical clustering of selected genes was generated using Spotfire Decision Site software (TIBCO Software Inc., Palo Alto, CA, USA). For a gene that was represented in the array by multiple probes, we picked a single representative probe by selecting the probe with the highest absolute value of the Log2 ratio between intensities of TNF-α Treated/Control.

Gene ontology terms were assigned to genes represented in the array as described by Verjovski-Almeida et al [37]. GO terms enrichment was calculated using Ontologizer program [42]. The *p-values* were adjusted for multiple comparisons using the Benjamini-Hochberg method.

Ingenuity Pathway Analysis software was used for identifying significantly enriched gene networks among the differentially expressed *S. mansoni* genes. For this purpose, *S. mansoni* genes encoding putative homologues to human proteins were identified in the following way. A search of each *S. mansoni* ESTs contig against the gene prediction data set on GeneDB was performed (cutoff  = >90% identity and over 20% coverage of the contig) and a total of 15491 contigs could be associated to Smp_xxxxxx predictions. The 15491probes, representing each of these contigs, correspond to 39% of the 39343 probes on the oligoarray; they comprise a set of 7480 unique Smp entries. Next, the sequence of each of these Smp predictions was aligned to the GenBank human proteins dataset (using BLASTp), with a BLASTp cutoff e-value ≤1×10^−20^; a total of 10036 out of the 15491 probes on the oligoarray that represent *S. mansoni* predicted proteins were associated to human proteins (**Table S1**, **part B**). This set of probes represents 3736 unique human protein-coding genes. The GI number of each human protein putative homolog was associated to the correspondent *S. mansoni* probe (see **Table S1**, **part B**) and the expression data was up-loaded to Ingenuity Pathway Analysis System version 7.6.

## Results

### Identification of *S. mansoni TNF*-α receptor gene sequence

**Figure 1:**
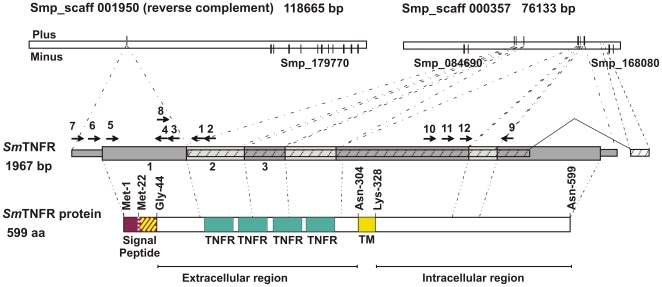
Schematic representation of *Sm*TNFR gene and respective ORF. Each gray bar represents an exon and its respective localization in genome scaffolds is shown. *Sm*TNFR gene is encoded in the Plus strand. The hatched bar represents the original Smp_168070 *in silico* gene prediction. The colored bars represent protein conserved domains of the ORF encoded by *Sm*TNFR gene: Signal peptide region is shown in purple; the white dotted vertical line marks the position of an internal Methionine residue near the translation start site; the yellow hatched lines mark the transmembrane helical domain predicted at the signal peptide region; Gly-44 indicates the predicted start of the mature protein, when cleaved at the predicted cleavage site, between Ala-43 and Gly-44; TNFR (green boxes) mark the Cystein Reach Domains, the hallmark of TNFR superfamily; TM, Transmembrane helical domain. Numbered arrows indicate primers used in RACE and PCR experiments. Other three *in silico* predicted genes in this region of the genome are all encoded in the Minus strand, and they are indicated by Smp_numbers.

Human TNF-α receptor sequence NP_001057.1, tumor necrosis factor receptor 2 precursor [Homo sapiens] was used as query to perform an *in silico* tBLASTn search in the *S. mansoni* genomic DNA sequence downloaded from Welcome Trust Sanger Institute ftp website. With this approach we found genomic scaffold Smp_scaff000357 that contains a 150 bp sequence (from position 38758 to 38907) with 42% identity and 54% similarity to human tumor necrosis factor receptor 2 precursor (NP_001057.1) from amino acid positions Arg-141 to Ala-189. Upon close inspection and a cross reference to protein sequence predictions at *Schisto* GeneDB website (http://www.genedb.org/genedb/smansoni/), a gene prediction (Smp_168070) with a partial sequence was found in scaffold Smp_scaff000357 (see scheme in **Figure 1**), annotated as “tumor necrosis factor receptor related”. This was the only TNF-α receptor sequence found in the entire genome. The 5′-end of this prediction was incomplete and did not contain sequence encoding all extracellular-domain conserved elements of the TNF receptor superfamily. Additionally, its last exon was not correctly predicted as we will show below.

In order to obtain the complete TNF-α receptor sequence in *S. mansoni* we designed three different experiments using total RNA or mRNA as template: (i) cloning the 5′end by 5′-Rapid Amplification of cDNA Ends (5′-RACE) extension (primers used are identified in **Figure 1** as arrows 1 and 2), complemented by PCR reactions with a reverse primer based on the sequence of 5′-RACE extension (primers 3 and 4) and a forward primer based on upstream genome sequence in relation to the 5′end of the RACE-extended message (primers 5 to 7); (ii) cloning the central region based on the predicted Smp_168070 gene sequence and the 5′-RACE extended sequence (primers 8 and 9); and (iii) extending the 3′end by performing 3′-RACE experiments (primers 10 to 12 plus adaptor reverse primer supplied with the RACE kit). **Table S1**, **part A** contains all primers used in these experiments.

The assembled cDNA sequence obtained from all the above experiments resulted in a 1967 bp transcript that was named *Sm*TNFR **(** **Figure 1** **)**. The full-length transcript matches perfectly (with exception of bases 1954 and 1958) to the genome sequence in scaffolds Smp_scaff001950 and Smp_scaff000357. Based on the alignment regions we concluded that the *Sm*TNFR transcript is composed of 7 exons.

The first exon has 408 bp and aligns with genome scaffold Smp_scaff001950 (between coordinates 84375 to 84782 in the minus strand; therefore, for clarity this scaffold is pictured in Figure 1 as the reverse-complement). The second exon has 207 bp (from base 409 to 615 of assembled cDNA) and aligns with genome scaffold Smp_scaff000357 (between coordinates 38695 to 38901 in the plus strand). The third exon has 143 bp (616 to758) and it aligns with the same genomic scaffold between coordinates 39254 to 39396. Exon number four is 206 bp in length (base 759 to 964 of assembled cDNA) and matches the genome sequence between coordinates 41864 to 42069); exon 5 has 441 bp (base 965 to 1405 of assembled cDNA) that matches with genomic coordinates 60952 to 61392; exon 6 has 102 bp (1406 to 1507 bp) and aligns to the genomic scaffold from 61778 to 61879; finally the last and longest exon number 7 has 460 bp and aligns to the genomic scaffold from 62996 to 63467. Donor and acceptor splicing sites are the canonical GT/AG bases for all intron-exon junctions.

Curiously, the genomic sequence between coordinates 63139 and 63202, corresponding to a region within exon 7, is composed of 11 copies of a three-base simple sequence repeat (with sequence AAT). Upon aligning the assembled cDNA to genome scaffold Smp_scaff000357 a gap was detected from 63139 to 63150, which in the genome sequence corresponds to 4 out of the 11 AAT repeats. Consequently, we concluded that our cDNA sequence lacks 4 of the 11 AAT repeats, being characteristic of the parasite BH strain used in the laboratory, which is different from the Puerto Rican strain used for genome sequencing (http://www.tigr.org/tdb/e2k1/sma1/intro.shtml). In addition, cDNA bases 1954 and 1958 at the very 3′-end of our cloned cDNA are the only two bases that disagree with the genomic sequence: in the cDNA they are A's that come from the poly-dT-adaptor reverse primer used for the 3′-RACE and in the genome we found T and G in the respective positions. As discussed later, an internal priming of the message has probably occurred during reverse-transcription at the cDNA synthesis step.

### Identification of *S. mansoni TNF*-α receptor conserved domains

The translated sequence encodes a complete TNFR protein with 599 amino acids, predicted by ORF finder, shown in the scheme of **Figure 1**. A second Met-22 is present in the *Sm*TNFR translated sequence, 21 amino acids downstream of the predicted ORF start (marked by a dotted vertical line in the purple block, **Figure 1**); in case that translation eventually starts there, the receptor would be shorter, with 578 amino acids in length.

A signal peptide was predicted with SignalP 3.0 [30]. For the longer sequence, using the Neural Networks (SignalP-NN) algorithm a signal peptide was predicted within the first 43 amino acids of *Sm*TNFR (max S-score = 0.918; D-score = 0.436). The SignalP Hidden Markov Model (SignalP-HMM) was not able to find a signal peptide motif. For the shorter sequence, SignalP-NN predicted a signal peptide within the first 22 amino acids (max S-score = 0.971; D-score = 0.826); SignalP-HMM predicted the same signal peptide (p = 0.980), within the first 22 amino acids. In all cases, the predicted signal peptide cleavage site is between Ala and Gly residues within the VIA-GPL sequence motif; in the longer sequence the most likely cleavage site is between positions Ala-43 and Gly-44 (SignalP-NN cleavage site C-score = 0.521). In the alternative shorter sequence, the same cleavage site would correspond to positions Ala-22 and Gly-23 in a re-numbered sequence (SignalP-NN cleavage site C-score = 0.923; SignalP-HMM cleavage site probability p = 0.967). The signal peptide is indicated in purple in the scheme of **Figure 1**. Using the TMHMM-2.0 algorithm, a transmembrane helix domain was predicted within the signal peptide domain (see yellow hatched box, in the scheme of **Figure 1**) from amino acids Val-27 to Ile-47 (max membrane probability p = 0.754), thus defining a predicted extracellular N-terminal domain that extends from Gly-44 to Asn-304 in the mature protein, following signal peptide cleavage. A second transmembrane helix domain (yellow box, in the scheme of **Figure 1**) was predicted from amino acids Gln-305 to Tyr-327 (max membrane probability p = 0.945), using the TMHMM-2.0 algorithm.

Four TNFR/NGFR domains (Cystein-Rich Domains, CRD) (green boxes in the scheme of **Figure 1**) were predicted in the extracellular region; these domains were found by PROSITE (PS50050, TNFR_NGFR_2 cysteine-rich region domain) between amino acids Cys-103 to Cys-142 (score = 10.108), Pro-144 to Cys-188 (score = 11.670), Gln-189 to Cys-227 (score = 10.943) and Ser-229 to Cys-269 (score = 11.371).

The intracellular region of the predicted protein encoded by *Sm*TNFR is composed of the C-terminal 272 amino acids extending from Lys-328 to Asn-599 (see scheme in **Figure 1**); no conserved protein domain could be identified in this region, suggesting that *Sm*TNFR belongs to the group of TNFR-superfamily members such as TNFR2 that typically lack the C-terminal Death-Domain motif.

*Sm*TNFR highest similarity to a human TNF-family member is with Tumor necrosis factor receptor superfamily, member 5 isoform 1 precursor, usually called human CD40 (P25942.1, TNR5_HUMAN), a non-death domain receptor, with E-value = 4×10^−9^, 51% similarity and 32% identity over 57% of the *Homo sapiens* protein sequence; highest sequence similarity was present at the 5′-end of the gene, which encodes the conserved ligand-binding N-terminal extracellular domain. According to TreeFam [43] HMMer prediction, *Sm*TNFR belongs to the “Tumor necrosis factor receptor superfamily” (TF331157) with score 233.8. Analysis with Conserved Domain Database tools [44] showed that *Sm*TNFR has a complete Tumor Necrosis Factor Receptor (TNFR) conserved domain (cd00185) (E-value = 5×10^−12^).

**Figure 2:**
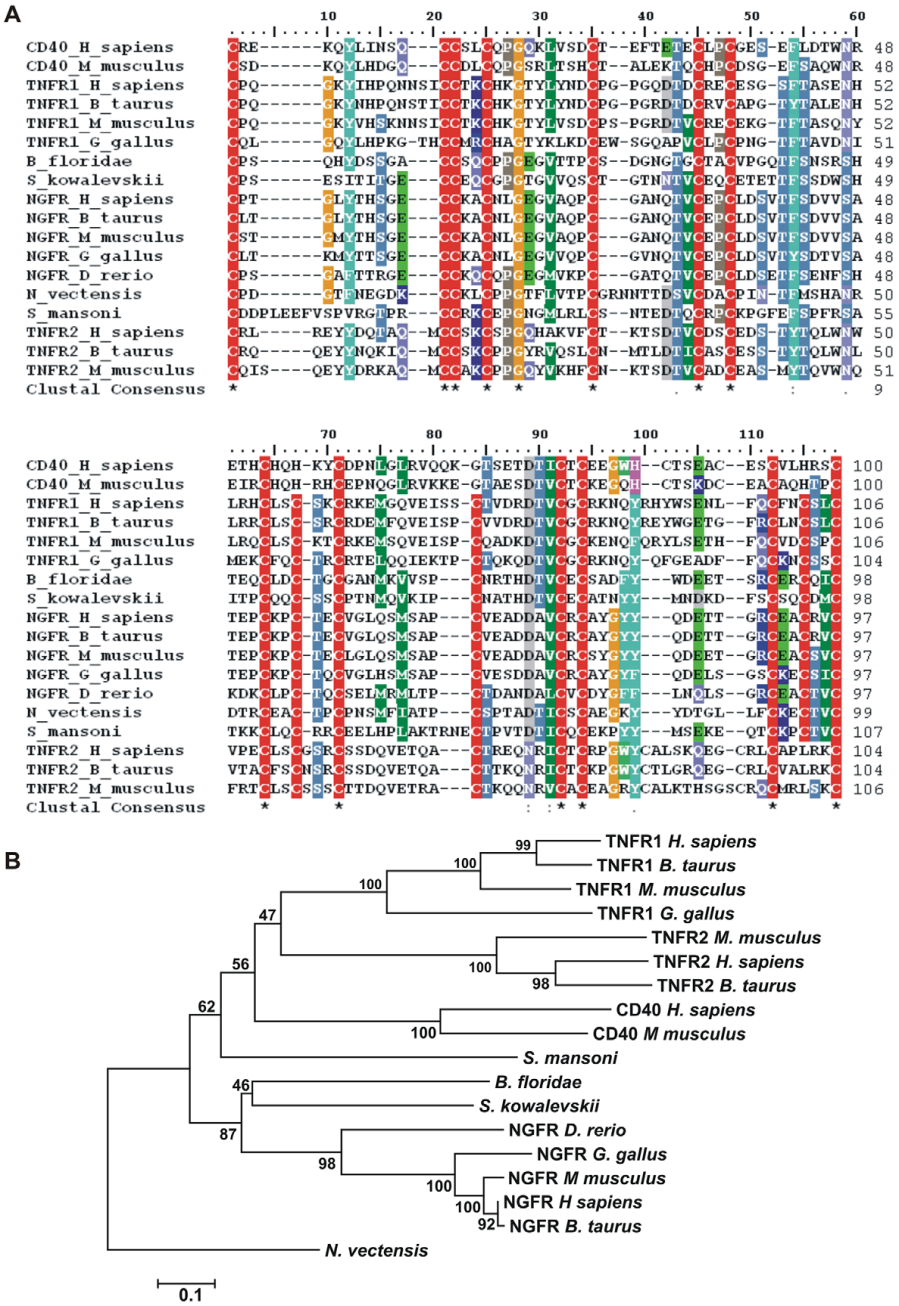
Phylogeny tree of the tumor necrosis factor receptor superfamily. Sequences corresponding to the TNFR conserved domain (cd00185) were (A) aligned using ClustalX, and (B) the evolutionary history of the protein family was inferred using the Neighbor-Joining method, as described under Methods. The percentage of replicate trees in which the associated taxa clustered together in the bootstrap test (100 replicates) is shown for each tree branch. Accession numbers for the sequences used in the analyses are given under Materials and Methods.

A multi-sequence alignment of this conserved domain, including invertebrate and vertebrate TNFR-family members, is shown in **Figure 2A**, where it is seen that the complete conserved cystein-rich TNFR motif is present in *Sm*TNFR. A gene family tree is shown in **Figure 2B**. It can be seen that *Sm*TNFR is placed at a basal position in relation to a branch that includes the vertebrate TNFR1, TNFR2 and CD40 clades, and was separate from the cluster of vertebrate and invertebrate NGFR-family members.

### Expression levels of *Sm*TNFR

**Figure 3:**
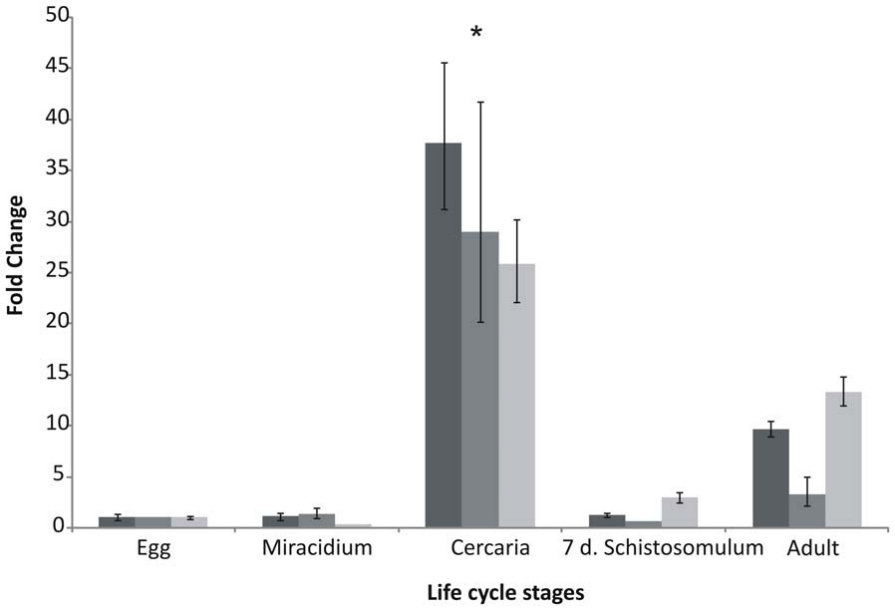
Relative expression levels of *Sm*TNFR among five *S. mansoni* life cycle stages. The different bar colors indicate three independent biological replicas. The expression levels of egg stage were arbitrarily set as 1, and values for other stages are represented as fold changes relative to this value, within the corresponding biological replica. Bars represent standard deviation of the mean for three technical replicates within each biological replica. Tubulin was used as internal normalization gene. ANOVA was used for calculating the statistical significance of expression changes among all life cycle stages (p-*value *<0.0001). Tukey test was used to calculate the significance between two life cycle stages. * *p-value *<0.01 between cercaria and egg, cercaria and miracidium, cercaria and schistosomulum and cercaria and adult comparisons.

Real-time RT-PCR experiments were performed to study the expression level of *Sm*TNFR among life cycle stages. Three independent biological replicas from five different life cycle stages were used (eggs, miracidia, cercariae, 7 day-old schistosomula and adult worms). The highest expression of *Sm*TNFR was detected in cercariae (**Figure 3**), with 3.5 (±0.7) times higher levels than in adult worms, which exhibited the second highest expression. Eggs, miracidia and 7-day old schistosomula had the lowest expression levels of *Sm*TNFR (**Figure 3**).

### Identification of TNF-α signaling pathway elements in *S. mansoni*

Human TNF-α receptors interact with cytoplasmic proteins that activate a signaling cascade composed of multiple protein kinases. In order to evaluate if the TNF-α signal transduction pathway is conserved in *S. mansoni*, we performed an *in silico* analysis using human protein sequences described as involved in the canonical TNF-α signaling pathway [45], aiming to find conserved signaling elements between *S. mansoni* and human.

**Figure 4:**
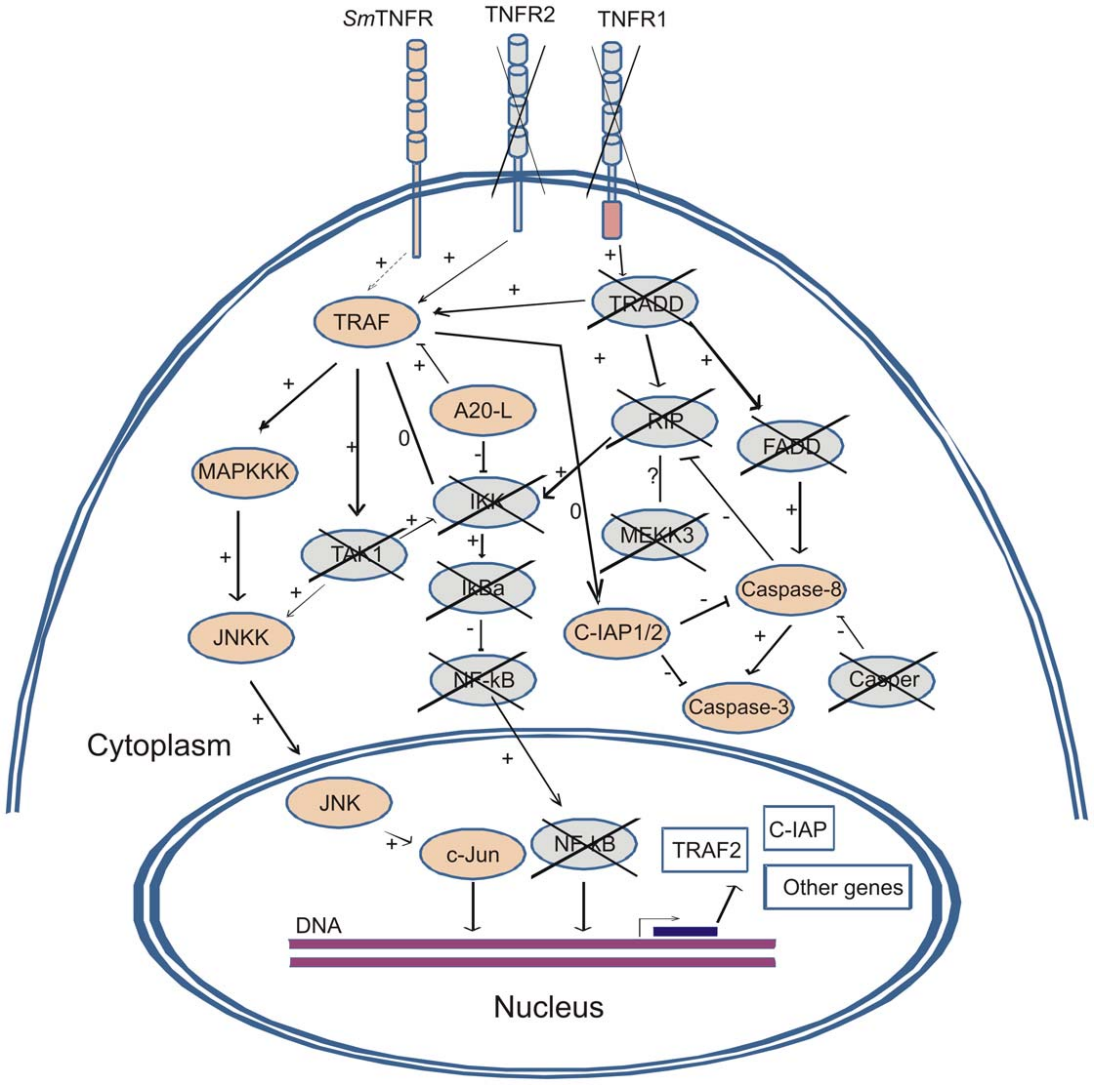
Schematic representation of putative *S. mansoni* TNF signal transduction pathway. Orange elements were *in silico* identified in *S. mansoni*, blue elements were not found. Symbol (→) indicates covalent modification : protein phosphorylation; (}) indicates non-covalent interaction : association : protein/protein. **Relations**: (+), stimulatory; (−), inhibitory; (0), neutral; (?), undefined. Adapted from ref. [45]. Inside the nucleus (rectangles) we have highlighted two examples of messages whose transcriptions are described in humans to be mediated by c-Jun.

We found *S. mansoni* genes potentially encoding 9 signaling elements that participate in the human TNF-α signaling pathway that is activated by TNFR2 (the TNFR group that does not contain the intracellular C-terminal Death Domain). **Figure 4** shows a schematic representation of TNF-α signal transduction pathway conserved elements in *S. mansoni*. **Table S2** contains specific alignment and similarity parameters for each component, including ESTs contigs, gene predictions, mRNAs that encode sequences with similarity to the human proteins and the *S. mansoni* genomic scaffolds where these elements are located. Human TNFR2-activated pathway involves a conserved signaling cascade composed of protein kinases (**Figure 4**). The human receptor interacts with TRAF2 that will activate MAPKKK, which in turn activates JNKK; the latter activates JNK that will phosphorylate and activate c-JUN transcription factor in the nucleus. Homologs of all these elements were found in *S. mansoni* (**Figure 4**). Phosphorylated c-JUN promotes transcription of target genes in the TNF-α signaling pathway. We also found a possible ortholog to A20-like protein (A20-L, Traf-Binding Domain-Containing Protein; TRABID), a protein that interacts with TRAF, along with orthologs to c-IAP (cellular inhibitor of apoptosis protein 1/2), and to caspase-3 and caspase-8, which are related to apoptosis in human cells, especially when activated by signaling through TNFR1.

We note that human MAPKKK is the most difficult element in the pathway for which to predict an ortholog in *S. mansoni* because it refers to two similar kinases, namely MEKK1 (also known as MAPKKK1) and MEKK5 (also known as ASK1 or MAPKKK5), and there are still other very similar MAP kinases [46]. Human MAPKKK5 best hit against *S. mansoni* gene prediction is Smp_162800.1, annotated as “protein kinase”, with E-value = 3×10^−156^, 36% identity, 56% similarity, and 64% coverage of the human MAPKKK5. The best human hit of Smp_162800.1 against GenBank is MAPKKK15, with E-value = 1×10^−160^, 39% identity, 59% similarity, and 64% coverage, while MAPKKK5 is only the second best human hit against GenBank. BLAST search of GenBank using contig C911313.1 gives the same MAPKKK15 and MAPKKK5 hits, although with lower scores than for the full-length predicted *S. mansoni* protein (**Table S2**). We propose that the MAPKKK homolog found in *S. mansoni* could activate JNKK because of the overall context of other signaling elements.

### Effects of human TNF-α on gene expression profile in *S. mansoni* schistosomula

In order to find the possible target genes of *Sm*TNFR signaling pathway we performed microarray experiments with *S. mansoni* parasites treated *in vitro* with human TNF-α.

In the first set of experiments, cercariae were *in vitro* transformed into schistosomula by mechanical removal of their tails [36], and incubated for three hours at 37°C for recovery. Human TNF-α (20 ng/ml) was added to these recently transformed schistosomula, in an attempt to mimic the exposure to TNF-α upon penetration of the human skin. We chose to use the same TNF-α concentration that was previously used in the literature [7] for *in vitro* experiments with adult parasites. The 3-hour old early schistosomula were incubated during 1 h with TNF-α; in parallel a negative control was maintained in the absence of cytokine. Three independent replicate experiments were performed. RNA was extracted from TNF-α treated and non-treated early schistosomula, and processed for microarray hybridization.

**Figure 5:**
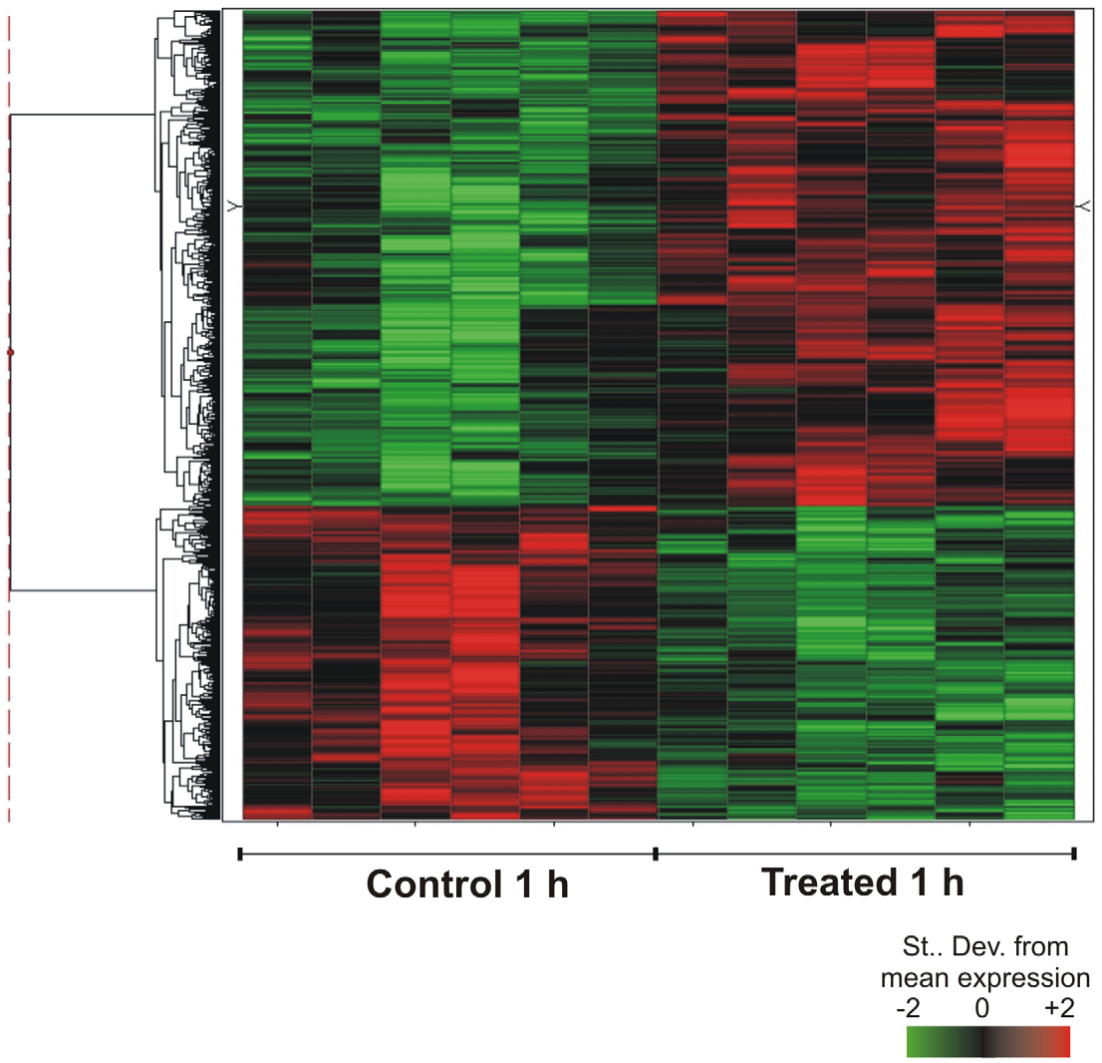
Effect of human TNF-α on gene expression profile in 3-h old early schistosomula. Schistosomula were treated for 1 h with 20 ng/ml human TNF-α just 3 h after in vitro mechanical transformation of cercariae. Microarrays were used for large-scale gene expression measurements, and the figure shows a hierarchical clustering of the 548 genes that exhibited significant (*q-value *≤0.05) changes in their expression levels, in relation to control non-treated 3-h old early schistosomula. Each horizontal line represents a gene; each column represents one experimental replica. There are two technical replicates for each of three biological replicates. For each gene, color intensity is proportional to the number of standard derivations from the mean expression for that gene, as indicated in the scale bar, and shows genes that were induced (red) or repressed (green) by treatment.

A set of 755 probes was identified with a statistically significant (*q-value *<0.05) differential expression between TNF-α treated and control early schistosomula (**Figure 5**). These probes represent 548 unique genes; among them, 309 were induced and 239 were repressed in presence of human TNF-α. **Table S3** shows the number of differentially expressed genes for this 1h-treatment, according to the three annotation categories: *S. mansoni* genes with orthologs in other species, *S. mansoni* genes with orthologs only in *S. japonicum* and in no other species, and *S. mansoni* genes with no similarity in GenBank (NoMatch). **Table S4** contains the list of differentially expressed genes.

*S. mansoni* genes represented in the microarray were automatically annotated with gene ontology (GO) terms [47]. The Ontologizer program [48] was used for highlighting gene ontologies that were significantly over-represented in the set of genes differentially expressed between 1h-treated and control parasites **(** **Table 1** **)**. Categories involved in the translation process such as ribosomal proteins and proteins from the pyruvate dehydrogenase complex were identified as over-represented in genes with higher expression in TNF-α 1h-treated schistosomula. In an opposite group, i.e. genes with higher expression in TNF-α non-treated schistosomula (control parasites), enrichment of GO category involved in the molecular function “nucleotide binding” (GO: GO:0000166) was observed. **Table S5** shows the names of the differentially expressed genes found in each category.

**Table 1:** Significantly enriched (p-value <0.05) GO categories among the genes with higher expression in 3h-old early schistosomula treated for 1 h with TNF-alpha.

Ontology	GO term description	GO term	Counts	p.adjusted
**Genes with higher expression in treated schistosomula**				
cellular component	ribosomal subunit	GO:0033279	9/135	0.045
cellular component	ribosome	GO:0005840	10/164	0.045
biological process	translation	GO:0006412	10/272	0.045
cellular component	pyruvate dehydrogenase complex	GO:0045254	2/2	0.045
**Genes with higher expression in non-treated schistosomula**				
molecular function	nucleotide binding	GO:0000166	20/242	0.021

In order to identify the possible networks of interaction among the significant differentially expressed genes we used the Ingenuity Pathway Analysis software. The most significantly enriched (p = 10^−29^) network comprising differentially expressed genes in 1h-treated schistosomula is shown in **Table 2**. Genes belonging to this enriched network are involved with the following functions: Gene Expression regulation (p-value<2.7×10^−2^), Cellular growth and proliferation (p-value <2.4×10^−2^), Cell cycle (p-value<2.5×10^−2^) and Cellular development (p-value <2.5×10^−2^).

**Table 2:** Genes related to gene expression regulation, cellular growth and proliferation, cell cycle and cellular development that comprise the most significantly enriched (p = 10^−29^) network of differentially expressed genes in 1h-treated schistosomula.

Symbol	Entrez Gene Name	Fold Change	FDR (%)	Sub-cellular Location	Gene family	Probe	Contig
LEO1	Leo1, Paf1/RNA polymerase II complex component, gi:20270337	1.50	0.68	Unknown	other	Q2_P32546	C909180.1
PFDN5	prefoldin subunit 5, gi:22202633	−1.27	1.17	Nucleus	transcription regulator	Q2_P25201	C809910.1
RPS8	ribosomal protein S8, gi:4506743	−1.66	1.17	Cytoplasm	other	Q2_P19300	C801996.1
H2AFJ	H2A histone family, member J, gi:29553970	−1.69	1.60	Unknown	other	Q2_P07060	C812342.1
PPP1R7	protein phosphatase 1, regulatory (inhibitor) subunit 7, gi:4506013	1.29	2.44	Nucleus	phosphatase	Q2_P22537	C806375.1
EIF3M	eukaryotic translation initiation factor 3, subunit M, gi:23397429	−1.65	2.70	Unknown	other	Q2_P19683	C802459.1
THOC1	THO complex 1, gi:154448890	1.16	2.70	Nucleus	other	Q2_P19648	C802409.1
TAZ	Tafazzin, gi:31317259	−1.27	3.02	Nucleus	enzyme	Q2_P34715	C913170.1
ANAPC11	anaphase promoting complex subunit 11, gi:18777675	−1.60	3.29	Cytoplasm	enzyme	Q2_P39504	JAP03774.C
RPL37	ribosomal protein L37, gi:4506641	−1.67	3.29	Cytoplasm	other	Q2_P27341	C813404.1
GOT1	glutamic-oxaloacetic transaminase 1, soluble, gi:4504067	1.37	3.57	Cytoplasm	enzyme	Q2_P26554	C812133.1
CDC23	cell division cycle 23 homolog (S. cerevisiae), gi:118402596	1.78	3.76	Nucleus	enzyme	Q2_P18475	C800690.1
AGPAT9	1-acylglycerol-3-phosphate O-acyltransferase 9, gi:21362092	−1.24	4.49	Cytoplasm	enzyme	Q2_P06232	C811032.1
GGT7	gamma-glutamyltransferase 7, gi:109148539	1.55	4.83	Unknown	enzyme	Q2_P27991	C901554.1
CSNK1D	casein kinase 1, delta, gi:20149530	2.03	4.99	Cytoplasm	kinase	Q2_P26368	C811860.1

### Effects of human TNF-α on gene expression profile in *S. mansoni* adults

In a second set of experiments, paired adult worms freshly recovered from portal perfusion of infected hamsters were incubated with human TNF-α for 1, 6 or 24 h. Two different patterns of expression were detected: a transient change and a sustained change in expression throughout the entire time of exposure to TNF-α.

**Figure 6:**
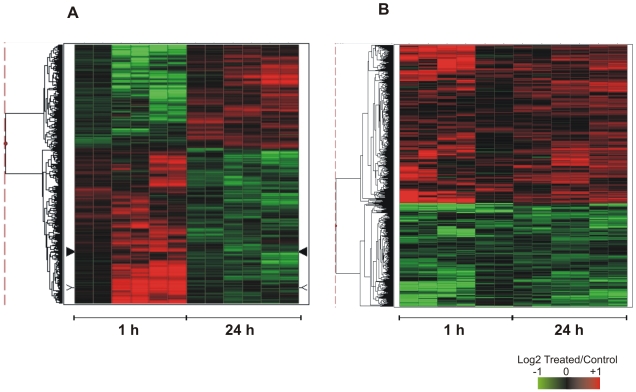
Effect of human TNF-α on gene expression profile in adult worms. Paired adult worms were treated for 1 h and 24 h with 20 ng/ml human TNF-α. Microarrays were used for large-scale gene expression measurements, and the figure shows the genes that exhibited significant (*q-value *≤0.05) changes in their expression levels, in relation to control non-treated paired adults. (**A**) Hierarchical clustering of 1365 genes with transient changes in their expression levels. (**B**) Hierarchical clustering of 492 genes with sustained changes in their expression levels throughout the 24 h period of observation. Each horizontal line represents a gene, each column represents one experimental replica. There are two technical replicates for each of three biological replicates, at each treatment time. Expression levels are indicated as the log2 ratio of intensities (Treated/Control) for each treatment time (1 and 24 h). For each gene, color intensity is proportional to the log ratio as indicated in the scale bar, and shows genes that were induced (red) or repressed (green) by treatment.

A set of 1594 probes that represent 1365 unique genes revealed statistically significant (*q-value *≤0.05) transient changes in expression. Among them, 821 genes were induced by TNF-α in 1 h and repressed after 24 h of treatment, and 544 have the opposite pattern (**Figure 6A**). **Table S6** shows the number of differentially expressed genes according to the three annotation categories: *S. mansoni* genes with orthologs in other species, *S. mansoni* genes with orthologs only in *S. japonicum* and in no other species, and *S. mansoni* genes with no similarity in GenBank (NoMatch). **Table S7** contains the list of differentially expressed genes that showed transient changes.

A search for enriched Gene Ontology (GO) terms associated to the transiently affected genes was performed. Significantly enriched GO terms were found in the sets of genes induced by TNF-α in 1 h (**Table 3**). Genes with induced expression in 1 h of treatment belong to categories related to translation. **Table S8** identifies the genes found in these categories.

**Table 3:** Significantly enriched (p-value <0.05) GO categories among the genes with altered expression in adult worms treated with TNF-alpha.

Ontology	GO term description	GO term	Counts	p.adjusted
**Genes with transient higher expression at 1 h treatment with respect to control**				
cellular component	ribosome	GO:0005840	23/144	6.47E-06
cellular component	ribonucleoprotein complex	GO:0030529	30/281	3.96E-05
cellular component	cytosolic part	GO:0044445	17/92	5.28E-05
cellular component	ribosomal subunit	GO:0033279	19/118	5.28E-05
molecular function	structural constituent of ribosome	GO:0003735	21/120	9.17E-04
cellular component	macromolecular complex	GO:0032991	58/1033	0.004948
molecular function	structural molecule activity	GO:0005198	23/255	0.004948
molecular function	RNA binding	GO:0003723	25/263	0.016548
biological process	translation	GO:0006412	23/257	0.017772
cellular component	cytosol	GO:0005829	20/199	0.025839
**Genes with sustained lower expression (1 h and 24 h) in treated worms with respect to control**				
biological process	nucleotide metabolic process	GO:0009117	4/53	0.016505

Using Ingenuity Pathway Analysis we identified that the most significantly enriched (p = 10^−59^) network with transient changes in expression in adult worms is comprised of genes related to these functions: cellular assembly and organization (p-value<2.2×10^−2^), RNA post-transcription modification (p-value<1.1×10^−2^), Protein synthesis (p-value<1.03×10^−2^), Cell morphology (p-value<4.6×10^−2^), Gene Expression (p-value<3.2×10^−2^), Cell cycle (p-value<4.1×10^−2^). **Table 4** lists the genes involved in these functions and **Figure S1** shows a schematic representation of network interactions.

**Table 4:** Genes that comprise the most significantly enriched (p = 10^−59^) network of differentially expressed genes with transient changes in their expression levels in adult worms treated with human TNF-alpha.

Symbol	Entrez Gene Name	Fold Change	FDR (%)	Sub-cellular location	Family	Probe	Contig
ING4	inhibitor of growth family, member 4, gi:189083828	−1.39	0.00	Nucleus	other	Q2_P25301	C810038.1
NAT13	N-acetyltransferase 13 (GCN5-related), gi:13376735	−1.51	0.00	Cytoplasm	enzyme	Q2_P25057	C809706.1
PSMD8	proteasome (prosome, macropain) 26S subunit, non-ATPase, gi:156631005	−1.34	0.00	Cytoplasm	other	Q2_P22050	C805727.1
EIF1B	eukaryotic translation initiation factor 1B gi:5031711	−1.31	0.67	Cytoplasm	translation regulator	Q2_P18585	C800830.1
TOLLIP	toll interacting protein, gi:21361619	1.37	0.87	Cytoplasm	other	Q2_P06108	C810894.1
PSMB3	proteasome (prosome, macropain) subunit, beta type, 3, gi:22538465	−1.26	0.95	Cytoplasm	peptidase	Q2_P17899	C800020.1
RPL35A	ribosomal protein L35a, gi:16117791	−1.21	1.00	Cytoplasm	other	Q2_P32151	C908319.1
PDLIM1	PDZ and LIM domain 1, gi:13994151	1.23	1.14	Cytoplasm	transcription regulator	Q2_P25128	C809800.1
RPL13	ribosomal protein L13, gi:15431295	−1.44	1.15	Cytoplasm	other	Q2_P25407	C810161.1
RPL11	ribosomal protein L11, gi:15431290	−1.31	1.35	Cytoplasm	other	Q2_P20966	C804195.1
HTATIP2	HIV-1 Tat interactive protein 2, 30kDa, gi:148728164	−1.79	1.78	Nucleus	transcription regulator	Q2_P06098	C810883.1
MBTPS1	membrane-bound transcription factor peptidase, site 1, gi:4506775	1.42	1.78	Cytoplasm	peptidase	Q2_P18217	C800380.1
LIMK1	LIM domain kinase 1, gi:4505001	−1.78	2.20	Cytoplasm	kinase	Q2_P35970	C915732.1
PSMB2	proteasome (prosome, macropain) subunit, beta type, 2, gi: 4506195	−1.33	2.58	Cytoplasm	peptidase	Q2_P19420	C802139.1
DHX9	DEAH (Asp-Glu-Ala-His) box polypeptide 9, gi:100913206	1.47	2.63	Nucleus	enzyme	Q2_P00121	C800369.1
RPL7	ribosomal protein L7, gi:15431301	−1.44	2.85	Cytoplasm	transcription regulator	Q2_P19898	C802735.1
POLR2G	polymerase (RNA) II (DNA directed) polypeptide G, gi:4505947	−1.56	3.12	Nucleus	enzyme	Q2_P33628	C911163.1
FBL	fibrillarin, gi:12056465	−1.59	3.86	Nucleus	other	Q2_P19894	C802728.1
MED10	mediator complex subunit 10, gi:49227854	−1.39	3.86	Nucleus	other	Q2_P26815	C812517.1
NEDD8	neural precursor cell expressed, developmentally down-regulated 8, gi:5453760	−1.38	3.86	Nucleus	enzyme	Q2_P38356	C921416.1
RNMT	RNA (guanine-7-) methyltransferase, gi:4506567	−1.30	3.86	Nucleus	enzyme	Q2_P25200	C809909.1
AIMP1	aminoacyl tRNA synthetase complex-interacting multifunctional protein 1, gi:45006986	−1.42	4.44	Extracellular Space	cytokine	Q2_P02741	C805453.1
ALDH1B1	aldehyde dehydrogenase 1 family, member B1, gi:25777730	1.46	4.81	Cytoplasm	enzyme	Q2_P19570	C802316.1
ATP2A2	ATPase, Ca++ transporting, cardiac muscle, slow twitch 2, gi:4502285	1.42	4.81	Cytoplasm	transporter	Q2_P19618	C802376.1
CHMP1A	chromatin modifying protein 1A, gi:103485496	−1.30	4.81	Extracellular Space	peptidase	Q2_P15994	C916183.1
HIRA	HIR histone cell cycle regulation defective homolog A (S. cerevisiae), gi:21536485	1.38	4.81	Nucleus	transcription regulator	Q2_P18264	C800435.1
NHP2L1	NHP2 non-histone chromosome protein 2-like 1 (S. cerevisiae), gi:4826860	−1.44	4.81	Nucleus	other	Q2_P00464	C801360.1

Fold change of expression at 24 h in relation to 1 h treatment.

A sustained pattern of expression change in adult worms throughout the 24 h of observation was detected for another set of genes, when treated with human TNF-α (**Figure 6B**). A total of 626 probes that represent 492 genes had a sustained change in expression; among them, 336 were induced and 155 repressed by TNF-α. **Table S9** shows the number of differentially expressed genes according to the three different annotation categories: *S. mansoni* genes with orthologs in other species, *S. mansoni* genes with orthologs only in *S. japonicum* and in no other species, and *S. mansoni* genes with no similarity in GenBank (NoMatch). **Table S10** contains the list of genes with sustained changes in their expression levels. Among the set of repressed genes an enriched GO category related to “nucleotide metabolic process” (GO: 0000166) was enriched (**Table 3**). The list of genes found in this category is shown in **Table S8**.

Using Ingenuity Pathway Analysis we identified that the most significantly enriched (p = 10^−29^) network with sustained changes in expression throughout the 24 h of treatment in adult worms is comprised of genes related to these functions: molecular transport (p-value<1.3×10^−2^), Nervous System Development (p-value<1.23×10^−2^), Tissue morphology (p-value<3.8 ×10^−2^), Nucleic Acid metabolism (p-value<1.1×10^−2^), lipid metabolism (p-value<1.36 ×10^−2^), Cellular assembly and organization (p-value<1.0 ×10^−2^), Cell morphology (p-value<1.2 ×10^−2^) and Cell signaling (p-value<1.3 ×10^−2^). **Table 5** lists the genes involved in the above functions.

**Table 5:** Genes that comprise the most significantly enriched (p = 10^−29^) network of differentially expressed genes with sustained changes in their expression levels in adult worms treated with human TNF-alpha for 1 h and 24h.

Symbol	Entrez Gene Name	FDR (%)	Log Ratio	Sub-cellular location	Family	Probe	Contig
PLBD2	phospholipase B domain containing 2, gi:229093316	0.00	0.37	Extracellular Space	other	Q2_P19393	C802110.1
SDHC	succinate dehydrogenase complex, subunit C, integral membrane protein, 15kDa, gi:4506863	0.00	−0.25	Cytoplasm	enzyme	Q2_P06919	C812100.1
NT5C2	5′-nucleotidase, cytosolic II, gi:6912598	0.55	0.28	Cytoplasm	phosphatase	Q2_P18085	C800231.1
VDAC1	voltage-dependent anion channel 1, gi:4507879	0.55	0.20	Cytoplasm	ion channel	Q2_P06247	C811048.1
ARPC1B	actin related protein 2/3 complex, subunit 1B, 41kDa, gi:5031601	0.86	−0.23	Cytoplasm	other	Q2_P07789	C813410.1
PDLIM1	PDZ and LIM domain 1, gi:13994151	0.86	0.25	Cytoplasm	transcription regulator	Q2_P25128	C809800.1
GPSN2	glycoprotein, synaptic 2, gi:24475816	1.16	−0.26	Plasma Membrane	other	Q2_P06002	C810781.1
AP2S1	adaptor-related protein complex 2, sigma 1 subunit, gi:70906430	2.05	−0.27	Cytoplasm	transporter	Q2_P05146	C809441.1
ATP1A3	ATPase, Na+/K+ transporting, alpha 3 polypeptide, gi:22748667	2.17	0.37	Plasma Membrane	transporter	Q2_P19925	C802766.1
SYNGR2	synaptogyrin 2, gi:4759202	2.17	−0.30	Plasma Membrane	other	Q2_P26766	C812444.1
RPS23	ribosomal protein S23, gi:4506701	3.59	−0.39	Cytoplasm	translation regulator	Q2_P02650	C805298.1
TMBIM1	transmembrane BAX inhibitor motif containing 1, gi:50593008	4.03	−0.26	Unknown	other	Q2_P18361	C800545.1
STARD3	StAR-related lipid transfer (START) domain containing 3, gi:31543657	4.62	0.50	Cytoplasm	transporter	Q2_P23697	C807937.1

**Figure 7:**
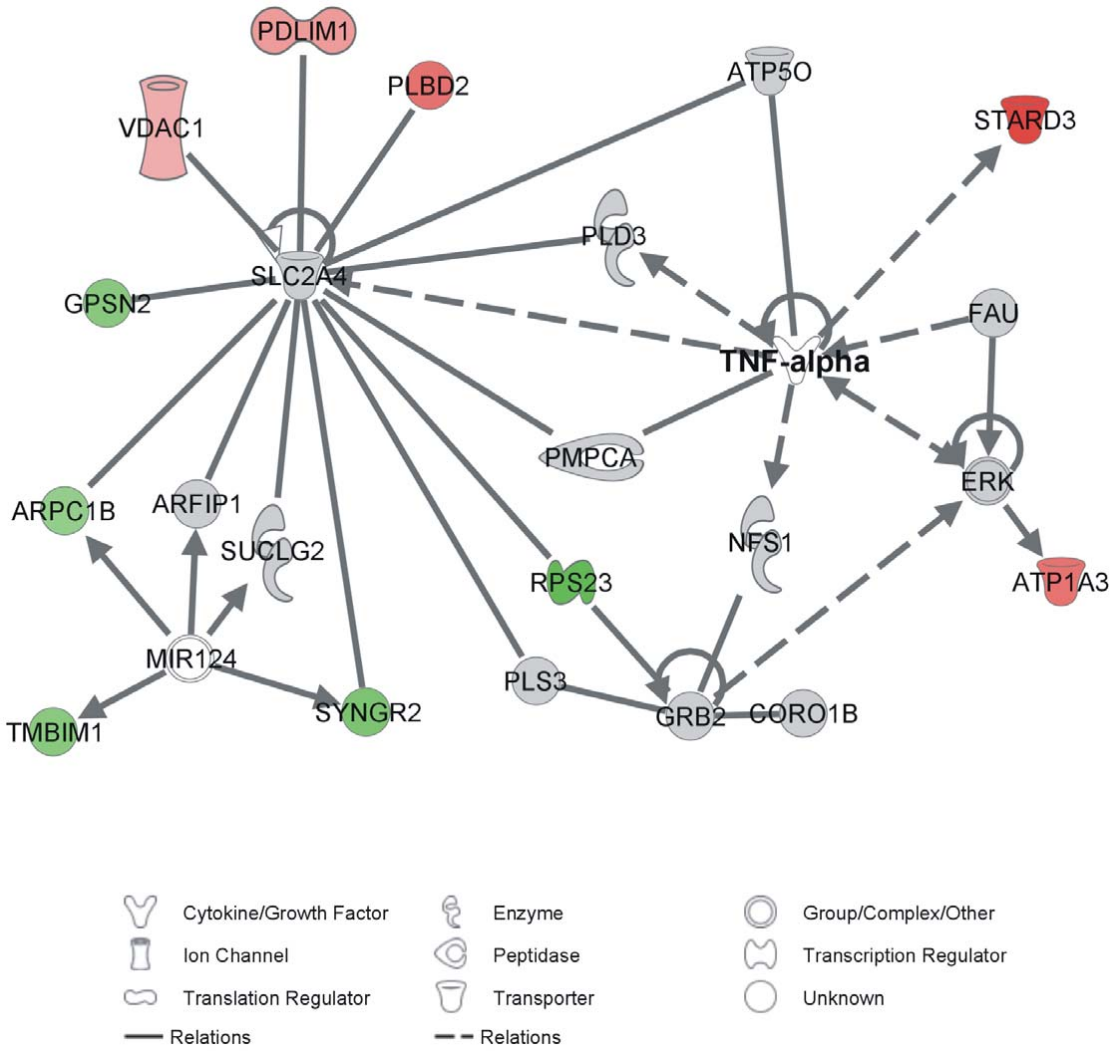
Most significantly enriched (p = 10^−29^) network of *S. mansoni* genes that were sustainably altered at 1h and 24h treatment with human TNF-alpha. In red are the genes that were induced in 1 h and 24 h with respect to their control. In green are genes that were repressed in 1 h and 24 h with respect to their control. Non-significantly altered genes are in grey; in white are the human genes known to belong to the network for which no homolog was found in *S. mansoni*. MIR124 stands for human microRNA 124. Direct relations are marked by continuous lines, while indirect relations have dashed lines.

**Figure 7** shows a schematic representation of network interactions for the genes with a sustained pattern of expression change in adult worms throughout the 24 h of observation that belong to the most significantly enriched (p = 10^−29^) network. It is interesting to note that one of the nodes in the network is centered at TNF-α. No evidence is found for a *S. mansoni* TNF-α gene in the transcriptome or genome. The identified altered network indicates that in adult *S. mansoni* the downstream targets of added human TNF-α are analogous to the known target genes affected by this cytokine in humans.

By comparing the lists of differentially expressed genes in early schistosomula and in paired adult worms, we found only 73 probes, representing 58 genes in common. **Table S11** shows these 58 genes and their expression pattern in each experiment. The very small fraction of differentially expressed genes affected in common in both early schistosomula and adult worms (11% and 3%, respectively) indicates that *S. mansoni* TNF-α response is stage specific; probably some additional elements might have emerged and have been incorporated into the signal transduction pathway during host-parasite co-evolution, causing the activation of different stage-specific target genes.

## Discussion

Our results indicate that the *Sm*TNFR identified in the present work is a possible *S. mansoni* homolog to human TNFR. The possible conserved elements of a complete TNF-α signal transduction pathway were *in silico* identified in *S. mansoni*. In addition, our microarray results show different sets of target genes that are transcriptionally activated by TNF-α signaling in the two different developmental stages studied.

*Sm*TNFR identified in the present work is the only detectable member of the TNF-receptor superfamily in the *S. mansoni* genome. A phylogeny tree analysis suggested that *Sm*TNFR is orthologous to vertebrate TNFRs. Considering the tree topology, *Sm*TNFR represents an ancestral protein that diverged before the formation of TNFR1, TNFR2 and CD40 families in vertebrates, but after the divergence to NGFRs and other TNF-receptors. Interestingly, due to its dissimilar distribution of introns [9] it has been previously hypothesized that the NGFR gene has been formed early in the evolution of TNFR superfamily.

**Figure 8:**
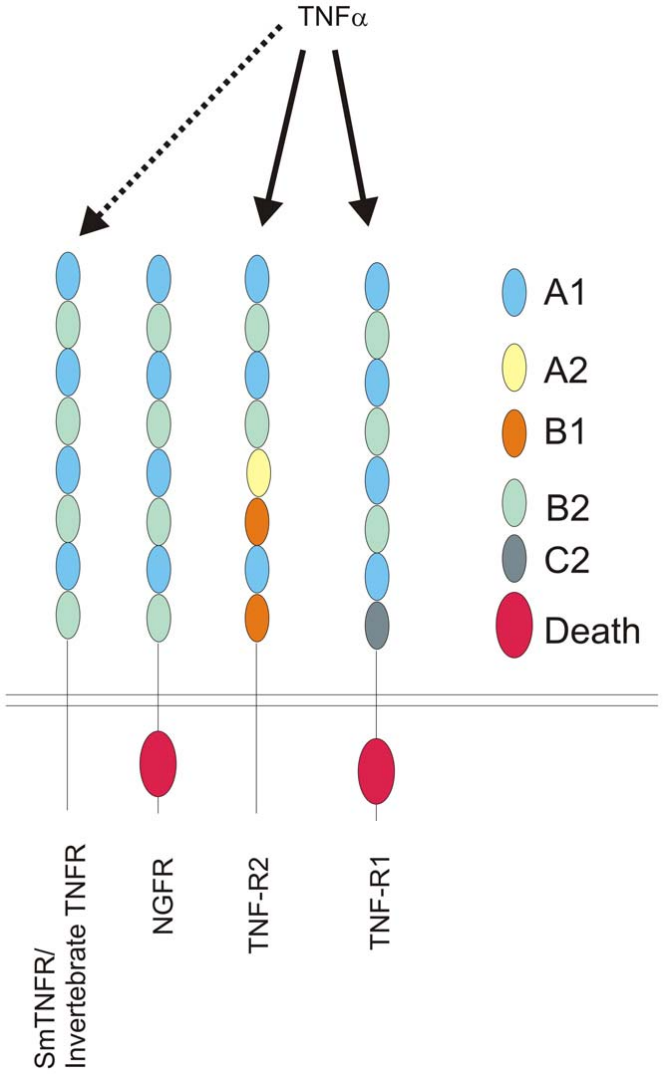
Schematic representation of modules for cysteine-rich domains (CRD) and for death domains of selected TNF receptors. Colored ellipses represent modules or domains detected in the represented TNF receptors. This simplified diagram is intended only to reflect modules organization as proposed in [11] and contains no information relative to size of modules or size of the whole receptor. Solid arrows above the scheme point to the receptors that have TNF-α ligand interaction, and the dotted line arrow indicates a putative interaction of *Sm*TNFR with TNF-α ligand.

Naismith *et al.* [49] developed a classification method for TNFR-family extracellular cysteine-reach domains (CRDs) based on the cysteine repeats: each CRD domain contains modules named according to their type (A, B or C module) and the number of disulphide bridges (1, 2 or 3). The four CRD domains of NGFR contain four A1 modules and four B2 modules that are combined in a manner similar to the first two CRD domains of TNFR2 (**Figure 8**). Regarding the intracellular region, human NGFR and TNFR1have a C-terminal Death-Domain motif, whereas *Sm*TNFR intracellular region lacks the conserved Death-Domain, indicating that *Sm*TNFR is a non-death receptor similar to human TNFR2 (**Figure 8**). The presence of Death-Domain motif in NGFR orthologs is also observed in invertebrate protein sequences such as in *Branchiostoma floridae* (XP_002224391.1). The absence of NGFR homolog with a Death-Domain in *S. mansoni* and in other invertebrates such as *Drosophila melanogaster* (accession numbers: BAC01264.1 and AAM484141) and *Caenorhabditis elegans* (accession numbers: AAP82639, AAB36865, NP_001024870 and NP_001024869), but its presence in invertebrates of the Deuterostomia groups suggests that this family of receptors may have been lost in Protostomes. Interestingly, the cnidarian *Nematostella vectensis* displays a TNFR with a Death-Domain (XP_001629570.1); the available sequence is partial, lacking a portion of the N-terminal extracellular region of the protein containing the TNFR conserved domain (cd00185), which prevented us from adding this sequence to our multiple alignment. However, analysis of the domain fragment suggests that this protein forms a monophyletic group with the other *N. vectensis* TNFR and is not closely related to any NGFR (data not shown). Overall, data suggests that multiple events of acquisition or loss of Death-Domains by TNFR/NGFR receptors must have occurred during their evolution.

Human NGFR does not interact with *bona fide* TNFR ligands and its only known interactions are to dimeric ligands of the neurotrophin family [50]. In contrast, the CD40, TNFR1 and TNFR2 receptors included in our phylogenetic analysis are able to bind *bona fide* trimeric TNF ligands. The protein region chosen for our evolutionary analysis includes the A1-B2-A1 modules previously shown in this family of receptors to be responsible for ligand interaction [49],[51]. Therefore, it is possible to infer that *Sm*TNFR must be capable of binding *bona fide* TNF ligands.

We were unable to find any obvious gene homologs encoding TNF-like ligands in the *S. mansoni* genome. The presence of a single TNF-superfamily receptor in *S. mansoni*, along with the observed effect of human TNF-α on the expression of a very broad range of parasite genes with a stage-specific pattern, is a strong suggestion that host TNF-α as well as other TNF-like ligands may bind to *Sm*TNFR and activate a canonical signal transduction pathway in the parasite. This hypothesis is further supported by the observation that human TNF- α treatment of *S. mansoni* adult worms has induced a sustained change in expression of genes that encode proteins belonging to a significantly enriched network that comprises homologs to known human TNF-α interactors (**Figure 7**). In this respect, we postulate that the minimal downstream elements of the TNF-α signaling pathway are present in the parasite (see below). Further experiments, involving *Sm*TNFR heterologous expression and direct ligand binding assays are warranted to confirm this hypothesis.

The 5′- and 3′-end sequences of *Sm*TNFR differ from the predicted Smp_168070 gene. The 5′-end sequence was obtained by a combination of 5′-RACE experiment and PCRs with cDNA template and primers based on upstream genomic sequence. This approach revealed *Sm*TNFR first exon that contains the start codon and a signal peptide, typical of this type of membrane proteins. The 3′-end sequence was obtained through 3′-RACE experiments; the resulting sequence is different from gene prediction Smp_168070 because in our cDNA the second to last exon is longer than the predicted and we found that this was the last exon (**Figure 1**). Therefore, the last exon of Smp_168070 (that matches the same genomic scaffold but in a downstream region: 64497 to 64519) had been wrongly predicted. Probably *Sm*TNFR cDNA 3′-end is longer than we have obtained here, because the poly-dT-Adaptor primer used in the cDNA synthesis step of the 3′-RACE experiment has annealed to an A-rich region of the message. This can be determined by observing the genomic sequence at the segment to which the cDNA 3′-end is aligned. This genomic sequence contains 12 A's out of 14 bases from 63453 to 63467.

The eleven copies of the simple sequence repeat (SSR) detected in genome scaffold Smp_scaff000357 were not observed in the cDNA sequence; rather, only 7 repeats were present in the cDNA. The seven AAT repeats occur inside the protein-coding region of the message and will encode asparagine residues in the protein. Strand slippage at trinucleotide repeat sequences and expansion in the number of repeats has been proposed to occur when DNA polymerase encounters difficulty while replicating a repeat [52] and may result in diverse human diseases. In bacteria, fungi and in kinetoplastids such as *Trypanosoma brucei* the SSR expansion is described as a mechanism that generates functional diversity [53]. The observed difference between the *S. mansoni* strain sequenced by the genome project and the strain used in this work, with the resulting change in the number of asparagine residues at the intracellular protein domain, could alter the receptor's affinity to downstream signaling elements.

An interesting point of the present work is the *in silico* identification of TNF-α signal transduction pathway elements in *S. mansoni*. We were able to identify nine conserved elements comprising a complete TNFR2-activated pathway (**Figure 4**) [54]. Noteworthy is the fact that none of the downstream elements that interact with the Death-Domain in the canonical vertebrate TNFR1-activated or Neurotrophin/NGF-activated pathways were identified in *S. mansoni* (**Figure 4**). Thus, similarly to the vertebrate TNFR2, it is possible that *Sm*TNFR intracellular region interacts with TRAF (**Figure 4**). Interestingly, the JNK pathway has been shown in *D. melonagaster* to be triggered by Eiger TNF ligand [55]; JNK pathway elements are present in *S. mansoni*, suggesting that Schistosomes may have a response to TNF ligands analogous to that observed in *Drosophila*. The c-JUN transcription factor (also known as AP1) is likely to be the downstream effector of the pathway, acting on promoter regions of target genes to activate their transcription. Other unknown signaling elements may integrate the signaling pathway, such as different protein kinases and transcription factors. Further experiments are necessary to confirm the postulated interaction of each element with upstream and downstream proteins.

An important point to be considered in *S. mansoni* TNF-α signaling pathway is the presence of Caspases 3 and 8 and C-IAP protein. Classically, caspases 3 and 8 are involved in the apoptosis process induced by TNF-α signaling and are inhibited by c-IAP. No direct evidence exists so far in the literature for the apoptosis process in schistosomes. The present findings open a new perspective for *S. mansoni* biology that warrants further investigation.

When cercariae penetrate the human skin, macrophages start secreting TNF-α in that tissue [4]. We propose that the highest expression level of *Sm*TNFR observed in cercariae reflects an evolutionary adaptation; during the penetration process, cercariae will be exposed to host TNF-α in the skin and this could act to promote parasite survival and development, in a similar manner as described in adult worms by Davies *et al* [56].

Finally, this is the first work that evaluated the effect of a human cytokine such as TNF-α on the expression profile of *S. mansoni* using oligonucleotide arrays comprised of 44000 elements [37], probing the entire set of known *S. mansoni* gene fragments.

We reasoned that the exposure of 3-hour old recently transformed schistosomula to human TNF-α would somewhat mimic the conditions at the skin during penetration. In fact, about 3-4 h after exposure to the host skin, schistosomula start invading the epidermis where they will have contact with human TNF-α [4]. In this respect, it is noteworthy that genes involved in mRNA translation and protein synthesis were found among the enriched GO categories in TNF-α treated schistosomula. High metabolic rates are probably present, as the expression of pyruvate dehydrogenase complex and the glucose transporter gene (C800471.1) were increased (**Table S4**). Two proteins related to cell cycle control were induced in early schistosomula treated with human TNF-α: cell division cycle 23 (C800690.1) and cell-cycle and apoptosis regulatory protein 1 (C917134.1) (**Table S4**). In humans, TNF-α signaling resulting from TNFR2 activation is described to induce cell proliferation through anti-apoptotic mechanisms [54].

It is noteworthy that a number of receptors were induced in early schistosomula by human TNF-α, such as Activin receptor (C811502.1), Olfactory receptor (C903953.1), Retinoic acid receptor RXR (Contig C910708.1), a putative seven-transmembrane receptor (C809780.1) and nicotinic acetylcholine receptor (C812523.1) (**Table S4**). These TNF-α induced receptors may increase parasite sensitivity to host stimuli.

A most interesting finding was the up-regulation of TRAF expression (C905513.1) induced in early schistosomula by human TNF-α (**Table S4**). Cercariae have the highest levels of *Sm*TNFR mRNA; cercariae-to-schistosomula early transformed parasites exposed to TNF-α apparently signal a positive feed-back regulation of expression of TRAF, a putative *Sm*TNFR partner.

The target genes activated by human TNF-α in paired adult worms have shown two distinct expression patterns: genes with transient changes (that are induced in 1h and then repressed in 24hs or the opposite pattern) and genes with sustained changes in their expression levels (induced or repressed by TNF-α, independently of parasite's exposure time). Among the genes induced in 1 h by TNF-α, we highlight those that are categorized in the “RNA binding” and “RNA processing” GO terms; they encode proteins present in ribonucleoprotein complexes, such as Ribosomes (**Table S8**). AUT1 gene (C918331.1) had a significantly induced expression in 1 h of treatment. AUT 1 is described in *Drosophila* as essential for development and autophagy process [57] (**Table S7**).

Among TNF-α target genes with transient expression it is worth mentioning a set of genes related to egg laying (**Table S7**). Curiously, Egg shell protein (C910370.1) and P40 egg shell protein (C806383.1) had an induced expression at 1 h treatment and were repressed at 24 h. On the contrary, p48 egg shell protein (contig JAP04349.S) and Egg protein (C804676.1) along with gynecophoral canal protein (C810707.1) had an opposite transient expression change, being repressed at 1 h and induced at 24 h treatment with TNF- α. Amiri *et al* [6] reported an increase in egg laying induced by human TNF-α, whereas Haseeb *et al* [7] described a decrease in egg production upon short time exposure to TNF-α (1, 3 and 6 h). Our results revealed that there is a complex pattern of regulation of the egg laying related genes upon TNF-α treatment that deserves additional characterization.

A particularly interesting finding is the sustained increase of expression in adults (1 h and 24 h treatment) of MAPKKK5 (contig C902728, probe Q2_P09086), a kinase member of the TNF-α signal transduction pathway (**Table S10**). MAPKKK5 kinase protein acts at the initial steps of the signaling process by phosphorylation and activation of other target kinases; the increase of MAPKKK5 expression suggests that as soon as 1 h after TNF-α exposure the signaling pathway is activated not only by phosphorylation but also by an increase in the number of MAPKKK5 molecules present in the cell.

In conclusion, the present work reports an important signaling element in schistosomes, namely *Sm*TNFR, thus giving a molecular perspective to the effects of human TNF-α on *S. mansoni*. The possible receptor is described along with all the elements of a conserved TNF-α signaling pathway together with a set of probable target genes activated by TNF-α in the parasite. The work extends the complexity of signal transduction biology of *S. mansoni*.

## Supporting Information

Figure S1 Most significantly enriched network (p = 10^−59^) of *S. mansoni genes* that were transiently altered at 1h and 24h treatment with TNF-alpha. In red are the genes that were repressed at 1 h with respect to their control and induced at 24 h. In green are genes that were induced at 1 h with respect to their control and repressed at 24 h. Non-significantly altered genes are in grey; in white are the human genes known to belong to the network, for which no homolog was found in *S. mansoni*. Direct relations are marked by continuous lines, while indirect relations have dashed lines.
(0.10 MB PDF)

File S1 Contig sequences in a FASTA format.
(17.60 MB DOC)

Table S1 Part A, primers used in real time RT-PCR, cloning experiments and RACE experiments; Part B, oligoarray probe numbers, the corresponding ESTs contig represented by each probe, the predicted protein Smp_xxxxxx number when available, and the putative human homolog (E-value <1e-20) accession number when available.
(18.58 MB XLS)

Table S2 *S. mansoni* orthologs to human TNF-alpha signaling pathway elements.
(0.04 MB XLS)

Table S3 Expressed genes and differentially expressed genes in schistosomula treated with human TNF-alpha for 1 h.
(0.02 MB XLS)

Table S4 List of 548 unique genes (755 probes) differentially expressed between treated and control schistosomula at 1 h treatment with human TNF-alpha.
(0.41 MB XLS)

Table S5 Significantly enriched (p-value<0.05) GO categories among the genes with changes in expression level in schistosomula treated for 1h with TNF-alpha.
(0.02 MB XLS)

Table S6 Expressed genes and genes with transient differential expression in adult worms treated with human TNF-alpha for 1 h or 24 h.
(0.02 MB XLS)

Table S7 List of 1365 unique genes (1594 probes) differentially expressed between treated and control paired adult worms with transient changes at 1 h or at 24 h of treatment with human TNF-alpha.
(0.73 MB XLS)

Table S8 Significantly enriched GO categories (p-value <0.05) for genes with changes in expression in adult worms treated for 1 h and/or 24 h with TNF-alpha.
(0.02 MB XLS)

Table S9 Expressed genes and genes with sustained differential expression in adult worms treated with human TNF-alpha for 1 h and 24 h.
(0.02 MB XLS)

Table S10 List of 492 genes (626 probes) with sustained differential expression between treated and control paired adult worms at 1 h and 24 h of treatment with human TNF-alpha.
(0.32 MB XLS)

Table S11 List of 58 genes differentially expressed in common between schistosomula and adult paired worms treated with TNF-alpha.
(0.05 MB XLS)
