# Supplementary figures and images for: Identification of the Schistosoma mansoni TNF-Alpha Receptor Gene and the Effect of Human TNF-Alpha on the Parasite Gene Expression Profile
Source: PLoS Negl Trop Dis. 2009 Dec 1;3(12):e556. doi: 10.1371/journal.pntd.0000556 (PMC2779652; doi:10.1371/journal.pntd.0000556)

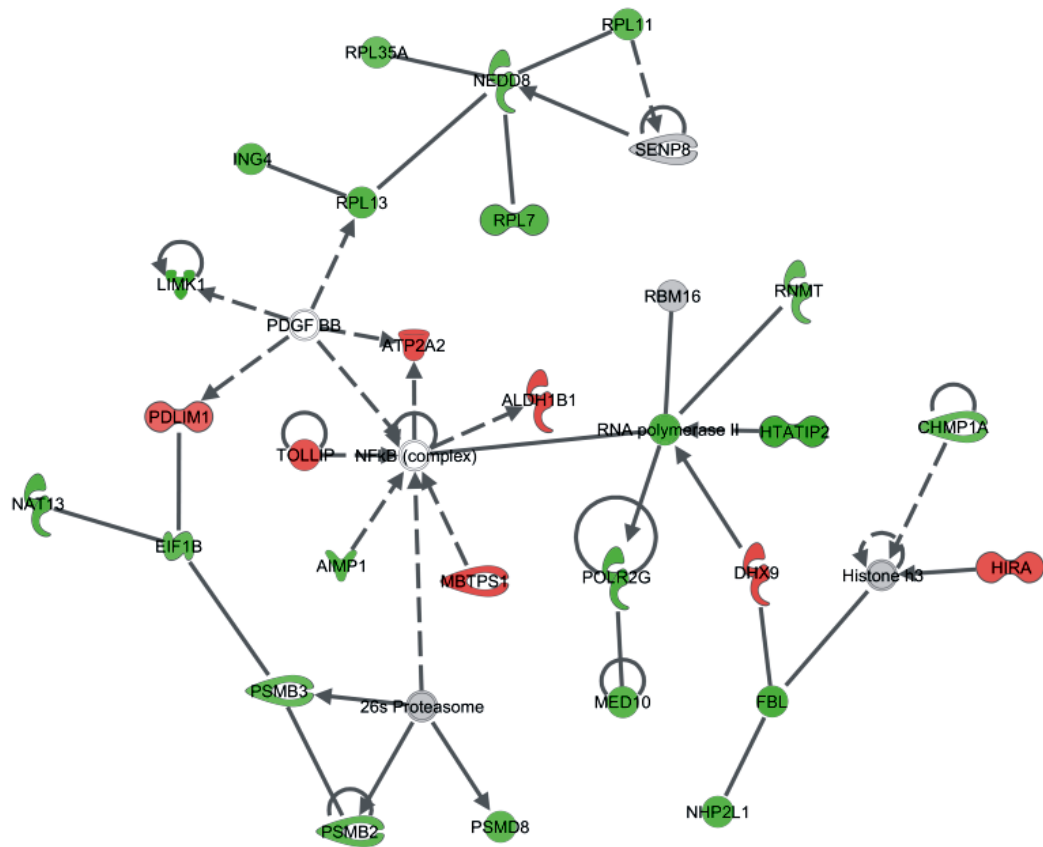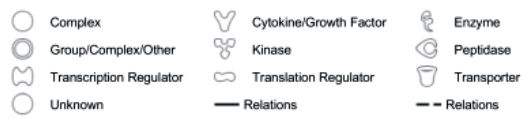

Supplement: Figure S1 — Most significantly enriched network (p = 10−59) of S. mansoni genes that were transiently altered at 1h and 24h treatment with TNF-alpha. In red are the genes that were repressed at 1 h with respect to their control and induced at 24 h. In green are genes that were induced at 1 h with respect to their control and repressed at 24 h. Non-significantly altered genes are in grey; in white are the human genes known to belong to the network, for which no homolog was found in S. mansoni. Direct relations are marked by continuous lines, while indirect relations have dashed lines. (0.10 MB PDF) [file pntd.0000556.s001.pdf]
