# Supplementary material for: Identification of the Schistosoma mansoni TNF-Alpha Receptor Gene and the Effect of Human TNF-Alpha on the Parasite Gene Expression Profile
Source: PLoS Negl Trop Dis. 2009 Dec 1;3(12):e556. doi: 10.1371/journal.pntd.0000556 (PMC2779652; doi:10.1371/journal.pntd.0000556)
Supplement: File S1 — Contig sequences in a FASTA format. (17.60 MB DOC) [file pntd.0000556.s002.doc]

>JAP00084.C|Supercontig_0000033:1046767-1046826

GTAAACTAACCTCTTCTTCAGTACAATCGAATGGCAGATTGCCCACAAAAACACAATTAT

>JAP00095.C|Supercontig_0000063:276764-277023

GTTAACAAAGGCAACGTTTTGTATCAACAACAACAATACGAAAGAGCTCGTGATTGTTACGCGGAAGCATTGCAAGATGATACACGTTGTGTGGAAGCCTTATATAATCTTGGTTTAGTATGCAAACAATTAGAAAGATATGAAGAAGCTCTCGAAGCCTTTTTCAAACTGTATTCTGTTCTGAGGAATAGTGCACCAGTAGTTTATCAGCTAATGGATATCTATGAGAAATTGGGAGATTCTACTCAAGCACAGGAATG

>JAP00118.C|Supercontig_0000054:478214-478428

GTTATGGCCACTAAAAGACCTTCGATTGTTGTAAATCTCCCACCAAGAGTACCACCCATAGACTCGAAATCAAGTTCGGGTATTTTCACAGCTGCAGTTTCGGACACCAAAATATCCCGTGATAAATCGTATATATTTGTAATTCGTAGTTTGTAATGTCTGCCTTTTGGTGAGACTCCACCACCGGACTTAACTTCACAATCCCTAAAACCACA

>JAP00209.C|Supercontig_0000674:409804-409892

CCACATCTAGCCCAAACGCTGTTCTTATTCGAACATCTAGTTCTGGTTGCCTCACAGTTATTAATCCATCGGGTACAACTAGTGAATTA

>JAP00250.C|Supercontig_0000161:991308-991605

AGGTATGGTTAACAGTTTTATCCCAAGCAGAAGCTCAGCGCCGGCTATGTGAGAGAAACAATCTAAGTCCGGAAGCAGCGTTAGAACGTCTTGTACGACAAGCTTCTGCTGTTGCTGAGGTGACTGGTGGGTACACTTGGTTTGAAGCAGGTCAGTATTGTTCTCAGAAAAGTCCTATCGACTATGCTCATGTAATTCTAAGTACAGAATGGGATCCAGAATGTTCGCAGTACCAAGTTGAAAAGTCCTGGAAAGCGCTACAACAAAGGTTAGAGTTAATTACAACAACTCAATGAGT

>JAP00267.C|Supercontig_0000046:143026-143230

GAATTTTTACAACTATCCATTTTCATAAAAAATATTGTTAATCATAGTATCTGTTATTTCATTGCGATGAAATCGGTTTGCTTTAACGACCTTGACTTGAGATCTATCCAAATTCGGCCATAAATCACAATAGGACAAGAAAGCACGTAGTTTTGCTGGAATAGGCAACTTTTTAATAAGGCTAGCATAATTACAATACTTCACT

>JAP00281.C|Supercontig_0000265:355823-356013

GTTTCATTGGACTTGATTGTTTTCTGAGCATCTGCCAAACTTTTCAAGGTTTGATCATGCTCTTCAACAATCTGAGTGTTGCGGTTTTTGACATTCGTGATTTCTGATCTTAATTTATCTAGTTCGGTATGTAGTCTCTGAATTTCAGAGACTTTGGAAACAAGTAAACGCTCCTGTTCCGCTGCTACTTG

>JAP00284.C|Supercontig_0000381:90165-90253

TCACGATATTCTTCCTGTGTTATCACATCAACAATATTAACTCGACCAAGAAGACAACTTGTAGGATATGATGTTGGCATATCTGATTT

>JAP00343.C|Supercontig_0000020:904804-904924

TTTTGCGTACTTAATCGTAGAATAATGATGGTGAGCAAATATGGTTATGTCCTATTTGTTTATTGGAAGATGATGGCAATTTGATGATTGGCTGTGATAATTGTCAAGATTGGTACCATAG

>JAP00507.C|Supercontig_0000306:238724-238878

CAATCATAGATAATTCCATCTACACCCGATTGTGCGAGTAATATACGTTTTTCCGGTATGTTTACAGCTTCACCCCATGCTAATACAACTAATTTGTGTGAATGTGCTAATTCAATAATTCCTGGAACTTTTAATAAACGATCACTTTCTAAACA

>JAP00671.C|Supercontig_0001958:53000-53093

ATTATCACGTAAATCCCAGTAGTCTTCATCTGCTGTAAATATTTCATGATTAGAAGAGTTTCGGATACTTGTAACACAAGTTGTAGTAGTAGTA

>JAP00716.C|Supercontig_0000149:25835-26258

AAGATTATATGGAAGTTGTTGCAGGTGAATCTGAAGAATTAGCTACAAGTATTGACTCTGGTACAGCTTTATCCATTGAATTGGCTAGTACAGCGACCTCCGCTGCCCCACTTTTGTTGGCTGCTCGTAAACGTCGACAGCAATTAGCTTTAGATAGTTTAGCTGGACATGGTAACCGTGCTTCCGTGGCTAATCAATCTAAATTACGTAAAAGGACAAATCTAAATGCTTTTGGAGACTCTGAAGATGAAGAAGAAGAGGATGATGACGATGATGATGATGAAGATGAACGTGCAGCTGCCTTATTGGGAGGTCAGATTCTTGGCTCTGGATCAGAGTTAAGAAATCCTCTGTCTGCTGCTACTCTCGGCTGTAATATGCCTATGGATTTATATAGGTTGCGGCAAATGCAAAGAGCTAAGTA

>JAP00838.C|Supercontig_0000489:436444-436554

ATTAATTCCGTAAAATAAGATACTAAACTTTTCACATTTGGTATAGGTGTATCCCTAGTTTTCACTAAATTAGGAAATAGATCCGTTGTTGATTCTGTTGAATGATTCAAT

>JAP00943.C|Supercontig_0000121:1203154-1203293

TTGCAGGTGCTCATTCAATTGGTGTGAGTTGTTGGCGCCCGGTAGGAGATTCTGTTGTAGAAGAACTAAGAAGATTTTTTATAGGTGGAACATGCCAACTAGAGGACCCCACATTTGCCAAAATACCTGGATCATTTGAA

>JAP01100.C|Supercontig_0000000:1054037-1054201

TTATTATTTCAATTTTGAATTGTTTAAATTAACTGTAAAATGTCCAGAACCGACTCGAAAATTCTCTTCAAATTTATAACCATTTCTTAAAAGATACTGTATAACTGCTGGTCTTAACACTGGAGCTATTGACACTAAGTCACTTTCATTACCAGCTGCTCGACC

>JAP01178.C|Supercontig_0000195:1286591-1287039

AAAATTTCTGAATCTTCTGTATCAGATATATAAAGTTTTTCACCTGTATACCTTTGTGGTTCAACTAATAATGGTTCAGCGGATAAAGCAATTAAATCACGTGGTTCAAATTGAACTAACCAATCTTTTCTAGGATGTTTATTAAACATAACTGGAAGATATTCATCAATAGCGATTAATTTTTTTAAAGGTTTTTGTTTTAATAATTTCTCGGCACCATTTCTATTTAAAATATAACCAAGTGTCCAATAAGTATAATCAGGATAAGTTAAACTAGTTGTATTTGGTACACGTTTCTCAGATTTTGACATTCTTTTACGACCAATATAAAGTAGATCCCAATTAGCAACATTATCATCTGCTTCGTTAATAACTTTATTTAAATTACGAACAAAAGCAGGAGCAAAACGTAGATCATCTTCAAGAATTAATATACGATTATAACCATT

>JAP01181.C|Supercontig_0000243:323219-323364

TACCTGATAATCTAGCTCCGCCAAATGGTTGTTGACCAACTACAGATCCAGTGGATTGAACATTTATGTAAAAATTACCAGTTGTATCGATAAGGCGATCAGTGGCATACTTAATAAATTCTTCATCCTGAGCAAAGATGGAACCT

>JAP01196.C|Supercontig_0000149:290097-290233

TTCCAGATGTTAGCCTTACAAGTGATTTTATCGCAGGATTTTGTGGTGAAACTGAAGAAGATCACTCTCAATCTCTTGAGCTTATTGAACGTGTTGGTTATTCATTTTGTTTCTGTTTCCCATACAGTATGCGTGAG

>JAP01286.C|Supercontig_0000149:628646-628861

TCAATACAAATGTTATACGAAATTGAAGTGAAATTGACTGATTTACTTGAAATATTACAAACCTTACCTGAAGATGAAGTTAACGAAGTCAAACAAGCCAAAGAAATTGAACATAGGCAACAAATTAAAGAGGACAAAAAGTATCAACAGCGTTTATATCAAGAGGAACGTATACAAAAAGCATTAGAACGTGCTAAAGCTGCACCAAAGAAACAA

>JAP01389.C|Supercontig_0000144:979194-979611

ATGGTATCAAGAAACAACTTCCTCCTTCAATAGCTAAACCAACTGCAGCCATTAACCATTGTTTACTTGGATATTTATCAGCTAACTTAATCGTTGTTATAACTCCAGCTACATGTGGTAAAAATGCTGGTAACCATACTAAACCTTCTTGTGCATTTGTTGCTTTCATACTTTTTGACATCCAATTTGATATTGTCGGTTCTAAAAATGCAAGTGATACATTAGCTACAGTAAGACATCCAGCACATATAGCAATATAAGGATCCATTAATAAACGATGTATTGGAGTTCCTTTTGGTAGATTACCTTGAGTTTTTAAAACTGTTCGTTCAATTCTAACAGGTTGCATAATAATCAATAATAAACAACCATCAAATAGTGCTATAAATGCAAGTGTAATAAATGGTAATTCTTTGCC

>JAP01393.C|Supercontig_0000673:232936-233218

AGGATAGCTTAAGTGACATAAATGCCAAGTATCGAGAAGCAAGTGGCGGAATCACTACTCGAAGTCGTACTTTAGCTGAAATCAGTGAAGAACTGGAACGTGTCAAAACAGAGATGGACGAGAAAGGTTTGAGTATGACAGACACTTCTTCTGTAGTGCGTATTAGACAAGCTATCCAGCGTCTTAAGTCAGAGATTACTACGATGGACATCAGAACTGGTGTTTTGGAGCACATCCTACTTAGAACACATTTACGAGTTCGGGAAGATAGTCAGAAACCATT

>JAP01474.C|Supercontig_0000283:232625-232914

AAAAGTCGTGGTTCCTGGAGACACTGGAATGAAAAGAATAAAACTGATGACCAAGTTTCTGATAGAAAAATACGTGAAATTATTGTACCTACAATGGATACAGCACGTTATAAATTTATAGTCGATTTATGCATGAAGAAACATCGTCCACTTTTGTATGTAGGTCCAACTGGGACCGGGAAATCAGTATATGTTCAAGAAAAGTTAATGAGAGAAATTGACAAAGATAAGTATGTAGCCTATTTTGTCAACTTTTCTGCTCAAACAAGTGCAAATCAAACTCAGGTAAT

>JAP01482.C|Supercontig_0000448:224474-224644

CCTGAATACTTTCGAATGAATGACTCATCATAGCAATTAACATGTTGACCAATACTATGATTATAATACCATGATACACAGCAAATAGTACCATACCGACACTGTCAACAATTGTTGTCGGTGAATCAGTGTCAATCATACCATCCGTGTATTTATGTTCAGCTTGATCTG

>JAP01565.C|Supercontig_0000020:927358-927459

AGAATATCTTACTGGATTTCGAAAAAGAAAGCTTGAACGTAAAGAAAGGGGACGCAAAGCAGCAGAAAAACAATTAAAAGATGAGATTAAGGCTGTTAAGGA

>JAP01677.C|Supercontig_0001517:1662-1787

CGTAAAGCAGTATATGTACCAAGATGAAGAAATGAATAGTTTGGATCATAGAAAACATAAACTGATGAAAGACATCGTGGAAGTAGATCAATAACACCGACAGCAATTAATTTTTCACCATCAAGC

>JAP01756.C|Supercontig_0000291:109565-109736

GTAATTTGATAATATCCAACACCATATGGTTTTCCAGGATGTTTAAGCAGTCGTACTTGACGAATACCTGTTGTTAAACGTGCAAGGAAAACTCGGAGAGAACAAATATGCCCCCAACTATCTCCAAGCGCAGGTACTAGACATGAATTTTGATCATTTCTGCATTGTTCTT

>JAP01761.C|Supercontig_0000131:1119503-1119716

GGATTAAGTTGATATTTAACTTCGTTACTCTCAGAATCCTGTTCTGCCAACCAATTCAAACCAAGATTTAACATTAAATTGACTAAATTGTCAAGCGCTAACTTCTCCTGTTGACTATACAGCTGTGCACTCAACGGCCGAAGAGTGGAAGCGATCAGACTCAAAAGAGTAATTAAAGTAGGTGCCATATCTAATAAAAAATGACGACGTGGTA

>JAP01868.C|Supercontig_0000167:261155-261300

ACAACTTCTAATCCTCAAACACCTGTAATGGCACCATTAAATCTTCCACCTGAAACATTACAAATTGATTTAGCTAGAATAATAGCGACGGCTTCATCAAATAATCCACAGCCTGAGAATATTATCGAAATATGGTGTGGTGATCA

>JAP01960.C|Supercontig_0000188:305230-305603

TGCATGTGATCTAAATGACCACCATGACTACAACCTCTACAACAAACAAATAAACCACGAACAACTAGATGACATAATGCACAAGTTATAGTAGCTGGATCAAACGATGTTGCATGACGTGAACAAGCCCAACCAGTATGTCCAGTTAATTGTTGAGTTGTTTCACTAGCACATAATGGTTTTGTGCATAAACCACATCGTATTGATAATGTTGTTGAAGCTTGATTTAAGGCTGCTACACCCGGTGCTAATGCTAAAGACATGTTACTTAAACGTGAGATAAACGGATTGTCAATACTTGTTAAACCTCCACTTTTCCAGCGAGTTCGTGTATTTGCATCTGACCGAGTATCTCCAGTATTTCCGGGAGTTTT

>JAP01964.C|Supercontig_0000187:907986-908207

ACCAGATTGAACAGGAATATGAAGAAATGAATACACTCTAGGATGTGATAATACTTCCGCGATTTCAACTAACTGATCTAATATATATGGCGGATTTGTCATACCCAAACGTAACATACATCCAGCAGGAATAATTGGGACAAGACCAGCCAACAGATCAGCAAGTGTAATGTGATGTGACCATTTTTCAGATAATCCAGGACAAATAAGAGATGATGTAGT

>JAP02033.C|Supercontig_0000121:1097964-1098213

TCATCAGATAATCTTATTGGCTATGAATTAGTTCATCGTCTTATCAATTTCGCTCTCAAACAATTTAAACATGCTTCCAATCGTGCAAGACAATCATGTTATGCATTGGAATCGAATAATGCTGAGCTGAAAAAAGCTTTAGATGAATCAAAGCAGCAAGTTAATCAATTAGTGGAAACTATTGATGGATTAATTGCAGATCAACGTCGTTATCGTGAAACCGAGAACAATCTTAACTGGGAAATTAAAG

>JAP02060.C|Supercontig_0000119:906147-906289

ATAGATTGTGTTAAACCAAGCATAGACCAACTTAAATGATAAGTAGATATAATACGAAAAAATAGTTGACGTAATTCCTTCGGTATATGCACTAATGGTTTAAATGGTGATATACTAATTTGTGCTATAGGCAAGCGATTACA

>JAP02118.C|Supercontig_0000684:356243-356502

ATATACAAATTTGCCAACATGTTCTAATGAAATGTTAGAACAAAATACAACGACAACTTTTGTACCAGTTTCTATAAAACTAGTAAATTTACAGCCTTTATGTTGTCAACCATGTGATATATTTACAGGTAGTACAAAAATCTTATCTAAACCAGATGACACTGTGATAGAATGTGAATATTTAAATAATAACTGCATTTATCCTATTGAAACTACAGGGTCAGTTGTAAAATTACATGTTGTATCAGATTCCAGTGGAT

>JAP02196.C|Supercontig_0000270:229394-229491

GGAATACCTCGTTGGCCAATTCCACTACCAGATTTACATGGTCGGAATGGATTGTTAACTCGTCGTCGTCTAGCAGATCAAGCTTGGTCAAGAGCTTC

>JAP02228.C|Supercontig_0000239:869109-869317

CAACCATATATTGAAGCAAGTGAATTAAATGCCAATGACGTACAAGTTACATTGATTATGCAAGGTTTACAAGCGCCTAGTCGAGGATTTTGTGGACTTATTAAACCCGGTTGTTCAGGGAATTTTCACAGTGATTCTTTCAGTACAACATCTGCAAGTATTCGTCAACAATTAGGCAATGGATTACTAAAAATGGAGCTTGGTGAATA

>JAP02258.C|Supercontig_0000295:660207-660408

AACAATAATCAAGATCCATCGAAATTAAATCAATACAATCATTCTAACATACGACCTCCAGGTTTAATATTAGATTGTGGGCTAGAAGAATATTCCTCCAACTATTACGCAGATCCACAACTACGTATTTTAATCCAATGGATATCAGCTACACTAGTTTATAATTCTCCTTGTTCTAAATTGAGTTATTTAGGTAGTAAAA

>JAP02266.C|Supercontig_0000262:563522-563654

GATTTTGAAGTTCTAGATTCCAGGGAGCGATTATGCTTAGAAATTATGGTTGCATCGGACAATTTGCAACCTACTCTCAGTATCTCGAATCGATTTAATTTGTTTTATAAAAAAATATTTTGTTTATGAACAT

>JAP02332.C|Supercontig_0000044:598016-598216

AATAATTTATGGACACCTTGTCAACAATTTCGTCAATCTCAAGCTCAACAAAATCAAACAATTTATTATTCACCATTATTACAAACACAATCAACACTTACTAATTATGAATATGCAAATTGTTGGGATCCATCAACACCACCAACAACTGGTATAATTACAGCTAATAATGAAAATGATTGGCCACCTTTATAAATTATA

>JAP02371.C|Supercontig_0001914:53852-53990

CATGAAACACCTGAAACAATGATTGATGAAAATAGCAATAATGGTAGAATATCCGAAACAAATTATCATCATCCTATAATTCTATTGTCATCGCCACCAATTCATGAAATTGCATTTACCCCAATTCATCCATCAACAA

>JAP02418.C|Supercontig_0000159:1311277-1311472

GTTGAACAAGTTATTCTTCATATAAAATGGAAACATTCATGTCGTGGAAGTTTAGAAATGCATTTAATTAGTCCATCCAATACTGATGCATTAATATTAGGTCAACGAAAATTAGATACGTATACAGGTGAAGGATCAATGATATTTACATCAGTGGTACATTGGGGTGAAATAGCCGCTGGTTTATGGAAGTTAA

>JAP02515.C|Supercontig_0000458:149837-150060

CGATTCTTTGAAATAGATGAAATCGGTAAATGATTATTTTGAATTCTATCCGTTGATAATAAACTTTTATGTATTGGGCTTAAATGAATGACATGTGGAAAATCATGTTCAGTATTATTTTTAGTAGGTTTATATCGTTCTAAGCATATCCATTGTCCAGTATCTGATTCACTTGCCCATTTTATAAATAAACGACTTTCAAGTGTTTTTGGATCTAACCATTT

>JAP02601.C|Supercontig_0000089:423366-423431

AGTTTACGATTTTCCTCCAGTAGATCAGCACACATATTTGCCAAATGATTTCGACGGGATTGTTCC

>JAP02615.C|Supercontig_0000241:229877-230073

GGTAAGCATAATTGTAACTGTTTATACTCATTGGCCGATCGATTCTGTAATCGAGAGTTTGAAAAAACTAGTTTTGAATTCTCTGGATTTATTTCCTGTTTATTTGATGATTTCATAAGTAATGAAGGTCGAATAAATACACATCCTTCGAAATCGTCCGACATTATCAAATCATGATCCATGACAATAAAAGCTAA

>JAP02646.C|Supercontig_0019079:92087-92217

CAAAAAATGGTTATTTCTCTGGAGGTGCAGGTTATGTCCTCTCACAAGAAGCATTAAAGCGTATTGTTGAACAAGCTATTGACAAGCATCCAAGTTGTCCGACGTATGATGAAGACAAAGAAGATGTGAAA

>JAP02648.C|Supercontig_0000453:386068-386267

TGGAAATTGGAGGCTCAGTATTTGCAAATCTTTATGGTAAACAAGCAAAACGTCTTTTAAATACCATCAGTATGATTGTCAATAAATCACCTGTACTACGCCAATCACTACCAGAAATGCAATTAATATCGTTGCTTGAAAAGTATGGAAGTATCGATACAATTCCAGATCGAATTGGTTTTATTGATAAGAGTTTTTGG

>JAP02653.C|Supercontig_0000122:553518-553731

ATCACAAGTATCTTCACAACAAGTTGAAATAGTTACAACCTTAGAAGAATTATTGGTATTTCGTTCCGAGTCAGAGAAATGTCGTTTCCTTGTTGGTTGTAATGACAACTGATTAGAATTGTTTAATTTGCTTTCTGTCACTGCCACTTCCTGTGGTCCACCGAATAAGCGGCGTTTAACCAATGGACGACTCGGAAGTGGGCCAGGAGATGTT

>JAP02711.C|Supercontig_0000064:628219-628368

TTAAATATTCACTTAAATTAGCAATCAGTCTTAAGAGACTAATAACTGCATCTGGATGTACAATGAGAATGTCTGCTACATTCGATGTTGTGTTGTTATTATTATTATTAATAAGTAATTCAGTATTCCCGACAGTATTATCATTATTCG

>JAP02758.C|Supercontig_0000453:142651-142891

GTTAGATGTAAAAACAAAAGAGAAGGTCGTCATTTGTCTGCTCAACATCTTTTAGCCCGTCTACATCCTGAAGTTAATACCTGGAGTGGTCTTTTGCGTATGTATGGTCCGGGGAGTAAACCAGACAAGCGAAATGAACTGGAAACAATACAAGATGCACAGACTCAACAGAAGTCTGCTGTAAAAGCCAGTTTAATTCGTTTATTGAAAGCGAAAATGAGTGAATTAGCTGACCAATGGG

>JAP02785.C|Supercontig_0000035:231228-231383

ACAATTGAAACACTAGCAGCTCTTAAACTTGTCAACATCGATTGTTGATGATGTTTATCTTCAAATAAATTCATTAAACGACGTTGAATGAATGCACGTATAACGTTAGCACTACAATCTTGAAAATCACAATCTTCTCGATGATCCGTTAAATAC

>JAP02805.C|Supercontig_0000030:1167304-1167430

TGCTGTAATCCAACATGTAAAAATCCAGTTTCTTATGATCCTCGTTGGAATGGTCAGTATTGTTCAACTAAATGTGTTGGAGAACATTGTCAATTAACCTTTATTGATTGGTGTAATAATAAACGAT

>JAP02806.C|Supercontig_0000160:1076391-1076572

AAATCGAATATAAGACTACATCTTTTGGCCCGTGTAGCTGAGTATTGCTTACAAGAACACACTCCCAACAATTCAATACACAAAATGAACGAGTATCATACGAGTCAAGTTACCAGTTTGACTGATCAACTGGTGTTTCTGTGTAGGCGTCTGTTAGTTCCCTGTCATTTTATTGAATATCC

>JAP02823.C|Supercontig_0000186:583121-583316

TTAATTTATACATGTCACCATCATAACTCAGTTGGCCTTCTCTAATTTTCTTATCTAGAAATCGGCGTAGTTCATCTCTGTTGAATCCTAAACTGGATGTAGAACCGAGTGCGAACATTCTAAGCATTGAATGGATTCGATCTAATGACGAACTTCCAAGGTTGGTTAACATAGCAGTGATATAAGACCAAAATAC

>JAP02845.C|Supercontig_0000159:903975-904183

GAACAATTTATACATAACCATTCTTGTTTGGGTGTTGACATTAATATCAATGGTTGATGACATAATTGATGATATAATGAACCACATTTGATGCACTCCACTAGTACATTTGCATTTAATGTTTTATTGTCCGGTTCTAATTGTGGTTGTTGTGACAGACGACCACATACACGACACACAAGATCTGCGACTAGACTTGCAGCCAATAA

>JAP02857.C|Supercontig_0000012:949370-949619

TTAAAGGAACAAGAAGAATTACATAAGCGAATAGATTCATTAAAATATGATTTCTCACTTGCGTACTCTAAATTCCTACTACTTGAACGAAGTCTAACACATAAAACATGCCCTAGACCAATTGATCCAGTTGCAACTGCAAAAACAGACCAAAATTTGTATGAAACCTTCTTTTGGTTTTTTGTTTTTTGTATGTGGATAACATTTCTACGAACAAGATTTAAAGAAATCTGTGCTCAAATTGGATGGT

>JAP02906.C|Supercontig_0000312:699371-699469

TGGTTTTGGCAGTCCATTGGGTTCTTCACGACCTAAACCAGCAGGATTACTTTCAGCTTCAGCACTATTCAACATTTCCAACTGACATTCAACTTTACC

>JAP02938.C|Supercontig_0002060:258616-258812

ATATAATTTATAAGTATTTCATGTATTTTTTTATAGCCTTTCTCTGTACATAATGATAAACAATCATTTTCCATACAGTCAATGATTTCAACTTTAGCTTGTTTGTCTAGTAAGAACTCAACAGCTTCCTGACAACCTTCACGTATAGCTAACATAATAGGTGTTCTATCCCAGTCGTCTTTCATATTCATTAATCT

>JAP02991.C|Supercontig_0000038:814514-814951

TCTTCATCATCATCCTCATCATTACCTAATTCACCGGAATCTTCACCAATTGATATATCAGAGACTGGACCTATTACATCTTCCGCATCTTCATCATCTGCACTAGATTGATTTATATCTTCTGGATTATCATCGGCACCGAATAGACATCGGGTTAAATTACCATGACCTCTTTGTTGATAAGCTATATTATTTTCATATTTACATGATTCATATCTATCACGTCGAGAATCATACATTATTTCTCGAGTACGTTCAGCATCAGCATAATCATAACCTTCTGGGCAAGTGTTCCATTCTTTATTTTCTACATCTTCTGTATCTTGTATCCACCAATTTACATTTGGTTCAGGATCAGAATGTATTGCTGGAGAGACAGATAAACGACTGAATTTATGATGATGTGAGCCAATAAGCCAACGTTTCTGACTTGAAAAG

>JAP03060.C|Supercontig_0000131:1485839-1486010

TAATTCACTTTATCCCATCCCAATTGAAACCACATATTTTCAAGCCATAATTGAACATCTTGATCATGTAATAATATAACAGAAAATAAATTAGAACGTAATTCAATTGGTAAACTATTACGTAATCGATTGCGTAATCTTAAATCAATAATTAATTGATCAAAACATTCCA

>JAP03097.C|Supercontig_0000091:773613-773799

GTTGATCGATCTATCCTAGTTGATTTGTTATGTTGTACATGTTGTACAAATTCAGATGATGAAGATGAAGCTTATTCAAATCTTATTGAAAATCATCGTTTAGCTCGTATAGTTACAATGGGAGCTATATTGACTATATTGGGTTTGATATTTGCTTATATAATACGTTCTGCAACTGAAACTCAAA

>JAP03192.C|Supercontig_0000019:1680229-1680368

TCTTGCCAATGGCGCTGAGCATCTTCCATAAATGATTTAGCTGGTATGGTTGCCAGAAGTGCAGGCCACAATAGAAGAGCTAATGATCTGTTAATAAAGTCTGTATATTTTGAGGACAAAAGTGTACGTGCCACATTGAA

>JAP03201.C|Supercontig_0000103:241072-241200

GGCAGCAGCATTGAACTCAGTCAATGAATATGTGAAACGAAATGAAGTGTCCAAATCATTTCATATATTACGTGAACATTTTGGTTTTAAAAGTTTACCTTCAAACCGTTATGTTGTTGATATTTTATA

>JAP03245.C|Supercontig_0000048:1302419-1302567

TCGATTACTAGGTGGGAATGCATTTGGATTATTATTTAAACCATGTAATACATTTAATGCATTTTCATGATTATTAAATGCAACAAATCCATAACCAAGTGAACGATATTGTTGCCTGCCCGGTTGAAGATTACGCATAATACGACACT

>JAP03264.C|Supercontig_0000119:34316-34693

AGCACTCGACTCTATCAGGGCAACCGTTGGGAAATGTCAATTATTGTTACAGAAAAAGTTGCCATTTTTCAAAACCCTAATTGAAATGGCAGAAACAAGCCTTGGGAAATCTGACACACTGTCGAATAGTGCAACAGCACCAGAAGCCACTATAAATGATCTCTTAGGTTATTGGACCTTAGTAGCTGATGAAATCAGTTTATCCGATAGTGCTTTCGAACGTCTACGTGTTTGGCGTGATGAATTACATTGGTCAATTGATCAGTGTCCTGTTACCCCAGCCAGATCACAAATTGTTAAACGACGCGGCCGTCCATCTAAACAAATAAAACGTTTACCAACTCCAAAGAATAGTAAATCCAGAAAGTCTCAGTCTGT

>JAP03356.C|Supercontig_0000012:848750-848993

TGAAGCAAAATTATTACTTGAAAAGTGGCTTGATGAAAGAAATGCTTTAAGACGTGGTAGTAAATGTGTATTTAATCCACAATTTGGAAGTATTTTTCGTTCTTTTCATAATCCATCTTATTTTTCTCAACGTCTTGGACAATATGCAACATTGTATACGTCAAGGGTCACAAATCTGTTACATTTTCCACTGGATCATACATTTTACCCTAAAAGAACTGCTTTACCACATGAATCATTTTAA

>JAP03363.C|Supercontig_0000045:55292-55474

AGAAGAATTCCATGTAGTTTGCTTACAAGAGGTTATACTTGTCTGTTTGGAAATTCTCCGTGAGAAGCTAGAGTCGACGTATCATATTTTTTCTGCATCTGATCATAACTCTCTATGGGATTATTTTGTTGTCATTTTAGTTAAAAAGCATCCAGACATAAAGGTTGACACGGATTCTGTTAG

>JAP03370.C|Supercontig_0000228:598383-598598

TTTTAGTTCTTGAAATCCAATATTGAACCTACTGATCCAAATAATCCTAAAGAATACCCCCGAGTTTTGACTCATCGTTTTCCTCCTATGAATAAAAGGAAACGTCGATTCATTCATGATATTGTAGAATACTATGGTATGGAAGCTTGTACATGTGATCCGGGACCTAACTGTCATGTAATGATTATTGCGAGACGTGGTTATTCCAAACTACCT

>JAP03449.C|Supercontig_0000048:772889-773057

TTAATTCCCATTTCCCTAAGTCGACAACCAGCAGCATAAGCTTGTCGCCAACCAATATCGGTGAGAACCTTTTCTGCCGATTTCTTTCTATTCAAATGATACTGTCCATGACGTATAAAAATAATTAATTTATGATAAGGTTGCTGATGTTTTACTACGTTAAGTGTAT

>JAP03478.C|Supercontig_0000176:203028-203348

GAAGTGATTTGGCTTTAACTACACAAGTTAAACTTTCACAATTTCGATATGTGAATGTACATCATGGTATATTTCGTTTACGATTATTACATTTCTTCATTGTATTTTGTGTAGTAATTACATGCTCAATACTTATACCTGGGATTATATTTACCTATTTAGAACAGAATTGGACATATTTTGATGCAATTTATTTTTGTATCATATCACTTAGTGCAGTTGGTTTTGGTGATATGGTGGCAAGTGAAAGTAAAGACAGCAGTGTATCATCAATAGTAACAATATTATATGTGAAAAATATTTACAGAGTAATGACTGCAA

>JAP03479.C|Supercontig_0000156:1423985-1424183

TGATTGAGCCGCACACCAATTCGAACATCTTCAGTGCCCATCATCTTTTTAGCAAACATTTTTATTTCTTTAATAGCTCGAGGAGCTCGCCTTTTGAAACCAACACCATGAATTCGTTTGTGCAAATGAATTGTATATTCGCGAGTAGCAACACCTTTTCTGGCAGTTCTTCTAGCTTTCGTTCCAGATTGCACCATGG

>JAP03616.C|Supercontig_0000521:6309-6385

TAATGACAATGTCAACTAATATACGTACAATACGTGGTAATCGAGATTTACATGCTTCAGGATTTTCATTAAGTGGT

>JAP03690.C|Supercontig_0000048:1204278-1204481

AGACAAGTTATCATATCTGATTAATATCAAATCTAATATTATACGAGACAATACACGTATACGATGTTTATTAGCATCTAATTCTGTCCAATTTAACCATTTTAAAGGTGAATGTTGACATGCTAAACGTAATGTAGCTGGTGAAATACATTTTTCAATAAAGTTCAAATTACAATTGAACATAGTTTTACGTAAGTAATCACT

>JAP03738.C|Supercontig_0000248:129242-129392

CCGCATAAAAAAATTAACACTGCCATAAGCCATGTTAAAACACCAACATGATATGTTGTTCTAGTCACAGTAACATGTTGACAACAATCACATCGAAAAGATTTTGGGGAACGACCAAGTTTTTTAGTAGGATCTATTTCTGATTCAGGAA

>JAP03769.C|Supercontig_0000008:1056401-1056816

TAGATCTGTATCCGGATTGTGGTTTAAATCTTCTACTGTAATTATTTCCTGTAATTGATCCTTGACCATTGTCAGACGATATGGTAGAAATGGAGGATTCCGGGGTGGAGATCCTTCGATTGAGTTCGCCCGTACTACGGTACCGGTTCGGCATTTTTGTGATTGGCATAATGTTGGAGGAATTGGATCCCAATTTACTTCTAATCGCATAAGGTTGTTTTGATGATGAACTAAGATATGAGTTTTTTCTTGAGTACGTACCGTTACAACTTCGGACTTTGGATGGACACCACACATCAGATTTTGTAAAGCTGATGTTACTAAACGATGTATGGTAGAGGAGCGTTTGAGTTGAGCTGTTTCGATCTGATCTATACAAAGGTGAAACATCTTGGGAATAATTTATCATGATTTTA

>JAP03855.C|Supercontig_0000136:37918-38206

AGTCATTTACCAAATGGACCAACAGCTACATTTCGTTTAACTAATGTATTACTTCGTCGTGAAATGCGTTCTGCTAAAAAAGTCAAATATATTGAATCAAATGTAATACCACACTTGATTACTACACGTTTTATGACACGTCTTGGTCTACGTACTGAACGTATTTTAAGTTCATTATTTCCTAATGAAAGTCGTTTACCACCACAACCACATAGTAGAACAATTGTTTTTCATAATCAAAGAGATTATATCTTTTTTCGACATTATCGTTATATACATCGTAATACAA

>JAP04007.C|Supercontig_0000156:96002-96125

AGTGTAGAAGAACGATCAAGTTTATTATATGAATGTTTATCTCCATTATTACGTTTGGGTAGTGCATTCCCAAATTTAGCACCTATAATTGCACGTCTTCTATTATCAGTTGGTTTAATGATTA

>JAP04051.C|Supercontig_0002058:24914-25092

TAGATAATGGGGAAATATGAAAGTTACGTTTTTCTCCACCAACACGTTTAAATGGTAATAAAGGAATAGGGGGACATGGTGAAAATAAATATCGACGATGACAACTTTTACCACCACCACCACCACCTGTACGATTTATAGTTGAAGGTAAACGAAGTTCAGTGAATCGTCTTCTTTTA

>JAP04089.C|Supercontig_0000276:181038-181229

GTATTGGTATCAATTCAATCGTTAATTTTTGTACGTGAACCTTATTTCAATGAACCGGGTTTCGAGTGTACTATGGGAACGCCTAGAGGTATAATTGTCAGTTATAAATATAATGCTTGTATTCGTGTTGCAACTGTTCGTTGGGCTATGATTAATCAATTAAAGCATTTACCAATTGGTTTTGAAGAAGTG

>JAP04094.C|Supercontig_0000398:22993-23189

CATACACTGAACTAGCTACAACTGTAGTAAATCAAGTCATTTCTATTCCATTTACTGGTAGTGCTAATCTTTTGAAATTTCATCTGGCTTTACTTGCTGGTAGCGTGGAAACAGTATCTCAAAAATCAGATAATGTACAATATCGTGTATTTGATGCAATTAATATTACTTGGCGACCACAACAGATAACTATGGAA

>JAP04095.C|Supercontig_0000160:1398493-1398672

TTTATTTGTCTATTTATTATTTTACGTCGTTATTTCACTTCTATTCGATATAATCGTAAAGGTTATACATTAACTGTTGTAGAAGTTGATGAAGTAATAGCGCCAAAAGTGAATTCACCACAATTTTTACCAGTTAAACAAAGTCGTCTTAGAAATATTCCAAAATCAATAACAAATCAT

>JAP04114.C|Supercontig_0000261:411830-412160

AACCTGTGTTAGCATTTTGCCTACCAGGTAATCCTGTGTCATGTTATGTAACAGCGCATCTATTTGTTTTGCCCTTATTAAGGAAGTTGGACTTAAGACCTCCTGAAGAATGGTGCTTTCCAAGTATTCGTGTACGATTGTTACATTCTGTTAAACTGAGTGATCGTCCAGATTACAGACGTGCGAGATTGGTTTGGAAAGAGAATTCAGAATATAACCAACCTTCCGTACCTTGTGCATCTTGTGATATGATGGAGAATCAAATGAGTTCTTGCCTAGCTAGTTGTCTCAATTCTAATTTGTTACTGGTACTACCTCCCCTTGTTAACGA

>JAP04131.C|Supercontig_0000311:811236-811648

ATCTTCTACAGCTATGCTTCGTGATTTAGAAATGCAAAAAGCTAAAGCTCAGCATGCCCAACTTCAGCTCCAATTACCTCCACCGCCACCACCACCACCTCCGCCTCCACCACATGCCATTGGTCTTCCTATTCCAAGTTTCCTTCTTAATATGCCTTTAAACACCAGCTTGACTAACATGCCGCCGCCTCCACCTCCGCCACCAATTATGGTTGGAGCGAAAGATACGAAGCCCTTGCTACCTCCCCCTCCTCCACCACCACCTTCATTCTCGCTAGGTGCTCCGCCACCCCCTCCACCGCCGCCTACTTTTGGAGTAAATTCTAATGGTTTAGGGACAAATGCTTCTTCAGCACCAATCCCACCACCGCCACCACCAACACCCCCAGCTTCTGTTCAACTGAATGGGAACT

>JAP04217.C|Supercontig_0000481:423660-423930

CATGGATTTCGGAAAGGAACAACGGATTTCGCCCAGGGTCTATCAATTAGCCCAAATAATTTATCGGGAGTTCGAAGTCATGAAGAAGGTCTTCGGTGAATCCGCATACGGTGGATTGGTACCTATTGTTGTTGGTATTCTGGAAGAAATGGACCTCTTGACTGTGGATAAGCAGAAATTGGATCTTGATTTAAATCTTTTGACTGCCGAAAAGAATGACATTTCTGTACAGTTGTGTCGTCAAAAGGAGCTTTACATAGCTGCGAAAAGA

>JAP04230.C|Supercontig_0000249:20490-20626

ATATCTGTACCTATGGGTAGATTTGTATCACCGCCAATATTACGTAATGTAATACATAATTTATTTAATATATTAATAAAACGATCTATACCGGTATAATCAATTTCACCACGTTCCCATGCATACATACGTTCATT

>JAP04308.C|Supercontig_0000183:38108-38212

ATCTTTACTGAAATACGCTAATAATCTTAATTCGAGTTGACAAAAATCACCAGAAATAAGTAAACCACCAACAGGTGCATGAAATGCACAACGTGGTCTTATAGT

>JAP04460.C|Supercontig_0000248:129242-129392

CCGCATAAAAAAATTAACACTGCCATAAGCCATGTTAAAACACCAACATGATATGTTGTTCTAGTCACAGTAACATGTTGACAACAATCACATCGAAAAGATTTTGGGGAACGACCAAGTTTTTTAGTAGGATCTATTTCTGATTCAGGAA

>JAP04796.C|Supercontig_0000365:277897-278089

TTGGATGTCGAATTGTATCTAGCACGTCTTGAGTCTCTCATGGATCATCGTCCATTACTCTTAAATAGTGTCCTGTTACGTCAAAATCCTCATAACGTAGCTGATTGGTTGAAACGTGTTGAATTATTGAAAAGTCAAGGAGCACGTGAACAAATTGCAGCATTCATGGAGGGTATTACATCGGTTGATCCAG

>JAP04798.C|Supercontig_0000042:1723654-1723763

TGTCAAATACTCTGGTGGTGTATCCGATGAGAAGGTTCCATCAGGAAACGAAATACGGCAGTATAAAGGAGTTACTCGTCGGATAGCAATAGCGGATGAAAATATAGGAT

>JAP04824.C|Supercontig_0000180:274712-274889

GATTTTGCTTCTGCAGTTCTGATGATGGACGTGTTTTTACGAAATGGTGAATGGAATTCTGCTGTTGCTGTTTCATGGGAACTCTGCCTTCAGGAATATTTCAGCTTAGAGAATGTCAGACCCCTTGTCTTTGCTGCTTCACTTTTTTCATGCATAAAGTCAATAGAACACGATTCGT

>JAP04827.C|Supercontig_0000248:127195-127305

TTTAGATACATTTAACCATACCGATTTCTGTACCACAGAATGGACATTCATGTTTAACATCTTTACAACAATTTGTAAAGAAAGGTATGAGAAAACAACCTAGAATGCCAC

>JAP04875.C|Supercontig_0000673:234668-234885

AACAGTAGTAAAGATGGCAGTTTTTACCATTTTCACTGCTTCGCCTGCTTTATTGATATATAATCGATCTTTCATACTAAATAATGATTGATTGTTTAATTTTAACATATCGCCTTCGTCATGTGGACAGTAGTGAAATGTAATACATTCCGGAAAGCGACGATTTGACGTGATTTTCACCACCATAGATAAAGGAATTGATCGATAAACAACAGCAT

>JAP04885.C|Supercontig_0000195:308780-309036

CCCCATAATTCTGCACCACATTTATCACGTACACATGTAGCAAAACTTAATGTTGATGGTTGTGAATGATCACCTTGACCACGTAAACAACAACTATTCATAACTTTCATAATTTGTTTTTTATTTTTTTGAAAATTTCTCCATAATCCACGTATTGCTTTAGAACATTCTTCATATTGACTACCACATTCTTGAACACATAGATCAAATTCAGGTTTACTCAATTTTTCACCATGTGAATCAGCATAACAAGAATT

>JAP04906.C|Supercontig_0000012:1612757-1613065

ATTATTATTATCAAATATATTTTGATTAACAGACGTGGCTATTGATGTAGTAAGAGATATAGATGGATAAATAAAACCACGAAATGGTAATATAATAAAAGATAATAGTATACCTGATAGAAAACTACCAGCATTTGCATAATTATCAATCCATGGTAATAAACCAAATGTGAATAATATAAATACTAATAAAATATTTCTTAGTAATGCATACCATGGATGTGCTAAAAATTGCCAACAATGGATTAAATCAACTAATGAGATACCGAGTAAAGCGAATTGTGCACCTGTAGGTCCAGTTTCTACCTA

>JAP04907.C|Supercontig_0010613:1089-1197

ATTTTTTGGCTTTGATTCTCGTGAAACTACTATTGAAGATTGGAATGAAAGACGGTTGCGATATTTAATAAAACGTTATGGAAGCATTAAAGGTGATCGCTTAACTGTT

>JAP04921.C|Supercontig_0000346:57905-57981

CCTGGTTTACTACATAATAAAGGAAGACCAATAATTAAAGCCAATGGTCCAATATGTCCAATAAAGCGCATTTGTCT

>JAP04951.C|Supercontig_0000381:445451-445701

AAGATTTAAATGCACTTTATTGCTCACAATATTTGAGACTTGGTGACACTTCTCAGGCTGCAAATCTAGGCTCAACACTGCGACCAACTCCAAATGAAATGTCCATCGCACATAGACTAACTGAAAAACTGATTAAACTGACGAAACATGCTCGTCCAGGGGATCTCATCCATCCATATACTGTAAGAAAAGCGATGGGTTTGAATCCAAATGAGTATTTTTTACCGGAACATTTAATTTCAAGTGATAAT

>JAP05004.C|Supercontig_0000001:669782-669863

ATTTAGTTCATCAAGAAATTACAGAACTTGAAATGAGAGCACGAGCTATTCGTGCAATGCTTAAAACTAAATCGGATCAACT

>JAP05066.C|Supercontig_0000036:898847-899008

CTTATCTCTGAATCATATTCTCCTGTTCAGTTAGATATTACTACATTGAAAGCGATTTCTTTCAATGAAGTGATAATTGTCAATAATCCTCCACTAAAGCCACGTTATTACAAATCTGTTTTACCTGATGTTAGTATTACACAATGTATCGTTTGTCATAAG

>JAP05105.C|Supercontig_0000265:79323-79424

ATTTTATAGCTTCCAGTTCTACAGGAAGATTTGAAGTGGTATCTGTAGAATGTCAATCAGAATCTACATATGATCCAGAACAAGATGATAAGGGCGAAAACA

>JAP05140.C|Supercontig_0000123:1122586-1122751

TTTTTATTTCCACGCATCATACCAGTACTGTATAGATTATGCCCAGCAGTCCCATTCATATTATAACTTGGTCGCATAGGTCTATTATTAAGGGCTGAATGATATGGATGATGTTGCATAGAATGTGGATTATTATACTGCATCATATTTGGATTATTCTGATTGA

>JAP05161.C|Supercontig_0000176:290339-290523

ATGGACGAAATAATGGGCATGATTCACAACCAACAGATGTAATAAATAATAACAATAATGGAAAATGTAAAGATGTAGCTAGAGGTGGTGACTTTCCTGAATCATCTGACAGTGATCATAAATATTCTGGACGTTGTTCCGATAGTTTATTAGAATTGCCAGGAAGTCAATTTGCGTTTGCAGGT

>JAP05198.C|Supercontig_0000104:183021-183151

TAATAACCTCGGAAATTATCATCATTGTAATAGTTATATAGATCATGATTATACCATGGAAATGCTTCACGATTCATTGAACGCTTACCCAAACGTGTATCCCATAAATAATTACGTTTCATCATATGACG

>JAP05289.C|Supercontig_0000058:1560789-1560897

TAAAATGATTATTTACTGAGATCCAAAATAGCTTGACTCTCTTCAAACTCCTTAGTATTATCGTATTTATCCTGAGATTGATCTGCATGTCTACAAGATACAGTTTGTC

>JAP05292.C|Supercontig_0000225:413810-414023

CATCAGAAATACCATTTGTTGTTTGTGATCGTGGCAATCCTTGAAATCGTACTCTATGAGTTGATGCATCAAATGTTTGCTTGTTATTTGGCATAGAAGATTGTTGTACTGGGTTTGAATTTGTAATAAGTCGGCTACTTGAAGAACTACGTCCGGATACGTTCGAACTGGTACAAAGTGACTGACAATATACACCTTCATCTATTAGAAAGCA

>JAP05304.C|Supercontig_0000044:1978049-1978202

ACTAACCTGAAAACAGTTGAATCCAAGTTGAATGGATATTCAACAATACCAGTTGTTGGTACACGTACACGTAAAATATCTTGTAGACTTGGCAAGTAATCTGGATCAGCTATTCTGTCAAAATTATTCAAATAGCTAAAAGGAAGTATAAAAA

>JAP05305.C|Supercontig_0000130:2152941-2153066

TTGAAATTAATCGATGAATCTTCACCTTTTCCAAAACATCTTGAAGGTACAACTCTATTTCAATGCCCAACATGTATAAAAGCTTTTATTACTAAAGAAGGTTGGACTTCTCATATACGTGAATGT

>JAP05320.C|Supercontig_0000005:1730834-1731066

AGGTCTGGAGTACCAATAGCTGGACAACCTCATTTTATCTATTCACATTATTTAAAATGTATCAATGGTTATCCACGTTCATGTCGAGCATTTCATGCTATTGATTTGGCGTTAAAATCACATCAGTATTTGGGCGATGTGATTTGTACACGTAGTAGAACATCTGCGGCTGGTGTTCCAGCTGATTATGCATTAAGACATGGTGTTTATTGTACTGTATTATATACAATATT

>JAP05326.C|Supercontig_0000130:103704-103807

CGTCTTTTCGTTCCAGTTATGGTGGATGAACTTCACGTTCTTAAGAAGAGGATTAGAACACTCAGTGAATCTTCCAGGTATGATTTTAACAAACGTCTGTACTC

>JAP05341.C|Supercontig_0000665:125802-125900

TTTCAGTTTTCCTGTAATTAGTTCAGTTAGTGAAGTTATCAGTACTCAAGCTGTTATCCCAACATCTATTGCCGTTACTTCTATAGTAAGTCTACCTGG

>JAP05398.C|Supercontig_0000227:350781-350941

AGCTTCTTATGACTCTTAGTGAGCCTTCATTCAAAACCAAAGATGAAGCTAAGGACTTCTTGAAGTGTTTATGTGATCAAGAACTTACTGTCGATGTATCAGATGGTCGAAGATATATTGGATTCTTATGTTGCACAGATAATGTTGGAAATATTGTAATG

>JAP05418.C|Supercontig_0000045:71514-71799

GCGAGAAAAAATTGGGCAAAGTAATAACTCCTGACCCCTGGAAAGCTGGTGCACGGAACACAGTCAGTGGCGGTGGGAGGAAACTTAATGAAAATAAACTACTAACTAGTTCCAAAAATCGATTTAGTCCTTATTCTAATGCGTTCCCAAGATGCAGAATTTGCGCTCAAACCGTACATCAGCCAGGTTCCTATTACTGTCAACAGTGTGCATTCAAAAAAGGCATTTGTTCAATGTGTGGTGTACGTCTGGTTAATACCACGCAGTATAACCAATCAGCTGCCTA

>JAP05444.C|Supercontig_0000491:515703-515930

TCTTTTTATCATATAAATCAATTGGATTTTTTTCATTCATTGATTGTTTAAATTCATTATGCTTTTTTTCTAATTCATTTCGATAATCTAATCGACGTTTTTCAAATTCTTCTGATAAATTTGGTAGACCAGAACCAAATACCCATGGATGAGCTAATGTTTCACGAGCTGTTAATCTTGATTTTGGTGTAATCACTAAAAGTGCAGTGATTAAATCTTTGGCATCTT

>JAP05461.C|Supercontig_0000672:411718-412105

TAGAGGATCCACGTATTACATTTTGGCGTGCAAGAATAGGTCATTTTCAATCAGGTGAAGTAAACTTAGGTAGAATAGTTACACCAAATCAATTAATTGCAGAATCAAAAGCAAAAGCTGCACAATTTGTTAATACACATGTACAAACTGGTCCCGAATTAATTAAAACATTACCACCACCAAAAGGTGCATTACTTCATCGTGCTATTCAAGTTGATTTTCTAAATGCTAATTTAGATGATCGAGGCACACAAACAGAATCACCAACTGTAACAAATATATTTAATTTTAAAATTGATGCTGGTACACAATATGTAAGTTCGGATGTAAATCCACCAGTTAGTATTTATCGCTCAATTAAACAAATTTTAGTACAAAAATGATATTC

>JAP05596.C|Supercontig_0000451:79798-80044

GATGTTGATGCATTACCATGGCAATTTCAAGTGGAAGAATGTTCATTACGTACTAATGTTGATCAATTTAATCAACGTCATTATACTGATCATTGGTTTGATCCTGAATATAAACCAGTTGATAATTGTTTAATGAATGTACTTAGTCAATCTACCGAGTTATCTAATGAATTTAAAGAAAATTATGAAAAATGGCTTGAAGAAGAAGTATTCTCTGTACCAATTAATTGGTCACATGTATTAGCTC

>JAP05603.C|Supercontig_0000019:1772821-1772961

TTATATCAGAATATATTATGCTATAAAAAAGACTAGAGGCAAACACCGCCGTATATGCAAACAAGTGGAAGAATCAACATGGGTGGGAAGGGATAGTTATAGCAGAAGCATCTAATTATATCCAATAAGTAACAATTAACA

>JAP05608.C|Supercontig_0000344:261377-261539

GATTTACATAAAGGTGATGTAATTGATGTCTATGAATTATCACATCGTTTATCTCAATCAGTATCTTGTGCACCTCCAATTATAATCCATCGGACAAGTTGGAACAATCAATATGGGTTAGTATGTAAAGGACCTGCACTTTTAGCCCTAGATGATGATTATG

>JAP05619.C|Supercontig_0000417:165006-165112

CAATTTATGGTAGAGATATTCTTCGTAATGCAGTTCATGGATCATCTAATAAAGACGAAGTTCAGCGGATTCAGAATTTATTATTCAGCGGTATCGAAGCTAGTGAA

>JAP05621.C|Supercontig_0000380:315125-315239

TGGTTCAGCATTAGGTTTAATTTTAGTATTTATTGGTTGTATCATTTTGCGTAATAATGGGAAGTCGTCACCTTTACCATTTCATGGTCATATACCCGGTCAACGTGTATAAAAA

>JAP05625.C|Supercontig_0000088:137793-137955

TAATCGAAAATTAAGTTATACAACAAACGATTTACCAAAAAATAATGCTTGTTTATTGCTAGATTACTCAGAAGAAGATGAAGATTACCAACGTTTGTCTGATTTTTCTTCCATATTGGCTGATTTAAATCTTGCGCATGAAAGACCTTTGACTAGTTGTTAG

>JAP05628.C|Supercontig_0000308:794426-794703

GATATTAATGCTTCAATTAAAGGTGCACGTTTTGTTAGATTTTGTGATGCATTCAATATTCCATTAATAACATTAGTTGATGTTCCAGGTTTTTTACCTGGTATAAATCAAGAATATGGTGGTATTATTAGACATGGTGCAAAATTAATTTTTGCTTATGCTGAAGCAACTGTTCCAAAACTTACAATTGTAATTAGAAAATCATATGGTGGAGCTTATTGTGTAATGAGTTCAAAACATTTACGAGGTGATATTAATTATGCTTGGCCAACTGCTGA

>JAP05632.C|Supercontig_0000076:927546-927859

ATAAACAAATAAACATTCGATTTAAATAATCACGAAAACCTGGTAAACCAATGGCATATATCATTATATTAATAATCGAATCAAATGTTAATAATAAATCTGCAACCAGTTTCGCTAATTGGAATTCAATCTTTAGATTAGTATAATGTTGTATAATAACTAATATAAAACTAGGCGTTTGAAAGCATAAAAATGAAATAGCTAAAAATAATATAGTTCTAGTCGCTTTCATTTGATGTCTTCGACGTACATTAGTGACTTCAATAATTTTAGTATCACGCCATGGTGAAACATAAAATGATATAATAAATGAC

>JAP05633.C|Supercontig_0017639:1104-1494

TATGGTGCCTTATGTAACATCCTTCAGTCAAATAAACATAACAATCAACAATATTCTGATAGACGAAATAATCATTATCCTTACAAAAAATCATACTTATCTAAGAGACGTAGTATTGGCAGTCAGACAGACTTTACTGATATTGAATCAGATAATCCAGAGGCATATTGTACTTTAGATAGATATATTATGCAGAAGCATAAGAGATATCCATATACTACTAATAATAATATTAATGATCACATTTTATCAAAACGCACAGCCAGTAATCACGTTTTGCCTTATTTTCGAAGCAATAATTTAAACCAAATGGATGGAATAGTCATCACCAAATCAAGTATTTATTCAAATAATTTATTTAGTCATAGTAATATCACTATGAATAATAATA

>JAP05650.C|Supercontig_0000238:208316-208467

TTTGGATTTATAGGGAATAATAATGACATTTTACGTACAATCTCTTTGAAATCTTGATTACGACGGTCGATTTTTTCTTTGCCAGCAGCCAAATGACAATTTACAAAGCATAATGAAGTATTAAATATAGTTAGTCTAAGACTTACACCACC

>JAP05663.C|Supercontig_0000064:660146-660324

GATAAACTGGATAGGTTTACTCATGAAGCAGGACAAACTTCAAATAAAGAAGTACAACGATTAGTTGCAAGTATATTTTATTGTTTAAACCTAGTCGCTTTACAGTATATTTCACCTTGTTTTCTTTTCTTATGTCTTTTATGCTTATATAAAAATCTATCAGGTCTATCATGGTTACC

>JAP05664.C|Supercontig_0000043:99309-99477

TAGGTAATATACCGACACTTCACGACCTACATTTAAATAATAATCCGAATTTGAATAGTTTACCTGCTGAACTCTCACTATGTGGAAAATTGATTATATTGAATTTAGAAAGTTGTCCATTACGTGCTTTACCTGAACCAATAGTTCAAGGTGGTTCTGCCATGATTAT

>JAP05681.C|Supercontig_0011744:182-315

AGTTGTCATTTAACGCCATGGTTCATATAATCGTATATCACCTTCTAATTGGGCAATTTGTAAATCTTTTTCGGCTAATTGTTTACGAAGACGTTGAATTTCTCTTCTTTGTTCAATAAATGCTTCACGTAACT

>JAP05682.C|Supercontig_0000651:160235-160605

TGAGTGAATTTCCACCATTTACGCAGTTCATTAACTACATTACTTCCTTGGATTAAATTAATGTTATTTTGTTGACATTTATTTGAATTAAATGTTGACAACTTTGAACTGTCCATAATCACACCAGGAGGAGGCAACTGATGTAACCAATTTCTTAACCGAGTCAATAATATTCGGATTGTATGAGGTAGTAATGCTTCTTGAGGACTTTCATAATCCAAATAGGCTGGTTGACGAGAAAATATTTTAGCATTGTTTGAAGGACGCCATCGTTTGGCTGGATCAGGTGAACGTGGAAAGTATAAGCCACAGAGAGCGCTTGAAGAAACGGGAGAATCACTTGGTTTAAACATTGCAGGAAGTGTTGAATA

>JAP05689.C|Supercontig_0000045:374021-374343

ATAAATTTCTTCCAGTTCCGACAATCAAACCACTTGTATTATTGTTACTACCCCCGCAACTATGTACAGAGTCTGAAATTACTGTACCACCTCCATTTTTAACTAATGATCCTGAATGTGACGATGTCCTGTGTTGTGTTTTATTATTTAATCTAATCGTATGACAATAATCGTGCTGGTTATCGAGTGAAAAATGTGAACTATGACAGTCATGAGATGCATTGATTAATTGTGAGTGTGCACTCTGTTCAGTCATCTTTTTGTTATAACTGTGATGGTGGTACCGATGAGAGTGAGTATGCGGCGGTAACTGATGTGAATGT

>JAP05693.C|Supercontig_0000241:878550-878661

CATGCTTTACAAACTTATGGTTATGGCTTTATATTTTCATTGTCCGGTTATTTCGGTGTACAGTTTGTACTGTGTTTAGTTCATTCACATGGAGCATTAACTGCTGTAACAG

>JAP05708.C|Supercontig_0000103:529186-529368

GATTATCAATCCACTCATAACACAGCTGAAACAATCATATCAGAATGTCAATTAGTAGAAACTACGAAACTCATGCATTTAATTGAAGAACGTAATGAAACAATGGATCAAGCTAAACGAATGCTTGCTGTACATAAGGATATGGTGCATCGTATTGAACGTGCACAAAATGTATTACAATTT

>JAP05724.C|Supercontig_0000002:159686-159825

GATACTGATAAAGATGGTAAAATAAGTAGTGATGAATTTCTTACAGCATGGAATGATTATTTTCTTAGTGAAGATCCACAAAGTCCATATAGAATGTTTTTTGGTCCAGTTATTTCACGACCTACAGAAGCTAGATAATT

>JAP05739.C|Supercontig_0000096:304101-304290

AGGTCAATGATACGCCAGTAGATAGTTTAACCTGTAGTGAAGTAGTAAATCTTATTAGTCGTTCAGAAAAACGATTAGATTTACTAGTACAACGTCGTATTACAAAGCATATACAACGAAGTAAACATTCACATCAGAATGATTCAAATGATCTACCGAATAATTCTTTATTAACTGGACTAGGAGGGGG

>JAP05747.C|Supercontig_0000091:14758-15062

ATTACCAGTAGATATAAGCAATTGTACCGAAACTTATAACGGCAACTACAAGGATGCTGCTAATTACAGCACCAGCTGTACTCTTTACATAGCCGCTTTCTGCATAAAATCGGCGAAGATATTGAAGCACTTGAGTTGGAAGCCATCTTTTGCGTGTCATATCTGTTTCACGCATTCGCATACGCATGACTTCGATTTGTGCACGCATTCTGGCTTGCCGAGCTAGACGAGCAGTACGGCGTTCATGTAAATCTATCGAAGCAGCTAAATATTCTTTGGTATATGAATCGGAATTATCTTCGACT

>JAP05759.C|Supercontig_0002065:714684-714839

ATTATTAAAGATTGTCAAAAATATTGTGGAACATGAAGGGGTGCCAGGTTTATTTAGAGGTTTAGGACCAAATATTCTTAAAGTTCTACCGGCTGTTAGTGTTTCTTATGCATGTTACGATCAAATTAAGGCATTTCTACATGTTTCAAAATAGAT

>JAP05766.C|Supercontig_0000130:1702483-1702646

TGATGACTATGAAAACGTATAAATGTCATAATGAATCCAATTATTATACCCAATAGTATAGGACCAAATGCTATTAACCAACCGAGTAGAATAAAATTATTCTGAATTGGATCATTTAATGGTGCTCGACTGTATGTACGACATTGAAAATCAGGTGGTTTTTG

>JAP05776.C|Supercontig_0000003:1301876-1302095

TTGATCTTCCAATTGTTTAATTCGTTTCGACAATAACGCTAATTGTTCTGTTTCACTGTGATTATTTTTATTTTCTGATGAAATATTTTCATTAGTTATAGTATCACGTCTATATCTTTTGACTAATAAAGATTCAGTCTGTTCTAATTTACCAGTATCAGTAATATAAGCTGTACCATGATGTGGACTTAAGAAACCGCTTGTAAATGGACATTCTTTC

>JAP05781.C|Supercontig_0000040:916749-917098

TTATAAATCTTCTAATATACCATAATCTATAAACAATCATAAGACCAAAGATTTCAGCAAATGTTGAAGCTGTATCTGCTTGTAATGTAATGTGTAAATAATTACATAATTCTAATACTTTCGATGAATTCATCATTCTTGATGGTCGATTAGAATGTCGATATATATTATATATATCTAATGAAAAACATACAGAACATATAAGACCATCTATAATGAATAATAATTGATGACCCCATTGACGAATACCCATAGCCCATATTTTAAATGGAATTTCCACAACAAATAATGATAATATAAATAAACTTAAACATTCTAAAGCTAATTGACCTTCTAAAATTAATCGTTGA

>JAP05784.C|Supercontig_0000121:837919-838370

TTTCAGATGTCTTTGAATGTCATTAACCAGATTATATCACAGTTGACAAGCCACGGAACATTTAACCAGGTTTTCAGAGGAATCCAAGCTGTTTCACTAACAGATAACTGCCTAACATGTAGATTCAAAGTTACAAATTCTGAAGCCAACTCACTAAGCACTTTACATGGTGGATACATCCTTGGGGCTATCGATTTTATAACTTCTGTCGATCTGATGAGATTAGGGTGTATGAAACACGTTAGTGTTAATCTTGAAGCATCGTAAGTCATTGTATATGTATATATTCTAACTAAAAATTATCGAAGATTTATAAACCCAGGAAAATTGGATTCATGGATTCGGTCAGATTCATATATACTAAAGAAAGGGAACCGTATAGCCTTCTGTGAAATCAAATTCGTGAACGAACAGTCAGGGGAGCTAGTTGCACGTGGGACTCACACAAAATA

>JAP05795.C|Supercontig_0000610:46851-47110

TCATCTATCAAAACTTCTGTGCAAACTAATACTGTACCAACTTCCTGTGATAATTCACAATATTGTAATTTGAACACTGTGGAAACAACAACACAGCATGATTGGTCAGCGAATGTAGATTTCACTCACAATCAAACTCAAACAATTGATGAAGATATAGAATTGTGGTTGAACAGTGTTGAAACACAAACAAATCTAGCGTTATTTGATCCCAGCTTCTTCTCAGATATATGTGTTGGAGTGGATGATGATTTTTTCCA

>JAP05797.C|Supercontig_0000063:747020-747333

TTTAATACACCATAACTTCACATAACAAAGCTGCAAACTCTTGTGACCACCTTCCACGAACTCCACGAGCTTCGACATATACATAACGACCAATTAACGGTTGTCTGCAAGGTATATGTAAACGTGGACTGAATATAGCATCATTAATTCTAGTTACAAAACCACATAGACTAGAACTTGATCTAGATAATGTTTTAGTATCGTGGTGACGTGGCTCCGATTCGACATAGATAGATAATCTTTCTAAATTTTCTGAATTGAATTTTGTTTTCTCTTCACTGCCAAATAGAGAAGACAGAAGATATCTTCCATCT

>JAP05804.C|Supercontig_0000360:134844-134991

GTGCTAAGTTCATAAATGCGATTAGCAGCTACATCTCTTATGTGCTCCCATTGAGTCTCTTTATTTCTCCCACTCACTTGTTGCAATTCCAATTGATTACGAAGCTCAGCTATTTCGTTAGTTAAAGAGAGTTTTTCATCTTGTTTTG

>JAP05806.C|Supercontig_0000093:884902-885107

TTGTATAAAATCATCTTCTTTCAATAAAATATATAATCCTTTTGTTGCCCATTCCCAGGTTATCATATTATGACTATCAGCATAACTAAACCAATCATATAATGTATTAACTAATTTTGTTTTCCATGTTAAGTATTCTTCACGATTAGTTTTATTATTACTATTATATTCAACAGATAATTGATCAAAAATTGGGAAACATAATT

>JAP05808.C|Supercontig_0000486:539067-539216

TACAGTCTGTCTTCCTTTAGATCTTTGGAAGATGATGGGTATTTAGAAGATGATGAAAGTTCCACGGACAGTGCACATTCTCCAGTTACAGCATCTGATCCCGAATTAAGACATGATGTTTACCCCGAATCCAGTAATGCAGAAGAAAAG

>JAP05822.C|Supercontig_0000063:897362-897519

AATGTATTATTAAAAGGATACAGGTGAAATTATATTTGATGCTTGTGCACGAACTCGTCGTTCTAACGATTTTTCATCCATGTAATAGAAATTATCTTCTATTATTTTTGAATTGAAATTAGTCCATAAATTGTAAAAACATTCTAATGGATTATAAC

>JAP05826.C|Supercontig_0000199:112751-112826

ATTAAAGTATCTGGACTGACAGAACTGAAAGCACATATAGCTTTGAATTTCGTTTCCAAAACACATCGTACATGGC

>JAP05834.C|Supercontig_0000456:51679-51813

TGGTATATCCAATTTTACATCACCCTTAATTTCGGATTTTACTAAAGAAATTGAATTACCACGTTGATTATTCAATGATTGAATAAGCACTCGACTATGACCAAGGAAATGAAGTTGAAATTCATATTTCAAATC

>JAP05844.C|Supercontig_0000195:1170800-1170928

GGTCCATACATTTGGAGTTGCCTTAGTATTTTATGGTTGTGGGTAGCTATGATAGTATACGGACATTCATTTTTCAGTAATAGGAATTCTGCCATAGCGATTGTAAGCGCATCTAACTCCAACTGAAAA

>JAP05862.C|Supercontig_0000370:10661-10807

ATTAATATGCTTTAAAGCTTCATATTTAGTTAGTGCATTTCATAAAGGCTTGCACTTTCCAACCAATTATGAAGGCCTTATTCCCACTCTTGAAATTAATAAAACCGAATTACAATGGAGTTTAGGAGCTTTATTATATAAATTAAA

>JAP05865.C|Supercontig_0000055:330282-330353

AACTTTGGTGGCGCTTCAGCTGTTGCTGCTTTAGCTGCTGTTGTCTCAGCGGCGATGTCAGCAGCAGCTGCT

>JAP05872.C|Supercontig_0000073:912974-913054

AGCCTCAGTGCTTTCACCGTTGTTGCCAAACGTGGGATTCGTCGATTAGGCACTCCTTATTCAAATGGCAAAGATAAACCA

>JAP05897.C|Supercontig_0000299:660784-660900

ACCTTCTGGAGATCCTGAGAAAGATATCAGGGCTCACCTTTTGCCTCTCAAAAGGTCTTATATGGAGAATTTGTCTTCCTATTCACAAGAACTGGATTTAGAATTGGGTAGACGTTC

>JAP05928.C|Supercontig_0000445:6444-6613

GATAAATGGAATAATTATAAAACATGTAAAGATACATCAAAATCACGTCGTTCAATTGTTCCAACTGCCAGTAATAATAATAGTTCAACTGTCAATAATAATAATAATAATTCCGGTAATACAACACGCCCAACTTCAGCTAATCTATCAGGATCACCTGTAGCTCATAC

>JAP05965.C|Supercontig_0000674:262515-262693

CTAATTCGATCAATGAATTTTCTACTTTTGTCACTAAAACGAATAGTATCTGGATCCCATTGTATTTCTAATGCTTCTAAATTTGGACAAAATCCAGCAAAACTTTCCAGAATTTTATCAATATGAACTCGTGTGTTGTATTTAAAAAACCAACCATGTGACGCTCCAAGTACACAAAC

>JAP05991.C|Supercontig_0000491:494853-494958

TTCATATTGGTGAAAATATACCATTTGGACAAATCTACATCCAACTGAAATCGATTCGTCCAGGACACGTATATTCAAACTGGTATATACTATGCTCAGATCCAGC

>JAP06000.C|Supercontig_0000071:471011-471241

GAAGCACGTCGTAATTCTAAAGCTGCTGCAATTATCTCGCTTTCATCAGACTGAGAACTGGTACTGCTTACATCTAGAACTACATCACCTGAGACACCACCGCGTAAATAATTGCGACATTGAAATGTTAAATGGCCGGCTGTATCAGCCGTTAGTAAGTATGTTGCTGCTTACTAAATCCACATTTTTTACAGCCCACTCGAAAGTTCGTCCTGGATTCACGGGGTTTAG

>JAP06008.C|Supercontig_0000300:595789-595874

TTACGCTCAGGTGGAGCAACTATCTTGATCTTCATTGTGCTCGGTGCCAATGCTGTGATCTCTTTCTGCATACGATCAGCAATACC

>JAP06009.C|Supercontig_0000191:92864-93088

TAGAAAGCATAAAGATCTTTCACCTAATCATCAATTATTGTATTCACTAAATCACAATAAAAATGGTACCAATAATTTGGTCAGTCAACAACCAATTCGAATAATTACTGAACTAAAAGCACGGATTCAACGATTGAGAGAAGAAAATATGGCGCTACGTCGGCTGCTTTTATGTCGACAACTTGTACCGGTAAAGCAATCAGATCAAGAAAGTGAACAACAACC

>JAP06044.C|Supercontig_0000263:345535-345809

TTTGTAATACATTAATTGATAATCAACGTTGTCTAAGCGTTGAAAGTCCAGATCTTCATGATGCTCAACAATCGGCAATGAGTAGTCTGCTGAATAAATTATGTGCTACTCAACCTGTGGTTGGTTTAATATTCTCACGGGAAGGTAAAGTTGTCATAGGCCAGTCACCAGCTGTATTTTTTCTCTTAATTGACTTATTTTTTGTAATTGTACCTTTAATACATTACCAATTGTACTGTTTTATGAATATACGAATATATTTCTTCAGTATAAAA

>JAP06092.C|Supercontig_0000307:509659-509859

GGTTATGGATCATTAGGTCTTATGACATCTGTATTAATGTGTCCTGATGGTAAAACTATTGAATCTGAAGCAGCTCATGGAACTGTTACACGTCATTACAGAGAACATCAAAAAGGTTTACCAACTAGTACAAATCCAATTGCATGTATATTTGCATGGACTAGAGGTTTAGAACATAGAGGTAAATTAGATGGAAATAAT

>JAP06131.C|Supercontig_0000055:1075940-1076133

AGGTATCCCTGGAACTTGTGAACTTATTTTAGCTGGTAAACGCTACTATCCATTTGAATTTGAATTACCTGCAGCTTGTCCATGTTCATTTGTTGGCTCAAGAGGTAGTATTGAATACAGGCTAAGAGTTTATTTATTATTATCGAATGGAGAACAATTATGTGCAGATAAAGGATTAAAAGTTTTACGTAAAA

>JAP06163.C|Supercontig_0000157:962886-962993

GCAAAAGTAACTGGAACGAAAAAGCCGCTTTGCAGCTACAAAACAAAACTCTAAAAACAGTCTCAGCTATACAAAATCTTATTCAAAATATTATAAAAACTTCACATT

>JAP06221.C|Supercontig_0000209:310899-310979

GACTGCCACCTAGTAAAACAGCTGGTTTATACCTTAAGTTCGGTCGTTGACTCCAATATATTGGCACAGAACCACGAAGCT

>JAP06291.C|Supercontig_0000064:789830-789895

TTATCCTTTTGGTCAAGCTAAAAATCGTTTTCTTTGGAATCGAAATGAATCAACTAGTTGTGGTTA

>JAP06297.C|Supercontig_0000344:82489-82571

CTGAGTTAGAATATCTAGTTGTGAAACAATATGTTCAAGTGTCGTAGATAATGGTGGTGGGACATTTTGGCGTTCACTTTCTA

>JAP06298.C|Supercontig_0000295:469411-469642

TTACATAAAAATAACCAGACAACCCTGGCTGAACGTGTAGCACGAGATGCAATGAATGTGGACCCAACAAGTTTTCGTGTATGGCGTTTACTTGCTGACATATTGGGTTCTTCAAATACACCGGAACCAGCATCTGAAGCTGTTGTTACACGTGCACTATTAACAGCCATTGAACTGGAGCAAACTGAACCAATTGAACCATTTTATTGCTTACCACTTGGTGTACGATGTA

>JAP06308.C|Supercontig_0000032:507396-507536

TTATTGTACATGACGTGAATATCTAAAGTTATTTATAAGATAAGCCATTGGAATAGGCAAGAAACGGGCAATCAAATAACATACTATAACTGTTTGAAAGAATATGAATGTTAGTAACTGAGGATGATAACAATAGGATTC

>JAP06314.C|Supercontig_0000178:641036-641220

TCGTCTTCATAAGATGGATTATCATAGACTAAATTTGTATCATTCTCATAATTTCCCCATTCTGACTCACAGTAAGCAGGACTCATTTGAGCATTCAAAACTCTTAAACTTTCTTTCTTATCTAATGCCATTTGATACATAATGGAGAGCATTGCATCTCCTGCACGTTGAATCTATTTGAAAAA

>JAP06319.C|Supercontig_0000055:511342-511476

CTTGAATTTGATTCATCTTGACAAAGCAACTTAACACAAACAGCTCCAGCATCATATAATGTCGGGTAGATTGATTTCAATCCAGGACAGACATTCCAATCACATGTAGATAAACATTTGAATGGAGTTGGATCT

>JAP06420.C|Supercontig_0000486:214507-214698

TCTTGGTCCGTAATTTTAAATTCATCTAAACCTACATCCCAAATCTATTCGTCTAATACAACTCATTCCAGTGTACCATGTTCAGGTGTGACTGCATATTCCTCAACTACAAATGCTGTAACCAGCTCAAATACAGATATGGTAGACACATTAAGTAGTCTATTATCATCTTCAACTTCAAAATTAAATTCT

>JAP06421.C|Supercontig_0015826:325-449

GAAAAATCGCATCGATATCAACCTATTGGTCCAAATCCTAAATTACTTAGTTCACGTATCATTCCACATGGTCCGAATCCCTTATTTGTTCATGGACCAAATCCATCATTAGTACATGGTTCAAT

>JAP06474.C|Supercontig_0000009:328981-329219

ATTGGTAATTTTCGTTCTGGTATTTCTTCCAATCGGTTAAAATATTCACTTATCTTATTTATCACTGTAGTCATAATTGATGCCCAATTAATTGATCTTTCATTTGAAATTATCATTAATTCATCATAAATTTTATCGGATAAACTAATTCGTTGACACCAACGATCCATGAAAGGGTCAAGATGAAATAGTTGATTTGCTGATAAGCTATTTAGTAGTGCTTGTCTTTTAAAACAAGA

>JAP06495.C|Supercontig_0000132:315288-315522

AAATGTTGAAGTTGTGCACGTAAATTAACACGCTGATCATTCAACTCAGCAAGTTCACCTTGCATTTCATTATTTAAACGAAGTAACGTGTGATTGACCGATTTGGCTTCCCGAATAAGTGAAGACAAAAGACGACGTCGACGACTACTAGATAATTCAGATAAAAACGTTTTTCGATGGCCAGTGATTGCAGATAATAATTGGATCGGATCAATATACCTAATTGTAAGATAAA

>JAP06503.C|Supercontig_0000673:242975-243143

GAAGTTGCGACTGATTTTCTAATTCAAAAACATCGCTAAATGTAGCCATGGCTTGCGACTGGGTGTCCAACCTTAGACATATGCGCCAAACGTCAGGGCGTAGGTTTTCAGGGACAGGACGCATGGCACAGATATCCCGAATCCGAAAAATGTCAGCATCCTCTGAAAA

>JAP06507.C|Supercontig_0000067:715015-715129

GGTTTGGTTTATCTCATGCATTCATTTTAAAACCTGATGATGGTGAGATTAAAGGTGGGACACGACTTAGTTTATTACTTAGTTCAGTTGAAATGGCTGTTTATGCAACACGATG

>JAP06509.C|Supercontig_0000480:213244-213450

TTTTTGTCTATATTGTAATCCAGTTAAAGCATAACATGTTACACCATCATTAGTAAATACACCAGTAGCTAATGGTGATATCATTTGTGTTGATGAATCAGATAACATATTTTGTAATTTACTAAATGGTGTAGATGCATAAATTTTTTCAATTTTTTTATTATATGGACTGGAAAATTTTGTATTTGACCGACTTGATCGAGATGG

>JAP06514.C|Supercontig_0000011:747667-747811

GAAATGAATGCCAATACCAAACGAGTAATCCGAATTCGACTATGTCGAAGATATTTATATGTAATATATGTAACAACAAGTGCATACGCTGAACCACCACCAAAAAGTAGACCTGCACCGAGGAGATGCATTGACCAAATAGCTG

>JAP06536.C|Supercontig_0000461:82434-82597

AGGCTACTTGCCTCTTTCGACGTGTATTAGCACTGTGTCTCAACGAAGATTCAGTCTCACCCGCTTTCATAGCTAGAAGTCTTGGAATAGAGTATACCGTGGCTCGTGGATTATTTAATCGTTTAATCAAGGAGCAAGTTATTAAAGGAGCAGGACCACGAAGG

>JAP06562.C|Supercontig_0000221:691343-691459

AGCCATGGATAGCAAGGTTTGATACGGATAAAGATAATAAAATCAGTATAGAAGAGTTCTGTCGTGGATTTGGTTTAAAAGTATCAGAAATGTAAGTATTAAAATCAATAAATTATT

>JAP06565.C|Supercontig_0000011:388261-388413

ATATCACATTTTTGAAAAACAGAACTCAGTTCTGTAGCTGTCAGATTAGATAAAATCAAACAAATGCGTTCATAATCCTCCATAATTCCATTTCCATTCGAAACCTTTGAGACAAGATCAAATACTTCATCAGGAATAGTCATTATGCAAGTG

>JAP06569.C|Supercontig_0002017:23996-24122

AAGAACTCAAACGGTATGCTGATGATGACTTAAACTCATTACCTGCGGGCTGGAAATCAGCTGCTGATTCGACTGGGCGATTGTATTATTACAATAAAGAAACCAATAGTGTTCAGTGGGAGAAGCC

>JAP06592.C|Supercontig_0000046:1621812-1621938

TGGATTCGACGTCTGTCATTGTTACTTTGTGCAACCAGTGGAGTTCTTTCGGTTTTGGGGAATTCGCACTTCCTCTTCGGTTCGAAAGTGGCAAATCAACTAGCTATGTGGTTGATACATGATCCAA

>JAP06605.C|Supercontig_0000196:229362-229551

ATCTACCAATATACAGAATAACCACCTTGCGGATCGACTAACAATTTCAAACTTTCTAATCGCCCAGCCATAAGACAATTATTCAGTTCATGAATTAATCTTTTTCTGTAATGAGATGATGAAATTTTGTAGTTTTGAGGACAACAAGATGTAAGTTTCTTGAGAACAGAAGGACTAAATCCAGTTGAAT

>JAP06620.C|Supercontig_0000184:122993-123143

AGAAATTGGAAAGAATGAAATCAAATGAAATCTGTTTTCAAGCATGGAAGCAAAGTAAACATCAACTTTTAATCGATACAATTAAAAAACGTAAAGCAGAAGAATTGGCACAGAAAAAGAAGCTTGAAGAAGAACAGGAACGTAAACTGAA

>JAP06656.C|Supercontig_0000156:1124898-1124988

TTTCTCAAAGATCTTATTCATTAAATAATATATTTGATAGAGATTTATCAATTCCACGTTATTCCAGTCCTCCACGTGACATTGTATATCA

>JAP06679.C|Supercontig_0000053:65030-65396

AATTTGTTAAAAGTTCCTGAAGGGTCATCTGCTGTACAGTTAACTACATCAGCAAATACACCCCTTGTCATAGGCATCGATCACATCCAAGCAAGACGTCTAAGTATGGCATTACAGCACTTATCAAGAAATTCCTTTCAACCCCAAACACTATCCTCACATAAGCACGAAACAACAAGTATGCTAAATATGGCAAGACGTTTGACAATGGCTAGTTCAGCCATTGCTTTGGGCAGTACTGATCGTGATTACTACGGCCATTCATCATGGTTAACTGGATCTAGAGTTCGACATGGTGTAAGACGTAGTTTAGCATGGAATGAGTCTGGTGCAACTCGATTTTATGAACATTGTTATGATTGATAAA

>JAP06682.C|Supercontig_0000042:1652142-1652382

GAATAATTAACATTCATAACAGAATTAGAATAAGTGCAGTTACATAAATTATTAGATACATTATTTATTTTTTGTGTAGCGCTATTCTCAAGAATAAAATCTAAATCGAGTAAACTTTCATATTCATTACTTGAAGTACTACCGCTACATTGTTCCGTACGAAAGCTACTACCAATAGATGAACATTGTTCCGAAGTCGTAGGTGATGTTAAACCTGAGAGATTGCACATGTACGTTCCTC

>JAP06690.C|Supercontig_0000024:921250-921360

GCTTTGCTTCTAAACAACGAAGGAGTTGTTGTAACCGGTTTATTAGCAACTTTGAATTCTATAGACTTCTGCTTCATACTGAAAGATAATCTGTCTTACATGGATAAACCG

>JAP06741.C|Supercontig_0000238:329956-330146

ACTTACCTGTGATAATATCATAAATTTATTAATTTGTTCAATAGATGGTGTAAAATCCTCTGGTCCATAAGCATTTATCGCACCTGATCGACGATTGCATATAACAGCTTTTTTCACTAGTCTTGTACCCAATATACTGTTTTTTAAAATTCTATCATATACATCAGAACTGGTTGTTTGATCAAACTAGA

>JAP06742.C|Supercontig_0000461:235079-235223

GCTATTCAGACAAAGAATTTTCTTCAAGTGCTTTTCTAACTTTGTTTTCAATACGACGAGCTGCACTTTGAACTTCTTCGCGCAATATGTTTATATGCATCTGCCAGGTACGATTAGCAGACTCCTTAGTTTTGAAATACGACTA

>JAP06745.C|Supercontig_0000448:81202-81318

TAATTAATTATTTTTTTAATAGGATTAACAAAACGTCCTTGGACATTACGTGACCCACTGAATTGTTGCTTGGATAATGCTAAATGTTGTAGGCGTAAAAGGTACTTTGAACGAAAA

>JAP06773.C|Supercontig_0000730:5971-6250

ATCTTAGTTTCCGACAGATGATGAATTCAAAAATGATGTTCGATTAGCTGCACCACCACCGAATTCTACAGCTTGTGAAGTTGCCTGACGTAGTAAAGATAACTGGTCCATTGAATTAGTCAGAAGATTATGTAAAGTGGTTAATTCCATATTAGTCAATTGACTAACTCGTGCTGTTGACGATCCACTAGAGAGGAAACCATTAACAGACGATAATAACTTTCCAACACGAATATCCCATACATCACGAACAATGTTGCGAATACTTTCAGGACTAAAA

>JAP06791.C|Supercontig_0000159:903443-903535

TCAATTGTAATCATCTTTTAATTTACGAGGTAGACCAGATAATGAACTTGTGAATGCTGGTTTACGTTTACGTGCACCAACATTAACTGTACT

>JAP06847.C|Supercontig_0000153:1196194-1196273

GAATTCAATAAATGTGCAATCAGTGAGTGGTTCTGTATATTCAGTATTATAGAAATCACTTGGATAACTGGTTTGGAATT

>JAP06908.C|Supercontig_0000137:270379-270493

GATGAAACTTTGTTCTTTGAAGGTACAAACTATTATGTATTCGATAATATAGCAATGCATACACGTCCTGGCTATCCAAAACAAGCTTCATTAGGTATACTTGGTTGTATGAAAT

>JAP06925.C|Supercontig_0000167:367362-367588

TTTGATGGAATAGGGGAATTATCGAAAAAGAAGCTAGCTGATACAATTTGTTCATCAGGATTATTGGAAATGCGAGGTTTTGGATTTTTTATTGTTCCCCATACCCAAATCCAATATAACGCTCCCATAATAAAAAATACAGCGCCAAATTCAATTTTTATGGCTATTAACCATAATAACAGAGTAATAATGAATTGTACATACCATAAATTCCAAATGGCAGTAAT

>JAP06933.C|Supercontig_0000025:1197278-1197623

TTCATATGTGGATCCCATTCCATTAGATAACAGCCTGACCAGATCAATTTGTCCTGACCGTCTAATATCACCGCTTGCTCCTCCCACACCAAGTAAACATGTTCAGCTTTCTTCAAAGTCACCAGCTTTATCAAAATCCACTGTAAATTCAGATTTATCTAACAAAACTAATCCCGTTGTAGATGATTGCCACAATAAGCGCAGTATGGATTCTAATTTTCTTAATGAATCTTATTCAGGGAATAATGTTTCATCTAAGCCAAATGTTCATCCTACCAATATATCATCGTCTGTCTTTCAAGAGGAATTACGAGATAGATTAAAACAACATTCTCAACAGCTTAAA

>JAP06966.C|Supercontig_0000301:187069-187256

AGAATAATTTGTTTTTGGAAGACCCACAAGTTCCAATCGTATATTTAGTCCATGAGCAACACTCTCACTGACATTAATTTTTAATAAGACATGATCGAACATACGATACTGTTGACATTCATTAGTTTTGGAGTTCCGGATTTCTAAAACCCCACAATCTGATCGTTCAGATGAATAAACACGTTCAA

>JAP06983.C|Supercontig_0000030:449340-449652

TTACCCGAGTAACAGTGGTGAACTGTAGTTGAAGAATACAGCAGCCGATATAAAAATCCACCCGCAAAATAGACGCAAACCAAAAGATGAAATAAGAGCGTAGAATAGTCCCCATGTGGCACGTGCAGGACTGGCTTTTATTGTACTCTGCTTGCATACTTCCAGATTATATGAGTTTGAGTTGTTATTAGTAGTAGATGGCGACAAGATATCAACATCAGGTGTTGCATTCAACATATTTGCATCATTCGAGACATTTTCAGTATCTTCATTATTCTCCTTAAAATCCATCAACAACGGCACATCATCATCC

>JAP06986.C|Supercontig_0000490:299698-299804

AAAGTTGGTGAAACCAGTCTGCCTTCGAATACTGGGGACTTACAGAACTGTTTAGTTAATTTTTATTTGGGTCATTCATCTCAATATGTCCGCAACAGTGCATGCAG

>JAP07015.C|Supercontig_0000020:1117866-1118090

GTAGTACTAATCATAACCGTTTCTCTGTAGTAGATCCAAACTTTCAAACACATACTTGTGCAATTTTTTGTCCTTTAAATAGAAGTAGATACCTCTTTAAACCATATTTAATTGAAGTAGACCCCTCATTTCGACGATTGGCTGGTTTAGTTACTTTATCAAATTGTAGAGATGTTATTGTTGTTGATCAAGGTGCTCAAAATATTTCCAGAATTCACTATGCAT

>JAP07025.C|Supercontig_0000123:1359538-1359695

TCACTTTCACAACAAGCCATTAAAGATTATAATCGTGTACGTATGTATTTAATTGATTTAGCTCAAGAATTAAATATCCCTGTTGCATACGATATTGAAACAGCTCTTAATTTGTGCATCTTTAAACTAACAGCACAAATTTGAATTCCTATGTAGGC

>JAP07026.C|Supercontig_0000123:957467-957628

GATTTATTTCATCTAGAATTCGTTTTAACTTAATAGCAAACAAGAGTACCCCAGTGAGGTATGATAGGAGGTGGCACATTGGCATTCGACAAATACTTTTGATAGTTAACATCCTTGTCCGTATACCTGTCCTTAAACATAGTTTCCAACTCCTCAAGACTA

>JAP07048.C|Supercontig_0000097:304003-304096

TTGTAATATTGCTTTCATGAAATATTTTCCTTCTAACACGTTTAGGAACATCCGGTACAGCTGCCATGATCACATTAGTTAGAATAGTTGCTAT

>JAP07065.C|Supercontig_0000064:2437149-2437261

TGTTGTAATGCATGTTCAGCTGATTGAAAATTATTTCCAAGTGCTTCATCTGTATCATAAGCAGATTGATAACGTGAATAACGTAGACGACAATAATAAATGAATATACCAAG

>JAP07077.C|Supercontig_0000005:81507-81620

CGTTTACGCTTTCTTTTTAGCTTCTTAAGAAAAGCACGTCGGGCACGATGTCTGGTCATAAGCAAACCAATCATGAATCAAGCACATACGACATGTGAGACAAATCCATTATCC

>JAP07119.C|Supercontig_0000374:276712-277037

GGAACTGTAACTAAACGAGATAATCCATTAAATTTGTTGAATGATAGACGATAAATACCAAGACATAGAATTCCATATTGTTGAATAGCGTGACAAAATAAATCGCCATAATTACGAAATAAAGTTATTTGACCATTGTTATCAGTTTTGATTAAACGAGAATTAGTTAAAGAGATTTGTTCAATTCGTGGTCTTTGTCTTATTTCAAGGAGACCGGGAAATGGGCGTCTATCTTTCTTAATGTTTTGAATATTCTTAGTGAATAAGGCTTTTCCTTTGAAATACCGGTCATCATGACCAATTAAACCACCACCCTCAGCTAACCA

>JAP07132.C|Supercontig_0000131:691174-691318

TTTCAAAAACTAACCTTGCAACATTGCAAAGACATGTGAGCATTTCTTGTGGGTTTAGTGCAGACAGAACAAAAATTAACGTTACTAGATCAACACCATGTTCAGCATTGTTGAATTCCTGATCACTAACTGAATTCAAAGAGGA

>JAP07133.C|Supercontig_0000131:690270-690541

TCACAAACAGCCTGAAGAAAAACACGCTGCACACACAGGTCTTTAGCTACGTTTCTCGTTTCTTTGTGAACAAAATGAAGGCGATGTACAGACAAACCAGCATTTGAGAATAAATTAGCTAGTTCGTCTTTTGTAAAAAAGTAAGACAGTGTTCCATCTTGTCTAACATAAGATGGTCGATCAGCAAATAAACGAGATCCCCGACCAAATCTCAATTGAGCATGGTCATATTGACCGTAATCACGAAACAAAAGTCGACCGCCGGGTGAAAG

>JAP07153.C|Supercontig_0000247:28437-28613

CCCACTGAAAACTCTTTGCACAGTGTTTATGTTCTATTGCTTCTCAATATGCCTTACATTTTTCAACAGCCGTTGTATAAAAGTTCGCGAAATTTCGTTGAATAATTTTAAAAGTTAGATGTTCCCGTATCCACTCTCAATCACTTTACTGCATATGATCATTAAATTCTTATTATC

>JAP07156.C|Supercontig_0000075:163868-163969

TCTGACAAATAGTTGAAAATCTTTTAAACTAGATTTTAATGCACTTGTGTAAGATTGAATTAATTCTGGAGATAATTGTTGTCGTGGCAGAAAGTAATCTTT

>JAP07171.C|Supercontig_0000131:970587-970919

TATCTATCACAGAAGGATTTGAATATACTAACTGGATTGGTATATCCCACACAAATTTTTCAGTTTTTAAGTTAAATGGAATTGTCCATACTGTACCGTAATCTCTTTTTAAACATGATTTACCTTGAAAATGATCTAACAAATCATTTTTACTATTATTATCAGATATTAAATCAAGCTCTAAGTGTAAACGCCATCGATAATCCAAGGAAATAAATGGACTAGTTAAACTAACAGAATGAAATTGTGGCGTGATATTTGTTGGAATAGGCAATGTAAAAGGTAGCAACAGTTTAGATATACAATATAATTTTATATCTGTCCATGTAGTAT

>JAP07199.C|Supercontig_0000026:1467439-1467665

AAAATGATTAGCTAATGCAAGTGCACTAGCACATTGCTTTACATACCAAAGATAGATATAACATCTTTTATGCATCAATTCGGTAGTATGTATCTTGTATATCTATCCTGAGTGGTAGGATGCAAGTAATTGAAAGTAATATGGTTTACTCGCTTTCAAAAGTTTTTTTGTTAACTGATTTTTGTTATTATTACTCAATTTCTTCTGTTGACAATATCTTATATATA

>JAP07229.C|Supercontig_0000306:473025-473144

GTTCCCATGGCATTTTCAACTGTATCTGAAGGTCGAGGTGTGAATTGTTTATTAGTTGGTGGTCCAGGCGGTCGATTAGTATGGTTGAATTCATGGACATTGGATACAGTACGAACATAT

>JAP07249.C|Supercontig_0000298:647721-647832

AAAACAGCCAGGTATATCTTCCCAGGAATAGTAAGGGGTGACAATATTTTACGAAGTACGTAACTCTCTGCTCCACTAATGAGAATCCGATTAGGATCTGCCTGTGAAGAAA

>JAP07269.C|Supercontig_0000055:359339-359495

AGACGTGAAGCTTCAAGTGTTTTTATTCATAATGATTGTGTATACATATGCGGTGGAGCAAATGAAACTAGTTGTGTAAATACCACAGATATTTTGTCATTGAAAACTACAAGTTGGAGTAAAGGCAAACCAATGATTTTACATCGGGCATTTGCTG

>JAP07305.C|Supercontig_0000064:1922669-1923060

ATAAATAGCTAACATGCATGCAGATCATAAAATAAATGCTTGTAGATATTATTTATATTCAAGATAATGGTTTAGTAGTAAGTATACAACTAATAGGTATGATGGATTGACTAGGTTGTATGGTTCGCGGAGTAAATTCTAATGTTGGACTGGTAAAACTAAGAGTAGAACCAGGATGAATAGACATAATTGGGACTGGGAGGAACCCAGATACAGTAGTCAATGAACAAGGTGAAATTGTAGGTATAGTGATACATTCACTACTGTTTATTCGACTGCTGTTGTTATTATTACTGATACAATTATTAACAATAACAGTCGCAGCAGGAGTAGTAGTTATAACACCGGCTCTAAGAGTTTGTGATGAACTATGATCTGGTTGCTCTGTTTTA

>JAP07312.C|Supercontig_0000353:82608-82912

TCGACAAGCTGTTTTTTTTCTCTTAATATCGTAAGTGTTCTTTCGTAGGCAGACTGAACAAGTTTCCTAACTTCTTCGTCTATTATTTGTGCAGTATGCTCAGAATATGGTTTGGTAAGAACCATATCACCTTGCTGAGGCAAATCGAAACTTAGCAACCCAACCTTTGAACTGAATCCTAGTTGGACAATTTGAGCATAAGCTGATCGAGTTACACGCTGTAAATCGTCCACTGCTCCACTTCCAACTTTACCGAAAAATACTTCTTCACTCGCTCGACCCCCTAATGCCAAACACATTTCATC

>JAP07341.C|Supercontig_0000093:1243873-1243955

AAACTTGAAGAACCTGATGATCAAGGTTGGTGTAAAGGCCGAAAAGATGGTCGTGTTGGTCTATATCCTGCAAACTACGTAGA

>JAP07438.C|Supercontig_0000103:329892-330023

TTGGTTATTAAATATTGTCACAGTATTAATATTTACTTGTCGAAGCTCATGTTGGTCATCAACAACAGATGATTGGATAGAAGAATGTATTATAGTTCAAACAGATAAAAATGTAACTAAGATCATTTGTCC

>JAP07467.C|Supercontig_0000235:879575-879669

GGATCTCGAGCACCTGACCCACCTTCTGGTGTTCGATGTCTTGCTGGGGCTTCAGGTTGTATAACGCAGTTAATATGTAGTGGAGCTGATGAAGA

>JAP07478.C|Supercontig_0000160:775969-776072

GATTTCAACTCGACATTCCGTTCATCTTCGCCGTCAAATTTTGTTTGGAAATTCTCAGATGCTTTTAAATCAGTTGTTTCAAATCCAATATCAATCAGTTCTCC

>JAP07481.C|Supercontig_0000007:1443837-1443950

AAAAGAGATCAATCAATCAAATCTTTATTCGATGAAGATAATATGTCAGCAGAGTATGAATTTATTATTAAACAGTTTGCTAATGTACGTATGGCTGATGTCTGGGAGATTGAA

>JAP07486.C|Supercontig_0016435:330-466

ACAAAATGGCGAACAATTGAAACATCAACACGATTTCGTAAAAACAGTCTATGCATTGTGTTTATTACAGGAAGTACATGACCACATGATAGTAAATGTACAAGTAATCCGACCAACATTCGTTTGTATTCTAACTG

>JAP07518.C|Supercontig_0000082:109444-109615

ATCGAAATTTGGTAACAATCAGTAGAACACCGAATATAACGACCAATCCAGCCAATACAAGAATAATAATCATTTTACGATCCTTCGATTGGTGACTTTGTGCTTTCGTTAAATGCTTCAAACCCTCTTCAACTCGAATTTGAGTATGTTCCACATTATAATCGATACGATC

>JAP07630.C|Supercontig_0000259:83950-84057

TCCCTTTAACTCTATCTGCTTATATAATGTATGTGTGTAAAGGTCAAGCCTCCAAGTATGCTGGTGTAAAAAAAGTGTCATATTTGACTCTATGCACTAGTATGTCAT

>JAP07905.C|Supercontig_0000046:1955291-1955466

TTTGAACATCCTATGGAGCTTACATTGGCCAAAGCATTGTGTCGTCTACCAGATATTTTATTAAAAATTCAAAATGATTTTCTATTGCATAGCCTATGCGATTATTTGTACGATTTGAGTTGTGACTTTACAAACTTTTACGATGCATGTTACTGTATTGAACGGAATCAAGAAAC

>JAP07960.C|Supercontig_0000009:937108-937288

GTATCACCAAATATACGTATTGACAATTGTGGTATAAAGTAAGTTTTACAAAATGGACAAAATGTTCTATGATCATTTTCATCTGTAGACCAACCAGCCATTATCTCTTCATCATAAACATAATGTTTACACTTTGGACATGTTGAACAAGTTGTAATGAATACATTCAAATGAGTAATAC

>JAP07977.C|Supercontig_0000479:424604-424708

TGAGTATCAATGAAGCTTTTAAACTCCATCAAGTCTTGCAATCCAAGCTCTATGCGAGTGAGTGGACGTCTCTGCATTAATGGAGGATTTTAGATGTGACTGAAA

>JAP08129.C|Supercontig_0000000:1691838-1692045

CCAACAACTTCAAATTTCGCTTTCATATGTTGATTACCATATTTTTTCACATGTCCTTGTGTATCATAGTCTAAAAATTGTCGTTTTTTCCCATAGATATCATAGCCACCACCTTTACGGAATTTAGTCAATGATAAATAATGTACACCATAATCTTGATGTCCAACACCAGTCATTTTACCATGAACATTTAATCGACTATGACTAT

>JAP08139.C|Supercontig_0000442:100716-100827

GATGATTGTTCTGATGGAGATTTCGGTAATGGTGGTAGTAGATACTTGACAGCTGGTCTTATTAATGGCCAAAATACATACCATAATCCAATGAAATAGACTGATTTCAATA

>JAP08210.C|Supercontig_0000003:830477-830784

CCCCATGAAACAGTTACAATTTGTGGTGAAATACATTTCTTCATTTTTAAAGGAAACCACTTTTTGAATTCCGAGAATATTACACCTTCCAAAAGATAACGAGTAAACCATCGGACACTCCATGTTAATGATTCCTTGAAGCCTAATGATTGAGCCGCAGCAACACTATTTATAGGAACAAGAACCGTACTTGGCAGATCCAAAAACTGATTATCAAACAGTGGTGTACTAGAATCAGGTACAACACCCAACCCGATATACGTAATAGATTTCGCACCTGTAACAATAATATCACGTTCAGAATCATA

>JAP08321.C|Supercontig_0000183:116999-117245

CTCAGATGAAGTAAAGGGATTTGTGCCCGATGATCACATACATAGTCCCATCCTAAAGCAACTCGCGCAGCTGACAAAAATGACGAGACATCCGATTCAAAATTTCCTCCCAGACTCAGAAGTACATGAAAGTCGATTTCAAAATCTTTGATGAAAAGTGGATCATTTAAGCGGGATGATATCCTGAAAAAATTACAAGACCTAAAATTACCATTCAGCTGCCAGACGAACTGAACGAAAGCCAGTA

>JAP08336.C|Supercontig_0000071:668936-669049

TATATTCGTTCTGGTGAAGTGTATAAACCTTTAGAAGTTGTTGCACAATCTTCTCTAAAAAATGATAAAGAACGTGCAAGTTTACGAAAGGCGCTTGAAGAAATTATCGATCAA

>JAP08359.C|Supercontig_0000306:65719-65809

TAGCAACACAAGTGGCTATATTGGCAATTATGTCGTCAAAATGTGTTACTGTTCGAACTACTACAACAACGAGAACAATTCGAGAAAGTCG

>JAP08413.C|Supercontig_0002065:403533-403735

TTCATCAAATTTGGATTCAGGATTAGAACAATCAGTTATGACATGTTCTGAATCATATGCGCATATGCTTTCACCAATGTCAGAAAAACATTTAGAAAATTCTCTTGGTCACCACTTTTTATATAATAGATATTTATCAAATTCTCAAGTTTTTAAAAATTACTCACATTCACTTTCAAAGTATTCACGTTTTAAAAAACGAT

>JAP08447.C|Supercontig_0000304:510064-510174

TACGACTGAATGGAAATCGCTGTGTTAATTCAGCTCAACCATCTATTAATAATCCAACTTTTGGAGCGTTTCCATCCCATCAACTTCATAAGATACATCAGCAAAAATCTC

>JAP08451.C|Supercontig_0000417:164088-164198

CAGTGGTCAAACTTTGTTCATGATTTTGACAAGAAGCAATGCAATTTCCGGTTGGCGGCAACTTATGGGACCAACAGATCCAAACAAAGCATCTGACGAATCATCAGAAAG

>JAP08477.C|Supercontig_0000066:178960-179041

ATTATCTCACGGATTCGGAGTAATAAAAATACATGTTGGGGTGCCTGTAAGTAAACTAGTGGGAGAAATACCATCAGCTGTA

>JAP08478.C|Supercontig_0000228:527731-527826

TTGAAGAACGACAACGTTTAAGTGAGAACTATCGAGCATTGTATAAATTCGCTTTTCCAGTTAAATACTTCACATGGCATGAAAACTCTTGGATAT

>JAP08482.C|Supercontig_0000130:1702483-1702646

TGATGACTATGAAAACGTATAAATGTCATAATGAATCCAATTATTATACCCAATAGTATAGGACCAAATGCTATTAACCAACCGAGTAGAATAAAATTATTCTGAATTGGATCATTTAATGGTGCTCGACTGTATGTACGACATTGAAAATCAGGTGGTTTTTG

>JAP08514.C|Supercontig_0000144:200379-200531

GCTAATACAGCAAAAGCATCACGTTGTAAACGACGTCGTTCAACTCTGGGTAGTGGAAGTTTATCTAACATAATGAATAATTTTTGAAATCTATTATTTTTCATCATATTTGGAACAATCATTTGTGATGAATTACGCATTCTACTAGATTTG

>JAP08525.C|Supercontig_0019088:295180-295322

TGTATTTATAGCTGAACACTGTTACATAAAAATTAAAGTAATAAATATACATACAAAACAAAACTGGAGCATACACAGCAAAATGAGTTGATTATCACCCAAACGATAGCTACTTAAAGAGAAATGCAATAATTTCTGTATAT

>JAP08541.C|Supercontig_0000276:213412-213612

GATAATGGATAAACATGCATTAGGATTTGATGTATTTCTTGGACGGCATAGTTTTACAGAAGCAGAAATTGCCAATCGTTGTCAACAAGAAGTAGTACTCTATGGAAGAGATACAAAAGCTGAACGATTTCAAAAAATGAAAGGTTCATTATTCATTGAATTTTACAGTGTTGGTCAAGAAGACTTTATGAATATATAATA

>JAP08591.C|Supercontig_0000107:485461-485643

TGTAAATGTCCTAATATTTGTTATAGTATTCTATGGTTTTTTATTTTAATATTTATTGCATGGCCATTATCATTCTTTATATCAATCATTTACTTAATATTAATTGTGTTCAGTGTTTGTTGCCCTGTTTTACAACCATTAACAGATGGTATAAGTAAAGTGATGATGTTTCCGGTAATGTGT

>JAP08621.C|Supercontig_0000342:191951-192139

AAAGTATGAAGTGGCTAGTCGACATCTGTGCAATGATTTGTGGAGCGTCTAATATTAGATCACTACTCACTGAAATTGAATGTCTAGAGCGCTTGCTTAAGTGGCACGCAAAAGCTTCATGTTCACCCTCAATTTCAGGAAATGATCCACGTCAAGTTGACTATGCACTTTTGTCATTTTTTGATTTGA

>JAP08652.C|Supercontig_0000225:70216-70415

TTTTCTTTCGGACTCGAACATTTAGATACGTTGACGGATGTTAATATATCCTCAGGATATTGAAGGGTTTTTTCCAAGTCTTGTATATAATCAATAACATGTTGCAATAATTCCAACTGGTTAACTTTCTTATTTCGTTCTATAGTTGGCACCATTCTCTTCAGTTTGACCAAACATCGTTTCATTTCAGTTGGTGGTGT

>JAP08785.C|Supercontig_0000493:126838-126905

TGATTGACTAAGCTTCCACGTAAATGTATCGGTACATGATCACTTCGCGTTGAACCAGACGGTGCAAG

>JAP08796.C|Supercontig_0000043:595674-595884

TTTCAATGAGATCTTGATGCTTGAACACAACCGCATAATTTTTGAAACCACATTTTTTGTCGTCTTTTATTTCTACATTGTGTAGAACTATCATAACTATGATAATCACGTCTACGTCTTATTGGTGCATATTCATGTTTTTCATGTTCTATTGTTATTTTACTTGGAATTAATTCATTAGTTGATTGATTTTGATCAATTTATGATTATC

>JAP08828.C|Supercontig_0000304:599331-599398

ATTGAAACTGTACAATTATTCACAGCCTTGATGTACTTGGGACCATATTTGTATGATGTATACATACA

>JAP08838.C|Supercontig_0000108:464002-464260

TGCACCGGGTAAATGAATTGAAACCGTAGGTGGTATAGATTGATTTTGATCTAATGATTTTGAATGTTTCGATAATGGTCCATTGGATTGCATTGATGGAGAATCAGGACGTTTTCTATTTGATAAACCAGTTGTTGGTCGTTCAGAATTCTGTGTACTGACAGATTCTTCTCCACCGGATAAATCATCTGGATTATCCACTTCTGGAGAAATGGATAAATACATTAGAATATCTTCACATTCTTCATCTTCATCACTG

>JAP08849.C|Supercontig_0000665:26459-26824

ATTCATGTTGACTGTGAACACATAAAACTCCAAACCGCCTTTGTATCGACTTTTTCTATCGGCACCAAATGGGATTCTGGTAGAGTAAATTCTTTACAAATAAAATCTTTACACAAAGCAGTAGAAGAAAACCCGATTAATTTTGATATAAATGAAAGACTTATTGTAGGACGGAAACTAAAAATAGTGGGGATATTTCGTAGGAAGTGGGTTTGAAAGATCAGAAAAAAAGATCATGATTGAAATAAGCAATTAACATTAGCAAGTGTAGTGGAAGAATCCATATCGGAAACAAACGAATACGTTGAGGGCATCAAGCGAAATTCACGTAAACGATATTTGCAGATAGTAGTAAATAGAAAAAAA

>JAP08860.C|Supercontig_0000076:402562-402720

GTTTACCATGTTTTTTCTTTCTGTGTGAACGGCGCGGTTGAATATCAACAAAAAAGTCATCAACGGGAGTATTTTCTATTAATTTGGATCTTTCGATTAAACCAGCCATCATTGTAGGTAGAAGAGAATTCAATGATCTATCTAGAAGTCGTTGAGGAA

>JAP08940.C|Supercontig_0000045:499677-499953

TTGTGTTTGAGTTACAACGTTTCATCGATGCTATGGCCACCTACCATGATCAGTGTTATGGTGTCATGAAGCAAATTAAAATTTTTCCACTTGAAGTGGATCTGGCTAGAGATGCATTTGCGTACAACCTAGGGATTTTTAATACAGGCGAAGATGAAGGCGGTGAAGATATCGATGAGGATGTAATCAGTGCTGAGTCAGTTGGAGAAAAATCTGTCAGTAGCAAGTATAACCAGTTAACTGACGATGTCAAATTAATAGACTTGGCAGGAATAAA

>JAP08959.C|Supercontig_0000096:2176533-2176700

TTTTCCTCAATAAATCCATCACACCCATTATGGCAGCATTACATTTATCTTGTTCTCGATCTTGTCTATGAAATCCAATTAGCATAACAAACTGTAATCCTTCTTCAGATCTTAAATATTCTTCATTTGGTATTTGTAGTCGCGTTGCTGTGAAAGTACTCAAATTGA

>JAP08987.C|Supercontig_0000136:11106-11275

TAAACCGCGTAAACCCATACCTGAAGAACATGACAGTTTCCTAGATGATTTAATTGATGTTAATGACGACACAACCACAGAAATTAATGTTTCGGAATTATTAGCAGCAGGTGAACGTGACTTAGATACTCTTATGTTGGGAGGACGTGTTCCACTGAAAAAGAGCTCAT

>JAP08990.C|Supercontig_0000241:405854-405933

ATGTTAGACGGAGAGCTTGTGCCTTCAGGTACATTTCAAGCAGAAGTTATTCCAGGGATTTTAAATGTTATTTCAGGTAC

>JAP09001.C|Supercontig_0000312:1402860-1403071

AAATCACACAGAAATGGAATCCGTTATGTCGTAGTTTGCCTTATGCTACATTAACTCATCATCCTCTCTATTCTAAACGTGATCAATTGCCATCAAAACAAGTAAATGTACCAGGTGCCGGTTATGAAGATTTATCTAATCCAGTATCTGTATACGATGTGAATAATTTTCTTACAGCACTACGTGAAGCTTGTTCTGGTATGAATATTCAG

>JAP09107.C|Supercontig_0000312:461064-461278

GTGGATATCAAAACTCCAGAGCATCCAGGAACTGCTTTTCGAGTATTGTTATATCCATTAGCTGGTTCAATTCTTGTGGATTGTCGTCGTGTCTCAGGTGATGGTTTATTGTTTCATCAAACCTATGCTGCAATCTATACAGATCTTTCAGCTATGGGTGCAGTAAATATAACACATCCTTACTTACATATTCTGTTACCTGAAAGGCCTACAAC

>JAP09132.C|Supercontig_0000003:2009430-2009517

GTTAATCCACGTTTATTGGCTTGGCAGGCACGTTCATTCTACAGACATTTTCCAAATTGGCTTCCTTCACAAACAGCAAATGATCAGG

>JAP09156.C|Supercontig_0000215:129374-129539

TTGGTGGGCATCATTTTCCCACTCATTCGTTGCATCGTTCTCATCCTTCTATTGCTAATCCTATACCATCAAATGTTAGACCACGAAATCCAATCCAGAATAGTCAATCAGGACTTACTGTAGCGGTATCACTCATGGGAGGTGGGCACGTATGTGTAAATGGAAA

>JAP09189.C|Supercontig_0000065:479198-479409

TTCAACTTCACAAGGATCATGAATGACAGGTTTTTTATTCTGATGGTCACAACATTTACGCTTGATAACCAAGCCCCAATAAATTAAACACAAAGGAATCGGTGTAAGCATAGCTACAGTAATTGGCATAATGGCACCTAATTCACCAGTTGGTTTTGGCATTGTATACAATAAAACAAGTATTCCAACACCAATATTTTGTATACCAGTCT

>JAP09213.C|Supercontig_0000417:164088-164198

CAGTGGTCAAACTTTGTTCATGATTTTGACAAGAAGCAATGCAATTTCCGGTTGGCGGCAACTTATGGGACCAACAGATCCAAACAAAGCATCTGACGAATCATCAGAAAG

>JAP09326.C|Supercontig_0000311:338013-338119

TAGCCGACGTGATAAATCGATTGATTCATTATCAGAATTACGTTTAATGCCAGAAATTAGCATTATAGGAAAATCTTCAGATGTAAATCGATAATTATTATTATTAT

>JAP09329.C|Supercontig_0001522:41484-41655

AATAACACTTTGTTGAACCCATTTAGGCAATTGTGATTGAATCGTTGAACGATAATACCGATTATCCATCAAGAAGACAGCAGCATAATCTTTGGCATGTCGTATAGATCGACCAATAGCTTGATTTATTAAACGCATACACATTGTCTCATAATATTGCCTACCTGGATTT

>JAP09394.C|Supercontig_0000144:186639-186842

TGATTCTGATTCTAATTCAATGCCAAAATCATTTTCTTCATGTTTTGACTTGGATAATGCTTCTTTAAGTTTTTCATGTTTAATTTGATTGGCTTTTAGACGTTCTTCAATATCCCAAGGGGAATCAAGTGGGAGATTTAATTTTTGTAAGTGATTACGCCATTCAGTTTGACGCTGATTACACCATGAATCAGCACAATCTGG

>JAP09484.C|Supercontig_0000173:456590-456748

TTTAGTCTCTGGAACGAATGGCTGCACGTTTAAGTCGTTTCTCCGTACTTAATCGTCAAATATTTTCTACATTAAATGTATATCTTCATCCTGTCGATAGACTAGATGAATCAAGTGTTCGTGTTCGCCAGTTTCCTATACCTACCTGGTCAAAATCAT

>JAP09488.C|Supercontig_0000067:796110-796234

AATTGTTCCATTTGATGTATTTGTCTCCAATCAACTGGTTTATTTGAATTCATAATAAAACCAGGTAAACCAATTAAATCAAGCAGATTACGCCATATACCATGATCATATGGACGATTATATTT

>JAP09492.C|Supercontig_0000073:266535-266859

TGCAACACACAAATTTATGCAATGAAAATTAAAAAACAATGCAAAGTAATGTAGATGATACACATTATAAGTAAATGGTAATAAAAAAAGACTACAAAAAACCAACAAGCAAAAACATACATAATTGTTTCCATTATTGAAAACGAAATACAATAGAGTAAATAAATCCAAAATCTTGTACTCTATCAGATTAGAAAGTAATGCTGCTTGCCAGAAATAATAAAAACACACAATGACTATAAAAAGAAAGGCACAAGTGCGAAATAATTGTGTAGTACGATAAAGATAAGTAACATAAGATAAAAACAAAACAAATGCGACTGTG

>JAP09502.C|Supercontig_0000277:40100-40437

CGTTTGTTTATATTCTGTTAAGTAAAATGATTCACATAAAAATTGATTTAGTCTCAGATGAATAAAATAATTGAAATTGAACAATACATTTTAAAGTTATTTGCTCATCAAGCATTAAAGATTATTCATTTAGTTTCTACTACCCATAATGGTTCTGGACCACGTTTAGCGTACGCACCAGCAAATGCACCATCCTCACACTGACTTAATATATTCTCCATAGTTTCTATAGAGTAGCGGATTCTACTGATTTGAGATGTGGAACAAATTTCATTTGACTTAGTTTTAATTGCTTCGATTGTCTGCCAAGTGAAAACATACAAAGTGATTGTTATCCA

>JAP09503.C|Supercontig_0000089:640595-640773

TAATCATAGGTACTAATAATGGAATAGGATGTTGTTATACTACATCTAATCGTCGTATTCAAAATGGAAATATTCATTGTGAACTTGATGTTGAATCAGCTGATTCTGGACGTGGAGCTAGTGAAGATGATCCAAGTCAATTAGGTGGACAATTTATGCATTTTTATCCTACATGTCAT

>JAP09509.C|Supercontig_0000120:243402-243562

CAATATATTTATCATTTGAAATGGTTTCTTCAATCAACTCGATTTAGTTTAACTGGAGTTTGTATGGCATCTATCAATGTGTTTGCTTTTTACTCACTACGATGGTCTTCTGTAGAATTACGCCCACTGATCATCGCCTATTTTGTTTTGACTATGGTTAC

>JAP09512.C|Supercontig_0000073:681471-681722

CCTCGTTCTCGTGTACGAATAGTGCTTAACTTTCGACCGTATACACTAGGAAAATCAAGTTCCGTTCTGGGAATTAGTGTTGAATATCACAAACAAGAAAATTTATCTGTGGAAAAACCCAATTTTAATGTCAAACCAACTATCGATTCTTTTCAAGTTCACTTATTCGGTACAATGAATTAACTTCAAATCTTTTTTATTCCTTGTCTTTTACAACTGTATGTTTTTACTGGTGATTTTAATATATATTTA

>JAP09517.C|Supercontig_0000262:6417-6537

TATATTTAAAACACTAAATAATATTCTTGGATGGATAACTATAACATGGCCATGGAATCCTACAATTGTCAATTTCCCGAAATCTATTAGGGAACCAAAAATAACTTTAACACAGAATTCA

>JAP09531.C|Supercontig_0000061:138292-138386

ACTGTAGGAAGCGTTTTATGTGCAATGGTAACGACATTTGTGAGCTATCAAAATGCATATGTAGTTCAGTTTACTGGCTGTGGATTCGTATGCTT

>JAP09670.C|Supercontig_0000107:485461-485643

TGTAAATGTCCTAATATTTGTTATAGTATTCTATGGTTTTTTATTTTAATATTTATTGCATGGCCATTATCATTCTTTATATCAATCATTTACTTAATATTAATTGTGTTCAGTGTTTGTTGCCCTGTTTTACAACCATTAACAGATGGTATAAGTAAAGTGATGATGTTTCCGGTAATGTGT

>JAP09716.C|Supercontig_0000480:489925-490040

ATGTTGACAAAATATCTCGATTTTCACGTATACGTAAAGATAGAAATAATTGTTGGGAAGCGATTAAAGCCCATGATACAATGGTAACAGTAGCTGTGTTCAGTCCAAATCCAAAT

>JAP09717.C|Supercontig_0000050:1321366-1321542

TAGACACGTTTCCACAATTTTTTAATTTCACGATCATAATCAACCCATTCCGGAGCGATACGCATTGCTGCAGTCAACTTCGGTTCTTTTGTACGTGGATTATTACACAGATCGATAGTTTTCGCTTTAGCTAAACTTTCATCAGAACACCATGTTTCACACCATAACCATTCTTGA

>JAP09718.C|Supercontig_0000050:913933-914123

TACTAATCATAGAATCAATGGATTTATTGGGAACATTTTCAATTGATTGGAAAACTCCATTAGAAAATTTATCTGAAGCGCCAGTTATCAACAAAAGTGGGATTTTGATTAAATTAAAAGAAAAACAGAAAAAATTGTTCACATCAGGTCATCAAATTAAACAATTTGACTGTGATCTTAATTTAGCCAAA

>JAP09738.C|Supercontig_0000191:331979-332085

ATAAACCATATTCTGCAAGCATATTACGTTTGGCCAAATCAGACGGTTTTGTGCGGTTCATAGTTTGTTTCTTCTGGGGTTTATCAAATGATAAGGCAGATGAATAC

>JAP09743.C|Supercontig_0000075:142171-142306

TATCCCATGAAAATCCAGGTTTATCATATGGTAATGATGGACAACAGGTTATAGGCCATGTACCATGTTCATAACGATATTTTAATTCTGTATTACTAATAAACTTTAATATTGTTAATAATACCATTAATATTGT

>JAP09750.C|Supercontig_0000638:101821-102067

CTGAAATCATTTGTCCAAATAGAATTTTTCACATACGCACCAACATGACCAGATTGTGGCAAAAAACGAATTTCACCATTAGGATATACTATTTTTAGTGAACAAACACTACCTCTTGGAACATATGCATCATATTCGGCTGCAACAGATAGAACAAGTCGAGAATCTATTACTGGAGAAAAATTTCCTAAATGAGTAAAATAATCTAGAAGATCACGAAGAAATTGTCGAACTTCCGGATCTGGTG

>JAP09765.C|Supercontig_0000074:1113619-1113802

GATTCTTTTAGCTGAACGTCAATTAAATTCTTCAATGTATTCCCTCATAACAACTACAAAAAGTGCTAAATTACAAAATAATCGAGGTCCATTACTAGAAGAATATCGACGACATTTAATGAGTTTAGCTTATGCAATTGCAGCTGATGCAAACTCTTTATATACAACTGTTTATGAAAATCGT

>JAP09866.C|Supercontig_0000263:312239-312764

CGCAAAAATAACTAAACAGGAAGACTTTTAGAAATTCCTTTTACTGTCCACAAACTACCACGTAATAGAGTTTTCAAGCCAATTCTACCTCCAGGTACAGTGGCAATAATTTGCCATACAACTAAACAAATTTGGGAGATCAAAGCCAAAGCTGAAAATATAGCACTCTGCCAAACAACCACAGCATATAAACCACCAAATAAGCTGACTAAATAACTTAGAGTGAAAAACAGTCGCTCAGTGCTAAATAAGCTTTTAATGTGATTGCACGGGCCCAAGAGAAATGAAAAACTTCCAATTAATAAAATACTACCAAGTGTGTGCAACAGTACGTATTTACGCATACCAAAAGGTGTTGCAATAACTGGCAAAAAAACCATCGCCAAACAGAGACATAAAGATGCTGAAAGAAGACACAAGAAAAACCCCATAAGACGCTGCTGTCGAGACATGCCTTTAGGCATTAAAGGATCAGCATCAGCTTGATAAAACCAAGACGTAAGACGACCTTGTGTCTCGATTTCAG

>JAP09888.C|Supercontig_0000357:77836-78025

AACTATGTACACCAGGCTGTTATTATGAACGAAACTATTAAAAAAGAGTTGAAACATCAAAAAATATTCACAGAATACAGTATCAATCCTTTTAAGAAAAGTAAGATTTCACGTATTTTATAATTTGCCAGTGTATCCCTTAGCTGATAAACCAAATAGAATACAAGAAGCTGATGCTGAAGACAGTAAG

>JAP09889.C|Supercontig_0000094:1004389-1004612

AGGTCGAATCTCATTAAATGAATTTAAACAAGCCTGTTTACGTTTACCCAAATGGACAAAAATAGATGAACAAATAGTAATGGATATGGCACGTTCAATCGATATTAACAAAGATGGTTTAATTGATTTCAATGAATTTCTAGAAACATTTAGACTAGTTGAATCCGATCATGATTATTATGAATCGTTAACTGAACAACAAACAGATGAGTGAAATCATTGAA

>JAP09892.C|Supercontig_0000231:633101-633374

AAATATACTTTAGCCATAATCATTTCCAATTCAAATACCATTAATAGTATACCCATAACAGCAAAAAATAATGAATAATCAGCTAATTTTCTACGACGTTCATTAAATATTTTACGCCAATGTAAACGCCAACCAATTCCACGACTTGACGATCTATAATTCATTTGTGGTTGAACTGAACCAAGTGGACCACTAGTACCATATAATGAACGTAAATCTTCTGCATTACGACGTATTTTTGCTCGTATACCAATTGGTCGATTAGTTTCATCAT

>JAP09900.C|Supercontig_0000180:274684-274889

TGTTTAGTCAAAAACAGGAATCTTCGTAGATTTTGCTTCTGCAGTTCTGATGATGGACGTGTTTTTACGAAATGGTGAATGGAATTCTGCTGTTGCTGTTTCATGGGAACTCTGCCTTCAGGAATATTTCAGCTTAGAGAATGTCAGACCCCTTGTCTTTGCTGCTTCACTTTTTTCATGCATAAAGTCAATAGAACACGATTCGT

>JAP10047.C|Supercontig_0000096:1341363-1341466

TTCGGCTTTCACTTCATCGTTCGGTAGTAATGAAATATCTTGATCACCGACCATTTGGATAAGTAATTGCAGAGTGTCAGTGATTAGTTCATTCGGAGATAAAT

>JAP10164.C|Supercontig_0000044:927818-927935

AATAACTTATTTGCAAGTGCTGAAGAAGTTGGTCATTTATATGATGTACAAGAAACTGCAAAAGAAAAACGACAACGATTTTGGGAACAGAAACGTTTACATTCACAGAGTAGTAATA

>JAP10174.C|Supercontig_0000066:372281-372460

ATGGAGGAATTGGAAAGTTGGAAAAGAACTCATGAAACTCCAACTGAATGGCGCGTAAGGCGATCGTTTTTAGAAAAAAATTTTAATAAATTACACCCTGAACGTTTGGAGTGTCTTTCACATTGTTTTACTAATGCTACATTGTACAAAGTCAAATATCCAGCAAAAGTCATGGAAGAG

>JAP10262.C|Supercontig_0001915:32228-32473

AGGACGACCGGTTGGATCAATAAACAAGTGTCTACACAATGGACAAGCAGGGTTTCGACTAGTTGTAAACCACTTGTACATACAAGCATAATGAAATAGTTTACGACACGTGTGACATCGCATTTTAGGCAAACTGAAATTTGTGTTGTGAACAATCGAGTAACAAATTGCACATTCTTCGACACCATCGAATTTTTTCCGAACATTACTTCGCCACAAATCGATACCGTCTAAAATTGATCCATT

>JAP10325.C|Supercontig_0000367:167435-167589

TTTTATATAGTTTATGATATGTTACAATTATTAATACATACTATAACAGCACGTTCTGGTATTGAAGGTAAACATTATCAAACCATAGCTAAAAAAATACGTCGTGAATTAATGGAACGTCCACCATGTTCTGGAATTGAATATCTTCGTCCATT

>JAP10374.C|Supercontig_0000107:782895-782965

AGGGTATCCATACAAAAGCACGAATTCAATCTCAACAAATGGAATTAGAAGCATTAGAATCACGTCTTGCT

>JAP10386.C|Supercontig_0000481:449170-449319

GGTTTTGAGCGTTGGGTACCAAGATCATTCTTTTCAACCGTCCAGTCGGGTTTAACTGGATTATTTTCAACTTTTGATCAGGCTGTTCCTTTAGCTTCTGTTTTGCCGTCAGAAGCTCAATGTCTTAGTCCATATCAGTTAAATGATTCT

>JAP10397.C|Supercontig_0000015:471902-472055

ATTAAGATTGTAGACGAAAGCGAGCAAAATCTTCAAATAATAAGTCATCTTCACTTAAGGTAGACTGTGTACCAACAGTAGATGGTCGTCGAGCTGATGGTCGCAAACCGGAGAAAAGCAAATGAAAATTACGATCCCATACAGGTGGTGAATA

>JAP10403.C|Supercontig_0000440:64569-64880

ATACCTCGAAAATCGATAGGAATAGTCCCAGTACGTTCCATTTCTAAAATATGCTGATGAATTTCACGTAAACGTTTCTCATAATCCATATTTGTCCATACAGATACCAAAAGAACAGCTCTTCCAGCTATATGAGTGACCACTGTCATTAAATCACCTTTTGAAGATAATCGGAATTGATATCGTCGACCATCAATCAAAATTTCTTCGAAATGATAAATATTATAATGATCTAGATTGATATACTGGAACTTGTCTGAAAAGACTTCTTGGAGAGTATCAGCTTTAGCTTCAGGTAACTCAACTGACATG

>JAP10460.C|Supercontig_0000304:851488-851597

AGGTTTTTGCATTCGATCCAATAGATAATGCTAGATATATGCGTCCATTGTTGTATAATCCTGGTATACCTGTTCCTTCTTCAGATCCAGATGCTCCACAATTTGTTTGA

>JAP10492.C|Supercontig_0000646:214556-214710

ACTGGCGGTACGGTTATGAACTGATGACTGAAGTAAATAAATATGTCAACCAAGTGCCAATGAACAAATCGAAATGCAGGGAAAACACAAGTGAAGTTAACTGTTCGATCGGAGGACAGATAACCGCCAGCCATATCATGACAGTATATAACTTT

>JAP10598.C|Supercontig_0000050:1523562-1523742

AATTTAGGTAAATCTTTTTTTAATATTCTAGTCCATAATTTTGTTAAATTATTTTTATTTGATTTACGTATTAATGAAAAATATTGAATATTATCATTATGACGTGAACGTATATGTAATTTTAATGATGATCCTTGAGTGAATGTTACACCACATGCAGAACATGAATAAGGTTTATCAC

>JAP10726.C|Supercontig_0000065:355882-356062

AAATAGAGTAATTCGTCATTACAAAGATTATGGTATTATTTATTTATGTGACGAACGTTTTGCATCAAATAATGCGCAATTGAACTTAGCTGGTTGGATGCAAACAAAATGCAAAGTATACAATAATGTGGAATTAGTTGTAAAAGATACCGATGAATTCTTTCGTAATATGCGCAATAAA

>JAP10727.C|Supercontig_0000269:486172-486329

GAATATTTCAAATATACCATCAATTTTTTGATTTTTATTGATCAAACCAATCAGTGGAATTATAGCTAAGCCAAGACAACTTGTTTCAGGTGAAGGATCTGTACTATCAACAATGTAAATGTTTAAATTCTGTGTTTTTAAATAACTGAAAAATAAAT

>JAP10850.C|Supercontig_0000132:742954-743144

GGTTATATAATTGATCTAACTCAATCACATGATCCAGCCTTCATTATTTCTGGTTTGGGTATGGTTTTGGCAGCATTTTCAGTTGTTCCTATTATTATACAGCATTTTCGAAGAAGGCGTGCGCGTCGATTACGTCGTTTACAACAAAATTGTTCTGAAATTGTATTAGAAGCTCAAACGTAACAACGATA

>JAP10903.C|Supercontig_0000194:70539-70604

ATCATCTGTTTGATCTAATGACAATAAATCGAAATTGTTCAACGCGGCAGCAGAATATCTAACAAC

>JAP10907.C|Supercontig_0000044:1791125-1791429

AAGATCGTTCAAAACAACGAAGACAAAATGCCCAAAGAAAATTAACTATCTTATTAATAGCTGTAATTTTATTATTTTTAATTGGTCAAGTACCTCAATCATTAGCTTATGTTCGAGTATTCACTACACTTGGTGTATGCTCCATTGATCAAATACAAACATGTGTAACTTATCATTTATATAGAATGATAACAATTAATTTAGCTCAATTTGCATTTGCATCAACATTTTTTCTTTATTTATTTCTTAATAGAGATTTCCGGGAAACACTTAGGTTTATTTGTTGTCGTCTGTGTCAAAAATGT

>JAP10930.C|Supercontig_0000234:384494-384657

GGATCAAGTGTTGTTAATAATTGATAAACTTGTTCATCTGTTAAATGTACACCAGTACCATATTGATATAATTCTCTAAATATTGGAGCATCTAAACGACCACAATTTGTTTTATCAGCTTTTTGCATTGATCGAAGCATATTATTCCAATTTAATTGAGAAAT

>JAP10931.C|Supercontig_0000043:1495430-1496101

AATTCACATTCCTTAGAATTCGCAACTTTTACCAAATCAATATATTTTGGTGTGAGACGATGAATTAATGGACCGATTTGTTCATTAACTTGACTAAGGAATCGATTATGTACAATAGTTTGATACCACATTGGATATATAGACCATTTACCGTTTGTTCTTTGAAACAATGCCCAATCACTTAAGTTCAAATAAATGAAAATTAATATATCATTCCATGAATGCTTGGTATGGTTTGATTGATAATGAACTAATGCACGTGTCATGTAAAATTCTGGTTGAAATGGTGCTAAATAAATACAGCGATCTAAATAATTTATTGCATTTGACCAATCACTTTGTTTAATTCTATTTTTACTTAACTGGTAACAAGTTAAACAGATTCTTCCATTGATTGAATTAATACTTTCGGGAATTTTCATCATATTGTTATTACAAATGCAATGGTTAGAATCGGTCAATTGTTTACTAATTATTGACTTTGCGTTTTCATATTCTTCAAGTGAGTTTTCATAATTCTTCATGAAATATTGACAATCACCAGATAAAATCAATAATCTATAATTTTTAGGATCCACTTCCAAGAGTTGACGAATCAATTCATTTGATTCTTGATATTTCTCTTGTTTTAATAGCTTTATTGCATAAGAGTTTATTGTAAGAAATAACTGA

>JAP10935.C|Supercontig_0000292:556666-557115

AATCAGGTTTGGTTATATGGTCCAGCTGCCAAAAGCAAATAAGGATGAACATAGTGCAGTCCAAGGACTGAGGTACGTCTAGTGAAAAACACTTCGCTAGCACATGCATCAGAAACGTCAGGACCAGTCGCTGAAGAATAATATCCTAAAACATTATGACCTCCAACTCCACTGCTCTTATTATTTTTTGAGTTTTGACGGACAAACTGCTGTTTGGTGAATCGATGCAATGCCAAAAGATTAGTTTTGGAGTTTTTATCATCGTTATTACCAGTAGCTGGAGTTGTAACAGCAGTTGACCAACCACTAACTGTATTCCATATTCGTAGTGCTCCCTCTAGACCACCTGAGGCCAAACGTCCTGAATTACCCGGACAAAATGCGAGAGATACAACTGGACCAGTTAACCACTGACTTGAGTCTGAATTAGCTTCCTGAGAGTCAAAATCA

>JAP10999.C|Supercontig_0000155:962573-962747

ATTTCATTAGTATGCATATTATAAACGATTATGTCAAGCTGCACTTTTAATCCAAAATCAATATCGTTGGTACGTAAATCAAAAGAAGAATGGTAATAGCGGGGGAATTTCAGCTGGTCCTAAACTTAGAAAACCTAGGTCAAGTCAATGTAACAATCCACGATACAGGTTTGTT

>JAP11010.C|Supercontig_0000195:266857-267026

ACCTTTCTGAACGACTTAAAAATTCACGTTTAGTGAACTCATCCCCGTAGTCCACAATTTTTAACGAGCGAGAAAGTATAGTGACCGTGCTGCCTAGATAAAAGTCAGATAATTTCAGTCCTTCAATCCTAGTAGGTTTAAGAAAAAGCCTATTAGTTTTCTCCTCATGC

>JAP11019.C|Supercontig_0000073:891419-891703

GGGAGCCTTTAGTTCAACGTTTTTATCTTGAACGTTCTCTGTTTGCTTGTCAAGAGTGTCGAAAAGGCTTTTACTATTATCAGCATGGTTTTCTGAATACATCATTTAATAAATCCTCTAATGCCAATGAACAGATATCTAATGCAGCTGAACATTCTCGTTTACGAAAAGATTCATTATCATCTGATTTCCCGGATTGTCGTGAAGTGATCTTGTTCTCTCGCCTGAATCGTATGATGAATGCAACTAGTAATGCGCTTAGACAACAAATAATGAATGATGAAG

>JAP11088.C|Supercontig_0000187:151863-151966

TAAACATAAAACTATACTTACATAATCTGTGTAAGCGTCATGGAGTTTTGCACAAATTCGCTTCAATAAACCATGAGCATTGAGAGTATAACGGAAGGATGCAG

>JAP11113.C|Supercontig_0000292:681530-681714

ATTCTGATACGTTTAAACCAGAAGTGATCAATAAGAACACAAGGGCGTTGACTCAATCTAATAATTCATTGGCTGAAAATTATGGACCAGTCACACCCGTTGTACAATATACCGATGATAATGATAAAAATATTCTCGTTGGTAATCAACAAAACCACCAATTTTCAATCAATCAGTATAGAAAA

>JAP11142.C|Supercontig_0000490:313346-313513

CCTAATCCAGACGTTCGCATAACCGGTGGTCTTATTTTATCCCCAATATTGCCATACCTCATTACATGGCCGCTAGCTCTTCTTCCTCCTAGCTTGGATAAAACTTTAGAGAATCCATCAAAGGAGGCGAGGATACTTGCTGTTCGTAAACTACTTCGCCTGTGAGTT

>JAP11313.C|Supercontig_0000130:689776-689882

AATTTCACATATTCTGGACAGATAAGAGATGCTGGTGTTGGTTTTTGCCAACGTTTCAAAAGTAATGAAAGAAGTATGACAGAAAGAGATGAAGCAGCCATTGCAGC

>JAP11352.C|Supercontig_0000304:39329-39509

GGTTTGTCGTTCAGTCTACATTAAATTGCGCATTATTTGGGATTCTTTGATATTGAAAGTTCCAGCTTATTGGCATCGAGATAACCTTGAAGCTGAAAAGGTATATGAGGTTCGAACCAAATTACAACCACTTTTACGCTACGCAATGTTATGTCCAGAGGCTTCTTCGATAAAGGCTAGT

>JAP11444.C|Supercontig_0019087:136099-136194

ATGAGCAGAACACGTAAAGCAGGATATATTATAGATGGTATACCAAATAGTTTGGCTCAAGCTAAAGAAATTGAAGCATACAATGATTCATTGAAA

>JAP11477.C|Supercontig_0000319:6536-6745

CTGAAAGTCCAAACAATCAAGCAACCTTACCAACTAAGAACAATATCCCACGATGCAATGTACCTCAGTGTCCTCCTGCAGATTTGCTTTCTGCTATACGCGCTGGCTGTCAGTTACGCAAAGTAGGTGAACGAAATCTTCCCGACCAGTCGAAATTTAGCCGTGCAAATAGTTTATCGACTCTGGTAAACAAACCTAAGGATGTTCAAG

>JAP11511.C|Supercontig_0000003:1548325-1548509

TATGAAGATCCAACCGATGATTTCTTAGAAATGTTTATTCAATTCGGTTATGTAAGTATGTTTACTGGCATATTTCCATTAGCCGGTTTATTGGCTTTTATGAATAATATAATTGAAATACGAGGTGATGCATATAAATTATCAACTAGTTATCAACGTCCATTTGGTAAATTTGCAAATAGTAT

>JAP11520.C|Supercontig_0000486:454306-454555

GTCGACTTCGTGCTCTTTCAACAACTAGCTCACCTATGGTACAAGATGCTCGTGTTTATGACATAATACACTGGTCATTAAGTCCTGAATGTCGTTGGCTTTTAGCCACTAATATTGGAGTACCAGCATTTGATCGTCTTTTAGAAAGTATTGGCTTCCGCAAGGCACGTGCTACCATTCCAAAATGGCTTCAAAGAGGTGTAATGGATCATTTGGACAAAATTGCTTCTATTCTTTTAAGAGGCAGCTT

>JAP11574.C|Supercontig_0019079:10733-11000

CTCAGAAATTATAAAAAACTAAAAAACACATACAAACTATTTTGTGTGGGCTGCGTTGTTTTTCTTACTGGTTTTTTCTTCATATTTTTTGGCCGTCCATACACGTTTGATCGGACAAACAAGGATTTATTCTTTGTTTTTGGACTAGTTTTTATTTTTCCCGGATCATTCATTACATTGATGTCAGCGTATTTTCTTTTCCATTTGTGGAAGATCTCAAATGATTTCATAAAACTTAATAAAACTCCTGTTTATCCTGAAGATACAG

>JAP11591.C|Supercontig_0000005:1677292-1677560

TTCATTTTTCCATTTATTACAAGATAGTCAAATAATAGTGTAAATATCAATCGTATATTTCCTGATGGTAGATCAATAATATCAACTGGTCCTGCGAATTTTATAGCAGATAAACCATTAATTCTTATATCGAAAAATTTAGCAGTACCAAATTTGATTGGATTACCAATTTTATATACACTATCTTTACCATAATGAGTTAATAATTGTTCTATATGATATTGTAATACATTACTTGTAAGTAATGAATTACAAGTTAATGGTTGATT

>JAP11611.C|Supercontig_0014211:1179-1281

TTTGGCGACGGTAATTCACTTCTTCTAGTGGAGATACCAGGAATGATAATATTTTGTATAAACTACTTCAGGTTACTTTGTCGTCATCATTGTAACAAATTCT

>JAP11620.C|Supercontig_0019089:86105-86357

AGTCACGAAAATCTTCTGGTCTTGCCTATATATCACGTTATTTAATTCAACGTCCAAATGAATCCATTCGATGGATGTTTTTATTTTCTTGGTTATTACATAAAAATCCAATTAGTCAAACTACGAATTTGTCTACAATTGAATTAAAACAAAGTATGAATACTTCAATGGAGACTACATTAACAATCTATACAAATTTAATTATTAAATGTTTATTATGTATCAATCAGTTACGTAATGAATGTCCTATGAA

>JAP11632.C|Supercontig_0000447:253772-254049

TTATTTTGCTTTACTTGTAATCTTTCTAATTGTCTCAGCATCAGTTCTTATTGAATCTAATTCATTCAAATCCATATTAAATGATTTTTTCAATGCATCACGGAGGCAATTTTGATAAATTTTCAATTTTGATTCACATGGATCTGTTTCATAGATATGACCTTTTAGAAATTTCGGGAAAAATTCTTGAAAGCATAAATCATAGGCAGTTTTTACTGTATCACATTCTGGTACAATACTGCCTAATCTAGAATGAATTAATTGTTCTTGATTTTCTG

>JAP00177.S|Supercontig_0000023:537875-538032

ACTAAATACTTCATTTCTAGAAGTATAGTCCACATTGCGATCGAAATCTGGATTAGTCAACCAAGCTTTTAATCGATCTAAAGAAACACCATGTGACAAGACTACTTCATAAAGAATTACAGCAATTAAGGAACCACATAATGGACCGACAAGATAAA

>JAP00193.S|Supercontig_0000002:557316-557424

TCCCAACTTTGATGAGCGACAACAGTATCGTATTCATTCCAATGGTAGTGACCTTGACTTATACGTTGGATAAGTGATGATCCACCAGCATCATAAGCGCCAGCAGTTA

>JAP00197.S|Supercontig_0000007:2153190-2153320

GCTTATTTATTAAATGACGCCATTCTGTTAGTTGTATCGGATAAAGATGTTCTTGAAACTCGTCGACCATCATGTTTAGCCTACAAACCAATAACTTTCAATTTAATTAGCTTTCAAGATTATGACATATC

>JAP00226.S|Supercontig_0000195:308929-309036

ACGTATTGCTTTAGAACATTCTTCATATTGACTACCACATTCTTGAACACATAGATCAAATTCAGGTTTACTCAATTTTTCACCATGTGAATCAGCATAACAAGAATT

>JAP00284.S|Supercontig_0000015:943918-944060

TACATTAACAGTTACAATGTTTATCCACCTGGTATTCCATTTGGAGGTTATAAACTATCTGGATTCGGTCGAGAAAATTCAGTTGACACATTGTTGGCTTACAGTCAGTTGAAATCAATTTATGTAGAAGGTGGATCACTTCC

>JAP00312.S|Supercontig_0000142:677225-677367

TTACCTTTTCAGCAGTCAAATAGCCTGGATCAATGCAACTTGTCAAACCATAAGTAATAAACGAAATACTGAGAAAGGCGAAAAAGCATACGAAATGAATGAAATCCTGTTCAATGAAAACAACTCGATGTAATTCTGAAAGA

>JAP00342.S|Supercontig_0000381:179187-179291

TTTTGTTTCTTGTTTTCTCATTTACTAATCAATTAGCTTTTAAATGGAGTTGTCACAGCTGGTCCAGATGTTCCTTGTATAGATGAGGTTATGGAGGTAGGCTTG

>JAP00396.S|Supercontig_0000079:272463-272680

TTTCGTTTCTGTTTTCCCATGCCAACTTCTCGTAAAAGACGCCGACGTTCAAGCGGCGTTACTCTTTGGCTAATTGAATTGTTTGATTTACCAATGCCAGCTCCAAGTGGAACAAATGCAAAAGGTTCTGGCATACCAGCTCGTTTCATATCACCTCTTGCTTTGCTTGATGCGTAGATACGACCAGGTATTGGGATAGTTTTCTTTTTTCTATGTTT

>JAP00414.S|Supercontig_0000214:481827-481971

ATCATCATCACCATCATTCAACACATAATAATTTAAGATTTCGTGGAGGAAGAACAAGTAATGGAATTTCGCCTAGTCGTAATCGACTTCTAGATACATCACAAGAAACACAAGATTGTCATGTATCGTCTACAACTACTAATCT

>JAP00451.S|Supercontig_0019086:91513-91725

GATGATGATGGTGGTATATGAAGATGTAAACTTAAAGCATGTAAATGTAAACCAATATAACGAACTTTAGATTGTCTAATATTTAAAACTTCTAATAAAGATTTTGGTAAACGTTGTGGTGCTAAATATTCTGCATGAGAATATTTATGATCACCAAGTATAGGAGTGTGTAAACCAAATGCTAAATGAGCTCGAATTTGATGTTTTATACCT

>JAP00524.S|Supercontig_0000343:253705-253765

GTTGGATACATCGCTGCTAATAGATTCGTTCGTAAAATATACTCGATTGTCAAGGTGGATT

>JAP00533.S|Supercontig_0010771:4941-5185

GAAGGTCTTGGTTTAACTATGGATCAATTTATAGATTTGTGCATATTACTTGGTTGTGACTATGTTGATACAATACGGGGAATCGGCCCTAAGAAAGCGTTAGATTTGTTGCATAAGTATCAGTCGATTGATTGTGTTTTGAAGAATATCGATAAATCTAAATATATTGTCCCAGATGATTGGCCTTACGAAGATGCGAAAAAGCTTTTCTTGAATCCCGAAGTGACAGACCCTTCATCAATTGA

>JAP00540.S|Supercontig_0000617:93039-93178

TTTCTTGTTGTTTCAATTGAGATTTAAGTATATGATTTTCAGATTTCCATCGTTCAAATTCTTGTTGCATTGTTTCTAAATCTTCTTGAACAAAATCCATCAGTTTACTCAATGGAATAGCTGAACGGGATAGTGTTTGG

>JAP00566.S|Supercontig_0000067:475695-475820

ATATACAATTGACGTGATTGACTTTGATATACCTCTAATATAATATTCAATTGATCACATATAGCTCTAGCTCGTGACCATTGTGTTTCAAAACATAATTGATGAAATAAACTGCTATTATTATTA

>JAP00581.S|Supercontig_0000012:1128720-1128843

CTTCATCACTATTTTCAAAGCCAAGGATTTCCCCAGTTTGTGCCATACTTGCTAACAGATCACTAGACATATATTTCTCAAGGATTTCACGTTTGCGCTGGGCTACTAGTGCCGCTTCAATCTG

>JAP00606.S|Supercontig_0000076:313866-313951

TTATGCTACTATAATTTACAACAAACGAACAAAAAGCGTTGAAGTTATTCACAAAAAGCCGGAGTTTCTTGAGCCTTGTTTCGATG

>JAP00668.S|Supercontig_0000032:738488-738658

GGTCCAACTAACTGGGGTCAAGTAATCACTTTGTTCTATTTTGGTTATCGGTTAGTAGTGCAACGAGTTAAAAAAGGTGTTGCCAACGCATTCTATCAAGTATGTCGATGTCTAGTCAGTTTTTGTCGACAAATCAATATTTTCGTGTGGATAGCTCAACAAGGAGGATGG

>JAP00768.S|Supercontig_0000280:266497-266772

ATCTAATTCATGTTGTTTTGATTTAGGTAAATAATCATCTAAATTTAATTGTCTACATTCATAGATATATACATCAATATGTGGTAGATTGTTATTTGGATAGTAACCAATACCAATACATAATTCACCAAGACCAAATTTATAACTTGTACCAGCTGTTAACCAACCTGTTCTTTCTATAATATTACCAGCATATTCACCATGATCTATATCTTTAAGATAGACTTCTAATTTACCAATACTTATATCTTGACATATATTATCATGAGCGAATAC

>JAP00795.S|Supercontig_0000299:322468-322581

ATGTAGACAGTGGCGCATCGGCAAGTTCCAAATTCCTGAATTTCTCTGCATCGTAAAATCCAGCCTTTACGACACGTCCAGCAAAAAATCGGCCATTCAGATCAAGTACCGCTA

>JAP00802.S|Supercontig_0000038:680553-680669

AGGTATGTTTGCTATACGTGCAGAGCGAGATTATACAATAGAAGAGGACTTTATGAAAGCTGTACGCAAAATAGCCGATGCAAAGAAATTAGAAACAAAATTAGATTATAAACCAGT

>JAP00884.S|Supercontig_0000064:882580-882818

TCACTTGTACATGATACAGAAGAAGGTGAAACGAGACCATTTCTTATTGAATCACTTAGATTGTGTGATGACATCGAATTTGATTGATTTTGTACGGATGAACTAAGTACACTGGACGTCTGTGATATATTATGCAACTGACTGGTCGATAAAACACTTCTATTAGAAATTCTACTATCTCCAATACTATTTGTCTTTTCGGATAAAGTTTTCATGTTTGCATTTGTATTACCAATAAG

>JAP00948.S|Supercontig_0000378:184076-184262

TTTAAATTATTCCTCTTTGACTTATTCTGCTTGTTCTCAATCGTTCAGAAAATGATTGTGTAACAAACAGGTAAAGTTTAGTGATAGCATGTGCAATTTCTACATCACCTTTGGATATTGTCACATGAACAGAAATTAGAATATTCATAGGGGCTGGTAATTTACGAAGTAGGCGAACTTCAACACT

>JAP00964.S|Supercontig_0000034:841954-842128

ACTGTCCCATTGATTTCGGATGTGAATTCACTTGAAACTGTCACACCATATACTGGGTTAAAACAAGTCTTTCCTTCGCCTCGTCCGATAGAACATTCTGTTTTTGTTTTGGCTAATAATGCTTTACATGCTTATACAAAAATGGGCAGACCATTAGCTTCTGTTGTGTAAGTTT

>JAP00976.S|Supercontig_0000488:135992-136125

TGTGATTTTCCTATATGACCAACATGATTAGGAACAGCAGATCCATAATTGATGGCATTTGTCTCGCCCATCGGTTTGTCATGAGACTTCCAAGCACAACAAGCACAAAAATTTCCCATTTTTAGTTTGTATTT

>JAP01043.S|Supercontig_0000245:1016059-1016182

GATTTTCTTTCTGCAACCAGTTTAATCTAGCTTTGTCAAATTCAAGTTGATTTTTACACAACTGACTATTTAGACCGTGAACTTTATTTTGTTCCGATGACAGAGCATGTTTTAAATGTTCAAT

>JAP01057.S|Supercontig_0000028:1939447-1939571

CAATTTTAGCTTTCAAGTTTTCATGAATTTCTCAACAGTCTTAGCCGTAATTTTGATTCAACCAAAGAATGCTTTTACACGATTCATCATCTTGTCAAGGCTACTATTGAACAACAACTAGTAAG

>JAP01106.S|Supercontig_0000032:1319933-1320152

TTGGGATGGTCCTTATTTTGATTGGTTATCTACTGTTCGACCATCAGCTTCCCCTTATCTACCTCAAACTGGTGATCGTGTCGTCTATTTATATCGTGGTCATCAAGATTACTTATCAAAAGCTTGGGAATGCGGCAATCTACCATTAATGGGTAATTTAGATCAACAAAAAACGAATTCTCCACCTTCCTTACCTTGGACTACTTGGCCTGATTTACCA

>JAP01140.S|Supercontig_0000104:441400-441506

GCATAATCACGAATTTTTTTGACACGTTCAACAGCATCCATTGAATGAATTGCTTGATTGAAATGATCTTTTACTGCATTTTCTGTTGTATGTAACATTAAATTTCT

>JAP01172.S|Supercontig_0000009:1317442-1317556

TAAGCGGCCACTTGTTCAACACGTGACATGACAGATTCTTCATCTTCTTCATCATGCATATGGGCAGTGGCTGCAGCGACGGCCGATGCTCTGATTGTTCGACCTCCTGTAGATC

>JAP01264.S|Supercontig_0000009:2287011-2287312

CTTTTCTGCTCTCTGAACTGGCTCTCCATTATCTTCCACAGTTATTACCATTTCATATGAACCAAGTTCATTTAGATTCAAACTTCTTGTTAACATAACACGACCACTTGGTTGATCCATTGTAAATGCATCAAGTACATTTTCATTTTTTAATCCTTGTTGATTAATATGTGAATATTGACGTGTTATACCATAACGTAATTGAGCATTTCTACCTTCATCTTCATCAATCGCTCTAGCAACTAATGCACAATAACCTGGTACTTCATTGTATGATACACGTACTACAGGATATTGTGAAT

>JAP01274.S|Supercontig_0000154:687171-687350

GGGCTTCTAGAGGTTCTTGAAGCACTTTGTGACTTCGCTATATGATTAGCAGTATCAGATGCTATAGTGTATTTAGACCCATCAGATTCAGGATAACGGCATCTCAAAGCACTGTTAACTGTGGCTCGATAATAACTTGGTGTCGGTGTCATTGGCTTGTAAAGGCATCTAAAAATAAAA

>JAP01280.S|Supercontig_0000193:1084782-1084901

TAGTCGTTGAAGATATAACACCACGGAATGAAGAGGAGAATGAAGAATTTCATATGGATACGATTCCAGATCATATTCTGAGAACACCAGAACGTCTTTCGCCACTCTTTATCAAGTTAT

>JAP01385.S|Supercontig_0000011:405759-405913

AAAGTTTACCGAAACTGCATTTCTTTCGTATGCACCCTTCTGTGCATGCGAAAATTGCTTGCATTACTGAATTTCTTTGCACACAATTCACATTGATATGGTGTTTCACCTGTATGAATTCGTATGTGAGCATAAAATCCACTAACATGGCGAAA

>JAP01388.S|Supercontig_0000028:1292524-1292823

TTTTAATAATAATCTTTTGTCGGCACTATCAATTGGTAATCAAAATACAGATAATTTCGGTAATCGCCCAGCTACCAGTGCACATTTAAGTCCTGGTTCTTTTTCAGAACCATCAAGTTATGCAACACAGCGTTCTTCACTTACTTCATCGAGACGTATTAAAAGGCCTGTTTTTGAACCTTATGATGAGTATTTTTATCCCAAAACTTCTCCAAACATAGAATCGAGATCGCGTTCCCCTATTCTACCTTTGAAACAAACAACTGTTTCACCTCCTACTAGCTATTGTGATTCAAATCG

>JAP01402.S|Supercontig_0000353:203205-203484

AATTATTAGTATTACTACAAATTATTAATTAACAATATGGGTCGTCCAGACAGTCGTAAACGTGGACGTAATACAAAATATGTCAAAGAATCTGATTCAAAAAGAACTAGGACAACTATTTATCGGCCTGAAGATGAAGATTCTACAATGTCAGGCTCACTGGATTTTAATAATGAATCTAATGAAGATGTTTATGATAATAGCGAGGTCAATGATTCTAATATCAAGAAAATTAGTAAAGTCGGCTCAGAGATTGATGATGATGTGGATGATAATGTTG

>JAP01420.S|Supercontig_0000124:144225-144328

AATTCAATTCATCAGTGTTGGCCGCCTACATTAGCTGGACAGTTTGCGAAATTACCTTGTCCATCAAATGTCACCGACTTTCATTACGATGAAAATGGTTAGTT

>JAP01423.S|Supercontig_0000333:20003-20068

AGAGTCCGAAATAAGAAGTGGAGTATGTGTATAAACTCTGCTTTGCGAGATACTTTGAAAAAAGAC

>JAP01488.S|Supercontig_0000077:1081525-1081743

TTTCCAAACCAATCAAGAACAATTGGCATTTGAATGTTTGAGCTCAGCAATTATTTTAAAACCGGATCATTTTGATGCAAATCAATTAGCTGGTGTAATTATTACACTGCATAAGGATTATGATGTGGCATTGAATAAATATCGATCAATATTAAAACAATCTTCTGAAAGTTCGATATTATGGAATAATATTGGTGTAGCACTAATGGGTAAAAGAAA

>JAP01495.S|Supercontig_0000235:777036-777210

ATAAACATCTCTGAGTTACAGTTTCAACATTACATTCATCACATTGTACTTTGAATCCATTGGCCATTCGACAGTTGCATTGAACAATTTCTCGTTTGGTCACATTTCTGTAACCACGGCCACAACACATATTCACACAATTTGATGTCCCCGTATCTTTCAATGTACAAATTCT

>JAP01505.S|Supercontig_0000005:419844-420056

ATTTGTTGCATCAGATTGAATACGTGGTCTTAATGGTATAAGACATGGTAAAGCAGTCATAGATAATGATTCAAGTACAGATGGTTTTTCACGATTAGGTTTAAATTTTTTTTGAAAAAGATCAAAAGTAGGACAATCTAAATGTAGTGCACTTGAAACATTTTTTAAACTTGTTTGAAATTCATTCGTAATATCTTTAGTAAATGTCCAACT

>JAP01507.S|Supercontig_0000043:1531609-1531738

TCGTCACTGTGGACGTTTACTTTGTGCTCAATGTTCAGCATTCGAAGTACCAATTGTAAAATATGAACTTTCTAAACCAGTTCGAGTATGTGAAGTTTGTTTTAATTTCCTGAATAATCCATTCTAGCAT

>JAP01517.S|Supercontig_0000093:1316668-1316827

TTCTCCAACTCCTGAATTCGTTCACACTTTTCAGTTATTTCTTTTTCGTATTTCGACTTAGCATTGGCCACTTCTACACTTAGAGTTCTTGCCTCTAGTGTTGCTCTTTCGGCACGCACAACTGTATTGTTTAGTTCATCTTGCAGTTGTTCGCATTGTT

>JAP01529.S|Supercontig_0015826:319-449

TCAACAGAAAAATCGCATCGATATCAACCTATTGGTCCAAATCCTAAATTACTTAGTTCACGTATCATTCCACATGGTCCGAATCCCTTATTTGTTCATGGACCAAATCCATCATTAGTACATGGTTCAAT

>JAP01530.S|Supercontig_0015826:319-449

TCAACAGAAAAATCGCATCGATATCAACCTATTGGTCCAAATCCTAAATTACTTAGTTCACGTATCATTCCACATGGTCCGAATCCCTTATTTGTTCATGGACCAAATCCATCATTAGTACATGGTTCAAT

>JAP01537.S|Supercontig_0000087:109194-109399

AATTATGGCATAAATGAAGCAATTAAATTAGGTGCAGAACATAATATAGCAAATGTAACAATAATATTACATAAACTAAATATAATTAAACAAAAACGATCAATCACACGAGCTGCGAATTTCCATTCTAAACTCATTAATTCATCTTGTTCATTATCACGTAATTTTTTAGTAATAAAACGTAATTCATTAATGATTAATTCTAA

>JAP01567.S|Supercontig_0000030:895957-896173

TAAAATATGAAAGACAAATTATTTGAAAGAAAAAAAAAAGAAAAAAATTACAAAATCTATAAAAAAATTTCATATCTTGATTCTTAAAGTTTTTTGAATCTCTTCAAAACGCTTCCTATCTTCATCATGTCTACGTAACATTTCTAATAGTTCCGCATCAGATTTCGACCATAATTTATAATCATTAGACTTTTTCAAGTTCTTATCATTTTCCTAA

>JAP01583.S|Supercontig_0000259:83950-84043

TCCCTTTAACTCTATCTGCTTATATAATGTATGTGTGTAAAGGTCAAGCCTCCAAGTATGCTGGTGTAAAAAAAGTGTCATATTTGACTCTATG

>JAP01585.S|Supercontig_0000018:441920-442132

AATGGAACACTTGTAAATGAATTCAATGGAATGAAACATTTACCATATGTGACACCAACATATGAACCAATTAATAGTACCGGTGATTTTCAAACATCTACAGGATATTCAGATTCACAAACTTATGATTTACCACGAATTCTTCCAACACACTCACCACCAACTATTCCTTTACCGCCATTACCAAATGGATTTATTAATAATGCTAGTAAT

>JAP01638.S|Supercontig_0000664:143108-143213

AATGAAGACCAATCGACTTCAGGTATTATACACCAATTTGGAGAAGTTTTTGTAAGTCGTCTATTAGGACAACTAAAATCATCAACTTCATGCCATAAATTACTAT

>JAP01650.S|Supercontig_0000364:226135-226697

TCCTTCGATCAAGTTTTTTCATCCAATATTATTCGGGAATCAATTTGTGAGTCGAATAAATGTGGTAAGCAACTGATAGTTTCTTCTCAAATAAGATCTTCGTTTGCAACTCCTAATGATCACCCATTTACCTCCAGTCCTTTTACTACTTCATGTTTTACAGAAAATGGTTATGTTGATGGTTTCACATCGATTAGTTCCAAAGAAGGAGGCTCAGTTACTCATACTCCTTTAATAATACCGACTCCAAGTTTTCATATGGCACCTCATCCTGTTAGCACACAATCTACATTTGTGGTTTCTCATCCTGTTACTCCTGGTGTTATCTCTCCTGCAGAGCTGCAGTCATCTGCTCCTATTGTTCTATCTCTTAAAGGTAGAGCATGTGTTGAAGCACCCCAGTCACAGCTTGCAAGTTTATCTACCAGTTCTGGTTGTGTTCTTCTACCATTACAAATCATAAATTCTCTACCAACTGTAAGTCCTGGTATGTCCATACTTCTAAATGTTAAAAACAATAATGTTGTACGACAAAATTCCACGCCATGTCTTCCAAATTTTAC

>JAP01656.S|Supercontig_0000067:599343-599543

TGAACAACGTTTAATTCAACTAAAATCTAGAAATAGTTTTCTTAATTTAGCATTACCGATTCTTTTGTTCAGTGAACCAGGACTGTGTCGACAAATACGTTTACCAAATGGAAAATATTTCAGTTTATGGGATCGTTGGATTATTCGACCATGTAAATCAATTGATAAATATCGATTAACTGATTTTATTGATCAGATCAA

>JAP01669.S|Supercontig_0000167:433571-433670

AAATTGAGATAAAACACAGTCGATTGTTTCTGAAAGGTGGAGCTCAAAATCCGGATCTCTACGAAGTACGCTTTTTCTTCGTGATTTCGGTTTACTTGTT

>JAP01674.S|Supercontig_0011174:3758-3922

CATTTCCTTTACCACCTATTGATATATTTAAAGACCGACTTGGAAGAAAGCAAAAGCTATGGCGACCATACAGTTCAAGACCGTTATTAATAAATAAATCAATTGCGAATCCTTCTAGTATGTTGACAAGAGCTTGCTCAACATTGTTAATTCAAAACCCTGAAA

>JAP01676.S|Supercontig_0000156:500200-500378

ACCTCTTCAACATCAGGATCTAGTTGTAAATGAGGTTCAAATTCAGTCAGCAACTCAGTTAAGGAACTCGATGTGAAAACAGAATTGGTTTGCTCATCAGTGATACACTTTGAGTCGAGATTCGTTTGACCATTTTCAGGTGAGCAAGGTATTTCGACGACAGTAACCCCAGCAGTTGA

>JAP01703.S|Supercontig_0000095:1252901-1253036

AGTCTACAATAGCTCAACAACTTGGATTACAAGTTTCAACAGTAGCAAATTTTTTTATGAATGCTCGTCGTCGATCACTAGATAAATGGCAAGAAGATACGAGTAAACTTTCGAGTACGATTAATTCACCATCGGA

>JAP01719.S|Supercontig_0000159:1056645-1056774

TGCACAAAAAGTAGCAAAGTAGAAAAATTGAGGGATGTTCAAAACTGCAGTATGTGCTTCTCTATCACCCAAAACAATACCTTTATTTATAATAACAAAAGATATAAACGAAAATGAAACTAAAACGAAA

>JAP01755.S|Supercontig_0000155:1385463-1385602

ACTTTCTAAACTTTGACCGGATTTCTTAGCTATTTCTAAACGTTGACAATGTGGTAAAGTTAGATAACGCCAGATTAAATCGCCATCTGCAACTTTACCGCATGGATTAGCTAATTCTGGTTGAGGACGATTATATGACC

>JAP01761.S|Supercontig_0000350:61048-61257

CATTATTCAACATACGCTACAAGACACAAGTATGCAAATATTTTCAAGAACATGGTGGTTATTGTCCAGTTGGAGTAAAATGCCATTTTGCACATGGTATAGAGGAACTGCGAGATCCTAAATCACATCCAAAATTTCGTAGTCAAATATGTCGAAACTATTCAACCACTGGTAACTGCTCATATGGTGATAAGTGTTATTTTAAGCATT

>JAP01762.S|Supercontig_0000064:738846-739017

AGAATGGCCTCCATATTCATCCATTACTGCTCCAGTTAGTCCTGGTGGATTAAAAAAAGAAAATCATAAAGCATTGTCTGGTACGTCTATACCATTTCATAGTTGTCGCTTTGGGCGTCTGATTATTTCACCTGATGGAGATGAAGCAAAAGAACTAAGGTCAATCACTTAT

>JAP01777.S|Supercontig_0000290:749870-749974

CTTTACACGGTCCAACACTTGATTTTCGAATACGTCGTACACCTACACATACTGTTTTACGTAATCAAAGCGTAAATATGATTCGTTCACCTGTATCAGCAATTT

>JAP01783.S|Supercontig_0000228:685521-685655

TTATTTATCTCAGTCTACTTTATTGTATATATGGGATCAGTTAATGTTATGTATTGTTGGAGCTGAACCGATTGAATCAAGTTTATCATATATTTTAAGTTGTTTCACAGCTATTTTCATTTTTCTATGCTGGAT

>JAP01788.S|Supercontig_0000121:1032358-1032427

ATTTGTCTGTTGATATAATCAATTGAATCAGTACAGAAGAATCCATCCTGATTGGATTCGCGTAAATGGT

>JAP01791.S|Supercontig_0000059:466989-467813

ATAAACATCTTCAAAACTATTATACAATGGTATCGGTGTAATACGTAGGAAATTTGGTAGTCTGTAATCACATATTACACCTAATCTCAATAGATTTTCATATAGTTTCTCAATCTTCACATTATTAAAACACAATGTTAACTGTGCTCCACGTTCCTCTGGACTCGATGGTGTAACAATACAATATCGATCACTATTTAGAGCTAATGAACTCTCTGTAATTAGATATTCAAGATAATTAGTTAGTTTGATTGATTTCTCCCTTAAATTATTCATACCACCACATGATTTAATAATACTGATACTGACTGTTAAAGCTGCAGCTAATAATAATGGTGGATTAGACAATCGATAGGCATCGGCACCTTTCGCTAATTCCATATTTCCAGTATAATTGAATCGTGTTTCAGTTCTATGACTCCACCAACCGGTCAGTTGAGGACCAGATATATCATTAATTATTGTATTATCACTACTATTAATATTGTTGAATTCATTAAATTTCGGTCCATAACTTGATTCTTGATGATGATGTTTTTCATGAATAAACAATCCTCCAATAGCACCGGGACTACCATTTAAATATTTATATGAACACCATACAGCCATATCAACATCCCAATCATGTAAATATAAAGGAATATTACCAACAGCATGTGCTAAATCCCATCCTACTGGACATTTACAATATTGATGACCCCATTCAGTAATTAATTTCATATTAAATAATTGTCCTGTAACATATTGTATTCCTGGTAACCAAATCAATGCAATTCTATGTTGATTTTTTTGAATTTCTTGTAAAATATCTTCATTTCTTAAACA

>JAP01792.S|Supercontig_0000238:324394-324680

TTACATTCCTTGAGCATCAAAATAATCTCTCATTTCAATTAATAATTGTTTTGCATCATTAGTTCGATTACCAATTTCATAGATTGCAATCAATAGGGAATTTTTTGTTAATAGACCACATAATTCATATTTGCCAACATTATATTCATTTGTATTTGGTCTTGTATAATATTGTAGTATATCAGCTAGAAGTATACCAGTTTCATCATCTACATCTTTATACAAACAAGAATCCATAGCTATTTGAAAGTGTCTATCTATAATTGCACGATCTGCTCGTTCAGCCT

>JAP01805.S|Supercontig_0000038:288656-288843

CTTTGTACTTGCTGAAGATGTCCGTCGTCGGTGCAGATTTGAACTATTCCAAAATAATACACTTGGTCTATGTTCAGTGATGTCAATATTTGCTAACGAACCAACAAATTTATCAGCTCCTTCTGATGCGTCCAAATTTGCTTCTGCAGCACCGCCTTCAGTTGTCATTTTTGTCCAATCTGGTAGTA

>JAP01818.S|Supercontig_0000483:60836-60989

CCTGTTGTATCCACAGTTTTATAGACTTCATCTTTTTGTCTTAAATGATTAATTGCTAAACCGAACAGTACACAAATAACTAAACTACACAATGATAAACCAATTGCAATAGATAGACTCATATCATGTGAGATATCACCAGCACAAACAACAC

>JAP01860.S|Supercontig_0000284:132370-132515

AAAAAATACCTGGAGTAATAACAGCTCGTCCACGTGTAACTACCGATGAAGAATTAGCTGCTGCACGTCAACGTTATCTAGATAGAAAGGCAGCTGGAATACATGCAATTATCGTTGAATCAGATTAATTATTTGATTAAGTGTTC

>JAP01889.S|Supercontig_0000055:511342-511476

CTTGAATTTGATTCATCTTGACAAAGCAACTTAACACAAACAGCTCCAGCATCATATAATGTCGGGTAGATTGATTTCAATCCAGGACAGACATTCCAATCACATGTAGATAAACATTTGAATGGAGTTGGATCT

>JAP01899.S|Supercontig_0001923:35200-35359

AAGATTTACAGAGGGGGCTTCATTGATTCGGGAACCCAATCGTTGGCTTTCGTCCTGATGGCTTGTGAAAGTGGACGTAATGCTAGCCGTTTGGCTGTTGGAAACTTGTCCGAATATTCTGTTTGCACATTAAGGCTCATTCAAAAATTCCTTGGAGTAA

>JAP01934.S|Supercontig_0000025:840202-840335

TGATGAGCACGAGCTTCATTCACGTCAACTGGTTGTGAATAGTTGTATTTGTAGTGTCCGATGGGATGTAATAGGTGAAGATATTTGATGGATGAACCAAGTGTGTATAGTAGACAGACAAGTGATAACGTAAT

>JAP02009.S|Supercontig_0000026:538749-538937

TTATTTAATTGAGCTAACAGACATGGAAGATACACAATCACCACCACTATCGGTCAAGTCTAGACGTTCCTCTAGATTTTGTATACGTCTTTCATGACCTTTAATAATTATCTTCATTTTGCGAATATCTTCCATGAGATGATGAATACCAGTGTGATTTACATGACAAGCACCGGTTGAAATACCATT

>JAP02047.S|Supercontig_0000007:162625-162762

ACTGATTTGCAACTCCAGAAGTTTGTTTCTGATTAGTGTATATTTCATTTAGTGAAAAAGCGTTAATCATATTGTCCCAAGTGAATGTGAATTTCTGTATTGTTTTCTTAGGTGTATTGATTATAGCTGGTCTGCGTG

>JAP02205.S|Supercontig_0000301:736069-736271

TACTTATCAGACAGCGATGAACAAATTGTTAACATACTGAGAAAAAAATTTCTCCAGACAACTTACGAATTTCAGAAAGCTGAGGTATCTAAACCAATATTCGACAAATGCATAGCGGAGATAGCATTCAATCCAGATAGAATCTATGTTATTTTGAATGACTTAAAAACAACCTTTAAGTTACACACACCAAAAATTGACAA

>JAP02209.S|Supercontig_0000028:1137799-1138154

ATATATCAGGTTTTGTTAAAACAAGATTCTTCTGGAGAAACTGTTGAATGAGTTAGAAGTGAAATTCGCTTATTATTAGATGCTATCGATTGTTGAGGATAAAGATCTTTGAATCGTTGTGTTTGCATGTCATGTTTTTGCATAACCTCTTGAAATGCTTGATGATGATGTCTGTATGATATAGAGGCTTGATTTGTATTCACCTGAGAATTTCTTGCTTCCATGCATAGATTGCCTAATTCACCTAATGAATCTATACAACTGGATTCTTCTTCATGTTCTACTTCATGATTTTTTAAATCACTGCTTAATACAGAAGTGATTCTTTCACGATCAGATGGCTGTGAAAAAGATTG

>JAP02213.S|Supercontig_0000678:313845-313964

TGATTCGGGAATGTCTGTGTTAACAAGAGGATATATGATCTCTGATGTATCAACAAAACGATTGAGCAATAGTTCTGTGATAACTAGCTTTCAGCCGTCTAGCAGTAATATGTGTGGTGA

>JAP02233.S|Supercontig_0000155:681987-682069

TCATCACAACAAAATGCAAATTCGATGATGCGTTCAATGAATACATCAGAAAGATCACTGGGATAACATCTTTTGACAGCTGG

>JAP02313.S|Supercontig_0000121:837919-838382

TTTCAGATGTCTTTGAATGTCATTAACCAGATTATATCACAGTTGACAAGCCACGGAACATTTAACCAGGTTTTCAGAGGAATCCAAGCTGTTTCACTAACAGATAACTGCCTAACATGTAGATTCAAAGTTACAAATTCTGAAGCCAACTCACTAAGCACTTTACATGGTGGATACATCCTTGGGGCTATCGATTTTATAACTTCTGTCGATCTGATGAGATTAGGGTGTATGAAACACGTTAGTGTTAATCTTGAAGCATCGTAAGTCATTGTATATGTATATATTCTAACTAAAAATTATCGAAGATTTATAAACCCAGGAAAATTGGATTCATGGATTCGGTCAGATTCATATATACTAAAGAAAGGGAACCGTATAGCCTTCTGTGAAATCAAATTCGTGAACGAACAGTCAGGGGAGCTAGTTGCACGTGGGACTCACACAAAATACATTATAGAAGA

>JAP02348.S|Supercontig_0000654:35239-35378

TACCGAAATTCTTGTGTTTAGAATAATGAACTGGTGTCCAAAATCGTAATATCCGCTTACTTTTCGATTCTATACATTTTCCCGCCATACAATAACCATTAAGACATGGACGATAATCTTGTAAATGAATATGTACTAAA

>JAP02357.S|Supercontig_0000048:115340-115429

CACTGATAGTAAAGAACTTTTACTGATGATTGTTGAAACTCAAACTTATTTACGTCGAGTTCACGAACGGATTGAGCTGTTAGCTTCATT

>JAP02371.S|Supercontig_0000193:13676-13954

ATCACGAAAATTATCCTGGTCCAGGGACATATAATATCACAACTGAACTAATACCAAAACATTTTATGTCTAGTTCAGTTTTCCTATCCAATGTACCACGCTGGACAATGCCAAATGTTCCACTTGCTCTTGGCATTGGTGATATGGTTGCAAATAGTTCTTCACTTACATGTCCTGGTGGAATTGATGCTTTACTTAATCCAGGACCAACAAAATATAATCCTTGCCTACCACAAAAAATGTCATTTCATTATAATCTAAATAATGAATGGATGTATT

>JAP02373.S|Supercontig_0000088:579753-579899

AGAGCTGATAACCAATCACCAAATGTATTTAAAGCGATTGATAATGAAGTTTGTAATTGTGTCTTATAGTCTGCTTCCTCAGGTGGAAAAGAATCCAATAACTTATCCATAGTATTAGTCCACTGAGTAAGACGTTTACCATAATGC

>JAP02374.S|Supercontig_0000638:68101-68227

TTTATTTTGAACTTGGTTTCTGAACAGGTGCACAGTCATATACTCCACGTACAACTTTATGTTTCACTCCGATAAGGTCTTGTGTGCGACCACCACGAACTAAAATAATATTATGTTCCTGTAAATT

>JAP02413.S|Supercontig_0000149:424109-424176

CGTTTTTTGCTTAGCGCACCAGGTACTTCACTTATACGACCTCTGTGAGGATTCCATGGTCTAAACCA

>JAP02419.S|Supercontig_0000015:1090796-1090886

TAATACAACTAATACTTTCTTGGATCATGTTTGATTGATTCAGATGGATAGCATTTTTCCCTGTACTTTTCTAATGAATCTCTCACTGAAC

>JAP02422.S|Supercontig_0000007:136780-136966

ATAACTTTTATAATATCGTTTTGCGAATTGTTCAGATGTACTACATTGTTTAACTTCCTTTGCACATTGAATTCCACAGCATAACATACATATTGGAACCGCTGAATAGTTGAATAAATCAACACAATGCATACAGATTGTATTCTTTTGATCATATCTATCCATGCCACACCAGCAACAAGAACCA

>JAP02461.S|Supercontig_0000567:10124-10227

AGTTCCAGATAATTGTTCAAGTGAAGAACTTCAGGCTGCGTACTTTTCTTCTTGTTTTGAGCATAGAATAACTCCCATTAATTTTTTGTTGGAACAAATAAAGG

>JAP02469.S|Supercontig_0000567:10124-10227

AGTTCCAGATAATTGTTCAAGTGAAGAACTTCAGGCTGCGTACTTTTCTTCTTGTTTTGAGCATAGAATAACTCCCATTAATTTTTTGTTGGAACAAATAAAGG

>JAP02489.S|Supercontig_0000150:433979-434081

AAGAACATGAAGATGGTGTAACATGTGCTGTGATATCAGGTTCTGTGATTATTTCTGGTTCTTATGATAAAACTGTCATACTTTATGATTTCGATGTGATTTA

>JAP02498.S|Supercontig_0000064:771977-772165

ATAATAAAAAAAGCTGAAAATCAAGATATGAATTCGATTCAACGTATTGTCGCATATGATCATTTAGAATTTGAAACAAGTATGTTCATATTCTTGTTTTATTTTTCTATGAAGGTGTTATTGAGCAAGCTAAAAATACTTTCAAGAGAAATAATAGTCTTCAGGCCAGTGCGACGAGGGAAAGACTTT

>JAP02503.S|Supercontig_0000104:233730-233838

GATAAGTGGGGGAGATGGAATTATGCAGTAGTAGCAATGGATGCTACTAAGTACAGTAATCCTATGGAACAGTACAATGTCGAGGAAATGCTTAGAGAATTAAATAAGG

>JAP02583.S|Supercontig_0000011:637219-637299

CTTCATATTTTGCCAAAAGTATTTTCTTCGTACACGACCAGCAACTGCCTGAAACTGTTTGGATTGAACTTGTAATTCATC

>JAP02599.S|Supercontig_0000132:315288-315522

AAATGTTGAAGTTGTGCACGTAAATTAACACGCTGATCATTCAACTCAGCAAGTTCACCTTGCATTTCATTATTTAAACGAAGTAACGTGTGATTGACCGATTTGGCTTCCCGAATAAGTGAAGACAAAAGACGACGTCGACGACTACTAGATAATTCAGATAAAAACGTTTTTCGATGGCCAGTGATTGCAGATAATAATTGGATCGGATCAATATACCTAATTGTAAGATAAA

>JAP02611.S|Supercontig_0000171:302796-303024

TAGATTCGTTTACCCAGACGACTAGAAGTCAAATTTAGTGTAGGTGGTGTACATTTCTCTGGTCAATTGCCTATTTCACGTGAATTCAAATTAGAGAATCGAAGTTTAGGCGTTTTTAGCATTATTTTGGATTTATCTCACAGAATTGGTCAAGTTGTACAATTGAAAGCCTTTTTCGCTGACGATTGGTTGTTGTTCAGTGAAATACGCTTTGAAAGTGGTAAGTTTT

>JAP02614.S|Supercontig_0015826:319-449

TCAACAGAAAAATCGCATCGATATCAACCTATTGGTCCAAATCCTAAATTACTTAGTTCACGTATCATTCCACATGGTCCGAATCCCTTATTTGTTCATGGACCAAATCCATCATTAGTACATGGTTCAAT

>JAP02624.S|Supercontig_0000082:181479-181611

TTTAGGTGCATAGCCTAGGACGAAATAACAATCAACAATTATTACTTTATCCTGTTGGACAAGAAAATCCCACTTTACCTGATTATTTGGATATATTTTATTCTAGACCTGATTCAAATTCTTCTACAAGATT

>JAP02638.S|Supercontig_0000223:533956-534074

GTCTAATTCATCTTTACCAATGGTTCACAACCCTCCAGGATTTTGGGATCCAAAAACATTTCCTTATATGTTTCATGTGCCTCGAAATAATTTTATGTTTTGTGAAAGAGAAAGGTCAC

>JAP02655.S|Supercontig_0000115:270684-270888

TAGACTATAGAACATTCACGTTATTTCTTAGGAGGGATAGCAACAAGACAATGTGTACAACATTGTAAATCAGTATCAAATTGGCTTTATTGGAGTTATAAATCAGTACAATTTACAGTACATTGTTGTACTAAAGATTTATGTAATACAGGCAATTTTAAATCAATCAATATGTATCTACTTTTAATTTGTCCTCTAATATGGC

>JAP02680.S|Supercontig_0000168:169983-170101

CAAAACGAATTCGGGAAGCCTCTCATATTATTGCTTGGAGTTATTGCCTTGTTCACTGCTATGATTTTTGTTGGTTTAATACTAGTAGTAGGGTTTTGGTTCGCAGGATCTTTTGAAAA

>JAP02697.S|Supercontig_0002061:367007-367208

AATTTTTGTTATCTTACAGCACCAAAATAAATAGCTGTACAAACTATGTATGAAAATACAGTGACTAAAAATAGCTATAGATTAATATGAATCTCAGTTATTATCGTTGAAAGATATATGACGGTTTGGAGGGTAAATTTTCTGCAGTACTAAATTTAGTCCATATTGTTTTCAGGAGACGAGTAGGTTTAGTTATCTGAAA

>JAP02709.S|Supercontig_0000358:264852-264985

GATCTAGGCTCGCTTAGCCTGCATTTTATTTTGTAGATGAAAAATATCAGTAGAGAAACAACGAGAAGTGCTGGAATGATAAACACGGCGTTGCTTATTTCTTTTTTAACAGGCCCTTTGTCTAGATCCACCAT

>JAP02713.S|Supercontig_0000103:241066-241200

ATTTTAGGCAGCAGCATTGAACTCAGTCAATGAATATGTGAAACGAAATGAAGTGTCCAAATCATTTCATATATTACGTGAACATTTTGGTTTTAAAAGTTTACCTTCAAACCGTTATGTTGTTGATATTTTATA

>JAP02714.S|Supercontig_0000103:230707-230893

CATTAAAGTTGTCTAAAATATGTCGATCTACATTGTGTCGTTTTCCAAACCAATCACCTTTGTTACAAGCCAGCATGGAAGTTGAGCTAGAGCAACATCTACGATCTGGTTATGATTTAGCTCAATGTGATCGTTTAGTCAATTCATTAAAACTTTATTGTCCATGGGAATCTGCTATAAGGCAAGT

>JAP02729.S|Supercontig_0000159:903443-903535

TCAATTGTAATCATCTTTTAATTTACGAGGTAGACCAGATAATGAACTTGTGAATGCTGGTTTACGTTTACGTGCACCAACATTAACTGTACT

>JAP02730.S|Supercontig_0000132:1071598-1071749

TATTTGGATTTTCATCGTCATCCTGTTGATGCAGGAGATTTTGTTCTTGTACCTCAGTGTATATCTGATTGCGAGGAATTAAATCATTCACAAAAGCATTATCTTGTTCCTTATTATGTTGCAAAAGTTTTATCTGGTTATGAATCGCGTTC

>JAP02765.S|Supercontig_0000195:358472-358639

TTTTAGTTGAATGAAGAGCAGTTAAAACAAATCCTCCTGCAGGTTTCTTCGGGCACACAGAAATCTACCACAGTAAAGGTAAGATATTTAACTCATTAGTTTTACGACTTACGTTCACATAGTTTGATCGTCGTCGTGCAGCTATCGATTCAGACGAGGATTCACTTT

>JAP02814.S|Supercontig_0000144:161094-161342

GCATATAATGCACGTTATTGTTTAGGTCAATGTCCATTTCCATTATCAACTCATTATAATACAACTAATCATGCTGTACTTCTTCAATTAGTACATTTATTAGATGTAGCTAGAATTTCTGGTCCGTGTTGTGTACCACATCAATTATCATCACAATCATTATTATATCATAGTCAAAATGGAGATGTTGTATTAAGAGTTTATGAAGATATGGTTGTAGAAAGTTGTGCTTGTCGTTAATATAAAAAA

>JAP02816.S|Supercontig_0000144:161196-161342

GATGTAGCTAGAATTTCTGGTCCGTGTTGTGTACCACATCAATTATCATCACAATCATTATTATATCATAGTCAAAATGGAGATGTTGTATTAAGAGTTTATGAAGATATGGTTGTAGAAAGTTGTGCTTGTCGTTAATATAAAAAA

>JAP02834.S|Supercontig_0000184:669942-670070

TACACATCAGGTATACTTTATGGATTTATTAGATAATTTTGGTTTTCCTGAATTTGAAGAACCGGATAAACTCAGACGTATTCGTGAACGTTGGCGACCAGATGATTCACCACATCCTGCTTTAGTTGG

>JAP02837.S|Supercontig_0000174:408359-408493

AATTTCAGGAATATAGACGTTGGAGAAGAAATGACTTGGGACTATGGATATACTGTTGATGCAGTACCATTTAAAGTTCTCTACTGTTATTGTGGTGAACCTAACTGCCGAATAAGACTGTTGTAATATATTGTA

>JAP02840.S|Supercontig_0000180:274690-274878

GTCAAAAACAGGAATCTTCGTAGATTTTGCTTCTGCAGTTCTGATGATGGACGTGTTTTTACGAAATGGTGAATGGAATTCTGCTGTTGCTGTTTCATGGGAACTCTGCCTTCAGGAATATTTCAGCTTAGAGAATGTCAGACCCCTTGTCTTTGCTGCTTCACTTTTTTCATGCATAAAGTCAATAGA

>JAP02898.S|Supercontig_0000285:468159-468268

CATACCTTTTATTTGATCATTTGTCCACAGATTAATATCATTTTCATCTGCCTGCCAAGCACTCACAAACATACCAGGATTATTACGTTTGGCAGCCATTACCTAGAAAT

>JAP02903.S|Supercontig_0000195:757903-758121

TTTTCTCTTCCAGAGAATTGTCAGAGTTCCGTCGGGTCCTGTACAAAATCCTGAAAATGTCGGCACATTGACACGACGACCTCAACAAACAGAATACGGGAGTAAATCTCCACCAATCAGCTCACCTGATCTATCACCTCAACATCATACTCTGAGTCCTCAACTACGTCAGGCAAAAAGTCGAACAATATCTGGCGTTGAAGGGTTATCTAATCCAGA

>JAP02917.S|Supercontig_0000104:232532-232755

GATTTCTGTTGGACTGGAGATTTTAATCATTCGGATAGTGGGATGAAACGGTAAGTTTCACATACATTCATAATACTGATCAACCGGATGTGATACTTGGGAGTTATTAAGAATATAAGATATCCGTCGGATTATTTCCCGTCTTCATACACGGTTAGTAAGTGGGTTACCATAAAAATGTTTGTTATTAGATTCCTAAAACATATTTTGGTCTTTCAGTTTAA

>JAP02924.S|Supercontig_0000174:408359-408493

AATTTCAGGAATATAGACGTTGGAGAAGAAATGACTTGGGACTATGGATATACTGTTGATGCAGTACCATTTAAAGTTCTCTACTGTTATTGTGGTGAACCTAACTGCCGAATAAGACTGTTGTAATATATTGTA

>JAP02951.S|Supercontig_0000598:11874-12035

ATGGCATAAAAAAGATGAGCATCAATCAATTCCTACTTGGCCTCCACCTTCATCATCTTCAATAGTGGATGAATCAAAATTAAATGGTCCTAGGCCTAGGATTTATGCTTTACGTGGACGTCATTTAGTAATTCATTCCGTTACTGAATTAGATTCTGGATC

>JAP03011.S|Supercontig_0000474:284535-284657

ACCTTTAGGTTTTTTTCGTGTACTATGACGAAACAATGAAACTGTTCTTTTTACAAGTGGCCTGTCTTCTTCACGCATTGCCATCCATGGATGACTTAAACACTGATCTACTGACGGTCTTTT

>JAP03054.S|Supercontig_0000008:346840-346939

TTAGAGTAGAACATTTGGAACTATTGGATGACCTTGAAGTGACAATGCAGTTGTTTGATCATTATTGTATTCTCCTGGCAACTAATGATGAAACACTCTG

>JAP03056.S|Supercontig_0000153:690687-690796

TGAGACTGATGTGATGAAGATTGAGAATGTGATTCAACATCAACTTTACTTGTAAGTAATAATCTCTTTGAAGAACTATCCCATTTGAACGTCCTTGGGGAATTTACACT

>JAP03164.S|Supercontig_0000385:537577-537759

AACTGGACTTGTAGAAAATCGTGATAATTCTCTACTAGCTGAACATCCTATTGTATTTCCTAAAGGAAATAAATGTGGATTTGTTTGTAGTATCGGAGAGAGTTTACTTGAAAAATTAGAGCTACAAGATGGTGATTTTTTTCCTTGTAAAATACCTGACCGTCGATGACGTGATTCATATAA

>JAP03199.S|Supercontig_0000065:377678-377818

TGTGACGAACGTTTTGCATCAAATAATGCGCAATTGAACTTAGCTGGTTGGATGCAAACAAAATGCAAAGTATACAATAATGTGGAATTAGTTGTAAAAGATACCGATGAATTCTTTCGTAATATGCGCAATAAAGTAAGT

>JAP03209.S|Supercontig_0000196:988754-988916

AGCCAAAAGAAAACGTGAACTGATGGATATTGATTCCGAATCAGGTTTGTCAGAAGAGGAAACAATGGACCTTTCACTTACTGAACTTGAGAAGGAGACAAGTAAAAAGAAAGTGGTTGATAAGTCAAAAACTCGTGGCAAGAAACAAAAAATCGCTTTGGAA

>JAP03235.S|Supercontig_0000156:846517-846736

ATTTATGTGAACGACATGCTCGTCCACATTTTAATGGGCATAAATTTTTTTCATAAATAGCTTCCCAATATGCATGATATGGTCGAAATAATTGACATAGAAATGCTTCTAATACTGTATGTACAAATCTACGATTGAAACGTTCTTCTTGAAGCATATTGAATACACATGAAACACCACGACGAGTAGTTTCATTTCCAAGAAATTGTGCAAATTCCTC

>JAP03279.S|Supercontig_0000154:339072-339178

AGATCAATATTAAACATATGTATGGCATATAATAGTCGAAATGATATTACGAATGCAATGGAAATTGTACGTTTAGGTGTGAAGGAAGGAAAAATTATACCTAGGTA

>JAP03294.S|Supercontig_0000156:1423985-1424183

TGATTGAGCCGCACACCAATTCGAACATCTTCAGTGCCCATCATCTTTTTAGCAAACATTTTTATTTCTTTAATAGCTCGAGGAGCTCGCCTTTTGAAACCAACACCATGAATTCGTTTGTGCAAATGAATTGTATATTCGCGAGTAGCAACACCTTTTCTGGCAGTTCTTCTAGCTTTCGTTCCAGATTGCACCATGG

>JAP03296.S|Supercontig_0000023:928129-928229

GTTACACGTGTTGAACTAAAAGATACTTTGAATGCGGATAATTATCTTTTGCATCATCAAGAGGCAGATTCAGATGGTAACGTTAAAACAAGTCTTTTTAA

>JAP03474.S|Supercontig_0000291:123092-123211

CTCAATCCAACAACTTCTGAAATACAATTAAATCCAGCACGTGTCAGCCCATGTAATATAGCTGGAGATAATGGCAAAGAGATGAGTTCACGGAATCCCGGATCGGATAACATTCATAGA

>JAP03506.S|Supercontig_0000378:265813-265959

ACTTACATCTTCAACTACTGATGTTCCAAATTGTAAAATTTCTGCACATGTTTCAGTATTAGTCCCACTGGTTTGACTTGTTAAAATTGGTAAATCTAAACTATGAGCAGCAATTAATCCATCATCACAACAATGTTTAAACTGATT

>JAP03554.S|Supercontig_0000053:572408-572507

GGTGGATTGGGTACAGAGGCTGCGTAACCTCCCGTATAACCTGCCCAATAATCGGTGGGTGTGGGTAAATAGTAAGGAGGAACCATTGCCGTACCTAAAA

>JAP03628.S|Supercontig_0000095:1170996-1171180

TGAAATTTTTGAATAGAAATTAGAATGATCCATTAAAGCTAATCCCGGTTCAACGGGTAATTCCGGAGCTAAATCAAAACTTGACTTAGTATATCTTATTCCTATTCTACTTTGATTATGGTAAGCGGCTAAATCATTAGCACTAAACGAATTAGTCATTGAGCGTAAAGCCGGTGACTGTAAAA

>JAP03677.S|Supercontig_0000661:51357-51514

AAATATCTGTCTTGGACAGATCCACATGAATTGGCTCAGTATCTTAGTCAGCGTATTGTAGCTTTAACTAAACATTTTGTTGTGATTGATAAACCACCTAATCTTTCTGTTTGGGGTCATTCACTAGCTAGTCGTGAAGCTATTACAATGATTAATCC

>JAP03707.S|Supercontig_0019079:15536-15661

TATACCTTAGATCTCCTGGTCTTGGGAGCAATTTTTTCTTTTCTTTAAAGTACACTATAAAGTCTTCAGTTTTTAAACGATGTGCATAATCTATATAGCCAGATATTTCTTGGCCACGTGAATTCT

>JAP03711.S|Supercontig_0000357:15171-15480

AAATATTTTATTTATATTTTATTTTACAATATTTGCATATAAGTGACTGTTTTATTTACTCAAATAATTCAGTCGGCCAGCGGTTCTGTCTTTTGATGTTCATTCAGTTGCGATTTGAGGACTCAAAAAGTCAAAGTGGATAAAACCAACAGGATGGATTTCGTGAGTATTGTCTATTCAGCCATCTCTCTTCAGTGTTCGTTCGACCTGCGTTCGATCTCAATCAAAATGACGGTTGTTACAGGTTTGTAAATTGGCCGTATCCTGCACCCATTTTGTCAGGGATATTACTGTAAATTTTTTGTTTAGT

>JAP03747.S|Supercontig_0000094:630276-630621

GCGGAATGGTTTTCATGGCTGGATTACTGGTGAACGACCGACGTCATCAGATGCCACACACAAAGCCGATAACGTCAGACGTTTATTGGCATTTCAGCGGCTATCAACCCCAAGAAATCAAACAATTTTATTGCAACAAGCCCCTAGACAGATAAAGGGTGTGCATTTAGCGCCTTCGTTCATCTCACAATATTATCAAACAGAAAACTTGGAAGGTAATGAAGCTTCGACTTCGAGTGACACTAATCGATTTGTCCAACCAAACATTTTAATACAACCAGAAAAGTCCTCAAAACTAGACGTGGTGAATGATGGTTCATCTCGGATTAACTGGCCTCCAGAATCT

>JAP03757.S|Supercontig_0000156:930410-930729

AGTTTCAATATGTTTGGAACGTGTTCTTTAAAAAATGTTCGGCGTATTATACGCCAGAGTCGGCGTCTTAAACCATTAAGAGTTCACTTACTAAAATCTGTATTAGATTTATGCAGAGCTGCTGACTTGGAACCATTTGTTTATGGTGGTACAGCTTTGTCAGTATTCCGTGAACATGGAAAATTCATACCGCATGATACAGATATTGACTTGGGTATTTTAGAATTTGATTCTGAAGGAAACAGTTCATACACTAAACTGATTCACTGCTGTCTAAAAAATGGCATAAGTGTTTTCGGTGTGGAAAATATGGATGATTC

>JAP03782.S|Supercontig_0000685:864726-865074

AATGATTGAAAGCAAAGTCCGAAGCTTGCAGTATATTTTTGAGTTCCCTCAAATTACAGACCTATTCAATGTCTCTGAACGACAAGGACAAGGTACATTCGCCTCTGTCTTAGCAGTCAGAAGTAAACAAGATCCTGACGAATCTAAGTATTACGCTTTGAAAATGATCATCCCAACAGTCGATGTTCGTAGAATTGAAAATGAAATTCGTATTCTGAGAAGACTGGGGTAATATATAGTTGTTCATTCATACAATAAAATATTTTCAGTGGTAAACATAATGTAATTCAAATGCATACAGCAATGCGGATCCGTGATCATGTCTTCATTTTAATGCCTTTTATTGATT

>JAP03785.S|Supercontig_0000144:329916-330105

TCATCTGATTTCACTAAGTGTTCTGACTGCTTTGAAAGCCATTGAATTACAGGAGCGAAATGTGACGAATTAGTATTTGAACAGGAACATTCAAAAGACAGTTGTTCATTATAATCATATTTCTTAAACTTGCCAAAATCATTAAGCTTACCTGTCGTTTGAAGTAGTTTTGAAATACATGAATATCTAG

>JAP03826.S|Supercontig_0000055:1078462-1078764

ATTTATGTAAAAAAGAAACGGAAAATATTCTAGATGAATATGATAACAGTGAGGAAAAGTTAATTGAGAAAAATTCAGTGTTAGATTCCACTTTTACAAATGATTACATACCAGATCATTTATCATCGTTAGGTCAAAGTGGTTACTCTAACACTGATCTAGATGATACGACTAAATTAACTAAATATTCATTGAGTTCTTGTTTAAATGATTCGCATCAAAGTAATCCATATTTAAATTCTATGCAATCAACAGAAGATGAGAGTCAATACCCAAATGGTGAAAATGATCAAAATAATAATA

>JAP03852.S|Supercontig_0000360:351853-352009

CTAAACGAAATGAGTAGGATATTTCTTCATCTCTAGTACGCGACACAGTGAAACGTTCTAACAGTACCACACCTGAGATTTCGTTGCTCGTTCTATCGGGAGAACGGCAAAGAAGCAGAATGTTTCCTTTCAATCGGCACCAGTGTTCAGAAAAAAC

>JAP03855.S|Supercontig_0000005:3450856-3451027

ATCCGTCAAAACTATCTCTTAAACGATTGATGCTACCACTAAGAGCTTGACGCCATCTTCTTTCATCATTAACTAGATCACCGCTTGTACCAAGATCAAATCTGGGGGAGATACGTTGAGTATTATTATATGACGTGTTTGAGGAAAGATTTTGTGGATGGATTGGAGATGA

>JAP03890.S|Supercontig_0000225:163437-163712

TTTTTAGTTATTCAAAATTTTAAAAGAATTTCAAGCACGGTTTAATTCTCCGTTTCGACTCGCTGAAGCGACTAGATGGTGTGAAAGCTACTTCATACGTGAGGGTGGTAACGTCGAAACTGTTGGCGTGTTTTTAGAATTTATCATGAGCCTGGGATTCGTTTATCAGGTCGACCAAAACGTTTTCTTTTCCAGATACTAGTAGGATTTTATTTCTTCCCAGATATTTTCTTAGTTTTAATGTAGAAAGAATTGAGGAGTTTTTCAGCACCTTTT

>JAP03906.S|Supercontig_0000067:491025-491359

AACTGTTGTACTGTAGTACCCAGTCGTGGTTGCACTTTAATTGACGTGCGCGGGAAGCCCCTAAGCCTCCAGAGCCAAACATTGAACTGACGCAGGGAAGGATCTACGTTTGCATCAGGAAAGCGACTATCTGGAACACGCTGATTTGTACCAAGTGTTGACCGAGCATCGTAGATAGCAAAATCAGAAAATAGTTTATTTCTTTTTAAATGTCCACTTAGCTTAGCTTGTTCTAGTGCCAATGTCAACCCAGACTTTTCTGATTTTAATGCTGTATCCGGTTTGTTTGTGCGGACTATCGGTTTTGATGGAAAATACTTGTCTACTTCCTCAGC

>JAP03944.S|Supercontig_0000633:198062-198237

ACCCAACCACCATGACTTTCGACCCATTTGTATATACCATGAATAAAAACAAATTTAACAACAAATCTTATGATATCACCAGCAAGACCACTAAATCCTTTGGAAATTGCATTGATGACCACTTTGACAGCGAACATGAACAATACCACAATTCGTGACCAGTTAATAATCCCATC

>JAP04007.S|Supercontig_0000007:915532-915660

TTGATAATCAGTATCGGGTAAACGTCCAGTTTGTCTACGTAATAATTCCATTTGGTATTGATAAATTAAATGAGATAATTGAATATCAGAAATCTCAGGAAAATATGATAATGGATTACGTTTATTATT

>JAP04029.S|Supercontig_0000349:79233-79384

CCATCTGTCCCGTCTTCGAAAGAAATGACCAGTATACTTATGTCTTGTCAATCATTTGATGATCAAGTTTCCACTTTTTATCATCTCACACGTTTAACTGAACAGGATATTTTGGCTAGATTTGTTCTAAGTGACAGTATTGAAGAAATCCT

>JAP04055.S|Supercontig_0000003:1117546-1117691

AGAAAGTCACAGTGAAATTGAGAGGAAACGACGTGAACGAATGAAATTAGAAACAGAATTTCTACACCGTCAAGTACCACATTCACAATGTAAAGATAAATTATCCATTTTTCTTGCTGGAGCTGAACGACTTATTCATTATAACA

>JAP04072.S|Supercontig_0000067:14593-15039

GATAAAGATGATCGCTATATTCAGCGTAGACTGAAAAACAACTTGGCAGCTAAACGTTCCAGAGACAACCGCAAGCGTCGTGAAGATACAATAGCATTACGTGCAAGTTATCTTGAAAAATCCAACTTGGTACTACAAACTCAAATACTAGCTTTAAAAAGGGAAGTTTGTTTGCTTCGTGGTATACCATTTGATCCAAACTACAAAGTTCGTGTTTTTAATGACAATTATTTATCCTCTTGCCAAACTACCCCACAGTCTACTGTGTCTACTGACTTCTCAAATGAATGTCCTTTGCCAGATTTTTCTCCTATTGCGTGTTCAAATCAGACCTTAAACTCAAATGTCCCTTATCCTCCAATCACTGAATTTTCTGGAAATTGTGGATGAATGTATATTTACTATTTTTGTATGTACATTACACAGAGCTTTCTATTTATTCCTTTT

>JAP04117.S|Supercontig_0000491:457474-457576

AGGCTGTAAATCATGATTTATACCTTGATTATTCTGGTTTAGTAAGTTTACTCTTATATAGAGCTGCATTTTCAATGCGATTCGCCAATTCTTTATATTGGTA

>JAP04270.S|Supercontig_0000124:1228430-1228513

AAAAAATCATCTGAACATCGTCGATTTTTCACAGGAACACGTAATGATCCATGAATAGAAGACAGAGAAATACCCTTACTATTA

>JAP04284.S|Supercontig_0019090:253007-253140

CAAATGTCTAACTATATTAATGAGGAAATAAATCATCTTCATAATTATTCTGGACGACATAATCCACATCCTCAAAGAGTAACATATTTTACTGGTTTTCTTGGTTTACCTGTACGTGTAGTACATGATCCAAT

>JAP04349.S|Supercontig_0000033:687474-687556

TAACCATATTTGTCGTATCCGTATTTGTCGTAACCATATTTATCATAGCCGTACTTCTCGTAACCATATTTGTCATATCCATA

>JAP04364.S|Supercontig_0000042:843813-843904

AAAGATGTTGACGTTTTCCAACGTGCATTTGCTCTCAAGGGGATGTATCGGTCCATGTAATTTTCCTTCGTTGGAGTCTTCATGGGGCTAAC

>JAP04437.S|Supercontig_0000304:851488-851597

AGGTTTTTGCATTCGATCCAATAGATAATGCTAGATATATGCGTCCATTGTTGTATAATCCTGGTATACCTGTTCCTTCTTCAGATCCAGATGCTCCACAATTTGTTTGA

>JAP04484.S|Supercontig_0000122:804974-805085

AGATACGAATTATATTGTATGGCCAAATCTCATAAACATCAACAGATTGTTTTGTTCAGCGATATCCCGCCTGAAATTTCAGAAAATTGGAATTCGAAAGTAAATTTTAATT

>JAP04511.S|Supercontig_0000025:453981-454207

AGTATATTTTTCAAGTCCAGATCTTCTGGTTTTACTTGATGATTTAAGTGCACAATTTTAGGTTGTTTCCCATATATTAAATCCCCACTGTTTAATAGTGAATTTTCGGTCAACCAGCTAACTGGACATCTGTGTGTATCAACACCTGAACTGAGTTGTATGTTCCATTCACATGGTATTTCGTAAAATATCTCATTGTTTCTTAACATAATTAAATTTATAACATC

>JAP04516.S|Supercontig_0000042:843700-843765

ATCATTACTGAGTCCATCAGTATTTCCGCGACTACTTTGAAACAGCCTACGTGTCGGATTACTATT

>JAP04530.S|Supercontig_0000106:802571-802688

AGATGGGGACATTTGGTGGGCATGCATTACCTGGAAGCTTTTTTATTATCTATGGTTTATGGTCCATTTGGCATATATTAGAGAAATTTTATCAACGAAAAAGTTACGAATTAAATAA

>JAP04683.S|Supercontig_0000261:459463-459614

CTTGCAGGGACTATGGCCCATTCGGTTCCAACCATGACATCTATCACAATACCTGCTGGATCTCTAGTTATGCCGCCGCCTCCACCTCCACCAATTCAAACTGCCTTGGTGGGATTCGGGCAGTCTATGGTACATGGTGCCATGCCACCTCC

>JAP04800.S|Supercontig_0000083:216253-216534

TAAGTGAATCATCGATTCCCAATGCAAATGCAGATAAATATAAACCATGATATGATTCGGAAAAGCGTTCAATAAATTCTTGTAAATATTTATAGCAAGTTCCAAGTTTTAATAATTCCGCATAAAGGACAAGTTCACCATGTGGTACAAAAGGAAGATTATTTGATATGGGTATTAAATGTTGGTCTAGACCTAAATCATCATCTTCTTCAGTCTTATCAGACTGGACGAATATACCACCGGAAATCCCATGTAAAGCAAGTAGCAATTCATGTAACATTT

>JAP04913.S|Supercontig_0000061:65923-66096

TTGGTCTGTCGTTCTGATGACATTCCCACATTTGTCATTATTTTCGGAAACAGTAATTAAAATATTATTTCCTATTTGGGTATTCGTCTTATTTACCTTATTCTGCGGAACACTTGTTCTCATTCCAAATGCAACTGGCACAGTTTTTGGGCCAGTCAATTTAGCTGTGAATTA

>JAP04981.S|Supercontig_0003363:687-942

GTCAATTGACAAATATTATTTCCCAATCTTAAAAGTTCCATTGATGGCATAATTAAAGTAGATTTGCATAACTTAGTTTCATTTACAGTTATATTATTACTGTTACCGTTCTCTGTTTAATAATCGTGCTTGATGTGGATAGGAATAAACTTGTTAGCGTATTTTCAGCATACTTCCATGTCATCTCCAAATATCGACCTATGATTTGATGTCGTGTACTAACTTCACGATGATCACTCCATTGTACACCCTTTTT

>JAP05180.S|Supercontig_0000255:263095-263156

GAACATGATATTCGGCAACAAGGTTGCGACTGCGTCATTTGACCAGCTGGCACCAGGTGGTA

>JAP05338.S|Supercontig_0000036:811415-811489

ACACACCTCTCAATTCAACTCACTGTAATATAGTCGTGTTTGAATTCGAGATGAATAAAACCTATTCATTCTAAA

>JAP05390.S|Supercontig_0000245:751873-751963

CTTGATAAGCAGGACCACCATCAATTACAGTTCTTACAAAGACAGCATTCCATTGTGAATCATTAGTTTTGTCAATTTGTACACTTGAATG

>JAP05396.S|Supercontig_0000035:508938-509032

TGAAAAAGTTCACACGTAAAATGCGCAATCAACAAAGACCCTCTACTACATTATTTTTCGGTATTTTAAATAGAGTTCCTAGTAGAATGGAGAAG

>JAP05403.S|Supercontig_0002065:917529-917654

CCAGCTATAGCCATAAACATGAATACAACATCTACATAAAGTTGCATTGCACCAAATATATACTCTTCTGGTTCAAGTTCAAATTCTCTTCCACCCATTATTAGTTGTGTATCGTAAGCCAAATAC

>JAP05407.S|Supercontig_0000036:836280-836355

ACACACCTCTCAATTCAACTCACTGTAATATAGTCGTGTTTGAATTCGAGATGAATAAAACCTATTCATTCTAAAT

>JAP05409.S|Supercontig_0000003:1409254-1409400

GATTAAACTATTTCTTTTCGACCAGGTTCGGTACGATAAGCGACACTAGTGCCTTCAGATCTTGCAAGGCACGACATTGGTGATATACGACGACCACCCATTTGAAATTCTTCTGGAGTAACGATATGAAATCCCATTGTGTTCAGT

>JAP05447.S|Supercontig_0000076:603735-603913

TTGTAAAATATTTGTTTAGAATCGCTAATCATTGGTTTCGGTAACCAGTTAACCATAGCAGTGGCTTGTAGGAAAAATGTGTATAGAAATATGAAGCACCAACAAATTAATAATATTCCAGGAAAACAAATGACAACTATTCCAATTATCCAAATAAAATCATCTTCTTTTTCATAAGC

>JAP05457.S|Supercontig_0000056:973705-973815

CACCAATTTCAGTATACCTGAGAAATTTTGATATGTTTTAAGATGGTTTATTGTATGGTTCAAAACCGTCACATTAGATCCTCCTAAGTCAATAAGTGATTTGCAAGACTG

>JAP05506.S|Supercontig_0000148:193313-193402

TTGACTCCTTCTGGATGTTGTAGTCAGACAGAGTACGTCCATCTTCCAACTGCTTACCAGCGAAGATCAAACGTTGTTGGTCAGGAGGAA

>JAP05524.S|Supercontig_0000284:277934-278128

TATATGAATCTAGTCTTGATCGAAAAATCACAGGTCGGACAATCGCTAATATATTTCATGGGATATCGACTCCTCACTTCCCTGCAACTACATGGTCATATTGTCGACGATTCTGGAAAGTCCATTTGGATATTGATTGGCGAACTATTAAGAAGATTGCTACCCAAGAGTTACTAATTCATTTTTCATGCACTT

>JAP05536.S|Supercontig_0000053:316946-317097

TGAAATATTCATATCTTTCATAAGCCAGTGTTTCTTCAGTATTATCATGTATATTCAAATGACCAAGAGATGACAAATCTCCATTCTTCTTTACTTGAATATACCAATCGACAAAATCTGCTCCCAACTTGTTCACAAGTATCTTGTCATCC

>JAP05572.S|Supercontig_0000295:432960-433115

TAACCATATCTCATCACATTAGGTAATCCTCTTGGAGGTTCCGCTTTCAATTTTCTTAGTTCTTCATCAATATTTCTTTGAGGTTTTAAATATAAATATGATTCAGACCATGTTGGATAATAGTCAACATCTGGTGCAATAAATTGTCCCCTTGAT

>JAP05578.S|Supercontig_0000012:1024789-1024924

TCTACTTCGAAAAATTGGTTTCTCTGATTCTATGATTGAATCCTTGGAAAAACAGTTTCCTGGGGGTCACAACCACATTAAACGTTTGAAAGTTGCCTTTGAAGCTTGGACTGGCTTTTCTTTTGGGTATGAAGAT

>JAP05608.S|Supercontig_0000661:223792-224038

ATTGCCTTAATCCTTTGCTTTGTAGTGTAGAAGGTAGGGTTAAAACAGTGGTTGCCATAGAAGTGGTTTCAGTTCCTGTTCCACCACCAGAACTATCTTCATCACTTTCTTCACTCTCACTAGGAGCTTCTAAACCAGAATGTAAAGTTAATTGAATAGCTCTTGTTAAACTGACAGCTGGATAATAAATCCCACTTGGTAAATTAGTAAACGCAATTGGACCATGTGGTTGATCATTTAAATAATA

>JAP05654.S|Supercontig_0000179:399309-399395

TACCAATTGAACGACCTAAATCTTTATCTACTGAAGCATTAATGAAACTGGTGAATGATATTGGACGTAAACAAGGTTAAATATAAT

>JAP05681.S|Supercontig_0000192:688114-688543

TTCACCGACGTACTTTAGGTTTGCGTTTAGTTTTTTGCTTCCTGTTGAGGCTAACTTGAGAAGGAGACCAACGATCATCTGATGAGAATGAGTCGATTGACAAGTCATCATCTTCATCAGTACATTGTGATGAACTAACAAAAGATGAATCGTTTGATGGTTCTGAAGTGGAAACGCAAGACATTTCCGAAGTATATAAAGATTGACTAGAAGAGTCACTATCATCGGATATATTTGAAGGTCGGCGTCGACGAGGTCGCTTACGAATAGGACGCTTTTTATTGGATTGAGCAATGACAGAATTTTTCTTAGATTTAACTTTTTTACTAGTACTGCTTGTTGAAGGGAGATTTTCAATTCCTTTTTGATTTTGTTCATTGGATGCTTCAGATGTACTTCTGATACGAGTAGATCGTTCTTGTATTAGGCG

>JAP05707.S|Supercontig_0000143:497520-497667

TTATTATGGTGGTACAGATACTAAACCAGCTGGTCCATGACCCAATTTTTGTGTATTTAATGTAGTTTTTAATGGACGAAAACCAAGTTCTTGAATAGGTATACCAAATTGTTGAAGTTTAGCTTCACATGTTAATAATAGATCATTA

>JAP05716.S|Supercontig_0000087:328802-328911

ATGGTTTGTATTCCATGCATCGTAATACCGGTACTGTTATGGATACTACATCGGCTTTTATATCCAGTCGTGTACTATTTTATTCCAAGATTAAAACCCATTCCACAGCC

>JAP05733.S|Supercontig_0000215:382752-383014

CTCGTAGTTTGTTTGTTGCTTCTAATGGTGGTGGCTCACGTATAGACAATGTCTCTACTGCTGAGACTGATCGGAAAGAGTTTTGGATGTCTGCAGCATCCTTAGGTGACACATCAGCTAGTTTGAAATATGTATCCCTCCGTGCCTGGGAACTGTTTGCTTCTAGAGCATATATACATCAATATGAAAAATATGGATTGACAACAGATATCTTCATGGATTGTGTTGCTACAGTAGAGCAAATTGCACATTGCTATTCTACT

>JAP05825.S|Supercontig_0000265:66724-66795

AGATCAAAAATTTGGTCCCACTTTCGTTTACAAATGAAATCACTCGGAGATTTTATCAAAAAGATTTACGAG

>JAP05867.S|Supercontig_0000142:730100-730223

TTTCCTTGGTAAGAATCTCATCTGATGGACGACAATCACCTTCTGATAATCTATAGGTAGAAAGTTGAACTACTCCATGATAGTGAACAAACGCTGCAGCAATGACGAATACATGAAAAATTTG

>JAP05925.S|Supercontig_0000443:317689-317833

TCATCATCACCATCATCATCAACGTTCGTCATTAGATTCATCGAATATGGAGTCTAATCTTAACAATAATAATAATAGTCCGGGTTCTGTTACCATATATTCAATTTCAGATTCCCAAGGCTCATCTCGACATTCACGTAGATAA

>JAP05990.S|Supercontig_0000221:589179-589268

TAACATTCATGTTCTATGCCTCAAACAATACTGAACATAATCATCAACCAACTTTGCGATACGTTCATCAGTAACCTTATGAGATGGTTT

>JAP06004.S|Supercontig_0000640:192148-192407

TTTTTATTTTCAAGTAGTTTCTTTGAATTAGACCACTCACTAACAGCTAAATACAAGTCGCCATAAAACCGAAGGGCATGGTTTTCAAAGACCGGTGATCCTTTCTATGCCACCCATCCACGGAAGCCAAATTATTGAAAAGGTTACGGTTCATTTTTCTAATTCGTCTCCAATAATAGTAGGAATTTTTATCAACCTCGTTAAGAGGGATAGGACCTCTCTCTGTCCGTAATTTCCAGAGCCAATCCGGATACTCACTA

>JAP06063.S|Supercontig_0000120:166036-166127

ACTTGCATTAACTGATCACCAGGCATGATACGACCATCTAAATCAGCTAACCCACCTGGACTGATACGTGTAATAAATATTCCAGTTGAAGG

>JAP06134.S|Supercontig_0000053:200517-200690

AAGGAAGATGAGATATTTACAAAGAAGCTGGGCAAAGACTTTTTTGAATGGTATACTCTCGTGAAAGAACTCGGTGATTTGGGTGCGTTGGGTCATTTGAATATAAAGGATAATCAAGAGGAAACTTTGGCATTCGAAAGATATGAATATCTTAAGTTTACGTAATAAGGAATA

>JAP06147.S|Supercontig_0000025:537462-537648

GGTTCAGATCCTGAAAGATGTCCGGTTAGTTGGTTAAGTCATGCGGAATTGAGAAAACGTAATTATTCTACAATTAATAGACAACCGAAAATTATCCATGTCAATCATCATATAAAACCGGAAGATAAATATTTTCCAAAACCTGTATCTACCAATAACATTGACCAATCGGATGTTTTACTTAAAC

>JAP06165.S|Supercontig_0000043:1459569-1459696

GGCGACCAAGGATAAACCCTCTGGAAGAAGAAGAACAAGGCCTGCATTCACTAAACTTACAAGATGACTCGTAAGACTATTTTGTAAACTAGCTATTCTTCCTGTACATGGCCCGCGAATTTTAATTT

>JAP06173.S|Supercontig_0000064:2138313-2138403

GACTGCTGTGAAGGAAAATGTGGTTGCGGATCGAGCTGTAACTGCACCTCTGGCACCTGTAAATGTGATGGCAAATGTTCGGGATCCAAAT

>JAP06180.S|Supercontig_0000174:471007-471137

GACTATATGTCATGTTCACCCGTGATCAAATTTGGGACAAGTATAACCTTTTCGTCATTATACATTACTATCTTCGAGTTACCATTCAGATTATTAGCATTCTCATAGTAGCCTGAATTTCTACTGCTTTG

>JAP06188.S|Supercontig_0000124:688252-688376

CAAATCCAGTCTCTGGTTTTCGATCTGGAACAACGCTCTGAGATGACATTTGATGTCATTTGACAAGCAGATGTTGTTGTGCAGCCTTTTGTAACGAATGCCCTCCTCTCAGCAAGGACAGTCCA

>JAP06210.S|Supercontig_0000253:124194-124295

CATTGCAATACGAGCTCTATGTGCCATTAAATAAGATCTTGGATGTACTGGTAAAAATCCGCATAATCCTAATAATTCAGCCACACGAACAGTTGGTTCAGC

>JAP06246.S|Supercontig_0000481:343722-343825

TATGTTTGATCTGGTTTCAATGGAGCCAAATGATAATAATCCATATAAGGATTATTGAATGATGGTGTATATACTTTCCACGAGCTAGACGGTGAAATATTTTC

>JAP06262.S|Supercontig_0000174:157094-157359

ACTCCTATTAAAAATAATCATACATTTTGGAATTCAACAGAATTAACCAATTCCTTATATTTGAATAATACTTTCATTGATAATCATCAACTTGTGAATACTTCAATGAATGGAGATACATCTATCTCTCCTACTAATAACAGCATTAATAACATTAAGAATGAAAATTTAAAATCTAATTTAAGATGGAATTTTTCAACAGAATTAAAAGAATCTAACTGGTTAGTTAATAAACAAATGACTAATTTCTATGATGGACAAAATTC

>JAP06319.S|Supercontig_0000089:711543-711758

TTTTAAATTATCAGAAACTTTTTCACTACGTAGATCAGTAAGTTGTTTACGAAGTTCACTGATCTGTTCAGACAGAGAAGTATTTTCATCAATACTATGCCATAATTCATTTTGTAATTTACGTCTAATCTGACCAGAATCAAGTAAACGTTGACGGAGTCCATTGATGATTTCAGTTGATTCTTCTTGAGCGCGTTTTAATCGTTTAATTCGACG

>JAP06344.S|Supercontig_0000225:644521-644652

ACATTATGTGGCACATTTTGTTTCTTTCTACGATCACATTTATTTATTTGGACAGTTTTTGCACCAAAAATGTTGTATTCTGCTGTGTTCTATTTAACCATGGTCCCTTTTTTATGTATTTGGTCTGTGATA

>JAP06394.S|Supercontig_0000155:262366-262543

CTTACAATGTTTGCAGCTGGTGTAGCATCACTGTTTTTAGAACTGTTGTTGCCTACAAATGATGAGTCAGATAATGACTACTTGACAGATCAAGATGAATATGATGACTCATTAGGTCAGAACACTGCTAGCGAGGCTGATTGGCTAAATGAAAACGGAATTTTGTCAGAAAATGTTG

>JAP06434.S|Supercontig_0000451:58872-58969

CAAACAAAACAGTGGTCTTCAGCTTTCATTTTCCCTTGAAAGTCTCCGACCAGACAAACCTTGCAAAATCTTGTTCCACACCATTTGCACATGTATGT

>JAP06435.S|Supercontig_0000277:240765-240910

TATGGTTTAGTATGATTGATTTACTAATGAAATGTGAATTCGTACAAAATCATCCCGTGTTAAACGCCCAGTTGAGTAATATATTCTATAATTCTTTCTCGTTAGTCACGACTTACTTACCGCCAACTGTGATCGTTTCACACATC

>JAP06502.S|Supercontig_0000474:284535-284657

ACCTTTAGGTTTTTTTCGTGTACTATGACGAAACAATGAAACTGTTCTTTTTACAAGTGGCCTGTCTTCTTCACGCATTGCCATCCATGGATGACTTAAACACTGATCTACTGACGGTCTTTT

>JAP06506.S|Supercontig_0019087:280814-280951

TTTGTGGGAAGGTATAGATATGAGGTGACATTGTACTCGCAGCTCCACTGAGGAAGTTGATTTCCTGTAAAACTTCTGGATCACCCTCTCCAACTAGTGCGAGCCTGATTTCTGAATAGTATGAATATCCAACTATCA

>JAP06525.S|Supercontig_0000010:740206-740371

AGGCTCAACCATACTTTATACCATTTCAATATCATCCGAATGGAACCGCTAATTTCTTAGCAAATTTTAATAATCCATTGGATGCACAAACTGCAGTACGTTATTGTCCAAATGGTAGTTTAAGATCAAATAAATATATCATAGGAGCAGCATGTTTAATACCACC

>JAP06563.S|Supercontig_0000096:974566-974761

AGTACCAATAAAATTGTGATTCTTCGCCCAATGCACAGATCATTGTTATTCTTAGCAATCATTTGTGGACGATTTCGTGTCGTTGAACAGTTAATTCGAATGAGACAATTTGATGTTCTACCAGCAGCAGTTTTAGTGACACTTATTCTTCGTCGACTTCACAAAGAACCTACTTTTCCTCAACAAGTAAGTTATG

>JAP06575.S|Supercontig_0000322:122680-122761

ATTGTTTGAAAGTAGTTTGGGCTAAACGCTGGCTTACGCTCATTTGATTGTAAAGATTCTCCAAATGAAAAGTTTTAATAAT

>JAP06629.S|Supercontig_0000019:1744029-1744097

TAATCAGTATAATCCAGAATCACGTAATAAGGAATAAGCTACATCACCACCCATAAGAGATCGTATAAA

>JAP06632.S|Supercontig_0002049:120448-120624

AGTATAATACGATATTTACAATCAACCGATGGGACTATAAGAGAAAGTTTATTAGCTGATATACTTAATGGTCAACCAGTTGAAACTATCAATGATACCGAATCTACTATTGAATTACCAAGGCACTTATTAGATACAAATTTAGCATATTTAGCCTTATTAAAACAAAATGAATAT

>JAP06633.S|Supercontig_0000461:93435-93553

ACTATAGTTGATGGTAAAATCTGCGATATAACAGGTTCTTGAGCAAGATCAGATGAACTACGAAGACACAATTCATAAAGGCTAGTGTCGAAATTGAAAGTGTAACAGTCAGACAATTC

>JAP06642.S|Supercontig_0000213:248099-248249

AACGTTCTAATGCTGCAGTCCATGAATTAAATGGTGTTAATACACTGTAACCATCTAAACGTGATAATTCATCTCGATGTACTTGACTTGATTGAGCTAATTTACCAATAAGAATTGCTGCATTCCTTTTAGTATCTAATCGTTGACTTTC

>JAP06669.S|Supercontig_0000277:503203-503487

GTATTCAGAACGAAATAAAATGCGTAACGCGTCACCTTATGAATCTGTGAAACTTACTAGTCATGATTTAAACAGTTCCGAAAATTCACCTGATTCCATCGAATCAAGAGTGGAAGCACTTAAACAAGCTAATCATTATTTAAGGATGGAAGTTGAAAATGCATGTAAAATGATAAATTCAAGTGTTTTGAATAAGAATAAAATCGAAGATGAATTTCTGTCCATGCCAAAAGTAAAGTATCCAAATGTAACTCAGTTTGATATTGTAAATCATAAACACAAAGT

>JAP06696.S|Supercontig_0000461:445335-445443

AACTTCTCTAATCATTCCATCTAATAATAGACAAGCATCATTCCATAATTCAAGTTCTTCATCGGTTTCTGCCATAAGCCAAGGTAAAAATCCTTCTTCAATATCTACA

>JAP06742.S|Supercontig_0000344:261230-261468

AATAAACCAGTGATTGAATATTATGGTTCAGACGGTTCTACCGTGATGTCAGTCTCATCTCATATCCCACCTTGTCCAGTTATTGATCCTGTCTTACAAACAACAATCGCTAGTCCATTATTACCAAATCATCATATTGGTATATGGGATTTACATAAAGGTGATGTAATTGATGTCTATGAATTATCACATCGTTTATCTCAATCAGTATCTTGTGCACCTCCAATTATAATCCATCG

>JAP06791.S|Supercontig_0000159:1839688-1839875

GATGGTAAACCATGAATTAATAAAGCACCCAATTGAGATGATACTGGTTGATTAAATTCTAATTGAGCATCATAACGAGATAAAAAATTGAAATGTCCTGGCATCCAATCTTTTAAAGATTCTATACAACCATTTAACCATGTAATCCAAGCATCACGTTCTTGTGGTGTATCAGCACAAAGTAAATG

>JAP06795.S|Supercontig_0000204:273578-273712

AGGATTTTGTTTTAAGGTGCATATAGATAAACATTGGGCAATAATATGACTGGCATTGTTTGTTTTTAATCCTGTTTCGCTTGGTGTACAGCCAAATGCTATTTCTGATAAAAGTTGTGACTCTTTAAGTAACCA

>JAP06838.S|Supercontig_0000308:271946-272274

GTTGTTTGAAGCAAATAGAAACGTGCAGCTGCTTCAGCGGCTTCTCGTTTTTCTGATTCATTACCTGGATGATTTACTGATGCAACAGCTTCTAATTTCTTACGAACAGACGGTGGCATTAAAAGACTACGTAAACCTCTTAACCTATTCTCATCACCAATTTCTGTTGCTGACTTTGGTGGTGTTTCGTCAATTTCTTCATCACCCTGAGTTTTGCTTTCTTTTCTTGACAATTTATCTTGGTTTAATTCTTGTTTACGTTTAACTTCATGATCCATCATTTTTTGTATTTGTTGCCGGTGGATATTCATAACGGAAAATTCTGTAAA

>JAP06883.S|Supercontig_0000096:1775102-1775216

ACTGAGTATCACATTTTAGCAATCTATGCATGAAAGCTCCAGCCTTAGGTGGAGACCCAACATCGATTTTTCGAGTGAATGCCACAGGTTTAAAGGCGAATTCCGGACAGCAGTG

>JAP06903.S|Supercontig_0000480:374368-374468

GAATTAAACAAATTATTGGGTGGAGTTACAATAGCACAAGGTGGAGTTTTGCCAAATATTCAAGCTGTACTTCTTCCTAAGAAAGCTGAAAAACCAAAAAC

>JAP06918.S|Supercontig_0000010:740207-740371

GGCTCAACCATACTTTATACCATTTCAATATCATCCGAATGGAACCGCTAATTTCTTAGCAAATTTTAATAATCCATTGGATGCACAAACTGCAGTACGTTATTGTCCAAATGGTAGTTTAAGATCAAATAAATATATCATAGGAGCAGCATGTTTAATACCACC

>JAP06934.S|Supercontig_0000148:193646-193721

GGATCTTCGCTTTCACATTCTCAATAGTATCACTAGGCTCCACCTCCAACGTGATCGTCTTACCAGTCAACGTCTT

>JAP06936.S|Supercontig_0002065:403533-403735

TTCATCAAATTTGGATTCAGGATTAGAACAATCAGTTATGACATGTTCTGAATCATATGCGCATATGCTTTCACCAATGTCAGAAAAACATTTAGAAAATTCTCTTGGTCACCACTTTTTATATAATAGATATTTATCAAATTCTCAAGTTTTTAAAAATTACTCACATTCACTTTCAAAGTATTCACGTTTTAAAAAACGAT

>JAP06938.S|Supercontig_0000026:446732-446863

CCAATATGCTTTGTCAAACTTTTACTGTGATATCAGAGTAGCTGCAATCGAAGCTGTTGTGGATTACATCAGAGGTGAGCGAGATCAGTCGGCATTAGACTGGCTCTTTAGCAACATTATAGAACGAGAAGA

>JAP06940.S|Supercontig_0000026:446732-446982

CCAATATGCTTTGTCAAACTTTTACTGTGATATCAGAGTAGCTGCAATCGAAGCTGTTGTGGATTACATCAGAGGTGAGCGAGATCAGTCGGCATTAGACTGGCTCTTTAGCAACATTATAGAACGAGAAGACGCCAGTGATGCTTCATATTTTCGCCTTCGCTTTGAAGCTGTCCAGTTATTACTTCACATGCCACCATTCCAACGTGGTGAGTCGGGTAGCCGATTAGATACTCCTCAATTAGTTGAAA

>JAP06970.S|Supercontig_0000126:184199-184332

TTGTATACAGCTTATGGATTATTACCATTCTGCATAAGTTATTGGTTTGTAGCCAGTTCATTGGCATCGGCTATTTTCGGTTTGTATAGTTTCATTATTTGTTCTTATTTATTAATTCGTGTAGATATTGTCAC

>JAP06995.S|Supercontig_0000018:1856513-1856868

TAATATGAATTTGATTAATGAATTATCTGTGAGTTCTCACTACTACTTGTTATTTTTCTTCATTACAAAAATGTGTAAATAGTACTTTTGACCTCCCAGAATGCTTTTTTAAATTCATTCTGTATTAATTTTACTGTCTACTTCTCACTTCAATTTTCCCCAATGGTGAGATCATGTGATTTTTTGCCATTTTCTGTATATTTGTACAGGCCATTGTATATCTATACGTGGCTATCCCAATTTTTGTTGAGCTAGATTATACACACATTCACACTGTCAAACCTTATATTAATGTATTACTACTGATGTAATTTTGTTTTCTTTAATAAGAAATTTCTTTTTAAATATGAATTCAC

>JAP07055.S|Supercontig_0000295:853308-853461

ATAAATGAAGAGTATCAAAAGATAATGGAAGAACGTAGGCTAGAGGATGAAAGACGACAAAGAGAGGAAGAACAAAGACGTCAAATGGAACAAGCCGTTACAACAATACAAGCATTTTGGAGGTCATATAAAACAAGAAAACTGGCACGTGGAA

>JAP07125.S|Supercontig_0000312:1402807-1403071

TATACGCAGTTCACCTGGTGTTCAGTTGAAATGGGGTGATGAAAATGATGTGAAAATCACACAGAAATGGAATCCGTTATGTCGTAGTTTGCCTTATGCTACATTAACTCATCATCCTCTCTATTCTAAACGTGATCAATTGCCATCAAAACAAGTAAATGTACCAGGTGCCGGTTATGAAGATTTATCTAATCCAGTATCTGTATACGATGTGAATAATTTTCTTACAGCACTACGTGAAGCTTGTTCTGGTATGAATATTCAG

>JAP07162.S|Supercontig_0000648:224729-224876

AAGATGCCCTTTTACCACCAAATGAACCACAACGCGCTAAGAAACCAGCGTCAATGTCTAAAATAGATAGTTATAGTCGATTCGTTTTTCCAGCATGTTTTTTACTTTACAATTGTTTCTATTGGCTTTATTATTTAGTGATTGTTAA

>JAP07186.S|Supercontig_0000025:765854-766041

AAGTGATATCTTCCAAGACAAATGAAACGATCAATAAAATATGTTTGGAACCAATTGAATCAGGTCCATGCAGAGCTTCCATCGAAATGTTCGCTTTTAGTGTAGACCAATGGAGGTGTATCAAATTCATTTATGGTGGTTGCCAGGGGAATTCTAACCAGTTCGCTAGCGTAGAAGAGTGTGAAGCT

>JAP07269.S|Supercontig_0000195:832918-833033

GTACTTGATTTATTTGATAATGAAAAATTAAATGTTTTTCTAATAGCTAATTGAGTTTCCGGTAATGAATGTCGACGTATAACACGTGTTGTAATAAATATTGTTTGATGTAATTT

>JAP07297.S|Supercontig_0000214:194414-194562

CTACTTGGAATTGGCGCATCGTCAGTGTACCAAATTTGCCAATCTTGTAAGCCTAAATGTACAACTGCCAATCTGATGTACTGCTGAAATGTGCAGCAAATTTTGTGAATACTCAAATCATTATTTAAAATAAAAACACCCTTTTGATT

>JAP07331.S|Supercontig_0000290:479336-479530

GTTTATCTCTTTCAGTCTCTCTGTGGTGAGAGATGGTAATATTGCTGGGCTAGTGATACACTTTTTTTTGTTTATTGTAAAAGGTGTGTTCGCATTGAGTGAACGACGTCCCGAACTTGTTGTAACTTTTGAAACAGCACGTCTTAATAAAGAAAATGGTTTTAAACTTAATCGACCTTTAAGAGCACTTTTGGA

>JAP07371.S|Supercontig_0000167:367362-367588

TTTGATGGAATAGGGGAATTATCGAAAAAGAAGCTAGCTGATACAATTTGTTCATCAGGATTATTGGAAATGCGAGGTTTTGGATTTTTTATTGTTCCCCATACCCAAATCCAATATAACGCTCCCATAATAAAAAATACAGCGCCAAATTCAATTTTTATGGCTATTAACCATAATAACAGAGTAATAATGAATTGTACATACCATAAATTCCAAATGGCAGTAAT

>JAP07445.S|Supercontig_0000237:306193-306304

CTCATACTCTTGACGATGGCCGTTAACTCTTCCCTTTTCTTCTTAGCTCTTCTGTGTCCACCAATGCGACGCTTAGCGAATTTGAGTGCCCTTTTTTCCTTATCGTTTTTCA

>JAP07469.S|Supercontig_0000121:1249429-1249531

TGAAAGACAACGTGAAAAGCTACTTAAAAAACTTTCAAAGCTTTCAGATAAGTCCTCAAAGCAAAGTCATAAATCTAAGCATAAACATTCAAGGAGACATTAA

>JAP07477.S|Supercontig_0000262:523535-523807

TCATTCACTACCGGATATTTTGAAAGTTTTCGAAAAATTCGTTCATGTTCAAAAACGCCTAGAGTATACAGAATTCGAAGTATATTATCAAGCTGTTTCGACTACTTTAGGAGAATGTCCTGTTGATCAGTTGAGATCAGATGCAGCATATATGGATTTATGTTCATTATTTCCATCAAGTGAGTGCGCTCAAAATTATTTTCTAGGTGAAAACATAAATGATTCCTGTGATACATTTATAAAATTAATACAAGATACATGGTGCATTTGATT

>JAP07526.S|Supercontig_0000096:851470-851622

GAACAACCAGGTGGACCAGCACCAACCCCAGCCGATAAAACACTAACCCGAATTATTTCAACAATAAATCATGAAAATCTGGATTATTTTCAAGAATCATTTAATAAATGTCAATTAACAATAGAAGATATTCTTGAAATTCTTGAACATCTT

>JAP07587.S|Supercontig_0000195:289064-289172

GATACTTACCATCTTTACCATCTGGTCTACTGCGTACTATTTTCAGAGAGTTTGACCAAGATTGTGACGGTCGAGTTTCATATGAAGATTATCTACATACTACAAAGTT

>JAP07619.S|Supercontig_0000096:1423614-1423779

GTTGGGAAATAAGTGATAAATGGCTTTACTGTATTGATATTTTGCCAATCGTAGAACTAAAGAATGAAACGCTTCATAAATATCGAGTGAAATTGAGTATACCATCTAGGCGTTCACCCATACCAAAATCTACAGTGTCGGTTTACTTCACTCTACGAATATCTCG

>JAP07664.S|Supercontig_0000032:1080697-1080820

ATGCCACTTTGGGCTATAACAGTTGGATGGTATTGGTTTAATCGTCCAATTAATATTGGGAAAACATTTGGCTGGCTAATGCTTATAGCTTCAGCACAAAGTATAGGAACTTTAATGAGAGGAT

>JAP07704.S|Supercontig_0000021:2302557-2302675

TATGGGATTTTGTATTTGCACCAAATAATGGTCCACAAGCACATTGTGTTACATCACAAGATTTATCAAAACCAGAAATCTATAAAAATCAATATTTATCAAGTAATCTAATGAAATGT

>JAP07707.S|Supercontig_0000478:99091-99167

AGATGGGTAGAAAGAGAAGAACCTAGCGGTCAATCAACTAGATCACATCATTTTGTTGAACGATCTATTGAAGGTAA

>JAP07709.S|Supercontig_0000655:84396-84480

GTCCTTTACGGATTGTACGTGCGCTTGATTGCGCGCGAACTGCTGTCACAACTAGTTTACCAGTTACTGGAGATTTAGTGATATT

>JAP07710.S|Supercontig_0000283:323631-323836

GACCAATTGCTAATTGAATCGGAGTATCACCGAAATGTTTCAATTGATTCAATCCATACTGTTGACGTCTTTCACGATTGTAATGCCAAGCACATAGTAGTTTTGCACAGGACATACGAAAATTATTATTTAATAATGAATATACCAGAGAATTTAGGCAGGCATTGAAATAGCCAAGCCATGTTGCAGCAAAAATAGCTTCTTCT

>JAP07926.S|Supercontig_0000213:453428-453528

AACAAAACATTTTGGACCTACTGTATGGTGATATAGTTGCACTAAATACATCTTTTAATCCAACTAACCATGCTGGTAATGGAATTGATATTTGTTTAAGT

>JAP07940.S|Supercontig_0000131:333769-333934

GTTGGATCAGTAACAGAACTGACAAGTGGTATTTATTCCTTGGCCTTCATTGAGCCAAAAACAAATGATAGCAGTAACAAACGTATGGTACAATTTGCATTTGCCTCTGGTACCCGTGTTTACATTGCACAATATGAACAAAAATAAAATTGCGGAGATTTATTTT

>JAP08075.S|Supercontig_0000015:1410049-1410235

AAGAAGTTGCATCAGCAAGTGTAATGATTAAAAAATATGTAAAATTGGATCCAGATGATGAGTTCAATTCACAAACATTAATTAAACGACATGGTGAACAACTAAAAAATAAACATAAACCTTCAAAATGGTGTCGATTGCATATAACAAATGATGAATTATGGTTATCTGGTGCTTATTGGTGGCC

>JAP08139.S|Supercontig_0000018:567988-568108

TTATAGTGATATTTTGTCCAGTACAATGGTAAGAAAATACAGAAGACCAGTTATTTTACTATCTTTAAAGGTTTGCCAACAAGATTTGGGATTCTTTAGGTCCGTTTTCCGTATCTGAAAA

>JAP08149.S|Supercontig_0000108:135423-135559

TGGAATGGCGCACTTTGGCTATTGTTTTAGATCGTTTATTTTTCTTTATTTACATTACCACCATAGTAATTGCAGTAGTTGTCTCAGTACCGCGAAGTACTGAACCAGAAGTTCCACCAGCATTTCGCGATGATTAA

>JAP08201.S|Supercontig_0000586:30114-30222

CATTATTAATTGTTCCAGAACAAGAAGATTTTCGAGCTGCAAGTCGTTCTAATGCTAGACGTTTATTTCTTTCAGCTCTTGACTCAAAGTCAAATGAAGTAGATGGTTG

>JAP08232.S|Supercontig_0001915:32228-32473

AGGACGACCGGTTGGATCAATAAACAAGTGTCTACACAATGGACAAGCAGGGTTTCGACTAGTTGTAAACCACTTGTACATACAAGCATAATGAAATAGTTTACGACACGTGTGACATCGCATTTTAGGCAAACTGAAATTTGTGTTGTGAACAATCGAGTAACAAATTGCACATTCTTCGACACCATCGAATTTTTTCCGAACATTACTTCGCCACAAATCGATACCGTCTAAAATTGATCCATT

>JAP08263.S|Supercontig_0000106:811161-811339

AAACATCATGGATGCCAGAATGGGATCAGCAAGCTAAACAGAGTATACCAGCAGCTGCAAATTTATTCGCCTATCATATGTTGGGAAATTACATTGTAATCGTTCTAATCACTATAACAGTCATGATTCGTACAAAAGTTAGTTTGATGAGTGATAAAACAATGAGAACACGTTTATCA

>JAP08324.S|Supercontig_0000259:83950-84057

TCCCTTTAACTCTATCTGCTTATATAATGTATGTGTGTAAAGGTCAAGCCTCCAAGTATGCTGGTGTAAAAAAAGTGTCATATTTGACTCTATGCACTAGTATGTCAT

>JAP08348.S|Supercontig_0000306:113385-113580

ACAAATTGAATCTGGTGAATATACATTTGAAGAACTTGCCCGAACTGAAAGCGATTGTAGTTCAGCTCACTCTGGCGGTGATTTAAATTTCTTTAGTCGTGGTCAAATGCAAAAGCCATTTGAAGATGCTGCATTTAAATTAGAAATTGGTGAAATGTGTGGTCCTGTATATACAGATTCTGGAATTCATTTAATC

>JAP08392.S|Supercontig_0000231:59416-59597

TCTCTAATCATCTAATTGACATAAAGCAGCTACACCACGATTAATCCAATGCTCTTGTGGTTGATTTGTTTTCATATCAATATATAATACAAAATTTGTTGAATGTCCTGTTGTTGATAGAACACCACGTCGAGCAGATGTTTTGTAGACTGGGCAAGTATAGGTTGATTCACGATTGATTT

>JAP08438.S|Supercontig_0000122:769721-769874

GATTTTTATGCGCATTTCTTTATGTAATATTTTCAATATTTCGTGCATGTTTTGAAGGATGTATGAAATTTACTGATATGTTACTTAAAGGTGCTGAATTACCATTAGTTTGTATGAGAAAAACAATGGCTGGGGAGAAAATGTGTTAATAACT

>JAP08450.S|Supercontig_0000094:492985-493141

TTCAAAATCATCTAATTGTGCACGTAATTGATCATTATCTAATCGTTGTGTATTATTTTCACGTTGTACATTAGCTTTTTCTGTTTCACTTCGAGATAATGCATCACGTATTCTTGATATTTCTGTAGATAGTGTTTCAATTTCTGCACGAGACATT

>JAP08511.S|Supercontig_0000265:79332-79424

CTTCCAGTTCTACAGGAAGATTTGAAGTGGTATCTGTAGAATGTCAATCAGAATCTACATATGATCCAGAACAAGATGATAAGGGCGAAAACA

>JAP08659.S|Supercontig_0000284:248215-248522

TGGTGAACCGCTACGTTTACATGAAGCCGATGTTTGACGATTTGTATTTCCTGATAAGGTAACTTTTTTACTTGATACAGCCAAATCAGTAGCTGTTGATCTTGGAATAGTTTGATAAGATTTAAAACGTAAATTAGGCAATATAATTCCATTTTTACGAACATAATCACATAGAGATGATTTTGCTAAGTTATACTTTTGTGCAGCACGTCTCATTGATATCTGAGAACCTAATACATCATTTACGGCTAATTCCAATAATTCCCGTCGTTCTGCAGGTTTATGACGAGGACGTCGGACAGAAGTAA

>JAP08785.S|Supercontig_0000488:464959-465141

ATAACAAGCTATTGAATTAGATATCCAATGTGAAGCACGAATAATCCACACATGGGGATTGCAGTCACGAAACTGATTGGTAAATCGTACATACAAATTTTGGGCAGTCTTTTCCAAATTCCATTTAATATACTGATTATTGAAATTATTTTGACTCATATTATATTCGTAATCCTAAAAATA

>JAP08811.S|Supercontig_0000070:245082-245165

AACTCACGGCTTCTGTTGAAGGAAAACTACCACACTTTAGTTTTGAAAAAATAAGATTTCCATTAACGGTAATTTCAAAAGATC

>JAP08824.S|Supercontig_0000104:441400-441506

GCATAATCACGAATTTTTTTGACACGTTCAACAGCATCCATTGAATGAATTGCTTGATTGAAATGATCTTTTACTGCATTTTCTGTTGTATGTAACATTAAATTTCT

>JAP08831.S|Supercontig_0000663:299538-299683

ACATTTGTTCCCTCTACAAGTAATGAATATCAAAGATATGCTCAATTATGTCGAAGTCCTGCATTTTGGTTCACATTCCCATTAACTGTTTTTATAGCCAATGCTCCAGGATTATTATGGCGTTTAGCTTCAGATACATGGTGGAC

>JAP08901.S|Supercontig_0000307:69886-69959

TCATATTCAATAGCTCCAGTCGAATGGAATGATGATGATCTGCAACAGTTAATCCATGAAGCAATAACTAATCC

>JAP08946.S|Supercontig_0000310:291809-291872

CATTATTGTTATGAGTTGCAGGCCAACGATGAGACCACCAAACGAGAAACTGATAAAAACGAGG

>JAP08958.S|Supercontig_0000462:234256-234347

ATTTCAAACTGTGATTTATAAATAGTTCTCACTGATTTTATTCTTTTTGTACATTCAATACCTTCCAGCTGCCTGAATCAATCAGCATTTAC

>JAP08969.S|Supercontig_0000234:154524-154695

ATGCCAAACCGCAATTAGAATAATTCATTACTTCATATGTTCGATTCTTAGTCATAACTAATGGCTGAGTGATCAAGTCACGTACAACAACAGCTGGATGACGTCGTTTAGTGGATGAACCAAAACGTACACCAATTACGCCCATTAGTATAATAGCTATAGCCTTGTAAAT

>JAP08978.S|Supercontig_0000441:11868-12011

ACGGAGCTCTTCTAGTGTACATTTAAGTAGGTCTTCATAGGTGCAGTTATCCGGTAGTACATCTAAGGCTCGGTCAAGCTGAATATCTTGTAGAAAATGGATGAGTCGCAATCGATCGCTGGCAGCCCACCTATGTGAAGGGCT

>JAP09010.S|Supercontig_0000305:801160-801371

ATCAGTGGATGCATACTGGTCCAATGTGTCAGATTATCTATAAGATATAATTCATTTACACGATTTAAATAATGTTTACTTGAAGAAATGTTCAAGTGTATCACATGTGTATGACTGTAATTTTCTTCAATTAATTGATAGACTAAGTTTATTAAAGATGTACTACCTGTTTTAGGGATTCTGTTGTAAACAACAACTGGATATGACTTGAT

>JAP09024.S|Supercontig_0000067:491166-491290

TGATTTGTACCAAGTGTTGACCGAGCATCGTAGATAGCAAAATCAGAAAATAGTTTATTTCTTTTTAAATGTCCACTTAGCTTAGCTTGTTCTAGTGCCAATGTCAACCCAGACTTTTCTGATTT

>JAP09074.S|Supercontig_0000087:599398-599515

TCATTCGCAGGCCACTCGAAAACTTGCCGTTGCCGAAGTCGCACTATCTCACGCTGAAGATCGGATTGAAGCTGCAGAAAGGTACTTCTTCTTAGATTTATTGGTGAAACTGGTGATT

>JAP09126.S|Supercontig_0000091:1728421-1728515

GTACTTCCAAATTCCTATGAGCCTAGAGAGGCAAGGCATTATACAAACCTGAATACAAAGTAGAATTTTCAGCTTCTTCTTTAATTCAGAGGCCT

>JAP09135.S|Supercontig_0000106:168436-168642

TTAATGTTGATCAACAATTGGCAGATTTACTGAAATCAACTCCAGGACAGTTTACTCAATGGTTAAATAATAATCTATTGAATTCTTCTTCATCTAAATCGAAAACAAATGATAAAAAAGTTGAAAAATATACTGTACCTAATAATATTTTATTCACTAAATCAGATATACAACTTTATGAAGAAATCTATTCACATTTAATAACAA

>JAP09175.S|Supercontig_0000239:250179-250393

ATATTTCACTGATAAAAAGATATGTACAAACAAAATTAAATTTTAGTAACGAGATGGAGGAGCAAAATCTGCAATTCGCTTATCACGTTCTTCTTGAATGTAACGCTGTGCTGAAATCATTGACTTTCCTTTATCCCATTCTCTTTGTTTACTACCGTAGGATGGACGGCTTGGATTTTGAGATGCCAGTACGTCAGCTAAATTTGCTCTATGTT

>JAP09193.S|Supercontig_0000021:2665100-2665569

ATCAAACATAGAATCAAGCATTCACCATTCACTGAATGCAGATATCGTACTGCATACATATCAACCTACACAGCCTTTGCCTTTTTGATCATAGCTTTTGTCACGCCATATTGGTTACAATCATGGCCTAGACTTCATACACCTTTTGTCCGCTTGGGACTGTGGGAATTTTGTGTCAATGGTTTTGTGCAACGTCTAGATCCCAATATGGTTTCTTACTACGGTTGCTGGTGGATTCTCTCTCCATATATGCGAAAAATATTCACGGATTTGGTTCCATGTACGTTTAATTTACCTAAATGTTCATCGTGATTAATTTTAGTTTGGTTTTTGATCATCCAAGTTATGGTGACAGTATCTCTATGTCTACAAATAGTGGCAGTGGTCACTCTTATCGCCTACATGTGTAAAAGGATAAGACATGTGGAACGTCAGTCGTTCTTTATCTCGCTCATTACATTCTGTCATTC

>JAP09215.S|Supercontig_0000032:1435415-1435565

GAGAATGATCCAATTATGCATCCTACTCCTGGTGTCTCACGACCTATGTCCCAGTTAACTGAAGAGGAGCAAGTGAGAATCGCTACACGTATGGGCTTGATTTCCACACTACCATTGTTTAAATTCGATGAATCGAAGAGAGAGAAACTAT

>JAP09250.S|Supercontig_0000102:366490-366749

TATTCTTTTACAATTTTTAGTGAACGGGGTTCACAAGACGACACTTTCTCAATAGCTTGTTTTTCCACTGATTTTTTTAACAATTTTTCCATATGCTTCTTCATTTCATGAGCTTCTTTTAGTCTTTCTTTTTTTGGTTTTATAACGCGAGTCCCTTTGCGCATTGGTTTGATGGATTGTTTTTGTAAAGACCTTTCACTTTTACCGGATTTCACTCCTTTGGGTAATTTGGTTTTGACTTTAAGTTTTCCTTGAGCCAT

>JAP09287.S|Supercontig_0000044:1846385-1846594

TATGAAGAGATTCAGAATTCATGAAAAATCAATACAAATTAAAAAAGTAACTATGGATGAAAAAATGCAAATGGACGTTATAAGCACTGTCGTTGCAGCATTACATATGTATGGAAATGAATATACACAAATGAAAAAATTCATAGAAAGCAAACTGAATAAACTTTATCTTCCTACGTGGCATTACATAATTGGTTATGATGTGGATAG

>JAP09294.S|Supercontig_0000032:1149640-1149768

GAATGTAGCCAATTGAATATTGCACGTAAATCATCAAAGTGATTTGCTTCAATCATTCGATCAGCTTCATCGACTACAACTACATTGACACTGTGTAATGTCAGAAGATGAGGTTCACCCTTGGCAAAA

>JAP09295.S|Supercontig_0000491:367477-367660

AAGTTCGGCTTTAGCATTATTAAGTTCCACAATAGTAAGTTGATGAGCCTCTAGTGTTTGTTGATGGGCGACTTTTTCACGTTCAGTATTAAGCGTCTGTATTTCTGCTTCTTTCTGATATGTTGTTAAGTTAGCAACAGCTTGGGCTAAATCATTTTGCAAGACCGAAACATGTTCGGAAAGA

>JAP09304.S|Supercontig_0000684:52642-52789

CAGGATCCATGCTTGGTGGAAGTATATACGTACTTACCGGTGCTGTAATGAATAGACGAACTGGGCCTGCAGTATTTTTGGCGTATATATTGGCTAGTTTTGTGGCATTACTTAATTCTCTAAACTATTCTGAATTAGCATGTCGTAT

>JAP09337.S|Supercontig_0000153:163623-163831

TTAATTCATCCACATGGATGTGTTGGCCAGTTGATGCGAAGTCCATTAATAAAATTCATTAATCATAGCGCATCATTTGCAATTTTTATATTCTTACTTTTGATTGCATCAACTGATACATTAACGAAAACAGATTTACAAAGACGTAGTGAAATTCGTGGACCAGATCCAAATGTTATTGAAATGTTAATATTATGGTGGGTTATTGG

>JAP09408.S|Supercontig_0000159:1841744-1841860

CATAAAACCACGAGCTAATACTTTCGCTTCCAAACTAACAAAAGCATCGACAAGACCAGCAGTTCCAGCTAGTGGAGTAGAACGAGGTAAATGTAATAAAAGTAGAGGTAAACGATT

>JAP09559.S|Supercontig_0001624:1679-1780

TATAAACCTCAGTCTATTAATCCGGTGCCTTTGGATTTTATAGGTATTTGGTCCTTCCTATGGTTGATTGCATTTTGCTTACTTTGCAACCGCTGGCAAAAT

>JAP09624.S|Supercontig_0000474:255514-255611

AGACTCGAGGGTCAGTATTCTTTGTGATTGGTGTGCTATGTTTGGTATTTTTCGACCATGACTATAGTCATGGGGAGACGGAGCATCGTATCCTTTTC

>JAP09692.S|Supercontig_0000633:194172-194378

AACAAAATCGATGGGACGCTGAATAATTAACTTTGAAAGATAACGAAGTAGAATTGTTGTGCACAGACGATACAGGGCACTGATGACCTGATTGAAAAGGATCAAATAATGAAAAACGAACACACAGAAACACGTACAGAGAGTAAAACATTAAGACGTAAAAGAGACTAAATATACCGTGAAATATTATCAAGTAACAATCAACAA

>JAP09829.S|Supercontig_0000456:51679-51812

TGGTATATCCAATTTTACATCACCCTTAATTTCGGATTTTACTAAAGAAATTGAATTACCACGTTGATTATTCAATGATTGAATAAGCACTCGACTATGACCAAGGAAATGAAGTTGAAATTCATATTTCAAAT

>JAP09868.S|Supercontig_0000093:1804344-1804479

AATGTTTATGTAATATTTAGGCCAAAGAGGCAGGGATTTCTACACTGGTCATGTTAGATGATCAAGGCGGTAAGGTTACGCATATATGTTGTGACACAATTTGTAAATTTCATGTTGTAAAGTTGTTGTCTAGACT

>JAP09922.S|Supercontig_0000101:504512-504908

TGAAATCACACCATCTGCTCCCAACCTTGGGTCTTTATTCGTTGATTGGCTATACGATTATGATTATCCATCTCTGCTGTCAAATCGGCCGTTTTTCTTCGTATTTCGCTGTTCTTGTTCAATCTTGTTGCACATTTGCATCTGTTGTGCGACTGCACGTGATATTAAATTCGGATTCATTATTTTAACCTTCATCTTCGTTAAGAGGGTTTATCCGGATGCTCTTTTTACACTTTTTGGTAGGTTTATGTCTTTTCTTGTTCTACCGTTACCCATCACAGATTTTCTTGTCTATAAACATTACTTATTTATTCTCTTGTTCCGTATTAATTCTGTTTTCATTCCTTATATTGTCTTGACAGCAGCACAACTGGTTTTTCTTTGTTCATTCTCATGA

>JAP09995.S|Supercontig_0000107:485461-485643

TGTAAATGTCCTAATATTTGTTATAGTATTCTATGGTTTTTTATTTTAATATTTATTGCATGGCCATTATCATTCTTTATATCAATCATTTACTTAATATTAATTGTGTTCAGTGTTTGTTGCCCTGTTTTACAACCATTAACAGATGGTATAAGTAAAGTGATGATGTTTCCGGTAATGTGT

>JAP09998.S|Supercontig_0000187:813273-813414

GCATGTTTACGTTTATCTAATCGTGGTGTTTGTACAGATTCATGTCCAGCATCAAAAAAATATAACAAATCAACATTTAGCTGGGTAGAAAATCCAGATGGATTATTAACATTTGGAATGATCTGTGTTAAATCTTGTCCAA

>JAP09999.S|Supercontig_0000441:190311-190567

TTGCTTGCTGGACTGCCATGTTCTACTCCTGAAACAAATAAATTTTCTACTTCCCCTTTAATAACTGCTTGTCGAAGAACAATACCTGACCTTCGATATCCAAATCGAGGTTGATAATCAGGTAACTGGATAGTAAATATACCTGTACCGTACGGTAAATTTAAACCAGAGATTCTTTTTTTAAGTTGATCCATTGAAAATGGAGGTAAGCCTGTTTGAACTTCACTAATGTTTGCATAAGGTATAAAACTGTCCAA

>JAP10027.S|Supercontig_0000160:223897-224188

CAGCACTAGCTGAAACAAATAATATCATTAATTTAATTGTTGTACATACTGGTCCAGGTATCCATTTACTATTACAAGCACCAAATAAATCACATAGAAATATTAAACCTAATCCACCAACACCAGCTGATAATAATATAAGTACAATAATAATAGGGATTGTTTTAAAACATTGAGTATATAAATTTCCACATGGCCAATCAGAAGTAGCTAATGAATAACCTAATAAACCAATTGATAATGCAAGTAATGGAAAATGTAATGATGTGGATTAAATAATCTTCCAGGCATT

>JAP10037.S|Supercontig_0000291:612935-613175

TTATCATCACCTTCTTCATATGGAAATAATCTAGCACGTGTAGTCCATAAATAAACAGGACGTGAATGTCGATGAGACAGGCGGCCAGTTAAACCAAATAATCGAACAAGCACTGAACCAAGTATATGGTTAAATGAATGTGATGTTGGACTAGTTGTTGGTTGAAAATTAATTGTAACAGCTTGATTTTCAGATTGCACATGACACTGACTTGTTTGTAAATGTCTTGGATGTACTAATT

>JAP10261.S|Supercontig_0000075:237827-237962

AGGAACTAATTACGATGGTTAAAGATCTCTCTGAACAATTGTTTGAATCGAAAGGTCAGTTGGTTGAACAAGAAGCACGTTTAAGGGCAGAAATGTGTGATAATATGAATCAGCAGATCATTGATTTTGAAAATAA

>JAP10277.S|Supercontig_0010511:1043-1189

TCCTGTGTGAAATTGTTATCCGCCTCACAATTCCACACAACATACGAGCCGGAAGCATAAAGTGTAAAGCCCTGGGGTGCCTAATGAGCTGAGCTAACTCACATTAATTGCGTTGCGCCTCACTGCCCGCTTTCCAGTCGGGAAACC

>JAP10349.S|Supercontig_0000148:175020-175102

GTTGGAGGTGGAGCCTAGTGATACTATTGAGAATGTGAAAGCGAAGATCCAAGACAAAGAAGGTATTCCTCCTGACCAACAAC

>JAP10398.S|Supercontig_0000020:927358-927459

AGAATATCTTACTGGATTTCGAAAAAGAAAGCTTGAACGTAAAGAAAGGGGACGCAAAGCAGCAGAAAAACAATTAAAAGATGAGATTAAGGCTGTTAAGGA

>JAP10526.S|Supercontig_0000085:614322-614471

CGTAACTGTGGCATATAACTTCGTAAGGCTGAACCAAGATCTGGGACATCTGACACTAGGAGGCATTTCTCAGCAACATAACTCAGAAAACATTCAATTCTTCTAGGATCTTTTGCCAAGTCTGATAGACCAGATGTTGAAATCTAGAAA

>JAP10569.S|Supercontig_0000285:170622-170843

ATGTGAAAAATTCTGTTGCATTATATGGACTTAAACTTGTTGAATATGACCAATGCATTGTATCAACAGCAAATAACAAATGGCCATGATAAAGTAAACGTATTTTATCCCACCAACCTAATGGCGGATAAGATTTGTCTTTTATTAAGGGTTTAATATCATCTAAACGCTGATTAAACCATGAGACAGTTGGTTCCCAATTAGCACCATAATATATATGAA

>JAP10697.S|Supercontig_0000159:1537275-1537420

TATTTGATTGGCGCATGAATATCATCAGTTATAAACTGATTCAAAAATCCTTGATCTTTTGACCAAAATAATCGAATATCTGGTATCTCATATAATCGCATAGCCCAACGTTCTAAACCAAGACCAAAAGCATAACCAACTTTATT

>JAP10716.S|Supercontig_0000678:202862-203024

AATGATAAAGAACCAATAGTATGGCGTTTTGCTGCACATCGTTTAATATTAGCTGCAGCATCACCAGTATTTGAAGCAATGTTTTATGGACCAATGGCTGATTGTGATAATAAAAATTTAGAAAATCGTCGAGAATATCATATACCAGATATACATCCAAAGG

>JAP10808.S|Supercontig_0000310:532219-532362

TAATAAGTTGATTAAGAAGAGCTTTTGCACAATGTGGTTTTATACCCAACTTTGCTTGTCCCATTGCCCATTTGTCACATGCTCTGATCATTGGAAATATGAGATGATGAACTATGAAATCTTTTAATCTAAAACTTATAGTTG

>JAP10953.S|Supercontig_0000026:1047042-1047141

AATAACACCGTTTCGTCCTAGTTTCAGATAAGTATCAGGTTGGGGTATGTTGGACGGAACTAAATAGTTTGTATTAGATTGACCTGGAATTAATTAACAT

>JAP11010.S|Supercontig_0000159:903443-903535

TCAATTGTAATCATCTTTTAATTTACGAGGTAGACCAGATAATGAACTTGTGAATGCTGGTTTACGTTTACGTGCACCAACATTAACTGTACT

>JAP11118.S|Supercontig_0000304:599331-599398

ATTGAAACTGTACAATTATTCACAGCCTTGATGTACTTGGGACCATATTTGTATGATGTATACATACA

>JAP11450.S|Supercontig_0000064:2138312-2138403

AGACTGCTGTGAAGGAAAATGTGGTTGCGGATCGAGCTGTAACTGCACCTCTGGCACCTGTAAATGTGATGGCAAATGTTCGGGATCCAAAT

>JAP11495.S|Supercontig_0000685:525464-525611

TGATTACAAAATCTACATCTGTCTTTTCAGGAAGAACATCCAGTTCTGCATGACGTTTGACATTTAAATAGCTTTCTTTGTCTCCTAGCTTAATTTCATCTTCAGAAAACATGGACAATAAGAGTTCCACTTCGTCGATTTTACTTTT

>JAP11496.S|Supercontig_0000685:525464-525611

TGATTACAAAATCTACATCTGTCTTTTCAGGAAGAACATCCAGTTCTGCATGACGTTTGACATTTAAATAGCTTTCTTTGTCTCCTAGCTTAATTTCATCTTCAGAAAACATGGACAATAAGAGTTCCACTTCGTCGATTTTACTTTT

>JAP11498.S|Supercontig_0000674:80881-81326

GAAATCACAATATTACTGATATTCATATCAATAGGTAGACGTACCGCATCCGATTTTGATGTATCAAAAAAATCTGAAGACCATAGAGAATTTGTTGAATTATAAAAATTTGAATATAATAAATCCATATCATTTGCTGAATCAGCTGGCCACACATTATGTACACTAGGTGGCATGATTGGATCAATAACTTTAACATAAAACCAAGTGGTTGTTTGTAATGATGGGTTACCATTATCAATCAACTGTGCACAAAGATGATAAATTCCACGTCTAGCATCTGACTCAATTAACTCAGGAGTTGATAATTTCTGATAATCAATAAAACGTGCACGAAACTCCCCAGAAAATTCATCTAAATGAAAATATTTTGAACCATTACAACTAGTATTTTGTTGATATGTAATACGTCCATTTTCACCAGCATCTAAATCATTCGCTTGTAT

>JAP11564.S|Supercontig_0018031:26-114

CGGCCGCATGCATAAGCTTGCTCGAGTCTATAGTCGACTGGGCCGAGGCGGCCGACATGTTTTTTTTTTTTTTTTTTTTTTTTTTTTTT

>JAP11565.S|Supercontig_0018031:11-110

AGTGAGCTCGAATTGCGGCCGCATGCATAAGCTTGCTCGAGTCTATAGTCGACTGGGCCGAGGCGGCCGACATGTTTTTTTTTTTTTTTTTTTTTTTTTT

>JAP11649.S|Supercontig_0000009:2188248-2188398

TTAAATGTTTCATTTCATTTGCAATTAATAACGCCACATTACGTCCACCGATTATAGCCATTTCCATACATGAAGCAGTTGATTCAATTGCATTTGGATAGTAAACTCTTGATGCCAATTTGAACTGACCCAAGTCAGTATATGGATCATT

>JAP11651.S|Supercontig_0000155:962573-962747

ATTTCATTAGTATGCATATTATAAACGATTATGTCAAGCTGCACTTTTAATCCAAAATCAATATCGTTGGTACGTAAATCAAAAGAAGAATGGTAATAGCGGGGGAATTTCAGCTGGTCCTAAACTTAGAAAACCTAGGTCAAGTCAATGTAACAATCCACGATACAGGTTTGTT

>JAP11657.S|Supercontig_0000371:25004-25226

GTTGTGACTGTTGTTGATGATGAACATACGATAATCCTCCCTGATAATTAGAAGTTGTAGGAGTTCCAAATGCAACTGATTGGAAAGCACTATGAGTGGATTGAGAGGAGTTACCATTTGCCGTATAAACAGGGAGATTGTTAGCTGAGAAGTTTCCGGATTGAACATTGACACATGATTTGGAACGACTGAAGGGATAATCAGCATATCCAGCAACAATTGC

>JAP11706.S|Supercontig_0000180:370215-370363

CTTAAAAAATACCCACTATTGTTGCAAAGTGGAAAAGAATGTAAAATTTTAGAAGGTTTTGGAGCAAAACTATGTGATTTCTTAGATGAGAAACTAAATAATTACGCTGACGATTTACAGTTATCAGCATTAGAAGCGCTACATTATGG

>JAP11745.S|Supercontig_0019083:150662-150780

CCAATTACAAATGAAAAACAGAATGCCACATCTGCTATAGCATAAACACTTCCATAGACTGCAACATGTCTTAAATCTACTAAATAACCCATGATTGGCATCATTGATGAATCAACCAT

>JAP11752.S|Supercontig_0000303:502278-502640

TTCAAATTTCTTTTTAGATTGATTGCCTTGCATAGCTAATGAATAAATTTCTTTACGATATATGGAATGTCTAGTTTTGTACAGACCTACATCGATATAATTAAAAGTTTGATCTGGTGTCTGATTTAATACAAGTTGAAAAACGTTATCTTGAATTGATTTAGGTAATAACAATATAGCAGTCATAATTAATAGTATTAAGGCCATCAGTGATACTAAGGCAATTAAACCAGGTTGAAGTTCTACTTTTTCTGGACCAATAATGAATACCATGGAATTGTTAATATTCACCGAATTATTTATTGAGACATTTTGTACACTTGTTTTCTGAATATCAGTCAGAGTAATCATTATTGCGACATT

>JAP11772.S|Supercontig_0000310:711315-711563

ATGAATTCAGATGATGCTGATGGTAATTGTAAAGTGTTGCTTAAATTGTCCCATTCATCTAATATATCTTGAACATAATTTTGAATTGATGCAGAATATGTAAATTCCATTGAACCATAAACCTTTTGATTTAATTCAGATAAATGATTTTGTGCACTGTTCCAAAGATCGCCAAGTAAGACTAATTGTATACCTAATTTACCATATTGAGGATCCAGTTCTTTGATACATTCATTCTGAAAAGTAAAT

>JAP11803.S|Supercontig_0000625:134680-134842

AAATTATGATATGACATTAGAATGTATTGTCAATGGATATCCTGATCCTGATTTATTATGGTATACTGGAATTTATGATCCAATAACTAATAATAAACCCCTATTTGATAATAATCGTTATGAACTTGAAAAAGCTCAAAGTTATGGTTCAGCTGGTACAAAT

>JAP11820.S|Supercontig_0000238:495023-495149

TTGTTGAATGTCTACGTTTTGGATTGGTACATCTACCTTTAAGTGGTTTGAATAGTTTACAATATAATTCACCACAACAACGAGTTCCCAATGTTTTGCTACATGCTTCACCTTCTTCATTACATTT

>JAP00022.C|Supercontig_0000168:172342-172440

CTCCAAGAGTCATTAGAACTCTTTTGGCGGCCTTAGCACACCCCTCACAACTCATTTCCATGTTAAATGTATATGTCCTAACTTGTCCTTCCATAGTCC

>JAP00027.C|Supercontig_0000108:94148-94335

GAAACTGAAAGCAGCATGGAAAAATCACTTATTCGTGTGATATTAGAACGTTATAAGCAAAATGGTGTCGTTGGAAGACCTGTGAATAATAGTCGTGTGAAAATGGTAGTTCAGTATGGTCTCCAAATGATTCAACTGCTTGGGCTTGACGAAAATAAACAAGTTTTAAGGACAAATTGTTGGGCTGT

>JAP00028.C|Supercontig_0000146:980250-980401

TTGGTGAAAAATGTAGTAAAACTGTTTTTGATCGATGTTGTGGTGATACAGTATGTCATTTATCTTCACCATTTCATGGTAAATGTGTTAAATGTTTAAAAGAGGGTACATTATGTGTATCCGATAAAAATTGTTGTTCCCATAAATGTAAT

>JAP00035.C|Supercontig_0000215:312523-312858

CTTTAAGTGTTGGTGTGTAGTGAGTAAGCTGTATTTCCTCACCAGTAGAAGCATCGATAGTTTTACCTTCCTCGTCGAAGATCACACTAACCAGGTAGTAAATTGATAGCAGTTTACTTACTTGGGAATATCCTTCGACTTTCTAGTGAAAAAGGATAAAGACGTATCATTACTTTACCTGGAATCACTGTTTACAGAATGACTACTCAGAATTCCTGAATTCAAGCGAGCGTTTATTTGCGCTTGTAGCTGAGCAGCTCGCCTCATTTTATCAACTGTCTCTGTATCAGGAATGACTCCTGGAGGAAAATTTAACCCATTAGCAGCACCATTCTC

>JAP00041.C|Supercontig_0000096:1392666-1392762

TTTTCTTAAAACTAAAATGTGTTTTGGTAGATTCGAACCAAGTGAAGTAAGACAAAACAATCGGGATAGTGCATGTACAATTTCCGGACAAAAAATA

>JAP00074.C|Supercontig_0000450:208012-208236

CTCTTTCATCATCCTCTTCTACCTCACATAGAAGTCGTTCAGCTCCTCCTTGTTCAGAAGACACATCTGAAGAAACAACAAGCAGTCCAATTAGTGTAAGATATGTTTCTAATAAACCAAGTGAAAATCTTTCATCTCAAATCTCAACAAATGTTAAGAATTCTGTACCCGAAAATTCTTCAAATGTTTCATGTATTCTCCCAATGTATGCAGAAGAAGTTCAAG

>JAP00083.C|Supercontig_0000283:380749-381010

CAGGTTTACCATGAAGAAAATCGTGGAATGGCTCCTTGTTTTATCTTTAATTTCCGCAATATGGGTATCTAAATTAATGGGGATTATCACTGTACAGTCTGACTGCGGCAACATAATCCTCAACTGGTTGCCTTTTCATATATTATTCATTTTTGGAACAGTTTCTGTGTTAATTATACTTTATCGTACGTACAGTTTTAATGATTGTCCCGAGGCGTCCACGGAGCTTATGAAGCTAGTCAACGAAGCGAAACGGGATCTC

>JAP00093.C|Supercontig_0000378:430177-430409

ATTGCTTTTAAAGGGTCATCTTTATGCAACAGCAAATAACCTTGGACTTGTGCTGAACTCAAAGGTGTATCCTTTAATAGACTTGCGAACTTTTGAGCTAAATTATTAATGTCAGACGACGTCCATTGTGGGTAGAAGCGACTAAACATGCGCACTAGTTGAGACGAGTCGCAAACGTCAACTCGTATACGCATATCAACGCGACCGGGTCGTATTAGAGCAGGATCAAGTCT

>JAP00115.C|Supercontig_0000141:270659-270742

TGCTATAACTCTTGCTTTAACTGGATAAGGTGCTTGTTTCATTATTTCTTTCATCACTTTCCGATCATCCTCACCCACTAAAGA

>JAP00117.C|Supercontig_0000228:511553-511630

ATTACTGACTATGTAGATCCTGAATGGCGTATGATAGCATTTATTCAATGGTATCTTACTACATTTCATTCTGGACAT

>JAP00125.C|Supercontig_0000483:266243-266668

GTTTTATTATTCACATTTGTTATTGTTGCATTAACAATTGATTTAGCATTTAATAATGCTTTTTGTAAATAACGTGACATTTTCTATTACGTATTTTACGTTTAGCTGTTCTATGTAATTGTTTCACCATAGATAATTGATCAGCATGAGCATAATATAATAAAAAATGATTTACTAATACAGGTATTGTATATGACATTAATAATGTACCTAATATAATACATGCTGTAGCAACAATATAACCCCATGTATTATTTGGTACTAAATCACCATAACCAACAGTTGACATAGTAATTAATGCCCACCAAAAACCTTTAGGTATATCAGAAAATGTTGAATCAACAAAATAAATTGCAGCACCAAAAAATAATGCACTAACTAATATTAATAAAATAATAATAGATAAATCAGTTATTGTAGTACGTA

>JAP00135.C|Supercontig_0000159:242262-242788

TTGTTCTAGATAACTAGACCAAACATTAGTTGAATTATTTGGGATAACAGATCCTGTATTCGTCATCACTGTTGGATTTGCAAAGTAAGGATTCATTTTTGTTGACCATTCAGTATATGCTTGTAAACTAGTCAACTGAGCTGGTGTTAAAGCTGGTGAAGAGAAAACTGCTTTTGGATTAATCTTGTAAGAAGGATAATATTTTAAATGATTTAAAAATGATTTGTTTTGATTAAGAAACTTAAGTTGAGAACTTTTATTGTCCGATGATTGAACTGTATCATCTGTTTCAGATTCAACGTTCTTTAATGATTCTTGTAAATGACATTGTTTAATATGGATTTTGCTCGGTGTTATGTTGTTGATGTGATCCTCGTTGTTAATAATACTACTTTCATTGTTATAACCGTTAGCGATTAATATAGGTGTATTATCCTCAATAGTTTGATAATCACAATTACGACGAGGTTGTTTAGTGATGCGCCTTAAATTAGAGGCATTAATATTTTGTAAGTTTGAATCGAATG

>JAP00156.C|Supercontig_0000452:234958-235276

AACCATAGTAGCTAATCGAGAGGAAAGAGCAACTTCTAAATTTCCTTTCAGTCCAAGAATTGGTGGGATGATTTCAAAAATACTTTCATCTTTCGAAGTTAAACTGCCCTACAGAAATGTAAATTATTAATCATGACCATACGGAAGACTTGAGCATATCAACATAACATCCCGCTACTACCATCCCAACACCTGCTAAAAGACAGGCGGGAAACATTTCACGAGCGACGTAGTAAATATGTCGAGAAGAAGACATTGTGAAATAAGTGTTTAAGCAATGAGAACATTTCGAAAATTCTTCAGACTTTGCCCAAGGAGT

>JAP00180.C|Supercontig_0000360:380818-381088

AGTATACAATATGTGTGTGCAAACTTGATTATCACGGTAATTATTGCCAAGACAAAATAAATGCTTGTCTATTTCGTATTGAACATCACTTACAACCAAATGGTGGAACATTAATTGCTGGTAATACAGCATGTAATGTTAATAGTTCAGGCAATAAATGTATCGCATTAATAAGTGCTGAAGGCGACGCATCATATCAATGTCAATGTAATGAAACGCACTGGATACCAAATATTGAATTACCTTATCCTAATTGCTTGCAAAAACTGAC

>JAP00183.C|Supercontig_0000132:205749-205980

ATCGCGAACCAGTCTATGTTTCGTATGTCCGATATATGGCTAAATTTATGCCCGCCAATTGGCTTGTACGAAGTGCACAAGCATTAAAATTAATGTTAATCAAATTAGTTGTGATACAAATATGATATGGCTTAATGCATTAATTGGTCGTTTGCTATGGGATTTTCTTCGTGAACCTTATTGGTTAGAAAAGATAAAAAATAAGATTCAAGCAAAGTTGAAAAAAATACAT

>JAP00203.C|Supercontig_0002027:49636-49751

TGGCTCTTTTTAGATCACTTGAAATAATAATATCTGGTTTGCTTTGGTTAATGAGAGTCCGAATATTCTTAGCTTGCTCAAGTCCAACTGCTGATAGATCGGTATCCAAGTGACCT

>JAP00205.C|Supercontig_0000213:628380-628716

ACTGAAGCCAAATCAATATCGGACGCTTGAGGGACACCTCGACGACTTTGTTCCATTACTCGAAGGACTTCATGAATTAAAAGTCGAGCTATTTGATTCAAATGTTTCTGAAGGAGATTGGCTGCAGATTGCGATAGGTTAGAAACGCCACACATCTCAGCACACAAAGTAGCAACATCAACATTAATCTCCTGCTTTGGCACCTTTTCAGATACAACAGATGCACATTTGGCTTGCTTTTGTGAATTACGAGACAGCTTCTGTCTCAAACCTAGAGCTTTTCCAAGCGATGACGATGAAGGCTTTCTTAACAGTTTATTTAATCTTCGTTTTTCTG

>JAP00206.C|Supercontig_0000003:1053016-1053127

TGTGAGCTATGTTAACAATGCAACCAAATTATTATGGTACACGAGTTGATACACGTCTTGTTAATGGTCCAGTTCTTAATTCACAATTAACAAATTATCCACAGAATCAACA

>JAP00207.C|Supercontig_0000613:47787-48148

GTTTTCAGTGTACGTCCCCCTAAAAAATCTTGGAAAAATATTGCCAACTTATCAGGAGGTGAAAAAACATTATCAAGTTTAGCCTTAGTCTTTGCATTACATCATTATAAACCAACTCCATTATATGTCATGGATGAAATTGATGCAGCATTAGATTTTAAAAATGTTTCAATTGTTGGTAATTATTTAAAAGAACGTACAAAAAATGCCCAATTTATAGTGATCTCATTAAGAAATAATATGTTTGAATTATCAGATCGTTTAATTGGTATATATAAAACATATAATATAACAAAAACTATAACATTAGATCCATTACCATTAATGGAACATTTACGTAGTTTAGTGATACGTGTTGCTAC

>JAP00268.C|Supercontig_0000665:29640-29845

CACAACGTACACCTTAAACATTAGTCTGATAGCCTCTTTTCTTTCACGATCAACAAACCAGTTTACAACATGCTTGCATCGTAAAGGAGGTTGTTGATCATCATTAGTTGGATAAAGAAGTTGGAAAAAACGATGATAATTACTCAACGACCACGCTGACCGGAGTTTTAAAGCAAATGAAATACATGGATTAGTTTTGTGAGTTG

>JAP00279.C|Supercontig_0000193:988864-988986

TTTGTCGACCTTCGATGCACTGTTTGACCACTTAACCAAGGCCTCAAGCCTTATGAAAAGATGTTGTGTACGACTAAAAAATGTAGCGATTTCTATCTTCTTTTCAAGGTCGGTTTTGCTTGG

>JAP00292.C|Supercontig_0000152:755832-755935

ATACGGTGAGAAACTAAAGGAAGTGGTTGTTTAAAATCGTTATTACAATTGGAGATTCCCATTCCGAGACCAAAACCAGTCCCGAAAATAAGCGGCCATGGACG

>JAP00308.C|Supercontig_0000008:306179-306353

TAGGTGTACTTATGTGGGAAGTGTTTACCTGCTGTTCAGAAAATCCTTATCAAGGCATGAATAATACACAGGTCTACCAGTATATTGTTGGCGGTGGGCGATTGCATAAACCTGCTGTATGTCCTGATCGAATTTATGTTTTAATGCTTGAGTGTTGGCAACATGTAAGTTTTAA

>JAP00310.C|Supercontig_0000095:1170596-1170803

TTTGATAGTAAACACCTGATTGACTTGAATCAACGCGTTCCCATCCCAGTGGTATATACGGTCCAATGAGTGGATGATTCCATGTGGATCGTCTGGTTGAATCACATGTATAATATGATCGACCAAGACCTGATCGTCTTTCAGACCATCCATTCGGTAAAATTAAACGACTATTGTTGTCCATTTCAATTAATGACTAGTAAATATT

>JAP00320.C|Supercontig_0017501:10211-10349

GTATTAGGCGTTTGCTTCTTCACAAGCGTCGATCCAATCATTGTAGACATCAATCTCTTGGCTAAGATAATTTATATTAGTCTGAAAGTCTTCTAAACATATTGTACACTTAATGTAACCAATATTGTTATCCCGTTGC

>JAP00330.C|Supercontig_0000000:3991446-3991586

AGAGTAAAAGAACATTATGCTGAATATGAGCCTATCTTAAGATTATTAAGACAAAAATATGAAACAGCAATGAAAGAGAAAACATTACATAGAATTGAACGGGATAGAGCTATGAATCAAGTGGAAGGACTTCGACATGCT

>JAP00334.C|Supercontig_0000171:744651-744864

TCTATCAGATCGGGATTTACTTAAGTATAATTGTCCCCTGTTTTGCATTCTCTTGCGTTCTGGATCGTTGGACATTTAATCAGTGGATAATCAACCAGTGGAATTTCATTAAGTTCAATCTTTTTTCTGGAGGATCACGATTTTATGCCGTTCAGCCTTGGCACTGGTACTTATCCCAAGGATTTCTGGCTATGTTGTTAACGCAAACACCTTT

>JAP00344.C|Supercontig_0000167:488264-488412

CTAAATTATGAGGATCTGAATGAAATTAGAAAGATGAAGATACTAAATGATTGTCTTTCTCCACGGTGTCCTGTCACAGTGAGTGGTAATAGGCGGTATTTAATCCTATCTTATACCACTGAATTCGACAAAATTCATTACCCTATACC

>JAP00357.C|Supercontig_0000306:626578-626819

TTGGTCAAGTGGATACTGTCTTTGGTAATATACCTCTTATATATGAATTTCATAAGCAAACATTTGAACCGGAATTAGCTAAATACACTGAAAGTGGAGATTTCTTACCGGAAGATGTTGGACATTGTTTTGTTTTGCATAGTGATCGATTAGCTGAATTATATGTAGAGTATTGTGTGAATAATACTGAATCTACTAGGTTAATTATTGAACATGGACAAGGTTATTTTCAATCTTTACAA

>JAP00374.C|Supercontig_0000341:25219-25344

TACTTTTCATCTTTAATATGCAAATAAAGACGTCGGATTTGATCGATGCACAGAGTCAATAACACTGAGCATATGAACAAGGCGAAAAGAAATGGATATATGTATCGAAATGCAACTTCACTATCT

>JAP00450.C|Supercontig_0000353:82608-82830

TCGACAAGCTGTTTTTTTTCTCTTAATATCGTAAGTGTTCTTTCGTAGGCAGACTGAACAAGTTTCCTAACTTCTTCGTCTATTATTTGTGCAGTATGCTCAGAATATGGTTTGGTAAGAACCATATCACCTTGCTGAGGCAAATCGAAACTTAGCAACCCAACCTTTGAACTGAATCCTAGTTGGACAATTTGAGCATAAGCTGATCGAGTTACACGCTGTA

>JAP00498.C|Supercontig_0000019:1599417-1599543

GTAAAAAACTGTTTCATAAGAGGGTCTATTGTGAGATATGTCCAGCTCCCTGCTGATGAATGCGATACGATTGAATTACAAAATGCATCTAGGAAAGAAGCAACGGTCGGAAAACAACCTCAAAACT

>JAP00503.C|Supercontig_0000144:669264-669412

TTGTGAACAATGCCAGAACAACCCACAGATTATCGTGTAGCTGTATATGGTTCAGGAGGAGTTGGTAAAACATCACTTGTACTTCGTTTTGTACGTGGAACATTCCGTGAGACATATATTCCAACTATAGAAGATACATATAGACAGGT

>JAP00506.C|Supercontig_0000657:218676-218869

CAATTACTTGTTGATACTGGCAGTAGTAACTTAGCAATTGCTGGTAGAAATTTAACAAATGTTGATAACTGGTTTAAGTCTACCAAGTCGTCTACATTGAGATGTAGTGATCACTTAATTCAACATGTACGTTATTTGAAAGGTTATTGGACTGGTGTTTATTGTCAAGATGAATTTGATTTTACAAATAAACA

>JAP00512.C|Supercontig_0000020:382196-382419

ATATGTTTTCTTGCTTCAATTTCTTTTAACATTACTTTTACTTCATCAATAACAAATGTTTCAGCACGTTTACTTGGATTCATTAATAATGATGCAACATCAGCATGATTCATTGATGTAACAAATTGTCCAGTTGTAACATTATTAATACCACTAACTACATTATCTAATGTTGTCATCAAGGCACCAACATTCCCAATCCCACCTTGGGATAGATTCATATT

>JAP00516.C|Supercontig_0000374:277173-277766

AGTTAATGAAGCCGCAATTTGTAATCCACCTGAATGATCTGGATCTAAATAGTGTATGTTATTGTCTGTCATTAATTCTGTGATCAATGGAATCTCAGAACCGCTTCGACGTTTAAGAATATTATGTGATAATGTTTTATGACGATGAAAAGGAGAAAATTGCATTGCTCTTATATTTAATGTACATAAGATCACTTCTTTATCCAACATATATGGATCATCAACCTTAACTGTACCTCTTGATCCTAATATAACACAAACTGATGAATATTGTATACGCGCAGCTCTTAGATCAGCTCTAGATAATGGACTACCGGCAAGTGTATAAACTTTTGGGAAATTAGCTATATTTGACCATTCTCTTTGTAGAAATTTTATATCACCTAGAAACAGTATAGTTTTTAATTGATTAGGATGAAAATTGCTTGCTCTTAATGGCATAACAAATGAATGCAATCCTAGCAGTGGAGCATCTGGTTCAGCTAGAATACATACTAGAATATGATTGTATAAATCATGTGATTGCTTATGAAATGAGTTTGAGGTAATTAGATTATCTTCAAATTCTTGACGGCCACAATAGTAATACATACC

>JAP00545.C|Supercontig_0000481:379486-379787

TCACATTTTCACTGAGTGTTTTGGGATCTGTCTGCTTAAGTATCGTATTGTGGTTCCATAACCAGCAAATACTAATCACCATTTGTTTAATTTGGCTTGGAGCATTTGGTTTCTCTCAGTTTTCCCTGTGTTTAGAGCTAGCCGCAGAGGCGACTTATCCAGTTTCTGAAGCAATTACAACCAGCTTTCTTATCATCTCCAACCAAATAATTTCCCTTGTAATGTTGCCTGTTTTACAGTTTACCGCACCTTTAGCTAACAATTCAACTGAAATTATCAGCACTTGTGGTCCTAATGAAGAT

>JAP00552.C|Supercontig_0000140:72190-72393

CTTCTAAATATTTAGCAGCATCTAAGCCAACATCTAAAATGCATATTCGCCCTGACTGAAGAATCGATCGAACCGATTCTAAACTTGTACCATAGTAATGATCTTGAAAACGATTAACTTCAATATATTTGCCTTGTTTAATATCTTCTTCCATAATTGATTTCGATGGTATAAAATAATAATCAACACCATTACGTTCATTTG

>JAP00555.C|Supercontig_0000083:1041738-1041998

TCATATGAAACACATTCTATGGAATCTCCAGGTCAGGATTGTTATATGGATTTATCTAGTTCATTACGTACACATAGCAGTCGTTCATCATCGTTAGCCGAATTTTTACGTTTGGAAACATCTTGTGATGGTAGTCGTGGTGATCTGTGTGTCGATGATGTTGATAATGATGATGATAAACGACTTTGTGAACATGATCAAAGCTTACATGATCTTATTCAAAGTATACAAGAATTACAAAAACAGCATTTAGATGAATCA

>JAP00556.C|Supercontig_0000492:303925-304060

GAAAAGTTTCAAATCTAAATTATGACTATTTTTTCTTCGAACACACATTAATACTCGACTGAAATAAAATCCATCCGATTCGGTAGTGAATGAATCTACCGATTCATAACCATGTATATTTGGAGAATAACTACGT

>JAP00563.C|Supercontig_0019052:20670-20976

ATCATAGACATTACTATTAATCCATGGAAAATTACATTGATCTATACATTCTTGAAAAATATCCATACCAAAATCAAATTCATGATTACCAAATAATGAACAATTAATAAACATTTTATTCATAATTGATGGAATATGTCGACCATGAGTTGCATTACTTATATATGATGGACTTAAACAATCACCTGAGAATAAAACAATACTATCTTGACCAGCTCCATGTTGTTTTAATGCTGTTGTAAATCTTGCTGCACCAGCAACTGGTTCTTGTTTTTGTTCTTCAACATTATATACATCATTAAAATGT

>JAP00567.C|Supercontig_0000082:406395-406487

TAATTTAACAGATTGTGATAAACGTAATAGTTCAGCCAGTCTTGACAGTGGACGAGATTCTACATATGCTACAGGAAGTGAAGGCAGTAGTGG

>JAP00585.C|Supercontig_0000064:1880528-1880772

TTAATATCTCCGTAATACTTGCAGCACCATCATGATCAATACCACAAAATGCAATACCCAAACGCTTTAAACATGGACAACTGATAAGACTTGATTTCAAATCAAATAAACCGGAAGAGCCAATATTTAAATTGCCAGATAACTGTAACGTTGTTAAACGTGTGCAGCGTGTTAGGCCACTTGCCAATATACTCATACTTGATCTGGTTATCATATTTTCTGATAGAAACAATCGGATTAAACCT

>JAP00591.C|Supercontig_0000461:243475-243692

AACTTAGCTATCTGAGATTCGTGGTCACGCAAGTTTTCGTCTAATGATTTTAAACTGGATTCCGTTTCAGCTAACTGAGTTCTTAGTGATCTTTCTTCGCCATCTACATTATTTATACTATTCATTTCTTTATCCACTTCGTTCAGACGATCATAAAGTTTCTTCATATCATCTTCACTTGGAGCGTTCAATACTCTGTTGATACGACAACGATAATC

>JAP00594.C|Supercontig_0000444:132450-132633

TGAAAATGGAGGATTTCACATCTACACGATTAACTAATAAGCTATTATGTCAAATACATGATCCTTTAGCGTTAGCTTCTGGTGCTTTACCTAATTGGTGTTTAAGTTTAAGTCAACGATTTAATATTCTCTTCTCATTCAATGTACGTTCCCAGTTGTTTTCAGCTTGTGCATTTGGTCCAGC

>JAP00609.C|Supercontig_0000024:1172811-1172889

GAAATGTTATATATACCACCACGTTGTTGGCATTATGTACGTTCATTAACATGTAGTTTTTCAGTGAATTTTTGGTGGA

>JAP00621.C|Supercontig_0000459:268807-269003

TCAGCTCTATCTTCTAACCATAATAAAGGTTTAGCACCAGCACGTAAACCATGAGCATAGATTGAAGCAATTTTACGATAATTTTTACCTTTTTCAAGTAATTGACACCATGTAATATAAAATTCAGAACACATTGTACCAATACCTTGTCGAAATAATAATTCAAATAATTCAATTGGTTGATTACAATATTCTGT

>JAP00624.C|Supercontig_0000445:54171-54327

TCCAATTGACTAATTCAATAGTTCGTTCAAGAAGATTTGTAATTGATTCCTGTGAAGTAGAATTGGGTTGTAAATTTTGTGAATTCGAATTATTAATATTTGGTAAATAGTAAGAGTCTGGCGCCTGGTAAACATCATCTCCTTCACCACCACCTGA

>JAP00629.C|Supercontig_0000248:117987-118266

CTACTTATACGACGGTTGTGTGCTGTGTTAAACTCGGTTAAATTATCCAATTCAACCTCGGATTGAGGAAGTTGAGAGTAGAGTAAGCCCAAAGAGTTTGTAGCCGAATCAGACCCATCCAAATTGCGATTTTCTGGACCCATTGTACCATTACCATCTAATGTTTGCGAGTTCGGGTCCGAAGGATTTCCGGAAATGTTGTACAAGGGATTAGGACGTTCACGGATGATATAAACACGAGTAGACGCACCAAAATGTATTTCGGAGTCGATTGGAACCT

>JAP00631.C|Supercontig_0000063:1653239-1653434

ATTATTAGTTTGATTTGAATCAGCTGATGATGATGACTTCGATGACGTTTGACGACGTTTACGAAATGCTTGACTAATTAAACATGTTTCACAGTAAGGGTCTAATTGCCAAAATGCACCTTTACCAGGTTCCGATTGAGAACGTGGTACACGGATAAAATAACGATTTAATGATAAATTATGACGTATTGAATTC

>JAP00645.C|Supercontig_0000050:1348205-1348442

AATCGTGGAACAATAGTTGCTGTCTTTTGATATAAATCAAATATTGTCTGAGAATTACTCAATTGACCTTGAAATACAGCTGACTGTACGTGCATTAATTCTTCCATGGACAGAGTAACAACAGTATCTTCGAAACCACCAATTTTTTTAATCCCATCATTATCTAATATAATTCCGTTAAAAAGTATTAGTGGAATCTTCTTGATTGACTGGAGTCCAGAACGAATAAGAAATTCAT

>JAP00664.C|Supercontig_0000001:1922527-1922760

CATCATCTATGCTATCTTCAAATTTAAAGCGTAAAATTGATTCGTCAAATAATTTGGATTTTGATACTCATCGTTCAATGTCATCATCACTGCCTGGTGTTCAGGATCGTTTAGATTCTAACGTTTGTCCTGAAACTCTGCAATCATCCAGTAATCCAAGTCCTCTAGAAGGTGGACGTAGTCATAGTTCATCTACCCCAACAGCTTCCGAAACAGATAGTTTCACAAGGGATC

>JAP00665.C|Supercontig_0000308:749335-749471

AGAAGAATGCAATTTCATACCTCTAGCACTTTCCAAAAGATTTAAGCGACGTAGACAATCAGCCAAAGAGCTTCGTAGACAAGCTAGTTCATTGGCTTGTTCAGCACTCCGTTTTTCCAAATCTATAACACGGTCCT

>JAP00666.C|Supercontig_0002037:76138-76513

CTCATTTATACGTAGTAACTCCAGTTCAACAGTACCAAGTCGTGCAAATTGTCTACGCATACGTCCACTGGTCGATGGAATATTTAATAGATTCATTTGATCTGACATTGGTAAACCTAAATTAGGACTTCTAAATGTATTGGAACCACTCGTATGTAGATTAGTTAAACTGAATGAATCCGAATTTGGTAGAGATACGTAATCAGTCAAAACAGTAAATGGTGTATTTGGTCGTAGATCAGGTGTTATTGGACACATTGGTTTTGGTTCTCTTAATCTTTCAGAAGTTTCAATGGGAATATATGTATTCCCAGTCATAACTCCAGCATCCCATGGTTCTAAACGAAATTGCATTCTCAACGGCCAAGAACAATTT

>JAP00669.C|Supercontig_0000131:2565569-2566041

ATCCTATATGCTTTATTATATGTGATATGACATGATCCAAGTCCCAAAGTGTGCTGATTCCCACCAACAAAGGGGAGAAGCATAGTTATAAGTGGTAATATCCAAGCAACAGCTATCATTGTAGAGGCTGTAAAACGAGTACGCTTTGTGAAATATTTCATTGGACGGCTGATTGCAATATATCTATCCAGAGATATACAACATAAATTTAGTATAGATGCCATACATAATACAATATTAGCGGATAACCAAAACTGACACAATCGATCAGATTCGAATGGCCAATAACCTAAATTCTGACGAACGATAGCCAGAGGCAGCACTAAGACCGATACAAGTAGATCAGCCACAGCAAGCGAAACAATGTATTGATCCGTTATACGCCGTAAGCGACTATTTGTTATAAAAGCAAGAATTATTAACAGATTTCCGAAGAATGTAGTCCCAGTGGCTATAATTAAAAATAATGATAT

>JAP00672.C|Supercontig_0000155:1431768-1432005

CCTGCCCATGTACCAATGGGTTCATATAGCACCAACAATGTTGGTTCATAAAATCCGTAAAGAAATTGCATGTCAAGGACATTGTTAATCTTTTCTCCAGTTGACGTGCTTAGACACGTCGTAAACGTGGCTAATAATGGTGCTGTCGCTCTTTGCTGCCACAAAAGCGAGTTTCTGACACTAATATGTTTTGGATCAGTCTCTGCGGAAACTAAAACTTCTGTACGTCGGAAAGGTA

>JAP00675.C|Supercontig_0000130:2145275-2145405

TGGAATTTTGTATGTTGTGGAATAAGTTGTCAGAAACCTGCTTATCTTCAACGATCAACGGAAAAATTCTTATGGAATCCTATAATTTTGATTAGAGGTTTTGAATGTCAATGGAAGGACTGTGATTACAC

>JAP00683.C|Supercontig_0000028:247583-247663

TGGATATCTTTGACGCTCAGCCCGTTCACGTTGTAAAGCAATGAAAAAGGCAGCATTCCAGAACCTCATTGACTGCCAAAT

>JAP00684.C|Supercontig_0012211:405-634

TTTTAGACTTTTCATGATTTGATCTTCTCGCTTCTAATTCTCTTGGACTGCAATGCATATCAAGCACATGAGTCATTAAAGCGTACGGTGCTCGAGCCAGTGATGCTGACTGGCAGCCTCGCCAACGACAGCAACGACGTACAAGTTGAACAGGATACTTATGATTTGTATGGTGTGATCCATCTACCTTCCAATTTTCCTTGCTAGGTAAGTGTGTACTGTAAACATGA

>JAP00685.C|Supercontig_0000192:417919-418021

AGAAAAATTACACTACGTCTTAATCGACCATCTAGTTCAGAAGATATAGCTACCTATAGTTGCCGTGCATGGAACTGTTATGGTCGAACTATTACATCAACTG

>JAP00687.C|Supercontig_0000074:952793-952873

GTTGTTTTCAAGACAAAGGATTGCACGTTTTTGAAATGTGTCAAGTTCGAAATCCCAATTTAGTGCTGGATTCTGAATTAA

>JAP00697.C|Supercontig_0000096:758380-758495

GTACCAAGTGATGTACTCCGCTTCTAAACGTTCAGCCATTAACTCATCATCAAAAGGTTCAAGGGTACCACCATAACCTTCGCGAAACCAAGTACCTCGCAAAATACGTGTGGGAC

>JAP00699.C|Supercontig_0000374:133064-133491

TAAAATTTTGAATAAGGATCCAAACAACCACTAGGTCGCCTATTTCTTAATTCTTTGAATATAGCTACAGGACAAGGACATCGAACAGGTGGTTCATGTATTGACTTCATATCGAATTCGCTTTTTTTATAACCCAAAGCACAATATGATGTTGGTGATCCAGCTAAGTAATAACGTTTGTTTGTAATTCGATTAATAAAATAAATATATTCTGTTTGTCTATCTGCAGTTTGGCACAGGCGAAAATCACCCCAACGTAGACTGACATGTTCTGTACGATTTTCTACTTCAAAAACTAACGTATTGATAAACCATGCAGTAAAATTTAAACACCATGGTCTTCGACCACCCAATATACCTTTACGAAATAACTGTTCAACATATGCAGGAAAATCTGAACATTGGAAGGCACTGGCAGAATTAATAAC

>JAP00717.C|Supercontig_0000167:198096-198612

TTCAGGCTAACTCTCTTGATGTAACTAAATATTTGAAAAATCATGGAATTTCCGATCCAGTCATTGACATTTTCACTAAGAACACAATCGATGGATTATCATTTTGTCTTCTAACAGAAAAAGACTTATGTGAAATGGATATCAAAGAGGTTGGTGTTAGAAAACGTATCATGTATTTGTGTTACTTCTGGCGTCATCAAGTTGAAAATCATAATCATGGAGAATATGCTTTTCCAAGATTACCAAGTGAGAATCCTGTAGTTGATCTGTTATGCTGCCATAATCCCAAATTGTATAATACGGAACAAGAAATATCCAGATTAAATGGAAACGATAATGTGGATTGTATAGCAGACAGTACTTTTTTAAATGATTACGATTATGTAATGGATGAAGACAATACAAATTGCTGGAAACTTATAACCAGTTGTTTATATTTCTTTTTTTCTACATGTATTACAGCATCTGTTATAGTTTGGGTCCATGAACGTTTACCTGTTACATCGAAATATCCTCC

>JAP00720.C|Supercontig_0000023:194419-194483

TCTTCATCTACAGATACTGCTAGTGTTAGCGATTCACCAGAATCAAATACACCAGCGAATAAATC

>JAP00726.C|Supercontig_0000262:17719-18020

TCAGCTATATTTTTAAGAGTAATTTCTGTATCCTCATTATTTTGATTAATATTATCCAGAAGATACTCATATTGACTTTCTAAGAATGTAAGATTACGAAATTTTGTCAAGTAAGTGGTGTAAAGTGAAGATAATTCCTCTTCTAATTGTTCATACTCTTCCATATATACAGGTCTTACAGATTGTAATGTAGATAAACGTTTACGATTACGTTCTAATTCACTGCGTTTCTTTTCAATTTTCATATCTAAATTGGTTTCATCAGCGGTGATATTGACAAGTGATGATTGAATTTTGTCAAT

>JAP00729.C|Supercontig_0000205:53815-53998

CCAGTATTTGATTGTTGAGGATAATAGACAAGAAGTTGAGCATTACCACCAATATGTGTTGAGTTAGCAGCAGTAGGATGTGTTCCAGAAGGAGCTAGAAGATGTGCAATACGAGCTGCATTATTGCTAGAATCAGTTGTTAAACATTCAGGTGTAATTACACTAGCCAACATCAAATTATTAT

>JAP00738.C|Supercontig_0000063:627715-627983

ACAGTATGTATATCATCAGGTCGCCTGCATGCAGGATATTTAGCACTTCCGCTCCCTATAGCAGTACTCCCCAAACCACTGGTAGCAGACAAACCACTACTACTTTCACCATTTCCACTATTATCACTGTATAATTGACCATTTTTACCACGTACAGGTGAATCAAATTGTCCAGGGTTAATAATTACTGAAGTATTTGGGAAAGCATTACCTAATTGACTGAGACTATGTGGTGCACTAACCATACACATTGTATCATGAAATTCTGT

>JAP00739.C|Supercontig_0000299:891607-891733

TTCATTTGTTTCGAATCAACCCATTTAGTTAGAAGGGAAGACAGTGTTTTTAAAGTTGGGTAATCAGATAGAACTCGATCAGAACAATGTCTTTTCTTTATAGGAGATGCATATTCACTTGAATCAA

>JAP00741.C|Supercontig_0000038:686088-686296

GTACAACGTATACCCCGATGACAGGTTCCAGTTTCGAAAAATCTTTCACAAGGTATTTCACTAAGACGTGTTGGGTGAGCCAATAGTACTCGTACTCGATCTGGGCTGTTTCCATCATGGTCCACAACTGAAAGGATTAGTGCATTTTGCCGAATAAATTTTCCAGATGAAGTCCATCCAGGAACACTACAACGTTGTCCAACTAAAGA

>JAP00743.C|Supercontig_0000079:169670-170162

AATAATAACAGTTGCGCTAATCATAGTAATAACCCTATGAACAATAAATTTGGCTCATATTTCACATCACAATTACCTAGCGAAACTGCTTTCTATTGTCCTGAACGCGTTTCAGCCGTGGCAGCAGCAGCTGCAGCCGCTGCGGCTATGGTTGCAATGACACCAGCTATCAATAATTCGCCGAGACGATGTTCACATCATCATTACCATCATCATCATTGTCATGCACATCCACATTGTCATATTAATCAAAATTTTCGAGAAGATATTCAACAACGACATGGACAAGGTAATATAGATGATGACTATGATGATGAAGATGATGATGTGGTTGATAATGACGACTTACTGAATGAAGCAAATACTATTGATGCTAAACGTTATCATCATTATCACCATATGTACAATCAATCCGCCTCAATTCAGCATCAACAACATCATAAACCTTACGAAACATTAGATCCTGAGTTATATTCAAATTTAGGACAAAACA

>JAP00750.C|Supercontig_0000192:257680-257799

CAATTATTTAGTGAAGAAGAACGTCGAAAAGAAGAAGAGTTGTTATTGGAAAGCAAACGATTAATAGAAGCATTCGATAATCACGTTATTATGCGTAAAAAGCTCAGAGATGAAGAAATC

>JAP00752.C|Supercontig_0000095:90212-90325

TCCATAATATCTAGGCGTAATCGTTCAATCAGATCAACATAATGACGTGGATCAAATACAACCACATCTTTGACTATTCTAGCAGGTTGGTTTAGTTTGTATGCGTATGGATTT

>JAP00753.C|Supercontig_0000667:24480-24617

ATCGGGTACATTATTTACAGATAACTTTGATTGTGGTTGTTTAAATGACTGGGTACACATTATTACTGATGGACCATGTTGTTCACAGAAATGACATAAAACAACAATGGCATCCATAGTTCTTTTCACGCCATCTAA

>JAP00780.C|Supercontig_0000271:371884-372083

GAATCAGATAGTTCCGATGGAGCTGAACGTAATATTTTTAATGGTCTACCAGAATGATGATGTTGGATAGTTTGTGATTGAATAAGATTAGATAGACTAGGTTGATTTTTATTACATAATTGTGTTATAGATTCTTTACGTATAGCATTTAAGGATTGACGAAATTCAATGGATTCACGATTCCATAAACCTAAGAAAGT

>JAP00783.C|Supercontig_0000096:2052439-2052599

TACAAACGATAAACAGTTTGATATTGATCTACTTCAAGGAGAATCTGATTCCTGGATCTAATTCCCCGAATCCTATGATAATATCCATCTTGTACATTGATCTTTGGACCATCAATAACAATCATTGAACCATCCAATAAAAATTTCATTTTTAAATATCC

>JAP00785.C|Supercontig_0000231:575838-576210

TCGTAGAATATCAGCGTTACGAACAGCTTTCTGTGGATTATTCACCAATAAACGAACATCACGACGTAAATTCTTCCATTTATTGTAACAATGTTCCATGGTAAAACCTAATGATTCCATTTCTGTGCCAATAATTTGCCAAACTTGTTTATGCTTTTTTTGTATATTAAATTCATCCAAATGTTTTTCTACACAATCTAACAATGTTTGAACATTTTGATTCGACCAATGTACTTTCTTTTCAGTTGAATCAGTTGGATGACGTGGGCTATCAACATTTAACATGAATCCACGTGTTGTTCCACCAATATCGTCGTCTTCATCCTCCGGTGGTAACATCGTCACATCGAGTTGTTCGTCAATGTCATCATCA

>JAP00786.C|Supercontig_0000184:815749-815968

CACCTGACAAGTACCAAGGATGGTTAAAAAAACTTGGTGGAAAACTAAAAATTTGGAAGGAAAGATATTATGTTTTAACAGATACACATCTTTACTATTATACTGGAACTGACAGAAAAAAACTTTTGGGTGAATTTTGCTTAGATTTCGCACAAATAGAATCCGATACTGGTGATTTAGAATATAATGGGGATAAATCATGCTTGTTACTACTTAAAAT

>JAP00789.C|Supercontig_0000251:80299-80444

TCAGATCCTGAGCTGCTTTATTCCTAGGGTCAACTAGAATGAGCCTTCTTCCAGTTTCTAGGGCTTCTTTTAACATTCCTTTACCTTCATAAGCTTGTGCACAACGATAGAGTGCCTTCGGATCACCAGGAGAGATACTAAGAGCT

>JAP00791.C|Supercontig_0000110:419191-419446

TGAATATGGGGTTAGATTTTTTGTTGACTGAAGATCAAACAATTACGTCAACTGCTACATGTTCTGATCAAAGAAGTCCACAAATGTCTTCAACAGTTGTTTCTGACGCTGTTCGTTCTCTTCTTACGTCAACGTCTGATACCGTTGGTAGTAGTATATCACCAACTATTTCTACATCAACTACAAACTCAGATGATTTTCCTGTCAATAAAATGAAATGCACTTACAATTCGTGTAGTGAACAGTCTACCTCACC

>JAP00792.C|Supercontig_0000153:773182-773497

TAGTTCGTAGGCGTTCTGCCTTGGAGCATAAATCTCTGTCAACACGAGGATCTAGTCCCTCTAAACTAAAATCTCCACTCTTCTTAGTCTGTACTGAAACAACAGAGTATGAAGTATTTTTCAGCAACGGTGGACTTTGTGGAATTATGGGAAGAGGTTCATCCAGCATCGGAACTTTTTGAGTTTGAATCAACCAATGTAAGTTGGTAATTGCATGAAGCCTTTGACTCCAATCTGCAAGAATACCATGATGTCTTTCAAAACTGTCAGCTGAACGCAAAACATCTCTGTAGCAGTCTGCAGCCTGAACTAACTG

>JAP00799.C|Supercontig_0000493:299956-300059

TTGTGCTGCTTCACGTGTAGCTTGTACAGCTGCTTCATTAGCTTCACATAGTTCTTTAATGACACTATCTAATTCACTCAAATATCGTCGTGAGTCATCTTTTG

>JAP00803.C|Supercontig_0000029:627164-627408

CTTTAACAACTCCGAAGCACCAAGATAGAGTTGTGCTGCATGTGTCGAAGGGTCAATGCGAACCAATGTGTACTCAATCTCATCACCCCATTTCAGTGTATGTTTCATCCGTGAATTCAGTTTGTGATACAATAATAAGAACTCCTTGATACCCTGTTCTTGAATGAACGACGCAATTTTCTTAGTTTCAGGCCAGTTTAGTGGTGATCCAATTGTTAACAAACCCATACTGTACGGAAATATAT

>JAP00809.C|Supercontig_0000478:280787-280898

TTGAATTGTTTATCTTTGTTACAGAAGGATGTTTGAAATCCATTAACTCTTTACTTGTATAACATACAAATCTTGGTGTACTGGAACCGAAACCAAAAGGACATTTTATAGT

>JAP00819.C|Supercontig_0001918:26067-26289

CTAATGATGGATTTCGACTTTGATGCTTTTCTTGATGAGATTCTGTAGATTCTGCATTAGGAGCACTATTTGTACTATTCACACTGTAGGAACTACTTCCATGTGTTTCACGACCATATCGTTCCATAGTATTATGTTTCAAAAATTTTGATTGTGATTGCTGTTGTGGCGAATGAGTGTTTAAACTCATTGATGACATAGATATTTCATCATCTTCCTGCAT

>JAP00826.C|Supercontig_0000666:248394-248617

TATCTACTCCAATTTCATCGGCTGATTTTAGTAATATTCTTCCACCTGGATTAGTTATCACTATTTTAGCGTATCTGGCTAACAGATTGGTTGTTGGTCCTTCAGATAAACATAAATTAAATCTACTTATTCGTGATAATGCTGCATGTATTATTCAAAATGCCTGGCGCCGTTTTCAAGACTACATTAATTTCTCCAAGTTAAAATTAGTACCTTATGGAAGG

>JAP00828.C|Supercontig_0000007:1526829-1527214

TTATCTAACAGATCTTGTCCACTTAATAGACGTGATCTATCTCTTGAGCCTAAAATCGATATAAGATTCGGTTGTGGATAATCTTTAAATGTAATAGTTTCACTTAAACTATGAGAGTTATTTTCCAATGAAGGAAATGATAATTCATAAGTGGAACAAGATGATGATGATGAACCAGAGGCTGGACCACGTACAGATACTTGTGAAGTACTTCTTGACATTGGAATTAACATAGAATCTGAGATATCATGATCTTCTTGATTAAGAGTTGACACCTTGTTAGATTCCGAATTTTGTGCAAAAATGCGACTTTCATTGTCTTTTACAGAATTATTTGAAGAGAAACCAAATAAAGATTTTGATCTATTTGGTGTTATATGGGAATG

>JAP00840.C|Supercontig_0000263:127346-127527

TATACATCACATGGTGGACGGAATGCCATCGGTAGTTGGCTGGGTCATTTACGTGCCAGAAATGTATTTGACTGGCTGATTATTATGGTCAATTCTGATCCACAGTATAACACACCGAAAATTCTGCAAAAATCAAATGTACTTGATAAAATTAAAAGTGATTTTAATGTTCGTACAGGGGA

>JAP00846.C|Supercontig_0000005:2383328-2383490

ATTATATAGACTTTGACCAACAATTCCTGGAACAAACCAATTTGTTCGACCAGCATTAAAACTTGGTGTAATACTACTAGTTGGAGGGAAAAATGTCGGTGAAATTGAACTGGACTCATTTTGTGGTGTACCAGTTGTTTGATTTCGATCAATAGACTGTTGT

>JAP00849.C|Supercontig_0000082:257947-258060

AGGAAGGTTATTTGAAATAATTGGGTTTACGTTTCCAAGTGTTTGTTTCAGCATTTGATATGATCGAATATGTTGAGGATTTAATAATAGATCAGAGCCAATATTTGATTGATT

>JAP00874.C|Supercontig_0000173:732152-732293

TAGTTCCATGGACGCTTTAAGAGCTACATTGGATAGTACTGATGGTCAAGTGGCTACTGTTAGTCGAACTAGCTCACCACCTCCTTCGGATCTTCTCAGTCCTCCTCCCGTTGTTGCTGAATATTGGTGTCATACACAAGTA

>JAP00885.C|Supercontig_0000626:32268-32599

CTCGAACGTCGTTCTACATTGGAAATGTATGCAGATTTCTTAGCACATGCTGATTTATGGGCTGTTATTCCTAATGGATCAACGCCTCGTGATCGTATGGTTTCTTGTCTTCGGTGGTATTTGTCTGCATTTCATGCTGGCAGACGATCGTCAGTTGCTAAGAAACCTTACAATCCTACATTAGGTGAAATATTTCGTTGTTATTGGCCTTTATCTAAAGAGAGTGGAGATGATTCATCAAATGCATCAGAAAAACCACAGGACAAATTATGTAATTCTGGTCCTGTTCCTTGGGCACCTAACAATTCCGTAGTCTTCTTAGCAGAACAGGT

>JAP00894.C|Supercontig_0000033:244614-244823

CAAACGTAAGCAATCTGCAGCAGTAAAATTCGGAAGACAGTCATATGACTTTTCAGTCGAAATCAGATCAATTAACATGCGACGGTAGTAGACAAACTGAGTAATTTGCAAACCACGGACTATTTTGTCTTGTAAGTGGTAAGGATATAGGAGATAGTGTTGTCTTCCATAATCAACAAGTTTTTCGTAGTATTTCTTTTCGCGTTTTAT

>JAP00910.C|Supercontig_0000298:731183-731452

AGGTCGTAACTTTCCTAATCGACCTACGCATCATTTAGTATGTGAAGCCAAATTCAATAATGAAGTTTTATCAACTGATCCAGTTGAACATAATGAGGAACCCCAATTTGAACAGGAACTTGCTTGGGATTTAGATAAAACAAGTTTAAAACAACATCGTTTACAACGGAGTGCATTGAAAGTGACGGTATCTGCTGTTGATAGCCAATCACCAGTTAAAGAACCAATTGGATTTTTCATGTTAGATTTACGTTCATGTTCTACTGAAAA

>JAP00924.C|Supercontig_0000096:2322405-2322503

AAACCTGTTTCTGACAAGTCCGGTGGTAATTTACGAAGATCATTTCCACTTAAATACAAAATTCGTAAAGATGGCAAATAACCGAGTTTTTCAATATCC

>JAP00931.C|Supercontig_0000215:312902-313063

CAAGCCAAGCTCAAACTTCCGTTGAGCTATTGCTCTCTGAGCATTAGCCATCATTTCACGGATTTTTCCTGCAGTGAGCTCCACACTTATTTGAAATATATAAATAATTTCTACATACGGTGCAGCTCCTGTATTTGCTGGCCCAGCGTTAAATGTCGACAT

>JAP00956.C|Supercontig_0000476:13480-13587

CTCATAATATTCTATCCTTCCTGCATCTGGATCTATCACAAACCAACGATACTGGTAACCTTTCATAACATTTGTAAATTTTAGCAGTTGACCTTCGGTTCGTTTTCT

>JAP00961.C|Supercontig_0000144:347045-347668

GAAGACAATTATCTGTACATTGACCAGAATCAATTTCTTCCAAAATGGATCGTACAATATCTAAAATTTCTTGCAACTGGGTAGGACGTTTGATTTTCAATTCACGAGCCCAACCACCAGCGTACTTAGTGAAAAATGAGAAAAATTTCTGATGACAAACTTCCATTTTCATTTTCTGTTTTGGTGTCCTGTCACCATGACGAATGACAGCGATTACACAACGAAGTTCCATTATTGCTCCACAAGTTGTTGGTACCAGAGGAGTATCTACATCCGTACCTGGAGGCAAGTTAGTCGGCAAACACAATCGCGGTGCTATTTTTCGAGTAATTAACACACCGAGAATGTGACTACAGTCATCATAGTACTTTTTGGAAGATTTTACAAAAGAAAATCCATTTACATCACAAACGTAGGACATTCCATTAGCCCGAAGAAGGTCAAATCCACAGATTTGCTGCCGAACAGCTTTTGCCACTTTCTTGGCAATGATTTTCTCTCTAGGTGTTAGTATCACAGGATATCGAACTTCTTTACCTTCGTGATCCCGTTCTACTTTCCCATCTAAGGCAGGAGATTTTCGAGCCTCTGCATGAGCATAATCATCCGCGACCGTGTAAACCT

>JAP00970.C|Supercontig_0000299:714441-714677

GATGGTGATGATGTAGAAGTTGAGATTGATTTAGAAAAACTATCACCCCCATTACCGCCAGATGGTGGATGGGGTTGGCTTATTGTATTTGGTAGCTTTTTATGCATGGTACTTGTGGATGGGATGTGTTTTTCCTATGGACTATTTCTTAGTGAATTAGAAGAAACATTTGGTGCATCAAAAATGCAAATGACATTGGGGGGATCATTATTGACTGGATTTTATTTCATGGTTGGT

>JAP00978.C|Supercontig_0000003:1841250-1841530

GTCAAATGTATCATATTAGCCAGTCCTGGATTTCTACGGGAACAATTTTTTGAATTTATGATTCAGACAGCGACAAGACAGGAAAAACGTGTATTCTTAGAAAATAAATCAAAATTTATGCTTGTTCATTCCTCATCCGGTCATAAACATGCATTGAAAGAAGTACTCACAGATAGTGTTGTTATGAGTAAACTAGTTAATACAAAAGCAACATCTGAAGTGACTGCATTAAATGACTTTTACCAAATGCTTAAAACAGATCAATCACGTGCATTCTATGG

>JAP00984.C|Supercontig_0000243:499059-499235

ACCCATCGATCAATTTCAGTAGGATTTGTTTTAGCCAGGTCACTTAATTCATCAGGACAAGGAGTCGCTTTAATATGATTTGTAGAGGGAGGAACAAATGACATGACAACTTCCTTGTAACCCCATATCCAAGCTAGGAAAGAACGGTTTAGTACAGAATCGAATTCCACAGGATTC

>JAP00985.C|Supercontig_0000221:564149-564409

TTGGCGTAAATGGGTCAGTCTTTATGTGCTCCTGTTTTATCCAAGGAAACAAAAAGCTGGCAAAATGAAAATTATAGTGTGGCAGTATCCAGCATGCAAGGATGGCGTGTACATATGGAGGACGCTCATATGTGTTTACTAGAGCTTCCTGGTGATCCAAAGGCTGCCTATTTCTCAGTTTTTGATGGACATGGAGGTACAAGGGTGGCAAATCATGCAAGTCGTCATCTTCATGAGAAAATTATTGAACAAAGTGAATAT

>JAP00986.C|Supercontig_0000370:116289-116371

TCTTTCAAAATTTCCACATCTTCTAAACCACTCAGGGATTCTGCTTTTATTTGCCATGTGTACACGAACATTCTCGAACCACT

>JAP00996.C|Supercontig_0000240:266411-266584

TTATTCAGAAGAACAAGCGCTTGCTTTACTTACATGGCATAAAATTGATTTTGATCATGCCTTAGCAGATTTATGCAATTTTAGTCCAATCAAATATGAATGGACAATATCTGAACGAAAAATATTTTTTATATCTGTCGACTATTATAATAAACAATTTCATCAAATAAAAAA

>JAP01001.C|Supercontig_0000649:115946-116151

TTGCTACCACTGGAACAAGTATTGTTGACGTGCTCATAAGAATTAGGGATGTAAGTAGGACTGAGCGAACGCTGAACTTTTGAATAAGGATACCTGCAGGTATCTGCCCAAGAAAATAACCCCAGAAAATGACTCCGAGAAGAACTCCCCAACTTGAGCGATCCCAATCAAACGGGCCATCTATCACAGTCTAAAATTTTAAAATA

>JAP01014.C|Supercontig_0000131:50852-51081

CAAATGTTGCTAGATCTAAATGAGCAAGTTCCTCCGATGAATTAGGATTAATCGGTGAGTCAAGCAAGTAGGGAGTTACCCAATGCAATGATCGAATTTTTTCCTGTAATTTCAAATCTATATTTTCATCATCCGTTGTAGGCGAAGCAAAAGCCCAAAAATAAATCCAAGTTGTAACGAAACGCTCAACTGAGTTTAAAACGGATTCTTTGGTTTTTGATGACAAATTC

>JAP01018.C|Supercontig_0000098:294835-294958

CTTACAGCCTTTTGTGATGGTTTAAAATTCCATTTCATTAATGAATGTGTATCAGCAGAGAAATATACAGTGTATTCACGATCTCTAACTGGTTGCATAGTTTTGATTTTCTCATTTTTCGCCA

>JAP01028.C|Supercontig_0000617:3802-4001

CGTATACGCCATCGAAGTATATAATAACCAAGTATTCTTAAAAAAAGGAAAAAGATGCATAATACACCGAAATCGATAGAAACACTATAATCTGGAATATTTAATGCTTGAAATATCATTGCTGGTTTGCCAAGACAAGGATAACTCATCTTATTTATTCGTTTACGATCGCAATCAAGATCTGTACGATTATTTCCATA

>JAP01030.C|Supercontig_0000261:516004-516257

ATCAAAGGAATGTTAAAGCTTTCATTTGAGGTGCAGTCATATAGAACTAAATTGTTACATTTTGTACCATAGACAACATGGTTATTGGTAATCCACTGGCAAGCGAAGATTTTATTTCTATGGCCAGGATTCAGACTGCGTCTTTGCCACACGAATGGGAATTTAGATGAAGACTCAGGTATCTGACCCGTACCAATGTACTGACTCGTGCCTAATTCACGAGAACGCAGTAACTTTATGATTGGTTTGAGTTT

>JAP01036.C|Supercontig_0000132:583046-583161

ACAGAAGAGTGATAGCCGATCCCATTAGGTTTATGTTCATGTCTCCCATCAAGAGATATATTGGATAGAAAGTTGAGAGCAACCAATTTATGACGGCTCTTTTTGCTCCTCATACA

>JAP01049.C|Supercontig_0000227:71966-72178

TTTCAGATACGCTTTCCAGTTTTACATAAAGAAGCGTTTTATTCAGCACCGGTTGCCCTTTATTGATGCTGTTTCTCACGTGCCATGGAGACCTATATATGGCGTACCGATTGGAGGCATTGGTAGTGGTTCTATCGGGCGTGGATATCGTGGGGAGTTTTGTCGTTCAAGCCTGATACCAGGTATGTACAGTTACGACGTACAACCTGTTGA

>JAP01067.C|Supercontig_0000118:587054-587190

TGATGTGAAAACGGTACAATGAAAACTTCTAATGGTTGCTCTTTCCACTGATCCATTTTATATTCAATCGGAAATCCTTGAGTCCATACACCGCCGGGTTGGTCATCAAATGCCAACTGGTTATAAATATTATTCAT

>JAP01092.C|Supercontig_0000138:555903-556128

TCATAATGAAATATTAGATGAATTTAATCGTCAAGTTTTGACTGATTTAAATGCTGCATGGGAAATTGATCCAAATACAAAAAAACCTACATGGGTTATGAATAAAACACAAAAAGATCTTGTTATGGTAATTATTTTGAAAATATCTGATATATCCAATGAAGCACGTCCTTTAAATGTTGCTGGTCCATGGATCAATCGATTATTAGCAGAATTTTTTCATCAA

>JAP01116.C|Supercontig_0000358:240048-240142

TTATGGACTGCTCAAGGTGCATTCATTACCGATTGTTCTACCAAATCTAATTTAAATCAACATTTCAGCCTGTTCTGGGGATTATTTCAGGCTAA

>JAP01117.C|Supercontig_0000488:464959-465134

ATAACAAGCTATTGAATTAGATATCCAATGTGAAGCACGAATAATCCACACATGGGGATTGCAGTCACGAAACTGATTGGTAAATCGTACATACAAATTTTGGGCAGTCTTTTCCAAATTCCATTTAATATACTGATTATTGAAATTATTTTGACTCATATTATATTCGTAATCCT

>JAP01118.C|Supercontig_0000019:179705-179831

GCATTTGCCCTGTTCTTTACACTCACTGCAATCATGGCTGATTTGCTTTGTTACTTTCTTGTACCCACTACATTGATCTACCTGCTTGGTAGTTCATGTGTATGGATGCATCTTTTATGGCAAACAG

>JAP01126.C|Supercontig_0002049:55156-55274

AATTGAAGAAAAATGTAAACGTGAACTATATGGACGTGCTTTATTACATCAACATCAAACAGCACTACGTAAACGTTCATTATTTATACAAAATGAATTAAAAGATGATTTAAATTGGT

>JAP01134.C|Supercontig_0000638:115045-115182

ATAACCACCCATTGAAATTCCGTGAAGTGCAAACGGTCCGTAGCCATTATGTTCACACCATTTTAGCAATGCGCTGCATTCCATAATCAAAGCTCCACCCATGATGAAAAGGTCAGAAACAGAATTCAGACCAGAACC

>JAP01141.C|Supercontig_0000091:30801-31118

CTTAATGTTTGTTATGATGAATTTCCGACTAATAAATGTTTTTGTATTTGTCTCACACGCCAACTCGACACTTAGATCAACGTCTTCTAATTTTAGCACAGAAGGACCACCATCATAAGGAAAATAAGGATGACACCTAAGAGAGCCTTTTTCAACAAGGCGATTGAGCATAATAATGGCGGGACACTGTCTCTCCCAAACCATGAGCCAGAAATGATTCACAGTTTGCTGCATTGGACCCTGGGTGAGTATATATTTTCTGGACGGCACCTCCGGAATGAAGACAAAGCTTGCATTAATATAGTCACCATCACGCGC

>JAP01144.C|Supercontig_0000091:573173-573343

GATTATAATACTCTTGTCGATAAGGCAACATTAGGAGATAGTATTACAAGTCTTGAGATGGATTTGGAAGATGTACGTGCTGCAAATGAACGAGCTGAACGAAATTTAGAAATGTTATTTGAAGATCGTCAAAGAAAAGAATCATCATTAAAATCATGTGAGCTGGAATTA

>JAP01149.C|Supercontig_0000065:95782-95934

TGCACGTAGACGTTTACTATTAACAGGGACTCCATTACAAAATCATTTGAATGAACTATGGTCATTGGCTAATTTCTGTGTACCAGGACGTTTAACTTCAAGTTTAGAAGAGTTTCGTCGTCAATTTGTCATTCCATTATTGAATAATCGTAA

>JAP01161.C|Supercontig_0000010:794429-794676

CTTGGTGATAGTATCGTGCATAAAGCTAGAGAAATTTTAGAAAATGCTATTGAACTGGTTAACAGTGGCAAGATTGTATTACCAGATACTTGTAACGGTCTTCCAACACCACGAGTAGTCTACGGTGATACAGATAGTTTATTTGTACACCTTAAAGGTTATGGTAAATCAGAAGCATTCGATGCTGCATATCAAATTGCCAAAGAAGTAACATCAATGAATCCTGTACCGATTAAATTAAAACTTGA

>JAP01171.C|Supercontig_0000118:571105-571309

CTTAACTCAGATTCTAATATACGTGATAAGAATTTTTGATAAGGATATGAATCGTAATATCCACTCCAATAGTCATGTTGTCGATCTGCATAAGTAAAGAAATCACCAGATAATGATGGAAATAATGTGGACATTGTTGAATTCATTTTGTTTACTCGTTCATTTAATGCATGAAAATAATTGGATAATGTGGAGAAATGTATCT

>JAP01179.C|Supercontig_0000676:436658-436732

AGTATAATATTCAAAGAATTGGATTATTAACTAGAGAAGGGGAATTTGTTGAAGCATTTTATGCTGATCGTCGAA

>JAP01192.C|Supercontig_0000621:91416-91622

TGACTTTAGGAATTCATATGAGTACATTATAATTGGAGCTGGTTCAGCTGGATGTGTTTTGGCCAATCGTTTAAGTTTACCACACCCTAAAACAAAAAATTCTAGTAAAGTTCTGGTTCTGGAAGCTGGTCCTACAGATGTTGGAATTTCCCGATGGACAATTAAGATGCCAGCTGCTCTTATGTACAATCTTTATCACGATAAGTA

>JAP01205.C|Supercontig_0000675:332946-333125

TTTTTAAGAGCGTTCATGAGACTTCGAACTAAAAACTTGATTCCAGCCACACAAGCAGTTGATATTGCATTTAAGCTATATAGATGTCGAGATAAACAGGTTCGGAAAACATTACGTCACTTCCTCGTAAGTGACATAAAACGAATGAATAAATGTCAAAAGCAAACAAAAGCTAATGCA

>JAP01217.C|Supercontig_0000058:124259-124341

TTTATTCCATCGATCAGCACGTATAATCATATCAACTAATGCAGTATAATTTGCTTTGGCAGCAATCATCAAAGGAGTTTGAC

>JAP01221.C|Supercontig_0000067:413040-413191

AGTGGGCTGATCAACAAGGTCATGATCATCGTGAACCATTATGGGATCAACTGACAAAACCAACTACACGACTAACACAGTATCGTTTATTAATGGAATCAATAAGGAAAAACTGTTGTAATTCTACTGAAGAACAAGAAGTCAGTGAAATG

>JAP01230.C|Supercontig_0000176:473157-473286

TTGTTTTTGTATATGTTTGAGAAGCAGTATCAACTTTTGTTTTTTCTTTATGATGTGTTAAAGTTTCATCTTCTGTATGTGATCGTTTTTCAATTTTCGGTGAACGTACCACATAATAATAAGATGATGA

>JAP01237.C|Supercontig_0000285:407139-407362

ACAATGGATACATTATTCATTAGTTCAACATTTGATTCATTTGATGTTCTGTATACATTACTATTTGGACTTCTTGTATTTTGCATTATGGCACCGACTACAGAGTTTATTTCTGCTGGTGTAACATTGGAGAATTTGTTTAATCGATTTCTTGGTAATGAAAGTCAATACTTTGTACAGTATCATTTAAAAAGAACTGTAATGGTACGGTTATTTTCATCGTC

>JAP01239.C|Supercontig_0000123:1348750-1348882

GCAGGTAGATGACTGGTCACCTGAACTCATGGAACTGGAAAGGAAGGCGAAATCAGTATCGGACATACTATTGTTTGTTTTTGAGAACTGGAGAACAAGAGGACTGGTCAGTCTTTTAGAAGCTACATACCTT

>JAP01242.C|Supercontig_0000082:337291-337446

ATTTGACCTTGGTGTAGTTATTTGCAGCTGCTCATCAGTAAAATCACATTCATCTTCGATAACATTGATCAAACCTTGAATTCTTTGTCCAAATGCCGATGCTATTAATTCAGCTTTATGTTTAGCATCTGAAACGGCTTGTTCAACAGCTTCTTT

>JAP01247.C|Supercontig_0000289:478666-478805

TATTCGTTTTGCTATGAATGTGGACGTTCTGTTGGTGTACGTCTAACAGTATGTTCACGCTGTCATAGAGTATATTTTTGTTCCAAAGTTTGTAAATTAAAATCATGGACAATGCGACATAAAAATGAATGTTATTTAAC

>JAP01248.C|Supercontig_0000043:1098872-1099143

AAGTGCCGTAGTGTCACCACCGCTCAAGGACGTGGGCGTCTCCTTATTCGTATGTTATTGAATTCTGGAACTTTGGATTTCCCATTTAAGTTATTGCTGAATAACATGCATTTATCAGCTGCATTCTATGAGGAGTCCGAGTCAGTTATGGGGGATGATATTCTTATTCAAATATTTTCTTCTCTTGTCAGTGAAGTTTGTCGGATTCCTTTTAATTTGAATGTGGATAATACGGAATTCCTTGATGAAACATGGTGTCTTCCAACATTTAA

>JAP01249.C|Supercontig_0000050:886905-887036

TTCAACATATAGTTCATCGGTTAGATGCTGGTATGTCTATTATGTATGCATGGGATTGTCCTCAAGCAAAACGTGAATTAATTTTCTACTGTAATGAAACCGATAATCGTCATTCAAATAAGTTAACTTATG

>JAP01250.C|Supercontig_0000302:61843-62015

ATTGCATGTGATATTCTCTCCAAACTATGTTGTTGAGTCGGATTTTATATTTTATTGCAAATCTTAGTCCACGAAATTTGTCCCATTTCGGGCTAGTCAGCTTTCCACTGTAAGCAATCTCCAAATACTTAAAAAGTGAAGCTAGGTTCTCAAAAATCAGCCTGTTTTTATCT

>JAP01260.C|Supercontig_0000085:618827-619060

AGCACGATAAAGTACAGTGTCACTGCACAACGTTAAAAAGCAATTCTTTTGCAACTGTCTATTGTCTGGAAACTTTTCCATCGCCGTTAAAGTCATACGTACTGCACGATTTAGTAGATCAATAGGTAACTGATTGCTTTGTTCACCCCGAGTTAAGTTGTAAATCACAGCAGTTCCTGCCAATATCATTTCCATTCTGCGTAAATGTAACCTCATTACGCGCAAAACAGCCTG

>JAP01263.C|Supercontig_0000643:100852-101197

TCAGGCTTTGACGGATGCCAATCCACTACAGTAGCTGGGCTATTATTCGTTCGTATTTCATGGTTTAATATGGCTAGGGGATGTGAACAAGCTTTTCGTACATCATATATAACAGGTGATGATTGATTAACATCTATTGTACCTCCTACTAACAATTTTGTTCCACATGGACTAAATACTGCTGTTACAGGTGGAATAAGAGCATTTCTACTTGATCCACTTGAATATATTTCAGATGAAAATTTTGCCAATTTCAATACAGGATATCGACTTTCTCTAAGGTCCCATATTTGTATACAATCTTTTGGTGCTACTGTTGCATATACTGTATATCCTGTACCAAGTG

>JAP01264.C|Supercontig_0000034:301387-301731

GTTAATTTATGTAAATAACCATCTTTACCATTACGAAAAAATAAATCAGGTAACCATAATTGTTCTTTTAGCTTAGGAGAGACTAATGGTTCATTAAATTGTGAATATTCAGGGATATCATACCATGATAAACGACGATCACGCCATACTTGTCTTAATAATAAATCAATATAATAATCCATTGTACGTACATCTATAGAAAAAATAGCTAATACTTTCATATTTACAGTAACTATAGTAGCATTACCACCATCTGGTGGACGTTCATAACTACGATATTTTGACATAATTAAATTAACAATACGATCTCTCATTGATGTTTTTGAATAAATTTCATCTTGTAAT

>JAP01266.C|Supercontig_0000058:1507128-1507381

TACATTATCCTCAAATTATAATGATCATTTAAATAAAATAATTAAACGTGTAACATTGAAAATCGATGTATCGCATACAAATCATAGTCATATAATTGGTCGTTATGGATGGAATATTAAGAAAATCATGATGAGTACACAATGTCGTATACATTTTCCAGATTCAAATCGTAATAGTTCAGTGATTAAAAGTAATCAAGTTTCAATAACTGGTCAGTTGGAGAATGTGGAGAAGGCTAGAAGAATAATTCGAG

>JAP01275.C|Supercontig_0000015:713861-714022

GTTTGCCATTGTTGTTTTCGGTTTGCTTCAGAAATGGAAACAGTGAACTTCTTAAGCTGAGCAAGACCAACAGAATAATATGTTAACGAACTTTCGTAGTTTCCAAGCAAAGCCAATTCTCGCGCTTGCTTAACATTCTCAACTATTTCACTCAAAGACATT

>JAP01282.C|Supercontig_0000755:6587-6748

ATTTCCATGTAAAACTTTACGTACACCATCTAAATAACTAGATGGATCTTCACCATTTATAGTCCAACGTATTTTAGGCGGTGGATCTCCTGATGCTGTACATCGAAATGTTACTGATCCATTTACAGGTACAGTAATATCTTCAGGTTTTTCTATAAATTT

>JAP01288.C|Supercontig_0000050:1359328-1359501

CCTGGAAATAATGTCTGAATGCATAATTACATAGACCATCATCAGACAATGCTTTTAAACTTCTATGCCATGAGTAAAATTCGTGATGACTTAAATCCCCATATAGAATTACTGTAGGAATGTTATTAGAAGTATCTTTTGGTGATGAATACACTTTTTCAAGTGGAAATAAAG

>JAP01294.C|Supercontig_0000481:267548-267699

AATGAAGCTATTCATTCAGCAGTTCAAAGCGGAAATATTGATATTTTAAGAATTTTAATGCGGCCACATACTGATATAACATCAAGGTTGGTAAATATTTCAGAAGAAAATGATGTAGTCGAAGGAAATCTCGTCGATCTAGACGATAATTC

>JAP01298.C|Supercontig_0000096:216957-217102

GGTTGTACTGTTAAACATATGGTTGTCAATTTAAAAATTAGTTCTGATGGTGTTGGTTTAGTTGTTCGTGGTGGATGGAATAAATCTCCGTTACTTATACGACCATTAACTGTTATGCATATACGACAAAATAGTGCTGCTGACTG

>JAP01302.C|Supercontig_0000646:208377-208698

GAGGTAATAAACCCTGACCAGAGCAACATGTTGTGCAAAATAAAATATCTGAATCATATTTTTCACTTAAATCACAAGGTAATTGGATAACCGGTAGACTGGGAAGTGTAGGTTTATGTAGCATTGTAACAACATCAACACTACTCAAACCCCGTAGACGACAAATTAATGGACTTAAATGTGACCAGAATTTTGTGTTCATTTCTGCTAATAAGCTGAATGCTTTGATTGGATCACCAGCTGCTTTCGTCAGGTCGCACTTTTCGAACACCCAACCAGGTGCAAATAAAGCTACTGATAATGGGCGATCGGGTCTAGAAGC

>JAP01304.C|Supercontig_0000488:464959-465132

ATAACAAGCTATTGAATTAGATATCCAATGTGAAGCACGAATAATCCACACATGGGGATTGCAGTCACGAAACTGATTGGTAAATCGTACATACAAATTTTGGGCAGTCTTTTCCAAATTCCATTTAATATACTGATTATTGAAATTATTTTGACTCATATTATATTCGTAATC

>JAP01309.C|Supercontig_0000074:1052502-1052699

CTCTACGGACTTCGACTATTAGGCCTTCGTGATAAAAATATAATTTGTAACCATGTTTTATGTAGGCATTTGACATACTCCGAGGTTAAGAAAATGTATGGAATTAGAAACCTTCAGCTTACAATATATTTGTTTCCTACAAAGTTTGAAGAGGCAGCAAGAAACGACAGGGCAACTCTCTTTTATTTACATCAACAG

>JAP01310.C|Supercontig_0000088:510783-511344

TCATCAACTCACTAAATGCTTAGTTAGTATAGAATGAACACCATCTGGTTCATCATAACATTTCTTTTTATTGTCCAGACAATTATGTAACAATTCTTCTGATAATAAAAGTGATTCTGATAAATTAGTATGATTTGTTAATTGATAGTTACATAATGTGGAATTTTGTGATATACTTCGATGATTTAAATTCGATGGTATACTAGATCGTGATAATGAATCAATTAAAGTGGAATTTTGATCTGTTATATCATTTGATTGATTAACATTAGTACAATCCATAACATTATTATTACTGCCATTACTATTATTATCAGCATTAGTATTGGAATTAGTAAGATTAAAGTAGGCTGAAGTATAAAAATTTGATGTATTAAAATCATGACCAAAATTTGTTGTGCCTATTGTCCCTGTATCCGTTACTGTTGCCGTTGTCGTTGGTGTTATTTGAGGAAGGAGACATGTATTTAGCGTTAATGATTTATTGTTCATTGTATTATCTGAAATTAATGCAGTCGTTGTAAATGCTGATGAATGATTATCATTTGTATCAGATATAGAA

>JAP01323.C|Supercontig_0002060:340419-340567

ATGCATAAACGACGAATAGACATGTTAAATAATGTATTAGGACAATTAAATCCAAATCACTATTTACACTTATGTCGCCAGTTAACATTTGAACTAGCAGAAGCTTTGAGTACATTACGTGATTTGAAAAGGAAACTACTGGATGAGGT

>JAP01325.C|Supercontig_0000376:348061-348338

ATTCAATCATTTCCTTCAAATGATATAGATTTATCTAATCATGAAATAAGTATATGTCATACATTATCTGGTTTAACTAATCGTCAAAGATATATATGTTTACAACATACTGGATTAATATGGGCAATGCTTGAAGGTACACATTTAGGTATGCATGAATGTGTACATCAATTTAAACATGAACAATGGAATTGTTCAGCTGTTAATTTATTATATCGTACTCATATTAAATCATCTAGTTTATCATTAACACATGGATTAGAGGGGATTTTACAACG

>JAP01330.C|Supercontig_0000174:353250-353373

TCAAAGTCTTTGTGACAGTCTACGCACGAAACCAAAGAACAACGTCGGGAAGATAGTAGGTGATTCCGCACATGGCTCTTTTTTAATGTGGCGTTGCATTGAGAACACACGAAAACCACCATGC

>JAP01331.C|Supercontig_0000088:895351-895648

ATAATTTTTTAAACCAATCTTGAAGTGTGACAGCATTTGGACCAACTTCACCGAGCCAAATCCGACGTCGTCGAACATCAACTAGAAGGACTGGAGGGACATCTATTCCCACTTCTCTACAACGCAACAGCTGCCTGACTTCAGCTCGCATTCGCTGCATCGACAAAGTAGCATCCAATGTACTATGACGATACCGTTTAACAAATCTCTCCTTTACAATACATGGAAACGTATATGTTGAATGGAACAACTTAGTGTGATAGATTCGTGCCTCTGCTCCTTGACTGATTAAAGTCTC

>JAP01334.C|Supercontig_0000675:197250-197400

TATCTATTAAATTTGAATGTGAATCTATAGAATGATTGATTTTCTGTGATCTTAATGAATGGGATTGTAATTCAGAAACCATATTGGATGGAACCAATCCACGTAGACCATTACATTCACCAAGATAAAATCCATCTTCATCACATTCAGT

>JAP01336.C|Supercontig_0000114:524312-524722

GATTCCAGTTGAAGATCAATCTTTTCTTTGGTCCGATAAATATAGACCAAGAAAACCACGTTTCTTTAATCGTGTTCATACTGGTTTTGTATGGAACAAATATAATCAAACACATTATGATTTAGATAATCCACCGCCCAAGATTGTTCAAGGCTATAAATTCAATGTTTTTTATCCAGATTTAATTGACAAAACTAAAACACCAACTTATACATTGACAGCATGTGGTGAACCAGAAAAAGATTTTGCTATTCTACGTTTTATCGCTGGACCACCATATGAAGATATTGCATTTAAAATTGTTAATCGTGAATGGGAATATTCATATAAGCATGGATTTCGATGTCAATTTCAAAATAATATCTTTCAGTTATGGTTTCATTTTAAACGATTTCGTTATCGTCGATAATT

>JAP01343.C|Supercontig_0000043:498534-498899

AATAATTATACATCACTTTCAACTCCAATAGATCCTATAAAATCAATTCGTGGTCTACAGAATACAGCATCTTTACTTGGTCTTCCATCCCTATCAATCAATACAATTTTGAATCCTCAACAACAAAATTCATTATTTTCTTTAGCTAATACAGAAGAGAGTATTATTGTCCGAGAAATGATTATTTCTAATGACGTGATCGGTTGTATTATAGGACGTGGTGGTACAACAATCAATGAAATTAGAAACGCATCTAAGGCACAAATCAAAATATCCAACTGTGAAGATGGTGCAAAAGAAAGAAAGATAACAGTTTCTGGAAAGTTGGATTCAGTGAATTTAGCTCAATTCCTAATAAACAGCAGG

>JAP01345.C|Supercontig_0000293:500792-501077

AGTTAATTTCAAAGCTTAATATTTCTCCAGATATTCGAAATGAACCCTTATTTGAAAAAGCTGTCAATGAGTTAGCGTCTTCAAATCTCGAAAACTTATCTTCTTCAGTGTATCAGTTACGGACCGTTCGCGATGTTCTAAAAAACAAGATTAAATCATTGGCTGCTGAAAATTATCACATTTTTATAGATACCAAAGATGCAAGTCAGAATATCTTGGAGAAGACTTCAGAAATGTCAACCGCTAATCAAGCTTTTCTATCGCAAATTACTGAGTTTACACAATG

>JAP01349.C|Supercontig_0000026:1602939-1603106

CAATCCTTTAAATCTATCTTTACTCTGAATAGAAACTTTGAAAAATGATGCCATAGGTGAAACAGATGAAGTTTTTAACAAAGTATCTCTCAACTGAATCATTGTTAAACTTTTGAAATTTTTGATGTTATACATTCCAGCTAACTGGTCCAGCTCCTGACGGTTAAG

>JAP01353.C|Supercontig_0000255:216891-217036

TTTGTTTGGGTAAGTGTGTTCTTTAACAAAAGGAGACGAGCAAGGTGAACACATAAGTTCTTTCATGGTTCCTGACATATAGAACTTGTTATAATGATCTCGGCAGTCCATGGAACTATGTGACTGAAGTTGAGCGGATATTTCAT

>JAP01359.C|Supercontig_0000308:288415-288731

CCTCTTAGCCAATACGCAGATTCTGGAATTAATATTTTATTTCGATAATTCACATAATTCTGATGAGTTGTCTCTTCAGTACCGGAACAAAATTTTGAAGTGGAACATGTAGTATTCAATAGAATTGATTGAGCCACTTTAAGTACATCTTGATATTGTCTCATTGATGCTTGAACTTTAAAGTCTACAATAATTCTTAATGAATATGAAGCCAAATACAAGGCAATAGATACCCAGTCCATAAAATTATAATAACTACGGAAAAAATAACGTAGTCCATAATGATAAACTTGTTTACATTCCATACAAAGTTGACC

>JAP01369.C|Supercontig_0000678:252324-252537

CACGTACAGTAACAATATTTTCATGTTGAGCTTTCATTAACATATTTATTTCACGTAATGATGTAATTGGGAAACCATCGCGTTCTTTTTCCATTTTTAAACGTTTCAATGCAACAATTTCATTTACTTTTTTATCACGTGCTCTATAGACAACACCATATGTACCTTCTTCAATACGATTAAGACATTCAAATTCTTCCACTGATCTACAACC

>JAP01374.C|Supercontig_0000260:142913-143094

AGTCTTATTCTGAAACTACTTACGAAGAATTATTCCAATCTAGAGTGAATAGTGTCAGTGAATGGTGCAGTCATTTATCTATAAGGTTGATAATTATTTCCTCCTGGAGGGAATTACATCATGATCCAGTGTATCCAACTCCACCTATCGACAAGTCGTGGATAGAAAAAACACCACATCTG

>JAP01384.C|Supercontig_0000063:454585-454735

GGAATATATTATGTTTTCCAATAAAAAATGAAAAGGATGGTATAGTTGGTGTTGCACAATTATGCAATAAAATCAATCACCCTTTTTTCACAAGAGCTGATGAAGATGTAGTAAAACATTTTCAATTTACTGCTGCATAAGTATAGTACAT

>JAP01398.C|Supercontig_0000283:423404-423769

GGTAGACCTATTTATGCATTTGAAATTGGTCCAAAATACACCACTTTAGAATTCAATGCAGATAGTTTAAAAACATCTACAACTACTAATCTAAATACTATGATGGATTCTGAATTTCTTTTTGATGTGATCAATGATACATATCTTTCAAATAATTCAATGAACTATATACCGAAAATAGCTGTTATTGGCAACCTACATGGTCATGATCAACTAACACCTCAATTATTAGTTCATTTTTTAGATTTTTTATGTGATAACCGTAATAGTCAATTAGCTGTACATAGTTTACTCAATTCTGCCATAATTACTATTATTCCAATACCAAATCCTGATGGTTTGTATAAAGCGTGGAAAGACTATTCA

>JAP01406.C|Supercontig_0001953:68240-68403

AGTTACCTTCAATATAACGGCGGCCCAAATGTTGATTATGATGTGCCATGGCCTTTTGCTTATCATCTTTACTTTCCAGTTCGATGAAAGCCTCTCCATTAGAACGGCCATTAGACCCCTGAGGAAAATATATACCACGCTTTCCACCTCTTATCCTGCAATCT

>JAP01413.C|Supercontig_0000026:521810-521989

CGATCATTTAGCAATGAAATCCTAAAACGAACGCCACTTTATGATTTTCATATCTCACAAAAAGCCAAAATGATTGATTTCTGCAATTTTTCCATGCCATTACACTACGCAGACCAAAGTATCATTGATAGTCATCAGTTTGTACGTCAACATTGCGGTCTTTTTGATGTTTCCCATATG

>JAP01420.C|Supercontig_0000185:696626-697195

GCAATCATATTATCACTAGTTTGATAATAATCATTTGGAGCTAATTCCCAATATGTGGTATTAAGTAAATTATTAGCAATTGTAAAGTTTTTAATTGGTAAATCAGTCCACCATGCAGTTCGTTTGTAAACACTTTGAGCAATATACATTGGTAAAGGTAGACAACAATTTGTCAATTCCACAATACCAATTAGGAATAATAAGAAAAAAACAGGAGATCTCATTTGTGGTTTACTAAAGACTAGACATACAAAAAAATTAGTGATTAAAACAAAAATCATTAGTATAGGTAATAGATAAGTATGTACAACATGGTCAAATGTATCATTATATAATTCGGAAAATGTTGGATATGGTCCACAAATTTGGTTAAGTAAATTGGATGAAATCACACCGGTTGATATATTAACATAACTATCAATAAGTAGTATCTTTGATAATGATGTATTATTATCTTCATTAAGATTTGTTGCAAAATGTACGGCATTTTTAGCAGTTTCACTATTAAATGCATAAAATATAGGGTTTGTTGATTGAGTAGATAAGAAATGCTTTATAAATATCTTTTCT

>JAP01422.C|Supercontig_0002054:207346-207986

ACCACGAATATTCACACGTCTGACGATATATTGCAATAATGCTGAAAATGCTGGTTTGTTCTTCCGTCAATTTGAAGATAAACTTGGTGCTTTTGATATTGTGAGCTTCTGTCCTACTTCCAACAACGCTTTCGCATATGCATGTGAAAAGGCTGTGAACATTGATCTTATATCTTTAGACCTTACAAAATCAAGTGAAATTCGACTGTTAAGTAAACAATGCAATTTGATGATGTCACGAGGTATCCACTTAGAATTCCAACTTGCTCCGGTACTTCGCTCTTCCTCTGGAACTTATAGCGCACGTTCAGTCTTATCACAGTACTTCAGCAGCTTACTCTGCATAGCACGAAAGTCATTTACAAATATGATAGTCGTCAGTTCCGGGGCGTCAAGCGGATGGGAAGTCCGTCGACCAGTGGCGGTATCTGCAATGCTTGGTTGTCTAGGTCTGCAACCTCAGGAGGCAACTCACTCCTGTCTTACCAAGGCGCCTTTCGCGGTAGTGACTCATGGTCTTATACGAAGCAGAACAGTACATGGTGCAGCTGCACTGCTTAAACTTTTGTCTATGCCAAATGTATCTTCTATAGTTGTTAAACCTACTGTAAATAAACAATCTACAGACGAAGCTAGTTGTG

>JAP01429.C|Supercontig_0000308:910093-910177

TTTGATCCAACATCATTGAATTCAACGTTGATATCAACAAATTTACCACGAAGTCAACATACAGTACATTACTGTCCAAAAATAT

>JAP01444.C|Supercontig_0000488:179087-179250

CGATGATAAAATGCCCCATTTAGTAAAACTATTGAAAAATGCTTCATCGATGGATTATCATCATATTTCCATAGGTTATCTGTAGTATTCCAATAATGTGAACGAGCTTGGAATCCAACTAAACGATTTGGAAATTCTTGCCAAGTTAGATATGCAAAATTTAA

>JAP01453.C|Supercontig_0000058:710185-710440

CTTGATGAAGTGGGTATCGAGGTGCATCGGCAACATAAATATCTGCACCTGCTTTAATAAGGATCTTGACCATTTGTAAATAACCTAATTCAACAGCCAAATTGAGTGGGCAAACTCCATCGATGACCATACTTGGATCCACACCATTAGGATAGCTGAAGAGATATTGAAGTGTACCAACATTATTGCTTCTTATAGCTCCACGTAGGAGCCATGGGATCTGGGATTTACTTATACGAGGCTTGTGACGCTTTTT

>JAP01459.C|Supercontig_0000061:200929-201033

CAAATCCGAGATAAACCATTCCATAGTTGACAGCCAAATTTATTGGACCAAATATAGACCCTGTTGCTGCTGGCATCATGGAAAAAACACCAGACAAGAAGAAAA

>JAP01461.C|Supercontig_0000136:59898-60061

AAACAATTCTTAGCAGCTTGTAAATTACAACCAAAAGTTTCAAGTTTATTTTGACCAGATTTCCCAACTCGACCCCAACGAAACCAGACTGAGTAATTATTTATACTTTCATCCTTCAAAAGCTGTATAATATAGTATTTATTGTTATTATTTTGAACATTAGT

>JAP01473.C|Supercontig_0000238:346013-346235

CACGTAAAGATGAAAAACCTGCTACAGATTCACGTTCAGCAGCTACTATACGCAAACGTTGGAGTTGGAGGTCTAATAAAAAGCCATCTGATGTCGGTGGTACTACAGTGAACCGATCCAGTACTATGCCTACAAGAACTAGATTACAACCAACTGAAGAAATTCCTGAAGACAGAAAACATGATGACATTTTTGATAGTGAACATGATGAAAAGAATTATTC

>JAP01486.C|Supercontig_0000213:563361-563655

GTGAACCAAAGGATTGTATACTGCTTAATCAACCATGTCAAGGATATGAAATATGTTCTCGCATTAAAAATCCAATGGTTTACACTTCAATCAACCGTAAATTGGATGCATCCGTTTATGACCTTTCCAATTCTTTTGAAGATACAATTCCACAGTGTATATGCCCTACATGCCCAGAATATGGTCTAGGTGGGCAGGTATGTGGATCAGATGGACAAACATATCGTAGTGAATGTCATTTACGATCAAGCGCCTGTCAACGACACTCCACAGATCTAACTGTTAAATCAAGAGG

>JAP01506.C|Supercontig_0000613:65538-65704

CCATCTGGTTGACGAATTGATAAACCGGAACCACTACAAGAAGGATCAAGTAGAATTGATTGAACATTAGAAAATTTTCGATGATATGGATCTATAGATAAGAAATCTGAACAATATGCTTCAATGGATGGTTGTGAACTTTGAATAGATTTTTTATGTTTACTATG

>JAP01508.C|Supercontig_0000269:798085-798242

AAACCTGTTGTTACTCCATTTGTACCAAATAATCTACTGTATGCAATTTGGTTACACGCAAGTCATTTAGCTGGTTATGAACAACGGGATGCTCATGAATTTCTTATAACATTGTTAACTTTAATACATGGTCATCTTGTTGGCGAGGAATCACCACA

>JAP01513.C|Supercontig_0000465:451867-452005

CCATAATTCCTTTGGGTCCAGGATCACCTGGTATACCAGGTATACCCCGTTCACCTTCTGGACCAGGTGGACCAGGCAATCCACGTTGTCCAACAGGACCTGGTCTACCAACATGTCCACGAGCACCAACGTCACCCTT

>JAP01520.C|Supercontig_0002033:84088-84258

ACAGATGGCCCAACAAAATTAGCAGTTGCTTACTGTAACACAGAGTTTCAATCTCAATCATTATCGAATGATTCATACATATGGGATTTAAACAATCCCAATAGACCAGAGTTGATACTTAAACCAGCTTCTCCTCTTGTTTGTATAGAATACAACCCTAAAGACTCTCAT

>JAP01524.C|Supercontig_0000654:93798-94645

ACGCCACTTTACTAGCGGTGATGTAGCACACCAAAAATTAGATACTTTGTCTTCGTAAATACCGCGATCAAACGGAAATAAACGTCGAACGACTTGAAATAAAAGACTCAAATCGGTTGTTAGTACAAACGGTGCAAATATCAAAAAAATTGTCAAGAAAACGACAAAGCTCAGTTTAGCTAGATGTGTTAATCCCCTGTACACGGATTTCTTATAAATACATCTGCCTAATAAATAGAAAAAAATTGGCAACGCATGATATAGTTCCATTTGTTTATAACCTATGGCTAAACAAAATAATATAGAACCGAGCATATCCCATTCTAGGAGGAGGAAATTGATTCCGCTTAAATACAAACCAAGACTAATACAATTATACTGGAAGTGCCCATGGTCAATCAATATAAGACCAGGATAAGTTAATGTTAGGCATGCTGAATAAAATCCACCAATTTGTTGTGATTTATTACCTTTATTCATTATGCTAGGTAGAACATAGTAGAAATATAGTAATATGGACGGTATAAAAAACAATAAATCAGCTACCAATACAGTATAACGCATGAACAACTTATGCTCTTTAGATTCGAAACCTCTCGATGTGTACAACTGAACCCAATCACGGTCAATTTTAGAAGCTAGTTTACCCATCAGCCAACTATGATATGCCGTAAGAGGTGGATAATCTAACCCCCAATAATTCAAATCATTATGGGTTGAATTCATATACCATTCCGTGAAAGGTAAATTGACGGTGATTTCCATCCAATGACGTTGAGCTTCATAGTCACCATACATAGGTGGTTTATTTTCACCGGAATATGAATGTAAACTGGTAGATGAGCGCA

>JAP01525.C|Supercontig_0000442:218678-218985

GAAGATATGGCATTCAGTCCAAATTTTCGACAAATTTCATCATACGTCAAATCTTTACGTTTTGATAAAATTATTCGTGTGGGTTTAGACATCACTAGAAGTGCTGCTGACAATGCCTTTTTTTCTAATCGATGTAGGCTAAATGGGGAAAAACTGTTTAAGATGGGTACTACAGTTTATATAGGTGATAAGCTTGATATAGTGATTGACGATACAGAAGGACCTGTCGGTAAAAGAGTGCGAGTTCTTGATATAAAACAATTAAAACAAAATAAATACAAGATTAGTTTGCGTTGTTGGAGAAATCA

>JAP01536.C|Supercontig_0000007:328929-329510

TTTCAAATCTTTATTGCTCATCCTATATGGAGTTGGTTTAAATGCTGTTTTTCCGCAAAATGCAGCTGAAAGACGTGCTTCACCTAATATATCAAAACCAACAGCTAATGGAGAACCCTTATGGACCAACAAAGGCCCCGTACCGCTGACACACGCTCGTCCAGGATGTGTAGGATGACTTGTTAATCCAGTTATTGGATTGTTCGCTGATAAGCCAAGAATCTTTTGCTTTACATTTGTCAATATCACTGGGATTGTACCACAATCACGCAATGCTTTTACTAAAATACAGTCATCTTCTGCACCACGATTAGTGCATATTGTATATCCCATTGTATGGTCATAACCATGAATAGGAAATATTTCTTCCAGTGCTATAGGTATCCCATGAATTGAACTAACATGTTCGCCTTCTGAATCCCGTGAGCTATCCGCTAATATAGCGTAGACGTCAGCTTCATAAATAATTTCACTAATGCAGTTGGATCTTGTACGAATTAATTCTAAACCACGAATTTGATAAGCGTCTAAAAGATCGACTGACGTAATACTTTTATTATGTAGCTTTTCACGCAGTTGACT

>JAP01546.C|Supercontig_0000000:2327848-2328167

GGCATAAATCATGAAAACCAGATCGTAAAGTATTAACCCAAATATCCAATTCATTTTCACTAATAGCCTGTAAATAATATGTTCGATGGCCTGAGGATATTACTTCAAAAACAAATCGACGATCTGTAGAATTATTCAATAAGTTTATAGGTTTCTCTGCAGCTGTCAATCGTTTATTTGGTTGGGTAATCCGGGCCGTACAAAATCTAAGATCACTTACCATAACAGTCCATTGTGGGTTAGCTTGACAAAGTAACTGTGTCAGAAGACCACTTTCAGACTGTTTATTTACAATTCCATTTGTCAAACGATCAGTATTC

>JAP01553.C|Supercontig_0019027:38141-38292

GCTTTGTTTGCTTCAATTGTTGATTGTACATCATCAGTTACATTTATTACCTATTTGAATGGAATGCCTCAATTGTATGTAGGTGCGTTACATTTCGGTGAAGCATTTAGTGATTTAGTTCCAAGTACATTAGCCCTTATTCAAGGAGTTGG

>JAP01561.C|Supercontig_0000007:448468-448593

TTTATTCGTTTGGTGTATGGCTGCTTTGATTCACATGATAAGACGACATTAGCTTGTAATAGCCATCTAACTAAGCATGCTGTTCCTCAAGCTATTGAATGTTGTAACTCTAGTAATATGTGCAAT

>JAP01565.C|Supercontig_0000020:927358-927459

AGAATATCTTACTGGATTTCGAAAAAGAAAGCTTGAACGTAAAGAAAGGGGACGCAAAGCAGCAGAAAAACAATTAAAAGATGAGATTAAGGCTGTTAAGGA

>JAP01570.C|Supercontig_0002060:285219-285456

ACCAACATATCCTGTTGGACAATGACAAAAAAACCTCATATTTGGTTTGTTTTCACAAATACCACCATTTAAACAAGGATGAATAACACAATCATCAATATCTTCCGTACAGTTTTGTCCAGTCCATCCTTTGAGACATACACAGTAATAGTCTGACAAAGTGTTGAATTTCTCTAATGATGTTGTTCTACTCCAATCTTGAATATTTACACACTCAGCACTGTTTTGGCATGGATTC

>JAP01573.C|Supercontig_0000478:416580-416760

TACAGTACAAACTCAATTGAAAATCAATGATATTGTTGAAGTTTATGGTATATTAGAACATGCACGTCTTTGTGATGCATTACCAGAAGATTCATGTGAACCAGAAGAGAAATGCAATGAACCTTTACCTAGAGTACATGCTCTTATAATTCATTCATTAGCACATAATAATCCTCTTGTT

>JAP01581.C|Supercontig_0000241:243726-244145

ATATTCTTCTGTTTCTAAGCTTCCTTTACTTATTTGATCATCACCATTTTTTAAGAAAAATTCAAGTAGTATACCTAAAGACTTTTTTAATCTTTCCAATGTAGATTTAGCTCGAAAAATTTGATTTTTATCTATTTCCATAGGTAAATTAGATATTAATTCATGCATTTCATGTGTAGATAATGGAATAATTGTTGGACTGTTATTATCTGCATTAAAATTTGATTTAATTTGTAGAAATGCACCAGGACCACCAACATTAGTTGAATTAATTTCACCTTCTTTAATTGTCTGTTCCATATACTGTTCAAGACATTCGTGCCATATTGATAGTATACAACGATGTAATAAATTACTAGACACATTGGTTTTTAATGTCCGTATATTTGAGTCCAAATAAACGATTAAATCATGCATAGC

>JAP01587.C|Supercontig_0000270:178022-178227

CCATAGTCAGTTCGTGCACCACATGTCCGTGTGACTTTTATCCATGAATGCCAACCAGTGCTTTCTTGATAAATTTTTACACAAAATGTTCCAGGTGAGTGTGACAAAAAGCTACCACAATCTATCATTGCATTAAGTCTCGTATCAAAAAACCTTCCGGCTGGACACGAATCATCTTCATTGCTATCGCACTGATAACATCGAAT

>JAP01588.C|Supercontig_0000201:36557-36707

AATTTACGTTTAAAACCAATAATTGATAAAGATCCAACACCAACTATACTTGGTCTATGGACTCCTATCGTTGAAAATGTAATTAGTGAAATGGGACGTTTGTCAATTTATGGAGATTCGGATTGTTTAAGTTCTACGCATTTAAATTCAA

>JAP01596.C|Supercontig_0000461:73448-73547

TAGCATCAAAATGCATATACAGACTAGCCGACGACTATTATCCGGATCTGTGAAATCTACTAAATCAACACAAGAAGATGAGAGCATCCATGAACTTTCG

>JAP01598.C|Supercontig_0000277:155220-155345

GACTTTATTGATTGTTTTATCAATGATAACTTACTTCGGTATCATCACAAGTTGTGCCCCCACTTATATTTGGGTTATAATATTACGACTTCTGGTTGGATTCGCTATTGGTGGTGGAAATAGCAG

>JAP01599.C|Supercontig_0000212:189559-189763

CACTTCACTGTCATCTGTGAACAACTTTTTGGAAACATATTCATTATGTCACATGAATGAGTCAGAAAAGACAATAAATCTGTTAGGATGCAATACAGTTGCCAGTTCCTCATTAGAACAACAAGCGTCATTTACCTTAGATAACTCTGATGAAACACAGAGTGAATTCAGTTTAGTATTTGGCCAGTCAAACGAAAATATGAAC

>JAP01600.C|Supercontig_0000007:2320277-2320726

TGTTTATGCATTGCTAATTGACAACTAATTGCAAATAAATTACAAAGAACACTTTTTGATTGATGACAATCAATGATACATTGTGGTACAACAACACTTATTCGATAAGTTACCGTATAATGAAATGAATTATTTAATATATTTTTATCCATATCCGATAGTAATTTTGACATTAATAAATGTATTATATAAGGTAATTGTAAAAGATTTTTAATGTCATTAGAAGATAAACATTTAGACCAATTAGTTTTGATTACATCATGAAATAATTCACCTAATTTATTTAGATTTACTGCACCTTCTAAACCTAAATCCCAATTAGATAATTTACCATTTATTAATAATAATAAACGACTAATATGTACAGCGACATGAATTCGTGCTTCACAACTAATTTCATTTAAATGAGGTGAAGCTATCACATTAGGATGTTGTAATAATTTATCCATA

>JAP01614.C|Supercontig_0000139:324350-324580

TGTAGCCGGAAAATCATAACTTGACATTTCTTGCCATGAAAAATCCCAATATTCTTTTTGACTTGAATCAAGATGCTTATGTTTTTTTGAATATGTATTGCCTCTGCTGTTCGCTAGCCACACGTCATACCCATAATCAGCCAAGATATATCCTAAACTCTGATTTTTCAAATTCATAACCCATGCATGTGACGAGTCCAACAATCCATGTTGCAATAAAACAACTTTACG

>JAP01617.C|Supercontig_0000456:241162-241288

CATTGAAAATCATTCAACACTTTTCGGTTTTCTTTTATATCTGGTTGGGCTGATATGGTTTTGTCTTCTGTCTCAAGATGAGCTCAACTATAAAACTTATATGAGTGAGAATGCCCTACTTGTTGGT

>JAP01624.C|Supercontig_0000136:453339-453589

TGACAAAAGTATTCTTCAGCTATTCTTGTTGCCCATTCTTTACATATAGATAATGGTCTTGTAGGATTATTAACATCGGAGCATTTAATAATTAAACGTTTAATTAATGTTCTATTTTCTGCAGTTGATAAATTTTCTAATGTACTAGATATACGTTCTTGTCCAGGACTTGGTGAAGTAGAATGTGATATTGTCATTCCCATGGAACATGATTCCATAGAACTCATACTACCAACAGATGAACGGTCATG

>JAP01626.C|Supercontig_0000462:104364-104651

CTGACTCAAGTCTTCCATTCCTTCAACTGAAGAAGCCAGTAATTCTGTGATAAATGCTAAGAGAGCTGGACGTAAATATTCATTGACCTCAGCTAGTTCATTCACTACTATACAGTAAGGTTTCATGGTGTCCATATTTAACTCGAAATCAACGCGTTTCGTATGAAAAACGACCCGAACAAAACGAAATACACCAAGTGAAACGGGTCGGCATGATAAACATCTCAAAAGCGATGGAAGATCTCGGAAAGCACGTAAACTAATTCCACTGCCAGACATATTGTTCCA

>JAP01642.C|Supercontig_0000012:1455375-1455580

GCACCATGCAGTTGCTCTAATTTAGCATATTGTTCGCGTTCCAAATATGAACACATATCTCGTATTAATGATGCATAAGCGCTGTATTGTTTTAAATTTAAACTGCTTTCAATGGATAATGGGATAAGATAAGGCAATACTTTATTAGCTAGTTTTTCTCGACTGATGCCCAATTTTTCATGACTAAATGCTAAACGATATATACC

>JAP01651.C|Supercontig_0000145:479986-480103

CAACATTCACTACAGTATTAGCATATGATTTGGGAATGCTTGCTCATCATTGTACATGTCAAACCGATGCAAATCATCCTGAAAATCCTCAACGATTAATTTCTATTTGGCAAAGACT

>JAP01656.C|Supercontig_0000299:75170-75363

CATCAATTCTCCCGGTTGATATTCGCTATCTGTATCTAAAACAGTAGCATAACCATCTCGAGTAAACCGTTTGAAGCCGTAAACACCTTTTAGACGTTCTACACAGCGTTCCAGAGCTGGCAATCGTGTACTAGTTGAACATGTACCATAAGCTGGCCAACATAGTGCAGGAATAAGTGAAGCATCAGTACCCT

>JAP01658.C|Supercontig_0000295:490100-490266

AAAGCCCAACGTAACTCTCTTCTTGAAACCCATCCAACTAGATATGGGGAATCTTGTGAAACAACAACAGGAAATCCTTTAACATCACATCTACTAACTAATTGGTCCAGATCGCCAACTGTCATATCATATTGTGTAACAACATATAGAGGAGAATTAGAATCTCT

>JAP01667.C|Supercontig_0000021:2822480-2822909

GGTTGATGTAATCATCTCTGAGTGGATGGGATATTTTTTATTTTTTGAATCAATGCTTGATTCTGTCTTAAAAATGGCATCGAAATATTTATCCCGTGATGGACATATATTTCCACGTCATTATACATTAAATTTATTAGGTGTACAATGTTCCGAACAATTACGTAAACGTCGTTTAGAACATTGGAATAATGTATACGGTTATAATATGCCAGCATTACGTCGTGCTGCATTGAGTGAAGCACATGTATTAAATTTAACAAATGAACATGTTACACCACCAATATCCCCTATTACTATATTAACACAATCATTTGAATTAGTTGCATTGGATTTAGACGATATGCATCGTAATCGTATTTATAATTTATCAAATCATTGTTCACTTTTATGTGAGCAAAAATTTCATTTAACTATACAACCTACTACT

>JAP01681.C|Supercontig_0000053:651921-652101

GGTATCTCATAAATTATTTCCCATTTGATCTTCTGTATCGATTTTGCAAAATACTTCCTGTTCGACTACTGGTGTGCTCATTAAAGGAGGTTCAGAGGGCGAAGAAGATTTTCATTGGTGTACATCATGGTCTTCATCTGTTTCCACAATCGCCTGTGATGTGTGTTCTCCTAGGTCTTTT

>JAP01684.C|Supercontig_0000050:879709-879812

GGCTAATCTTCGTGTATGTGAATCGAATTGGTCAGATAAATTTTCATTGGATACAGTTGGTAGTTCAGGACGTGTACATTGTACAACAAAATCAAGAATGTCAT

>JAP01686.C|Supercontig_0000134:73343-73462

CAGGTGACCGCTTCGAGTTTGATGAGAGTGGAGATACATTCCTTTGCTTCTTGACTGCATTCTACACTTTAGTTTTAATCCCATTGACTTATTTCTGTTGGCCATCTCTTGAATTCAAAG

>JAP01687.C|Supercontig_0000196:180723-181126

TGAAATCTAGATAAATTCAGTAGTTTACTTGTTAAATTTTCCATAGAATCTTCTATGATATTACATGGATTATTGGAATCGATCGAAAGTGGGAAAGAAAAATGATGTCGACGAACTTCTTTTGAAGTGATATCAATGCCATTTGAATGATGATAATGATTTATTTTATCCACACGTATATCATTCATAGTTTGAGTGGATCCTGTAATAATCTGTAATGACTGTATAGCATGACTGGAATCATCTGACTGGATAAACAAAACTTCATTTATTTCTAAATTTTTTTCTATATCACTGGAACTGCCGACTACATCATTTCCAGAAGATAACTGTTTCATTGTTTGTACAATATATGAGGGAGGCAGTAACCTGTTTAGACGTGACTCCAAACAATTACGAACTGC

>JAP01688.C|Supercontig_0000304:203526-204522

GTAGATACAGATGGTATAGCTGAAAGTGAAATGATTGAACAAATTATAGAAAAACAAAACCCTCCAAAATTACAAAAAACTAATTTTGGAAATTTACTTGGAACATCATCTATCACTTACCAATTAGAAAATGAAGGTGATGGTAAGATATTTTCAATTAATTCACGCAATGGAGATATACGTCTTAATAAACATTTGGATCGTGAAATTCGAGCTCATTATGAATTTCGTGCTTTTGCAATTGATGATATATTAAAATCTGTTAAAACATCACAAAACAATCCATATTTAATCAACAAATGGCAACATCAATATACAGCTACAGCTACAATAATTATTACAGTATTAGATGTTAATGATAATCCACCAATATTTGAAACACCATTAAATGGTCAAGAATTTCACATTGAACCTGGTTCTTCAATGACTACAGCTGGTAGTACATTATTCACAGCAAAAGCACATGATCCTGATATTGGCGATAATTCATTAGTTAGATATTCTTTAGATAATAATGGTTATGGTATAGTAGAAATTGATTCAACAACTGGTGTTTGTTATTTTCGTGAAACATTACAATATTCATTAATGAATAAATTAATTTCATCTAATCAATATGCAATCAATGGTAGAACATCAAATCATTTAACTGATTCTAATTTAATTACTTCAGATAATTTAATAACAAATCGTGCACATGAATTACATTCATTTAGTTTAAGTCTTACTATTATTGCTCGTGATTTAGGTACACCATATTCATTAAATAATACACGTACAGTAAAACTTGTATGGACAACAGAATCCCAAATGAAATCATTAAGTATATCAAATAATGATTTAATAAGTAGTAGTATATTTGGTAATTTTGAATTTTTATCAGATAATCGAATGACTATTAATAAATTAATAATTCCATTAATTATTGGTGCTATTCTATTATTATTAATAATATTTTTAATATTATTTGGTATATTTCATTGTCGTAAAAATTCAA

>JAP01689.C|Supercontig_0000373:421460-421778

AGCCTAAAATGGAGGATGAAGTAAGAGCAGCACCATGGAATACAACACGTGCTTATCTTGCTTCACAACGTGGTGGTTGTTTTCTTGAATTACATGGTGCCGCTGATCCTACTGGTTGTGGTGAGGCGTTTTCTTATTCGAAAACCTCTGCAAAACCTGGTGCATTGTTTCGGCAAGCCGGTGGTGAAGTTGCAAGAGGTCTATTAAAAGGGAAACGTACTGTAACGGGAACTGATGCTGACCTTCGTAAACTACATTTACGCGATGCACGTGCTTTATTGAGATCATTTGGGATTAGTGAAGCTGATTTAAAAACTTT

>JAP01694.C|Supercontig_0010910:519-758

AGGATTTTGCATTTATTCTCTCTGGAATAACTCGTTTATTAAATAATCCATTAGTACAAACATATCTTCCTGGATCTAGCAAAAAAGTTCAAATGCATCAAGAACTTTTAGTGTTATTTTGGCGAATATGCGAATGCAATAAAAAATTTATGTATTATGTGTTAAAATCAAGTCAAGTATTAGATTTACTTGTACCTATACTCTATCATCTTAATAATTCACGTAATGATCAATGTAAGT

>JAP01699.C|Supercontig_0000285:164170-164321

CGTAGTTTATCAGCAAATTTCAATACACTTGTATAGAATGAACACCAAGGTCTTTGATCCATAGTAGATGATCCAGTTGGAGGTGGTTTCACGGTGAAGTTAATACCCATAGCTAAACGATTAGCCCGGAAGTATTTAAAGGAGTCGTGCTG

>JAP01702.C|Supercontig_0000141:432536-432687

GTAGTTAAAAATCGTTACGATGGAACTTTGGGTTGTATGGATTTGAATTTTCGTAAAGATCGTCTTTCTTTTCGACCACGACCACGTGAACTAGTAATCCCAACAAAAGCAACAACATTTCCAAGAATAGTTGGTTACGATTAAATATTTTC

>JAP01707.C|Supercontig_0000208:25311-25595

GTAAATGATATGGCTTTCCATCCAGTGCATGGCACACTAGCCACAGTGGGATCTGATGGATGTTATTCGTTTTGGGATAAAGATGCTAGAACTAAATTACATTCTTCGGATTCTCCAGATCAACCACTCACTTGCTGTGTATTTGATCCTAAAGGCCAAGTGTTCTGTTATGCGTCAGGTTATGATTGGTCTAAAGGATATCAGTTCGCCGATCCATCTAAACCCATCAAAATAATGATGCGCTTGTGTATGGAAGATATGACTCCTGGTAGAAAATCATAATTT

>JAP01713.C|Supercontig_0000670:189672-189851

AGATTGGTCTACTTTCGGAGAAGTTATTCTACAAAGACCATGGCGTCGTCGAACCTTCAGGTTACCATCTCAACTTTCGGAAGCTGATAAGGCTTATATACGAGCTACTGCAATCGAACATTTTGATCGAGTCATGTCAGTTCTTGAACAGATGCCACGTCCAATGCTTCTGTTTATTAG

>JAP01714.C|Supercontig_0000256:114847-114954

GGGTTGGGAAGAGATGCGTGAGCCATAGCAGCAAAATGGTCAAGAATCTCATTACATTCTTTGTGATAATTAGGAGAATCATCGTCTATTTTAGAAAACTCTACAAAC

>JAP01722.C|Supercontig_0000106:949720-949936

TATTTTAAATTCTGATTGTCACACTGTCAATGGCCATTCCATAGGTCAACGCAGTATAAGACAACAACGTGAAGTCGCTCATAAAATTGAACATCCTAATCGTTTACATCCTGATATTTGGCATAATGAAGCTAACGAGTTCAATGATGGTCCAGCTTGTTCTTGTAAACCGAAATATCGTATTGGACCATTACACAATCAATACGAAGGTGAACAG

>JAP01730.C|Supercontig_0000036:287647-287787

CCTGACTTACAAAAATTCGGTGTTACGACCATATCAAATATATCGGCTAACTCATCGAGTCTATGGGTGCATACAATATTTGGCATTGGATTTTTTCTATTGGCATTTTTTCTAATGCAACATTTCTCGAGGCAATTTCGT

>JAP01739.C|Supercontig_0000447:315537-315782

ACAATAGCACGTGCAGCAAAATCTAATCTATCCTCTAAAGTTAATTGATGACATGGTGTAACAGCTAAATGTTCCAACACTTGAGCTGCTTCTAAACGTGCACCACGACGTTCCAATAAACGCCATAAAAGACAACGTAAATTAGCATCATCTGGTGTTTGACGCAGTCTTGAACGTAGATATGCTTCAAAATGCGCTGAATTTAATAACATCACCGTATCAGTAAGACCATTTGATAATAACCAA

>JAP01746.C|Supercontig_0000463:129599-129806

GTTAGTGATGATGAAGCTAGACGTCGTGCTAAACGACGTGAGCGTAATCGAGTCGCAGCGGCCAAATGTAGACAAAGAAGACAAGATCAAATTGAAGAGTTACAGCATCGTGTTGATGCATTGACTAGAACAGGACAAGAACTTCGTTCATCTCTACATTCATTAGATTTAGAACGAGCTAGACTAGAAAGTCTTGTAGAAGAACATA

>JAP01757.C|Supercontig_0000287:212666-212795

ATGAATTATATTCAATGTGGAAAGATTTACCACCAGCTGTTATGACTGAATCAATTGGTCCATTAGAAGATCTTTGTCTTATACCAGATGATCCAATGATTACAAGTGTTTACTTGAATTCATCTTCATC

>JAP01768.C|Supercontig_0001992:2805-3147

ATGATAAGACTCCTGTTACCACTTCTCTATCACTACCTATACGTAAGATAAGCAGCCAGGATTTGGAATTAACCTATGACAAGCGTATTTTATCTTCCAACGAAGATGGAAAGCCCACATTTATAAACTCTGTGTTCTTTCCTTTCCCTTCCAATGAACACATCGATTCAAATACTAGTTCTGTTCGTCAAACTGTGGCTTCGCCAAGTCGTCCGACACATCTAACTGAGAGAGATTTGATAAGTGAACTTGCTTTAGATATTTGTCGTGAACTTGACGTGACATCTTTATCCTTTAAAATTGTGCAGAATGTTTGTAGACTAATCAACGCTGATCGTGGATC

>JAP01778.C|Supercontig_0000150:503919-504345

AAATGTCTTCTTTTCAAAAAGCACTTGAAAATCTTAATGAAGGTACAATATGTATTAGTGTATGTGTTAGTGTATGGCTGTTATTAATTGCGGCTAGGATGGATAATGTCTTAATAATTCCTTACTGGACAATATTTCTACCACTTTGGTTGTGGAAAACAATTGTTTTTGTCGGCTGGTTAGTTGGTTTAATAGTATGGTGTAAACGTTGGAGAATTCGTGTCGATGATTCAGAGCATAGTACATTTCTTGTTCGTGATGACCCTGCTCTACCTTACATTCAAGCAATGTCTGTATCAGCATTTTTTCACTTTCTTATCACATGTTCTGAAGTGTTATTATGCATTCATTTATCAATTGAATCATCTAATCTTACTTATTTAACTATTTTATCACCATTACTAGTTGTTGGTGCGTTGGGTGTGTT

>JAP01781.C|Supercontig_0000042:721584-721811

ATTCATATCTAAAATGCATCGTGTAACAATTTCTAAGAGTAAAACACCATTATTATCTTGTCTTTTCATCATATCTCTAATAAATGATAGTAACGCCCAAAGGCCACGTGGTTCGTTGGCACGATAAGAGCGAAGTATACAGTGAAAATTCTCTACTTCCGTGGGTGAATTAAAACTTAGTGAGCCAATATCACTGTATAATACAGGTCGTGGAGCCCAAAATCTACG

>JAP01782.C|Supercontig_0000153:299135-299357

AGGAAAAAATAATGCATTTACGTCAAACTGGGGCACGCTTTGAAGAGGAGCGACGTCTCAGTCGAAGAGCTTTGGATGATGCTGAAAATCATATCACACAACTTGAAGTAACTAGACGATCATTAGAAGGTGAATTACAGCGACTAAAGATGTGTATCAGTGATAAGGAAGCCGAGAATCAAATCCTCGAAGACAAATGTCAAAACCTTTGCAAACAGATACA

>JAP01793.C|Supercontig_0000153:309355-309624

TTTTAAAGGAAAGACAAGAACTATTTGACAGGATATCGTGTTTACAGCGTAATTTAACAGATGTTGAAAATATGCGTGATCACTTGATGAGATCAAGTACCCATCTAGAAAAAGATAGGCATAATCTTAAACGACATTTAGAAAAAGCCGAAAGAGAGAAACAACAAAACGAGGAGATTGTGAATAAGAATAATCTTGATCGTACAGAGTTAGAAATAACATTACGACGTCTGGAGGAAGAGAATTCAAATAGAAGAAAGCAGATTCAGT

>JAP01800.C|Supercontig_0000165:229542-229739

ACAATTTCTGAACGACGTTCATTTTCTAGTTTCCGTTGACGTTTTTCTTCTTCTTTTAAACGTTTAGCTTTTTTTAATTCATCTTTTGCTTTTTTTCGTTCAATTGATTCATTACGAATTTTTTTTATTTCTGATTCAATTTTTCTACGAGTCTGAAATGAGGTACGTATACCTTTGTCCTTCTTAATTGCACTATGT

>JAP01818.C|Supercontig_0000061:760281-760454

AGGTACACCAGTTAATCGTATACCAATTATGGCTAAACAAGTTTTGGATTTATATGAACTATTTCAACTAGTTGTTGCACGTGGTGGTTTAGTTGAAGTGATTAATAAAAAATTATGGCGTGAAATAACTAAAGGTTTAAATTTACCATCATCAATTACAAGTGCTGCATTTAC

>JAP01826.C|Supercontig_0000077:1347300-1347427

ACAGTCTTCACTTATATCAGTAAAGCCTGGTATAAATCGACTAAGCCAAATGTGTGGTTGCATACACGCTGATCTGAAGCGTTTTGCAAACCACGGAAATCGGGTATCTAAGTAAGCAGGAACGGGAA

>JAP01832.C|Supercontig_0000259:101881-102137

TCACGTGTAATACAGTTTCATTGAATTATTTTATTTTTGGGTGAAACTATGACTAATTATCAAAAGAATGTGGGAATCCAACCTTTGAAAACATTTTTAGCTCGTGCAATATATGATAATCTGTCAGAAAATCCAAAAGAATTAAATTTTTCTCGTGGTGATTTAATCACAGTTTTGGATAGAAATCCATCTGGTTTAAACGGTTGGTGGGTATGTAGTATTCGTGGTCAATTAGGAATTGCACCAGGTAATCGTCT

>JAP01834.C|Supercontig_0001914:60448-60632

TATATTAAATAATCTATATTATGTACAATTTACAATGAAATTAAAATAACACGTTCACTGTACAGTTGCCTTTGCAGTTGCCTGAGCTTTTATTTTACGCATACGATGATATTTTTCAATATACAGCTTTACAAATGTGCTTATTTTTTCTGTATTGATTGAAGAAGTACCACTTCGCGAAATCT

>JAP01835.C|Supercontig_0000152:164822-164976

TAATCCAACTGAAACTGAAGATTCACAGACAGCAAAACGTCTTACTACTGAAAATCTTTCTTTAAGAGTTGAAGTTGAACAATTAAACAAAGAATTAAAACTGCGTAGTCAACAAATATGTACTATGTTATCTGGGAATTCAAGATTGAATCAAG

>JAP01837.C|Supercontig_0000063:1901138-1901457

ATTGACAGTTCAGTCCATTCTATTTCAGGACGTGTAGCCCGTGGTATAATTCCTGGATCTGTGAATGCTGTACGAAGAAATGCAGTAACAACATATAGAAACTGGATTGCTGCTAAAATAGGAACAGCGGGAGTTAAGATTGGGGTTAAAAGACGGCAATCAAAAGCAAAGAAAAGAACTGTAACTGAGATAATTAAAAGAAGGGTAATAGTGAATATACACATATCCCGAGAAAAGACCCCTCTGCCATGGCAACAAAATCTGTTACGTCCAGGATGTAGACGATAATAAGGTATACGAAGACTCTGATTATCCTGCAT

>JAP01839.C|Supercontig_0000462:432127-432252

TGTAGCGGTAAATAAATCCCCAAAATAAATAGTTCTGTAGGTCTTCGACATTGATGCTCACCCCATTAGTTTTACCGGACACATCTAGCAGAAGGTGCTCGGTCATGCATCCTCACCAGATCACGA

>JAP01844.C|Supercontig_0001914:40979-41150

GTAATAATAATAACAATCAACATAGTTCTGCAGCCATGTTGAAAACAATGCAGTCATATATGAGGACTCTTCCACGACCAATTGCAGTACGATTAGATTCAAAAATAGTGTATAACGAAAAGTCAAAAAGTTGCACTAATATGGATAATGTCAAATTATTAAATTCTAATGA

>JAP01858.C|Supercontig_0000009:449154-449302

TTGGCTACCTCTCATTGTGTCCCAATCGCTCTACATCTCAATCGTACATGCTTAGGTAATCATTTGCCTTACAAGCTGACAGCCCCAATACCACTTGGCGAGGATGTGACTTTTTACAGTCTAGAGTTTTGGACAGCCCTACAGTCTAT

>JAP01859.C|Supercontig_0019075:49407-49514

AAACCCGTTCATAATTACGAAAGCATGTAAATAATTTATGTCTGGCATGAAATTCCATTTCGTACAGAGAGAATAAATTACTTGTTTCTTTGTCAACACATTTTTTCA

>JAP01872.C|Supercontig_0000155:961139-961324

TAAGTGCTTTCAGTTTAAATAGTCCACCACCGTCTACAGCACAAATTGCTGAACATTTTAATGCAGTACCGGGAAAATTTATGGAAACTGACTTAAGTAGATTAACTCTATCGGATATGGAACAGAAACGTTTATACGAAGCAGCCAAAATTATACAGAAGTATTATCGAGCTTATAAAAAACACA

>JAP01876.C|Supercontig_0000680:34566-34835

TTCTGAATCGGACATATTCTTAAGACATAAAACTAACTGAGAGAACTTTGCCTCTTTTACTTTTTTGTTACGCCAGATATGAGGAATATATAATTGTTTAATAAATCCAATTGATTCAAAATACATTTTTCTAGTGAACTTAGTATAAACAAAATCATCACCCCATAGGAACCAATTAACTGCAGGAGCTAAACGACGCTCTATAGTTGAAATTAAAGCACATGATTGAACACCCTCAATGTCAGATAAGTCATATTCTAAGCCATAATT

>JAP01902.C|Supercontig_0000412:41114-41458

CAGGGGAATATGTAAAGCGTCTATCCCGTGGCTGACCACGTTCAACTTAGATAGACGAATGCAATCAGATGAATTCTCCCCGAAGTTTATACAGCACTTTTCCCGTTCAAAACTTGCCGACGCCCAGTCTATGATCTTCTCTACATTTGATACAGATCAGAATACACAAACAAAGTAACCTGGTAAACAAGGGTAGGTTCCACTCCAAAGACATTGTGAGCGCCATTTCCAATACGACTGCCGACGTAACCAGTAACAAAACTGTGCACACACAGAACATAGCGCAATGAGATCGTTAATCCGCAAATAGGAGCAAATTTGATAAAATAAATCCTGAGGTAAATC

>JAP01903.C|Supercontig_0000115:57973-58103

AGGAGGCTGTTTTTCAATTAGAATCGATTCATGGTTTTATGTTTTCTGGTCAACAAAAGCTGAAGGTACGATTACTGCCTGACTCACCGCACTATTTGAATTATACACTACTTCCATTGGTGTCTGGGAAT

>JAP01904.C|Supercontig_0000072:585281-585642

GAATTATATATAGATGGTATAGATACATTTTGTGAGATCATTATAAATAATCATTCATTAGGAGTTACAGAGAATAGTTTTTTATCTTATACATGGAAAATTAGTCATTTATTAAGTTTGAAGCAAGCAAACAAATTAGAATTTAAATGTACATCAACTGTATTGATAGCAAAGAAAAAAGCGGAACTAATGAAAGTTAGAAAAAAACTTATTCCACCACCATTTTGTTGGCCATCTAGATTTCATGGTGAATGTCATATAAATCTTATTAGAACAACACAATCAATATTTGGTTGGGATTGGGGGTTAACTTTACCGATTCAAGGATTATGGACATTACCTCAATTGGTGATTTCACCTTT

>JAP01916.C|Supercontig_0000159:1170079-1170320

GTTCGAAACCATGGTTTTTCTGTATAATCTCGATAAGGATTTAAACTCATATCAATTGCTTCTGCTGCTTGGGTTGCTAAAAGTGGTTTATACGCATATGGTGCAAATAGATCCCATGTTGTACCATCATGATCAGTAAACATTTGATAATCAAAAAATATTCCACTTGATACAATATCAAAATTTGATAAAACATTGGCAAAAACTTCACCAAATAAATGAAAACGATTAAGTGGAAAACC

>JAP01919.C|Supercontig_0000040:515224-515434

GCAGAAGCTTATGAATATATGACAACATATAAAGAAGTTGTAGCACGAGTAGGTCGTACTGAAAATGTTCTTGGTTGGTATCATTCTCATCCAGGTTATGGATGTTGGCTTTCTGGTATTGATGTTAGTACTCAATTAACAAATCAAACTTATCAAGAACCATTTGTTGCTATTGTTATAGATCCAATCAGAACAATATCTTCAGGAAAAG

>JAP01932.C|Supercontig_0000132:936670-937299

AGCATGTGGTCGTACACTTCTAGAACCAGTAGGAAGTTTGTCTTCACCACAAATGGCATGGAAATTAGAAGATCGTTTTGGTGGTAATGCCAACTCTTCATTATTTCCGGACCCGATAGGTTTAGAGCAATCAATTCAATTGGGAAATGCTATATCAGTTGATCATCTTTTACCAGATGATAACGAGAATATGTATCATCCCTATCGATCATTAACAAATGGTAATTCAATTGATGGTGGTGAAGATCATAGTTTAATCGGTATGGTCAACGATAATAATTATCAGTTAAAAACACTTCCATATAATCCATTTAAAGGATCATTCAATAATGAAGAAGTTCCATTGATAGCAATGAGTCGTTTATCTAAACTATCGAAAACAAGTTCATCATCTACTTTAGGTTTCACATCAGCTAGTCCTATACTTTGTCAATGGAGAATAAATGCTGCATCAGGTGAACGGATACGTTTAAATTTTACACACATGGATATATCTGGACCGATAACACAGGATTCTATACATACACCAAATAATTTAAATGATTTACACAAAGCATACGAATTAAATCAAAAAAGTATCAATCGTATCTCATGTATTAATGATTATGTAGAAATAAGAGATGGATATTA

>JAP01941.C|Supercontig_0000008:148714-149054

TGTGGGAAGAGGGCTTCCAAATCGTTTAGCAGATGTGAATATACTAGCGACAACTTAATCAGACGAAACCGTGCATCTGAGTTGATATCGAAAATTTTCTCTTTGGAATTTTTGAAAAGACTAATAGTTTGCTTGGATTTTTCAATCAAAGTCGAAATATATATTTGAAAGTATTCTATTTCCGAGAGTGCATCATAGTTTTCTTCGTAATTTGCAATTATTCTTTGTAGTTTTTCATATATATCCGGAATCGTATCCAAAATATAAGGCGGACTGGCTTTCAAGCTCAATTTTGGTTGTTGGCAAAGACGTACAACTTTGTCAAGATATTTATAGCAAAA

>JAP01942.C|Supercontig_0019079:89377-89570

AGTTAAATTTAAGTTATCCAGAAAGTCGTCAACATTTGTGGAGTAAAATGCGAGCAATTCTTCGTTATGTCTATCAATATCGAAATGATTATGATTATTTTTTAAAAGCAGATGATGATACCTTTGTAATCATGGAGAATTTAAGATCTGTTTTACATCAGCATAATCCTAAAGATCCATTTATGATTGGTTAT

>JAP01947.C|Supercontig_0000485:439301-439479

CCACTCATAATAACTGAATCATCAAATATATACGCTTTAATATGTTGTACACCGAATATTTCATTCCAACGATTTGGTAACATGTAACGGATCCATTTGTATGGAAATGGATGTCTATACAATAAGATTGATACATTAGGCAAAGTAGTTAGTGGAGCTAAAAAATGACATGAAGAATA

>JAP01980.C|Supercontig_0000008:1083992-1084116

GGAATAAGTTCACGTTCTTCCGATTCTAATTCAACTGGTTCTGCACTTGCTTTAATGGACTCCATAGCAGTACAATAAGCAACAACACGTTTTTGTGAATGTGTTTTATGTACGCCGCGTTGAAA

>JAP01994.C|Supercontig_0000378:578912-579145

AGAGTTCGGAGATTTTCTGTGATGAATTGACAGGGCTAAACATTATTTTATCAGCAACTCATTCTGAAAATCTCTCAAGGGATAATAATGCTGTTAACGTTAGCAATTATGTTCGATCTGAGAATTCTTCAGTGTTTGTTCTTACTCGGATGGATTCTCGAAGTATTTTCGAACGCAGTGGGTTTTCAAGCCAAGGTGTCCTTCCTAGTATAGCTGTTCTGATTTCTGTTGCCG

>JAP01998.C|Supercontig_0000173:455387-455757

GTTCTTAATAAAATTAAAAATTTTCTTCATGAAGAAGATTCTGATGGAAATACTTGGAATCGTCTTTCCAATATTGCTAAACGTAATTCCATATGTAATGGAACTACTACACATTATAATAATAATGATATACTTGGCTTAGAAAGTTATACACATTTTCATCGTTTATGGTCTGCTATTCAATTAGTATTTTGTACACCATTTGGACAGAATGAATATACAATTGAAGAAATGTTTGGTGAAGGATTAAATTGGGCTGGATGTGCAATCATTTTATTATTAGGTCAACAACGTCAATTTGAAGCACTTGATTTTGGTAGTCTTATTTTACGACTACAACGAATCGATAAAAAAGATGTGACACCAATGGG

>JAP02001.C|Supercontig_0000264:242550-242707

AAGGGGATGATTGAAAAAGGAGCTCTAGACTATGAAGACCGCCCATTGTGGTACGATGTGTATAAAGCATTTCCTCCACGTGTTGATCCTTCGTATGATCGACCATGTCCCACTACCACAGTGCAGAATATTATCTATCCAGAAGATTGTGAAAGAGC

>JAP02008.C|Supercontig_0000305:796530-796958

AAATTAAGCGCCAAATTTTGGACGGATTTTATCAAAATAAATCTTTTCTTTATACAATGCAGAAAATCTTGAAAGTGGTTGGTGTGAAGACTGTCCTTGGAGAGCATTGAGTATTGTATGGAATTCTGTACGTACAAAGTTGTAAAACTCTTGTTCAGCTTGCCAAATCGGATTGTCTTGATATATTTTAGTCGTTGCCTTACTAATTGGTAATTTATAATTTGTGCGTCTTAGATGCCATTTATCTTTTGAACGAGATATTAGATTATGAGCACCGGTAAAGAATGATGGTAAGAGTAGTTCTAACAAGTTGACAAATTTATCAAATTCTTCTGTCAGGCCAACAATAAGATAATCTTCAATCACACGACGTTTAGCAGTTTCTAAAGCAACTGGATTACCAGGGATTCTGCAATACATTGCTTGTCC

>JAP02014.C|Supercontig_0000194:635778-635908

AGCTCCCCAAATATTGCTAAACCATTCCACCTGGGGCATTTCCGAGCCACTGTAACAGGGAATTTTGTTAAAAACGTCAATGAAGCTTTTGGACATAAAGTAATTGGAATAAATTACATTGGTGATTGGGG

>JAP02018.C|Supercontig_0000066:229780-229918

TACCTCGCTGCAATAGATATCTCCTTTGAAATTCTTGTGGATGCCCCTGAAATGGTCAAAATGGAAATGGGAAAGGAAGTAACAGGTACATCCAGGAATTTCAGCATAACAGAAAGCATCAACAGTAATTGATGTATCT

>JAP02026.C|Supercontig_0000048:1249076-1249214

TTTCAACATCGTTTTTCGAATTTTGAACAATAATTATATGAGATATGCAATTCGCCATTAATTCTTCTTCATTTTTGGCATTATCAAAACCCCCAACATACAAACCAGGTAATATTTTTGTCATACTGCCACCCATTTT

>JAP02027.C|Supercontig_0001992:37356-37607

CGATTGGTCTGGAATATTTACTTATTCGGATGAATTTTTATTTGAAGCATTCGCTTTATTTGTTGGCTTGGGTATTTCAAATAGTCAACTGTATGAAAAAGCTATACGAAGTGCAGCAAAACAAAAAGTTATCATGGATGTTTTGTCGTATCATGCTACAGCGCCGACATCTGAAGCTAAACGCTTAGCTACATCATTAATACCAACTATGAGATTTTATCATTTAGATAAATTTTCATTTACTGATGTACG

>JAP02028.C|Supercontig_0000103:77992-78089

GTTGTTGACGTAGAAGTAGTTGGTAAAAGTGCTCGATTCCAAACTGTTGCTTTAGCAGTAAGAATAGCTATACATTTAGCTTCATCACAGGAATTATA

>JAP02035.C|Supercontig_0000380:113402-113644

AATTATTACCTAAACGATTACCATACAGCGTTAAATTGCATTTACAACCAACTTTACGTCAACCACCAAAAATGACGAAACAAAATGTCGAAACATTCTTTCAACCAACTGTTAAAATTCCAATACCAAGTTTCACAATCCATCCAGATTTTACTTCAGAATTCTATAATGCACATAGAAATTATATGATTAAAAAAGATCTTTGGAATTACGCTACACGTAATTATTCACTTGCATATTAGA

>JAP02044.C|Supercontig_0000012:735311-735397

TCAGGAGTTTCGAATAGAACACGTTTGGATGGGTCCTGCTGTATATAGACATGTTCAACACATTAGTAATGATGATTATGAATCAAC

>JAP02051.C|Supercontig_0000054:895339-895652

ATGGTCACCTGATCCTCTAATCAATGGTCCTGCAAGATGTGGTTGTTTCATGGCTACTATTCTAGCTATATTCAGTATTATATTATTACTTATTGGGATAATTTTAACTATACTACACTATGTCATCGGTATGGAATTTTATACAGTTAATCGTGGTCAAACTGTTGGTCCATTATTATTAGGCCTTACAATTATACCTCTTATATTTATGATCTACTTCATATGTATAGCTAAGCATAAAATAGCTGATTATTTTGAACAGTTAGCATTGAATTATCGTGCCAAAGAACGAACAGCTTACAGACAATATCAGG

>JAP02064.C|Supercontig_0000122:1392325-1392413

TTCATTTCCATTGGCTCAATTGTTATGAGTGATTCAAACTCGGTGCTCTCATTTAAATTCAGATGCTTTTTATTCCAATCCATATTGTC

>JAP02065.C|Supercontig_0000046:1630169-1630412

CGAAAATCAACGAGATAGTGTTCACACCTTAATTGCAAGGTATTACATCCTACGGACAGTGGAACAAATACTTTGAACATTGATTCGCATAAAGTCCACTCCATTTCCCAATCGCTTTCACGATCATTGTGAACAGAAACAAAAACTGATTGGCATTGACAATAAGGGTCAATATTTCCCTTTAACTGGAAAAGTGGATAATTTACTGTAAGTAAATCATAAGCATTGAATATATTGAACATTG

>JAP02069.C|Supercontig_0000293:451983-452052

CGTGCAGCAAGTTGTTGCAATTGTGAAGATAATTCAGACATAAGATTGGCAGTTTCTACGGAGACCAAAT

>JAP02071.C|Supercontig_0000322:162771-162941

TGATGACGAATACGTCGATGTTCATTACTGTTTGTTTTATTTTGTATATTATCGTAAGAATCGGTTATTGGTTGAGATTCATTTAAGCTACAAACAGTTTGTTGACTAGTTACTGATTCCAAACTACTTAAATCATTCGATATTGATTCAACAATTGGTCGAGGTCTTTCA

>JAP02075.C|Supercontig_0000436:35901-36025

GAGTATACTGGTATTAGACAATCATTAAATCTTCCATGCTCTTATATTTCAAGTTTGTTCCAATGGTTCACATTTTCATTTCGGTTCGTGGGGAATTTAGTATATTTATCTGTATTGGATTGTAG

>JAP02077.C|Supercontig_0000235:761495-761709

AATCCATATAAACCAAAAAGTTTGAATGAATTGGAAAATAAATCGTACAATCCATATTGGGCAGATATATATCAAGGTTCTGCATTAACTGGATTTGTTTCTCTTCAGAATGTACGATCACCTCATGATTTGACTTTGTCCCCCACAAATGATATTATTTATGTATCCGAAATCTATCCATATAAGGTTCATCGATTTGAAATCCAAAATGATTC

>JAP02080.C|Supercontig_0000313:308406-308568

CATATGTTTTTCGAATAAATAATCGACGAAATGGTTCTACAATACCTGCTGTACTAAAACGTGTTTTTGATTGACGATATACACGATTTGGTCGTTGTTTTATATAAATTGGATTACGAAAATCTGTAAGACCAGTCATTAAACGATTCCAAATACCATTGTT

>JAP02081.C|Supercontig_0000153:960001-960189

TCTCTACGAACAAAACTGGATTGTTTATATAATATATTACATAATGAGTGTCCATCAAATGCAGTACGACTAATAATGAATTATTTTAAGGAGACATTACCTGATGGTTGTACATTTAATTATGAAACAAGATTATCAAAACATTTAGAATCAGTACAAATGACTCAGTTAATTAATAATGATGAAAAT

>JAP02087.C|Supercontig_0000111:166494-166638

CGACATATCACCGCATTCCTCAAATTAACAAGAACGCTTTTAAGTTCACAATCGCATGTGTAAAATATATTTAGAGGTCCATCATAATCCACAGAATGTCTTTCAGAAAGTTCCGGTGATGCAAGTGCTGCATTTTCCGACATAG

>JAP02092.C|Supercontig_0000362:44547-44809

TGGTCATGCTCAACCATTTATACAAAGACCATTTGCACCATTTTCTCGTGCTATTCAACAATCTGGTCTATATCCAAATGGATTAACTAATGAACCAGGTTCTTTAAATCGTTTATCTTATACAGAATTAGAACAATTACTTATATCATTAACAAATGATTTAGAAACAGAATTAAGAAATTTAGCTGTACGTTATCGTCATAAACGTCAACCATTATTAGATGCAATTGCAGAGAAAACAGCACAAGCATCAATGAAAACAA

>JAP02094.C|Supercontig_0000059:274160-274474

CAGTTGGAGTTTGCAAACACAAATTTCTCAAACTCCCCAGATGCACTCCTGTACAGAAAACTAGTTAATCCGTTTCATCAGTCTGTAAATTTGGATTATGCGTCATCTAGTTTCAAACCGAATAGCATTGTATTTGACAGTGAATCCGAATTTATTGAACATGTTGAAGATAAAAAGGATAGTGTTTGGCTTTTAGCCGTAGCTTCTCCACAGTCTCTCTCTTCATCAATCGCGGGAGATCATACAAGCACAACTCCATCCAGAATAAAACATTCATCAGTTGTTACTGAGCAGATTTGGTCTGTTTTATGTTAT

>JAP02096.C|Supercontig_0000160:643633-643754

ATCATTCATATGTTTTAATTGATTCTGCTTTTCAACCAATTTATTTCTTGATTGAACTTGAGTAACCTCCAGCTGATTTAAAGTAGCTAATTCTGAGGCTAATTTGGTGCATTCTACTTCTA

>JAP02106.C|Supercontig_0000442:270525-270598

CCAACTAAGATTTACACCATTATTAGACTTGAAAGACACGAAAGCGTCATTAACAACGACTGCGCCAAACGACT

>JAP02110.C|Supercontig_0000379:272362-272970

TAATATTTCACAAGATTGCACTGGACAGCTGGGACCATTTTGCAGTAGTTTTGAAGATAATACTACCGAATCGGATTTATTACATACATTCGAATTACCGATATCACCCAGTCAAGATTTATTTGGTGAAACTACAACATCAAGTTCCAATAGATATTCTCCTCCTATTGGTCGGACAACGGTAACTACTCCATCTTGCTTTTGGAAAAACTGCGAATCTTCTGTTCATCAAGTTTGCGTTAATACAGATAGTGGATTTTCTAGTTGGACTGGAGGTGGTGATTCTAAAACCAGATCCCCATCAAATTTGATTTCATCTTACTGTGCCTTACCTCACTCAATTTTTTCACCACTGATATCATCCGATCGTGAAAGTGTTTTTATGCAGAATCAATCTGATCAAGCTAATTCGCCTTGGAGGCTGACACATTTAAACGAATCAGACATAAGCTCTAGAAACTCTGTTACCAGCTCAGGTTCATGTCTAAATAATTTACCTTGTGCTAGTGAACTTAATGAAGAACTTTCAGATAGAATGTTCGTTACAAGTATAAGTGATATGATAAGAAATTCTCCAACTTGGTCTACTCCATGGACTTGTTCAAATAA

>JAP02117.C|Supercontig_0000030:797409-797541

CCACTTAAACTTCCACCGCCCACACAGATACCAGAACTATCAAGAATAGTTCCACTTAATAGATTGTTTTTGTATTTTTTCGGTGCATCTCTAGACTGATTTCTCGTAGTACTGTTTCCGTTGTTACTTATCA

>JAP02120.C|Supercontig_0000132:363214-363362

AATGATATTATCTTTGAAAACACGTGGAGATATAGGTCATTTCCAGGTATAAAAATACCGGTTCTTCGTTCCCCTTATCCAACTTTTGGATCACCATGGCCATTACCATGGAGTTGGTCATCATCTTCATGTTATTATAATGTGGATAT

>JAP02124.C|Supercontig_0000282:452321-452662

ACATCGCAACATTTTCTTCTGATACATTTGTAAAACATTTCCATCCAGTTTTAGCATGATGTCGAATTGTAGTCATTAAATTAACAACAGCTTCAGGTCTAGGTGGACGAATTAATTCAGAATTTGGTATCCAGTATACAGCATTCATCCATGCCTCTTGAGCGCCATCAATATTTACACATTTATTTGGTTGTTCATTATTCAGTACAGGAATCCAATGTGATTGTGAAGCTGGGAGTAATAATGCATTTACACAAGATGTAATACAATCAGCTACAGTTGAGGGAGAATAACCACCTTCCAGTGCTAAAACTATACGACTATTTGATATAGACATACACT

>JAP02129.C|Supercontig_0000227:649562-649690

ATTATCATGATTTTTACCATATTTTGAATTCCATGAACTAAGAAATGGTTGTTTCGATGCACCTGTTAAACCAATTCTTCCAAATATATGCAAAGGATAAGAATAAACTGTACTATTCACATAATAACA

>JAP02147.C|Supercontig_0000024:1249459-1249706

TCTCAACAACCAGCTTTCAATGCATATCCCCTCGAATCTTCACCTAATTTGCTTATTCCTACAAATGATGTGATATATATGCCATTAAATCCCACAAATGTGCCTTTATCGCTACAACGTGCATGTGCCTCTAATGAGACTATAGGCGATGATACACAAAGTTATGGCCATCCAATATGTCATGGCTTCATTTTTCCAAACTCAGAAGTAGGTTCAAATGATCCTGAATTAAACCAGATAAACATTCC

>JAP02148.C|Supercontig_0000089:186258-186419

TTGTAGTATGATTTGTAAAAACCATCTTTGGTTATGATTCGACGCATTATCGATTCAGTTTCTTTACTTGTTGATCCGGAGCCTCAAAATAATATGGAACTACTTACAAATAAGGATTACAGTGTAGACGCTTCTATGGACGATGACTCTGATAACGTTAAC

>JAP02150.C|Supercontig_0000153:38848-38965

TGAATTTCTAGAAAAGCTTAATGCTGCTAAAGACAATCCTCCTAGTGGATCAACTTTATATCAAGATGATAATCATTTTTGCCCGGTTCTTTTTCCTGATCCTATATTCAATTGGGAA

>JAP02156.C|Supercontig_0000005:203674-203819

TTATCTACTGGTTTGTCATATGTATGTTCTTTAGCAGGTGAACTAATTGGAGTCCATGCATATAATGTCCAACCTGGAATAAAACCTGAGGCGAGATTTGAATTTCTTTCTAACGGTCTTGTTCGTTGTGTTTTGTCAGGTGAATT

>JAP02157.C|Supercontig_0000043:460462-460873

TGAATGGCACTAACAAGTGTTGTAACTTCTTATAAAGTCGGAATGTCAGGTGATATCATACCAAATAATTTCGATATGATACCCTATTCCACTTTTCATACAGCTTTATTACCACATCCAAATCCATTAGGACCTGGGAATCCATCAAATATTTCAACAGGTTTAATGCTTACAGCACAACAACCAAATGGACAAAATCCTTATTGTCCACCAATAACTCTTACTAATGGATATTATACAAATGTTAATTATCCACATAATGCAGAATCATTTTGTTATGTTCAACCAAATGATGCTAACTCAATATTGAATGCATATCATACATATCCTACATCAATAAATTTACCATTACAAGCAACACATATATCATGTGATAAGTTATATACCTCTAATAATCATATAAGTAATAACA

>JAP02161.C|Supercontig_0000088:142934-143139

TAAACCAACTCCTTTATTCGAACTGGGTTCAAATTTTTCAATGAGTGATGTTAGTTGATTATGAACAGAATTTTCCGTAACAGGTTGAGATGTAGGTATATGAGTTGTTCTAGATGATAGGGATAACGTACTCCACTGATCGTAACCACTTATTGAATGCACAGAGGAGACTTGACAACTAGGATGATTAGAAAACGAATCTGACA

>JAP02166.C|Supercontig_0000375:469214-469451

ACTTTAAATTTCAAATCTGCATCAGTTAATTCTTTTAACGAATGATGATCTATACTTATACGTTGTAAATAAGGACTTTTTGAGCAGATTACAATGTCCCTATTCTCCATTTTTTCATAGATCTCTTTAGTTTCGATTTCTTTAATACATGATTGCGTAAAAAAGCTTGGTGCAGGTTGAGGTAGACTATTAAATAAATATAAACTGCCTAAACGAATGGACTGATATTGCTTTTCTA

>JAP02167.C|Supercontig_0000035:237488-237734

TTTCCAGTTTATTTTATCGTGTGGTACATTCATTCTAATTCCAGGGAATATTTCAAAATTAAATGGAACTATACGCCATAGTCGATGGCTTAGAATGAGTCGTTCATAAATTTTACCAGATGCTGGAGCTGCACGTGGACCACGTGATGGTGGTCCTTTACGTGGTGTTAGAACTGTATCTACTTCAAGTTCGTCACCATTACTTCTTGTTAAAATAGCTTTGACGCATTGTTCTTCAATTCTTAAC

>JAP02177.C|Supercontig_0000159:171148-171440

GATTTTGTCAGAATGCCATCCTTGCGTGATTATCAAGAAGAGGGACAGTTTGCACGGAATTGTTCTTTACTATGCAATTGTCACGTCGGTTGTCGATATGAAATGTGTTATGGAACAGAGATAAAAATTTGGCATGCTTATTCTTTATCTAATCGTACTAGTCAAATTGTTACTTTTCCAACGCGAATACAAACAATCAATATTTACCAACCAAATTTATCTTTAGGTTGTCTTTATCTACCTAAATTTAATCGTCACTGTACATGTTATGCAAATGATCATGTTGTCTGTAA

>JAP02179.C|Supercontig_0000018:1198155-1198390

AAACATTTTATAGATGGTTCATACTTTTTGTACCAGTGAGAAAGGAAGCTACACATATCCATCAATAATTCGTCATCTTTCTCAAGGTGATTGTTGAGGTGGAAATGATGTGTGTTTTCTATTCTAGGGCTTGGATTTGTAAAATTTGATTCCTCGGTGAAGATTTTTACATGATTTGTATGTTCAACTGGAGAAGTTTGATTTTGCATAAGTTTGTTATAACTTGTGAAATTTAA

>JAP02186.C|Supercontig_0000009:1397154-1397268

CGTTCTCTTAACATTGGTAATTGTTCACGCCAGAACATACAATAATGTCTACGTAAATTCTTACTTATATATGAACGTGGCAATGTAAAAACCATATGTTTACGTGAATCATCAT

>JAP02188.C|Supercontig_0000327:125163-125468

CAAGAAAAGAAGTACTTTCATCAGCTCCAACATCACCAAATAGTAGTGAGAATGAAACACCTGGTCCTATATCTTCAATTAAATTTCAATATACAAAAGATTCAAGAAAATGTTTTCGAAAAATGGCATCACAAACAAACTGGCGTAAACGTCAAGGAATGACTCTACGTTGTGGTCCATTCTTTAGTCGTACACATAATGGACTTATCAATTCACCATATCAATCAGATGTTAGTCAATCAGCACCAAGTAGTCCTACAATTGATACATATGAAACATTACCACTTATTCCTAATGCATCAACAA

>JAP02189.C|Supercontig_0000036:743820-743927

GTATCAAAGTTTAAAGGACGTAGATAGGCTAGAGGACCTAGGAGATCACACCATGGTGAAGGAAGCAAAGAAACTGAATGCTTTAAAATGGGAGTAATTTTCTTGTTT

>JAP02201.C|Supercontig_0000171:292188-292426

TATTCACTAATCAGTTTGGTAGTGATCAGTGATCTTTTTGCCAGTTACCTGTGTTCATCTGTTTTGGCATCGTCTAATTCAGGTGTCAGCTTTATTCAAAATGATTATGATCTAGATAAAACAAAATGTATACAATCACTTTTAGTCAATCGACATCAAATTCCGGACTCAGCGTTCAATGCTACCAGTGAAGTAGTGGATCCGTCAGGAGCTAAACGTTATAATGCGCATTCTATTCG

>JAP02203.C|Supercontig_0000144:386094-386346

CAAAGCATCCGATCCACATTAACAGTGCACCCCATGCAGAACCAGATGAAAATCCATAAGTGTCAAGTAGTGGACACAGAAAGGGTGCAAAAAATTGAGGCATTGAAAAAATCATTCCAGGTAGGATGGCAGCTTCAACACTGCTAAGATGGAAATCACTCATGAGTTCCGAATACAATATACCCATTGAGTAAGTGTATCCATCAGCCAGCATATACGAAATAAATCCACCAAAAATTAACAATAACTTCTG

>JAP02204.C|Supercontig_0000153:861853-862086

CGAAATCATGGATTACAAAATCGAGAAATTGATAATGATGAATCAGAATTTGAAAGATCCCCTGGACCAAGTGATAATGAAATAGGTCAAGATAATTATAACAGATCACGAAATATTCCTCAAGTTCCAACACGTAATATACGACCATCTTCTTTACAAAATCGAAATATAATTCGTAACAATAGTAATTCTTCTATTCATTCTTATATTGCAGAAGATCCTACAATAATCTAA

>JAP02206.C|Supercontig_0000020:1217096-1217248

CATTTTGAAGAAACATTGGCAGATCATTTAAAAGCACTCAAGTTATGTGATCGTTTATTAGATAATCTGGGTTCAATTTATCATCGTGTACGTCCTTTAAAAAAAGGACAAACTATTCTAGCTAGAGCTTTAACAGATTTTGAATCAAAACAG

>JAP02209.C|Supercontig_0000083:55941-56157

GAAATTTAGTATTGGTGTTGTTGTGACTTCTTTTGACTCATTATGACATTTACATGAACACATTTGTCCACTTATCATTACATGGTTATAACGGCAATCAATACAGAAATTTTGTCCATTCATATTATTTTGGAATGATACAATTGAACAGTATGGAGATACTCCAGAATCATTACTAATAGAATCACTAGTATTTTGTATAAAATTAGGACTATAT

>JAP02211.C|Supercontig_0000085:1216001-1216284

TCTGACAGACAATCGGACAAACTTTGGAGTTTATCCACAAGAAAGATCTAATTCTAACTGGTTCAATGATCAAGCCAATATTAACAAATTTATGTGACATACGTCTTGCACATGAATCATCAAATTTAACTATAGTTGAAGAATTCAAATCAACAATTGTATCCTATTTAATGAATTCTTTCCCTAATAATGGTGCCGTTTATGAAACATTATGGATTGCCAGTTTATTGGATCCACGTTTCAAAAGTCAAGTGCAGGATAAAAAACCAACAGTGATTAAATTG

>JAP02213.C|Supercontig_0000123:1453269-1453516

GATGATTTGATTAATGTTAAATTTGATAATTCTGAATTTACTTCAGAACTTTTACTCAACATTTGTACTGAAGGAATTAATTTCAGACATTCAAGTTTATCCAACGGAACAGTATCATAAGCATGACAATTATGCCTAGTCGATGTAGCTTTTGGAATTTTTGTTTCATATTGACATCCATTCAAATTGAATGGTTGTAAATTAGTTTGTGATGAGTTGTTTTTAACAGATGATCGGTGAAAACTGTA

>JAP02214.C|Supercontig_0000067:802470-802809

CCTGTGACTTATGATCTGAGAATCGATCAATAATAAAACGTGCTATAAAGGATAATAATATTAATAAAAGAAATTTAGTGGAATATATTATCCATGATGAAATTTTTGACGATTGATTGATAAAATTTGAATGATATAATGTGGATGGTATATCCCAATTTATAAGTAGACCACAAGTAATAAGAATTAATGGTGGAAGCATAATTGTTATTCGTTGACGCCATACAGGTGAATAATGTAGCATAGATAAGCCTAAACGTGAGAAACGGTGCGACGAATTCATCATGTTCTGTACAGTGGCCATTTGTTTGACACGATCTGTTAACCATGGGACTACACG

>JAP02218.C|Supercontig_0000453:164148-164248

CGATTATGATAATGAGACAGAGCTGTGTCTTTTGTAACTTGACAAATCTTTTGAGTAAATATTAAGTTCCGTTTTCCCATATAACGCCGCTTCTTATGTTC

>JAP02227.C|Supercontig_0000215:46957-47095

ACTGGTGGAAGCTTCTGTGCACCTGCAAACCAGTCTTTATCCATTTCGACGAAAAAGCTTTGTAACATATCAGCTTCTTATCTTCTGGATATTTCACCTGAGGGAGTTCGTTGTCCACTTACTCCTGAGGAATATACAG

>JAP02230.C|Supercontig_0000018:1332987-1333058

TACGGGAGCTTATCATTGATGGTCGTATACCGGATATGTTAAAAACAATATCTAAAAAGATCGTCAAAGAAA

>JAP02233.C|Supercontig_0000088:130356-130544

AGAATGCATTCACTCAGAAGAAGTCATGAACAATTTAATTCAAGGAATTTTTCACCTCATTTATCAACTTCCAGTCCTGTACCTGAAGGTCTATTTAATGAATTGGATAAAGATGAAGATCGTGAAATTGTTGAATCAATCGAAGCTGTTATGAAAGCAGCATCATATGATTCAAATAGTCCAAAAGAA

>JAP02234.C|Supercontig_0000453:336276-336356

ACTTCATCCAAATTTTCGCAATTGGCAACAGTTGGCAGAATTTCCTTCAATCGTTCACATTCAATTTGATGTGGTTTTAAA

>JAP02235.C|Supercontig_0000098:55774-55998

GATGGTGTATGTTGAAGAAGATCCAGAAATTGGTGCAACTATCAAACAAACATTAGATGCAAATTTCCCTGTACATGTACAAGATTTGCTTAGTTTAGAAATATTGGCTCATTGGCCTAAAGAACCACAAATGTTAATGGTTGTAGAAGTATGGCAATTTCGATTGGATATTATGGAACGGCTTAATACTACTACATTACAGTTTACAAATAGTCCTAATCCAAC

>JAP02238.C|Supercontig_0000290:738150-738606

TGACTCTTTATTCTGAATTTGTTCAGAGTCCACAGACTGGAGCTATGTCTAAACAATCACCAAAGTTATTTAAACAACAACGACAACAACAATCTCATATTACATTTAATCATAGTAAACATCATAAAGATAAATTAGACGATACTGATTCGGGATTTTTAAGTAGGGCTACAAGTATTGAAAAATTATCAAATAATAGTGTACATGATACAATTTCTACAGTATTTTCTATTGAATCATTACAATCATCAGTTAATGAACATGATACCGACAAACGACAACAATCAGAACGCAGAAGTTCATCAGGACAAAAATTAGCAAAATTATTAAATGTTTTTACACCATGTCGAAATCGTATTGCACGACGGAGTAGATCATTTGGTGATATAAATAAAACTTTAAAATCTAATCATAAATTTAACTGGAAATTTAACACTTGTTCATCACAATTGAAGGT

>JAP02240.C|Supercontig_0000225:302179-302269

TCATCATTACCCAATGATTATCATTGGATTGATAGTGGGGTTATTGTGGAACAATTAAATTTTATGTGTTGTCATTGGTATCACGTTGATC

>JAP02241.C|Supercontig_0000085:815664-815940

GCTCCACCTACATTAAATAATCTCTTTAATAAAATTGATGAATTAAATAAACTTGTTGATACAGATAAACCAATTGCAGAGTGGAGATCTTCCTTACGTCTATCTGTGGAACGGTCAATTCTACATGCTCATGTTTGGCAAACGGATGAAACTAAATTGCCTCGTCGATTTCACCAACAGTCAAATGTTTGGAAACATAAACCTGAGTATGGAATCCATCCGGAACAGAGTACTCGTTATCTGTTCCACAATCTATTTCGTATTTTGGAATTTCAAG

>JAP02247.C|Supercontig_0000055:516383-516522

ATCATATTATTAATTGTTTTGTAAATACGTTCTGTGAAGTTTTCAGCTGATGAAAAATTCAATGAACCTAGAACACGTATTGCTTTGATCGATGACAGTAGATAATCAGATTGAGTTTGATCTATTTGTGTTTTGGTTAA

>JAP02251.C|Supercontig_0000096:1430385-1430646

CATTATCCATTTGTATACGCTTGGACATTGTATTTACATATTGCTGAACAGCTTTATAAATAAATCTATATTGAGTTTCAGTTTGAACCATCCCAGAACGTTGTTCACGAACTGCTTGGATTGTACGGGAAATATCTATGTCACAGTTAAGACCCATTGTTTTGATATAATTTATAAGCATATCGATTACAATAAATGCTCCAGTACGTCCAATACCTGCACTACAGTGAACAACAATAGGTCCAGCGCCTGGGATACTATC

>JAP02262.C|Supercontig_0000009:489866-490032

CATCTCATCCATTGTCAGTTTATTCAAATATCTTAATGCAAAGTAAATGTTCAACATATATTAGTCCAGATGATGGTGAAATATCTAGTGTTAGTCAAAGTGCTAGTCAAGTTGTTCCATGTGCATCATCTCCATCATCTTCATGTACAGACTCATCATCTTCTTCA

>JAP02269.C|Supercontig_0000235:794954-795134

AGCATGTGCAGCTCTAAGTACAGCAAGCATTGCATAACCAAAACGTGGGTGCATACAAAATCGTGCTTGTCTTCTTCGAAGACCTAATTTATTAGCAGCTTTTTGACATTCATCTGGAGTAAATCGATCAGCTGTTGAATCCACTGGATGATCTGCACTTTTTGGTAATCTGTATATACCT

>JAP02274.C|Supercontig_0000476:42909-43021

TGTTCCATGACCTCTGCCACTGCGTCTTCCTCTACGACGATGAGTACGGATTTGCATAAGCCGTGAGTTCAATTTCGTAATAGGTAAGGATTTAACAACATCATAGTCGGAAT

>JAP02276.C|Supercontig_0000079:108762-108888

GAATTATTCATAGACCTCCGGCTCGTATTCTAATCGTATCAAATGGATCTAATCATTTAGGTGCAGTATTGTCGTTGAATGTAGCTCGATATTTATCTACACTTGGAGCTTGTGTTCTATTAGTCAC

>JAP02279.C|Supercontig_0000461:95019-95119

CCACTGTACATGCCAATTCCTGTAAAATTGAAAATATTCTCATTGTTGAGCATTTCTAAAGCTAGTCTTTCACTGAGAAAATAATGTGAAGATCCTGATAC

>JAP02287.C|Supercontig_0000488:288744-288878

TGTCAAGTAACCAATGAATTAGAATTTGATGCAAGAGTTAGTACTGATTTACGTGATGTTTGGCCCACTGGATTAGCTGTAAATCAATCGAATGGAGATATTTACGTTGTGGATAGAGATAATTCTAGAATCAAG

>JAP02297.C|Supercontig_0000157:802745-802986

CGTCATTTAGATGCTACACGTGAAACCATTGCAGATTATGAACAAACATTAGGTAAATTTAGAGATTTAGTCGCTGATTTACAAACTCAAAATTCTGATCTTCAACGATCATTAGCTGATGGAAAACGTTTACAAGAACAACAACAACAACTACATTCAACAGTTTCTACAGAATTTGCTAGTTCTGCTGTTTCATTTATGGGAACCAAGTTTGGTGCTACTTCTCAAGCACAAACTCTTGC

>JAP02298.C|Supercontig_0000019:1829698-1829852

TCCTTGGAAACATGACATTCAGTCCATATCCTAGCCAATGTACAATAAATATCACTTTCATGGCAGGTCAACAGGTTAAGTTCATATTCATATGCCATATCAAGGATAACCTCTCTAATTAATTCATATTCAACAAGTTTGGAATAGTATCCCAA

>JAP02307.C|Supercontig_0019075:44686-44761

GCTACTTCATTTATAACAGCTTTCCAAGTTGGTAGAGTATCATCTAAAGTGTTACGATAAACTGTTAGACCATTAG

>JAP02315.C|Supercontig_0000096:973817-974503

CTGATCCAATGTTGCCTACTTCACAAGGAAAAACGGCATTAGATTTAGCATATGCTGCTGGTAGATCACAGATGTTAGTTGATGTATTGCCAAAAAAACAATTGATTGCCTTACTGATGTTAATCGATGATATCGTTCCACTAAAAGTGAGAAAACGATTATCCGAAGAGGAAAAGCTAAAGGAACGTACTGGATTAGGTGGTATGACAGCTTGTGAACCATACGCTGTAATGAGTGCCAAAAGAGAACCCAGTCCAGGTTTAAGTGCTGATATGCATAAAAGATCTCGTAAAGGTTATTCATTTATTCGTCGTTTAGCTGATAAAATGGATACGACCGATGTTAGCAAAGGTCAGTTGGGAAATTTCACAAAAACTGTTAAAGTCGATAGTACGAATATAATGAGCATATGGCGTAAGATTTTATTAAATGATAATGTAGAGTTATTAGAAACACTAAGAGATTCTATGGCCTGGCGACAAAGCAAATCGAACACTTCTACACTTCCCATGGATTTTAGTAAAATAAGTGATAGTATAACGATGCCTACAGATCACATTTCATTAGATGATTTTGAAACTGCACAACAAATTAGCCTAAAACAAATGTCAGAACTATTTTCTGATTGCTATGAAAATGGTACTGGTGTTTTTACACACGTTCAAGGTGCATTTCAAGAAAATTTAA

>JAP02317.C|Supercontig_0000115:386747-387042

CCTTTTGTACCACGTCCACGGAATATTAAATTTGGATGTGATTTTGCACGTTCATTATCAATACAATGTTGTCCATCATACATTAATTGATAACCGGGTACACATTGGCAAGTGAAACTACCAGGTGTATTTTCACATATTCCATAATCTGGATCACATGGTCTATGGCGTACACATTCATTAATGTCTAATTCACATTGAATACCTTCATAGCCACGTGTACATTCACATAGATTACCAGGTCGACAAATACCACCATTTTCACAGGGTGGATTGCAAGATACATTGTCTTCTTT

>JAP02325.C|Supercontig_0019089:82813-82978

GTTTAACTGAAGAAATTCAACGTCTTCCTAATCAAGGCTCATTTGTTACTAAATCTGCTTTACAATTTCAACCAAATGCTGGTTTCTTATTATTATTAGTTATAAAATTAATAAATTGTCCATATTTTGGTCCAATGCAAGAAATTCAAAAACTACATACTTATTT

>JAP02327.C|Supercontig_0000272:232763-233116

CTCAAACTTGGTACTTTCCAGACCAGCATGTGTACAAGACCATAAACCTGCCAGTCGTTTGTCAGTTCGATCCACGTTTAAATTCAATACAACAGTACCATTGGGAAATTCAATTTTCTGACAAAATACTGTTTGGTCATGAGGTACACATCCAGATTGATACTGTGTTGATTGCGTGCAAGGATTGGTCGGTTGACATCCTTGGTAACAATTCCCATCTGTCGTACTTTCTCCTAAGGCAGGACATGTCAAGTAGACTTCTGCATCTGGTTTCGGATGATTTATACGACATGTAATTTGAGTAAAATTCTGACCATGATTCGCTTTCAACTCTGGTTTAAAATCTATTTGATA

>JAP02330.C|Supercontig_0000150:363869-364210

CCAAAACCAACCATAATAACATGGATAGATATGTTCGAAATTCCGGACTTTCGCTTCAATGGTCTCCAAACTGACAAAATGTACATTACGCATAGCTAAAGTTTCCGCATCAAATCGCCATGGTGTTTGTGGAATGACCATGATTACAAAGTAGTGAAAACGACGAGCCATTTCTGTATAATAACGTGCTTCCCAACTTCTCGTATTTGTGTTGTCAATAACTATTGGGCTGTATCCAGAACAAACAGCATTATACACATTATTATGGCACCATTCGTGAGCTTCACTCAATCGACTGATGTCAAATTGATACTCACACCCGTTCGATTCTAAATACCAGAA

>JAP02342.C|Supercontig_0000231:47495-47649

AGATGCTACTCAATGTGTTAATATAACTGTACCATCAGGTTATTTAGCACATCCAATTGGTTATTTAATTTTACAATATCATTGTTTATTTTGTTTAGAACGTAATGAATTAGCTACATTACGTGCTTTATTAAAATTAGCTGTAACACTTGCAC

>JAP02350.C|Supercontig_0000020:1371165-1371232

ATTACTGATGATGCAGATATCCTTGAAGCACTTCCGGGTTTGGAAAAAAGTCGATTAATCGGAGACCA

>JAP02351.C|Supercontig_0000114:155029-155349

ATGAATCGTTATTCAACAATCTACAGTTCAAGTTATCGTCCTTTTGATGAAAATCGACGAACAAATGCAATTAATAAAGCTACCATTCCCCCTCCGCCTCACTCAACCACAATTTTACCACATCAAAATATGAATGGCACAAGTCTATCATATGTAGGTCAGACGTGGGCTTCCGATCCCACTAATCATTCAACGATTACGACTGGCACAACAACTAATACAAATTACACCCACGCCTCCCCATATATTACTTGCATTAATAATCGTTTAATAAATAACAGTACAACAAATCAGACATCAAATAGTCTAAGTTTACGTCGT

>JAP02358.C|Supercontig_0002065:402486-402707

ATCTTTAATTCATCGTGTACATAGTGATGCTGTGTTAAATAGGAAAATAAGTAAATCTTGTAAATATTTGGCGAAGGAACATGATTATACTACTCGGACTATTACTAATCCACCTATATATCATCGTTCACGTTTTCATTCCAGTTCAAGTTCATCTGCTTCTTTGTCATGTTTAAATATTGATGATAATGAAGGTTTTGAGTACGATGATATTATTTCTAT

>JAP02367.C|Supercontig_0000033:95107-95365

TGACAAAATATCGTACAAAAGTTGCTGTTGATATATTGTCAAAAACATCGGTTTTCGTAAATCTTGTATCAGTTTATCGGGAATTGGGAAGAATGGCTATTCCTTTAAGTTATTCTACATTCAATCCACTCCCTTGTGAAAAATGGGTCATCAAGCTGATAGACTTTCAACAATTTTTGTCAAATTATATGACTGTCAGTATAAGCTTTACACGGAAATTGATTAAATCTGATATATATTTGATGGAAGTACGGCGTAT

>JAP02378.C|Supercontig_0000079:1461912-1462158

TGATAATACACTCGACACTTTATCAATAACACAATGTGATCCTGAAAAACTTGAACAATTTAATTATAAAGAAACTACCTCAGATATTACCTCTTCTCTTGCATTTCTGAGCCGACGTATAACACGAAACTATGCTAGTGAATTTATTTTACCAGATGATACACGTATTGCTGTCAGTCGTCTTGAAGGGATATATCCTATGGATAACAATGAGCAAGTTATATTAAGCTTGTCAGTTGGGCATAAA

>JAP02385.C|Supercontig_0000262:510813-511012

AGTTCCAAACCATTACCATTTTTACGTCGATCATTGTATCACTGTGTATGGGGAGCAAGAAATGGGCAGGTGTATTTACCAGAAATTGATTTTTTCCCTGGAGCAATATTATTTGTTCGCTATTCAGATAATCATAATCCACCCAATCCATTAAAACAATTTTTGCACCACACTGGAAATGTACTTCGTATTCTTATCAC

>JAP02386.C|Supercontig_0000174:670589-670824

AATGAAGCAAAATGTCAGTTTTCTGAATATTCCGTTCTTAAACCAAATCAACTTTGGTGGACACATGATGGTCAACGTTTACCACCTGTTTCGTTTAATCTTAATGATTACTTACGTAAATTTGTTGTAAAACAAGGTTTCGATTTAACTGCTGCCTCTGAAGGATGGAATCAAATGCTTGTATCATCAAATACTGATTTATTGTGGATACAGCAATTGTGTTTACACCCATATCA

>JAP02390.C|Supercontig_0001995:55281-55991

AAAAGCAGTATTCGATTGTACAATAGCTATATATTTCGTCATCAATGTGACGACATGTTCTCCCAAGATGTCACCAAGTTGAAAAGCTTCAGTAACTCCAATATCATTGAATAGAATCGAATTAAATTCATTAGTCATACGATCTTGCATTGATTCAGTTGCTTCAATAAGGCTTTTTACAACAGGTTCAATATCATGTTTAGTTTGTGGAATAAATTCACTGATATAAGCTCTTATTTGTGCTAAACCACGTAGTATACGTGGGAATACATGTGTTTTTTCAGGATTTGTTTGCATTAATTCAGTAGGAGTTAATAATCGTCCAATTGATTCTTCATGACTTGGTGTAGATACTAAATTTGTATGAAATTGACCACATACAGTAAAACCAAGTAATACTGCTGTACCTAATAAAATTGTTAAAATAGTTAATAATAAAAATTGTATAACAAGACATATCCATTTAGATTGTAAACGTTCACGTGTACGATAAAATGTTGATTTAGGTGAATGTTTTGGATGTATAGCAATTTCAATAGTTTCTCTTGATGTATGATTTTCTGTTGGTGCAAGATATAATAATGTTTCTGAACGTTTTTTTATACGTTTACGTTGACGACATGTTGAATATGATACAATTATTAATAAAATAGTCCATATTATAGAACATACAATACTTAATGCATAACCTGTACGTGAATATGTTGATAA

>JAP02393.C|Supercontig_0000119:266552-266664

TGGATTTATCTAGTTCTAGTGGTAGTTCAGATTGCATTTATGACAGTTTATTGTGGAAGTTTGTTGACGAAAACTATGAATTTCTGATAAATGATGACTTTCTGGAGCATTGC

>JAP02394.C|Supercontig_0000043:1542891-1543023

GCCAATTTAATACATTGATCAATATGATGCATAGCTTTTGTCATATCTTTACTTTTCACAGCCATCTGTACACATTTGTGATGTTTTCTAATAGTTGTTGAAAAATATTGTGCAGTTGTCAATTTGAATGAAG

>JAP02399.C|Supercontig_0000298:616753-616849

GATATCTACTAAGAACAGGATGACTGTACAGTATTAAGCCTGTGAAACAAGGAATTAAAGAAAGGCCAGAATTTACAGCCGTAAAAACTCTTGTTAC

>JAP02405.C|Supercontig_0000228:676981-677334

AGGTAGCACATATCATGCTATGCTAGTCAAACTGAATCAAATACTTACTAAGAATTTATATAATTCAGTTGAGCATTACATGACATTAAAGCAGCCTACATCAGTGCCAAACATAGAAACTCATGGAGAAGCTTTCTTATGGAAACAATGGGATCAGGGATATTCAGATCGATACTTTAAACTAACTCCAAGTGCATATCCGAGCAAATCTGATTTTATTACAATGGAAGCGAATGAAGCGCTAATTGCATTGTTTGAAAAACATCCAGAATTTCTAAATAAACTGTCTTCTCGTTATTTTAATCAAACATTTCAAAGCTCTCATATTCGAAGGAAACTATGGAGTATTATATT

>JAP02411.C|Supercontig_0000044:1596074-1596324

GCATTTGACTCGTTGTCAGGAGAAGAAGTAATGAGGGATGAAGTTAAAGCCAAATCATTAAAAAACATTCAAAGAGGACATTGGGATTCAAGATGGCGGTTAAATTCTAAGAACTCTGAAAACTCCATAAATCCAGATCCTCAAAGACGATCAGTTCGATCTGGTCATGAATTAAATTCTTTGGATGATAATGCATTTGTGATTATAATTGCAGTCTCTGTATTCGGATCATTTATTTTAATAGCGAATTT

>JAP02412.C|Supercontig_0000155:651740-652072

CCTTTCGTGATAAAATATTCCAGGTAAAAGAACTTTCAGATTTAACACGTTTCGTTGACTCTGGCAATCCTTTTTGACAAATACGACCAGGATTTTTCAATTTTTTAGCTCGTAACCACTCTGGACTGCCAAAATTATATTTTTTATGTCCAAGTTTTAAATCATATTTAGTAGAATTTGATGCAATATTTGATAAGGAACTTGTAAATATTGGACCAAATGATACGGTGGCTTTGTAGTGATATGTGTTTTTAGCTTGACCACGTAATTGAGAGGCACGATTTTCAAATAAATCAATTTTTTGATCAGGTGGTCGTCTGGAACGTTGATCTA

>JAP02429.C|Supercontig_0000193:307795-307912

TTTACCTAAATGTTCAGTGTGAAGAGTTGAAAAATAATCTGTCCAGTTGAAACCCAATGGAAGATCAGATAGAGTAGAACAATAGGAAATCTCTTTTTTGCATTCTTTCGTTGAACAT

>JAP02434.C|Supercontig_0001539:1084-1524

AAATTACTTGAAACATATTTAATTGCTGACAAATTCATTGAAAGGTGCGAAGCTTTTATTGACACGTATTTAAAGAAAAAAGCTCAGACAGCCTTCCACCACGAACATCATGCAATCCTGTCAAGTCCAACTCAACAACAATCCGTAGAAGTTAATACACCTAAACAAGCAGTTACATTGTCCAAAGGTGGATTTGGTTTTGGTGCACGTGAAATTAAGACCAAAAACGTTAAGAAAAAGTATAATCCAAGTAAACGTAAGACGAGTAGTTCATCAGCCGCTAATGATCCAGAAAATAACTTTTCTAGTGGCATTGGTGATTCCCAGTCACTAATTAAAGATCTTTTTGCTCGTTATGTTCTCATGGACGACGTTAGAAGTATTTTGTCTTCACAGTTACCAAGTGATGTGCCCAGTGAAGTGATTGATCAGGTCGCTGAT

>JAP02446.C|Supercontig_0000000:3169211-3169432

GAATTCAATAAATTCTCCAATGAAAATCATATTCAATTAATGTTTAATAAAATTCATGAACAAATGAAAATCATTAAAACATATCGTGATTTTAATGGAACATTATTTTGGATTAATCAAGTATATACACAAATACCTGGTGTTTCTAATCCAGTTATAGTTGGACGTGTACAACTTGGTCAATTATTAAATAATCAAATTTTATGGCTTGGACCAGATCAA

>JAP02450.C|Supercontig_0000093:885779-885857

TCATTCATTGCACATTTCATTGCTATTGGAAAAAACATATAATCATCTATTGTCCATGCATCGAAACGTTGAGGTGATC

>JAP02453.C|Supercontig_0000656:193289-193713

CATTTCAACTCTTTACAAAATTCAGCAATAAATGGTGGATTAGATGGTCATAGTATTGATAATGAATGTGCTGCTCTATTACATAGTCGTAATTCAGTAGCTAGTAGTCAAAGTGCACATATGAATATGATCAATAGTAGAATATTTGCTGAAGCTTATGCTGCAGCGGCGAGAGCAGCTGCATCTTTAGTCAGTGGAAGCACTGATATTTATTCACCAAGTTCACAAGTGACAAATGATCTAAGTTCACAAAATAATGAAGAACGTAATCGATCACAACATTCACTTCTTTATTCTATGAATCAAGAAATTAATAATGGATTTATAAATGGATCAAAAGAGGCTAATATAAATACAGAGAATACTGGTGGTTGGAAGAAATATAATAATAGCAACAGTAGTATATGTTTAAAGAATCGGGACAA

>JAP02458.C|Supercontig_0000043:1514116-1514192

TTGATTCAATCCATTGAACGTGATCAATTCGAAGTTGTAAAATTCTTGGTCGAACATGATGCTGATATTAATGAGAA

>JAP02461.C|Supercontig_0000462:481887-482128

CATACTCAAGCCGATTCGAACAGTAGGTGGCCAAGTGGACTTTACCGGTACAAGCATACCATGTTTGACGCGACTTCGTTTTCTTCGTAAACATGTTTCGGACCCACCGATAACTTCTGACCCAAACAAACGACTAAGTTCTCCAACATGATTTAACATCTTGATATTTACTTTTAGCAAGTCTATCACTTGTTTACTTTGATCAGCAATCCCTGGTTCATTTGAAAGTTTTATATTATCTA

>JAP02469.C|Supercontig_0000115:523044-523265

ACACCAACTGGTGGAGGAACAGGCTTTACTTTTGATGAAGTTTCTACAAATCCATCTTTATTCATTTTTTCATTACATTCTTCAACTGTATTCACAATTGATCCAGCTGATACAAATCCTGAGCTTAAAGCTAATGGACTTGGTTGAAGATATGGACTATTACATTGTTCTAAAGTTACATCTGGTTTACGACGACATGGACTTAATGTAACAGAATGACGA

>JAP02470.C|Supercontig_0000350:147313-147461

TCTGTAGCCGCAGCTAACAGGCATACAGTACAAGCCCATATTGCCAAATAAGAGGGTTGACGACTTAATAGTGTAGGAAATGAAAGAAGTACCCATTGTCGGACTAAATTTTGACCCTTTTCACTTTGCATAAAACTTTCAAATAACTG

>JAP02473.C|Supercontig_0000095:1284256-1284352

TATCATCCAGTTGGAGCCCATTGGTTCTATTCTACGACTTTTGAAAATATATCCGTTTGGTGGCCATTTTCACGTCATGATTCTTACCAAATTGAAA

>JAP02479.C|Supercontig_0001990:53595-53785

ATGGCTTATTCAGGTTATTCCACAGATAATTCTGATTCAGATCAAACTAATACATATCCACTTAAAGAAAAGTCAACATTGAAAAAACGTAGCAAACGAAATCATCATCGTAAACGATTTGGACGAATGGCTAAATCATGGAAATTTATGAAACTAAGAAAATTATGGGAAAGGGATATGGCTAAACAAAG

>JAP02483.C|Supercontig_0000311:576095-576481

AATGTGTTATTCGAATGGACCAAAAGAATCAAGAAGTTTATCAAATGATATAGCATTGCGAATAAAAGAGAAATTTGATCAACGTGTATCAAAGCATTTATCAAAATCTAAACAAAAAACAAAGTCAAATATATACACTTATACATCACAATATCACTTACTTGCAGTATTTCTGGTTAACAAAGGTAATGTAGAAGCATGGTATGCATATGCACCAATGCCAAATCACTACAGTAATCAACAAAATGAATTAGGGCATACTCGTTTATTAGAATTAACACAACGTCGATTACGTTTACTTCAACGTTATTATTCTGATTCCAGACAGTATGTTGTACTCTATGTAAATAATAATAATTCTGAACATTATTTACCGATGGGATTAGT

>JAP02489.C|Supercontig_0000157:85706-85893

TCATCATCATCTTGTAAACTATAATTCTGAAGAATAAAACCACCAGGTAGTCCAGTGAAAGATGGATTAACACTTGAAACTGGTGAATCAAATAATCGATTATTTCCAACACCAACACAACTACTAGTAACACCAAATGTTGGTAATATTCTAAATCCATGTGATGGTGATGAATGATACATTGTAGT

>JAP02498.C|Supercontig_0000313:121645-121739

ATGACAACAAGTTTTCGTCATCAGTCGGCACAGAATTCCTCATCATCAATACAACAACCACCATCTATGCCATTATTATACCCATTACCAGTATC

>JAP02509.C|Supercontig_0000095:932612-932783

ACATTTTCAAAAAAATGGAATCTTCACCGCCAGTATTATTGAATGCTTTACGGCCACTTAAAACAAACAGACCAAGACCACGGATGAGGTTAACATCTATACGCCAAACACCACAACGTGTTCTAGCTGCTAAACTTGACGTAAATATCAATCAAAACTTAAATCCTGTTAT

>JAP02510.C|Supercontig_0000009:2283504-2283736

TGAATTTGATTAGAATTCTCTTCATCACTAGCACCACGACCACTATCTGATCTTTGTTCTTCACCAATGATTGAATTATGAATTCGTGGATCTTTAACAATACATTCGCCAATAACCGGAATATTTTGATTTGTGTAACTTGGACGCATTAAACTAGCTGGACCAAAATCAAATATCGTTGATGTGTTATATAGTGGATTTGACGATAATGAATCAGTTCTATTTTGTTGATA

>JAP02511.C|Supercontig_0000305:615293-615416

AATAACAAGAATCGAGCTATGTCCGTTGAATCAGTTCGTAATTCACTGTTAAGACCTAATTCACTGATTGGCGATTATAGTGAATATTCGAAGAAAACATGTGATGAAGCAGTTGGTTCATGGA

>JAP02520.C|Supercontig_0000144:418816-419219

GAAAGTTATTACAGTCATCAAGGTAGAAGAGTTCGTTCAATGAACAGTTATATTTTTAATAAACCAAATATTACTAGAAATTTCGGAGGTATGGGAGTGCCTTCGGATTGGGTTATTGCATTCAGCACAACACTTGGTCGACCAGCAACACGTCCATGGAGTCCAGCATTGATTGATTGGATGACTATGACTGATGTTCGTATAAGCCTAATGAGTTTTCATCAGTCTAACGAACCAGAATATCGTATTAAAAGAAATGCCTATCGAAGTCAACTATCTGATCACTCTAAATTAACACAGAATTCACAAACTATAAACAAACTACCCTTATTTCAATCAAATTACTTTTATGATAATTTTAATAAGTATCAATTAAAACGAACACGAAATTTAAGAGATGGTCA

>JAP02522.C|Supercontig_0000459:255122-255208

TTGTACTTTATTCCATGAACTAGGTTCCATTTGATTTTCTTTTTGCCATGATGAAGGTATTCCAACAGGTCGTATAAAATCTGATGT

>JAP02535.C|Supercontig_0000011:211628-211860

AATTCAAATCTACAAACAAAAAGGTTGCCAAGTTTAGCAACGCTTACCGAAACTTTAGGTGGGGTTGTACAACGATTTCCAGGATTATCAACAGGATTTCAAAACATCCATTCCGCACCGAGTACGCCCCCTTTAGGTTGTTATTCTTTGAACTCTGCCAATCGTAGTCCATTTATTAATACTCCAACAAAAATTGAATCTCCTATAACACTAGAACAGTATCTTATTCATCC

>JAP02540.C|Supercontig_0000142:541090-541248

ATTTCATCAACTAAATCAGAAATCACAGATCAAACAGAAAGTAGCGTCGATTTTGAACCAATAAAACGAATTCAAACCCTTGTTGAAAATGTTCGAGATGAATCGATTAGCTTAGATGAAGTTCGACCAATACTACGTAATATATCACAAGAAATGGTT

>JAP02559.C|Supercontig_0000275:539992-540288

CATTAAATCTCTTACGTGGTCGAAAATCTTGGTGTGATCCACACAGAGGTTATCAAGCTCGTGGTCTTCATTTTCACCATCTGCGTCATCATCGTCGTGGTGGTGGCAGTACTACTGGCGTATCAATGACTACTCGGTCAGCTTCAGCAGCAGCAGCCGCTGCATCACAAGACTCAAGGGAGGGGAGTGTAGATTCGAAAGACCAGTCTGTTGTTAATGATCCTTATTCAGCTGAGTGTATTGACCAACCTTATGTACCTATAGAGACAAGTTATAGCAAGAAGTCTGGCAATATTG

>JAP02565.C|Supercontig_0000585:77954-78145

TCAGACAAGGTGATGGTGTGTACCTATCAATTGCCTCATGACTGTACGTCGGCATTTTGTCTCACTAGGCAAAATATTGTGAACTTCCGAAAATCTTGTTCAGTTTTCTCACTGAGAAAGCGGAGAGAATATACGAAAATGGCTGAGCATTTTGAGTATAATCATATACCTGCTCATTTATCTTCCATACAG

>JAP02572.C|Supercontig_0002057:185641-185782

TATCGTTAAGCGTATCATTTAATAACACAGTTTCGAATAACTTCCAAAATTCACTCACTGTTATTCCATCTATATGCTCCCAATCAATAAGTACACCATCAACTGGTGAAAGTAGATCAATAACGTCTGAATGATCATTACA

>JAP02573.C|Supercontig_0000043:1407559-1407725

AGATAAACAATATCATTCAATGATTCATCAGAATTTTGGTGTTTTGATTTAATCGTATCCAGTACATACTCTTTCTCTAGGCGATTAACTACAGGATTCAAAGAAACTGGACGTTGATGTGACATAATAACAACTAACAATTGTAAAGCTACACGAGCATGAGCACA

>JAP02577.C|Supercontig_0000679:134751-134870

GTTGTTTGTTGTACAGGTGTAAATGGTGACTGTACGGACCCTGTATCTGAAGATACATCAGAGCATGTCTGTTGAGAGGGTAAAATAAATGAAAAATTATTAAATAAATGACTATTATTA

>JAP02579.C|Supercontig_0000121:1115871-1116038

ATCAATAACAACACACTCACTTTCTTGATCATCAATTGTGTAGCTCATCAGTAGACGTAAACTTAAACTTTCATCATGTGGAATCATATCAGCCAGTGTCGCTAAAAGACGCATTTTCAACCCATTAGCTTCTAGATTCTCTTGATTGTTTATAAGTGTATAACCTTG

>JAP02598.C|Supercontig_0000016:902524-902744

TCATTATTTGGATGATCATAAGATGAGATAGGTATTATTTCACCATTTAATAATTGTAATCTTTTACATGCTGTAGCATGACCATATGCATCTAAACTTGATGTTGGTGGAGATGTTAACCATGCATATGTAATATTACATTGACCTAAACGTCTTAAACCAGCTTGACCTAATATTGTATAAGAATCAGCAGGTAATAAACGTCTTGAACGTAATGAATA

>JAP02605.C|Supercontig_0000156:1587717-1587876

AAGCTCAAAGAAGAAGTATATCTGTTACATGATGTCGTAGAAAGAGTCAACAAACGCGCAAATATGAAAGTAGCGTACTCTTGGAAGTTTCCTGGAAAATCTGCTTTTGAAACAAAAATTCTTCCTGTTTTATCTAAATGCAATTTCTCCACATCCAACA

>JAP02608.C|Supercontig_0000310:241931-242171

TCCGACTGATGATCTCTAAAATTCAGGTTTGGAATGTTGATATTAGTAATAGACACATCACAAGTACTTGGAAGGTGGAGAGAACTACTTTCCTCCTTTTTTTGCCGTAACTGTTGTAAAACACCATAAAACTGAGAAGATTCGCTACTAGTTAGTGTTCGCGTAGAATCAGACCGATCACGTGGAGTATATGACCCAAGTTCTTCTAGATATAATTTATTAGCGTTTGCAATAAATGATG

>JAP02620.C|Supercontig_0000123:596166-596379

CGTATGTCAAGTGATATCTCCCCGAGAATTCCTCTTGCTTTGTTATATTCACGTTTATTTCTACGTCGTTTATTGGCTTATTTAAATGTGAACACATTTATTGTACATATTTTACCTTATATAACCTTTAGTCTTATTGGAGGTAGTATAAACGCTTTATCTGATGAAAATATTGTATGGCATCAAAGAAGTTATGCTGGTCTTACAAATGAAC

>JAP02630.C|Supercontig_0000676:31797-32192

CTCCCATCATCTGATTTCTGCGAAGCGGATCCTTGTATTCGACTTCGTGAAGGAGTTGAAGCGTACGTACAAAAGTCTAATGATGAGAACTTACTGTTTGATCTTAATGGCACAGAAGTTCATGAAGCATTTTATTTGTGCTGCCTTAATTTTCTGTACGCTTGTAATGACCTACATAAAAGCAATGCAACCAACAGTAGCTCTGACATTCCTATTTTCAGTGTGTTTCAGGAAAAACAGTTGTCCTCATGCCTTCAGTTTGTTGTAGCATTTGGCATTTATCCGCTTTTAGAAACCGGAGTTTCTTTACCCTTAAGTATGCGTTTAGAAAATATCGACAAGTTCATCTGTCCTCGGAAGGAAAAGGATGAAGAAAGAATTGAAAAACTGATGAAG

>JAP02632.C|Supercontig_0000120:209856-209981

AGGTCTTCTGAGTTTGCGAAACGAGCAGGTCTTGATGGTGTCGCGTATATATTGGCTATGCGTTTTCGTGTTTTCCAGTTATTTGCACTCACTCCAATTTTCGTTATTTGCTTACCATTATTCTTT

>JAP02644.C|Supercontig_0000353:31478-31757

GTCTATTCCCAAAGTTTAATACCCATTTTGTGCGTTACTGCACGCTTGTTCAAACTATGTATGGCCGACGTCGAGCCGCAGCTTTACCCCATGTCTCCAGACGTCAGGGTGATGCTGTAATAACAGATACTGTTCAACACGTTGACTGTGCAGAAGCCACTATTGATACACTACATTCTCGTCTACTGAGTCGATCGAGGCCTGTGGGAGCGTCTTTCATTTCTTCATCATCATCCAGTAATTCGTCACGTTCACCACCTCCACTAACAAGACGGACACG

>JAP02652.C|Supercontig_0000153:404087-404518

ACAAACTCGTGATCACAGTGATTGGAGCATATTTTCTTCGTTAGCCAAACAGTCAACTCCAATAAAAGATGATAATATACACCAAAATTTATCTTCGACATCTGGACCCGCCGCTGAGAGTAATGACATTCCAGTGAATTCTACTGCTGAAACTTCGTATGACACCGAAGATGATTTGCCATCGTTAGAGAAGCATGATAATGATGAAGTAAGCATTCCGTATGAATCATCTATTTTACTCCATGAAAGTGATAGTCTTAATAATCGTGCTGAGAGCCTGAATGGGTATAATGATGATGATTTACCTCCTCCAGTAGTCTCAGATATTGATAATCAACTCAGCTATTCAGATATGCCTGTAGTTGTCGGGACTGTCCCGGATATTATAGAAGAAGTTATTTATGATGGTTTGGAAGTGGCTTTTCAAGATGA

>JAP02659.C|Supercontig_0000284:362451-362634

CTGATTTAACCAGTTTTGTCTTGGAGTTGTAGGTTGTTTTAAATAGATTTGAGATAATTCTCTATTCATTCCAGTAGCTTCTATATCATGCCTTGGGGACCACATAATAGGTTCAGTAAATGATGAACAATATACAGGAGGATTAGGTGCAGGCCCATGTGGATCAATCGATGGTGGTTGATAT

>JAP02666.C|Supercontig_0000238:472976-473154

TCATCAAGCTTTGTTAGGGAGTTTTCTTTACTATTTGATGGATGGATATGATTCTCCAGAACCAACTTTGGAAGTCTTGCATCTTCATTTTTTAAAGGTGAATCAGTGTTGTTTGCGCGAATTATTGAATTTTCGCCAACTGGTCGTTTAGGTGAACCAAGAACAATAGAAATTTTTTC

>JAP02669.C|Supercontig_0000073:285405-285491

TGAGTCCAGTGGATTTGAAGAGTATGAGAAAGAAGAGTTAACTCGTCAAAATGATTTGAAAGAACGTGATGCCTTCGTTAAACGCCT

>JAP02682.C|Supercontig_0000230:196635-197117

TTCCAGTCACGTGACTCAGCAGAAATTAAAAATATATATGGCAGATCTTCTAAGCCAACATAAATTATTTTATGATCTGCAGCCAGACATGTGATTTTTGGAGATTCATTAGGCGAAAATTTATCTGGTAGACTAGCATAGCGAAGAACACCTTTGATTAATTGATCACCTGGATAAATTAACTTTATATCATATAATTCATCATCTTTCGTAGAAGGAAATCCTAATTTACTTTCGTTAGAAGATAATAATCCTGTATGATCTGTTGTCTCGTCAATACCGACTAGTCGGTTTTCTGTAAGATTATTTCCAGGTATATACATACTGACAATATGATTCAATCGATTACGTAGAGATTTACCGAATGGTCGTCGTCTGACTGGATGAATAATTCCTTGTTCAACATTACCATTTGAATTATCATCACCAGTCATATTGTCTACAGCTGCAAGACTTTCATAAGCTGGAGGTTGTAATTGCATT

>JAP02700.C|Supercontig_0000182:480881-481201

CAATCTAAACAGCATGGTAAAGGGTATGATTTACGTATTGTCTTAAAACCATCTACAATAAGCGCTGATGATTTATCTCCAAAGTCAAATAGCGCTTCAAGATTACGAATTAGTCATTTAAAACCAGGAGTATTAAATTGGATTATTGAAACAGTTCGACTAAGTCTGAAAATGCCTTTGAGTCCTGTTCATTTGACAATTTCATTTATTTATTTCAAACGTCTTGGACGAGATGATTTAGGCAATCTAGCAACAAAGTCACGTGTAACACACTTAAGTTTATGGAACCCTTTCTATTGGGATGAGGATAGTTTTATACCT

>JAP02702.C|Supercontig_0000350:157286-157611

TGTATAAAGTTCATTTAACAATGATGAACCAGTTGGTGGACTATGCTGTTGTAATTGTTGTTGATATGGTTGTCTTCTAATTAATAATGCTGTATGTAATAAATACATTGCACTATGTAAACCAGCATTTTGTACTGTTAATAAACTAGAATTAATAGCATTCATTGTTTGTTTTACAGCTGGTTCAAGTGAATAAGGTTGAAGTACATGTGAATTAGTTGGTGAACTATTAACTAATTCTAATACTGCTGTACATTTTGAAATCCCTAATGCTAACCATATATGTATTGGTGATTGGAATTCATCCCATGTCGGTAATTGTTTTA

>JAP02703.C|Supercontig_0000361:1678-1916

GAGTCGGATGAGTCTGTTGAGGCATGGTGGGAGAGTTTTTTAAAGCAAAAGCTTGCTGATGATGAGTTAGATGTAGAACTTCGGTCTGCATGGGAGAACTGTGTTCAGAGACGAGCTAAATTTGCTCAAGAAGTCAAACGAATAATGGAGTTATATCAACCAATAGAGAAGTTACTTAACTCTACACTTAAATCCGATCGTGAACAACGAGGTCAAAGTCTCTATAGAGAATGGGGTAA

>JAP02713.C|Supercontig_0000139:143499-143983

TACCCGAATGCCGTATACGCGAGTATAAAAAATATATGTTTTATATGTTTTTTCTGGTGCAAATACCCCGAGTGGTGTCCATTAACCACGTTAGAATCTTTCGAATTAGTTTGTTTAGGATCACAACAAGAGCCCGTAAAATCAACTTGATTGCGAAGAGCACGTAAATTTGTTTCGAAAACCCCCAATTCTCTAGCACTTGATTGAGCTAGTCGTATCAATGTCACAAGGGTAGCGTCTATGATACCTTCCAACTTAGAGCGACCGGTACTTTTCACAATTAAGTTGTTGATCCCCATCAACGAATGGTGAACAAGATGACTTACAATTTCCTGTTTTTGTAAATGGTCACTCATGCAGCTAGTGAATGTTTTGATGTATAGAGAGAGGGCAATATACCTGGTTACAAATTTGAAACGACTGGGAACAAGATCATTTGATGTGATAAGCTGATTCAAAATAATTGTAAATGGTGAAAAAAGACG

>JAP02718.C|Supercontig_0000074:389027-389309

TTCAACAAGAGGTTATTTTTCACGTATACGTAATTCACGAAATGACTGTAAAAATGACGGTATACAGTTAAAACATATTAGGTCGTATACACCGGATAATAATATGAATACATTTGAAAAATATTCACTTTCTCAAGATTCATCGATTCCACCAACAGCATCGCAATCATCAATATCATCACTATCATCTTCATCTTCTACTCGACAATTATCACCGGTCACAACTCCAAAAGCTCCTTTATTAGGTACAAATTATAAACGAAATTTAAATAAAAATAAAATA

>JAP02720.C|Supercontig_0002061:372267-372563

CTCCATTAGCCATATCTTTCGACCATCGAGGAACCACATCTTTTTCATAAAATCTTAGTAACGTTTCACGAAATGCATCACGATCAAAAATGATTTGTGGTGAAGATATTTCCTCTGATATAGTTATATTGTTATTATTTTGTACAGGCCAAATTAACTTCGAAAGAAAACATGTAAACCTTAAAGTAGAGTATGGTTTTCCATCACCACAAACTTTCAATAAATAGTTGGATTCAACTGTTTGTAAATCGTCAATAATTCCTTGACAAAGGCTTTCAACCTCATTAAAGAATTCTA

>JAP02729.C|Supercontig_0012473:662-817

TGAAGATGATAAAGTAGTGGTGGTGAAGGATCGTACAATACTTTGTGTTTTTATTTCACTTTGTTCAGTGAGTGTGAAATTTAATTGGTAGGCTTGTAATTCAAGTGATTCCAACTGTAACTGATTATTAGGATCCAAGAAATTAATATAATACTG

>JAP02732.C|Supercontig_0000079:282063-282379

CACAGAAGCTAAACTAATAGAACCTTCTTTAATGAACAGTATTAATGATCGAGCAAGTTGTAAACCTAAACGAATTAATTTACGTTGATTTGATTGAACTAAACATTGACTAATTAAATTTACTTCGGATAATAATGTAGATACTAATTGAGAGATTAAACATGATTCAGAATTAATATTTAAAATAAGCGCTTGTCTAAAACGCCAATGTTTCATGAGTAAACAGATTGCACGTGCAGACGATTGTTGTAGAGCTAATTCTATTGAATTTGTTGCAGGACGATTATGCGGTGGTAGACATGGCCATAAACGTGATA

>JAP02733.C|Supercontig_0000292:846303-846445

GAAATGGATATTCTAGAAAATTTCTCATAGTTACATTTGGTGGATATTTGTAATGATCACTTTTGATTGACTTGGCTACAGAATGGCTTGACATTGATACATTCCACTGGTGAATAAACTCAGCACGTGCTCTCATTAAATCA

>JAP02747.C|Supercontig_0000454:125426-125687

ACCTTTACTACATCTGCACATGTGAATTTATCAGGTCTGTAATTGCATTTCGAAGATCCTACAGTGGTCTGTGGTGGAAGCTGGCGCCAAAGACCACATTTATTACATTTTAACCACCAGGGTAACATTTGTGCAGCCAAAAAGTTAGCCGCGTTCAAAATACGACGGCGACCCAACTTGCTTGACTTTTTCAGCCACTCCCGACGCCATGAATTCATTCTATGACGCCAGACAGAATTCACCGACTGGGATCTGTGTTTTG

>JAP02757.C|Supercontig_0000177:784274-784656

AGAATAACTCATGTTGCCTATTTTGATAATCAGTTGGATAATATTTTACTTCAGTAACATTTGTATTAATGCAAACAATTTAAATCCTCATTCTGATTTAAATCAAAAACATTATACTGATGAAAAAGATTCAAATATTAAAGTAATTTATGAAGCAACAAGATCTGTATGTTATACTAAAGGTCAAGCAATTGAATGTTCTCCACCATTTACAAATATTGCTGAAAGTGTTGATATTAAAGCAACTTCAACATGTGGTCAATTTGATCAGTCAGAAGAAATATGTAGATATGTAATGGGACATGAAAAGTGTGAAACATGTGGTAAAGGTGAGAAATTTGGAACACACTTACTAACTGATAGACATCAAATGAATAATGAAA

>JAP02762.C|Supercontig_0000111:608296-608479

CCATAATAATCTTTAGAATAAAATGTAAAATCCGTATCACATTCTCTTGTATACATTTCATTTTCACTAATCAATTCAGTTGATGGCATAAATTCATTGAGATTAATTGTATCCATAGTTTCATCTGATGGGCTGTAACGTGTTAGAAATTGTCTACGACGTGAATCAGATTGTGTTACATTGA

>JAP02763.C|Supercontig_0000058:1561104-1561539

GCTGTAGTATCTATTTCTCCGGGATTTCCACAAGCAGACATTAAAGTTGAAGCTAAAGATGCAAGGAGTTGTTGGGAATCCCAAGGATTAGTTGTTTGGGTTGCCTGTCTTTGTTGCATCTGTTTAGCTGACAACAAGTAGTTGGCGTACAGAGAAGCCAGAGTGGCTTGTTGTGAAGCGAAAGGGTTAGATGTCAAATTAACATTAGATGATCCGATATCTGGTGGAATGACTGATCCAGAACATGCTAACATAAGTTGACTATATAGAGCTGCCGCAGTTACAGGATTGCACATGTAAGCCAGATTCATTGCAGCAGCCTGCTGATATACAGCATTCAACATTGAATCCATCCGTTGGTTACCGGTGTTTAATTGGAATGGATCTTGTACTGTTGGAGTACTACCAGAACTCCTATCGGGAACAAATGGATATG

>JAP02784.C|Supercontig_0000050:171880-172062

TGACATTCTTTTCTGCTCCGGCCAAATACGCTCATCAAGCGACATAACTCTTGCAAAAAGCTTTGAATCTGAGAATATTAGTATTGGTCAAGTTCTACTAGATGCATTTTCTAAGGAAAATAACGTAAATGCTGTTGTTAATTTAGTCAACTCTTTGATTGGTTCTTTTGTACTTATATTTGT

>JAP02791.C|Supercontig_0000228:370319-370653

GACGTTTTCTACGTTCTGAAGTTTATTTAACAATGTTAAAAGAAGCTAGAGAAACAGCTAAAGCGGCAGCTACAGCTGCATTAGCAGCATCACTTCAAGAAGCTAGTGATGAATTAGATGAACCAGTATTTCAATTAGTACATTCAACTACATATACTTCACCATTTTCATTTTTAAGTACATTTAGTAGTACATTTAGTGCACAGAATACACCAAGTTTAACATTACCTGGAGTAGCAGCCGCTGCAGCTAAAGCTGATGCTGGTTTAAATAATATTCAAGATTTACAATTATTAGGTTTATCTACATCAAGGCATATGCGTGAAAATAAACAA

>JAP02810.C|Supercontig_0000130:877842-877995

CATGTTTTTCAGAACACGAACTGTCTGACATTCCTTTGAGTTCTAATGACGAAGACAGAATAAATTTTGATGTATCTGATATTTCGAAACAATTGGTTCTGTTGTCCCCTTTTTCCGGGAAAAAGTTCGCAACTATCCGTTCAGAAAGCAATTA

>JAP02816.C|Supercontig_0000329:1338-1511

AGATAAACTTAAAGATCATTTGAAACATCATGATCGTGCAGCTAGGAATTTTGAGTGTCAACAGTGTCAGCAACCTTTTGTTCAAAAATCTGATCTTAACAGACATATACGTGGAGTACATCAAGGTGAGCCAGGAGTTGGGATAAATATGACAATAAAACGGAAAGCGCCAGG

>JAP02817.C|Supercontig_0000456:107331-107476

ACCGACAATTACTGCTTTCATCCATTTCTTTGATAAAATTAAAAAATATGATTCAAGTATCATTTTACCACGTAAAATACATAAATGCCCAAAACCAGCTAAACGTATTTTCAACTTAATATCACATTCTTCGTCCATGTTTCCCA

>JAP02821.C|Supercontig_0000149:459828-459999

ATAGGTGCTTTGATTAAGAAAGATGATGTAGCTCAAGAAATATGTTTCAAGAAAAGTATACAAGCAGCAAACAAACTATTACAGCAACACGATTCAACGAATCACCGGTTTCAATTAATACCAATAATAGAAACAATTGATGGAGATGACAGTTTCGAAGCAACGCGAAAAG

>JAP02825.C|Supercontig_0000263:76813-76959

TTTTAAAGCTTCTGTAATATGTTGATTGAAAATATCATCAACAAATTCATGAATAACACGATATTTATCAGGAAACATTTCTGCTTTGAATATTTGTCGATCCAATTGTAATCCTTGTGGTACAAAACGTGGTTCCGGTTTATGATA

>JAP02828.C|Supercontig_0000084:182079-182240

CTAATGTACAACCGGAATATGCAATATTTATGTTAAAATATTTTATGTCACTTGTTGTTGGTATTACATCAGGTTTTTGGATATGGTCTAGTAAAACTGTGAATTCATGGCAATTATGTTTAAAACGTGTATTCAGTCGTTCATATCAATCAAATTCTAAAC

>JAP02831.C|Supercontig_0000567:32074-32255

GGAATCTTCGCTTTATCCAGCTTTCTCAGTGAATCAAAGTGTCTAAAGTATTTGGATATACGCAACAATAATTTAGATATAGCCTCTATAATGGCCTTATCAAAAACTTTATTCATTAATAGAACATTAACAAGTCTGGTTTCAGACGCACATCGTTGGGCGAACTCTCAGCCGGATCTATC

>JAP02870.C|Supercontig_0000215:546725-547060

AGATGGTGATTTCACAAAGCAACCGGGGAATGTACAGTGTACAAGTGAGCATATATTTAATTTAATATGTTCTCCCCACTCCAACAATGTAGAAAATGTATTCATGCATTGACTGCATTGCAATTTGGAGGTCTCAGATGAACTACTATGTTGAGTCTCCCAGTGAATCTGCATATGTTGAAAACGATCAGTACTAAATTCATGAGAACAATGAGGTTTAGATGGACAAGAAAAGATTCTCAGCGATTTTGAGGAGGAAGGAGTTAGTAAGTGCTGGGAAATGTGAATCATCAAATAATTCCAATGAACTATGGAGTCACAGTAAAGGCATAAATG

>JAP02877.C|Supercontig_0000235:476234-476416

GAAAGTAAACCACAGACGACCAACTTCGTCAACAGAAGACTCATTAATTGTTAAACCAGATTCCTCATTCAGCTCTCGAAGTGCAGCAGCTTTTGGGTTCGCATCACTTAATTCAACTTTACCACCAAATCCGTTCCATCGTCCTTTACCGAAACCACTCTGCTTTAAACCCAAAAGTAACCA

>JAP02895.C|Supercontig_0000118:551583-551783

CTGAGTTTTATCAATTGAAATTATTTCAGTAAATAAATGTAATTGTGATTTTACAATCCATTCTCCATGTAAATTAGATTGAACACCACGTGAGTCATCTCGTGAAGATCGACGATCAATCCATACATTGATTTGACCAACTTTAGGACTAACTACACCAGTAGGATGTGAAGTGAATAGATTTAATCGTTGATATTGTTT

>JAP02897.C|Supercontig_0000196:538512-538683

TATCAAAACTTAAATGGGCTAATACTTTATTAATATGTTCGTCTGGTATATCATCGATTTTTGCAAGATAACGATAGAATTCTAACCATTGTTCAAATGGTAATCTAGCTGCCCCACCTTCAGGATCTGCCGTTAAAAGTTCACATATCAATTTCAAGGTTTCTGATAATGT

>JAP02911.C|Supercontig_0000063:1876431-1876577

GGTGGGTTATGTTTACTAATAACAGAAGATACATTTGATGGATTGTCAGTGGAATCACGAATATGTCCATTCGAAGAAGAATCCAGTTCTTTAGCACCTACTGGCCAAGCATCTTCGCCAACTTTCCGCCAACCGCGTAAAAGGCTA

>JAP02928.C|Supercontig_0000118:361160-361411

TAATTTGAGAGATTCTACAAACTTACAAAACACAGTACCAACCACAGTATACACAATGCATGATGACAAAGGGCGGTTAATACTACGTATAGATAATGTTGATGTACAAGATATGGGGCACTATAAATGTCATGGCCACACCACAAGTTTGGATGCATATCTAGTGGTGGTCAATAAGAACTATCCTGAAGAACCGCAAAATAAAACGTTGCCAAATGCCGGAAGTATGCTACCACACTACATGATGTTTTC

>JAP02929.C|Supercontig_0000058:1688547-1688761

TAATCTATGCTACTTTAATATTTATTTTAGTAGTCATTATACCATTAATGTTAAAAATGACGTTATATGGTGAACCAAGCTTTTATTCACTTTTCCCAATCTATTCAATACTTTTCTCATGTACAACAATATTTGAAGTAATCAAACGTCTTCGTACATTACAACATGAAATGAATGAAGACAATGCACTAGTGGAAGTATATTCTGCTTAAAAA

>JAP02935.C|Supercontig_0000096:786677-786913

TGTTCATATCGTACGATGCAGTTTTTATACATATAGACAGAAAAGTACATGTATAACCATCTGAACCAAACTAACTGTGATTTATGACTTATCATACTGAGATAAGGAACGTACATTAATGGGAAAGGCGAACAAATGAATTCCTCTTTAAGTAAAATAGAAACGAATAGATCAATTAAGAAACAATATTTACGTAAAATGGAAACACTTTGTAGGACAAATAGTTGGGGAATAAGT

>JAP02954.C|Supercontig_0018514:984-1212

ACAAGATAACCTCTCCAATATGATTGTAACTTAATTGCTGCTTCTTCATCTGTCCATTGTAAACTTAATGGTAATGGTTTACGTGGATGTTTCTTCCATTCTTCCATAATCCATGGTATATTCAATAAGCTTTGTACATTTTTACGATTATCTTCATTAGTATAGATATTATGTTTATATAAATAGAATGTTAAAAAATCTAATCCATTAAATCCAAAACGTTTTTTCT

>JAP02958.C|Supercontig_0000284:355931-356079

GATCTTCGAGAAGCTTTCATTCTATTTGATGTAAATCGTGATGGTCGAATAACTGAAACTGAATTAGAATCTGTTCTTGGATTTCTTGGTGTAAAAACAACACGAGATGAAGTACGTCGAATGATTCAAGATGCTGATTGTGATGGTAA

>JAP02988.C|Supercontig_0000156:1382739-1382959

ACCAGCAGCTGTCATTTGAGCAAGAATGAGAAGAAAGACAGATATTGCATCAATCTGCAAATGACCCCATTCAAGATCACCAACAACAGTTTTATATGAAGTACTGTCAAACTTTGCATGCAATGAATGAATAGGATTTTGAGTCTTCTTCAATAGTTCTACTTTATCAGACTGTTTCATATAGCAGATGAGTATGCCACGCATCAGCTTCACTACACACT

>JAP02997.C|Supercontig_0000480:628275-628527

AGTTGATGAAGATTTGGGACTACCTGCTCTTCGCGTATGGTCAGACATAAACTCTATACAAACGGCTTCCTTAGAGAGACGCATTTATTTGTCGCAAATATTGATTTCATGGATTATTGATTTTCGAAACCCGGTGCGTGCTAATTTATCCATCAACTTATCCTATGACCATTTATTACCTGATGTTCTGAACGGAATCAGTAACCACCTTGGAAGTCCACGAATTGAAATTCGTACTCTTGGTATGGTTATA

>JAP02999.C|Supercontig_0000107:801657-801752

AGCACAGCTTAGACCAGCTAACTCCATCAGCTGATCAACAGAGAAAGCATATTCTGTAAATAATTCATTATCTACTTGTTGAGCTTCTACCTGACT

>JAP03003.C|Supercontig_0000223:41263-41401

CTGTTGTTTTTGATGAGGAGGATGATGATGATGGTGATGTTGTGCATTTGATGCAATCATTCCAAATAATTTATACCAACCAAAAACAACTGTTTTTAAATCTAAATCATCAAGAACAATTTCACACATTCCTAAAAAT

>JAP03023.C|Supercontig_0001960:53500-53616

GTGTGAAGTGTAACGGGAAAACTGTAAAACAAGCCTACCATACGATCTGTACAGATTGTTCATTTGCTTTGAAAATCTGCTCTAAGTGTGGAAAATCAGATGGACCGATTACCTTTG

>JAP03031.C|Supercontig_0000280:215666-215823

TTTGAATCTTACACGAATTCAATAACTAAACGTATAGATACATTGACAAATTTAATCAAAATGGCACATCAGTTAGAAGATCTACCAGCAATTATTTATAATACAGTACGTAGTCGTCTAAGAACACATTTAGGCAATTCACCAATACCTAGAACAGC

>JAP03040.C|Supercontig_0000205:224799-225100

GAAGAAATAGAAGAAGCTGGTGAAATGGTATTGGCCAAGAAACAAGAATGCATAGTTTACGACAGGAATCGGCAAAAATCACGTGAAGCTGTGCGACAGCTAATGCAATTGGACTCAGAAAATAAACAATGGGCGTGCTTATCTGACCAGTTCTTTCTGTTTCCGTCTGTTGGATTGAGGAAAGCAATTGAGGAGGATATTAAAGTTTATGATTCTGAAATCCAGAAGTTAAATCACCAATTAAAGGAAGATATTAAATGGTTACATGAACTTGAAGGTAAAGAACCCCTCAAAGGATTTGA

>JAP03055.C|Supercontig_0000143:696831-697384

ATCTTATTAATATAGATGATATCAAAGTTACATTTAAAGAACAAATTATATTAATTTATGGAGGGTTACGTGGGGCTGTTGCATTTTCATTAGCTATATTAATACCATCTGATATTTTAGGTAGTAATGGTGAAGAAAATCAAAGTCTCATAGTTACTACAACATTATTTATTATTATATTTACTGTTGGATTTATGGGTATAACAATGAAACCATTAGTGAAATTATTAAAAATACGTATGGAAAATAAAAAAATTTTAAGTTTATTTCAATCATTAAATAATAATATATTAGATGAATTATTAGCTGCTATGGAGATTATCATCAATTGTAAACGACGTAATGTATTAAGAGATTTTTTTAAAAATATTGATGAAAAATATATAAGAAAATTATTACAAAATAAACCAGAATATTATAATGAAAAATTATTTAAAATTTATGAAAAAATTTCATTACGTTTACATTATGCTGCTATACGTCCTAATCAATCAAAATGTTTATTAACTGATTTACCTGATACAATTACATATAAATATCTTACTAATCAATTA

>JAP03062.C|Supercontig_0000101:283791-284011

ATGATTTTCATTTAACTACAACCCCCAAATCGACGACAACTGTACACAATTTTGTTCACATTGGACCCATTTTAAACGAGCAATTATACACTGACCCAAATTTGGAGCAAGTAGATGCAAGCCAAATCTGGGATAAATTTCCTGAAGACAGCTTAAAAGAACTTATGGAACATGGACCCATGAATACGTTCTTTTTGGTTAAATTCTGGGTATGTTTTGAT

>JAP03069.C|Supercontig_0000077:1103280-1103483

TCTTCTTATTAATGAACGGTTCGAAAACCTACCTCTTTCAATTTGTGCTGAAGCTGTTTCTGCCTTACCATCTGAATTTAAATCCTCTAATTTACTTCCTACTCATCTCTTCATATTATCATGGGCATATTCAGAAGAAGCCAAAGACAATGATAAGAAATATGAATTCGCATATCCTGAAATTGAATTTATTGTTCCCCATGC

>JAP03071.C|Supercontig_0000303:878815-879014

TTACCACGGTACTACAGTCGAAGTCCGTATGGCAATGAAACTAAAACACTTGTTTTCCTCCGATCAGATGTAGAGCAAATGTAACTCATTTAAATATCTAATGTTACACTCCAAGGGCTTACTCAAAGCACGGAAGCTATGATGATTTTGAGTTGCGACGGAAACTCATGAAGGATATGGAGCGAAGAACCAAAACAGGT

>JAP03072.C|Supercontig_0000191:392889-393086

GACTCAATAGCAGCACGAGTAACATCTACACTTGGACCAAGAGAACAATACCCGTTAGCATCTGGAGGACTGATATTCAGTAATGCTAGGTCAAGTTTTATTATATTTCTGCGAAAAAGGTGTGGTATTTCACTCAAAAATATAGGAACATAATCGGCTCTTCCACTTTTGATCGGATCTCTACAATTGCTGCCTGTA

>JAP03078.C|Supercontig_0000143:65980-66115

AGTGGAATACAGTGATCAAGCATGCGTTGCTCTTCAGGTCCTGGGTAATATAGCATTAACTCCAGATGGATGCAAGTTGCTACGTAATAAATGTGGCTATCTCTGTAATATGTTAAAAATGCGCGATCCATACGTC

>JAP03083.C|Supercontig_0014919:743-965

TTCCATCTCTCCAAAACCCAATAAATCGGTGATGAGGATGCACTAATTCTCCATAACCATTCATCTTCCCAGCTTTCCAGTTTCCAATATATTGTAGCTTAGTTGAGGCGAATGTGTATGTACCTCTACCATGTCTTAAATGATCTCTCCATTCCCCTTCATATGTATCTCCATTAATGTAGGTATACTTCCCTTGACCATATCGTAAACCCTCGCTCCAGTT

>JAP03084.C|Supercontig_0000072:498500-499029

ATTCATTAACTCATTATCCATTAATACGTGATACAATTGTATGGTTAACTATGGAACTTGATTATCCTGAAATTTCTGAACATGAATTTATATCTATTTTACAACCATTTGGTTTAAGATTAACAGAAGATTATCGTCCATCTATACAATTAGCTGGTCTTAATGTATTATATAATTTAGCAAATAAAGCTAAAATTGCTGATTGGCGTCAAAGTAATCGAGCTGAAGCAGTTATTTCACAATTATTAAATCATAGAATTGCTTGTAGTTCAAACTCATCAGAAATATTACTTCATAAACTTTATTCAACTTTACTTGTTTTAACTAATTTATTGAGTAATACAAATTCAGCTAATTGGTATGAAAAAATTGCTGAAAGACTCTTATTTGATCTTTTAATGGAAACACGATATAAACGTCAATTAGTGCTACTTAAACATTTGTTAAAATTAATTGATATTTTAAAAGCATCTTTCTCATTATTTACCCGACAATTTATAAAAGTTACTTCAAGTATTTTACTTGGTCCA

>JAP03087.C|Supercontig_0000235:20934-21120

TGATGCTTTTCGTAAAATTCAAGATTTAAATAAAATTTATGGTGATATTGTATCATTTCAAGTTTTGGGAAAAACAATAATCATATTGTATAACTATGATTTAATACACGAAGCAGCAAATGGGAATCGTTCGAAAGTTGGACGATATACAATGACTGTAAATGATTTATTAGCAGAAAATTCAGGT

>JAP03098.C|Supercontig_0000054:791666-792043

ATATATATATATATATATATATATATATATATATATATATCACCTTGCGAATAGGTTGATCCTGTCTGCAGTCGATTAGCATTAATGAGTTATCTTTGGTCAAAGCACATGCAATATTATTCTCGACTGGATTGAACTTTGCGACATGAACCGGTTCTTGACCCCAACTCCATTCACGCAACGGTACATCAATTTTAACTGAAGAGTATAGCAGACACGATTCTGGACCACCAATAAGAAACTCATTTGATGAAGGATGATGATCCATACAATGAGGAGTCCATTTCATTAAAACACTACTCAGTGGTTGTTTCCAACATGATACAATATCATCTAGTTCATTCGCCATTGTTTGACTTGACCATCTAGACTACACTA

>JAP03102.C|Supercontig_0000146:516409-516593

AACTTACTATAAATAAAATCCCCAGTGTAATATAAGAAATTACACATTAAAACATAATAGACTATAATTGCACCAAAAAGAGCTAACATAGAAAATGAGATTGCCAAAATATAACCAGGTCTTCCTAAATGATATTGACATACGTCTGTAAACTCTAAGTCAGCAGATGAACGAAGATTTTGTAT

>JAP03111.C|Supercontig_0000076:985178-985375

ATTGAGATACAATGGGCTGAACATTGTTTCCGTCATGCAGAAGCTTATTTTCGCTTACTTTGCTCAGTCTCTGACCCCTCAAAATTAAAACTAACTGCCCTAGATGATGTTATATTCGACAAATTTATGGGAGAGTTTAGTGATGTACAATTAGACGTTATTTCTGAAGATTCTTTGAAATCAGAGCAATCAAAAGCA

>JAP03114.C|Supercontig_0000156:1226513-1226678

TCTCTGATACCTTGACGCATTACTTCTCGTTCTTCTTCCATTTTACGATGTTTAGCTTCTCGTTTTTCTTCTGCCTCACGTAATGCAGCTTCAACTTCTGGATCAATTTCTTTACGTCCACCTAAAACACCTTCACCTTCATCTTTCTTATCACCTAATGCACCTT

>JAP03121.C|Supercontig_0000041:1184764-1185078

TCACCTAACGGTGGGACTTTAGCTGTTGTTGGGAGCGCAGCCATGTTCTCTGATCCATATATAAAAAAAGAAGATAATTTTAAAATATTTGAGTTTTTATTCAGATATCTTACAGAGGAATCCGTCACGCTAAACTCCATAGATTCTGGAGAACCGGAAGTTGAAACATATTACCAAGTCCCTGATATTATAAGCCTATCTAGTAACTTAATGTCTTGTTTACAAGAAAGTGATGAACTCCCACAAAACGTCAAAAATTACTTTGATCAGGGTTTGTTTTGTATGGACACAAGACTCGTTCCGAAAGCTATTCAG

>JAP03123.C|Supercontig_0000155:907131-907204

CAAATATCCAGCCAGCATCATCAGTTGTTGGAATCTTCCCGTCGTATCGTCCACAATATGGATTCCCAGGTTAT

>JAP03130.C|Supercontig_0000076:928062-928584

GATTACAAACAATTGCATAATCATAAAATGCACGTACAATAGCAAGAAATCCAGATAGAATCCATAAAAATCCATAAATAAATTTGATTGAAGAATGTGAAAGTATACGACGTTTACGCATTGGATACACTACTACCTCTGTACGAGCTGAAGTAATTAATACATTACACCAATTACGTGTACATAAAGAACAATCAGAACCCCAATTAATAAGAGCAAATAATATAAATTCTGTAGGACTTATCATACGTGGATTTGATGAACATATAAGTTGTGTTGTATATTCTATACAAACAAAAAAATGAAATAGTACTTCAGTAGCTGATAAAACAAGTAAACTTGTTCGCATAACTGATTTTCTATGTTGTCTTGAAAATATTGAAATACATAATCCATTTAATATTAAAGCAATTATGCTAAGTATTAATATTGGTAAAGAATATTCCAATTTTATAATACCAGTAGTACTTATATTTCCATTACATGTACTATTGTATTCTATCCATAAAGTTCCATCAAGCAT

>JAP03131.C|Supercontig_0000007:2444210-2444353

TGAGTTTCTATCATAGTTCTAACTGTTTCAAGAGCTTCATCAGTTAACAAACTCAGTGAACCTAATTTTTCTTGAATTAGCGAATAACTCGAAAGAACTTGTTGTATTTCCATGCGAGCTGCCCAAATTTCATGGTGAAACTTG

>JAP03156.C|Supercontig_0000159:911319-911468

AGCACCACGTATCGCACCGGCCAATACGTTGGAATAAAGTAGGTTTTGATGTTCAGCAGGAAGATCAACAAACTCTGTTAATGGATTATTCTCCAATACAAGACTGAATTCATCACCAGCTGAACTGAATTTACAAATGGTTGGAGTTAT

>JAP03165.C|Supercontig_0000058:1682903-1683036

CTTCAGATCCCTCTAGATTTAAATGACTGCTCTCCAGAAGAAAGACAGAAACGTCTTCTTAGACGCGCTCCTCGATCAAAGTTGACTTTGCTAGAACAGATAGAAGACACTTTTGACCAAGAAGCTTACAATTT

>JAP03167.C|Supercontig_0000053:258955-259025

CTAGGTAACCATACAGTTAACTTTTGCTCTGGAAGGCCACTACGCCATAAATTCGACCATGAGTTCTCAAC

>JAP03173.C|Supercontig_0000465:43123-43647

TGTTTGTATGGATTTTATTACTTCACACTGGTTTTGTCATACAGCTTTTCGGAACAACCTATTTTCAGACAATTTACAGTAACGAATATAAGTCAAGAATTGAAAAATTCCGTTGATAAAATTTTAAAGCCAAATTTCCCCAATGAGTATAATTTGTTCCATTGGACTACGGTGACAGGATTTAAAGAAATTATTAATAATACTCGTGTAGTATGGTTAATTTTTGGTGGATCAATTGACAGGGTAAACGTAAGTTCAGTGAATCCATTCGCAAAGAAAGCAGTCGGGGTTATTGCTTGTCGGTTAACTTACAATTCTGAGTATTCATTATGGCTACATCTAGATGAATGTTCTCCAATGGAAGTTAAAAACATATCAGATCAAGAAAATGAGCAGGTTCCAAAACATCATGAAGGCACCACCCTTTTAGGAGTGAGCCTTGGTAGTCTTGAGTTTGAAAAACCCATTAGTGGAGTAGTTATTGTTTATTGTGATCCTCTTTGGCATACTGGTGAAAGGAAATAT

>JAP03181.C|Supercontig_0000093:374848-375151

CAAGTGCCAGTTTCTCGAGTTCCTTACGTGAATATTCCAATGTACTACTCGACAAGTCAATAGATTCAGATTCAGCATTCATATTAAGTTCGTTTTCAATACTTGAATCACTTTCAGATCCATTTAGGAGTAAACGATTCTGAACATCTAAAATCACTTTACCAACATGTCCTGGAACGTTAGGAGGTAGTAAATTTATGTGACACGGTTTTAAACTATCTGATGAACGGCACCATGCTGGTTCTCTGGAAATCTGTTTTCGTATGTAACATGAAGCGCGGTAACACCAGTCTGAACAAAATGG

>JAP03195.C|Supercontig_0000306:306294-306452

TTCATCTATTGGTTCAATTCAATCAACTAATCATAAAGGATTAATTGTTACATGTGGTTTAGTGATACATTCATTAGCAGATGGATTAGCAATTGGTTCAGCATTTGCATTGAATCAATTACAATTGGAATTAATATTATTTTTAGCTATTATTTTACA

>JAP03209.C|Supercontig_0000081:200593-200770

ATTAAATATTACCATCACTTATTCCAATTCCTGGTGTTATTTGTTGGTTTATGCTTTGTTTTAATTCAGCATTTTCACTTTCCAACATCTCTACCTTTTGTTTTAATTCTGCGATTTCTACTTTCATTGCTTCGATATCTGAAGGATCTGGACTTTCTGCTCGTAGGTGCTTCTTCAT

>JAP03235.C|Supercontig_0000130:1735865-1736367

TCACCAAGGGAAGGGTGCTTTTTCACGGAGAGCTCTCGAACCAAATTTGTTCCATTCGGCTGGTGTAATCCACAAGTCATTTATCAAGTCAGAAGTTAGAAGCAACCTAGCACCAGACCATGAGAGGTCTCCAGGCTCAGAGTGATGAATGATTTCAACTGTTGGTATGTGGCTTGATACGGTTGTTGCTGCTTGCTTCTCCCGTGCTATTGCGATTAGCTGCTCAGTCAACCATCTTTCCATTATTTGATTATTAACACCAGCTACCCCTCCACCAGTGAGGATAATACATCCCAACAAACGTCGACGTAGTTCGTCACTTACGGAGTTTGAACTTTGCGAAGGCTTTCCGGGTGCAAGACCAGAGGCTTGAACAGTAACTTCTGGTCCTACCTGAGAACCAACTAAGTTTGCACACTGATTGATACTCCACCAAACTGCCTCTGGAAGAGATTCGAAGGAGTTAGGTTTTTGTGGAAGAGGGTTGCTTACTGGAGCCACCT

>JAP03254.C|Supercontig_0000196:366114-366437

ACGACGTATGCGTCTTTTACTTCGAATAATAAGACGTTTAGTATTTTCAGATCCTGTTTGTCGTTCTCGACTTGATTCTACTGCAAATTCAATGAAATGTAACTCAAATTTATTCACTACATTAAAGTATCTATTAGTTATAACACAACGTTCAGCAGATGCTTCAACAAGTAAACTTTTGAAAGAAATAACTGATCTTTTATCACCTAAGCTGCATTCAAATCATAATAGAAATGCTATAAGTAGTGATAGAAGTTATCATAGTCGTTCTGGAACTGTACAACCGGAATCTTCATCAAACAGAATTATTTCAAAAGCTGTTGC

>JAP03256.C|Supercontig_0000466:446407-446598

TCATTCATTTAATGGACATGATGGTAAATGAACTGTCCCAAAATAATTTAATTCATATAAACTACAAAATACAAGTACAATTATATAACCAATCAATAATGCATAACCATAAGTTTTAGTTAAACGCCAACGATTGATATGGGTTGCCATTAAAAGATAAATCACGGTTGAGAATAAGGTTAATGTTGAATA

>JAP03259.C|Supercontig_0000058:230583-230736

ATGGGATCCTGATGAAAGAATTACACCAAATGAAGCATTAAATCATGAATGGATTATACGTGAAAATGAACAAACTAAACAAAATGTTACATTTCAAACATTGGAACAAACTAATTCAGAGAATAAACAGAAAGATACAATTGCTTGAATCAAT

>JAP03278.C|Supercontig_0000345:250051-250311

TGGTTGGTTGTTATTTAAAAGTCTACAGTCTCTATTCACATCATCCACGTTTGGGTCCTAAATTAGTTATGATTCGAAGTATGCTAATTGAATTGCTAATGTTTATATTCATACTTGTCGTTGTACTGGTTAGTTATGGTGTATCACAACAAGTATTACTTTATCCATATCGTGCTGATTTCTCATGGAGAACTTTACGTGATATACTTTATTATCCTTATTGGAATTTATATGGTGAAATTATGTTGGAATATGCTTTTG

>JAP03298.C|Supercontig_0000235:490030-490194

AAACCGAAAAACCCAAGTGATTTACGTATGAGTAAAGGAAAGGTGTTGAATTATCTCCGGATACCGAAAGCTGTTGATTTCATGTCTCCAGATCTAACTGAATATGTTACAGAAACCCAAAGGGCTGGTCTAATTGCACAACTTAAAGTTAATAAAGACTAGTTT

>JAP03300.C|Supercontig_0000160:76180-76436

TCTAAACCATTTACCATAAGATAATGTATAATATAAAATAATAATGGAATTGGTTGTTCAATTAATTCTGTAGAAGATTTTTGATTATTTAGTGCATTTTTGGCATTACTATTATTGTTATTATGATTTTTAAGAATCAACGATTTAATATTTACAGTTGGTTTAATTTGTAATGTTTGACATAGAAATTGTCTAGGTAGATTATGTAAATTACCATGACCAGATTCCTGACGAAAAACAGATTCTGTAAATGATAA

>JAP03309.C|Supercontig_0000220:102076-102265

ATCGTCTGACTCTGTGTATGCGGAGAGGAAAGCATCAGCATATTCGGATCCAGAGTCTTCCTCAGAAGTAACAATTTCCATTGGGACATCATCCAAATTTACTTCACATAACGACGTTTGCATGATGTCATTTGAAGGACCTAATAAACACACTTTATGTAAATGAACTCGATATACTGCTTGTTGGTGG

>JAP03312.C|Supercontig_0000093:677199-677375

ATCAATGTGAATATTGAAAATTCACCGTCTTCATCACCACATTCCTATTCACCAAATTTCGGAAGACGTTTAACAGCTTCTGTACCATTTGATTGTGCTTCGAATTCGCCGATAATTAGCAGTACTGCTTTTCGAAATCCAACTTATAGCAGTGATCATGCAAGTCCAAATTCAGAA

>JAP03313.C|Supercontig_0000600:67742-68027

GAGCAAGAAAATCAGCTAGATAAAGTGATACTTTTGCTTAAGGCTGCTGGAAATGCTCCTGTAATGAAAAAGATAAAGTGGTCTGTAAGCAGGAAGCAGAAAATTCATTGGGTAGTGGATTTTATAAGAAAATATATATCATGTGCTCCTTCTGAATCTTTATTCCTCTATGTAAATCAGTGCTTTGCACCTGCCATGGATGCTGAGATAGGTTCATTGTATGACTGTTTCAGTGTAGAAGGAAAACTAATTTTGCATTATTGTAAAACTCAAGCATGGGGATGAA

>JAP03317.C|Supercontig_0019082:23692-23828

ACCACTTTGATAAAACGTATTCCATGCAGCAAAACACAAACTGGGGAACTGTGCAGCAAAACCGAAATAGCTAAGAAGATTGATACGATAGTCCGCATTAGAGCTCGTTTTTGTATTTAACTTGTAATCAAAATACT

>JAP03318.C|Supercontig_0000178:119147-119618

AATCTGTCAATTTCATCACCAAACAAATCAGAAAATACTAAATTATCTCGTCCAAGTTCTTCACTTATTCCTGCATTTGTTTCAGATTTTTATCTCTCTAAAAGGTATGTTCCCCCTAAGCACAGAGGGTTAGCCACAGTAATTGAAGATTCTGTTAAACGGAGTCAGCGACGTCCTAGATCGTACAAAAAGACTGAACTTTCTCCGATAAGGCGACAAATATTTACAGGCCAAACTCTTGAAGAAGCTTGTGCCCCTACTGCTGTTGGTATTGCTCCGCTAGACTCAGAGCTGGTGTTGTCAGTTACACGTAAGCGGGTTATCAGGCGTCAAAAGCTTGCGAAAAAGTTAGGTTTTCGTGGCATTAGTATATCCAAGCAGACTGAAGAGAACTTTGCTAAATTTCTTTCACAATCGAATTTGAATTCTTCTCACAGTAGCTCAGCATCTGTGTGAGTAGAAATGCAACTGT

>JAP03337.C|Supercontig_0000344:76389-77070

GTTCTAGCCACAACATTAATTCGTGATGCATGTGAGACAAGATTAGAAATAAGATTTAAACAAGATCTTTTATCTGTACAATTGTATGAACATTATTATAGTAAATCATGTGTCATACTATACTTTTTGTTAACTATAATTAATCTTTCAACAATCATATTAGAATATCCAGGAAATATATGGATTAATGGAAAACCTGTACCATATTATATTCCATTAGTTATTAATTTATTATGTGAGTGCTATTTTTATTATAGATGGTATATTATTTATGTTATGTCAGAAAAGAAGACATTAAAATCAAATATTTCATTCATTGCAACAATTACTATATTATCATTAATGACAATTGATGCCATTATTTATATCATATTTCATGAATTAAATTTTTCTAGTCCTGTAAGATGGTCAAGAGCTCTTAGACCGGTACTACTATTGACATTTCCAGAGAATAGAAGATTGCGTGCAGCGTTTTTTAACTTACGACGTACATTAGTCGATGTATTACCTGTATTTGGTTTGTTTGGTGCATGTTTAATATTTATTTCAATTGTTAGTTTAACACTATTAGGTGATATGAAACTGACATATCCTAATGGCAATAAGTATCTATATGATTTTATTGATGTATTATGGGAATTTTATGTTTTAACAACAACAGCCAATTCTCCTGATGTTATAC

>JAP03373.C|Supercontig_0000362:114443-114608

CGGATATTTTTATGGGCCGCAACTTCCAACATATTCATCCACGTAGTTGCTTCTCTTGTAGCATTTTACAATTTGAGGGCGCATAAATCTTCTAGGATATGTGCTCCTATCTTCATACTTGTCATGGGGGTTTTAAATACGATATCATTCATGTTAATTTCAAGTA

>JAP03382.C|Supercontig_0000310:159682-159840

ATTATTATTATGATGATTATGATTATTTGAGTTAAATATTGTCATCGGAGCTATTGAATCATTGATCTGATCAACTTTAAATTGAAATGTTTTATAATCGGAATCAACATAGGAGTATAATAAAGCTGGTGTTCTTGAAAAATAGTCTCCAGACATGTT

>JAP03394.C|Supercontig_0000005:3317468-3317617

ATGCATATTACAAGAGCAAGAACAATCAGATTTATTAGGAAACAACTCGGAGGGATACTTCGGAGTGACCAAACCACATGTATGTCTTTGACAGGAAGAGCAAGCAGTAGAACAACCACTTGTGGAAGACGTTCGACGTTCGTTGCGAGA

>JAP03399.C|Supercontig_0000029:537530-537694

ATTTCATCTTTTTTACAATATTCTATAACTACTCATCCACTAATTCATTTTGGTTGGCTAAATAAATATTCACGACGTGGATTTCAACCTAGATTAGTTTTTCTATTCACAGATCGTTTAATCTATACTAGTCGAATACGTGGTGTAGCTGGCTTATACTTAAAA

>JAP03402.C|Supercontig_0000042:62785-63155

AGCAAATTCGACATATTACAGAAACTGCAACCGTTCAGGGTGTTCCAGCTTTTCCTACTGGTGTTCCATTTACATTTGTTGAACATTATATTTATTTGGTTCGAGAGACAGCAATTGCTTTCGGCATATTTTGTACTGTAATTCTGTTTACTGGATTAATTTTATTCTCGTCTCCGATTACTGTTTCACTTCTACTTATAGTTGGGGCTGTCGGTGGAGCGTGTTCTGCTATCATCGGTTTAGTTATTCTTAATTTGGCTTTAAATCCTATTTCTGCTTGTTTGTTACTAGTGTCCAGTGGTTTAGGGGCTAGGATATCTGTTGGATTTCTTGGATCGTGGCCAGTATCCCATTTGTATACAGATTCATCT

>JAP03409.C|Supercontig_0000005:81345-81409

CGTTCTTTAGCTTCAAGTGCGGCTAACTCCAACTCCTTAGCCTGTTGTGCTTGATAAGATGGAGA

>JAP03414.C|Supercontig_0000000:3871352-3871694

ACTACATATCTTCATATTCAACTCGAACACGTTTTAAACGACTAACACGATAACTTGGATGTTTTTTAGCAGCAGCTAAATTACTAACAATAGTTGACCGACCAGGATAAGCATTAGCTTTGAAAAAATTATCTTCATTTGGATTATAACGTACTGGTTCTAATTGTATATTTAAATTAGATGGTAATTGACAAATATTATTTTGTGCATCTGATGTTAATTTATATAAAAAATCTGGTGTTTCTACAGGTGTCATAAAATAATTGATTAAATCCTCGATGGAATTAATTTCGTGAAGGTAACTATTGGCAATGTGATGTTTGAACTCCGTGATACATGCAGT

>JAP03438.C|Supercontig_0000295:759171-759600

ACTTTACAGATGAGTGCTCTATGTTACCTCACGAGAGTAGAATCATTCTCCGCCTGTCATCGCCTTCATTCACAACATCTTAGTGATGAAGAAAATATTAAAATGTTTCAAAAATGTAATAATCCTATGGGACATGGTCATAATTACAAACTGGAGATCACAGTTTCAGGACCTATTGATCAAAGAACCGGTATGGTTATGAACATAAGTGACTTGAAATCAGTTATTCAAAAACATGTTCTTGACCTATTAGATCATAAAAATATCGATGAAGATGTTGAATATTTTAAAAAGAATAGTATTGTATCAACTACGGAAAACCTAGCAGTTTTTATATGGTCACAATTAGTCAATGCTGTCCCTAATAATTTACTTTATGAAGTTAAAATATGGGAAACTGAAAAGAATATCGTAACTTACAGAGGTGAAA

>JAP03452.C|Supercontig_0000155:90803-90910

TGCAATAATGAAAAATGGTAACCAGCATAGACAAAAACATCCCATAATAATTCCAAGTGTTTTAACAGCTTTCGATTCATTTTGATGACGTGAATTACGTTTTTTCAC

>JAP03453.C|Supercontig_0000071:471011-471140

GAAGCACGTCGTAATTCTAAAGCTGCTGCAATTATCTCGCTTTCATCAGACTGAGAACTGGTACTGCTTACATCTAGAACTACATCACCTGAGACACCACCGCGTAAATAATTGCGACATTGAAATGTTA

>JAP03457.C|Supercontig_0000119:499243-499392

TTTTTAGGTTGTCCTTTTTCCCAAACTGCGTGATATTTACTGCCATTTTGAAAGTGCAGAGTTCCTTCTCCGTGAAACATGCCGTCAAACATTTCCCCTTCGTAATATGTCCCAGTAGGCAGTGAATACTTCCCAAATCCCTCCAACCTT

>JAP03468.C|Supercontig_0000489:297554-297811

GGTTCTTTTTGAACAGTTCAGTGTATTTTTAAATCTCGTCTTCCTTGTAATTGCGTGTAGTCAATTTATTCCTGAACTTGGTGTGGGTCGTCTGTATACATATTGGGGGCCACTTGGTTTTGTGATTGCAGTGACAATGATTCGAGAAGCGGTAGATGACATTGGAAGATGGTCTCGTGATCGTGACGTCAATATGGCTATCTATGGAAAATATGTCCGCAACAAAGAAGTTGCTGTTATGAGTTCAGATATCAAAGT

>JAP03479.C|Supercontig_0000156:1423985-1424183

TGATTGAGCCGCACACCAATTCGAACATCTTCAGTGCCCATCATCTTTTTAGCAAACATTTTTATTTCTTTAATAGCTCGAGGAGCTCGCCTTTTGAAACCAACACCATGAATTCGTTTGTGCAAATGAATTGTATATTCGCGAGTAGCAACACCTTTTCTGGCAGTTCTTCTAGCTTTCGTTCCAGATTGCACCATGG

>JAP03485.C|Supercontig_0000091:939598-939742

GATAATCCATATTCATGTACAATACGTAGTAATTCTTCACCTTTAGACGGTGGTAAAATTTCACTAGTATCACGTAACACTGTCACTGGACGATTATCATAGCATTCAACTAAGATATGCTTAATGACATGATTTGGAATATGCT

>JAP03495.C|Supercontig_0000148:960980-961157

TTCTTGCCTTCTTGCGCAGTTACTTCAACAACGCTTTGACAAAATTCTTGAATAAACTGCTCACATAGAAACCCGATCGAAACCAGTGCCTCAGAAGTAACTAGAGAAACAGATGGCACCGTTTTTACTATTATTTTAACGCGTGAGAGAGGAAGTTTAATTAACTTTTCTGTAGCAG

>JAP03497.C|Supercontig_0000263:162644-162830

ATTAAAATTCTACTGAACCAGTTATCATAGCTACAATATGAGTTTCTATAGGATGACAATAGAGTATAGTCATATTGTATTGTATTTCATTTTCTGAATGTCGAAAATCGTATAATTCCTTGACAAACCATGGAAATGTTGGTGGACCATAAAAAATAAATGTTTGAGGTCCACGAATTTTTCGCCT

>JAP03501.C|Supercontig_0000067:541085-541204

AAACGTTTAACTTTTGCTAGTCAAGGACAATTAGCACCATTATGTGCTATTTTTGGTGGAATAGCTGCACAAGAAGCTATGAAAGCGATCACATTTACATTTACACCAATTAATCAATGG

>JAP03503.C|Supercontig_0000055:521754-521924

TTCATAATGTTTATGGACATAAAGTTCTGTGCCTGAGATATTGCACAATTCCAGTAGTTTGATGCTTCGCTGTCTTTCCGTTAATACAATAAGACAAGAGATCAGACCAACTAACAGACCATAAGTTACATCAAGTATGAGAGTTCCCAAAAATGTTACGGTGAATAGAAA

>JAP03515.C|Supercontig_0000277:225809-225950

TGCTCAGTGAATCTTTCCTAAACGTAAAGTATTCACTCATATGTTTCCAAAATGTTAAATATCCTGTCTTGCACCTGCTCCTGCAATACAACGCAATAGACTTTCTCAATTTACTCACTTTGTCAGCTTCTGATGAATTTTT

>JAP03561.C|Supercontig_0000071:706095-706245

TCTGTTCGTGTTGATGAATATGCTGCTGCTCTTGAGCAAATACATATATTTATGAAGGTGAAGAACTTTCTCGTTTTCCAAGGTGCTGTCGAAAGTATAGCTATGAAAAATGCACGTGAACGTTGTCAGATGTTTGAGGAAATCAGCAAGT

>JAP03598.C|Supercontig_0000122:180569-180838

TATGTCGACCATGTGATTCTAATTGTCGAACTTGTTCTGGACCTAATCCATTTCAATGTACCAGTTGTAATAGGAAACCACCAGCTCCACGTTGCTTATTGACAAATCAACGTGTTTCAAAATTACACAGAACTTCTAAAGATTTAGCCCCATGGACTGATTATTCAAATGTAGAACCTAATTCTGGACAGTGTTTACTTTGTTGCCCTTATCGGTTAGCTTTAACTAGTCGTGATCCTAGACAATGCATGTTTTGTGTTGCTGATAAAC

>JAP03619.C|Supercontig_0000367:89700-89773

GGTTTTATATTAATCATGAAGAAAAGACTACTAGTTGGGATGATCCACGACCTGCATATTATTCTGCTCAACAA

>JAP03667.C|Supercontig_0000272:212978-213291

ATGTTTGCACGGCTACGTAATTATGAACGTGTAATATCTGATGCGTACGTCATCAATCAAAAAATACACACAGGAAACACAATTTTCAAGAACACTCGTGCTGGGGTGGTTCGTCGAACTAGAGATCAGTCTCACCCCCTCACCTACGAGCAGGCAAAGAAGCCACATCAAATTGGTTTAATGAAATCCTGGAATTCTTGGAATACTTCAAATTTAGAAGGAGAGGACCGTTACACTTCAGAAGTTACCCTTCATGACCAGTTTATTCGAACATTTATCAATGGAACTTTCCCTTTGTTGGTCCAAAGTGAAGT

>JAP03674.C|Supercontig_0002003:7197-7390

CTTTAAGTGCTTCGGTCGCTGAAATGTTATTCGAGACATATATGATTCACTAAGTGGAGGAAATCCAACACCCAGACAAGCTAGCATGTTATTTTCACCTTGTTCCAAAACAGTTGATTGGTTACACCAAGGTAGAAATTCTGAATAACGACCAACGTCAATTGCAATATCAAACATGTTTTCCGGAGAATAAC

>JAP03684.C|Supercontig_0000311:431796-431867

ACTGTTGTTGGTGGACAGACACGAGTGAGTACCATGCTTGAATTTTGTTGATTTACACGAACGATAAAATTC

>JAP03688.C|Supercontig_0000186:130535-130647

ACTCTATGTAATGTTGTAGAGATTGAACGTAATTTGATTTCTGTAGAGTTAACAATAAATTGTGGACCAGTCTCCTCCCAAGAAGTAGGAACATTTGCATTTCGATCAGCATA

>JAP03721.C|Supercontig_0000220:10651-10811

AGTGGTAAACAACGCTGTTTAGACTATTGGTCAAAATACAGCTTAACTATGAAAGAATGTGGGTCCCAGTGCGCAAACATTTGGTGTGTATACTTTTGTACACTATCCGTATTTCCGGCAGTTCAGTCACGTGTTAGACCAATAAATCCAGAATATTTTAT

>JAP03726.C|Supercontig_0000155:341663-341888

AATAATCATTAGTTCCACCGAGATAAACTAATAGTTCATTTGTATGAATTAAACGCCCACGGACAAGCGCTTTGCGACTAAATGGAATATACGCATCTTTATACACCGACTTTTGCAACTCCTGAATTTTATTACTAACAGACTCATAATCTGATCTATATTTTTCCAAGCTGATAATCTCCTGTTCAGTAGTTTCAATATCAGAAGTTTGTCGAGAAAGTAACCG

>JAP03728.C|Supercontig_0000089:8384-8487

AAATCTAGACTTCTTTATGGATCAAGTTTAATGCCAACAGTTGCTGTACCAGTTTCAATTCCAGTACAAGCACAAATTCCTGTTTCAGTGCCTGTATCACAACC

>JAP03733.C|Supercontig_0000028:660335-660487

CCTCCTAATCGATACGCTGATGCAATTGCACGTTTAAATAAATTATTCGATGAAGCTGAAACAATTAATTCTTCTGTTTGTCTGGAGAACTTGTTTGGTTGCTTAACAGCTTATCTGATATTTTTATGCATGAAGACACATTATGATAAGGTA

>JAP03735.C|Supercontig_0000184:630504-630661

AATAAAGCTGGAGGAACAATTCATTGTATAAGAGCAGATAATAAAGCAGCTATTGTTGTTCATGGATGTACAGAAGAATACCAAACTCTAGCTGATGAATTACCTGAAATCATTATGAAACAAATTGGACCTAATATGATAGCTCATCCTATGATATT

>JAP03738.C|Supercontig_0000248:129242-129392

CCGCATAAAAAAATTAACACTGCCATAAGCCATGTTAAAACACCAACATGATATGTTGTTCTAGTCACAGTAACATGTTGACAACAATCACATCGAAAAGATTTTGGGGAACGACCAAGTTTTTTAGTAGGATCTATTTCTGATTCAGGAA

>JAP03745.C|Supercontig_0002055:172902-173101

CCTTCAGGTGGATTATACTTCAGTTTTTTTACTACAACCTCAACAGGTTTATTGCTTTACACAGAAAATAACATTACTGGACAATTTCTTGCACTCACGTTGTTACATAATAGTAGAATAAAATTGGAACTGGAATTACGTTCACCTCAATATCGTATAACAAGAGCACACACAACTATTACGTTAGATTATTCAGAAAA

>JAP03756.C|Supercontig_0000028:1934650-1934733

AGAATAATGATGCGTTCATAGTTCGTTTTGAGGATCTAATCAGTAGATTAGAAAACTATCAACTTCACAACAGGAAGAGCGAGG

>JAP03763.C|Supercontig_0000304:315820-316056

TTTGTTCTAATAGCTAACCGGAATAGCGTATAATGATCCGTATTTATCTTTGATAGGAAAACTTTTTCGCATACTTAAACACCTTTGATCAATAGATAGTGAATTTTCTTTGGTTTCTATTTCTTGTTCAAGGGAAGTCCGACTCTTTTGTAATCTAGCTAATGTGATGTTTGCATTCCTGCGGTTGTTTTTAAGTTCTTCGATTGTTTTTCTAAGTTCCAAAACTTCAGTCTGAAG

>JAP03774.C|Supercontig_0000074:751457-751700

AGATTAATCATTGGTTTGCAGTTTCGGCATGGAAGTGGTGTACTAACGATGATGACTGTGGTATTTGTCGGAACGCCTTCGAAACATGTTGTGCAGATTGTAAACTCCCAGGTGACGATTGTCCTCTTGTTTGGGGTCAATGTAACCATTGTTTTCATATGCACTGCATTATAAAATGGTTAAATAGTCAACAAACGGCTCAACATTGTCCTTTGTGCAGACAAGATTGGCGGTTCAGAGAATA

>JAP03788.C|Supercontig_0000005:1299594-1299755

AAATCACTCCATATTACTTTCCAATGTCCTCCGTTCTGAGGTACATAACAATCAATACATATGATCTGATTCTGATTTTGTGGAGTAAGTTGAACACCTGAAATATTTATTGATTCATTATAATGAAGTAGTAATAGTCTGGCCAATAATCTAGTTGAATGA

>JAP03797.C|Supercontig_0000064:2472398-2472600

AGTTGGTCTCCTACACGTCCATCCGTATTTTATACATGTCGTGTTGATGGTAGTATAGAAGTTTGGGATTTACTTGATAAAACTTATGAACCAACAATGATCCAATCAATATCTGCAAATTCACTAACTGCTATATCTATATGGGATTCACCTAAACGTCAATTTATAGCTACTGGTGATATTCAAGGAGTACTACAATTATT

>JAP03800.C|Supercontig_0000046:123526-123668

ATGATTTTGGAAAGAGCACACGAAGCGGCCATATTTGGTCATTCTTCCGTTCCTTTACCTTCCATTCTCGGTACAGATCCAGCTGAAGGCAAAGATAAAATAGTTTCTTCTGTGGATACAGTGAAGCAACTACTTCATCATTC

>JAP03801.C|Supercontig_0000228:609576-609703

TGGATTATTTTATTTATGTTTGGTTATTCATTTGGCTGGGGACCATTACCAGTACTTATTAGCATGGAATTGTTTCCAGTTAGTAGTCGTGGAAATGCTTTAAGTGCTGCTATTGCTGTCAATTGGAT

>JAP03825.C|Supercontig_0016440:795-1103

TAATTTATCCCTGAAGCTATTTATACAAGAAAATTTCATAGGTGGTACATGAGCTTCTGTAAAACGTTGTTCTACTTCATTAAAATTTTCTTCAAAATTCACCCATCTTGCTGTTTCCAACCAAAGAACTCGAAAATGAGCTGCAACTGGCATTTTAAAAATTGATTCATTGAATTGTAATCCACCATCCACTCTTGGTACTAATTCTTGAAGTTCAATAAACATTTGTGTTGGATAAGACCCAGTATGAAAACTGAGTTTATATCCTTCTCTATTGTCGGAACCTCCACTGTGCTCTTCCTCAACAAC

>JAP03837.C|Supercontig_0000292:784272-784408

ACTAAGACCACGAGAATGACCGTTGACTCCATATGAGTGGGGTGAATGTGGTAATTTGCGAGTGCATACCAACGAATGGCGAGTTGAACTTCGCAAAGGGTTGCTCAACTGATTCATTCTTTGGAACCAACGGGTAA

>JAP03856.C|Supercontig_0000480:540032-540174

CCATAAATTTCTTGCTTGAACTGCTTCAATACGTACATATCGATTGGGATCATCAACTAATTGATCTAACAATGGTAGAATATTTACATCACGATGACGATTAATCAATTGTTCTGGGAATAATTTTATAATGTTGCACAAAC

>JAP03860.C|Supercontig_0000021:564534-564648

GTGCTTAAAAATCGACCACCAAAACCTGTAATTATACGTCCACCACCAAGTCAACCTTTTACAATATGGACAAGTGATTGTAGAGAACGACTTGAGCATGTTTCAGAAGCAATCG

>JAP03866.C|Supercontig_0000023:647028-647387

ATCACTGGATTCGATGCTGAACGGTATAAAGTCATCAAGGGTGAATATACACCACTCGCATGTTATGGTGAATTACGAATTAATTATTGTATCACACGTGATATCCCTTTCCTATCAAATTCATTTATTACAAATTGGTCTCAAACACAAGTTAATCTTGACAGACAATTGTACGATAAATGATACAAGTGTAGATTTGTTTTCTAGATTTAATGAAGCCTTTGATTTACCCTTACCATATAATGCCTTGTATGACTATAATGGAATTTGTTTTGTCCTGTCATCTACGTATACGGATAAAAAAGGTATGCATGAACAGTTAATAGCTGGTGCGAACATGAAATTATATAATTCTAAAAA

>JAP03891.C|Supercontig_0000030:1167304-1167464

TGCTGTAATCCAACATGTAAAAATCCAGTTTCTTATGATCCTCGTTGGAATGGTCAGTATTGTTCAACTAAATGTGTTGGAGAACATTGTCAATTAACCTTTATTGATTGGTGTAATAATAAACGATATGATGGAAAGGTAAATGTATTATCTAAACTACC

>JAP03903.C|Supercontig_0000488:214715-214937

AGAACAATACTTGCAATTCATCCCTGTTAATGGAGTTAGACATGACACCTATTTCTATGAACATCCTGGTCTGAGAAATCTCATTGAAATAGATGCCACTATTAAACAGCAGGTTCGTATCAGGATTGTCGAACATCTGAACGAGCTTCCTGTGCTAAGAATCCATTGCATTGTTGCTCCACTCGATTCGACTCATGAACTCGTCTCCGCTTACTTATGTCGC

>JAP03944.C|Supercontig_0000044:573994-574278

GTAAAAATAAATAAGGCACTATTATCATATGCATATACGTCACGTTATTCTCAGAGTGAACAATATATTTCTGATCTGAAAAGAATTGCAGCTGTTGAAGGAATGAATCTTGATGACTATATTAATAAAACGGTCTTAAGTAGACATAATTCAGACCGTTCAGACGAACCTCATACTTCTAAAGATCTGTTGTCGAATACTTCACCTCAGTTTATATCGAGATCTTCTATTATGATAAACTTGCTGTACAACTACGCTGTTGTATTATTTTATCAACATCAGTAT

>JAP03947.C|Supercontig_0000238:471150-471474

TATTTGAAGCTTTGATAACTAGGCGAAGAACACCAGTATTGGCAGCTGTAGGACGTCCTTGGGCTGCTGCAGATCGTGTAGTAGTCGAAGTAATTACACCAGAACGAATAGCCTTTTCTATACTTCTTGCACGTCTGGATACAGGAGTCACAGGTGTATTAGACTGAGTCACAGTATCAGGAGATTGATATTCATTGGTGGAACCATAGATGAGCTCTGAAGGTAATTGCTTCATATCTGCTTCTGTAAGCCAATAGGGACCATATAAACAACCAATTTCGGTATAATTGTTATCTTCACCACAGAAGGCACATATCCAAAGACT

>JAP03949.C|Supercontig_0000279:42316-42541

CAAGATGTTTACAGTCAAGTCACTAGTGGTGCTTCAGATTCAATCTCCAATTTAGCGGCTACATTTCAGGCAGTCGATGCTTCGTCACCTCAGTATACAACTGAATTTTTAGCGGAATTACTAACCCGTGTATTGGATAGCAATCCACGATTATCTGCCAATGTTAGGCGTCGTGTATTAGATCGCTTACGCATGAATTCGTCCGACTCTTAATGTAAATTATTTT

>JAP03951.C|Supercontig_0000110:50754-50902

ACTTGAAACCGGGATAGTGGATATAATAACGGCACTTCTACAGGTGGTTGACCATGGGCGCTAGTTTGTAGAAAATAATGAAATTGACCATTGACTGAATGTGCAACAGCTTTTTCAATAAATTCCACCATAGTGTTTGCACTATGTGA

>JAP03965.C|Supercontig_0000306:113384-113586

AACAAATTGAATCTGGTGAATATACATTTGAAGAACTTGCCCGAACTGAAAGCGATTGTAGTTCAGCTCACTCTGGCGGTGATTTAAATTTCTTTAGTCGTGGTCAAATGCAAAAGCCATTTGAAGATGCTGCATTTAAATTAGAAATTGGTGAAATGTGTGGTCCTGTATATACAGATTCTGGAATTCATTTAATCAAACGA

>JAP03974.C|Supercontig_0000617:107717-107937

ACCTTTTTATTGACAGCTTCGGCAAGTAGGTACAGCATCTCATTCGTTTTCTCAGCCTCCTTACCAGCAACAATGCTCATTATTCTGACAGGAATGGTTCGTTCATGCACTACCGTCAAAAAATCGACCAACTTTTGAAGAAATGCCAACTTTTTATCTTTGTCTTTAACATTATCAGCTTCTAATTCTTCAGAGTTAAATAAACCTTTCATTAGTCCTGT

>JAP03980.C|Supercontig_0000166:251918-252042

CTGCAATGACTCAAATGGTTGTAGTTTTATGCGATTGGGTTGGCTCATCACAATTAGCACAATCTTTAGCAATAACTATGGTTATTTTAGGATTTGCTATATCACCGGGACAATTTTTGATGGGT

>JAP03981.C|Supercontig_0000663:149689-149829

AAAGGTTACTTCAATTCTTGAAACATATCCTAATTGGGCAGATTATCGTGATCAAGGTCATTCTTTATTTATTTCTAATGTACCACAATCAATGACTACATATTTAACAGCTGGTCCAGCATCAGGTAGTTTACATAGTGG

>JAP03986.C|Supercontig_0000159:497172-497514

CTTGAAAAGTGTGCACAAATACTTTGTGACCGCCGTTGGGGAACCAATGACAGTCCATTAAACTCAATCCAGTGTCGCCGAAATGTCCTAATCGGGCCTTTTCTTTCCATAAACTGCACATATCTGATGAATCTAAAGTATTTTCACTAGGAGGTTGCCAAATGATTAGTGACTTATCCATACTGCAAGTAAGCAAATTAGGAGGGAATAGACCGTTTATTATTGGTCCCCAGCGAACACCAGTGACCCAATTTTCATGGCCGGCTAAGACACTTTCTGGCGCTACGCACACACGAATGTTATTAATAGCAAACCATTCAGGAAGCTGAAGACTACTGGCCTT

>JAP03989.C|Supercontig_0000476:317783-318082

CTTACCTTATGGACGTTAAAATTCGGTCGGATAAGCATGATTGCTAGTAAATTCCTTTCTAAAACGTTGCTTTTTGATAAGAACTCTTCCGCTTCTTTTTCACCTTCAGATGAATCTAACTTTTCTTTCGCCAACCGATATCCTACTAAGTCGGGTCTGTTGGCCACTTTTTTTCCAGCTCTTGTTGATAGCCAACTTAACTGCCATGATTTCGTTGGCCAAGTCGATGTTGATCTGTCTGTTTCATAAGCGAAGAAAGCACATGGTAGTGTGCAAGATAGTACCACATCACCCTGTGAA

>JAP03995.C|Supercontig_0000007:1675806-1675898

AGGTGAGTATCAAGAGGCTGAAGCAATTCTGCTACAGAATCATTTATATTTTCGTGCTATCATGCTCAATTTGCATGCATTTAAATGGAATAG

>JAP04000.C|Supercontig_0000021:2429546-2429688

CAATTTTATAACTTAAATGATCCGGTGTTCGAACAGGAAATGGTGAAGTTTCATTTTTATCATCGTATAGTTGAGAACCGGCAAAATCTGTAACAGGTGCTAAATGACTAAAATTACCAAGCCAAGGTAAACCAGTACATTCT

>JAP04005.C|Supercontig_0000130:1269387-1269469

GCTGCTGCTGTTGGCAAATTTGAAGGAAATATGGGAACTACTTTGGGAGACAAATCATTGTTCGTTGCTAATCATGCTTATTA

>JAP04027.C|Supercontig_0000086:14469-14763

GGTGATGGATCCCTTAGTTATTTATGTGAATGGATGTGGTGGATGGGTTTCATTTTAATGGGTATTGGTGAATTTGCCAATTTTGTAGCGTATACATTCGCTCCAGCTATACTAGTGACACCGTTAGGTGCACTATCTGTACTTGTTAGTGCACTGTTAAGTGTACGCTTTTTAAATGAACATTTAAATTGTATCGGTGGATTTGGATGCTGTGTATGCATATTAGGTTCAACTTTAATTGTGTTACATGCGCCAAAAGAACAAAATTTAACATCATTACATGAAATGTGGTCAA

>JAP04028.C|Supercontig_0000441:106121-106333

CTCTTTCATCTTTTGCATTATTAATACCCCAGTGTAGTAAGGAATTATATGAAGTTCTAGGACTTGGTGAATTCGATATAGATTCCATACTTGAAATACTACCTTGTTCACATGGTTTCCAATCAGTATCATCAGAAGTAAGTATTATAGATGGTGTGAGATTATCGTTATCTGTTTGAGTGTAAATTAAATCCATTTCATTCGATTCAATCA

>JAP04053.C|Supercontig_0000176:538531-538749

GGAATACAAAGCTGAATTGCATTATAGGTGTACAAAAATTGCACAAGAAATATCTAATAAAGAGCAATGTGAAGTCAATATGGATATAATTTGCTTGATGACTGAATTGATCTTTCGTTTTCATCAAATTCTTGCCACAGATCTAGAAACATTCAGTCGGCATGCAAAACGTTCAACCATAACTGCGGATGATGTCTTTTGTTTCGTCCGAAGAAATCC

>JAP04058.C|Supercontig_0000058:2306919-2307248

TGATCCTGTTGATTCCTGTGAAAAACATTCTACTGATGAAAATATCCGTTTACCTTCAAAACGTCATTATCGTCAAAGAGCTCATTGTAATCCCTGGTCAGACCACACACTTGATTATCCTTTAAAACCGGAATTGATGGATTGGGAGAAGCTGTTTGGAGGTGAACCAGTGTCTTCATTGTCTGTGAACAATAACTCTTTTGATCCTGTTGTTCGATTTTTGGATGTGGGTTGCGGCTATGGTGGATTGTTGTTTAATTTATCTACTTCTTATCCATTTACCCGAAGTGTAGGCTTAGAAATACGTTTGAAAGTCTTTGACTTTGTTCA

>JAP04075.C|Supercontig_0000042:1327976-1328088

AATAATAAACCTCAATTAGTTTCAGATGTATCAGTAAATGAACTAGCTTCATACATGGAACATATGGTTCATATTCCGGGCAGAATGTCTGAAATGGCCCAAAGAATGTATCT

>JAP04109.C|Supercontig_0000195:565785-565970

TGGATGAAGAAGAGCTAATCGATGGGGAAAGTAAACCCGATGAATTGTACACTCTCTGTGATGATCCTATGCAAGTACCCATCAAACTCATCGATGAACAGCTAAGGTTCGTAGACCTTGCAACAGTTCAAGACCTTTTTGTTGACAATTCAGGATTTCTTGTTGTTGGTGCCATTGGTTTACAGG

>JAP04126.C|Supercontig_0000417:143938-144113

GGATTCAGGCAGCAAGAAACACAATGCTCGTAGATAGAACAGCAGTGATTAGACTTGCATGAATTACAAAAAAAGCGTTGTGTTACGTTTTGAAACGGGCAACAACTGTTTGTCAGGAGATTAAAATAATTACACAGATATCCTAGTTCGTCAACAACAAGTATGGGACTTTGTTT

>JAP04131.C|Supercontig_0000311:811236-811648

ATCTTCTACAGCTATGCTTCGTGATTTAGAAATGCAAAAAGCTAAAGCTCAGCATGCCCAACTTCAGCTCCAATTACCTCCACCGCCACCACCACCACCTCCGCCTCCACCACATGCCATTGGTCTTCCTATTCCAAGTTTCCTTCTTAATATGCCTTTAAACACCAGCTTGACTAACATGCCGCCGCCTCCACCTCCGCCACCAATTATGGTTGGAGCGAAAGATACGAAGCCCTTGCTACCTCCCCCTCCTCCACCACCACCTTCATTCTCGCTAGGTGCTCCGCCACCCCCTCCACCGCCGCCTACTTTTGGAGTAAATTCTAATGGTTTAGGGACAAATGCTTCTTCAGCACCAATCCCACCACCGCCACCACCAACACCCCCAGCTTCTGTTCAACTGAATGGGAACT

>JAP04145.C|Supercontig_0000001:945660-945817

GGTATGAATTATATAGCAGCTGTACTACTACTTGTTCTTGATTGTCCGCCAAATGAGAGAGAAGTAAAAGCATTTTGGTTGTTGGATGCATTGATTAATCATATTTTGCCAAAATACTATTCTTCTGATATGTTAGCTGTACGTGTTGACTGTATGGT

>JAP04148.C|Supercontig_0000010:533320-533386

TGAATAACTTTCTTTAAATCGACCAAGCCCCGTTTACGTCTTCTTTCCATGTATGCAGAACCTGACT

>JAP04153.C|Supercontig_0002053:7071-7351

AGGAGAATTAAAGCCACCGAAACTTTCTCGAGACCCAGGTGATACATTGTAGAGTTTAAAAATACTTTTAACAAACTGATAACGATATTTCGACTTAAGGATGGTTGCTCTTTTGTTGATACGTTTTTCTACAGAAGAAGGAAGTTTGAATCCATACTCTTGTGAATTAGGAAGGATGTCTGGGACACCTTGATTAGTAGCTTTCTCCTTGGCGACGAGAGTTCGTCGCATAAAATTCATTTGGAGTAAGTTCTTCGATATACCAAGCTCACTAATATCTA

>JAP04163.C|Supercontig_0000043:1554926-1555216

AGACCCAAAATAACCAAATCATGGAGTTTACGGGACAGTTTGTTTAGTTCGAGACGTGGTCGAGGGTCGATTTCGGATACTCATGAATGGCAGTATATTGCCCCAAAGCTGTATCAGTTGGAAGATCAGTTTCATCATCCAGCTTCAAAATGTGATGTTTGGCGTTCTCTTTGTCCTCAAGAATTACCCGATGCAGCTGTTTATCCTACAATGCCTGTTTACAATTTGTCCAATAAAGTCTTTGCTTCATCTACAGGTCCCAAAAAGGGTTTGTTAGCAGTGAAAAAGTGG

>JAP04185.C|Supercontig_0000672:389116-389690

CATTTTCTTTTACAATGTATTAAACCTGGAGCATCTAAAAAAGCTCTATCGGCTGCTAATGCTAAATGGAACAGCCCATTACAGTATACTCAAGTAACATCTCCATTATATCCATTCACATCACCTATATGTCAAGACGATTCTGAGGATGATGAACGTAACAAAATGAATTTACCGAGGTGGAGTTCAGCCGGACATAGTATCGGTAATCGTAGTCATCGTGGTTTAAACCATAATTCTGATAAGGAAATTAATCATGATCCAGATAATAATAATAATTTGTGGCGTGCTAACTCAAGTAGTTTTATTAGCCGTAAATTTAAAAAATCAGCCAATAACTCTGATCAACGTCTTTTAAAACGTGTGCACATGGTTACACGTGATGTTTCTGAAATAGTCAAAGGTATACGTTTTATGCAAAAACAAAATGAGAAAAAGGAAAAAAACAACAAAATTATTTCTGAATGGAGAGCAGTCGGTATGGTGTTAGATCGTCTATTCTTTATTATCTACCTCTGTGCTTTAGGGATTTCCATATTACTTTATTTTCCAAGATCTGGAGAAGATTCAGAAGA

>JAP04186.C|Supercontig_0000123:364944-365254

CCACAACCTAAACATACTGGAATTAGAAATGTTATGATTACATCAGCATTGCCTTATGTAAACAATGTACCTCATCTGGGAAACATGATCGGATCGACTTTAAGTGCAAATGTATTCGCTTTGTATTGTGATTTGGCTGGATATAATGTGCTATCTATTTGTGGAACTGACGAATATGGAACTGCGACTGAAGCAAAAGCAGTCTCAGAGAATATGACTCCACGTCAGATCTGTGATAAGTTTCATAAGTTGCATTGCGATATATACAAATGGTTTCAAATTGAATTCGATTATTTTGGACGAACTACTAC

>JAP04193.C|Supercontig_0000310:573240-573406

CTAATCAAAGAGCTCAAATTATTGTTAAACCATCTGAAAATATTGATAAGAATTCTTTACTTTTATCATCAAAAATATCACAACATTCAATAGTTAATGAAGATCGTTCATGGCCACCTGAACATTCACATGAATTTGAAAGAGTTGATGTACGTGTATTAGGTGAA

>JAP04211.C|Supercontig_0000632:129344-129451

GGTCCAAAATGCGTCAATATTGGCAAACAGGTTGTCATCAAAAGAACTTCTGTCAGCATTCCAAATTTCATTTCATCTAATGGAAAAAATCAGTTATGTAATACTTAG

>JAP04251.C|Supercontig_0000058:1618741-1619051

GGGATCATTAAAATTGACTGATTAGTTATATCTTCATTTGGATCGAACATTTCATTATCCATTAATACTTCTAGTTCCTCATTTAACAAAGTTGGTGAATAATCATAAATAGATGATGAACGCTCTGTACAACTGGTGTTTTTCTCTTTGTAGTCATTAACATGTCTAGATGTTGCTAAAGACTGTCCATGAATAATATCCTGCACAGAATGTGGTAAAGAATCAGAGATCTGTTCCAGCTGGCCTAACAAGGCTGTAGCACGAATTATAGAGTCTTCCAGTGGAATCTTCATGGATAATTCATTCACATG

>JAP04253.C|Supercontig_0000243:35077-35249

CGAATTTCTCTCATTGCAATTTTACGAATGACTGGATCTTCTTCAGATGCTGTGAACTGTTTAATTGCCACTAATTGTCCTGATTTAATATCACGACATTTAAATACTACACCATATGCTCCTTCACCTATCTTACATATTTTCTCGTATTTCTCAAATAATAAATCATCATG

>JAP04281.C|Supercontig_0000088:812381-812588

AAGAAAGACTCAATTATAATGAATGTTTTCGTGAAAAATTGGAATCTGTATGAGAGTATGGTTTAATTTTAAATTCAACAAGTTCACAACCACGTATAAGATTTCGAATTTCTTCTACGTCTAGATTATCGCATGAGAGACCTTGAACTTCTAAAATTCTATCACCAACATGAAATACATCCCAAAGTTTAGCTGCACATCGAACACT

>JAP04284.C|Supercontig_0001997:38709-38853

CGTGCAGGCAACCTGAAACTTGCTGCAGCCTTCAGTCCAGACCGCAATGAAGACCATGCGTGCCAACACAAAAGATTATCCCACAAGGAGTCCGACCAATCATTCTTTAGCCACTCATTTTTTACGTACCTTGCAGTAAGCTCAG

>JAP04291.C|Supercontig_0000208:284589-284959

TCCAAAGGACTTATCCTGGGAACAAATAGTAACGAATGATGGCTTTAAAAAGCATTTTCTTAAAATATTTTGTGAAAAAGTACGTGAAGGAGATCACTTTAAAACTATTAGAATTAATGGTTCAACTACGGCAGGATCAGTTGTTAAAGAGTTAGTTGACACTTATTATTTAAAATATGGAACACGTGATGATTTTTGTTTATATGAAATTATTGGACATATTCAACCAGTTTCATCTCCTAATTTATCAGCTACACCAATTTGGTCTTTCACTGAACGTAATACTCGTCAAATAGATTTAAATGAAAATATTATTTCATTATTACAAAACACCATTCCAGGTTACGGATTATGCCGGAGATTAGAACTTA

>JAP04303.C|Supercontig_0000071:284684-285002

AGCTTAGAAATTGGGCAGATATACTACTAGTGGCTCCATTGAGTGCTAATACAATGGCAAAGATGGCATATGGACTAGCTGATAATCTACTTACAACACTTGTACGTGCATGGTGGTTTCCCAGTGAACAAAATATTGTCAATAAACCAGTATATTTTGCTCCTGCTATGAATACTTTAATGTGGCAACATCCTTTTACTCATGAACAAATTGAACGTCTAGTTGATAGACTTCATTGGAAATGTATAGATCCTGTTCAGAAGACATTGATTTGTGGAGAGACTGGTATTGGAGCGATGGCAGAAGTCAGTGATATTGT

>JAP04320.C|Supercontig_0000228:793997-794177

GTTTACCTGATGATTTTCGAGATTCTGAGTAGTTTTCACCAATTTTTCTGGATGTTGATACAAGACCGGTTTTTAAGCGTTCTTTCTGGTCACGTTCACGCCTTCTTGCCTGAGAATCGAAATATTCTTTGCTAACACCCCCACGATTGGAAGGGGTTTCGACGCGCCTTTCCCTATAAAA

>JAP04323.C|Supercontig_0000485:446189-447090

TTTCAAGTTTGCATTGATGGTCTAACACGTTTAGCAGTTAATAATGTTCGTTCATCAGCAAATCCAAGAGATCCTCTTTGAAGTTCAATAAAATCAGATGATTTCATTTTTTTACCATTAATAGGATCAATCATTTCTTTTTTTATTACAGTATCTACAACTTCTTTAGTAACAACTGCTCCAGAAGTTTTAAGAACAACACAAACTGTAGCATTTGTTAATGGATCTTTGGAAACAGCACAACAATATTTGACTTCACGATCTATTGGTTTATCTTGGCTAAAACCCATGACCTTACTGTCATCTTCAAAGGTAGTGAATTTAACACCAACTAAATCTTTATACCTTAATGGTTTGCCACTCATTGGACAGCGAACAACAGTATCAGGCTTATCTAAGAGTTCTTTATTCAGTTTCTCATTTGTTTTGCTACCAGATGCCCAAAAACATGAAGTTGTTTCATTCAAACCATTGCGTTTATTGGACAATGAGGCCAAAGCTTCTGCTTCAGCAGCTTGTGCAGAGGCTTTTGAGTGGGTATCTAGAGTATTCATTGATAGAAATCGTCGGGCTTCTTCCTCTTTTTCAGCCTTGAGAATCATTTTTGTCTCAGCATCTAAACGAGCTTTCTGTTTTTCATACAACTTTAGTTTCCGTTGAATTTCAGCTTTTTGAGAAACAATGTATTCCAGAACTACTGCTCGATCATAGAGGAATCCATCATTTGTAACGACAGGATCCTTTGCTGGTTGTAGACAAAGAGAGCAACAATCAAGAGCTTTCACTGAATCTTTTCCTAAACGGATTTCCTGGGTGCCAAAACCTGACCTGTGTGCATCATTTTTCCGCTCATAATAAGTATATACTGTATGAGCTGTGCAGTTTTTTGAGTGACGTGTCAT

>JAP04337.C|Supercontig_0000227:717243-717532

TATCTTCACAATGTTTAAAACTTCCTTTTACGAAATATTTGGCTTTACATGATGTTGACTTACTACCTGAAGATCCAGCATTAAAATATAATATGCCATCAGAATTGGGACCGATTCATCTTATTCCATTTTATCTTCATCCACGTTATTATTATTTTAAAGAATATGCCGGTGGTGTTTTAATCATTAAAAGAACTCAATACACTTTAGTTGGTGGTATGTCAAACTCATTTTGGGGATGGGGACGAGAAGATGATGAATTTCAGATACGTTTAAAATTGAAAGGATTT

>JAP04358.C|Supercontig_0000284:605429-605623

AGCAAAACATTGCGAATCTTCCATTGAGAGAACATCACATTCTACGGGTACTATTGTTAGAGGAAATTCAGATATCATAGCAAATTTATTGATCATCTTTTTTTCTTTTAAAAAACTTTTACAAGCAAGGGAAATCCTTGGAATGGCAATAATACTATATTGCTTTCCAGCACTTTGATTGGATTGTGAATCTCT

>JAP04382.C|Supercontig_0000155:341663-341888

AATAATCATTAGTTCCACCGAGATAAACTAATAGTTCATTTGTATGAATTAAACGCCCACGGACAAGCGCTTTGCGACTAAATGGAATATACGCATCTTTATACACCGACTTTTGCAACTCCTGAATTTTATTACTAACAGACTCATAATCTGATCTATATTTTTCCAAGCTGATAATCTCCTGTTCAGTAGTTTCAATATCAGAAGTTTGTCGAGAAAGTAACCG

>JAP04385.C|Supercontig_0000344:90087-90401

TTTTACCGACTTGTCTGCGGAAGCTGTACACATCCTTGAGTCATCAGCGGACACATCTAACCATCGAACTGCCGCAGTATGGGCACGAAAAGTGATAGACTCTCCTTTTCTAAATTTTATTATTTAAAACATCTTATTTTACATGCTCGGAATCCATAATTTTACTGTTTTATCCCTAGAAGCAGTTAGTATAAGTTCACCAGTCGGTGAGAATCGGACGCAGAATACAGCGTCCTTGTGGCCACTAAACTTATATGCTCGTGTTTGTGGCTTAATGTTCCAAATCATTAAACATGCGTCCATAGAGCAAGAAGC

>JAP04399.C|Supercontig_0000678:277377-277651

ATGCTTAACATTAACGTGGGAGTGCTGGGACACGTCGACTCAGGAAAAACGAGTCTAGCAAAAGTCTTAAGCACTATTGCTAGTACTAGCGCGTTTGATAAAAATCCCCAAAGTAAAAAACGAGGTATCACGCTTGATCTCGGATTTTCTTCTTTTACCGTGGATTCTGCGGGCTACCCTTTCACACCATCAATACGCGAAAACTTCGAAAAAGTTCAGTTTACTCTTGTTGACTGTCCCGGGCATGGGTCACTTATAAAAACGGTTTTGTGTGG

>JAP04407.C|Supercontig_0000385:527376-527554

ACTTCGATTATATCAGAATTTTCTTTAAGTCGGTATTTGCAGGCTGGGCACTGAAACGTCCCGTCAGAAGTTTTAAGTTGAAGAGGACTGTGAGTAATGCAGAAGATGTGCTTGCATACAGTGCCAACGGCACAGATTTTCAAGGGTTGTCTACATTTTCTGTAGTTGCACACAAGCAT

>JAP04421.C|Supercontig_0000000:1691838-1692019

CCAACAACTTCAAATTTCGCTTTCATATGTTGATTACCATATTTTTTCACATGTCCTTGTGTATCATAGTCTAAAAATTGTCGTTTTTTCCCATAGATATCATAGCCACCACCTTTACGGAATTTAGTCAATGATAAATAATGTACACCATAATCTTGATGTCCAACACCAGTCATTTTACC

>JAP04440.C|Supercontig_0000070:512503-512680

GAAACGAAAATAGGAAATTCAAAATTGTTTTGCTTCAGCATTTCGTGTTTTAACTGTAATAGTTTATCAATAGGACGACTAGCAGTGGGACGATTACAAGCTATCACTAAAACTGGAATAGTAACTTTATCAGGTGGAAAGTTTGCCTTCTTCAGTTTTGAATGAAGTTTCATTTGCA

>JAP04460.C|Supercontig_0000248:129242-129392

CCGCATAAAAAAATTAACACTGCCATAAGCCATGTTAAAACACCAACATGATATGTTGTTCTAGTCACAGTAACATGTTGACAACAATCACATCGAAAAGATTTTGGGGAACGACCAAGTTTTTTAGTAGGATCTATTTCTGATTCAGGAA

>JAP04477.C|Supercontig_0000644:221270-221371

GGAAAGGAAGTCAAGTCCGCGATGTCGTACCTGCTGCTCAAGCAAACATTCGGTGTCCACAAATTGTTATCAAATTCTACGAAAGCATTTTGCATTTTACTT

>JAP04488.C|Supercontig_0000046:1295413-1295581

CATTTCTTCTGCATTCTTCATTAATGCATGATGATATGCTTCAATTGTTTCCTTTGATGGTTCAGCAACAGAAATTACTGGTCTACTACATAAAATACCATGAATAAAACCTTTTGATAATAATAAAACAACTTCTTCAAAACTTAATCGGACAGGTAATCGATCTGGT

>JAP04500.C|Supercontig_0000319:87054-87665

TATATCCTATTCCACCACTGTGTGTACCTCCTTCTGGAAATTACTGCTGTTCACATACACCACCTACCCCGGTTAGACGACCATGGTTAGGCGATAACTCAGAACAAATAGCCACCGGAAGAAGTCGATCCGGAGGGCAAAAACGTTCAGTTTATGTAAATGCATCAATATATCCAATTCCTGAAATGATACGAAATAATGATAGTAATGGAAATAATTTTAGTGGCTATGTAAGAGATCGAGAAAATTCCACTGACATTGTTATCACATCTACAAGCTCGAATCCCGTGAATGCATCCACAACAGAAGTTATCACTACTATTAGTAATCATAATAATGACAAAACCACACCAAGTAATACATCGAGTGTACCAATCCCCTATTGGACACATTTATTACCGTATCGCACATCAACTGTACCTCAAAATTTATCTGGTCGCTTGAGAAAATCAGAAACACTTCCAAATGATTGGGTAAATCATGAACCGAATAAAATTGTAGATCATGCAATTCATTCTATACCAAAATCCACATCACATCAATTGAATATATTAAATCAAAATCATAACTCACAACGTTCAAATCATTCTGATGAGTGTAAATCACGTTCAT

>JAP04506.C|Supercontig_0000367:89700-89773

GGTTTTATATTAATCATGAAGAAAAGACTACTAGTTGGGATGATCCACGACCTGCATATTATTCTGCTCAACAA

>JAP04513.C|Supercontig_0000160:1385011-1385209

TGTATTCAGTAGCATTTGAGTGTGGTAAACCGGCCATTTATTTAAATAATGAGTTTTGGGTGACGGATCTTACCCAGGGTTGTCTTGAAGATGAATTGTCGATCCTGAATTATTGTAAGAAAGTATACAGCGAGAGGAACATAACCGCAGTTGTCTCCGCTCCTCCTATTGATGTTGTACTGTCAGATTGGTGTGAGTT

>JAP04517.C|Supercontig_0000000:3869656-3869762

CGAAATAGTCGATGTTGAGATGATTTTGACCGTTCAATAGTTATGGAACCACATTCAACAATCAATAATGCCTTCATGATTAAATCCCCAAATATATTATGTAATCT

>JAP04552.C|Supercontig_0000635:81949-82137

AACCAAACGTTCTTTCATATTTAACGACTTATGATCATTGACAAACTGACGTAAAGAACTTTCAGCTAATTCACACCCAATAATTTGACGACCTATTTTGATAAAGCCATTCCGTGTCAACAGATTTCCACAGAGTGGAATAAATAACCTGTTACCTGGATTATCATTCAGAAATAACTCATTCCAAAA

>JAP04570.C|Supercontig_0000131:52542-52826

TAACGTACTGAATACATAGGATTGTTTGAAATACGGTTCGAAAGGTTGTGATAAAAGTCTTGCACAATCATTGAGGCTTGATTAATGTTTGAACTTCGAATAGGCTCCAACTGTTCCATAAGCTTTGAAACCTTGAAAAAATTTCAGTTACTAAATTCGAATATCTACATGGCGGGAAATATCGGCATTTGCAGAGGCTCGAAGGGTATTTAGAAATGTTTTAAACTCTTTTTCGGCTTGTAAACACTCTTCTGGGTTCACTGGAATTTGGTCCCGGTTATCATC

>JAP04594.C|Supercontig_0002053:390848-390950

TCCAGGGACAGCTTCTACTGCTGCTGCTGTAAATGCAGGTCATCTGGCTGCTATGGCATTGCTTGCTCAAACTCTTGTCCAATTAAAAAATGCGACTCAGTCG

>JAP04607.C|Supercontig_0000642:77963-78075

GCTTTTTGTATCAATATCTTGCCAATACGCATAGATCTACAACAATCTCTTAGGCCACGTTCCATTGCTTCACCAGATCGCATAACAGATACACCACAATTTCCACGAAGAAA

>JAP04649.C|Supercontig_0000682:251463-251743

TTGATTTTTTTCATACGTTTCTGTGTTTTATTCTTACCGGAACCCTTTCCATGAAATTTATGACTTAATTGACGAAAAGCTTCTTTAGCACTTAAATCACGTCCTAGCTCATCAACATATTCTAATTTAATATCCGGTTTATATTGCGTTAACTCTTTAAAATCTGATAATGGACCAGAGTAACGATCGCGTTTGTAAAATTTCGCATCAATATCATCATAGCGTATATCTTCTTGGACAAAGTGTTTAGCCATTAAATTGACCATAGTTCCTGAACCAGT

>JAP04688.C|Supercontig_0000050:28065-28236

AATTTCATCTGAGGACTTTGAACTTTCATCATCTGATTCACTAGGTTCACGAAACCATTGTTTATTAGGATCATTAAGGGATGGATCATTTTTACATAATGCTTGATATAAAGTTAAAAAATCATCATGTTGTTTTTCTTCCCAACTATCATCAGTGTCTGAGGAACTTGAA

>JAP04713.C|Supercontig_0000019:1038841-1039112

TCAATTATGAATAATGCATTTCTTAGACTTTTTTCATATTGTTCAAAATTAATTGCTAATGGATTGAATAAACGAATCTCAGGGGATCTTGTAAACTTCTTTTTATTCACATATGCTATTGGTAGGGTTTGCTTTAATACTTCAGACACAACATGATGTCTTAATGCTGGTATATATGTTGGACGTAGACGGACTGTAAATGATTTTAGGAGATCAGTTAGACGAAGCTTTTTTGCAGCTAAACCATTATGTGATAACCAGTTAGGAATTGT

>JAP04716.C|Supercontig_0000032:790723-790897

ACAGTAGACTTTCATAGCTGGTCTAGTTTCTATAAATACACCCTCTTCAGTTGGAGTGGGCGGATTTGATTGAAACGCTTCAGGAATGTAAAAACCCATTGTAAAACATCTTTTCATGACAGACTGATTATCTGGTTTGCTTTCAATAGTAACTGGAGCAGTCATCGGTACTTTA

>JAP04722.C|Supercontig_0000131:52860-53116

CGAGGAACCTTAAATATATTTTTGATAGACGTTGCATTCTTTCCAATGGTATGTTTGGGTTTAACCTCCGCTTCTGAATTTTGAGTTGAAAGTGATGAAGTGGAAGAAGCCTGACGAAAATTCGATTGCCTTTGATGAGCGTTGCGATAGCAGACGGAACAATATCCATCCCATGAAGGATTCCCATAAAAGCCACACTTATTTTTACATGACAAACTTGATACTTGAAGACGAGATGGAGGGTAGTTTGCATTCAT

>JAP04724.C|Supercontig_0001921:17899-18125

TTACTGATAATCGCCCCTTTAAAACTGCCAGTTCAAACGTCGATGCAAATCCTTGATCAACACCAATATCAAATTCTTTTTGTAAAACAACGTCACTTTTAGCAAAATATCCTTCTCGTATGCCTGCACTATAAACTTCCTCAGAACGACGTTCCCAGTCTCGTGACCCAACTTCTAACTCTGAATGGACATCAGATATTTCGCTATTGAATACATCATCCATAGAA

>JAP04729.C|Supercontig_0000598:60091-60243

ATATGATGAAGTGCTAATAAATGGTTTGCCCAGTTGGCGAACCCCAGTATTTTACTTTAGGAAGCTCCGCAAAATGTCTAACTACGAACTATTTGGATTGTTCTTCGGAATGGCTACCGTAATTCATTATGCTGCACTTTGGGGCTCAGTTTT

>JAP04749.C|Supercontig_0000225:305004-305176

ACTTAACGAATTTAATTGACCAATCATCCGTTTTGGAAAAGTCAAAGAAAAATTTAATTTTACTCTATTATTCATCAACATGTATATACTCATTTGGTGGAAATAGTGCTTTGTGGCAGTTTGAAGCAACAGCTCGGTTTTTCAAATCAAACCAATATCTTTTGTTTGCTAGG

>JAP04798.C|Supercontig_0000042:1723654-1723763

TGTCAAATACTCTGGTGGTGTATCCGATGAGAAGGTTCCATCAGGAAACGAAATACGGCAGTATAAAGGAGTTACTCGTCGGATAGCAATAGCGGATGAAAATATAGGAT

>JAP04844.C|Supercontig_0000447:235926-236182

GAAAATTTTACCGTAGAATTCAAGCCTGATATGCGACATACAATTGTTCGGTATAATGGTCAAGTTTACCAAGGTCAGATTATGGATCTCCCATGCATAATAGAATCGTTGAAGACTACAGATAAAAAGAATTTCTACAAAACAGCCAATATCTGTCAAATGATGATCTGTACCCAAGGTGATGAAACTGGTCCACTACGTGGGACTGCTGCATATTTAGACAATCGTCCTGGTTCACGCAATAAAACGTTTGATAT

>JAP04850.C|Supercontig_0000225:674864-675111

GGTATGGTTACAATCTTTTGTAATCGTCTTGGCTGGAACCATATGGAACGAGTAATAGCTAATTTTCAATCACGTCTATGCTATGGTGTTTCGGATGAACTTGTCGACTTAATACGTCTATTACCTTTGGTTAACGCCGAACGTGCTCGTGCGCTATATGCAGCTGGTTACAGTTCTGTTTCTTCATTGGCTTCTGCACGGCCTCAAGAGATTTCAAGAATTTTACAACGTGCAATTCCTTTTGAACG

>JAP04875.C|Supercontig_0000673:234668-234885

AACAGTAGTAAAGATGGCAGTTTTTACCATTTTCACTGCTTCGCCTGCTTTATTGATATATAATCGATCTTTCATACTAAATAATGATTGATTGTTTAATTTTAACATATCGCCTTCGTCATGTGGACAGTAGTGAAATGTAATACATTCCGGAAAGCGACGATTTGACGTGATTTTCACCACCATAGATAAAGGAATTGATCGATAAACAACAGCAT

>JAP04878.C|Supercontig_0000201:70693-70841

GAATTCATCATCAGTTATTATGAACATTCTTGTGCACAATCTCGTCATCGTTTTCTAAAATCCATACTCAATTCGTTTCCATTGAATTATTCCATAATGGAGAGATGGTATCCACCATCTAATGCGAAGGAGAAAAAAAGTGATTTTGA

>JAP04885.C|Supercontig_0000195:308780-309036

CCCCATAATTCTGCACCACATTTATCACGTACACATGTAGCAAAACTTAATGTTGATGGTTGTGAATGATCACCTTGACCACGTAAACAACAACTATTCATAACTTTCATAATTTGTTTTTTATTTTTTTGAAAATTTCTCCATAATCCACGTATTGCTTTAGAACATTCTTCATATTGACTACCACATTCTTGAACACATAGATCAAATTCAGGTTTACTCAATTTTTCACCATGTGAATCAGCATAACAAGAATT

>JAP04906.C|Supercontig_0000012:1612757-1613065

ATTATTATTATCAAATATATTTTGATTAACAGACGTGGCTATTGATGTAGTAAGAGATATAGATGGATAAATAAAACCACGAAATGGTAATATAATAAAAGATAATAGTATACCTGATAGAAAACTACCAGCATTTGCATAATTATCAATCCATGGTAATAAACCAAATGTGAATAATATAAATACTAATAAAATATTTCTTAGTAATGCATACCATGGATGTGCTAAAAATTGCCAACAATGGATTAAATCAACTAATGAGATACCGAGTAAAGCGAATTGTGCACCTGTAGGTCCAGTTTCTACCTA

>JAP04907.C|Supercontig_0010613:1089-1197

ATTTTTTGGCTTTGATTCTCGTGAAACTACTATTGAAGATTGGAATGAAAGACGGTTGCGATATTTAATAAAACGTTATGGAAGCATTAAAGGTGATCGCTTAACTGTT

>JAP04921.C|Supercontig_0000346:57905-57981

CCTGGTTTACTACATAATAAAGGAAGACCAATAATTAAAGCCAATGGTCCAATATGTCCAATAAAGCGCATTTGTCT

>JAP04951.C|Supercontig_0000381:445451-445701

AAGATTTAAATGCACTTTATTGCTCACAATATTTGAGACTTGGTGACACTTCTCAGGCTGCAAATCTAGGCTCAACACTGCGACCAACTCCAAATGAAATGTCCATCGCACATAGACTAACTGAAAAACTGATTAAACTGACGAAACATGCTCGTCCAGGGGATCTCATCCATCCATATACTGTAAGAAAAGCGATGGGTTTGAATCCAAATGAGTATTTTTTACCGGAACATTTAATTTCAAGTGATAAT

>JAP05004.C|Supercontig_0000001:669782-669863

ATTTAGTTCATCAAGAAATTACAGAACTTGAAATGAGAGCACGAGCTATTCGTGCAATGCTTAAAACTAAATCGGATCAACT

>JAP05023.C|Supercontig_0000035:199842-200276

CCCTTGAACAAACATTGAAACATAGTATACCTGGTTGTTTAATATTAATGGATTTATTTTTAAACAGTATTCCATTATATATATGTCATGCATTCTATCCTGTTATTGTACATCTAATCTATTTGGCTGTTGTAACATTAAGCCTATATTTAGCATATACAATTGGTCATCTGGATAAAACTATACCGTTTCCTTCAAATCCAACTGTACTTATTGGTGGACATGCATTGTTACCAAATGGTTATCAAGATTTTTCAAATATATCACGTATATTCATCATTTTATTAGGAGCTATCTTTATTGGTATTATGATTCATTGTATTCTATTAACACTAGTGATCACTAGAGATTTTCTTGCAAGTTTATGTCATCAAAGTACCAATATATGGTATGATTCTAATGATGATATAGCATATTTAATGGATAACAGTACAA

>JAP05071.C|Supercontig_0000050:518766-518960

TTGGTGGTATATCAGGTTTCTTGCACACAAGTCTAGCTGAAGTACAAGCTGTTGTTGGAGCCCATACAACTAGCCTAGGTGGAAATGCACTGTTATCATATCACTTAAGTGAAGTTGTTGTTCTTCGACCAACTTCAAGAAATCAAGCTCAGTGTTTATTAAATGTTTGCGGAGATATGGCTAGAATAAGTTGGG

>JAP05085.C|Supercontig_0000345:217689-217851

AGTTTGTTCGAATTTCGGATGAAGATAGACTTGAAGATGTAATGATACTAATGAAAGATTATTGGAAAATGATGGAACCGGAAAAACCAAGTCTGTGCATATCTGTTATTGGTGGTGCAAAGAGTTTTGTTCTTGATGGACATAAAAAAGATGTTTTCTATTC

>JAP05093.C|Supercontig_0000019:1163221-1163452

CATCAAGTTTAACAAGACTTTTGCGTTGCACTGTCATAGAAGGTGATTTGTTTGTCGTTTTCACGCGAATACCACGGGATGCAAGCCTGCCATTTCTGAGGGAAATAACTGGAAGTCTGCTTGTGTACGATGTTGAAGGACCAGATGATCTATCCAATCTATTGCCAAACCTTACTTTGATCAGAGGACAAACACTGGTGTTCGGTTATGCCGTTGTCATTAAAAGTACATC

>JAP05105.C|Supercontig_0000265:79323-79424

ATTTTATAGCTTCCAGTTCTACAGGAAGATTTGAAGTGGTATCTGTAGAATGTCAATCAGAATCTACATATGATCCAGAACAAGATGATAAGGGCGAAAACA

>JAP05122.C|Supercontig_0000002:143852-144091

AACAATGACAATTGAATTACCTGAAGTTACTGGTGGTCCAACATGGATACGTAAAATGCGTACATTATTTCGTCGTCTTGATACATCAGGACATGGTTATTTAATGGTTGATGATATATTAGAAATTGGTACATCATTATTTAATATTTATCCTAAAATGTTATCATATAAATATGATGAATTAGTAAAAACATTAGTTTATTTATGGTATGATGTAATATGTATCCATGTACCAAGACA

>JAP05128.C|Supercontig_0000046:1902021-1902228

ATCAAAGATATTTAAGTCAACAATCATTAAATCCATATCAACATGAATTATACAAATATCCTAATAGTATTACATATAAACGTGGTCCATCTCCATCAGCATCATCATTTAGAAATTCACCATCATTTTCATTTAATGGTGATATGCGTTCAATTTCTAATTTACGACATATGATGTCTGGTGCTAATCCATTAGCAGCATTTGGGTA

>JAP05129.C|Supercontig_0000093:82542-83001

TGTTGTGATTTGTGTCCACCCATCATCCAAAAATGTAGTGAATTCTTTATAGCTACACTTAAAGCAAAACATATCATACCAATTATCACAGAATTTAATGTTACTAAATTAGATGCTTTGTAAGGTAATGTAACAACAAGTAATCGTTCCACTGCAACAGCACATAATACACCAACAGCAAAATCTGTTGAAAATCCTACAGCAAATACATGCATTTTACAAGTTAGCGGGCCAGTTGAGCGTAAATCCCATCCACAAACTTCCATTAACCAATAACGTAATACGGCAGTTAATAATACCAAATTATCAGCTATGGCTAGAATAATAATTATTAAACGAGTTGTCAGACGATTTGACTTATCTCCTCCCATTATAATAGCACTAATAAGATTACCAGGTAGACCAAATAATAGTAATAAACTTGCATAAACTTTTATAATCAATTTGCCTATATCTGGAG

>JAP05136.C|Supercontig_0000187:836901-837006

TCATTAAAACCAAACATGGCAACATTACGTATAATCACTGAATCTGAATTAATTCGTGGTCCATTGATTGGTTCTGGTGCATTTGGTACAGTTTATTGTGGTGTTT

>JAP05140.C|Supercontig_0000123:1122586-1122751

TTTTTATTTCCACGCATCATACCAGTACTGTATAGATTATGCCCAGCAGTCCCATTCATATTATAACTTGGTCGCATAGGTCTATTATTAAGGGCTGAATGATATGGATGATGTTGCATAGAATGTGGATTATTATACTGCATCATATTTGGATTATTCTGATTGA

>JAP05161.C|Supercontig_0000176:290339-290523

ATGGACGAAATAATGGGCATGATTCACAACCAACAGATGTAATAAATAATAACAATAATGGAAAATGTAAAGATGTAGCTAGAGGTGGTGACTTTCCTGAATCATCTGACAGTGATCATAAATATTCTGGACGTTGTTCCGATAGTTTATTAGAATTGCCAGGAAGTCAATTTGCGTTTGCAGGT

>JAP05170.C|Supercontig_0000228:347988-348142

AGGTTTTCAATTCTTTCGACCTAAAACTTCTGTACAACCTGGTGCAAAACTAGTACAAGTAAAAGAACTTAAACGTATGATACGTGCAGGTAAACCAAGTTATGAACAAATACATAACTGGGGACATTCATTTGAAGCTTTATTAAATGATAAAT

>JAP05172.C|Supercontig_0000155:1394255-1394498

AAATCCTAAACGTCGACCATCTACAAAGAAGTTGGATGTATACACTTCTCTAGAGATATTATCTCGACTGACAACAGAAAGAACACGTAGATCTGATTGAAATCTTAACAACTGAACAGATTTTAATACATCAGCTGCTAAAACTAAATTTTTTACACATAATAAATTATGGATGTATAGTTCGGAGTCAACAAATGCTACACCCACAAGATCAGTATTTTTCAATGTCCAAATATAGATCTAA

>JAP05190.C|Supercontig_0000156:1291091-1291304

GTCGTTTGCCCTCCTGAAAAAGGCCTTCGGTATCGGGAAAGCAATCGACGACCGAGAGTTTGACGAGTTTCTTAAGGCAGTTGACGTCAATGATGTGCATGTCGGGTTTGTAGTTCAGTTTGCTCTGACTAACATTTAAGGCAATTGAGTAAATTATTAAGAAAAAAAGCAGTCCGGTTGTTTGTCCTAAAAAATGAAGACTCAGGTGTTATAT

>JAP05197.C|Supercontig_0002048:272963-273264

ATCTACGGTGAAGATGATGATTCAGGTCATCATATCGAAGTCCGTGTATTAAATCGAGATATCGATGAAATGCCGACCTTACCATCAGAATTGAAAAATAAACCTGTATTTGAAATAAAACTTGGAATGCTTAAACAAGCGACAGTTTATCGGACAGAATTTACAATACCGGATAATTTGAAGTCAGGTGAAGTGGAAATTCTACGAACTGTTACAGAAATGGATGGTCACGTTGGTGTACCAGTTAATGTCGGTGCTTCATTTAATTTACTAAGCTGTGAACCTGTCCCATCACCTGGTCA

>JAP05198.C|Supercontig_0000104:183021-183151

TAATAACCTCGGAAATTATCATCATTGTAATAGTTATATAGATCATGATTATACCATGGAAATGCTTCACGATTCATTGAACGCTTACCCAAACGTGTATCCCATAAATAATTACGTTTCATCATATGACG

>JAP05245.C|Supercontig_0000255:47655-47818

TTTGGATTTCTGAATTGTAAAGGAGGAAGATGCCATTTAATGAATTCATCACATCCGTGTGATATTTCTGCTCCGGGGACATGATTGATGACAATAATCTTGACACCATCGTTCAGAAGTATTCGTCCAGCTTCTAAGTACTTTTTAGTCCTTGTCACCGCAGC

>JAP05289.C|Supercontig_0000058:1560789-1560897

TAAAATGATTATTTACTGAGATCCAAAATAGCTTGACTCTCTTCAAACTCCTTAGTATTATCGTATTTATCCTGAGATTGATCTGCATGTCTACAAGATACAGTTTGTC

>JAP05292.C|Supercontig_0000225:413810-414023

CATCAGAAATACCATTTGTTGTTTGTGATCGTGGCAATCCTTGAAATCGTACTCTATGAGTTGATGCATCAAATGTTTGCTTGTTATTTGGCATAGAAGATTGTTGTACTGGGTTTGAATTTGTAATAAGTCGGCTACTTGAAGAACTACGTCCGGATACGTTCGAACTGGTACAAAGTGACTGACAATATACACCTTCATCTATTAGAAAGCA

>JAP05305.C|Supercontig_0000130:2152941-2153066

TTGAAATTAATCGATGAATCTTCACCTTTTCCAAAACATCTTGAAGGTACAACTCTATTTCAATGCCCAACATGTATAAAAGCTTTTATTACTAAAGAAGGTTGGACTTCTCATATACGTGAATGT

>JAP05312.C|Supercontig_0000289:167010-167187

TGAATCATTGAATTGTACAAAAAAATCGGAACATATTTTACCTGGTTGTCAAACAGCTGGTCAATTTGAACATCAAGTAAATCAGTTTTTACGATCTTTATGGAATAAATACTTGGGTAATGAAGAGGTAGATGCATTAATTTCACGTTTAACTACAAATATTATTATATTTCATACA

>JAP05320.C|Supercontig_0000005:1730834-1731066

AGGTCTGGAGTACCAATAGCTGGACAACCTCATTTTATCTATTCACATTATTTAAAATGTATCAATGGTTATCCACGTTCATGTCGAGCATTTCATGCTATTGATTTGGCGTTAAAATCACATCAGTATTTGGGCGATGTGATTTGTACACGTAGTAGAACATCTGCGGCTGGTGTTCCAGCTGATTATGCATTAAGACATGGTGTTTATTGTACTGTATTATATACAATATT

>JAP05326.C|Supercontig_0000130:103704-103807

CGTCTTTTCGTTCCAGTTATGGTGGATGAACTTCACGTTCTTAAGAAGAGGATTAGAACACTCAGTGAATCTTCCAGGTATGATTTTAACAAACGTCTGTACTC

>JAP05333.C|Supercontig_0000357:15239-15655

TCAGTCGGCCAGCGGTTCTGTCTTTTGATGTTCATTCAGTTGCGATTTGAGGACTCAAAAAGTCAAAGTGGATAAAACCAACAGGATGGATTTCGTGAGTATTGTCTATTCAGCCATCTCTCTTCAGTGTTCGTTCGACCTGCGTTCGATCTCAATCAAAATGACGGTTGTTACAGGTTTGTAAATTGGCCGTATCCTGCACCCATTTTGTCAGGGATATTACTGTAAATTTTTTGTTTAGTCTTTTGCTAAACAACTCAAGTATTGCTCATCAGGAATACTGTCCTATTCTGACTTCTTGCTGCATACTTCTCAAATGCATTCATACAGTGATTACTAATGCATTTATTGTTCTAAACATTTTATTTTCAGTTTGCTTGGTTGGACAGACTATCGACGATAATCTAAATAATTGCA

>JAP05341.C|Supercontig_0000665:125802-125900

TTTCAGTTTTCCTGTAATTAGTTCAGTTAGTGAAGTTATCAGTACTCAAGCTGTTATCCCAACATCTATTGCCGTTACTTCTATAGTAAGTCTACCTGG

>JAP05380.C|Supercontig_0000156:958864-959361

AAAGGACTTATAAATAATCCCACCAGACGCAACGAAATTTTTGGCATATGATAAAGTTACTGTTCTCCAATCAACTATACCTGTTTCAGGAGAATGAATTGCTTTCAAGCCAACACAGTTAGGCTCGATTTCTCTTATCTTGTCACCTGAAATGTAAGATACATTGGGTACACCGTTTTGCTGGGCAAAACTGTACAGCTTTTCTAGTTGAGGAAGCTCTAGTTCATCAACTGCAACGATTAATTTTCCACATTTCTTGTAAGGTACATTGTTTGCTTCAAAGTATTCATATGACTTCTTCAAACCTTCAACGCATAGCTTAGCTTTTAGAGAGCCAGGAGTGTAGTAAACACCTGCATGAATAACACCACTGTTATGACCAGATTGATGCATCCCTAATTCTTCTTCTTTTTCCAGAAGTGCAAACTGAAATTTCGGAAACCGAAGTGCTAGTTCACGAGCCGTTGCTAAACCCACAATTCCACCTCCAACAATCGC

>JAP05398.C|Supercontig_0000227:350781-350941

AGCTTCTTATGACTCTTAGTGAGCCTTCATTCAAAACCAAAGATGAAGCTAAGGACTTCTTGAAGTGTTTATGTGATCAAGAACTTACTGTCGATGTATCAGATGGTCGAAGATATATTGGATTCTTATGTTGCACAGATAATGTTGGAAATATTGTAATG

>JAP05403.C|Supercontig_0000009:1228928-1229132

ACTTCTAAGTCCACGAATAATAGGATTAGCCATATTACCTAATCCATAATCAACAATTGCACCAGCCAAAATCAAGTCATTTAACCGTCCAGGTGTAGCAACAAGAATAGTGGAACCTTTTTCTTTAAAGTTATTGAAATCTTGAAATTTTGTTGTAGCTCCACTAGAACGTCCACTGCTAGTAAAGACAAGCGCTGAGAAATTA

>JAP05419.C|Supercontig_0000366:133077-133240

AGAGTGTGACAAGTATTGCTCGCCAGCAAATCAGTTATACCAACAACCCCGTTTGGACCGAAAGCGTTGTCACTGAGATCCAATTCAACTATTTGAGACTTGGAAACCATAAGACCATTACTCAAATGCTTTAGTGCTGGTGCTATTTCAGATTTAAGGCGACC

>JAP05439.C|Supercontig_0000107:900201-900328

AGACCAGATGGTAAACCAGATTATTTAGGTTTATTGGTCTTGGATGAACCATGTGCTAATCAGTCAGATCCAACAGTACTTAATTTACAATTGAGAGCTTTGTCAAAACAAACTGCAACAAAACAAGT

>JAP05444.C|Supercontig_0000491:515703-515930

TCTTTTTATCATATAAATCAATTGGATTTTTTTCATTCATTGATTGTTTAAATTCATTATGCTTTTTTTCTAATTCATTTCGATAATCTAATCGACGTTTTTCAAATTCTTCTGATAAATTTGGTAGACCAGAACCAAATACCCATGGATGAGCTAATGTTTCACGAGCTGTTAATCTTGATTTTGGTGTAATCACTAAAAGTGCAGTGATTAAATCTTTGGCATCTT

>JAP05447.C|Supercontig_0001787:538-665

AAACATTGAATTCACTGTAAGATCTCTTCTTTCAGCATCTAACTTCCAGTTATTTGTAAAGACCACTTCACTATGCCTTCCATCTGTGACAACGTCAACACGTAGAGTGGTAACTTCAAAATTTTCCT

>JAP05455.C|Supercontig_0000114:267796-267903

CTTTATGTACATTTAAAGTGATAGTACGTACAGCAATACGAACCATACCCTCTGGGTGAGCGAACAATTTAATCGCTTCAACATATAAATCAAACTCTCGACGAGACT

>JAP05461.C|Supercontig_0000672:411718-412105

TAGAGGATCCACGTATTACATTTTGGCGTGCAAGAATAGGTCATTTTCAATCAGGTGAAGTAAACTTAGGTAGAATAGTTACACCAAATCAATTAATTGCAGAATCAAAAGCAAAAGCTGCACAATTTGTTAATACACATGTACAAACTGGTCCCGAATTAATTAAAACATTACCACCACCAAAAGGTGCATTACTTCATCGTGCTATTCAAGTTGATTTTCTAAATGCTAATTTAGATGATCGAGGCACACAAACAGAATCACCAACTGTAACAAATATATTTAATTTTAAAATTGATGCTGGTACACAATATGTAAGTTCGGATGTAAATCCACCAGTTAGTATTTATCGCTCAATTAAACAAATTTTAGTACAAAAATGATATTC

>JAP05465.C|Supercontig_0010602:14361-14649

CTTTGTCAGGTGTTGTACTACTAATCCACATTCGTATGTTCCAATCCAATCGTGAGAGTCTACAAAAACTCTAGGCTTGTCTCCGATTTCATGCAATATATTTTGAACTTCTAGTAAAGTAGGTACATCGAAAGGGCAGGATTTCGTAAGTTTGTACCAAGACAAAATGGTTTGCAGTGTTCTGTAACCACAGCCCCAGCCCTTAAAATAATTTGCTTTTAAAACTTACTTGGTCATTAACACCTTGAGCACCATAATGATAATAGTAGCATGAACCCTTAAAACCGAC

>JAP05467.C|Supercontig_0000000:2941284-2941437

TGCAGCCACGCTATTTAAGTCAAGCACATGAGTCAATTGGACGTCGTTTAACATCACCTCCTACACGAAGTATGTGGTCAGCCGTTGGTGGTGCTGGTGTCCGTACAAAGCCCAGCTCAGACAATTTCGGTGGAATAAAACACAGTACACCAAA

>JAP05472.C|Supercontig_0000622:64739-64833

ACATACGAATAGCCGAAAAGATGTCCACAACGAAGACAGCAGACTTTGTGTGGTCCTGACGAAGTCCATAATTCAAAACATATAGGACATGTCTA

>JAP05473.C|Supercontig_0000024:1031466-1031610

TAAAATACTGCGACTGCGCCTATAAGTCCGTGCCATGTGGCGAAATGCGGTTTTCCGTTTAATATTTTGTTGGCGTAAATTCCAATAAAACCAATGAAAATAAATAATACTGAAAGAGACTCGAGTATCCAGTGGGCTTTTATCT

>JAP05477.C|Supercontig_0000082:111588-111729

CAACCATTTGTGCAACATCGCGAAAAATTTCGTTCAATTCATGAATAGACTGAACAATCTGGTGAATTTCTTGTTCACGTTGTACAACCATATTTGTATTTTCTGTAAGTAATAAACTCCGTCTTTGTTTTTGTGATTCCCA

>JAP05505.C|Supercontig_0000331:153546-153667

TTCCCCCAAACAACAGAAACGTAAACCTGTGGTAGAGGAGCTGATAGTTCTTTATACATTTGTCTAGAGAGAAACACTATACACAACAAATGAGCTTCGCAATGCAACAAGTAAGTATTGTC

>JAP05526.C|Supercontig_0000380:304357-304673

TCAGATTCTACGAAATTCAGTTTATCCATAATCCCAGCAAACCAACCAGTACTTATTGGTCAAAAAGTAATTTTAAGATGTCAGATACAAGGAATACGACTTTCAACAAAATATGCAGTGTTATTTCGGCATAAAGAAACTGTAATTTCAGAGAAGTGTTTCGTGTACCAAGCACAAAAGTTTCATCTAATATGTGAACCAAATGATTGGCAAATAGCTGAAACTTATGAATTGCATATTAAATCGGTTTCTTGGGCAGATCGTGGTGATTGGTCGTGTATTTATGCAGCAAATAAAACCAAACAAACTCTTGAAGT

>JAP05577.C|Supercontig_0000026:1718336-1718606

TCTAACCATAAGATATAGTTCAAACGCAGGGGAACTGTTGGTATCAAACGATCGCCTGGGAAATCTACGTCTAAACCAAAGTCATTCATCAACAAAGCTTTGGACAAAGCAGATAAGTGAGATGGTATTCTAAAGTCAAGGACAACCCGGCCACATTCATCTTTTATGGCCACTGCATCGAAAAAGTCGAACTTTGCAGCCAATTCTTTGAAATTAGGTTTTTTCTGTTTGTAAATATTTCGTTGGTGCATATATTTATTTAAAGCCATGA

>JAP05603.C|Supercontig_0000019:1772821-1772961

TTATATCAGAATATATTATGCTATAAAAAAGACTAGAGGCAAACACCGCCGTATATGCAAACAAGTGGAAGAATCAACATGGGTGGGAAGGGATAGTTATAGCAGAAGCATCTAATTATATCCAATAAGTAACAATTAACA

>JAP05608.C|Supercontig_0000344:261377-261539

GATTTACATAAAGGTGATGTAATTGATGTCTATGAATTATCACATCGTTTATCTCAATCAGTATCTTGTGCACCTCCAATTATAATCCATCGGACAAGTTGGAACAATCAATATGGGTTAGTATGTAAAGGACCTGCACTTTTAGCCCTAGATGATGATTATG

>JAP05621.C|Supercontig_0000380:315125-315239

TGGTTCAGCATTAGGTTTAATTTTAGTATTTATTGGTTGTATCATTTTGCGTAATAATGGGAAGTCGTCACCTTTACCATTTCATGGTCATATACCCGGTCAACGTGTATAAAAA

>JAP05625.C|Supercontig_0000088:137793-137955

TAATCGAAAATTAAGTTATACAACAAACGATTTACCAAAAAATAATGCTTGTTTATTGCTAGATTACTCAGAAGAAGATGAAGATTACCAACGTTTGTCTGATTTTTCTTCCATATTGGCTGATTTAAATCTTGCGCATGAAAGACCTTTGACTAGTTGTTAG

>JAP05663.C|Supercontig_0000064:660146-660324

GATAAACTGGATAGGTTTACTCATGAAGCAGGACAAACTTCAAATAAAGAAGTACAACGATTAGTTGCAAGTATATTTTATTGTTTAAACCTAGTCGCTTTACAGTATATTTCACCTTGTTTTCTTTTCTTATGTCTTTTATGCTTATATAAAAATCTATCAGGTCTATCATGGTTACC

>JAP05681.C|Supercontig_0011744:182-315

AGTTGTCATTTAACGCCATGGTTCATATAATCGTATATCACCTTCTAATTGGGCAATTTGTAAATCTTTTTCGGCTAATTGTTTACGAAGACGTTGAATTTCTCTTCTTTGTTCAATAAATGCTTCACGTAACT

>JAP05689.C|Supercontig_0000045:374021-374343

ATAAATTTCTTCCAGTTCCGACAATCAAACCACTTGTATTATTGTTACTACCCCCGCAACTATGTACAGAGTCTGAAATTACTGTACCACCTCCATTTTTAACTAATGATCCTGAATGTGACGATGTCCTGTGTTGTGTTTTATTATTTAATCTAATCGTATGACAATAATCGTGCTGGTTATCGAGTGAAAAATGTGAACTATGACAGTCATGAGATGCATTGATTAATTGTGAGTGTGCACTCTGTTCAGTCATCTTTTTGTTATAACTGTGATGGTGGTACCGATGAGAGTGAGTATGCGGCGGTAACTGATGTGAATGT

>JAP05694.C|Supercontig_0000019:1688371-1688464

TATTCTGTAAGCTTCTGTTTCAGGATCAGGAACACCAGCATTTTTCAACTGAGCTACAAATTGTGTAGCAAACGGTGAATACAATAAAATATTC

>JAP05698.C|Supercontig_0000138:134674-134852

GAACAAAATGGCATATCAAAGTGTATTCATGTGCTCAATTAACTATTCTAAATCGTTGCTCACCTGAATCGACTTCTCATTTTCGAAATATAATGTGGCAATTTATTTTTGATACAACACCTTATGAAAAAGCAGCAAACTATTTATGTCAACAATATAATCTACGTAGTACGTGAGTT

>JAP05724.C|Supercontig_0000002:159686-159825

GATACTGATAAAGATGGTAAAATAAGTAGTGATGAATTTCTTACAGCATGGAATGATTATTTTCTTAGTGAAGATCCACAAAGTCCATATAGAATGTTTTTTGGTCCAGTTATTTCACGACCTACAGAAGCTAGATAATT

>JAP05739.C|Supercontig_0000096:304101-304290

AGGTCAATGATACGCCAGTAGATAGTTTAACCTGTAGTGAAGTAGTAAATCTTATTAGTCGTTCAGAAAAACGATTAGATTTACTAGTACAACGTCGTATTACAAAGCATATACAACGAAGTAAACATTCACATCAGAATGATTCAAATGATCTACCGAATAATTCTTTATTAACTGGACTAGGAGGGGG

>JAP05747.C|Supercontig_0000091:14758-15062

ATTACCAGTAGATATAAGCAATTGTACCGAAACTTATAACGGCAACTACAAGGATGCTGCTAATTACAGCACCAGCTGTACTCTTTACATAGCCGCTTTCTGCATAAAATCGGCGAAGATATTGAAGCACTTGAGTTGGAAGCCATCTTTTGCGTGTCATATCTGTTTCACGCATTCGCATACGCATGACTTCGATTTGTGCACGCATTCTGGCTTGCCGAGCTAGACGAGCAGTACGGCGTTCATGTAAATCTATCGAAGCAGCTAAATATTCTTTGGTATATGAATCGGAATTATCTTCGACT

>JAP05766.C|Supercontig_0000130:1702483-1702646

TGATGACTATGAAAACGTATAAATGTCATAATGAATCCAATTATTATACCCAATAGTATAGGACCAAATGCTATTAACCAACCGAGTAGAATAAAATTATTCTGAATTGGATCATTTAATGGTGCTCGACTGTATGTACGACATTGAAAATCAGGTGGTTTTTG

>JAP05776.C|Supercontig_0000003:1301876-1302095

TTGATCTTCCAATTGTTTAATTCGTTTCGACAATAACGCTAATTGTTCTGTTTCACTGTGATTATTTTTATTTTCTGATGAAATATTTTCATTAGTTATAGTATCACGTCTATATCTTTTGACTAATAAAGATTCAGTCTGTTCTAATTTACCAGTATCAGTAATATAAGCTGTACCATGATGTGGACTTAAGAAACCGCTTGTAAATGGACATTCTTTC

>JAP05781.C|Supercontig_0000040:916749-917098

TTATAAATCTTCTAATATACCATAATCTATAAACAATCATAAGACCAAAGATTTCAGCAAATGTTGAAGCTGTATCTGCTTGTAATGTAATGTGTAAATAATTACATAATTCTAATACTTTCGATGAATTCATCATTCTTGATGGTCGATTAGAATGTCGATATATATTATATATATCTAATGAAAAACATACAGAACATATAAGACCATCTATAATGAATAATAATTGATGACCCCATTGACGAATACCCATAGCCCATATTTTAAATGGAATTTCCACAACAAATAATGATAATATAAATAAACTTAAACATTCTAAAGCTAATTGACCTTCTAAAATTAATCGTTGA

>JAP05784.C|Supercontig_0000121:837919-838370

TTTCAGATGTCTTTGAATGTCATTAACCAGATTATATCACAGTTGACAAGCCACGGAACATTTAACCAGGTTTTCAGAGGAATCCAAGCTGTTTCACTAACAGATAACTGCCTAACATGTAGATTCAAAGTTACAAATTCTGAAGCCAACTCACTAAGCACTTTACATGGTGGATACATCCTTGGGGCTATCGATTTTATAACTTCTGTCGATCTGATGAGATTAGGGTGTATGAAACACGTTAGTGTTAATCTTGAAGCATCGTAAGTCATTGTATATGTATATATTCTAACTAAAAATTATCGAAGATTTATAAACCCAGGAAAATTGGATTCATGGATTCGGTCAGATTCATATATACTAAAGAAAGGGAACCGTATAGCCTTCTGTGAAATCAAATTCGTGAACGAACAGTCAGGGGAGCTAGTTGCACGTGGGACTCACACAAAATA

>JAP05793.C|Supercontig_0000044:2359943-2360039

GTTAAAGAAACGGAACACAGAACGACAACGTCGTGCATGTATTTCAGATAAAATGAATGCATTACATAATTTAGCTATGAAATTAATTGGAGAAGAT

>JAP05795.C|Supercontig_0000610:46851-47110

TCATCTATCAAAACTTCTGTGCAAACTAATACTGTACCAACTTCCTGTGATAATTCACAATATTGTAATTTGAACACTGTGGAAACAACAACACAGCATGATTGGTCAGCGAATGTAGATTTCACTCACAATCAAACTCAAACAATTGATGAAGATATAGAATTGTGGTTGAACAGTGTTGAAACACAAACAAATCTAGCGTTATTTGATCCCAGCTTCTTCTCAGATATATGTGTTGGAGTGGATGATGATTTTTTCCA

>JAP05797.C|Supercontig_0000063:747020-747333

TTTAATACACCATAACTTCACATAACAAAGCTGCAAACTCTTGTGACCACCTTCCACGAACTCCACGAGCTTCGACATATACATAACGACCAATTAACGGTTGTCTGCAAGGTATATGTAAACGTGGACTGAATATAGCATCATTAATTCTAGTTACAAAACCACATAGACTAGAACTTGATCTAGATAATGTTTTAGTATCGTGGTGACGTGGCTCCGATTCGACATAGATAGATAATCTTTCTAAATTTTCTGAATTGAATTTTGTTTTCTCTTCACTGCCAAATAGAGAAGACAGAAGATATCTTCCATCT

>JAP05804.C|Supercontig_0000360:134844-134991

GTGCTAAGTTCATAAATGCGATTAGCAGCTACATCTCTTATGTGCTCCCATTGAGTCTCTTTATTTCTCCCACTCACTTGTTGCAATTCCAATTGATTACGAAGCTCAGCTATTTCGTTAGTTAAAGAGAGTTTTTCATCTTGTTTTG

>JAP05806.C|Supercontig_0000093:884902-885107

TTGTATAAAATCATCTTCTTTCAATAAAATATATAATCCTTTTGTTGCCCATTCCCAGGTTATCATATTATGACTATCAGCATAACTAAACCAATCATATAATGTATTAACTAATTTTGTTTTCCATGTTAAGTATTCTTCACGATTAGTTTTATTATTACTATTATATTCAACAGATAATTGATCAAAAATTGGGAAACATAATT

>JAP05822.C|Supercontig_0000063:897362-897519

AATGTATTATTAAAAGGATACAGGTGAAATTATATTTGATGCTTGTGCACGAACTCGTCGTTCTAACGATTTTTCATCCATGTAATAGAAATTATCTTCTATTATTTTTGAATTGAAATTAGTCCATAAATTGTAAAAACATTCTAATGGATTATAAC

>JAP05826.C|Supercontig_0000199:112751-112826

ATTAAAGTATCTGGACTGACAGAACTGAAAGCACATATAGCTTTGAATTTCGTTTCCAAAACACATCGTACATGGC

>JAP05834.C|Supercontig_0000456:51679-51813

TGGTATATCCAATTTTACATCACCCTTAATTTCGGATTTTACTAAAGAAATTGAATTACCACGTTGATTATTCAATGATTGAATAAGCACTCGACTATGACCAAGGAAATGAAGTTGAAATTCATATTTCAAATC

>JAP05844.C|Supercontig_0000195:1170800-1170928

GGTCCATACATTTGGAGTTGCCTTAGTATTTTATGGTTGTGGGTAGCTATGATAGTATACGGACATTCATTTTTCAGTAATAGGAATTCTGCCATAGCGATTGTAAGCGCATCTAACTCCAACTGAAAA

>JAP05873.C|Supercontig_0000202:119993-120156

GAACGAGAAACGGTCTTAGCAGAGAATAATAGGGAACTATTAAAACTTCGATCAATAGAAAAGGAATATCAGGAATTCCAACACCAAATTTATGCAAACTCTCCCCCTGTCAGTACTCAGCTCCCGAAGGAAATTCACGAAAAGGTGACTCAGACTCCCAAATC

>JAP05897.C|Supercontig_0000299:660784-660900

ACCTTCTGGAGATCCTGAGAAAGATATCAGGGCTCACCTTTTGCCTCTCAAAAGGTCTTATATGGAGAATTTGTCTTCCTATTCACAAGAACTGGATTTAGAATTGGGTAGACGTTC

>JAP05928.C|Supercontig_0000445:6444-6613

GATAAATGGAATAATTATAAAACATGTAAAGATACATCAAAATCACGTCGTTCAATTGTTCCAACTGCCAGTAATAATAATAGTTCAACTGTCAATAATAATAATAATAATTCCGGTAATACAACACGCCCAACTTCAGCTAATCTATCAGGATCACCTGTAGCTCATAC

>JAP05964.C|Supercontig_0000021:2342212-2342482

GGCCAACTCATTTGAATCAACTGACCAATAGACGAATTTCTTGGTAAATATATTAGAATTTTGTTACAAACATGTCTTGAAATATTTAATGCTTTATTAAGAAAGGGAATGTCTTCAAGATTTTTAATAGGTTCCATTTCTTTCAAATCATATAACCAATGTCTCCGTTTACGTTGATTCCAACTAGTTGAGCCAGGTGGAAAAACTTTCCCAATGTAATCAGGACCACCCCAAGGTGGTGACATGAATATGATATCGATGAGTGAATTAA

>JAP05991.C|Supercontig_0000491:494853-494958

TTCATATTGGTGAAAATATACCATTTGGACAAATCTACATCCAACTGAAATCGATTCGTCCAGGACACGTATATTCAAACTGGTATATACTATGCTCAGATCCAGC

>JAP06000.C|Supercontig_0000071:471011-471241

GAAGCACGTCGTAATTCTAAAGCTGCTGCAATTATCTCGCTTTCATCAGACTGAGAACTGGTACTGCTTACATCTAGAACTACATCACCTGAGACACCACCGCGTAAATAATTGCGACATTGAAATGTTAAATGGCCGGCTGTATCAGCCGTTAGTAAGTATGTTGCTGCTTACTAAATCCACATTTTTTACAGCCCACTCGAAAGTTCGTCCTGGATTCACGGGGTTTAG

>JAP06008.C|Supercontig_0000300:595789-595874

TTACGCTCAGGTGGAGCAACTATCTTGATCTTCATTGTGCTCGGTGCCAATGCTGTGATCTCTTTCTGCATACGATCAGCAATACC

>JAP06009.C|Supercontig_0000191:92864-93088

TAGAAAGCATAAAGATCTTTCACCTAATCATCAATTATTGTATTCACTAAATCACAATAAAAATGGTACCAATAATTTGGTCAGTCAACAACCAATTCGAATAATTACTGAACTAAAAGCACGGATTCAACGATTGAGAGAAGAAAATATGGCGCTACGTCGGCTGCTTTTATGTCGACAACTTGTACCGGTAAAGCAATCAGATCAAGAAAGTGAACAACAACC

>JAP06044.C|Supercontig_0000263:345535-345809

TTTGTAATACATTAATTGATAATCAACGTTGTCTAAGCGTTGAAAGTCCAGATCTTCATGATGCTCAACAATCGGCAATGAGTAGTCTGCTGAATAAATTATGTGCTACTCAACCTGTGGTTGGTTTAATATTCTCACGGGAAGGTAAAGTTGTCATAGGCCAGTCACCAGCTGTATTTTTTCTCTTAATTGACTTATTTTTTGTAATTGTACCTTTAATACATTACCAATTGTACTGTTTTATGAATATACGAATATATTTCTTCAGTATAAAA

>JAP06117.C|Supercontig_0000367:89700-89773

GGTTTTATATTAATCATGAAGAAAAGACTACTAGTTGGGATGATCCACGACCTGCATATTATTCTGCTCAACAA

>JAP06126.C|Supercontig_0000227:106447-106553

TGACCCAACTATACCCAGCAACATGTACCGTCGAAGGATATATGGCCACACTCTCCAGTGTCCTCTAAGTAACTGGATGTCAACATATAATCCAACAGACCCAAACA

>JAP06131.C|Supercontig_0000055:1075940-1076133

AGGTATCCCTGGAACTTGTGAACTTATTTTAGCTGGTAAACGCTACTATCCATTTGAATTTGAATTACCTGCAGCTTGTCCATGTTCATTTGTTGGCTCAAGAGGTAGTATTGAATACAGGCTAAGAGTTTATTTATTATTATCGAATGGAGAACAATTATGTGCAGATAAAGGATTAAAAGTTTTACGTAAAA

>JAP06163.C|Supercontig_0000157:962886-962993

GCAAAAGTAACTGGAACGAAAAAGCCGCTTTGCAGCTACAAAACAAAACTCTAAAAACAGTCTCAGCTATACAAAATCTTATTCAAAATATTATAAAAACTTCACATT

>JAP06221.C|Supercontig_0000209:310899-310979

GACTGCCACCTAGTAAAACAGCTGGTTTATACCTTAAGTTCGGTCGTTGACTCCAATATATTGGCACAGAACCACGAAGCT

>JAP06249.C|Supercontig_0000167:328070-328245

TCATTTTCAACATTCATTGGAAAAGATAAACTCTCCATGAGACCATCCACTTTTTCTTGAAGTTTACTAAGAATATCTGCATCGATAAACAAATGATAGTCATCTAAATCCGTATGTACGAAATTTTTGCCCAAAACTAAATTTTCTGCTAAATGTAGGAGAAATTGTTTCATAGC

>JAP06305.C|Supercontig_0000015:422933-423122

ATTCCTCTAGTTAAACTAACAGCAACAGATTCAGATGAAGGTGTGAATTCACAATTAACATTTCATCTTCTTGATGAGTCAAAGGAAAAAGATATTTTTACAGTAGATCCTCATACTGGAGAACTAAGTCTACTACCAACAGTGGATCCATTCAATATTGGAGGTCGCTATCAACTGAAGTTTGAGGTAC

>JAP06314.C|Supercontig_0000178:641036-641220

TCGTCTTCATAAGATGGATTATCATAGACTAAATTTGTATCATTCTCATAATTTCCCCATTCTGACTCACAGTAAGCAGGACTCATTTGAGCATTCAAAACTCTTAAACTTTCTTTCTTATCTAATGCCATTTGATACATAATGGAGAGCATTGCATCTCCTGCACGTTGAATCTATTTGAAAAA

>JAP06319.C|Supercontig_0000055:511342-511476

CTTGAATTTGATTCATCTTGACAAAGCAACTTAACACAAACAGCTCCAGCATCATATAATGTCGGGTAGATTGATTTCAATCCAGGACAGACATTCCAATCACATGTAGATAAACATTTGAATGGAGTTGGATCT

>JAP06335.C|Supercontig_0000054:205810-205912

ATGCACTTACAGTTATATGACGAGGATAGTATTCTGGTGTATCTTCCCTATCTGTAATCAGCGAAAACCCAACATATCCACACACTATACCGCATGTTCCTAG

>JAP06363.C|Supercontig_0000021:1754285-1754552

TAAGCAATCCAATCAGTGGACTTTTTTCGTGTATTGTCGGTACAGACGAACTCAACGTTATTGAATCAAATGAAATAGAGATACGAGATGCTGTGTTTTACATTCTTCAACTAAAGGGTACTCCTTCTGCAAAGATAGATGTTGTTCCGCCAATATCAGAATTTCCGTATGAGTTGAGGTGTGTAGATGATATCAGCATTGTATCCCGCTGGCCATACTGGATGCACCAACTAGATGGCAGATACGGACCATACAGATGGGAATATTG

>JAP06381.C|Supercontig_0000227:602223-602548

ACGTATTCCATCAGTGTTGTTGTTTCGGAAGACCGTTGTTTGACCAAAGACAAGCGTAAACCAATATTACAATACAGTCGAATATTTTCAGCTTCCAGTATTTTTTGTAGATCTCTTTTGGTTCGTCTGCTTAAATGAATTACTGGCCATAAAGAGCAACCGGCTGTACAACAACAACACAATAACCCACAAATAAACCATCTAACGTGACTAGGTAGTGAATTCTCCAAGCGCTCATTAATTTTACGTATACTTTCCTCAAATTCTTCAGGAGCTAGTTTACCATGTAATTCATTCGGGAATTCTTCCTTGAATTTATTGTTCAT

>JAP06386.C|Supercontig_0000282:236729-236951

ATGGAACATGTATTACACAATTAGATGGATTTTATTTAGAAGTTGGATTATCAGTTCTATTTGGTTTAATTGTTTATCCATTTTTCTTATATCCAATGGCTTGTCGATTAGATAATTTACCAAGTTCAGCTTATTCATTTCGATTAACTGGTACTGGATGTTGTTCACGTCAAACACGAAATAAAATTTCTATGAATGAAAATGAAACTGAATGTTCACAATT

>JAP06420.C|Supercontig_0000486:214507-214698

TCTTGGTCCGTAATTTTAAATTCATCTAAACCTACATCCCAAATCTATTCGTCTAATACAACTCATTCCAGTGTACCATGTTCAGGTGTGACTGCATATTCCTCAACTACAAATGCTGTAACCAGCTCAAATACAGATATGGTAGACACATTAAGTAGTCTATTATCATCTTCAACTTCAAAATTAAATTCT

>JAP06435.C|Supercontig_0000075:228288-228403

TTTTTGAAAATGTGGCCGCAGACAAAATCCGAGCTTTTGTTGAAGGTTTGAATGGTCTTATTTTTGCATATGGTACCACTAGTTCTGGGAAAACTTACACGCTTCAAGGTAATTAC

>JAP06461.C|Supercontig_0000191:445153-445259

TGATCCAAACACTGAAGTCCGAAAAGTATGATAACTGAAGGCTGGTTCGCATTACACAGAAAGTCAACATCAGCAAACTGGAATTGCTCATTTTTGTAATTCTCAGC

>JAP06474.C|Supercontig_0000009:328981-329219

ATTGGTAATTTTCGTTCTGGTATTTCTTCCAATCGGTTAAAATATTCACTTATCTTATTTATCACTGTAGTCATAATTGATGCCCAATTAATTGATCTTTCATTTGAAATTATCATTAATTCATCATAAATTTTATCGGATAAACTAATTCGTTGACACCAACGATCCATGAAAGGGTCAAGATGAAATAGTTGATTTGCTGATAAGCTATTTAGTAGTGCTTGTCTTTTAAAACAAGA

>JAP06503.C|Supercontig_0000673:242975-243143

GAAGTTGCGACTGATTTTCTAATTCAAAAACATCGCTAAATGTAGCCATGGCTTGCGACTGGGTGTCCAACCTTAGACATATGCGCCAAACGTCAGGGCGTAGGTTTTCAGGGACAGGACGCATGGCACAGATATCCCGAATCCGAAAAATGTCAGCATCCTCTGAAAA

>JAP06507.C|Supercontig_0000067:715015-715129

GGTTTGGTTTATCTCATGCATTCATTTTAAAACCTGATGATGGTGAGATTAAAGGTGGGACACGACTTAGTTTATTACTTAGTTCAGTTGAAATGGCTGTTTATGCAACACGATG

>JAP06536.C|Supercontig_0000461:82434-82597

AGGCTACTTGCCTCTTTCGACGTGTATTAGCACTGTGTCTCAACGAAGATTCAGTCTCACCCGCTTTCATAGCTAGAAGTCTTGGAATAGAGTATACCGTGGCTCGTGGATTATTTAATCGTTTAATCAAGGAGCAAGTTATTAAAGGAGCAGGACCACGAAGG

>JAP06562.C|Supercontig_0000221:691343-691459

AGCCATGGATAGCAAGGTTTGATACGGATAAAGATAATAAAATCAGTATAGAAGAGTTCTGTCGTGGATTTGGTTTAAAAGTATCAGAAATGTAAGTATTAAAATCAATAAATTATT

>JAP06565.C|Supercontig_0000011:388261-388413

ATATCACATTTTTGAAAAACAGAACTCAGTTCTGTAGCTGTCAGATTAGATAAAATCAAACAAATGCGTTCATAATCCTCCATAATTCCATTTCCATTCGAAACCTTTGAGACAAGATCAAATACTTCATCAGGAATAGTCATTATGCAAGTG

>JAP06569.C|Supercontig_0002017:23996-24122

AAGAACTCAAACGGTATGCTGATGATGACTTAAACTCATTACCTGCGGGCTGGAAATCAGCTGCTGATTCGACTGGGCGATTGTATTATTACAATAAAGAAACCAATAGTGTTCAGTGGGAGAAGCC

>JAP06574.C|Supercontig_0000015:48501-48594

AGATTCAACTGTGATTCCATTGAAATCTAAAGTTGTGGCACGATGACGACTAGATTTAATTTGACCATTTAAGCTGACAACTTTAGCACAATCT

>JAP06584.C|Supercontig_0000012:717645-717800

ATCTGAATTTGATAATGAACATTTACTATCGAATTCATTGGGAATACAATTTTCATTATTACCACTTATCAGATTACAACCTGCAATGCCACGAATGTGTATGTTTCCAGGTCGTATTTGCAGCGCATTCGATTTACATGAAGCTATTGATATATT

>JAP06592.C|Supercontig_0000046:1621812-1621938

TGGATTCGACGTCTGTCATTGTTACTTTGTGCAACCAGTGGAGTTCTTTCGGTTTTGGGGAATTCGCACTTCCTCTTCGGTTCGAAAGTGGCAAATCAACTAGCTATGTGGTTGATACATGATCCAA

>JAP06598.C|Supercontig_0000065:367380-367487

TGAAGTCTCAATGAGATGTTGCAAATGCTCAGCTGATCCGGCACAAGTCCCAGTGATATCATGCTGTAAGCACATAGCTTGTTTTAAATGTTGGCGACATATCATCGA

>JAP06605.C|Supercontig_0000196:229362-229551

ATCTACCAATATACAGAATAACCACCTTGCGGATCGACTAACAATTTCAAACTTTCTAATCGCCCAGCCATAAGACAATTATTCAGTTCATGAATTAATCTTTTTCTGTAATGAGATGATGAAATTTTGTAGTTTTGAGGACAACAAGATGTAAGTTTCTTGAGAACAGAAGGACTAAATCCAGTTGAAT

>JAP06610.C|Supercontig_0002044:79209-79360

CTCACGAACAAGTGATATCACTTCTGCTTCAGTTGGAACACCACCTAATGCTCTGATTACTGTCCCGAGTTCTCTAAAGTCTATTGTTTTGTTGCGTTCGTGGTCAAATAGATCAAAAATCTCCCTTATTCTCACCTCTAAATCACCTAAAA

>JAP06620.C|Supercontig_0000184:122993-123143

AGAAATTGGAAAGAATGAAATCAAATGAAATCTGTTTTCAAGCATGGAAGCAAAGTAAACATCAACTTTTAATCGATACAATTAAAAAACGTAAAGCAGAAGAATTGGCACAGAAAAAGAAGCTTGAAGAAGAACAGGAACGTAAACTGAA

>JAP06649.C|Supercontig_0000600:67742-67914

GAGCAAGAAAATCAGCTAGATAAAGTGATACTTTTGCTTAAGGCTGCTGGAAATGCTCCTGTAATGAAAAAGATAAAGTGGTCTGTAAGCAGGAAGCAGAAAATTCATTGGGTAGTGGATTTTATAAGAAAATATATATCATGTGCTCCTTCTGAATCTTTATTCCTCTATGT

>JAP06667.C|Supercontig_0000084:543837-543980

AGATGAATATTCTTGATTTGTTACAGGATGGTTCCATTGCGTAGACCGAGTGTTATGATCCACAAAATAAAAGTGCCCTGATTTCTCATCAAGTCTCATTTCCCAACCAGGAGGTAGAGATTCTTTGGGATTCATTTTTGAACT

>JAP06679.C|Supercontig_0000053:65030-65396

AATTTGTTAAAAGTTCCTGAAGGGTCATCTGCTGTACAGTTAACTACATCAGCAAATACACCCCTTGTCATAGGCATCGATCACATCCAAGCAAGACGTCTAAGTATGGCATTACAGCACTTATCAAGAAATTCCTTTCAACCCCAAACACTATCCTCACATAAGCACGAAACAACAAGTATGCTAAATATGGCAAGACGTTTGACAATGGCTAGTTCAGCCATTGCTTTGGGCAGTACTGATCGTGATTACTACGGCCATTCATCATGGTTAACTGGATCTAGAGTTCGACATGGTGTAAGACGTAGTTTAGCATGGAATGAGTCTGGTGCAACTCGATTTTATGAACATTGTTATGATTGATAAA

>JAP06682.C|Supercontig_0000042:1652142-1652382

GAATAATTAACATTCATAACAGAATTAGAATAAGTGCAGTTACATAAATTATTAGATACATTATTTATTTTTTGTGTAGCGCTATTCTCAAGAATAAAATCTAAATCGAGTAAACTTTCATATTCATTACTTGAAGTACTACCGCTACATTGTTCCGTACGAAAGCTACTACCAATAGATGAACATTGTTCCGAAGTCGTAGGTGATGTTAAACCTGAGAGATTGCACATGTACGTTCCTC

>JAP06690.C|Supercontig_0000024:921250-921360

GCTTTGCTTCTAAACAACGAAGGAGTTGTTGTAACCGGTTTATTAGCAACTTTGAATTCTATAGACTTCTGCTTCATACTGAAAGATAATCTGTCTTACATGGATAAACCG

>JAP06725.C|Supercontig_0000649:116039-116131

CTGCAGGTATCTGCCCAAGAAAATAACCCCAGAAAATGACTCCGAGAAGAACTCCCCAACTTGAGCGATCCCAATCAAACGGGCCATCTATCA

>JAP06741.C|Supercontig_0000238:329956-330146

ACTTACCTGTGATAATATCATAAATTTATTAATTTGTTCAATAGATGGTGTAAAATCCTCTGGTCCATAAGCATTTATCGCACCTGATCGACGATTGCATATAACAGCTTTTTTCACTAGTCTTGTACCCAATATACTGTTTTTTAAAATTCTATCATATACATCAGAACTGGTTGTTTGATCAAACTAGA

>JAP06742.C|Supercontig_0000461:235079-235223

GCTATTCAGACAAAGAATTTTCTTCAAGTGCTTTTCTAACTTTGTTTTCAATACGACGAGCTGCACTTTGAACTTCTTCGCGCAATATGTTTATATGCATCTGCCAGGTACGATTAGCAGACTCCTTAGTTTTGAAATACGACTA

>JAP06745.C|Supercontig_0000448:81202-81318

TAATTAATTATTTTTTTAATAGGATTAACAAAACGTCCTTGGACATTACGTGACCCACTGAATTGTTGCTTGGATAATGCTAAATGTTGTAGGCGTAAAAGGTACTTTGAACGAAAA

>JAP06769.C|Supercontig_0010621:1504-1578

GATCAAGGGTTGTGTTTCCTCACTTTGTCGCTGTGTTTTGTTTTCATATATTTCGAATTTAGTCTCTACTCAAAA

>JAP06773.C|Supercontig_0000730:5971-6250

ATCTTAGTTTCCGACAGATGATGAATTCAAAAATGATGTTCGATTAGCTGCACCACCACCGAATTCTACAGCTTGTGAAGTTGCCTGACGTAGTAAAGATAACTGGTCCATTGAATTAGTCAGAAGATTATGTAAAGTGGTTAATTCCATATTAGTCAATTGACTAACTCGTGCTGTTGACGATCCACTAGAGAGGAAACCATTAACAGACGATAATAACTTTCCAACACGAATATCCCATACATCACGAACAATGTTGCGAATACTTTCAGGACTAAAA

>JAP06791.C|Supercontig_0000159:903443-903535

TCAATTGTAATCATCTTTTAATTTACGAGGTAGACCAGATAATGAACTTGTGAATGCTGGTTTACGTTTACGTGCACCAACATTAACTGTACT

>JAP06820.C|Supercontig_0000235:686769-686869

TCAGCTTTTAGAGCTTCATTTTCAGTCTCTAGATTACGTAGATGCATTGAGAAATCACTTTTTTCTGCAGCTAATTGATTTTGATATTTCCTTTGAAGATT

>JAP06847.C|Supercontig_0000153:1196194-1196273

GAATTCAATAAATGTGCAATCAGTGAGTGGTTCTGTATATTCAGTATTATAGAAATCACTTGGATAACTGGTTTGGAATT

>JAP06908.C|Supercontig_0000137:270379-270493

GATGAAACTTTGTTCTTTGAAGGTACAAACTATTATGTATTCGATAATATAGCAATGCATACACGTCCTGGCTATCCAAAACAAGCTTCATTAGGTATACTTGGTTGTATGAAAT

>JAP06925.C|Supercontig_0000167:367362-367588

TTTGATGGAATAGGGGAATTATCGAAAAAGAAGCTAGCTGATACAATTTGTTCATCAGGATTATTGGAAATGCGAGGTTTTGGATTTTTTATTGTTCCCCATACCCAAATCCAATATAACGCTCCCATAATAAAAAATACAGCGCCAAATTCAATTTTTATGGCTATTAACCATAATAACAGAGTAATAATGAATTGTACATACCATAAATTCCAAATGGCAGTAAT

>JAP06933.C|Supercontig_0000025:1197278-1197623

TTCATATGTGGATCCCATTCCATTAGATAACAGCCTGACCAGATCAATTTGTCCTGACCGTCTAATATCACCGCTTGCTCCTCCCACACCAAGTAAACATGTTCAGCTTTCTTCAAAGTCACCAGCTTTATCAAAATCCACTGTAAATTCAGATTTATCTAACAAAACTAATCCCGTTGTAGATGATTGCCACAATAAGCGCAGTATGGATTCTAATTTTCTTAATGAATCTTATTCAGGGAATAATGTTTCATCTAAGCCAAATGTTCATCCTACCAATATATCATCGTCTGTCTTTCAAGAGGAATTACGAGATAGATTAAAACAACATTCTCAACAGCTTAAA

>JAP06966.C|Supercontig_0000301:187069-187256

AGAATAATTTGTTTTTGGAAGACCCACAAGTTCCAATCGTATATTTAGTCCATGAGCAACACTCTCACTGACATTAATTTTTAATAAGACATGATCGAACATACGATACTGTTGACATTCATTAGTTTTGGAGTTCCGGATTTCTAAAACCCCACAATCTGATCGTTCAGATGAATAAACACGTTCAA

>JAP06970.C|Supercontig_0000074:159828-160060

CCAAGAGTAGAAGAATCTTTAATTGCAGCTTTGCACAATTCAATCCGTGTTGAATGATACGGTACATAACGATCTTGTGGACTTCCAACTAACAGAACATAACGAAATAGATCCAAGCCAGGACTAGTACTTAAACGATACAAATATGTATTACGTAAATCCGGATCATCTCGCATTCTTAATTGTGAAAGACTTTCAGATTTTTTAATTTTTTGCATAACCCATATGCCCAT

>JAP06982.C|Supercontig_0000165:140417-140550

TTGGAACAATGTCACAATACATTTCTTGCTTTCTATCAAGAACATCATACTGGTCGTAAATTAACATGGTGTTATCATTTATCACGTGGTGAAGTTGTTACTAACTATACTAAAACTAGATATATATTTCAAGT

>JAP06983.C|Supercontig_0000030:449340-449652

TTACCCGAGTAACAGTGGTGAACTGTAGTTGAAGAATACAGCAGCCGATATAAAAATCCACCCGCAAAATAGACGCAAACCAAAAGATGAAATAAGAGCGTAGAATAGTCCCCATGTGGCACGTGCAGGACTGGCTTTTATTGTACTCTGCTTGCATACTTCCAGATTATATGAGTTTGAGTTGTTATTAGTAGTAGATGGCGACAAGATATCAACATCAGGTGTTGCATTCAACATATTTGCATCATTCGAGACATTTTCAGTATCTTCATTATTCTCCTTAAAATCCATCAACAACGGCACATCATCATCC

>JAP06986.C|Supercontig_0000490:299698-299804

AAAGTTGGTGAAACCAGTCTGCCTTCGAATACTGGGGACTTACAGAACTGTTTAGTTAATTTTTATTTGGGTCATTCATCTCAATATGTCCGCAACAGTGCATGCAG

>JAP07015.C|Supercontig_0000020:1117866-1118090

GTAGTACTAATCATAACCGTTTCTCTGTAGTAGATCCAAACTTTCAAACACATACTTGTGCAATTTTTTGTCCTTTAAATAGAAGTAGATACCTCTTTAAACCATATTTAATTGAAGTAGACCCCTCATTTCGACGATTGGCTGGTTTAGTTACTTTATCAAATTGTAGAGATGTTATTGTTGTTGATCAAGGTGCTCAAAATATTTCCAGAATTCACTATGCAT

>JAP07018.C|Supercontig_0000018:27253-27381

ATTTACACAGGAAGAATATACTACAGCTGTCCAAAAGACAGTGGAACTCGATACTGTACTTGAAGAGCAGGCTGTTCAACATCGTGAAGTTGAACGATATGAATCAAACTTATTTAAATCCTACTTTTC

>JAP07019.C|Supercontig_0000286:211761-212061

TGCCCCCTCTAACCAAAGTTAAATACTCCGCGATAGCTGTACGCGAAGTTTGATCTAAATGCCGAGCGGAACGATCACATACCCAGCAATGAACCCCACGACGACCTGAATACACCCACAATAAATGCTGAAAGCCAAAGTCTTCTCTTAAAGCGCGGTCGATACAGAGGACTGCAGAACGAGCAAGTGGCCAGCATCGAAGACAAACAGGAGATCCAGAAGTACTTTGTTCACCACAACAATAACGAACTTCATCATAATCAGTCAAATCAATATCGAAAACTAATTCTTTCCATTCAGG

>JAP07026.C|Supercontig_0000123:957467-957628

GATTTATTTCATCTAGAATTCGTTTTAACTTAATAGCAAACAAGAGTACCCCAGTGAGGTATGATAGGAGGTGGCACATTGGCATTCGACAAATACTTTTGATAGTTAACATCCTTGTCCGTATACCTGTCCTTAAACATAGTTTCCAACTCCTCAAGACTA

>JAP07048.C|Supercontig_0000097:304003-304096

TTGTAATATTGCTTTCATGAAATATTTTCCTTCTAACACGTTTAGGAACATCCGGTACAGCTGCCATGATCACATTAGTTAGAATAGTTGCTAT

>JAP07065.C|Supercontig_0000064:2437149-2437261

TGTTGTAATGCATGTTCAGCTGATTGAAAATTATTTCCAAGTGCTTCATCTGTATCATAAGCAGATTGATAACGTGAATAACGTAGACGACAATAATAAATGAATATACCAAG

>JAP07077.C|Supercontig_0000005:81507-81620

CGTTTACGCTTTCTTTTTAGCTTCTTAAGAAAAGCACGTCGGGCACGATGTCTGGTCATAAGCAAACCAATCATGAATCAAGCACATACGACATGTGAGACAAATCCATTATCC

>JAP07094.C|Supercontig_0000033:641905-642170

GTGCAACGAATCGAAAAGCGCATACTTTCATGACACTAATAAATGGAAAATTAAAAGGATTTTCTTCAGGTGTCATATGACGATTTTGTGCTATACTCATAAGATATCGAATGAATTGAACTTTAGAACATTTATGCACTAAAACTAAATCCAAACAGCCATCACCTAAATGTGCCCAAGGTGCAGGGCCACAAACAGCTCGTGAACATCGACAACTTTGAACAAATGCATTAATTGCTAAAAACGTGCCTCGAATAGTATGCCAA

>JAP07129.C|Supercontig_0000012:1445809-1445907

TGCCAAAGGCATCGAGCTGAAGGATAAGTTTCAGAATATAGGAGCCAAATTGGTACAGGATGTTGCTAACAACACAAATGAAGAGGCTGGGGATGGTAC

>JAP07132.C|Supercontig_0000131:691174-691318

TTTCAAAAACTAACCTTGCAACATTGCAAAGACATGTGAGCATTTCTTGTGGGTTTAGTGCAGACAGAACAAAAATTAACGTTACTAGATCAACACCATGTTCAGCATTGTTGAATTCCTGATCACTAACTGAATTCAAAGAGGA

>JAP07153.C|Supercontig_0000247:28437-28613

CCCACTGAAAACTCTTTGCACAGTGTTTATGTTCTATTGCTTCTCAATATGCCTTACATTTTTCAACAGCCGTTGTATAAAAGTTCGCGAAATTTCGTTGAATAATTTTAAAAGTTAGATGTTCCCGTATCCACTCTCAATCACTTTACTGCATATGATCATTAAATTCTTATTATC

>JAP07156.C|Supercontig_0000075:163868-163969

TCTGACAAATAGTTGAAAATCTTTTAAACTAGATTTTAATGCACTTGTGTAAGATTGAATTAATTCTGGAGATAATTGTTGTCGTGGCAGAAAGTAATCTTT

>JAP07199.C|Supercontig_0000026:1467439-1467665

AAAATGATTAGCTAATGCAAGTGCACTAGCACATTGCTTTACATACCAAAGATAGATATAACATCTTTTATGCATCAATTCGGTAGTATGTATCTTGTATATCTATCCTGAGTGGTAGGATGCAAGTAATTGAAAGTAATATGGTTTACTCGCTTTCAAAAGTTTTTTTGTTAACTGATTTTTGTTATTATTACTCAATTTCTTCTGTTGACAATATCTTATATATA

>JAP07249.C|Supercontig_0000298:647721-647832

AAAACAGCCAGGTATATCTTCCCAGGAATAGTAAGGGGTGACAATATTTTACGAAGTACGTAACTCTCTGCTCCACTAATGAGAATCCGATTAGGATCTGCCTGTGAAGAAA

>JAP07256.C|Supercontig_0000053:395691-395971

GATACAGTCACGTCAGGATCTACTGCCGAACGTGATGTATTCAATGAGAAACCAACTAAAGAAGAAGTATTAGCTGCAACAAGAGATTCAGCAACAGCTGCTTCACGATTAGCTGGTAGCGCTATTCTACATACAACTTTAGGTGATATACATATTCGTTTATGTCCTAGAGAATGTCCTAGAACAGTGGAGAATTTCGTTGGACATTCAAGAGCTGGTTATTATAATGGTCATATATTTCATAGAGTTATTAAAGGTTTCATGATTCAAACTGGTTGTCC

>JAP07269.C|Supercontig_0000055:359339-359495

AGACGTGAAGCTTCAAGTGTTTTTATTCATAATGATTGTGTATACATATGCGGTGGAGCAAATGAAACTAGTTGTGTAAATACCACAGATATTTTGTCATTGAAAACTACAAGTTGGAGTAAAGGCAAACCAATGATTTTACATCGGGCATTTGCTG

>JAP07292.C|Supercontig_0000249:137637-138065

TTATGACTTTTTCTTGTAACTTTTTTCAATTTAGGTATAGCATACGTACGATGTTCATATTCAATCACTTCAGATAATTTTGTATCTAAAGGTAACCAATTGATCTTCTGAAACCAAACAGCTCGCACCACTTGGGTTGAACACCAAGTTTTTCTTCTACTATGTACAGATCGTTTTTTGCCAAACCAGTATATAGGAATGCATAGGAAACTTTCCATGTCTACTTCGAACATCTCTCCACGAACAGTAATCTTTTCGTAAATTGGGTCCGTGCTGCCGTTATAACGCTCTCGATAAGCTGTTTCGAGACGTATTGAATCATATCCACAAAATGGTTTCCATGTACTCACTCCACCAGAACGACGGAAATACAACCAACGGACAGCTTCGATAGGAACTTCATCATCAAGCAGAAAAGTGCTCATATTT

>JAP07302.C|Supercontig_0000095:345351-345556

CACTGGTCGTTATCTATTTACTGGCAGTCAAACTGGTGTTGTTTGTGCATACGATTTACCTCAATGTATTGATCAATTTTCTGAAAATGGACATACCTATTCTTCAAATTGGCGAGCACATTCTGACAGTACAAATGGTGTCAGTGTACATCCTAGTCTTCCTATTATAGCTACTTCCTCAGGTCAACGTAGAATTAAACAACCAA

>JAP07304.C|Supercontig_0000153:493190-493439

AACGAGGAAAAAGAGAAACAAGCAGCGGCTCTTCAAATAATGCGCGAAGCTCGCAGGCAATCTCAGTGGAGTTCTCGCGCGGCTGAAGAGAAGGCCATTCGTGAACGCGAAGAGGCTGACAGACGACGTCAGGAAGAAGAACTTGCTTTGGAAGCTGAGAGGAGGAAAGAACAAGAATTGCGCCGTATTGAAGAAGAGAAACGCCTTCTTGTTGAAGCTCGAAAGCGTGATGATATTGCAAAGCGTAAGG

>JAP07305.C|Supercontig_0000064:1922669-1923060

ATAAATAGCTAACATGCATGCAGATCATAAAATAAATGCTTGTAGATATTATTTATATTCAAGATAATGGTTTAGTAGTAAGTATACAACTAATAGGTATGATGGATTGACTAGGTTGTATGGTTCGCGGAGTAAATTCTAATGTTGGACTGGTAAAACTAAGAGTAGAACCAGGATGAATAGACATAATTGGGACTGGGAGGAACCCAGATACAGTAGTCAATGAACAAGGTGAAATTGTAGGTATAGTGATACATTCACTACTGTTTATTCGACTGCTGTTGTTATTATTACTGATACAATTATTAACAATAACAGTCGCAGCAGGAGTAGTAGTTATAACACCGGCTCTAAGAGTTTGTGATGAACTATGATCTGGTTGCTCTGTTTTA

>JAP07327.C|Supercontig_0000026:874269-874550

TAACTTGGCAATGACAACATCCCAAAAACATCGCAGTTTTGTTTCAGAACCAATTTCTGAAAAGGTTGTAGAAGAGTTACCAGGTATCGGTGAAAAACTTGGAGAAAGACTCAAAGCTAAAGGGTATGACAAAGCGTATGTCGTCTTGGGACAGTTCCTTCTTTTGAGGTGCGAAGAAGAGTTATTCAAGGAGTGGCTTTCACAAACATGTGGTGCTAATTCAAAACAATCCGGAGATTGTTACACTGCTCTTAAAGACTGGTGTAGTTGTTTCATTTTCTA

>JAP07333.C|Supercontig_0000307:240859-241054

ATCATTGTCCAAAAATCAATAATTGTTTTAATCATTGGAGCTTGTGCAATAATATACCTTGGACACCAATCACCTAAATGATATTGAAAATCAATATAACTTGCATTAATATAATCATTTTTTAATGATTTTAATATGACACGATTATTATCGTAAGGAACATAATTTTGATTACGATTTTTGGATGGACAACATA

>JAP07341.C|Supercontig_0000093:1243873-1243955

AAACTTGAAGAACCTGATGATCAAGGTTGGTGTAAAGGCCGAAAAGATGGTCGTGTTGGTCTATATCCTGCAAACTACGTAGA

>JAP07346.C|Supercontig_0000123:1225518-1225939

AACTCTCCAGCTTCCGTCCCAACGCCTCCTGCTACACCATGTTCAGTACTTCAAAACTCACATAATTCCTTGAGAGGCTCTAGTGAAGAACGACAGCTGAATAAATCCGTGAAACGTAAGCATTTCACAAAGTCACGCACCGGCGGGAGATATGAATATGTTCCAAAGCACCAGTTAGTTGCTTCAAAGACAAAATATTTCGACGATATTCGAGATGGAAAAATCGACCTGTGCTCAAATGATCTTTATTCACGTGTTAAAACCCGCAAGCGATGCGCTGCTGAATCTGCTAGCAACTCCGTAAGCAATGAATCTACTCAGAGCCGAAATGGCAGTCTTCAAGGAGAAACGGAATTAGAATGTGAAGCCTCTATGGAACATTTTTCAACATGTTCTCCGAAACGCGACCCAGTATCACCATC

>JAP07350.C|Supercontig_0000043:498534-498898

AATAATTATACATCACTTTCAACTCCAATAGATCCTATAAAATCAATTCGTGGTCTACAGAATACAGCATCTTTACTTGGTCTTCCATCCCTATCAATCAATACAATTTTGAATCCTCAACAACAAAATTCATTATTTTCTTTAGCTAATACAGAAGAGAGTATTATTGTCCGAGAAATGATTATTTCTAATGACGTGATCGGTTGTATTATAGGACGTGGTGGTACAACAATCAATGAAATTAGAAACGCATCTAAGGCACAAATCAAAATATCCAACTGTGAAGATGGTGCAAAAGAAAGAAAGATAACAGTTTCTGGAAAGTTGGATTCAGTGAATTTAGCTCAATTCCTAATAAACAGCAG

>JAP07374.C|Supercontig_0000152:752438-752578

CCTTTGAATAAGAAAGATTGTACAGTGACCAGTATTCATTTAACCAACGCCAAGTTTCTTGACAAACGGTTCTATATTTTAATTCCAAAGTAGTTTCATTCGGTTTAGATTTAAACTTTAATGGGTGGATGTTTGAAGAGT

>JAP07388.C|Supercontig_0000099:53608-53826

GTGATCTTGTGCACAGGCAAACTGCGATGACTGCAGTCGCTCATATGGCTCTTGGTGTATATGGGTTCGGCTGTGAAGACGCTCTTTTGCATCTCCTGAACGTCGTTTGGCCTAATGTCTTGGAAACTTCACCACATGTCATTCAGGCTTTCATGTTTTGCATTGAAGGTCTCCGTGTTGCACTTGGTCCTAACAAAGTACTTCAGTATTGTCTTCAAG

>JAP07394.C|Supercontig_0000480:505549-505755

TTTCAAAACAATGGGTTTGGTCTTTCGTCTACCTCCCAAAGAGTATGAATTTCTAGCACTTATTGTGGCTGTCTACTGGATATTAGTTATTGGTCATGCAGCCGCTGATTACAGTCTACTGATTGGAATTCCTTTGCTGCCACTGATAATCTTACAGATTGCCTTACTTTTACATGTCTGGAAAACGTTAAAGAAGCGTGCAAAATA

>JAP07397.C|Supercontig_0000635:94494-95326

GTTTGTTTTGGTGCATGTGTTTGCCCATTCTTGTTTTTCAATGTAATTCGTTCTCGATGGCTTCAACTACTGACCGTTTGTCTACGATGGATTGGTTTTATTTTGATGTTGAGTCTTTCAATTGAAAGAGCTATTATTCTGCGCAAAAATATTACAGTTGCATCGTTTCACCATAATGTTAGTATGTATTGGAATCCTTCAATCTATTCACTACTTACAAAATCTGAACCAATACCAAAACCGCCAGCCTTTCAACCTCAAAATATTCCAAGTTTATTCGGAGTTTGTGTGTACGTATTTATGTGTCATCATTCCATACCTGGTATTGTAACTCCTGTTAGAAATAAGAGTAAAATATTATGTAGAATATTTATACCTGTTTTCATTACAGTTCTATCATTTAACCTCTTATTATCTACTACAGCTATCATTGCATTTAATCATATTGAAGATATTTACACATTGAATTTTCTACCGAATAATGAATTTATTGACATATCACAAATTCCTTATACATTAGCTTTGGTAATAGGTTATTTTTTATGTTTATTTCCTGTGTTTGCGTTGACTTCATCGTTCCCAATAGTTGGCACTAGTTTACTTGGAAACATTTTTTCTTTATGTAACTTCTTCTCTGTATTCAAAAGTGATCAAGCTCAAAAAATTTTGAAGTACATATTACCATTTGTTGTGCTATTGCCACCTTTAGGGATTTCATTAATAACTCATAATGTTGGATATTTGACTGGTTTTACCGGGGCAATTTTTGGCTCAGGTATACAGTATATAATACCAGCACTGTTAGTATTTAAAGCACGTCGATACTTTACACA

>JAP07418.C|Supercontig_0000184:316648-316756

GAAATGGATAGAAACCAATTAATAAACAGTTCAAACATAATGATCGATAGAAACTTGCATCCGATATCCATTGGGATCGTAGTGAAAACCAGTGAATCCATGGATGAAC

>JAP07425.C|Supercontig_0000275:56904-56986

TCTGTCCTTACACAAAAGTAGAGGAAAGCTTCGGCATGCAAGCCTCACACGACTTGCTCTATTTGCGAACTAACATATCTATG

>JAP07435.C|Supercontig_0000088:893217-893580

ATCATCAGACGTTTTCGACCACGAAGTCGAACGTCCTCCAATTTGTTTAAAATTTCTTTGACTTCGTTTTCTTGTTTTTCCAAATGACCACCTGATTTTGTATTATCATGTTTAGGCTTTATCTGTTGATCATTCAGCATTGTCGATACATAATTATCACGATAGGACTTTATAATTAAATTCAGAAGAGATTCTGGAGTATTTAAATCGGAGTGGGCAGCGGTAATTTTTGACAGGGCTTTGTGATCAAGACCACATGTTAAGGCCCGTTCAAATACATACAAATCCACAGCTTTATCTTCTGCTAAACGCTGTGAGGTTAATCCAGAAGAAGAGCTTGAAAGTCCGAAATCTACAGGAACGA

>JAP07478.C|Supercontig_0000160:775969-776072

GATTTCAACTCGACATTCCGTTCATCTTCGCCGTCAAATTTTGTTTGGAAATTCTCAGATGCTTTTAAATCAGTTGTTTCAAATCCAATATCAATCAGTTCTCC

>JAP07481.C|Supercontig_0000007:1443837-1443950

AAAAGAGATCAATCAATCAAATCTTTATTCGATGAAGATAATATGTCAGCAGAGTATGAATTTATTATTAAACAGTTTGCTAATGTACGTATGGCTGATGTCTGGGAGATTGAA

>JAP07489.C|Supercontig_0000475:299152-299279

CAAGAAATTTATCATAATGTAATTTTCGAGCTTGTTGATGTTGTACACCTTTGAGATGATTTCGTCGATTAATTGGATTATCTGGAAATGACTTATCACAATAATCACAGATAAAACGTCTACCCATT

>JAP07497.C|Supercontig_0000111:633265-633736

GATCTTAAAGGAATATCAGCCATTTCACTACGCATACATAAAAATTCACCCATTACTGTCATGATAGCAATAGATAATAAATTAACAATCCACCATAATATAGATTTTGGTAAACTAGACGAATATATAATACAAAATATAAAATGCCAAAAATGTATAGTACATGAAAAATCAAGACATTGTTTACTACGTTTTACAATAAACCATAAACCAATACTACATATTAATGGTGTTAATAGAAAACCAATAAAGAGACATATACCAATTGAATCAGGAAAATGTATTTTTGAATCAATAAAAATCATATCCAATGTTGGTAAATCATGTGAAAAGATTATCAATAGAAGTCCAGAAATTGTACCGGCAGATATATAAAATATACATTGCATAAATATAATTTGACTGATAATTAAAATTGGATCCCAAACGGTACTACGAAAACCTGATGCCATTCATATGAAACAATGAAGCC

>JAP07498.C|Supercontig_0000307:496356-496523

AGCTTATATTTCCGTTCGTAGACGTCGATTGTAAATATTTTGACTTGGGACTTCCCAATCGTGACCGAACCAATGATCAAGTGACAATTGATGCAGCACATGCTATTAAACAATATAATGTGGGGATCAAGTGTGCAACCATAACTCCTGATGAGCAACGTGTAAAAG

>JAP07533.C|Supercontig_0000461:343846-343911

AGAAAGCACGCGTGAGGGTTTGGCTGTATGAACAATGTAACTTAAGAATTGAAGGAGTTATAATTG

>JAP07544.C|Supercontig_0001825:29307-29483

TAGTCAATGAAATAGTGTGTTATCTTCGTTGTTTTCCCATCAACATCCGTTTCCAACTGTAATTTCGATTTAATAAATTGGTCACATTTTAGTGTGTGCAGATATTGTCTTAGTTGTTTTCTCTCAAAACGAAGTCTTTCACATAGGTCATCTTCTTTCATAATTGTATTACGTACC

>JAP07552.C|Supercontig_0000008:1620497-1620776

GTAGACAAAACAACAGGACATCCACGCCATGTACATGGAAACTTTAAGTTACCATGCACTAAACGCATATGAGTATCTAAATTTCTTTTAGCTGTAAATGCAACAAGACCATCTTTTCCTTGTGAGTTGGAATTATCTTTATCGTCCGTAACACCTGGCGAGCATAAAGGACAAATAAAACGTCGTCTTACAGACGGTCCACCAGGGGTATGTGTAGATTCGTGTTCTGCTAGTGCAACGGGTCTTGTAAAACGCATATTACACGTAGGACATTGCAAAG

>JAP07563.C|Supercontig_0000188:499046-499196

GGATTCTTATTTACAAATCCTTTGAAGCACTTAGTTGGAGAGCATGGACTCCATGAAGACCATGGGCACCATTTACGTCGGACAAGTCTATTTCCATTATCATCAAAATCGTCCATTTCGGAATCTACTGAAGAGTTGATCATGAAAAATA

>JAP07564.C|Supercontig_0000131:791576-791671

GAAAGTGTTTCATGGACTCACTTAAGCCTACCATTTTTGGAACCCTCTAAAATCAAAGACATCAATGGTCGAAGGCCTGACCATCCAGAATATGAC

>JAP07574.C|Supercontig_0000064:815093-815356

ATCGTCAGTATAAGGTTATAGAATATGAAAAGGGTATTAAAGCTATCACCATTGTAAGACATTTGTTTGAAAATTGTTTGAATTCAGACAGTGGATTACCTCCTCATATTGGACGTCGTTTACTTTACACACATGATATCCCAATTATTCTTTGCAAACTACTTGAACAAAAACCATGGATAATAATTGGTTATGACGAATCTAATAAACAACGTCGTCAACATATTTGGCATGAGAACGGGTCATGGATACCAGATGACAAAA

>JAP07587.C|Supercontig_0000420:66166-66265

AATGGTTGGACGGTATTATTTTGTTATTTGTGGGCCTCATGATGAGGCATTATTTGATTTAGATTACAATTCACCAGGAAAATCAACAGGAGAAAAGGTA

>JAP07593.C|Supercontig_0000093:728578-728678

TCTAATGTACCTAATTCTTATCCAACGTATGAAGAAGCTTGGGATTTAAAATTAGCTCGTCAACTTGGTATCGGTGTATCACGTCTGCCATCAATTATTCC

>JAP07596.C|Supercontig_0000103:590212-590390

ACCAAAATCAGCTGGACTATTTGAAATCAATCGCAATGAAGCAAGATCAGAGGCCTCCTGGTCATCTTTCGAGAAGGTTATGTCACCATATGGAAACATATCTATTCAAGAAAGAAGATCAGTAAATCAATTATACTTGTCACCGATATTTCCGACTCATTGGGATCAAAACATTAATA

>JAP07610.C|Supercontig_0000028:334600-334856

ATGCAAGTAGAGTATAAATTCGACGTGGATTATGCCAAAAGTAACAGATCCAAGTGCAATAAATGCAAAGTTGAAATCAACCAAAATTCTCTGCGTATCGCCATCTTAGTACAAGCTCCAAATTTCGATGGGAAAATTCCTAGGTGGTTTCATTACGATTGTTTCTGGAAATCGAAAGCGCATGTTGAGAGTACTGCTGAGATCAAAAATTTCGATTCCATTCGTTGGGAAGATCAGGAGAAAATTAGAGCTGCAAT

>JAP07621.C|Supercontig_0000126:670078-670191

TGTACGTCGTGTTGGACTACTAGAAAAAATTGAACTGAATGGTCTATTGTAGCCAGACAAAAGAGGATTAGAACCATCGGAAAATGAATCGTTTGACAAAGAATTTACTACCGT

>JAP07630.C|Supercontig_0000259:83950-84057

TCCCTTTAACTCTATCTGCTTATATAATGTATGTGTGTAAAGGTCAAGCCTCCAAGTATGCTGGTGTAAAAAAAGTGTCATATTTGACTCTATGCACTAGTATGTCAT

>JAP07652.C|Supercontig_0002065:384340-384450

GTTGTAGTCCAGTTCGTATGTGGGCTGTTCAGTGGATATGAGAATACCGACAGCACAAAGAATATATCGGAATAGTAATCAATGTTTCTACTACGATTATTCGAAATTCGC

>JAP07686.C|Supercontig_0000383:2237-2381

TGGAGACCAAAGAAGATAACGTATAGTGAGACTAGCAACAAAGCCAGCATATGTTGTTTCCAAACCACTGTTTAAAAACACAGATAATAACAGCCAAAAATTTGACGGCCATTGAATTCTTGGCCAATGAGTTTCAAATACAGAA

>JAP07689.C|Supercontig_0000481:53523-53672

AAACATTTTAAATGTTTTAGAGATACTGAGATCTCATCGTTGTTATTTAACGGTTGCTTCTTGCCACGTCCCATTTTTGATCCATTGACAAAAGTCCCATTGCTGCTAAGGTCATGTATAAAAACCAGAGGATTGTCACTCATTGATTCC

>JAP07712.C|Supercontig_0000619:37577-37948

CTGATATAACCAAATGATTAGCGCTTTCTTCCAATGAGTAAACAGCTTCTAACTGTTCACAATCGATTATTGACATGTTATACGTTTTAACGATTCCGAATTTACAGAAGTACTGAATAGTCAAAACAGTATCCTGAGATGATAAGAACATCTTGCATTTAAGAACGGACCTCTCCAGTGCGGAAGTAAGTCTAAAGACGTTGGAACATGACTAGGGAAATTACAGAGAATAATACGCCATACACTACTAGGGATTTTAAAACGTTGCAGAGACCCGTTAGGTAAAGGGCTTGAGAACTTGTCGAAAAACACTTCTCTAAAGTGAACCGAAGCAAAAGAAGACCTCGAGGCGTTGACTGTTTTGACCTTTAA

>JAP07729.C|Supercontig_0000061:582956-583043

GTAGGCGGCAAAGGCAAATTCTGAGGTGATCCCCATGATGCAATAGGATCATATTCATCTAAACCGGGTGACATCATTTGATTACTAT

>JAP07739.C|Supercontig_0000486:212093-212185

TGGTGATATCCCTTTTGTCAATGATAAAAGTATTATCGGAGGGAAATTGCGATATCGTAGAGACAATATAAGTGAAGCAGCAAAACATCTTAT

>JAP07749.C|Supercontig_0000245:533349-533459

TCTTGACAACAATATCTTCCACGTATAGAAGTGTTAGACCGTTGTGAATTAAGTGAATTCGATAAATATTTTTTATGATTATTGAGCTTTGTAAAATTTATTCGTCTTTTA

>JAP07753.C|Supercontig_0000180:412266-412458

TCACAAGGGCTGCAAACATGAGAAGTTCTGAAACAATAACACTAAGTCGTTGGAAAATAATAAGTTCATTTGATATATACGGACGAGAGGTGATGGTGCAGATTTCGGGATCAATTTTGATAGCTATAAATGATAAAAACCATTCGAAAAACGCAAACAGTGGAGGATAATCCAGTGTCCATATGGAAGTTTC

>JAP07767.C|Supercontig_0000674:399004-399204

CGGTTCGGGTATGCCAGCTAATGTCCATGTACCTCCAAATGATCCTAACATTCAAATGCAGTCTGTTGGAAATAACTTTCAATTGAGTGGACAGCAACAACAACAACTAATGAATACTCAGATCATCTCTTTACCACGATCTGGACCTACTACAATCCAATCACATCCTCAAATTCTTCAAGTTATGTCACAAAATATGTC

>JAP07769.C|Supercontig_0000102:366490-366740

TATTCTTTTACAATTTTTAGTGAACGGGGTTCACAAGACGACACTTTCTCAATAGCTTGTTTTTCCACTGATTTTTTTAACAATTTTTCCATATGCTTCTTCATTTCATGAGCTTCTTTTAGTCTTTCTTTTTTTGGTTTTATAACGCGAGTCCCTTTGCGCATTGGTTTGATGGATTGTTTTTGTAAAGACCTTTCACTTTTACCGGATTTCACTCCTTTGGGTAATTTGGTTTTGACTTTAAGTTTTCC

>JAP07790.C|Supercontig_0000019:1795504-1795626

AACAGCAGGTATGTAGAAATCCTTTACCAGCTTCAAACGCCGGACGTAACCTTCTTCGGAACTAACTAGTTCCATAAGTGGTTGTTTTCGCTGTATTAACGCTCGTGAAACATCATCCTCAGC

>JAP07792.C|Supercontig_0000488:262612-263048

ACTTGATCCAGATCAAAAAAGTAATCCATATCAGGATTTAGATAACGAGCCATGACATATTTTTGTTTTTGTTCAGATAACAAATCAAGTTTAGACATTACGTTTATATGAGCAGTTGATAAGGATACCATAGCAGAGAGAGCAGACAATACACCAGCTAGAAAATGTGATGAATCTACAAGGAATCTAGCATCCAAAATAAATATTGTAACAAAACGAAAATCCCATTTTCTTTGCATATATTCAATTATTCGTGGCATAATCGGTAAGTGTGAGTATAACTCGATTTGACCAGGACAATCAAATAATAAATAGTCTCCATCTATATCACCAAGTGCTGTATCTAACCAATTCAAATTTTGTTGAAGATATTCTAAGCAGAAAATAAGTCCGCCATTTGGACCTAAACGTATAGCCTCATCTTCCATTACATCATC

>JAP07793.C|Supercontig_0000023:822170-822340

TGGACGATGGGACTGGTTGTATAACCTGCACAATATGGCGTCGTGACTGTTCAAACCATTTTAATGTATTATCAATAACAAAAAATTCATTTGATTTTGAACAGCATTATTTATGTGAGCAACTCATCAAGTTATCATGCAAGTCACGACCTATTTCATCTTCAGCTAAAC

>JAP07794.C|Supercontig_0000305:272521-272584

CTTTGGATTTAGAAACGGAACAGTGGGAGTGGCTTTTAAACGTTCTATCTGATCCAAATTTATC

>JAP07808.C|Supercontig_0000000:2254967-2255276

AGACATATATGGAATATCAATCAGAAGGAGACCATTTAGTAATAACATGATTTTAGAGTTCTCAGCCATTACATTCTCAGGCACAGTCAGTTTATTTTGAACTTCACGACAAATAATTTCATTTTCTTTGCTAGTTTCAGTTTTTGCATTTATTATTATTTCCCTTTTAGAAGTCAGATATGCACTAAGTGAGTCCTGCTTAATTCCTTTCGCATTCAAAAAAATTTTAAGGAAGCAATGACAACCGGGGGTATTTACAGTGAAAATGGTGCACACGTGTTCCAGAATATTAGACATTTGTTCCATTGGT

>JAP07809.C|Supercontig_0000157:962886-963397

GCAAAAGTAACTGGAACGAAAAAGCCGCTTTGCAGCTACAAAACAAAACTCTAAAAACAGTCTCAGCTATACAAAATCTTATTCAAAATATTATAAAAACTTCACATTCTGCTGAACTTATTTCTTCCGCTATCAGCCACACTATCAATCAGGATCCTGTTATATCTTCAACATCTAATAAGGTCATTCAGCTTACTTGTACCATTGACCGTCTGTCCGATCAAGAGAATCTTCTTCGAACACGTTTGGACCTCCTTTAATAGTACTTTCTACATTTTTATCTATAAAATGCCCACTGCAACTTTACCAAACATTCTAATAACGGGCACACCTGGCACCGGTAAAACAACTATCTCTAAAGAAGTATCTAGACGTAGTTCACTGAATTATATCAGCATCAATGACGTTGCCAAGGAAGGAGAACTGTATGATGGATATGATGAAGCTAATCAGTGTCATATCCTGGATGAAGATAGAATCGTGGATGAATTAGAAGACGCTATGTCATCGGG

>JAP07852.C|Supercontig_0000383:6975-7189

CCTTGTAAGGAAATTGTTACAAATAAACCTTGAAGAAAAAACACTGCCAGCATCCACCAAAGATGTTTAACGAAAATAATAAGACCATTGGATATCACTAGGCCGAAAAGTCCAACACTTAGTAAATCACTTGGTTTAGTAGAACCTTCTGAATCCAATAATAAACAGCTAACTATCCATCCACCAATACACCCGACGGCCTTCGATGTGAAAAC

>JAP07857.C|Supercontig_0000046:123526-123668

ATGATTTTGGAAAGAGCACACGAAGCGGCCATATTTGGTCATTCTTCCGTTCCTTTACCTTCCATTCTCGGTACAGATCCAGCTGAAGGCAAAGATAAAATAGTTTCTTCTGTGGATACAGTGAAGCAACTACTTCATCATTC

>JAP07860.C|Supercontig_0000042:481572-481793

TGATTTTGATAAGGCTTTTGATTTTGGTGCAATTTGATGACCTGATTCCAAAATCGAATAATGTAATTTATAAGCTAGTCTAGAAAGCCACAGACCTGATGCTAAACGACAAAGTAGATAATGTGCTTCTATAGGGCATGCATTAGCAGTTGAAAATAAATAATGTTGTAACCATTCCGTGAGATCTTCACGAACAACACACATAGATTCGTCAAGTATCTG

>JAP07867.C|Supercontig_0000362:284971-285328

GATGAGATAGGAGTTAAGTAACTGTCAGAATGTTGTAATGTCCATAATGAATTATTTGTAAAATAATTAGAATAAATATTATATGTATGTTGATCAGATAAAGATGTACCACGACAATGAGTTGAAAATTGTGAAGTCGGTGATTTGATTGCAGATAATGAAAGGAAATTGGCTAGTTGAGAATTTGAAGCACATAAATTAGCATTGGTATTGAACTGATTCACAATTCCAGTATCACTGCATAGATCATAAGCAACGGCTAAAGCTGTTCCTAAACCAAGACTTCCAACATGTACTTCAGTTTTATCATTAACAGAAACTGATTCCAAACAAGTGGAAGATTGTCGTGGATTATCAG

>JAP07879.C|Supercontig_0000002:12231-12396

GTATTAATGAAGCTTGACATGAATTCGTTGAATCTGTTGGTACAGTTATTGTATGTTGATGACTTACTGTTTTTATACCACGATTACCACTAGTTCTGTATGTTGCTTCAGTAATATAACTTTTTAATGAACCACGTAATATAGAATAACTTGGAATACTATTACG

>JAP07882.C|Supercontig_0000195:1491067-1491293

TCAGACATTAATCGTCTTCGAGCTGAGAGACTGCAACGAGAACGAAAGGAAAGAGACAGGGCAGCAGTTTTACTGGCTAAATCTTTTGGTTTAGATGCTTTAAAACCTCCAGATCAAGAACAATCGTATGTGGACGAACGAAGTCTGCCCTTTAATTCTGCATTTAATCCCGAACTGTCAGCTATCCTGGCTGAACGACGCCAACGACATCGTGAGTCTAGAAAGCG

>JAP07883.C|Supercontig_0000046:1315013-1315159

ATGATGGCTAAATGTAGCAATTGGAGGTAAACATGCTTCTGAATCTTCTTGAATAGCTGGTAATTTTTGGGATTCATTTTTTAATGCAAATTGTTTACGACATGATAAATCTTGGGGGAGCCCAGGTGAAGCTGTGTATATATCCCT

>JAP07924.C|Supercontig_0000082:204548-204700

AGTATCACCAACTGTGGTTCTCAAGTTAATTCGATCAATTATAAATGAACTAAATGCTTTGTTCACCGGTGAAATCATCCCTGTAGCACCAAAAGCTCAGAAAAAAGTTCCTATTCCCGAAGGGTTAGATTTAGACGCTTGGATTAATCCACC

>JAP07927.C|Supercontig_0000365:276707-276912

AGATGCCTCGTATATGGATAGATTATTTAATGTTCCTAATGTCACAAGGATTGATTACTCGAACAAGACATGCTTTTGACAGGGCGTTGAAAGCTTTACCAATTACCCAACATGATAGGATATGGAATTTGTATTTAAGATTCGCTGATAGACACGGTCATAAAATTAACGAGACCTGTGTACGAATATACAGACGATATGTAAAG

>JAP07944.C|Supercontig_0000306:331709-331838

CTCAATGGTATCTACTCAAAGATTGTATGATTGAAGAACTAATGATTGATGAGTTAGTAGCGTCAATATGGATTAGAATGCGAAATAGGCTGTTACAGATTCTTGTGGATATTGGACATTCGATGTTTGA

>JAP07950.C|Supercontig_0000379:139781-139946

CAGTTGAATAAGAACATTTGTAATTAACGTACTTATATCGTGTCCCTTTATCAGTGTGCATTTTTTCGAAATCGCTCTTCACGTAAGTAATGCGCGTAATCTCTTGAGCATAAAATAGTGCTTTCTCGAAATCAGCCCATTGATATAAAGGCCGTGACGTCATTAC

>JAP07956.C|Supercontig_0000196:365707-366026

ATTTTGATTGCTGCTAGACCACATCCGAAATTGGCTGGTTTGCTTGCTCGTTTAATCGGTTTATGGAGACCACATCAACTACCTACAATTCAGCCTAATGTATGCGAATCTGATTTACATAATTGGACACATTTAATATCAGCATCTATTCAAGAGCAAAATTATGAAGACGGAATTTGTTTCCGTTTATGTGAAGCTTCTATCACTGCGCTTACTAATTATGCTTCATTATATGGTGGTGAATATTCACGTAGTGCATTGATACCTTCATCAACTGATCCTTTGTCATTAGGTTTATTAAGATTAGCTGAGGAAGATCA

>JAP07960.C|Supercontig_0000009:937108-937288

GTATCACCAAATATACGTATTGACAATTGTGGTATAAAGTAAGTTTTACAAAATGGACAAAATGTTCTATGATCATTTTCATCTGTAGACCAACCAGCCATTATCTCTTCATCATAAACATAATGTTTACACTTTGGACATGTTGAACAAGTTGTAATGAATACATTCAAATGAGTAATAC

>JAP07977.C|Supercontig_0000479:424604-424708

TGAGTATCAATGAAGCTTTTAAACTCCATCAAGTCTTGCAATCCAAGCTCTATGCGAGTGAGTGGACGTCTCTGCATTAATGGAGGATTTTAGATGTGACTGAAA

>JAP07978.C|Supercontig_0000124:1570282-1570476

TATATTATGTAATGTTAAATTGACATTTTGCCATGTATATGATTCATTAAAATGATATAATTGTAATGTAACTAATCCTTTCGGTTGAACGTCAACTGATTTACCCCAAAAACGTAATTTAAATTGTATAGTAGCACGTAAACTATAATTATCTGATTCAACATGAAAAGCTGTGACTGGTGGATGATGAGATAC

>JAP07980.C|Supercontig_0000141:305819-306124

AAAATGGATTCGTCAGACGAAGGTTCATCTCTTCCGGTAACCCAAATAGACGCACCAGCTTGTGTCCATAAACACGAACCTACTGCTGAAGAAAAAGAGCTTACGAGACTTCTGATTGAACGTGTCCTCCCTCCATTTTCAAAATCACTAAAACTACAAGCTCTTGTGGTTTATCAAGCTGATCAAGAAAAGATGAAGTGTTTACTTGTTAACCAAACTTATGAAAAAAATGATCATTCTTTTGAAAACGACAGTCCTAATCAAGACTTATCGGATTCAGGAATTATGGATACATCTACAGAGTCG

>JAP07994.C|Supercontig_0000058:1648555-1648764

CTTCTAACGTCAGCCAAACTACTCAAACTTTGCAAAAATGAACTTGAACCACCTTTACGGAACTCGAATGGGCCATAAGGTTTTTGTGATTTTAAGGATAAATACACTCCACGACAAGGAAAGCATGGGGCCTTTCGTATAACAAGAAAATCCATATCCCTAATATCGATTTCATAACCTTTTCTATCTTCTTGGGATAATGACGAGTCT

>JAP08018.C|Supercontig_0000023:812597-812777

CTGTCATATATATTAGGATATTACTGTGCTCGTCAGTATTGAGGCGCTTAGAAGTAGGTGTCGAAGGAGGTAAACGCCCAGTTAATACACGAATGAAGTTTTCTACAGTAACTTCGTAACCACGATAATCAATCTCTATCTCTTCGCCATATAAATTTACACGACTGTAAGGGTTGTTGAA

>JAP08022.C|Supercontig_0000056:789642-789914

TCATTCTCTGTTGGAGCACGATATTCCGATTTAGCACGAACAAACTCTCGAGCTACATCATCTGTATTAACACGTTGTGAAAGTACACGTAATACTGTCCATCCAGTTTCTGTAAAATGTAAACGTCGTCCTAATGGAAGTCGAATTGGAGTTGCACCAAGATTTAATGCTCTAGATAAAGATAATACTGTAACACCAACAAGACGATCAGTTCGTCCAAAACAATAATCTTTTACAGATAAATGTAATTCATACCATTCAGGATCACTTTGA

>JAP08029.C|Supercontig_0000169:470637-470908

CTTTCCAAGAACAAAACAACTAAATAGGTCATCCGTTCCAAATTCGCTTTTGCAGTAGGCGTACCCCAGGGTGAGTAATTGCTGGGGAGAGTTAAACTTGTTAGATGGCGTTGAAGAGATCGAGAAAGCTCTTTGCTATGTCTGACGCGACCGACACAAGAACTATTTTCCTCACAAACAGAAGAAGCAGACGAGTTAGATCCTTGGTGTACTGGTAAAGGAGTAGTCAAACCAGAATCGATCATCACCCGTAGATTTTCGACTGACATATT

>JAP08040.C|Supercontig_0000417:47029-47388

AAGTTTAATTTACAAAATGGTGAAATGATTGGTAGTCTGGATGATGAATATATCAATGGTTATTCAACAGAAGTTAGTAATACAGGAACTGCATCGAGAATGCGCAAATATATTACATCTAATTATAATAATGGTAATGGAGTCGGTGGAACTAGACCGTTAGTTCCACGACCACATTTCTCAGATTCTTTACATTCTGCAGTTAACTATCATCAAAATTCATATGCAAATGAAAGTAATTCTGTGACAGTCAATAATAGATCTTTACATAATTCAAATGATACTACAACTCTTAATGGAATATTGTCATCATCATCGAAAATTGATCATTTATGTACTACACTTACATCAGAACGTATG

>JAP08062.C|Supercontig_0000011:1401816-1402063

CAAAGATCATTACCCCATGTTAAGCGTATTGCATTTCTGCGTAAAAAATTTGATGGAGCAGATTTGATGAGAATTATTAGTTCTGGAGCTTCTCCTGTTGTTAAGAGAATGTTGTTACACTTTTGATGTTCACTTATAAGTATTGGAAATTTTGGATCATTTATTGGACTAGATGTTACAGTCAAGTTTGAAAGAACCTGTCCAACTAGTTTTGGCATATCTACCACCATTGGATACTCCAAATCATC

>JAP08071.C|Supercontig_0000292:46082-46263

TCAAGTTGGCAAAATCTAACTGCAATGAATTGTAGCGCTGTTCCTTATGGAGCTGTTATCTGTTCTGACCAGTCAGGTTTTGTGCACCTTATAGATCCCCGAGCTGTATGTTCTGGAAGAACAGGGAGTTTACGTTTTCATAGTGGTGCGAATTTAGCTTCATTTCTATTAAACAAAGAACG

>JAP08072.C|Supercontig_0000641:280221-280749

AATATTTTGATAAGGGTTTTGAAAGACACTCCATAAAGTCACCTTGTAAAACTGGAGAGCCAAAATTATTATCAATTTGAGTCAATTTAACTCTTATTTTGTCAAAAGGTTTTAACATGTACTGGCTGAATTCTTCTTTTTTTACTGACTCAGAAGACAAGCGTGGAATACTAATCATTACATTGGAAATTTCTGTACGGCAAAGAATAAATTGTGGTCTAACCACTGAAACAATAGCATCTACTTCCAAACCCAAATGTGGGCGAAAAACATTGACTTTTATCTTTCCGTGTACACGAAGATGGTTTAACTCTGGATGAATCAGGAAAAGACCACAAGTGAAACCATTAGGGACATAACGATCAGAATAATCAACTTCAGTTATGATTTGTAAACTTTTGGCATCAAAATCCACAAGGACACCTTCTAGATCAGGGATGAACTTATTGACACACGAAATAACATATTGAAGTAAAGAACGGGTCAACTGAGTGAAATTATTGGGATAAATTGGGACACAAAATGACCA

>JAP08077.C|Supercontig_0014216:968-1240

CTGATATACAGCTCTAGCTAAACTAACACGTTGTTTTTGACCCCCGGACAAATTGACACCTTTCTCACCAATTTCTGTCTTATCACCAGCGGGAAGATGAGCAATATCTGTTTCAAGAGCGCATGCAGATATAACATTTTTATACCATAATTCTTCAATTTGCTCTGCAACTGGATTCGACTGAGTAGGATCGCTGGATGTGATAAAACGAATATTTTCTCGTAGAGATTGTTGTTGTATCCAAGCTGTTTGTGAAACATAAGCAATGGAACC

>JAP08097.C|Supercontig_0016744:1072-1179

GCAACAATTTCATACAGTCATATACTCCTCGTGCATTTGAATCTGGTGGTGAAAATAGAATTTTGGTAAGCCAATTATCGGAAGACAAACGGTCAGCCTTGATGAAAG

>JAP08111.C|Supercontig_0000131:1130842-1130919

TCTGATATCAAGTCTAAATAAGTAGATGGGGAATATTTCGTAGTCCATAACTGTGCGAAACCAGATTTGCTGTGTTTT

>JAP08120.C|Supercontig_0000199:121539-121738

CGATCATGTCTTAAACGAATGCCACGAACATCTGTAGCACAGGTGATTTCTAAGACAAATTGTTGCTTTATTCCATCCCATAAACAAGCGACATTACTTGGTAGACGTGAATGATTACCGCCACCCAGTATACCAAGTAAATTTGTACGAAATAACATTTCCATGTAGCCGACACCGGTACCATCTCGTATATCGAATTC

>JAP08139.C|Supercontig_0000442:100716-100827

GATGATTGTTCTGATGGAGATTTCGGTAATGGTGGTAGTAGATACTTGACAGCTGGTCTTATTAATGGCCAAAATACATACCATAATCCAATGAAATAGACTGATTTCAATA

>JAP08163.C|Supercontig_0000453:305669-305896

AGAATATGGATTCGTTTCAAGGAGTTAAAAGATCAAAGATTAGAAGAAGATGAATCCTTTCATGATTATAAACTAGTGGATAAATTAAATGAAAATCCAACTAATGTATTCATTAATTATACACCAAAACGAAGAGTTTATTGGGATCATTTATTTCCATCTGGTTATATGCCATGTGGATGTCTATTACCTGAACCATTTAGTTTTAATAATATAAAACAATCTCAT

>JAP08180.C|Supercontig_0000643:92948-93136

GATGTGATGCGCCATATAACTCGTAAAGTTGTAAGAGAAGCACGTTTGAAAGAAATCAAGATTGAACTACTCAATTCTGAACGTTTGAAAGGTTACTTTCAAGATCATATACCCGATTTAGAAGCGCTTCGTCATGACAAACCACTCAAACATGTTGCTCAACCACATCTCAAAGATGTACCAGATTAT

>JAP08189.C|Supercontig_0000330:121746-121851

ATTAAAGGGATTTTTGTCTGACTCGACCAAAGATAAACACTAGTACACGATCTATCAAAAATGAAGCTATGAAATCGAATACCAATACTTTGAAAAAGAATATACG

>JAP08193.C|Supercontig_0000055:325612-325868

TAATGGTTTGTTCACCTGGATCCCAAAGATCTGTAAGCCGAAGTACAGCCCGCACTCATAGCGCAAGCCGAAGTCGATCTAGAAGCGGCAGTATTCGACGGAGTTATACACACTCGAACCGTAGCCGATCAGCAACTAGATCATTTTCTAGACGCAGTTCCCATTCACGTCGGAGCACTAGGTCCAAAAGTCATTCCAACTATTCTCGTTCACGTTCTGGCAGTCGACGTTCCAGATCATGGTCTAGATCACGTTCA

>JAP08205.C|Supercontig_0000103:560862-560965

CTGACTACCATCTTTAGCTGATACATCAGCTAGATTATTATTGATCGCTTGATGTTGAACAATAGCTGCACGCGTTGCACTACCAGCGACACCAACTAAAGACT

>JAP08210.C|Supercontig_0000003:830477-830784

CCCCATGAAACAGTTACAATTTGTGGTGAAATACATTTCTTCATTTTTAAAGGAAACCACTTTTTGAATTCCGAGAATATTACACCTTCCAAAAGATAACGAGTAAACCATCGGACACTCCATGTTAATGATTCCTTGAAGCCTAATGATTGAGCCGCAGCAACACTATTTATAGGAACAAGAACCGTACTTGGCAGATCCAAAAACTGATTATCAAACAGTGGTGTACTAGAATCAGGTACAACACCCAACCCGATATACGTAATAGATTTCGCACCTGTAACAATAATATCACGTTCAGAATCATA

>JAP08211.C|Supercontig_0000040:188768-188919

AGTGCAATGACTGTATGGGGTGAAGAGAATGAAACAAATGCATATTCAAATTTACTCAATTTATATCCTGATGGAACATTCGCCTGTGTATCCGATAGTTATGATATATGGAATGCATGCAGTAATATATGGGGTGAGGCATTACGTGATAA

>JAP08255.C|Supercontig_0000238:88154-88284

GTGTGCGTCGCTTTATTCATAACTTTTTACCATCAGCTATGGATTTAAAAGTATTTGGCGAACCGGTCCCACCTTACGAACATCCTCAACTGACCATATGTGACGAAAGTTGCATTAACATTTTAAAACGT

>JAP08263.C|Supercontig_0000035:47475-47566

CTGAGAGCTGTAAAAGTATTCATTTCTTAAAACTTTTCTCAGAGATGATAAAATCTGTATCAAATGCCTTCTTTCACACAAAACCCACGTAT

>JAP08264.C|Supercontig_0000215:312142-312339

GTATATCTGTCTCATCAATGGAACGTTTGGGCTGAATGGTCGACAGTTTGGCTGCACTAGCAATACCCGTTTTCCTGACAGCTTGGGCGACGGATTCCTGTAAGATTTGAAGTTGGAACTTGGTTCGCATGCGCTGAGCCATTTTGACGAACTTTCCAGGTTCATGGAAACTCAGCTGTCGTTTTGGACGTGAAGCAG

>JAP08290.C|Supercontig_0001521:84435-84587

TTTAAACATGGTTCACGTGTTGCTCGACAAGTGATTGGTGGTTTAACTGATTTACTAAGAAATCATGCTGCTGATCTACCAGATCCAACTACTGATTGGAAACTTATTTTTGGTTTATTAGAAATATGTGGAGCTGGTCGAAGAGCTAATATC

>JAP08319.C|Supercontig_0000463:229815-230033

ACCATGAAAGTCGGTTAATATAACACGTTTGTCGTAACTAGCTGATAGTAGATAATAAGCAGTTGGTGAGAATCGTACACTACGCACTTCATTTGAATGTGGTCTATACGCATTGATATAACGAGCACCACGTAAATCAAATAATGATATTGTTGAATCTTCATGTCCACTAGCCAGTAAATTACAATTCGGTTCAACAGTGACTGAAGCAAATGCACT

>JAP08320.C|Supercontig_0000156:1374418-1374597

CCTAAATGTCTATAAAGTTGACCGAGAATTTTAGCTGGATAGATATTTATTCCAGCTTCATTATAAATTTCTTCAAATGTTTGTACTTCAACTTGATATTCCATTAACTGTTGTTTAATCTCGTTATCTTCAGCTACAAGTACAACTTGCACTGATAGATCTGGTTTCGGTTCAGTAACC

>JAP08321.C|Supercontig_0000183:116999-117245

CTCAGATGAAGTAAAGGGATTTGTGCCCGATGATCACATACATAGTCCCATCCTAAAGCAACTCGCGCAGCTGACAAAAATGACGAGACATCCGATTCAAAATTTCCTCCCAGACTCAGAAGTACATGAAAGTCGATTTCAAAATCTTTGATGAAAAGTGGATCATTTAAGCGGGATGATATCCTGAAAAAATTACAAGACCTAAAATTACCATTCAGCTGCCAGACGAACTGAACGAAAGCCAGTA

>JAP08330.C|Supercontig_0000308:77570-77787

AAAAGGTATGGTATCACTCTAATTGCTCTACACGTGGTCGTGGCTGTTATAATACTCGTTTGTTAGATCAAGGCGGTTGTTGTATTTGGTATAACGAGCCCAATTTATTAAACGAAATTGAAGAACACTTAGGTGTGACTATCGATACAGTGGACAAAAATTTAACTATACCTGTAGATGCATTTGATGGAAAAGTTGTATATGGTCAGAAATTGAAA

>JAP08336.C|Supercontig_0000071:668936-669049

TATATTCGTTCTGGTGAAGTGTATAAACCTTTAGAAGTTGTTGCACAATCTTCTCTAAAAAATGATAAAGAACGTGCAAGTTTACGAAAGGCGCTTGAAGAAATTATCGATCAA

>JAP08353.C|Supercontig_0002065:604389-604951

AACGCCTTCAACTTGTCAAGTGGTGCATACTTTTGGCTATATAACTCCCAGTGCAAAGGTCCAAAGGCCTCTCCATTAATAAGCTTAACAAACACCATGATTTTGAATAATTCGGGTGAAGATGATCAATCAAGTTTGACAGAAAATTGTCCTTCAAATGTCTATCAAGACGATTTCGTCTCTTCTAGTGCTTCTTCTCGTGCTATTAATCCAATAATACATTATCCTATATCCCCATTATCCTTGCCAGGATACCCATTTGTCGTTTTTGAAGATCCTGTTCAAAGAAGATATGACCTAGTTCACTGCTCGAAATTGCGTCGAGGTGATGGTAATGATGTAACTCCAAATTTAATGCGTTTACGTTCTATGGGAGAAGAACTTGCTCACTTGAATAAAAATATTACAGCTCAAGGTGAGTTGATTGCAGCCGGTGCAAGTTCAGTGCTTGATCAGTCAGATAATAATGTGGATTTTAAGATCATTCAGGATTTTTCTGGGAGTATCTTCAATTCCAAAATAGTTGAACAGGCGAAACGCGAAAAAAACAAGTTGGCTAGCAA

>JAP08359.C|Supercontig_0000306:65719-65809

TAGCAACACAAGTGGCTATATTGGCAATTATGTCGTCAAAATGTGTTACTGTTCGAACTACTACAACAACGAGAACAATTCGAGAAAGTCG

>JAP08361.C|Supercontig_0000227:527080-527362

TTATTACGTAGACTAGTTGATGAATATCTTGACAGGAAAATCACTTACTCACACCAACCAAAGTATATACTGGGTGTTCTGAAATGTGTACTGGAGAGGAATTTCCCTGAATTCAGTGATTCATTTCATCGTGAAAGAATATGCGCTTATCTGAAAGCATGCAGACGTAACGCCAAAAGAAAGAATGGTGAACCGTATGTGAGAATGAGTGCACGTTATCTAAGTTCTGGAAAGGCGTCCAGTTTGGCTGAAACAATTTACCTGAAAGAGCACAGTTATTTAA

>JAP08384.C|Supercontig_0000126:594168-594291

ATTCTCAACATGCAGCAACGTATCATGGGAAACGCCAAGAATAACGCCTTGAGTACTAATCTCAGTCTCAGAAGATTCATTTCCACCACGCAATAAAACACTACAACCAGAACTTGATGACGAG

>JAP08386.C|Supercontig_0000613:71701-71826

TTTCGATACGAAGTTTCTGATCGATCATATTTCACTTGTTTAAATCCACAGGTTTCAAGCTTTTCAATAACATCAGCCAGTTTAGTTTTCAAGAAATTGACACGAGCATAACATGGAAGTAATCCT

>JAP08402.C|Supercontig_0000045:347847-348169

ATGTCGTGTGATGTCTGGCAACAGGTCCTGGCTTTGGACTCTCGCTATAGCGCTGTCAGGCCAATAACAAAATCAAGCACTGAATTAAGACAACTTTATCTTCGAAGAAGGAATCAATTAACAAGAGAAAAAGCCGCACTGAGTGCCAACGAGGCTTTCAGACATCCTAATTTATCTTGTAGTTTTCAATATACAGCTGAAACTTTTGTCACATCTTTTGTTGCCTACAGGAACATTCGTGAGCAGCTATTACTTTCTCTTTACGGTCCACCCAAACCTCACAAGTGCGCGTCACCTCAATTCAGCTTAACCAAAACCAGGAG

>JAP08413.C|Supercontig_0002065:403533-403735

TTCATCAAATTTGGATTCAGGATTAGAACAATCAGTTATGACATGTTCTGAATCATATGCGCATATGCTTTCACCAATGTCAGAAAAACATTTAGAAAATTCTCTTGGTCACCACTTTTTATATAATAGATATTTATCAAATTCTCAAGTTTTTAAAAATTACTCACATTCACTTTCAAAGTATTCACGTTTTAAAAAACGAT

>JAP08441.C|Supercontig_0000382:406812-406876

AAATCTGTATCATTTGCTGATGAAGTTGGAAAATCCCTAACTGAAATATTTACACTATGCGATGA

>JAP08447.C|Supercontig_0000304:510064-510174

TACGACTGAATGGAAATCGCTGTGTTAATTCAGCTCAACCATCTATTAATAATCCAACTTTTGGAGCGTTTCCATCCCATCAACTTCATAAGATACATCAGCAAAAATCTC

>JAP08451.C|Supercontig_0000417:164088-164198

CAGTGGTCAAACTTTGTTCATGATTTTGACAAGAAGCAATGCAATTTCCGGTTGGCGGCAACTTATGGGACCAACAGATCCAAACAAAGCATCTGACGAATCATCAGAAAG

>JAP08462.C|Supercontig_0000169:70710-70880

TTCTAATTTTTCACCTTTACGCGGTAATCGTGTAGTTTTAATTGATGGTCGATTAAAACGATTTCCAATACGTATTGAACCTGTCATAGATTGACGTTCTTTATAACAACGAAATATATCATATTTTAAACGTTTTGAATCTGCTCGAAAATAAATATCTGTGATATATGA

>JAP08473.C|Supercontig_0000294:109232-109566

TTACACTTGAGTAAGTGACGTTTCCTATGACTAGGGTTATGAAAAACAGAATTACAATATGGACAACCATAACCTCGAGTACCAGTATGTAATCTTACATGTAAGCGTAGACTGCTTCTAGAGGAAAATCCGGAACCGCATAAATGACAGAAAAGTGCATGCTTCATTAATGCCTTCTTTTTAGAAGTTAGAACTATTTTAGACATATTTGATTCGTGTACTTTAAATTTGTGTTGACGAAGTTGAGAATGTTTCATAAAACGTCGATGGCATTTATCACATTCATATGGTCGAATTCCAAAATGTACATTGAGGTGATATCGAAGATCTGATTT

>JAP08477.C|Supercontig_0000066:178960-179041

ATTATCTCACGGATTCGGAGTAATAAAAATACATGTTGGGGTGCCTGTAAGTAAACTAGTGGGAGAAATACCATCAGCTGTA

>JAP08482.C|Supercontig_0000130:1702483-1702646

TGATGACTATGAAAACGTATAAATGTCATAATGAATCCAATTATTATACCCAATAGTATAGGACCAAATGCTATTAACCAACCGAGTAGAATAAAATTATTCTGAATTGGATCATTTAATGGTGCTCGACTGTATGTACGACATTGAAAATCAGGTGGTTTTTG

>JAP08497.C|Supercontig_0000160:1189157-1189418

GCATACAAAAGTACAATTGATTGGGATTCAGGTGAGAAAGAAAAATCTATCTCATGTGTACATACACAACTCAATCCAAATAAATATCAGAATCATAGTTATTCGTTGACTAGTTTATTAGATCAGCAACCAAGAGATTGGCTTATTGATGATTATGATTCAAAACACAAAGGATGGAGACGGACAGATCAGATCGTAAGAAATAAAGAATCATTTACAAAAAATTCATTATATCGTGAACAGTTCCCACCAAAGGAAGCTG

>JAP08510.C|Supercontig_0000356:118005-118251

TGACGATCGGTTATGTGCTATATGCTCAGAAAATGAGTTCAAATACAAGTGTCCGAAATGTGAAGTAAAAACTTGTAGTCTTTCATGCTGTAATGATCATAAAGCACAGTTCGATTGTACGGGCATCTGTGATACTATTACCTATTGTCGGAAAGAGAATTATTCAACGTTTCTTTTTCAGAAAGACTATAGGTTACTAGAGGAAATAGACAGAAGAAATGCTTATAGAGAAAAACAACTTTTATCG

>JAP08514.C|Supercontig_0000144:200379-200531

GCTAATACAGCAAAAGCATCACGTTGTAAACGACGTCGTTCAACTCTGGGTAGTGGAAGTTTATCTAACATAATGAATAATTTTTGAAATCTATTATTTTTCATCATATTTGGAACAATCATTTGTGATGAATTACGCATTCTACTAGATTTG

>JAP08525.C|Supercontig_0019088:295180-295322

TGTATTTATAGCTGAACACTGTTACATAAAAATTAAAGTAATAAATATACATACAAAACAAAACTGGAGCATACACAGCAAAATGAGTTGATTATCACCCAAACGATAGCTACTTAAAGAGAAATGCAATAATTTCTGTATAT

>JAP08535.C|Supercontig_0000177:603600-603766

AGGAAGAATTAGAACAAGCTATCAAAGAAAGTGAAACAAGACTTATGATACTGGATTTTTTTGCGGATTGGTGTGGGCCTTGCAAAAGAGTCGCCCCGGAACTGGACAAAATTTGTGAAGAATGGGAAGATGTTTTATTCGTAAAATTAAATGTAGATGAGTTAGAG

>JAP08541.C|Supercontig_0000276:213412-213612

GATAATGGATAAACATGCATTAGGATTTGATGTATTTCTTGGACGGCATAGTTTTACAGAAGCAGAAATTGCCAATCGTTGTCAACAAGAAGTAGTACTCTATGGAAGAGATACAAAAGCTGAACGATTTCAAAAAATGAAAGGTTCATTATTCATTGAATTTTACAGTGTTGGTCAAGAAGACTTTATGAATATATAATA

>JAP08545.C|Supercontig_0000064:620711-620975

TTACCTGGCTGTAAAAGACGTTCTTGAGTGGAAACAACTAAATAATGATTGATTGGATGTATATAGATTCGTAGAAACACTAATTGATCATTAACACTTTGAACTCCTCTTACAAGTGTTAATAAATGTACAGCTTGTGGTTGTTGAAATGGTTGTTTCATAGAATCAATATCATAAGAATTAGTTGTTACATTATCAAATCGTGATACAGAAACCCATGCAGAATATGTAAATCCACATGATGGAGGAAATGAGCGTTCTCCTG

>JAP08553.C|Supercontig_0000074:548627-548921

AAATTTAATTCAAACGACATTGGATCATACATTGAAAGATGTTAACTACGAACCATTACAAGCACGTGTTTTATCAATGAATTTGGCAAATATGTTAAGAAAAAATGTACGTGAATTAAACACGCCATCAAGGTATAAATTTGTCGTACAAGTTCATATTGGTTCACCGGAACATAATAGTATATTTATTGGTAGTCAATCTATATGGAATATTGAAATGGGTGATACTTATGCATCGGCCATATTTTCAAATTCAAAGATATTTGCTGTTGGCATTATACATGCTGTATATTTT

>JAP08581.C|Supercontig_0000644:129669-129920

TACTACCTGTGGATCATCTTTTAACATAAAAGAATCACCACAAAAAAACTGTGAACTTTCACAAGCAGCTGTAAACTTAATTAGTGGTCTCTTATCTTATAATCAAACAGACAGACTTAAAGTTGCAAATCAAATAAAGTCAAGTGATTTTTTAATACCTGTTGGTGATTGGAATAATTTAGATGATTTAGAAATGCCTTTCATTCCTCATCCTGATAATTCAACAGATACATTTTATTTTGATGTAAGTAA

>JAP08582.C|Supercontig_0000685:957841-957937

TCAGCTAACATACCATCTTGAAATAATTCATCACCTGTAAGATGACCACGCCAACGTTTTGGTAACATATGATTCCACGACCAAAAATAACCTATAG

>JAP08591.C|Supercontig_0000107:485461-485643

TGTAAATGTCCTAATATTTGTTATAGTATTCTATGGTTTTTTATTTTAATATTTATTGCATGGCCATTATCATTCTTTATATCAATCATTTACTTAATATTAATTGTGTTCAGTGTTTGTTGCCCTGTTTTACAACCATTAACAGATGGTATAAGTAAAGTGATGATGTTTCCGGTAATGTGT

>JAP08597.C|Supercontig_0000011:275162-275342

TATATCTGTCCTCGATGTGGTATTAATTATTGTTCACTGACTTGCTATCGAAATGAAACTCACAAAGACTGCTCCGAATCATTTTATCGAGATTGTTGTATTGAATCACTGCGCTGTACCTTTTCTAAGGAGTCAGAAAGATTGCATATGAAAGAAGTATTAAGGCGTGAATCGTGTGCTT

>JAP08608.C|Supercontig_0000155:904809-904908

ATGAAACTACCTGGTTTCTTTGACATTATCAGTCCACCAATTGCAAATGCCACGTTGAAGCTGGCATCTGTATTTTTGAATACACCAGGCCGAAAAGCAC

>JAP08613.C|Supercontig_0000379:605895-606002

TTGTGAAGGTAGTTCAGCTAATACTTCTTTAGCTAATGCTACTTTACTCCAACGTATACTATCTGATCCATCAGCTGCTAATCGTAAAAATCGTCTCATTTGTACAAA

>JAP08614.C|Supercontig_0000491:177894-178004

ATTCCCATCAATACCACCGAAATGGTTTGTAGACAATGAGGAGGTTAGAAACATTAGTATAAATTTGTTGAACGATATTTACAAACTTTCAAGAAGAAGACGCATAAATAG

>JAP08615.C|Supercontig_0000626:69702-69887

ACAATTATGCTGTGGGAAAAGAAAGTACAATTATGCGAAGAAACTAAAAAATCCGTTGATTGTGATATAGGTCAAGGTGAAATGAATGTTATGCGTCACGAAATTCATCGTATGGAAGTTCGTAAATCCTCATTACTTCAACAACAAGAAAGATTAATTCAGGCACTTGAACGTTCTGTATCAAAG

>JAP08617.C|Supercontig_0000295:67135-67273

ACTTGGAGTCGGTGTAAATATTGGACATTCATGGTAACATCTATCACAAGCTCGAAATACTTGAATTGTATAACTCACTGTCAAAAATACTATAACTGTGACAATTACTCCAAGTGGTATACCCCATCTTAAACCTGGC

>JAP08618.C|Supercontig_0000304:995291-995571

AATGTACAACAAGCACTTATTGAAGATCCTGTACGTCGTCGAGCCTTATTTACAAGTATTATTGGTGGTCCACCGTTTGGTCAATTTTCACTTAGACGAATGCGTGGTTTACGTCTAGCGCCAAGCCGCATTAATGCTGGTGTGCTACCATACGGTGTAACCCGTAGGTTTTCAGTGAAATTGGTAAATTGGGGTCCGGAAACGGCATACTTTAGAATTAAACAGCTGCCAATTCACAGTGGGATACGTGTTTTTTATACGCCGGGACCGATTCCAGCAGG

>JAP08621.C|Supercontig_0000342:191951-192139

AAAGTATGAAGTGGCTAGTCGACATCTGTGCAATGATTTGTGGAGCGTCTAATATTAGATCACTACTCACTGAAATTGAATGTCTAGAGCGCTTGCTTAAGTGGCACGCAAAAGCTTCATGTTCACCCTCAATTTCAGGAAATGATCCACGTCAAGTTGACTATGCACTTTTGTCATTTTTTGATTTGA

>JAP08622.C|Supercontig_0000283:323631-323834

GACCAATTGCTAATTGAATCGGAGTATCACCGAAATGTTTCAATTGATTCAATCCATACTGTTGACGTCTTTCACGATTGTAATGCCAAGCACATAGTAGTTTTGCACAGGACATACGAAAATTATTATTTAATAATGAATATACCAGAGAATTTAGGCAGGCATTGAAATAGCCAAGCCATGTTGCAGCAAAAATAGCTTCTT

>JAP08652.C|Supercontig_0000225:70216-70415

TTTTCTTTCGGACTCGAACATTTAGATACGTTGACGGATGTTAATATATCCTCAGGATATTGAAGGGTTTTTTCCAAGTCTTGTATATAATCAATAACATGTTGCAATAATTCCAACTGGTTAACTTTCTTATTTCGTTCTATAGTTGGCACCATTCTCTTCAGTTTGACCAAACATCGTTTCATTTCAGTTGGTGGTGT

>JAP08657.C|Supercontig_0000064:1528712-1528946

CCTTCAGCTTTTGGATATTCTGATAATGAATTTTCTAATACTTCTATTAATTGACTACCAGTTATTTCAATAACTGATAATTTATCTAAATATGGTAGTATAGTAGTTAAATCACGTAAAGTGAATAATCCAGCTTTAAAAATTCGATCACCTCTTAAGGTACCAGAATTAATTAATACACAGTCGGCTTCTACAGCTGTTAATACTATATCACAAATGAAATTGCCAATATTTG

>JAP08668.C|Supercontig_0000379:495184-495404

GTCGTGACTGGAGAGAACGCTCTCTACTACACCTTGCAGTACTTTACCGTAGAACGGATTTTGTCCTTCAGTTGATTGAATTGTGTCAAGAGCTTGTCAATTATCAAGATTGTCTTGGACGTACCCCATTTCATTATGCAATCTGTCTACCTGACAACCGAAGATTGTTCTTAAAAATGGTTTCAAAATCTGGAGGTATTGATAAACAGATCAAGGATCTG

>JAP08685.C|Supercontig_0000009:642953-643084

TTACGATCAAGATGTATACCAATTGTTAATTCATAAAGAATTGCCCCTATCACTGCACCAATATATGGACCCAATATAGGTACCCAAAAGAAATAATTCCCTGAAGTAAATGCTGAACCACCCCAACCTATA

>JAP08703.C|Supercontig_0000058:1130865-1131078

TTATGGTGGAAAAATTATTTAACTATAGCTCAAATGATTCAATTTATTATATTTATTCTTCATCAAGGTCAATTATTTCTACCCTCAACAACATGTAACTATCCTAAAATATTCCCAGCTGCTATTATATTATATGCTACTGTATTCTTAATATTATTTACTAATTTTTATATTAAAGCATATTGGCATAAACAACGATTAGCTAAAAGTTTAG

>JAP08749.C|Supercontig_0000124:1104487-1104626

GAGAAATATTTCGATTCATTGATCGAGACAATGATGGTACAGTAAGCAGACAAGAATTTAGTACGTTGATACGGCTTGTCAGTTCGGAATATACGGATAATCAAATTAAGTTGTTAATGAATAAAGCAGATATGAATGGT

>JAP08750.C|Supercontig_0000291:187191-187516

TCAGTATTTAGAGTGAGACATACACCTTGTAACACCCAATGACTAGGATGAACTTCAGTTTTTAAATAAGCTTCGAAAACGAATCCAACTCCCATTGCATTCCGTCTTTCTGAAGTAACGTAAACTGGAGTACAAAATGTGTTTTGTAATTTTAAACGATGTGCTTCAATGGCATTAACTCTAAGAATAGGCAAGGGTTGAACTAATTGTCGTGATTTTTGACGTTTCAAACAACCTTTAGCTAAATCCCAGCTTGCACCCTCTAAATACAGTCCTTGAACAAAACATCCTTGATGTGCTCGTTCTTGTATTTCAGTAGAATCTGT

>JAP08752.C|Supercontig_0000034:180006-180193

ATTAAATTATTCACTTCAATATGACCATCTTCTTTAATTAATTGAGCAAGTAATTGATCATAATGAAAATTTATTGGATATGATTGATGTTTTCTTTGGTTTATAGTGCGTAAGAAATGTTGTCGTTGTGTTTCTTCAATTTGTAATGATTTCATAGTGAGCTGAGTTTCATTTGGACCTAAAATACT

>JAP08761.C|Supercontig_0000094:492867-493190

CCTGAGCTTCAGATAAAGCTTTTTGTTCTTTTTGTCTTGTAATTGTAAGTATTTCATTAATATTTCTATTATTTTCACGTTCATCTTCTAATTGTTCTTTAATTTGTTCTAATTCTACTTCAAAATCATCTAATTGTGCACGTAATTGATCATTATCTAATCGTTGTGTATTATTTTCACGTTGTACATTAGCTTTTTCTGTTTCACTTCGAGATAATGCATCACGTATTCTTGATATTTCTGTAGATAGTGTTTCAATTTCTGCACGAGACATTCTAAGTTGTTCATTAGCTTCAACTAGTGAACGTGATGTTGCATTGCTCT

>JAP08767.C|Supercontig_0000673:122305-122706

AGATATTTAATAGTGCTGGAGATGGTCCTGAATCAAGTTTCACAGAACAAACAACATTTAAAACAGCTCCACGTGAATTTCCCACTATGGTTACTTTAAATGCAACGGGTATACCAAATACTATTCGTGTTTCATGGGTGGGAATTCAAGCTAGACCAAATGAAGAATCAGTGGAAGGCTACTGTATTCGTTATTGGCCAACTGGCTCTCAATTCAAACGAACTTTCAAAGATATTAATGTTGGACTCCAAACTTTTGGTTACGTTAGTGATTTAAAAACTGATGTAAGATACAATTTACGTGTATATGGTTATAGTCGTGGAGGTGTTGGTACTATGAGTTCACCAGTAAATCAATTTCAAATTATTACTAAAGAAAAATGCATTCCTGGAGCAACTTGGG

>JAP08769.C|Supercontig_0000131:2726462-2726568

TTAATTTCATCTCCTATATCAGCCAATTCCCTTGCCAAGGCATTAACCAAGCTCCATTTACGCTCAAGCTTGTGTGATGCGGAACTTGTACTAATCTTGTGTTCCAT

>JAP08784.C|Supercontig_0000148:799986-800075

TATTCACAACAAGCTGTTGTTGGATTTTTTCTACCAAGTTGTAAATAGACATCTTCAGCTGTTACTGCTTGCTGATATACTATTTCATAT

>JAP08785.C|Supercontig_0000493:126838-126905

TGATTGACTAAGCTTCCACGTAAATGTATCGGTACATGATCACTTCGCGTTGAACCAGACGGTGCAAG

>JAP08792.C|Supercontig_0000359:37084-37305

AAAGCTGCAAGGCCAATTCTGTCCAGAAATCACGCAGAAGCTCGAAGGCGAGTCATTAGTTTGTATCGTGCCTGGTACAGACAACTTCCTTTTATACCAAAAGAATATAGTCATAGTAGCGTAGATCTTACAGTGCCAGTTTTGCATGCAAGATTAAGAGAAGAGTTCCGTAAAAATAAGGATATTAAAGATCTACGTATAATTGACTTGTTAATTCACAGA

>JAP08796.C|Supercontig_0000043:595674-595884

TTTCAATGAGATCTTGATGCTTGAACACAACCGCATAATTTTTGAAACCACATTTTTTGTCGTCTTTTATTTCTACATTGTGTAGAACTATCATAACTATGATAATCACGTCTACGTCTTATTGGTGCATATTCATGTTTTTCATGTTCTATTGTTATTTTACTTGGAATTAATTCATTAGTTGATTGATTTTGATCAATTTATGATTATC

>JAP08806.C|Supercontig_0000046:1302731-1303099

TTTGAACGATAAACAGCACTTTCAGGATAAATTATAACTTCAGGGTATTCCGTTTCACATTCTTCATCATTATTAGTAAGTAAGAGGTCGGTAAAACTGAAACCTGGTTCAGGATCCCATATTGTATGTGTTGTAAAGAATGTATTATCGGTGCATTCATTTGTCGATTCAAAAAACAATGTTGTACAATGCTGTGAAGAACTTAAGTCAATTGGATTTACAGTTATTATATCTACAGAGGGATCAAATGATAAATCTATCGCTTCAGTAGCTCCTGTCAATGTAAGATAATGTGTATCAGTAAGATCAAGACCAGAATTTGAATAAAATAAATTTTTGGATGAAAGACCATTGTCAATAATTCGATTA

>JAP08808.C|Supercontig_0000619:22196-22599

AACCAGTTGAATTAACATACACTTTATTAAGTGATGAAAACTCAATGGAGCTACGTCGTTTAAAACAAAATCATATGGATGAATTCATAGTTGTGGCTGAGGCTAATGGACCACATTTATCTAAACAAACAAATATTCAATCAAATTTAAACACTAATAACTACACTGTTATTGTCACATGGCATCCTGTTACATCTACTGGACATTCAAAAAGTGAATCTAGACGTTCATCGAGAACACTGAAAAATCGTACACCAAGTATAAAAACTACAAACCCAGAAATACGAGGTGTTCGATTAACTTGGGGTCCAAGATTATATGAACCAATAGAATATGAAGTGTACAGTAATATTATTCAACCTCTCATGGATCCAGAAAAAACTCAATCTAAAGTTTTAGATTTA

>JAP08815.C|Supercontig_0000156:1244863-1245137

GATTTAATTCAGGATCTGTTTCCATAGTAAATTTCAATATTCTAAGTGGTTCCTTAAATTTACCAAATGAATTTAATGCCCATGGTGTATATTGAATAATCGTCGGTTTTGGACAATATTTACACTTTAAATAATGATGTGCTCTTTTCAATAATTTTGGTGTATTATCCGGTTGTTCGATCAATTCTTCACTAGATGGTCCAAATACTTGATCTAACAAATAAACAAATTGTTGTGGATCAGAGTCATAACAGATTGAAGGATCATTATCATTA

>JAP08816.C|Supercontig_0000637:46345-46457

CAATCAAAATGTTTTCCAACAATACAATGCCAATGTGTATGGTGATGAGAGTCAAAGTAAGATTTAATGAAAGATGCAACCTCTCGTTCCTCTGTAAAACGATTCATTGCTTT

>JAP08828.C|Supercontig_0000304:599331-599398

ATTGAAACTGTACAATTATTCACAGCCTTGATGTACTTGGGACCATATTTGTATGATGTATACATACA

>JAP08849.C|Supercontig_0000665:26459-26824

ATTCATGTTGACTGTGAACACATAAAACTCCAAACCGCCTTTGTATCGACTTTTTCTATCGGCACCAAATGGGATTCTGGTAGAGTAAATTCTTTACAAATAAAATCTTTACACAAAGCAGTAGAAGAAAACCCGATTAATTTTGATATAAATGAAAGACTTATTGTAGGACGGAAACTAAAAATAGTGGGGATATTTCGTAGGAAGTGGGTTTGAAAGATCAGAAAAAAAGATCATGATTGAAATAAGCAATTAACATTAGCAAGTGTAGTGGAAGAATCCATATCGGAAACAAACGAATACGTTGAGGGCATCAAGCGAAATTCACGTAAACGATATTTGCAGATAGTAGTAAATAGAAAAAAA

>JAP08870.C|Supercontig_0000186:130506-130647

TTGAACGCTTATATGAAACTGTATGATCTACTCTATGTAATGTTGTAGAGATTGAACGTAATTTGATTTCTGTAGAGTTAACAATAAATTGTGGACCAGTCTCCTCCCAAGAAGTAGGAACATTTGCATTTCGATCAGCATA

>JAP08887.C|Supercontig_0000642:180842-181092

TTTGCATTGATTTCACCTTGTTCTGCTAATAAAACTTCATTTTCACGTTCTAATCTTCTTAAAGCTGATATAGAAGAATGATTATCTGATAAAGGTGAACATGTTTTTGAAATTAAACTATTCATTAATGTTTCTTTTTCAAGAAGTGTATTCTCAGCAACCAATGCTCTTTCACGCCATTTTTTTAAAATTTCCACTTGGTTACGTAATTCTGAATTTTCTTCTTCAGTAATAAGCCATTTCGCTCGTTC

>JAP08912.C|Supercontig_0000016:901443-901610

TGTACTATCACCATAACATACACTATTACGTGAATATCTACCTAATGAACCAGTACGTGTTATACCATTTTTTCCATCAGAATGATTTGAAGGTGCTGGACTAACTGAACCATTTTGACATTGTAATAATAAACCACGTTGTGTTGAACTAATTGAACCACGTGATGG

>JAP08915.C|Supercontig_0000025:449696-449888

GTCACATTTATTCTAACCAGAAATATTTGTGCTGTTCCATTTTGGTATTTGGTATCCTATAATATGCATCATCATAGCAATGAAGAACAACGGATATTTCTTATTGGTGTCTTCAAAATTTATTTTATTTTGGGTATCATACTTGACATACTGAATCTATTCTGGGGTAAAATTATATGTTCAATGTTGTGGC

>JAP08920.C|Supercontig_0000488:531636-531939

AATTCTTTCAAATTCAACTCTTTCACGACCAGCTTCAATTGCTAACTGACGTTTTCTGTCTTCAGCTTGCGCTGCTCTAGCTAATTTCAATTCATTTTCTGTTTCAATTTTTTTCTGTGCAGCTAATAATTCTTTACGTCTCCATTCTCTTTCATTAGCTTCAGCTGCGCGTTTAGCTCTCAGTGCATCACGTTCTGCGGCTTCATCCGATGCTTTTTCTTGTAATGCACGTAAACGTGTAATTTCTAGTTCTTTCTCATGTTTTATACGTAATTGTTCAGCTTCATAGGCAGCTTCACGTTCC

>JAP08940.C|Supercontig_0000045:499677-499953

TTGTGTTTGAGTTACAACGTTTCATCGATGCTATGGCCACCTACCATGATCAGTGTTATGGTGTCATGAAGCAAATTAAAATTTTTCCACTTGAAGTGGATCTGGCTAGAGATGCATTTGCGTACAACCTAGGGATTTTTAATACAGGCGAAGATGAAGGCGGTGAAGATATCGATGAGGATGTAATCAGTGCTGAGTCAGTTGGAGAAAAATCTGTCAGTAGCAAGTATAACCAGTTAACTGACGATGTCAAATTAATAGACTTGGCAGGAATAAA

>JAP08947.C|Supercontig_0000360:146947-147148

TCTTCTAATTTAGCTTTAGAGCTTCGGGTTTCTGAAATTCTTTTGTTCAAAGCACGATTAGTTGCTTCAATTTGGCGTTTTATGTCAGATGCAACTTGTTGTATAATTCCATCAACAATGCTTCTTAATTCATGTGAATTTTGCAATTGTTTATCCCCATTTTCTATTGAGGCAGTTGAAAATGTTTGCCATTCTTCTGGAG

>JAP08948.C|Supercontig_0000237:60578-60747

GAAGAAGCATGCCATAAGGCATGTGAATCAAATATCCAACCAATTGGTACAAAATCGCATAATTCTAGCAGCATAAAAACCATAGAGGATGTAACTGCTAACCAACAATAGATAATATATGGTTGTCTTTTATAGTCACAAAAGTAAACGCAGAAGAATAACCAGCCAAA

>JAP08957.C|Supercontig_0000435:121126-121387

TATCTGGTGCTCGAGTTCTGGGCCGTGCATTTGCCCAAGCTGTCAAAGAAGAATACGCTTCAAGTCAAAGAGTTGCTAATGCCCGTCAAAACAATGGGTCTGGTGGTCCTGAACAAAATACTTATGTCCAAAACGCTGGGATCTCACTAGAGGAAGCAAAACAAATATTGAATGTCAAAGATATTCACGATATAAGTACTTTAAATAAACACTATGAACACCTCTTTTCCTCCAACAGTAAAGATAAAGGTGGATCATTTTA

>JAP08959.C|Supercontig_0000096:2176533-2176700

TTTTCCTCAATAAATCCATCACACCCATTATGGCAGCATTACATTTATCTTGTTCTCGATCTTGTCTATGAAATCCAATTAGCATAACAAACTGTAATCCTTCTTCAGATCTTAAATATTCTTCATTTGGTATTTGTAGTCGCGTTGCTGTGAAAGTACTCAAATTGA

>JAP08968.C|Supercontig_0000397:17629-17731

TTTTTTTCAAGTGTGGCGCTCATCTGGGCCATGTGTTCGAAGATGGACCTAAACCAACAGGTTTACGTTATTGTGTAAACAGTGCATCTCTGTCATTTAAAAA

>JAP08987.C|Supercontig_0000136:11106-11275

TAAACCGCGTAAACCCATACCTGAAGAACATGACAGTTTCCTAGATGATTTAATTGATGTTAATGACGACACAACCACAGAAATTAATGTTTCGGAATTATTAGCAGCAGGTGAACGTGACTTAGATACTCTTATGTTGGGAGGACGTGTTCCACTGAAAAAGAGCTCAT

>JAP08990.C|Supercontig_0000241:405854-405933

ATGTTAGACGGAGAGCTTGTGCCTTCAGGTACATTTCAAGCAGAAGTTATTCCAGGGATTTTAAATGTTATTTCAGGTAC

>JAP09001.C|Supercontig_0000312:1402860-1403071

AAATCACACAGAAATGGAATCCGTTATGTCGTAGTTTGCCTTATGCTACATTAACTCATCATCCTCTCTATTCTAAACGTGATCAATTGCCATCAAAACAAGTAAATGTACCAGGTGCCGGTTATGAAGATTTATCTAATCCAGTATCTGTATACGATGTGAATAATTTTCTTACAGCACTACGTGAAGCTTGTTCTGGTATGAATATTCAG

>JAP09002.C|Supercontig_0001960:28549-28678

TTGAACTTTTGGATCTGGTTTGGCTGGATTACAAGTAATTGCATCGGGCTCATTTTCATTGATACGCTGATTACGATACTTCTCAATTGCATACAGCATTTTTTCAGCTTCAGCCTTTTGTGCATCATCT

>JAP09010.C|Supercontig_0000062:940503-940709

ATTAAAGCAGTTAAAGGATTACCAGAATAACAACTATAAAAACCACCGAAAGGTAACGAATATTTACCGCCTAATTCACGATCAGTTGATAAACAGATACGCATACGTCGACCAAGTGTTATTGGCCAGTAATATTGGGGACAATTATATTGTTTAGTTAAACTGTTCACCTCTTTATCTGTATAAAGACCACCAAATAAATAACCA

>JAP09024.C|Supercontig_0000466:406146-406213

ACACCAGCGAAACAAGCTCCACTTGGTTGTGGATTAAGTAGATAAAAGAAGATTGCTGATTGATAATT

>JAP09035.C|Supercontig_0000036:669718-669814

TAAATACAAAAACAAGTTCAGATCGTGCCAGATTACTAGAAGATTTGCAAGAATGTATATTGGAAGTCACGGAGATGGAGCGTTTAAGACGAGAAGA

>JAP09056.C|Supercontig_0000466:355201-355412

AAAAACGACTCATATGATGGACAAGCGAATATATCCGAACAGTTACCATAGAAGATCAATTTGAAATTCGAATTTTCACCCTGTCTATGTAGATCAATTACAACACCCGTAAATTGTGACAAATTCCAAGTATTCAATATGTAGTCGACACCGGCGAAACATGATCCATCTGGTTGTGGGTTGAGTAGATAGAAGAATATTCCTGATTGATA

>JAP09058.C|Supercontig_0000464:126513-126691

ACTTTTAATTTGATATTCATTCTCACGTAATGCTCTAAGAGTATCATGTATACGCCTACGTAGTTCATTATCTACTTGACTAGCTTTGCTGGACATCCTATTGATTGCTTTATGCAGACTATTATGCAATCGATGTAGCAAATCATCAGCTTTTTCAATTACATTTTCAGCATTTTCTA

>JAP09075.C|Supercontig_0000214:194414-194617

CTACTTGGAATTGGCGCATCGTCAGTGTACCAAATTTGCCAATCTTGTAAGCCTAAATGTACAACTGCCAATCTGATGTACTGCTGAAATGTGCAGCAAATTTTGTGAATACTCAAATCATTATTTAAAATAAAAACACCCTTTTGATTATCACTAAAAACAAAAATAACCGTGGCGCCATTCAGACACTTTTCTAGTTCATAA

>JAP09129.C|Supercontig_0000036:26183-26323

AATTTAGTTATATTTGCTGAATTGTTTTGGAATCCTGGTGCATTAGTTCAAGCTGAAGATAGGGCATATCGAATTGGTCAAATGGATTCTGTGTTAATACGTTATTTAATTGCAGAACAAACTGCAGATGATTTCATTTGG

>JAP09132.C|Supercontig_0000003:2009430-2009517

GTTAATCCACGTTTATTGGCTTGGCAGGCACGTTCATTCTACAGACATTTTCCAAATTGGCTTCCTTCACAAACAGCAAATGATCAGG

>JAP09141.C|Supercontig_0000218:237817-237975

TTTTAATTATGATTTATTAATTATATCATGTGCATAGAATTCAAGTATAGAATTATTTTTTATATCAAAACGTACACCGGGTCTGTTAGAACGATTTGATGTTAATTTATCTTTTGATTGTGTATCAAGTTTATTTTTTAGTGTAGATGATTTTGGTCT

>JAP09160.C|Supercontig_0000680:347348-347462

TTATTTCGCTTTAAAAACTGTATTTCATCTTGATGTTTCTTTTCTTCTAACTGTCTTTGTTCTGCTGCTCGTTTTTCAATTTGTTCACACTTTGTTTTTAATTCATATACTTGTC

>JAP09168.C|Supercontig_0000003:1223847-1223981

CAGAGTGTTGGTTGCATTGCAATGATGCTAATGTATCTTTATGCGATTTCTCAGAAGTAGCATCAGCTCAGGCCTATATTCTATTTTATTCAGAGCTCATGCCACGAACACCCTTTCCAAAGTGGTATAGAGATT

>JAP09175.C|Supercontig_0000228:554453-554556

TGTTCTCGTTGTTTTTCCTCATTATTGACTTGATAGTCAATGTGGATACGATTTGTCCTGTTTTCTGACTTTTTATGGTGTTCCTCTACATCCCTTTTCGTTCT

>JAP09182.C|Supercontig_0000096:1942937-1942997

CTTTCTCACTTCTATTGGTTCATCATAATGAAATTCTTTGACAGGAGACCAACGTTTATCG

>JAP09189.C|Supercontig_0000065:479198-479409

TTCAACTTCACAAGGATCATGAATGACAGGTTTTTTATTCTGATGGTCACAACATTTACGCTTGATAACCAAGCCCCAATAAATTAAACACAAAGGAATCGGTGTAAGCATAGCTACAGTAATTGGCATAATGGCACCTAATTCACCAGTTGGTTTTGGCATTGTATACAATAAAACAAGTATTCCAACACCAATATTTTGTATACCAGTCT

>JAP09199.C|Supercontig_0000040:515224-515434

GCAGAAGCTTATGAATATATGACAACATATAAAGAAGTTGTAGCACGAGTAGGTCGTACTGAAAATGTTCTTGGTTGGTATCATTCTCATCCAGGTTATGGATGTTGGCTTTCTGGTATTGATGTTAGTACTCAATTAACAAATCAAACTTATCAAGAACCATTTGTTGCTATTGTTATAGATCCAATCAGAACAATATCTTCAGGAAAAG

>JAP09213.C|Supercontig_0000417:164088-164198

CAGTGGTCAAACTTTGTTCATGATTTTGACAAGAAGCAATGCAATTTCCGGTTGGCGGCAACTTATGGGACCAACAGATCCAAACAAAGCATCTGACGAATCATCAGAAAG

>JAP09239.C|Supercontig_0000349:154581-154738

CCAGCATCTAAATATACGTCTACAATGGGATCAGTTAATTTCGGATACTTATACCATTCTGTAATCCCATGTTTACTTAAACAACGTATACTACAGAAATTATCAATGCGTTTCTGTGCAACCGAATGATCATAATGCGCATAACGTAGAAAACCAAG

>JAP09253.C|Supercontig_0000030:8404-8587

CAATTATTCTATCTAAAGCAGCTAAACTTGTATGTTGATGTAGTTTACAATCTTTTAAAAATTGACAGAACTGAACATAACGTAGTATAATTGTATTATCTGGTAACTGTTTCATACCAAGTTTACCATAAAATCTATACATACTGCGTAATGGAGTCAAATAAGCAGTTATTACATTTTGCAT

>JAP09263.C|Supercontig_0000040:517894-518102

AAATTGAAGATTTTGGAGTACATTGTAAACATTATTATTCTTTAGAAGTATCTCATTTTAAATCTGTATTAGATAAACGTTTATTAGATTCATTATGGAATAAATATTGGGTGAATACATTAAGTTCAGTTAGTATATTAGCTCAACCAGATTATTTAGCTGGTTTAACAAAAGATCTTGCTGAAAAAGTTGAACATGCTGGATCATCA

>JAP09265.C|Supercontig_0000222:146519-146695

TTTAAATACATCAGTTTCAATAAAACAATGACCCAATTAAGTTCAACACTTGGTAGTTGTGCAAAAGATCTAGCTGGTTTCGCTATAATGTTCTTTATTGTGTTCTTTTCATTTGCACAACTTGGTTATTTAGCATTTGGTACACAAGCAAAAGATTTCAGTTCATTTATTACAGTT

>JAP09285.C|Supercontig_0000096:1774518-1774735

AGCATGCATGACTCATAAGTTGGTACCATATATTTTATATTTATGAAGGCATTTTCTCCACCATGCATCTCAAGTATTTCCAAAGTTTCATGGACCAAATCACCCAGATTCTCGCGTTTTTGTTGACTATAATCAATTCCGTCGCCTAAATTCTTGTTTTTATTTATAAATGTATTCATAACAGGTAAAAGCTGTCTGTAGTACGGTACAAGAGCTTC

>JAP09291.C|Supercontig_0000123:1355096-1355239

TTCGTCAAGGATTCAAGTCATTAAATGTTGATCTAACCACTGATAAAAGTTTCATTACTTATTGGGATAAGGTTGGTCGTTTTGACTTGTTATCTTCATTGTATGCTGAGCACATACTGAAACAAAATTCTCCATCATTATTGG

>JAP09294.C|Supercontig_0000162:137365-137597

TTTAATTGACAATAATAATTTGACAAAACTTTCCATGCTTTCTCAGCCATAAATCCATTCGGTGGTTCCATACCTTCACGAGCTAATGTACGCATTTTAGTACCAGATATAAATAGAAAATCTGATGAACGTGTTGAATCAAAAAATGACATTTTATTAATTGTTTTGTCATAGGCAGCAACACGAAATGGAATAATTTTTAAATTGGATAAACCAGGAGCCATAGATAATAC

>JAP09304.C|Supercontig_0000657:104950-105155

GTTAAACCATGTACCCAACAACCTCGACAATTTTCACAGTTTGATTCTGATCAACATGTAAACTCATCAGCAGTCTCATGGATAGAACCATATCCGAAACCACAAGGTGATCCACCACCTGCCTTTTTCAACTGGCTTCGTAATCGATTTGGTTCTTTACGTCAAAGTATTATGAGTCGTTTATCAAGTCAAAGTAACCAATTACA

>JAP09317.C|Supercontig_0000087:12851-12997

ATTTAGAAATACTGAATTCAGTTGACGGCCCATATCAGAGTACTTATTCAACAGATTATGTTGGATGCTGGACTCCTCAACCAGATTCTCCGATTAGAACAGCTACTTCAAGTGGGACAAGAGCAAATAAACCTCATCCAAGAAAGG

>JAP09318.C|Supercontig_0000644:320108-320336

ACCTGAAATTACTACAAGGATGTATCACCCATTTTCCTTGTTGTACTTGGCGTCGTTTCTCTTTAAGCAGTGCATTTTTACTTCCAAATAATTTCATAGCTAGTTTATTATCGGATGGTTGAAAAAAAGCTTGAAATTGTTCTTTTAAATAACTTGATGTACTTTGTTTTAATGGATCCACAGTTGATTCATTTACACTACCATTAGCTAAAGCGGTAATAGCTGTTGT

>JAP09319.C|Supercontig_0000076:778654-778882

ATGACACAAAATACAAGCATTCCAGTCAATTATCCGATGATGCCTCCGATGGATGATTTAAATAAATACGCACATTTATTGCAAGTCATTGAAGAAATGGGACGAGATATTAAACCAACCTACGCCAATAACAAAAATGCCGCAGAAAGACTAAAAAAGAATATTCACACTGCTAGGATTTTAGTCAGAGAATGTGTTTCAGAACTTGAACGTGTTATGAAAGCATAGT

>JAP09322.C|Supercontig_0000115:688816-688973

TACAGTTGCAATCAAAGAGCGGAGCTGTTGGGCATATAAAAGAACCTGTTGGAACCCATGGTCATATGAAATGCGTTTTCGATCGACCTATTCTAGCAAACGATGTAGCTTTAATGCCTTTGTACAAGCGAGTGTTTCCAAAATATGTGTACGAACCA

>JAP09326.C|Supercontig_0000311:338013-338119

TAGCCGACGTGATAAATCGATTGATTCATTATCAGAATTACGTTTAATGCCAGAAATTAGCATTATAGGAAAATCTTCAGATGTAAATCGATAATTATTATTATTAT

>JAP09329.C|Supercontig_0001522:41484-41655

AATAACACTTTGTTGAACCCATTTAGGCAATTGTGATTGAATCGTTGAACGATAATACCGATTATCCATCAAGAAGACAGCAGCATAATCTTTGGCATGTCGTATAGATCGACCAATAGCTTGATTTATTAAACGCATACACATTGTCTCATAATATTGCCTACCTGGATTT

>JAP09331.C|Supercontig_0000228:554453-554556

TGTTCTCGTTGTTTTTCCTCATTATTGACTTGATAGTCAATGTGGATACGATTTGTCCTGTTTTCTGACTTTTTATGGTGTTCCTCTACATCCCTTTTCGTTCT

>JAP09358.C|Supercontig_0000449:252764-252915

TTATTTCTAGGATTTGAATTAGGCTTATCTAATGCGACAATGACACCGAATAAAATATCAATAAATAATAAATTACTTGGATCAATTTGTACTTATATTTATCGAAAAGTTATGGCTGATATTATTAGTGAACGGACTCGAACCACAGATTT

>JAP09380.C|Supercontig_0000003:663444-663776

CGTCTTACCCAGTTACAACAAGGTGAAACGGAAAAACAAACAAATCAACGTTTACAAATTTCTCGATTACAACGTGAACTACGTGAAACAAAAGATAGAGCAGAATCACTTGATTTACAAATAGGAGTTATGCGACGTCGTTTAATTGACGCTCAGGAGAATAGGAATCAAACAGTTATGCCAGCAAGTAGTACTCCTATGACATTGGCTACATCGGAGAAAGAAAGACGAAAATTATTAAAACAATTAGAAAATGTTAAAGTATTAGAAAATAATCTGCGTCAAGAAGTGGTCATGTTAAAAGCTAGATTACTTGAATCAAGTCAGACTAAG

>JAP09391.C|Supercontig_0000243:70520-70740

GATGACTATGATTGCAAAATGCTTGTCCTTTATGGAGTAAATTACAATGTTAAAAAAGGTGAAATAGCAAGAAAATTCCCTTCTTCCACTAATATATCACTTGATAAAACTAGTCACTTAAATGCTAGACGTAAACCTGGCGTCGCTTACTTGACTTTTGCATCAGAAGAAGATGCTGTGAATGCCATAAAATGTAGACATGGTTGCTTATTGAGAGGTAG

>JAP09394.C|Supercontig_0000144:186639-186842

TGATTCTGATTCTAATTCAATGCCAAAATCATTTTCTTCATGTTTTGACTTGGATAATGCTTCTTTAAGTTTTTCATGTTTAATTTGATTGGCTTTTAGACGTTCTTCAATATCCCAAGGGGAATCAAGTGGGAGATTTAATTTTTGTAAGTGATTACGCCATTCAGTTTGACGCTGATTACACCATGAATCAGCACAATCTGG

>JAP09413.C|Supercontig_0000644:133251-133437

CAGATTCTTCATCTAAAAATGGTGGATTTAAAAACTCCAATTCTATTAGATCATTTGTAGGTTGTGAGAATATTTTGTAGCTATTATGAACATTTTCATAGTCGATACTTAGTTGTTCAGTTAGTTGATTGAATGCTTTTGACATAACGTCACGAAATAATGCAGCTTGTTCTTTTAATTTTTGTTC

>JAP09415.C|Supercontig_0000644:221144-221371

GAACCGTACGGATTTGATCGTGGTCTAGCACCTGACTTTATCAATATGGTTACGAAAAAGGACAATGAATTGTATTTTCTAATCAAATGGTAAAGAAATGTTTCCATAACTCATCAGTAAACATTAGGAAAGGAAGTCAAGTCCGCGATGTCGTACCTGCTGCTCAAGCAAACATTCGGTGTCCACAAATTGTTATCAAATTCTACGAAAGCATTTTGCATTTTACTT

>JAP09433.C|Supercontig_0000305:25841-26103

TAACAACTATATCTAGTTTACAAAATAAACATCAGCGTCTACAACAAAATCTCCCCAATTTAAATAGCAAACGACATCATAATCACATGAGTTACATAAACAAATTATACGATAAATTCAATGCAAATGAAAATTTACGCAATATAAAGCTTGACAAATCACTATTATTGAACGACAATTTTAGCGGTGAATCAACCAGTGGGATGACAATATCAGAATCTTATCAAAATCAACTGATTAATTATTCACATGAACTACGAATG

>JAP09436.C|Supercontig_0000666:118263-118433

ACCTGTTCCCCCCAACCAATTATTTGTGCATTTGAATTTAATTGTCTAGCTGTATCCAATGCAACTGGTCCAACAGCTGCTTGTACGGTAGCTTTTGGTAACCACGCGAATGATATGAACAAACGTTCACGCATATTTAACTTTGACGGTAAAACAGCCACCATTGTTGCT

>JAP09439.C|Supercontig_0000035:594111-594498

TTTCATGACTTCTCGTGATAAATATGTTTATTCTCAACAGAAACATGGGGGGATTAGTGCATCTGCAAACTTAGCTAGTAGCACTTCAGAATACTTGCCAAGACCTGTACATGATGATCATGAACGTCTCAACCTTGAACTTAGAGAGAAAGTTGACGGACTTCGTAGTCTTTCGATAAAAATAGGAGATGAATTACGATCCCAAAATAGTTTACTTGGAGATATGACTGGAGCGTTTGATCGATCAGAAGGTGTTCTTCTTTCTACAATGTCTCGATTATCTCGAATGGCTAAACAGAATTTATCCTCTGGATTATGTTGTTATGTAATTGTATTCGTGTCCATCATTTTCCTCATGTGCTGGTTTATTCTCCGATTTCTTTAGTCA

>JAP09444.C|Supercontig_0001524:13058-13253

CATGACCATCCTTGATTTAGAATTTGTTCCATTAAACCAGCTTCTTTAATTCTATCACGTTGCCAATTTGTATAAGCTTCATTTGATGGTAGTGAACGTATTGGATTATGTAATTCATCAATTGGTGTATTATTTTGTTTAGAATCCAGTAGAATACTAACACCAATACCATTTTGTCCTTTATGTAAATTATCTA

>JAP09447.C|Supercontig_0000081:150485-150603

ACCTTTATTTTCACGTATATCAAAAGTCAACCAATTTGGCAGTCATTAAGGTTTTGGAATGCCTGCTTTTTCCAATCTTTACAGGAGGCACGTTCAAAGGCAGAGGAATCATCATATGA

>JAP09475.C|Supercontig_0000291:544008-544204

AGAATTTGAGCATACACATTATCCTGATGTATTTTCTCGTGAAAAGTTATCCAGTCGAATTCTACTGCCTGAAACTCGTATACAAGTTTGGTTTTCTAATAGACGTGCAAAATGGAGACGTGAAGAAAAAATGCGAATGAAACAATTAGCTTCTAGAAATCAACAGAAACCAATTCATCATCAGTATCATAGTAACA

>JAP09484.C|Supercontig_0000173:456590-456748

TTTAGTCTCTGGAACGAATGGCTGCACGTTTAAGTCGTTTCTCCGTACTTAATCGTCAAATATTTTCTACATTAAATGTATATCTTCATCCTGTCGATAGACTAGATGAATCAAGTGTTCGTGTTCGCCAGTTTCCTATACCTACCTGGTCAAAATCAT

>JAP09488.C|Supercontig_0000067:796110-796234

AATTGTTCCATTTGATGTATTTGTCTCCAATCAACTGGTTTATTTGAATTCATAATAAAACCAGGTAAACCAATTAAATCAAGCAGATTACGCCATATACCATGATCATATGGACGATTATATTT

>JAP09492.C|Supercontig_0000073:266535-266859

TGCAACACACAAATTTATGCAATGAAAATTAAAAAACAATGCAAAGTAATGTAGATGATACACATTATAAGTAAATGGTAATAAAAAAAGACTACAAAAAACCAACAAGCAAAAACATACATAATTGTTTCCATTATTGAAAACGAAATACAATAGAGTAAATAAATCCAAAATCTTGTACTCTATCAGATTAGAAAGTAATGCTGCTTGCCAGAAATAATAAAAACACACAATGACTATAAAAAGAAAGGCACAAGTGCGAAATAATTGTGTAGTACGATAAAGATAAGTAACATAAGATAAAAACAAAACAAATGCGACTGTG

>JAP09502.C|Supercontig_0000277:40100-40437

CGTTTGTTTATATTCTGTTAAGTAAAATGATTCACATAAAAATTGATTTAGTCTCAGATGAATAAAATAATTGAAATTGAACAATACATTTTAAAGTTATTTGCTCATCAAGCATTAAAGATTATTCATTTAGTTTCTACTACCCATAATGGTTCTGGACCACGTTTAGCGTACGCACCAGCAAATGCACCATCCTCACACTGACTTAATATATTCTCCATAGTTTCTATAGAGTAGCGGATTCTACTGATTTGAGATGTGGAACAAATTTCATTTGACTTAGTTTTAATTGCTTCGATTGTCTGCCAAGTGAAAACATACAAAGTGATTGTTATCCA

>JAP09503.C|Supercontig_0000089:640595-640773

TAATCATAGGTACTAATAATGGAATAGGATGTTGTTATACTACATCTAATCGTCGTATTCAAAATGGAAATATTCATTGTGAACTTGATGTTGAATCAGCTGATTCTGGACGTGGAGCTAGTGAAGATGATCCAAGTCAATTAGGTGGACAATTTATGCATTTTTATCCTACATGTCAT

>JAP09507.C|Supercontig_0000303:394100-394254

CAAAGTTTATCTAATTCACACTTATTTGCTGCAGCTTTAATCATCGGCAAAAGAGCCGAAACAAAAGTTCTTGCATAGTACTTGAGCCCTTCTGATTGTGCAAAATTACTAACATCAAGTCCTTGAAGTCTTGAAAATTTACAAATATATTCTGG

>JAP09508.C|Supercontig_0000035:489697-489836

GTAAATAGAGCGTCTAATTTGCCAGCAAAAGATCGGAGTGGAAAATCTAATGTTTTCTGTGAAATAAGACTTGTTAATCGAGTTGTACGAACATTCACAGCACAGAAGAATGCTAATCCTGTATGGAATCAGGCATTTGT

>JAP09512.C|Supercontig_0000073:681471-681722

CCTCGTTCTCGTGTACGAATAGTGCTTAACTTTCGACCGTATACACTAGGAAAATCAAGTTCCGTTCTGGGAATTAGTGTTGAATATCACAAACAAGAAAATTTATCTGTGGAAAAACCCAATTTTAATGTCAAACCAACTATCGATTCTTTTCAAGTTCACTTATTCGGTACAATGAATTAACTTCAAATCTTTTTTATTCCTTGTCTTTTACAACTGTATGTTTTTACTGGTGATTTTAATATATATTTA

>JAP09517.C|Supercontig_0000262:6417-6537

TATATTTAAAACACTAAATAATATTCTTGGATGGATAACTATAACATGGCCATGGAATCCTACAATTGTCAATTTCCCGAAATCTATTAGGGAACCAAAAATAACTTTAACACAGAATTCA

>JAP09523.C|Supercontig_0000235:32799-33026

GGTTCTAGATCATGTCCTGGTGCTCGTATAGCTAATTTATTAATTGAACAAATTTTAACAGCTATTAATCAAGAATTTCTTATTCAAAATATTACTCAATCACCATTTGAAACAATTAGTCCAGGTAATCAAGAAAGTCTAACACCTTTTGGAATTACACGAACACCACATAAATCTATGTATATATTTGTTACAAAATTAAATGGAAATAGAAGAACAAGTATTTAA

>JAP09531.C|Supercontig_0000061:138292-138386

ACTGTAGGAAGCGTTTTATGTGCAATGGTAACGACATTTGTGAGCTATCAAAATGCATATGTAGTTCAGTTTACTGGCTGTGGATTCGTATGCTT

>JAP09534.C|Supercontig_0010771:4898-5185

TAGAAAAATGGCCATTCAAGAGTTCAACCTGACTTCAGTTCTCGAAGGTCTTGGTTTAACTATGGATCAATTTATAGATTTGTGCATATTACTTGGTTGTGACTATGTTGATACAATACGGGGAATCGGCCCTAAGAAAGCGTTAGATTTGTTGCATAAGTATCAGTCGATTGATTGTGTTTTGAAGAATATCGATAAATCTAAATATATTGTCCCAGATGATTGGCCTTACGAAGATGCGAAAAAGCTTTTCTTGAATCCCGAAGTGACAGACCCTTCATCAATTGA

>JAP09538.C|Supercontig_0010787:2469-2779

AAATTTATTTATTTAACAAATAAATCTTTTTCTGTTGGTTCATCTATTTCACCTTGTGTTGATCGTTTAAGTGAATCAGAACGTCGTAAAGTTGAACATCTTGAAAAATGATGAATTGGAGAATCTGTTAGATTTGGAGTTGTTGGTGAGACATTTTTATAATTTTTTACTGCACTATCAACAATACTTGGTAAAATTAATTTTGGCGGTTCAGGATCTATTAAATCTTTTAGAACTGATTTTCGATGACTTTCAGTTTTGAAACGTTTTGCATCTTGACGAGCTAAATTTCTTTTCTTTATTTTTCGTCT

>JAP09556.C|Supercontig_0000019:1089440-1089617

ATTTCAAATTATGCTACTGTATTTCCAAATGAAATGCTATTAGAAATGGCTTCAAGTTTGCCAGCTACTCGTGAAGAATTACTTCAAATACCTCAGTGTACGGATTACAAATTGAATTGTTTCAATGCAGAATCATCATTTTTAGAAATTACTTTAAATTATCTTTCAATTCTTGGTG

>JAP09572.C|Supercontig_0000090:806157-806332

GCTGATGCTTATGATTGTAGAGCTATTATATATTTACAACTTGGAAGACCACAATGTGCTATTGATGATCTTACAACTGCAATTCGTTTAAATCATTATTCTGCAAAATATCTTGTCAATCGTGGTGTTGCATATTTTCGTAATCATCAATTGATGCCTGCTATGATTGATTTCAA

>JAP09582.C|Supercontig_0000661:142296-142467

ATGATAGTAATGAAAATGACAATAATAAACGCCGACGACGAACACGAACTAATTTTTCAGGACAACAGTTGACTGAATTAGAATTAGTCTTCCGTGTTAGTCATTATCCAAGCATGATTGTAAGAGAAGAGTTGGCACAACGTTTAGGTTTACCAGAATCCCGTATTCAAGT

>JAP09621.C|Supercontig_0000292:910470-910800

TAATCTTCTTTCTCTCGCATATTTCCATAAAAATAATTAGAATAATAATGATGATATCTATCATATTCACTTCTTAACATATGATTGGCTCTTTCATGAATATAATTACTAGCATCAGTTGTTTCAGCTAGCATATTTTTAGCTTGTTTCGAATAAAAATATGGATTAATTCTAGGTGGTGGATTTGGATCATATTTTACCGGTGGTGGATAAACAAGATATATACTACGTGTACGTAATGTTGGTTGTATAGGTGTAGTAATTCTATGCTGATTATCAATTGAATCTGAAGTAGTTGATTTTTCTTTTATTATTCCATTTCTATTCATTG

>JAP09627.C|Supercontig_0000005:3775766-3775894

CTTTATACAATAATTCTGCAGCATCTAAAAATCGGCCAGCATGTTTATATAATTCAACAGCTTCAATTATTCTACCTTGTTCAATAAAACAAACAACTGACTGTTTTAATAAATTATCAATTCTTTTTT

>JAP09642.C|Supercontig_0000016:209593-209836

ATAAATAATACTGGTCGGACAATTATACATTTATGTGTTATTTACTCACAATTAGATGTTTTAAATTATCTTTTAACTAGCAGCAATCATCATAATTATATGAAAGAAATCGTAAATGAATATGATGATCAAGGTGCCACAGCTTTACATTATTCTGTTCAGGTGAACGCTGAACAAATCGATGAACTATTTGAATTATTAATCAATATCGGTGGAGCAAATTTAAACGCACCGGATACACATG

>JAP09660.C|Supercontig_0000192:19080-19143

AAAGGCATTAAAGTTGCTAAAATCACACTATCCGCTTCATTAGACCCCACAGAATGATAATTAG

>JAP09670.C|Supercontig_0000107:485461-485643

TGTAAATGTCCTAATATTTGTTATAGTATTCTATGGTTTTTTATTTTAATATTTATTGCATGGCCATTATCATTCTTTATATCAATCATTTACTTAATATTAATTGTGTTCAGTGTTTGTTGCCCTGTTTTACAACCATTAACAGATGGTATAAGTAAAGTGATGATGTTTCCGGTAATGTGT

>JAP09671.C|Supercontig_0000093:807198-807407

GCGCTGGACGGTTCCGACCCAAATGGGTTTGTTGGTAAGACTTGAATAGTTTATTGTTATATTCTGTGTTCCAGGTGTTATGTGGTCAATATGCGGTGTGCATGATCAAGGTTGGGCAGAAAGGCCTGTTTTTGGAAAAATCAGATGCATGACCTATAAAGGATGTTTAGGGAAGTTTTCTGTACCTACGTTTGTAGCGCGATACCCCAA

>JAP09716.C|Supercontig_0000480:489925-490040

ATGTTGACAAAATATCTCGATTTTCACGTATACGTAAAGATAGAAATAATTGTTGGGAAGCGATTAAAGCCCATGATACAATGGTAACAGTAGCTGTGTTCAGTCCAAATCCAAAT

>JAP09718.C|Supercontig_0000050:913933-914123

TACTAATCATAGAATCAATGGATTTATTGGGAACATTTTCAATTGATTGGAAAACTCCATTAGAAAATTTATCTGAAGCGCCAGTTATCAACAAAAGTGGGATTTTGATTAAATTAAAAGAAAAACAGAAAAAATTGTTCACATCAGGTCATCAAATTAAACAATTTGACTGTGATCTTAATTTAGCCAAA

>JAP09724.C|Supercontig_0019089:321581-321691

CACGATTTTGTCTTTCTTCTAATTCCATTGTCATAAGACGAAGCATTGCAGCTAATTGTTCACGTGTTTCTCTTAATTGTTCAACTACTCTTCTTTGTCTTTGAGGTACTA

>JAP09738.C|Supercontig_0000191:331979-332085

ATAAACCATATTCTGCAAGCATATTACGTTTGGCCAAATCAGACGGTTTTGTGCGGTTCATAGTTTGTTTCTTCTGGGGTTTATCAAATGATAAGGCAGATGAATAC

>JAP09743.C|Supercontig_0000075:142171-142306

TATCCCATGAAAATCCAGGTTTATCATATGGTAATGATGGACAACAGGTTATAGGCCATGTACCATGTTCATAACGATATTTTAATTCTGTATTACTAATAAACTTTAATATTGTTAATAATACCATTAATATTGT

>JAP09746.C|Supercontig_0000540:83478-83625

TCTGAAATGTATTCCACAGGTGGCAAGCATTGTAAGCGTCGCCAAAGTGTCTGAAAGGCGTCTGTTTGTGGAAGACACATAAGTAGACAATATAAGGTTTCGCGAAGATCAGCACTGAATCGTTTATCAAGCATATGCATCCGAAGAC

>JAP09752.C|Supercontig_0000044:548892-549092

CTGATTACCGGTTTGATGTCGGTGAACTAAAATATTAACCATAGCTTTAGAACCATTTGGATAATTTCCAGTAGGTATTCCATGCTGTACAGTTTGAGGTTTTGAATATGATGATAATTTATTTGGTTTCATTGTAGAATAAATACGATTTGATGAATTTCTTGAACTTAACATCTTTTTAGCATTTTCTAATGGTGTATG

>JAP09759.C|Supercontig_0019090:103798-103947

TTTTTATTTGGTAGTGTATGATGTTTCTCATAATTTAATCCTGTTAAACCTGGTGGTACACTGGCGATGTGTTTGCGTATTTCTGCGGAAATATTCTTCTTATCTTTACTCTGAAGAGTTGCATATATATTACCATGTAACGGACAAGGT

>JAP09765.C|Supercontig_0000074:1113619-1113802

GATTCTTTTAGCTGAACGTCAATTAAATTCTTCAATGTATTCCCTCATAACAACTACAAAAAGTGCTAAATTACAAAATAATCGAGGTCCATTACTAGAAGAATATCGACGACATTTAATGAGTTTAGCTTATGCAATTGCAGCTGATGCAAACTCTTTATATACAACTGTTTATGAAAATCGT

>JAP09767.C|Supercontig_0000059:466989-468140

ATAAACATCTTCAAAACTATTATACAATGGTATCGGTGTAATACGTAGGAAATTTGGTAGTCTGTAATCACATATTACACCTAATCTCAATAGATTTTCATATAGTTTCTCAATCTTCACATTATTAAAACACAATGTTAACTGTGCTCCACGTTCCTCTGGACTCGATGGTGTAACAATACAATATCGATCACTATTTAGAGCTAATGAACTCTCTGTAATTAGATATTCAAGATAATTAGTTAGTTTGATTGATTTCTCCCTTAAATTATTCATACCACCACATGATTTAATAATACTGATACTGACTGTTAAAGCTGCAGCTAATAATAATGGTGGATTAGACAATCGATAGGCATCGGCACCTTTCGCTAATTCCATATTTCCAGTATAATTGAATCGTGTTTCAGTTCTATGACTCCACCAACCGGTCAGTTGAGGACCAGATATATCATTAATTATTGTATTATCACTACTATTAATATTGTTGAATTCATTAAATTTCGGTCCATAACTTGATTCTTGATGATGATGTTTTTCATGAATAAACAATCCTCCAATAGCACCGGGACTACCATTTAAATATTTATATGAACACCATACAGCCATATCAACATCCCAATCATGTAAATATAAAGGAATATTACCAACAGCATGTGCTAAATCCCATCCTACTGGACATTTACAATATTGATGACCCCATTCAGTAATTAATTTCATATTAAATAATTGTCCTGTAACATATTGTATTCCTGGTAACCAAATCAATGCAATTCTATGTTGATTTTTTTGAATTTCTTGTAAAATATCTTCATTTCTTAAACAATATTCATTAATATGTGGTTTTAGTTTAATAATACAATCATTTGGATTTAAACCATGCCAATTTATTTGTGATTCAAGTACATAATAATCACTTGGAAAGATTCCATCTTCAATTAGTATACAGCATCTCTCACCTTTTGGTCTATAGAATTTTGCAATTAATGTATGCATATTCACTGTTAAATTACAAGTCATAGTAACTTCACTTGATTTAGAATTAACAATCCATTGGGCACAATCAATAGATAATTGTTGATCACAATATGATGCTGGTAGTTCACCATAATGATAAGCTAATACACCTAAATTTCTCCATTGATTTAATAT

>JAP09769.C|Supercontig_0000161:524195-524393

TTTATACCGAACCATTTAGAAACCATTTGTACTTTTGTTGTAGCATCTCCAGACCCATTAGTTACTAAAGCAACTGGAACACGTAATTCTTTTTTATCAGGATCACATAATAATTTAAATGCTTCAGCTGCTTGAGGCAAAACTTCTAATCCACGACCAAGTACTCCGTCAATATCGAATAATAAACCGAAATTTGGCT

>JAP09774.C|Supercontig_0000040:136266-136504

AATCGACTCATATCACGTTCATATTCATTAAGTAAATTTTCTTTATGTTGAACTTGATCTTTCAATATTTCAATTCTATGAGATAATAATTCAATTGTAGTTTGACGTTCTTTTCTAACTGTTTCTCTTTCCATTTTTGGTACTTGAATAAGTGATCTTTGACCTGGTAATCGATCTAAATCTAAACGGGTTGATATTGATCGGACTAGATTTAAATAACATTCTTCTGAAGTCTGTAA

>JAP09816.C|Supercontig_0000312:455584-455860

AGTCAATAAAAACTGAAGCACAACCTATCCTGCCTCCAGGATACCAAATATCACACTGCCTTGCAGAAGGTGGAATTGGACGAGTATATTTAATTGTCAATCCTGATACTAAAGATTGTTTAGCAGCAAAAGTAGTCAATGTATATGGTCAAAGCAAGCTTCGTAGTAATACAAAAGCTGAAGTTAATGTATCACAAGTTAGGGCAGAACTTCGTCAAGAAGCTGAGTTACAAAGACGCTTAAAACATCACAATATTGCCACTTTATATGGAATCAG

>JAP09819.C|Supercontig_0000269:596888-597105

CTTAATCCATATGTCCCTACAACGCATTTTAATTTCCGTTATTTCGAAGTTGATTTGGGTAATAATCGTAAATGTTGGTGGTATGGTGGTGGAGCAGATTTAACTCCATACTATTTATTTAATGAAGATGCTAAACACTTTCATGAACAATTAAAATTAGCTTGTGATCAACATGATGAATTGTTTTATCCGCGTTTTAAAAAATGGTGTGATAATTA

>JAP09822.C|Supercontig_0000404:8803-9044

AGATTGTGGATGCTACTTTCTCTCATGATTCTCGGTGGGTAGCTGTTAGCAGCAATCATGGAACTACACATGTTTTCCCTGTTACTGCTTATGGAGGTCCGATCACTGTTCGTACGCATACTCGACCTCACGTTGTGAATCGTACTTCAAGATATCATCGATCATCCGGTTTAGAAGAGTATCATCTAACCAGACCACAACCTGAGAGAGGTGCAACTGAGTCAACTGGACTTAGCAGTAAT

>JAP09826.C|Supercontig_0000618:4770-4832

AATTTATAACAACCAAATTGTATTACTGAACCATGTCTTAATATAGCAGGACCTTCCCAACCA

>JAP09838.C|Supercontig_0000087:116973-117166

ACCTAATGATATTTTTTCACCTGCATCTGGTGGTAACATAAATACCATTAAAGCCATACCAGATATTAATAAACAAGGCACAATAAGATTAAATACATAATAAAGTGCACGTCGTTGAAGTGTAATTGCAATTTTAACATCAACAAAATCATAATCACAACATTCATAACGTTGTGCATAACGTCGAACTGAAG

>JAP09888.C|Supercontig_0000357:77836-78025

AACTATGTACACCAGGCTGTTATTATGAACGAAACTATTAAAAAAGAGTTGAAACATCAAAAAATATTCACAGAATACAGTATCAATCCTTTTAAGAAAAGTAAGATTTCACGTATTTTATAATTTGCCAGTGTATCCCTTAGCTGATAAACCAAATAGAATACAAGAAGCTGATGCTGAAGACAGTAAG

>JAP09890.C|Supercontig_0000130:1418261-1418433

ATGATGTTTACCAACACCTCTTCTACCAATTATTAATATGGAGCTACTTTCACCTTTTGAAACTGTGGATGAAATAAGGTCCTGTAAATATTTCAACTCTTTATCAAAATACAGAAGACGATGTTGATTTCCTCTTAATTTTATGCGTAGCACATCACAAATCAGATCCATTT

>JAP09912.C|Supercontig_0000005:3355483-3355646

TGAGACCACCACTACCCAGGAAGATAACATACCACATTGCATCACCATTAGCAAAGCACAGAAAGGAATATGAAGTTGGTGGCGGAAGAAAGGACCCAGATACGCGTTTAACTAGAGTTAAAGGTATTCAGCTGTGGCATGCCCATACGGGATCATTAGTTCAC

>JAP09919.C|Supercontig_0000311:887007-887299

AGTTCTGATTGTGTTACAGAAAGAGTCAGTTATCGGGAGACTCCTTACAACTCAGTAAATGTTTCATCCACTCTTGATGTGTCACATAGTCCATTAATTTCTCATCCATCTTCTAAAGATACCCTTCCTACTTGTGCTGGATGCAGACAAAACATCACAGATCAATATCTTTATCGTATTCAAGGTTTAGCTTGGCACGAATCGTGCGCTATCTGTTCGGTTTGTTCAGTTGAACTCGTAGAAGTATGTTTTATCGTCAACAAAAATGAACTGTTGTGTCACCGAGACTATGA

>JAP09920.C|Supercontig_0000043:560270-560398

AACTTCCAATTACCGAAAGATTCAAATTTTCATTCTATCAGTAGTTTATCACCAACTGCATGGAATCCAACATGGTCTGCTTTATTCTGGACAACATGGTCCGAATGGAAAAGATGTACACAAAATAAA

>JAP09936.C|Supercontig_0000312:734652-734770

TATCGAGAATCATCAAAATTCGTTTCACCACTTCGTATAGTAACATAGAATCGAGTCCACCAGTCAGCTGTAGCTAATTGTTCAGGTGTCGGTCCAGAGTGGGTTGTAACAAGATGAGC

>JAP09939.C|Supercontig_0000101:364718-364850

GATAATTGTTTATACAATCTAATTATTTGTTTACAATTTTCCGGATTAAATTGATTGATGCTGGTTAATAATTCATCGTTTGGAAAACTTGCCAGCATGTTTGCTAGGAATTCACATAGTTGATTTTTTAAAG

>JAP09953.C|Supercontig_0000243:570325-570460

ATTACCTTCTCCTTTTCAATAGGGTTTCCATGTAATGTAAGTTTTGTGAGCTTCGGTAGCTGTCTGAGTTTTATTACTTCCTGGAATGTCGCTATTTTGTTTCCATGCATATAGATGATTTTCAGGTGTTTAAGAC

>JAP09965.C|Supercontig_0000094:498436-498744

CCTGTAGGTCTGTTTGTAATGTTTGTGATTCTCTTGCCATATGATTTAAATCTTCTCTTAGTCTTGATGTTTCACGTTGTTCCGATTCTAATAGTCGACGTGTTTCGGATAAACTTGTTTGTATTATATTCAACTCATTTCTAGCAGCTGTTAATTCACTATTACGTGTAGATAGAGCTTCTTGTGTACGTGTATTTGTACGTACAGCTTCTTCATGTGCTGATCGTATATCATGAATTTCACGTAGTCTACTTGATAAATCAACTTCAGTATGTGATAATCTTTGAGCTGTTTCCTTTAAATTAATCT

>JAP09976.C|Supercontig_0000364:223531-224063

ATGAGCAGTCAAACTAGACTGGTAACTAACCATGGCTCCTTAAAGTTGCAGCCGTTTATTGTTAGTGAGGCAGTCAATTTAACACAAGATTGTGGTTTGATCAAAACGGGCGATGAATCTGATTTTTACTTATCACCATTGCCTACGCCATTGAGTGATGAAGCCCCAACCAATAATAACTCTATTCCAGGAATGCAAGAAGTTTTTGAAAAGTTGGCATCACGTCCTCACCCGTCTCCAATTTTGGCTGCCACACTTACGAAATTCGGAAGTCATATTGACGTTTGTCAAGACCCAGTTGCCAACAAGTGCTTATTGAACTCAAACACATTACATAGTCATAACGGTTCTGATTTCTCAAAAAGGTCACAAGTTTACTTAAAATCACAAGTTGAAATTTCCTCTTCATTTATTGATTTAAAAGATTTTCCATCTAGTCCTCTATCCGGTGCATTTGCTGATTTCCAGTCATTGAGATATGTCCCTCTAAGTTTAGATTGTTCATCTTCAGCAAGTATTTCACTATCAACTGC

>JAP09987.C|Supercontig_0000156:959216-959361
[truncated: 15,961,037 more chars]
